# Total Syntheses of Linear Polythiazole/Oxazole Plantazolicin A and Its Biosynthetic Precursor Plantazolicin B**

**DOI:** 10.1002/anie.201410063

**Published:** 2014-11-25

**Authors:** Zoe E Wilson, Sabine Fenner, Steven V Ley

**Affiliations:** Department of Chemistry, University of Cambridge, Lensfield RoadCambridge, CB2 1EW (UK)

**Keywords:** cyclization, heterocycles, natural products, peptides, total synthesis

## Abstract

Plantazolicin A, a linear decacyclic natural product, exhibits desirable selective activity against the causative agent of anthrax toxicity. The total synthesis of plantazolicin A and its biosynthetic precursor plantazolicin B was successfully achieved by an efficient, unified, and highly convergent route featuring dicyclizations to form 2,4-concatenated oxazoles and the mild synthesis of thiazoles from natural amino acids. This report represents the first synthesis of plantazolicin B and includes the first complete characterization data for both natural products.

Plantazolicin A (**1 a**) and its biosynthetic precursor plantazolicin B (**1 b**) represent a new class of ribosomally synthesized thiazole/oxazole natural products isolated from the soil bacterium *Bacillus amyloliquefaciens* FZB42 (Scheme 1).[1, 2] The biosynthesis of these molecules has been shown to involve the extensive post-translational modification of a 14-amino-acid peptide to give **1 b**, which has two pentaheterocyclic regions, one of which is not fully oxidized in an unusual overall linear structure. Plantazolicin B (**1 b**) undergoes dimethylation at the N-terminus to afford **1 a**.[3] Subsequent investigations by Mitchell et al. has shown that the absolute stereochemistry of **1 a** is derived from all natural L-amino acids.[4]

**Scheme 1:**
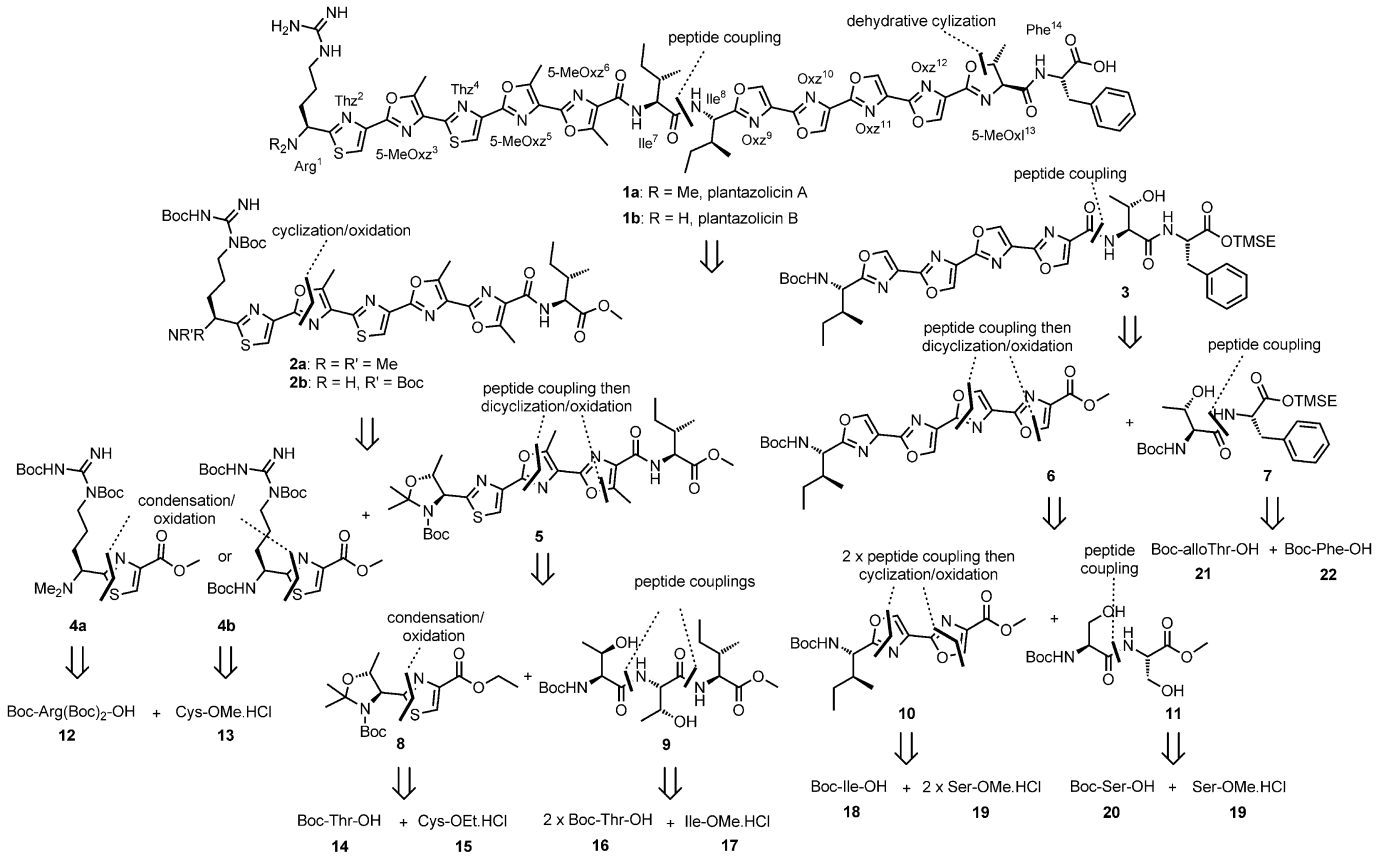
Retrosynthetic analysis of plantazolicin A and B.

Plantazolicin A (**1 a**) has been reported to exhibit antibiotic activity against related gram-positive bacteria, including, notably, the causative agent of anthrax toxicity, *Bacillus anthracis* (strain STERN), whereas **1 b** is inactive.1a, [4] The challenging linear structures of these molecules, in combination with the desirable biological activity of **1 a**, makes them attractive targets for total synthesis. Süssmuth and co-workers have recently reported the synthesis of **1 a**[5] and Mitchell et al. have reported the preparation of shortened analogues of the left-hand half, as drawn, of **1 a**.3a However, the total synthesis of the desmethyl precursor **1 b** has not been reported to date. The primary goal of our research was to develop a unified, efficient, and convergent strategy for both **1 a** and **1 b**, which we report herein.

Our strategy was based upon a late-stage peptide coupling of two equally sized fragments, **3** and either **2 a** or **2 b** (Scheme 1). Our quest to obtain the left-hand fragment of both **1 a** and **1 b** was designed based on the union of three components: the tripeptide **9** and two thiazole-containing fragments, that is, **5** and either **4 a** or **4 b**. The planned installation of arginine-derived thiazole **4 a** or **4 b** as the penultimate step of these fragments would allow a highly unified approach to the synthesis of both natural products. Initial attempts at employing a modified Hantzsch thiazole synthesis[6] for **4 a** and **4 b** were low yielding and unreliable, echoing the recently published works on similar fragments by the groups of Süssmuth and Mitchell, where the preparation of the required thioamide precursors in particular were low yielding (13 %[5] and 25 %,3a respectively) and required the use of unpleasant sulfurating reagents. Therefore it was decided to attempt a more biomimetic approach to these thiazoles, based on the condensation of an amino-acid-derived aldehyde with a cysteine ester hydrochloride, followed by oxidation of the resultant thiazolidine.[7] It was hoped that the use of **9** as a coupling partner would allow the formation of the two adjacent 5-methyl oxazole rings in a single step by using a modification of Wipf’s conditions for the cyclization of β-hydroxy amides.[8]

The synthesis of the right-hand fragment **3** was based on the union of the tetraoxazole **6** and dipeptide **7**. It was thought that a double cyclization/oxidation, this time of serine residues, could also be employed during the construction of **6**[9] after two successive coupling then cyclization/oxidation of serine residues to form the dioxazole **10**. Overall, it was proposed that both fragments could be obtained from inexpensive natural L-amino-acid starting materials, which correspond directly to those used in the biosynthesis of these natural products. The only exception to this would be the use of the L-*allo*-threonine **21** to allow a Deoxo-Fluor-mediated oxazolidine formation, as this proceeds with inversion of the configuration at the β-position of the amino acid.[8, 10]

The assembly of left-hand fragments **2 a** and **2 b** commenced with the straight forward preparation of **9** through two successive couplings using 1-hydroxybenzotriazole hydrate (HOBt) and *N*-(3-dimethylaminopropyl)-*N*′′-ethylcarbodiimide hydrochloride (EDCI) with diisopropylethylamine as a base in dichloromethane in an overall yield of 78 % (Scheme 2).

**Scheme 2:**
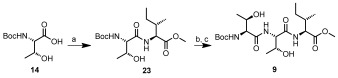
Reagents and conditions: a) Ile-OMe⋅HCl (17), HOBt, EDCI, N*i*Pr_2_Et, CH_2_Cl_2_, RT, 19 h, 99 %; b) HCl, 1,4-dioxane, RT, 23 h; c) Boc-Thr-OH (14), HOBt, EDCI, N*i*Pr_2_Et, CH_2_Cl_2_, RT, 18 h, 79 % (2 steps).

Next, attention turned to the formation of known thiazole **8** (Scheme 3). Threonine-derived Weinreb amide **24** was readily synthesized from the Boc-threonine **14** before reduction using diisobutylaluminium hydride (DIBAL-H), gave the amino aldehyde which was immediately condensed with the cysteine ethyl ester hydrochloride salt **15**, before oxidation of thiazolidine **25** using manganese dioxide to give **8** in an overall yield of 42 %. This yield was comparable to those obtained previously for **8**, but avoided the use of sulfurating reagents.[11] No epimerization of either chiral center was observed.

**Scheme 3:**
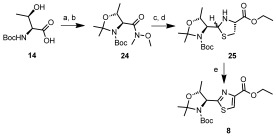
Reagents and conditions: a) CH_3_ONHCH_3_⋅HCl, EDCI, HOBt, N*i*PrEt, CH_2_Cl_2_, RT, 22 h; b) CH_3_C(OCH_3_)_2_CH_3_, PPTS, THF, reflux, 18 h, 86 % (2 steps); c) DIBAL-H, CH_2_Cl_2_, −78 °C, 1 h; d) Cys-OEt⋅HCl (15), KHCO_3_, MeOH/H_2_O/toluene (1:1:1), RT, 18 h, 83 % (2 steps); e) MnO_2_, toluene, 80 °C, 24 h, 59 %. PPTS=pyridinium *para*-toluene sulfonate, THF=tetrahydrofuran.

This approach was then applied to the assembly of the challenging arginine-derived thiazoles **4 a** and **4 b** (Scheme 4). Significant optimization determined that both **4 a** and **4 b** could be accessed by a common route. Commercially available tri-Boc-arginine **12** could be readily converted into the Weinreb amide **26** before reduction, condensation with cysteine methyl ester hydrochloride **13**, and MnO_2_-mediated oxidation to afford **4 b** in an acceptable 41 % overall yield. This approach is a marked improvement on the previous synthesis for related fragments. Removal of all nitrogen protecting groups from **4 b** allowed the selective dimethylation of the α-nitrogen atom by reductive amination using aqueous formaldehyde and sodium cyanoborohydride.[12] Reprotection of the guanidine moiety afforded **4 a** in 35 % yield (3 steps) and minor amounts (13 %) of regioisomer **28** which could feasibly be progressed further if desired.

**Scheme 4:**
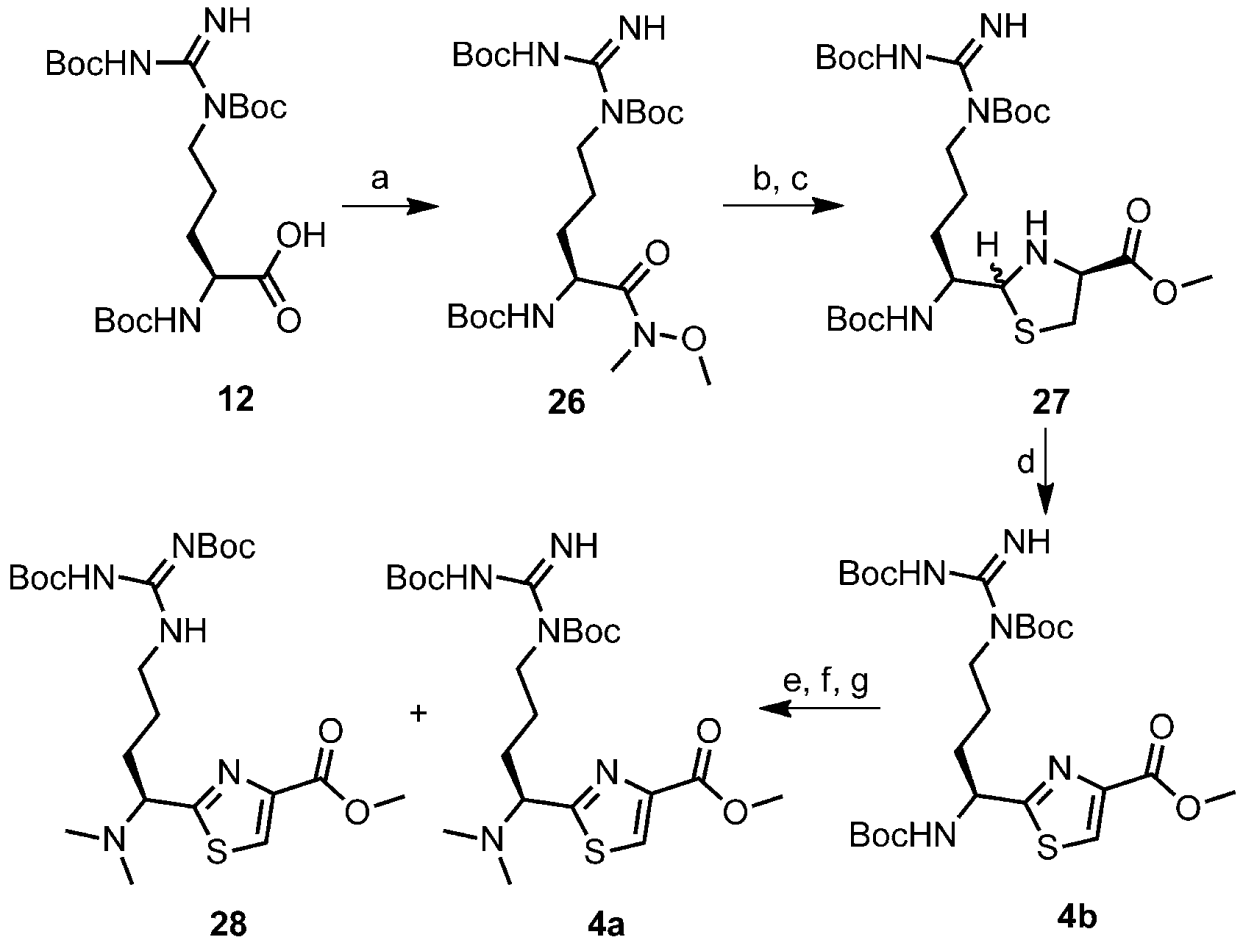
Reagents and conditions: a) CH_3_ONHCH_3_⋅HCl, HOBt, EDCI, N*i*Pr_2_Et, CH_2_Cl_2_, RT, 16 h, 96 %; b) DIBAL-H, CH_2_Cl_2_, −78 °C, 1 h; c) Cys-OMe⋅HCl (13), KHCO_3_, MeOH/H_2_O (2:1), RT, 41.5 h, 78 % (2 steps); d) MnO_2_, toluene, 80 °C, 15 h, 48 %; e) HCl, 1,4-dioxane, RT, 1 h; f) formaldehyde (37 % in H_2_O), MeOH, RT, 1 h then NaCNBH_3_, 15.5 h; g) Boc_2_O, N*i*Pr_2_Et, CH_2_Cl_2_, RT, 48 h, 35 % 4 a, 13 % 28. Boc_2_O=di-*tert*-butyl dicarbonate.

Completion of the left-hand fragment then continued with assembly of the three building blocks. Deprotection of tripeptide **9** and ester hydrolysis of **8** followed by peptide coupling led to cyclization precursor **29** in good yield (Scheme 5). It was then found that a one-pot, double cyclization/oxidation could be effected using a modification of Wipf’s conditions[8] to give 2,4-concatenated triazole **5** in an excellent yield of 64 %. To our best knowledge this is the first example of such a transformation, and represents a useful extension of Wipf’s methodology.

**Scheme 5:**
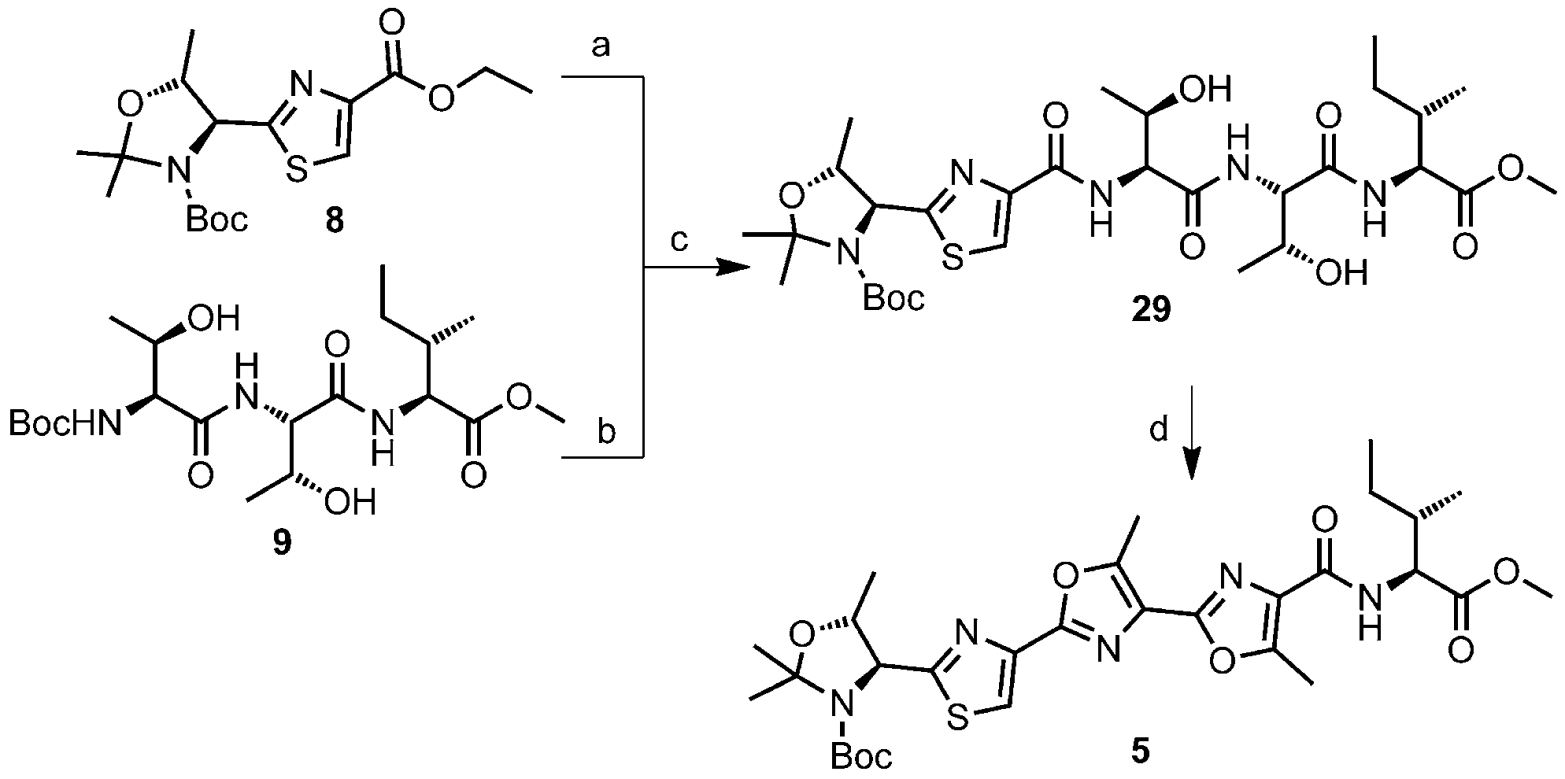
Reagents and conditions: a) LiOH⋅H_2_O, MeOH/H_2_O (3:2), RT, 3 h; b) HCl, 1,4-dioxane, 30 min, c) HOBt, EDCI, N*i*Pr_2_Et, CH_2_Cl_2_, RT, 18 h, 71 %, (3 steps); d) Deoxo-Fluor, CH_2_Cl_2_, −20 °C, 2 h, then BrCCl_3_, DBU (portionwise), 5 d, 0 °C, 64 %. Deoxo-Fluor= bis(2-methoxyethyl)aminosulfur trifluoride, DBU=1,8-diazabicyclo[5.4.0]undec-7-ene.

**Scheme 6:**
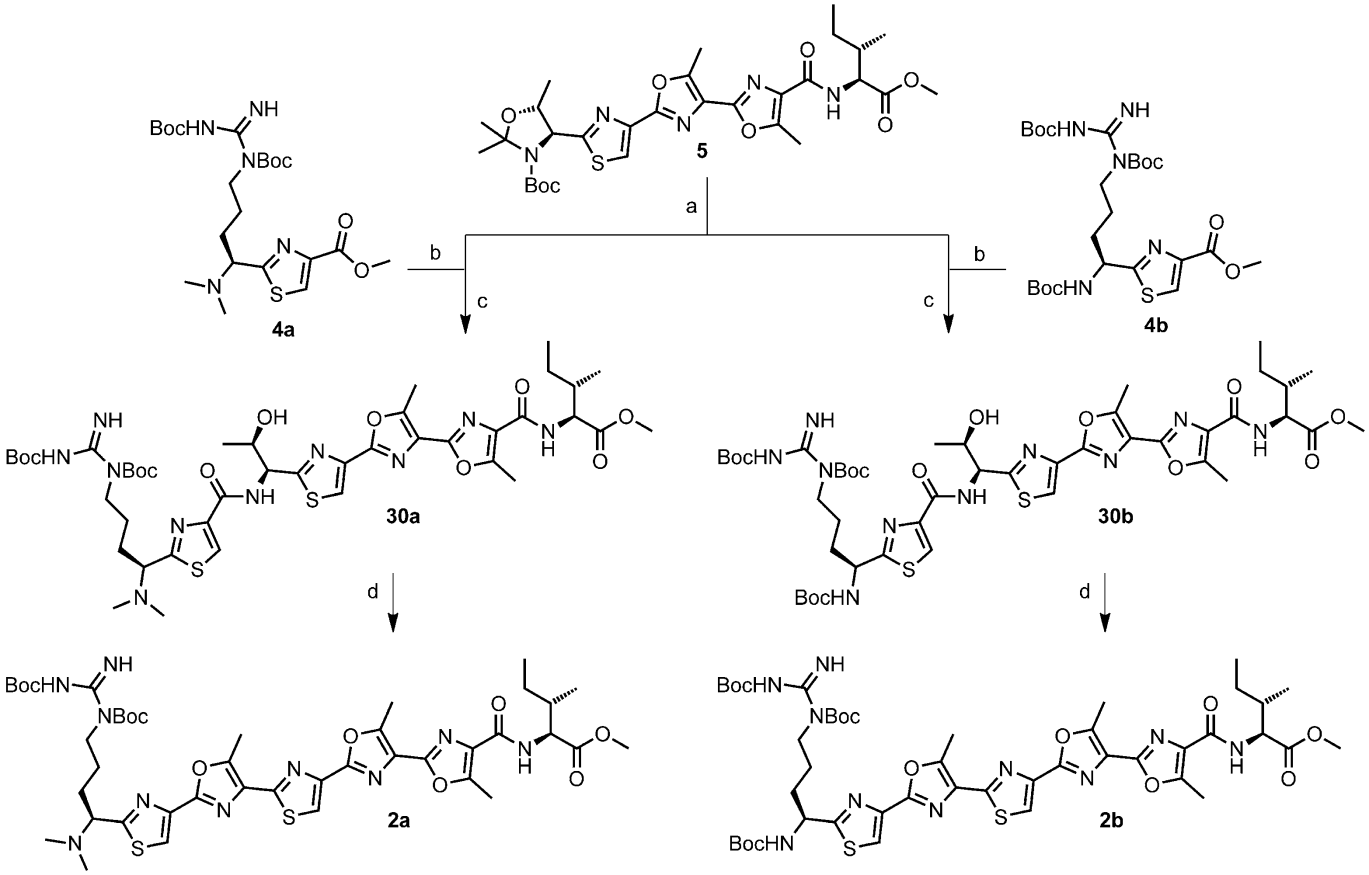
a) HCl, 1,4-dioxane, RT, 1 h; b) LiOH, THF/H_2_O (1:1), 0 °C, 1.5 h; c) HATU, N*i*Pr_2_Et, CH_2_Cl_2_, DMF, 0 °C→RT, 22 h, 61 % 30 a, 66 % 30 b; d) Deoxo-Fluor, CH_2_Cl_2_, −20 °C, 2 h then BrCCl_3_, DBU, 0 °C, 20 h (2 a)/15 h (2 b), 69 % 2 a, 92 % 2 b. DMF=*N*,*N*-dimethylformamide.

The synthesis of the common right-hand fragment **3** commenced with the preparation of the dipeptide **31** followed by one-pot cyclization/oxidation to reliably provide oxazole **32** on a multigram scale (Scheme 7). This process was then repeated to give methyl ester **10** in 78 % yield. After saponification, **10** was coupled to the deprotected serine dipeptide **11** to give **33**. A step-efficient double cyclization/oxidation could then be performed to provide tetraoxazole **6** in an excellent yield of 77 %.

**Scheme 7:**
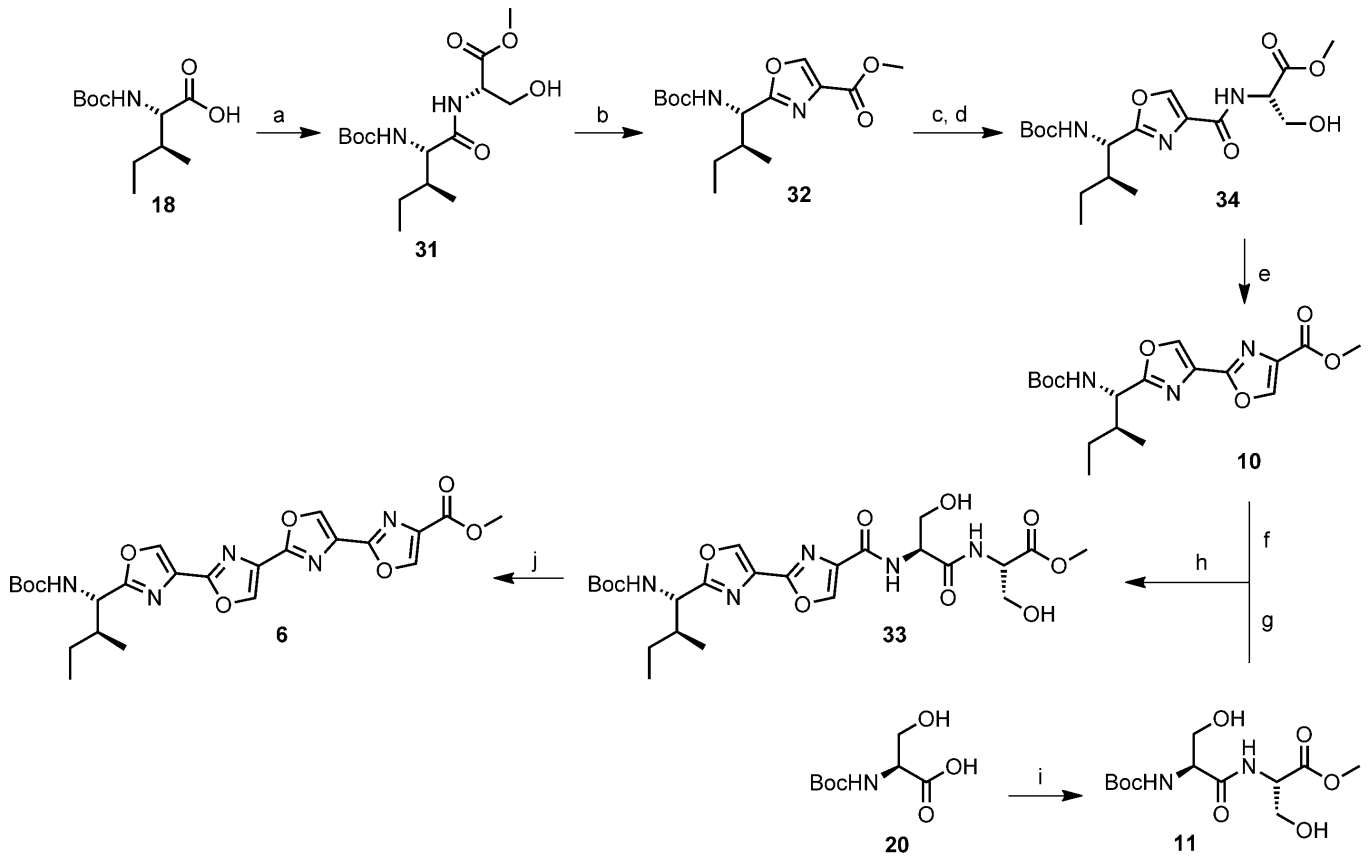
Reagents and conditions: a) Ser-OMe⋅HCl (19), HOBt, EDCI, N*i*Pr_2_Et, CH_2_Cl_2_, RT, 20 h, 91 %; b) Deoxo-Fluor, CH_2_Cl_2_, −20 °C, 30 min, then BrCCl_3_, DBU, 2-3 °C, 8 h, 81 %; c) LiOH⋅H_2_O, THF/MeOH/H_2_O (5:5:1), 0 °C→RT, 18 h; d) Ser-OMe⋅HCl (19), HOBt, EDCI, N*i*Pr_2_Et, CH_2_Cl_2_, RT, 20 h, 82 % (2 steps); e) Deoxo-Fluor, CH_2_Cl_2_, −20 °C, 30 min, then BrCCl_3_, DBU, 2–3 °C, 7 h, 78 %; f) LiOH.H_2_O, THF/MeOH/H_2_O (10:6:1), 0 °C→RT, 2 h; g) HCl, 1,4-dioxane, 0 °C→RT, 3.5 h; h) HOBt, EDCI, N*i*Pr_2_Et, CH_2_Cl_2_, RT, 20 h, 61 % (3 steps); i) Ser-OMe⋅HCl (19), HOBt, EDCI, N*i*Pr_2_Et, CH_2_Cl_2_, RT, 20 h, 88 %; j) Deoxo-Fluor, CH_2_Cl_2_, −20 °C, 45 min, then BrCCl_3_, DBU, 0 °C, 24 h, 77 %.

Boc-protected *allo*-threonine[14] **35** was coupled to the trimethyl)silylethyl (TMSE) protected phenylalanine **36** using HATU and diisopropylethylamine (Scheme 8). The resulting dipeptide, **7**, and **6** were deprotected under previously employed conditions, taking care to avoid epimerization of the *allo*-threonine during the deprotection, and coupled in 77 % yield to successfully complete the construction of the right-hand fragment **3**.

**Scheme 8:**
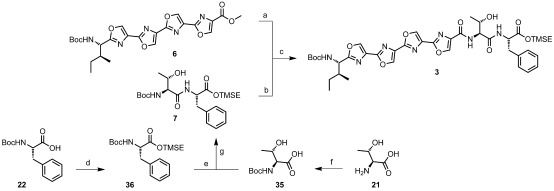
Reagents and conditions: a) LiOH⋅H_2_O, CHCl_3_/MeOH/H_2_O (9:3:1), 68 °C, 48 h; b) HCl, 1,4-dioxane, 0 °C→RT, 4 h; c) HATU, N*i*Pr_2_Et, CH_2_Cl_2_, DMF, 0 °C→RT, 18 h, 77 % (3 steps); d) (CH_3_)_3_SiCH_2_CH_2_OH, EDCI, DMAP CH_2_Cl_2_, 0 °C→RT, 18 h, 74 %; e) HCl, 1,4-dioxane, 0 °C→RT, 30 min; f) NaHCO_3_, Boc_2_O, H_2_O, MeOH, RT, 15 h; g) HATU, N*i*Pr_2_Et, CH_2_Cl_2_, 0 °C→RT, 15 h, 81 % (3 steps).

With a successful route to the coupling partners for both **1 a** and **1 b** accomplished, all that remained was the deprotection of the final coupling partners and coupling using HATU in the presence of diisopropylethylamine (Scheme 9). After partial purification the *allo*-threonine residues of coupled products **37 a** and **37 b** were cyclized using Deoxo-Fluor to give the oxazoline-containing protected natural products **38 a** (43 %) and **38 b** (35 %), respectively. Pleasingly, it was then found that removal of both the Boc and TMSE protecting groups could be effected in a single step by treatment with trifluoroacetic acid (TFA) to deliver both the natural product plantazolicin A **1 a** and its biosynthetic precursor plantazolicin B **1 b** after HPLC purification. The products were identical in all respects to published data (full characterization and comparison of synthetic and natural plantazolicin A is included in the Supporting Information of this paper). To our knowledge this is the first reported complete characterization of **1 b**.

**Scheme 9:**
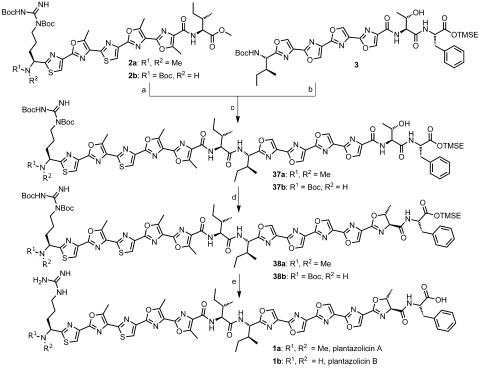
a) LiOH, THF/H_2_O (1:1), 0 °C, 2.25 h; b) HCl, 1,4-dioxane, 0 °C, 5 min, RT, 30 min; c) HATU, N*i*Pr_2_Et, CH_2_Cl_2_, DMF, 0 °C→RT, 16 h; d) Deoxo-Fluor, CH_2_Cl_2_, −20 °C, 24 h (38 a)/17 h (38 b), 43 % 38 a, 35 % 38 b; e) TFA, RT, 2 h (1 a)/1 h (1 b), 59 % (1 a), 64 % (1 b).

In conclusion, we have developed an efficient, unified strategy for the total syntheses for both thiazole/oxazole natural product plantazolicin A (**1 a**) and its biosynthetic precursor plantazolicin B (**1 b**). This was achieved through application of solution-phase peptide coupling chemistry, with step-efficient multiple oxazole formations as well as the application of a readily scalable preparation of the thiazole fragments from natural amino acids. Late-stage introduction of the N-terminus dimethylation allowed access to both natural products through a unified approach. High levels of convergence leads to **1 a** and **1 b** in 14 and 15 steps, respectively (longest linear sequence). An extensive account of our efforts towards these targets will be presented at a later date.
