# Supplementary material for: Total Syntheses of Linear Polythiazole/Oxazole Plantazolicin A and Its Biosynthetic Precursor Plantazolicin B
Source: Angew Chem Int Ed Engl. 2014 Nov 25;54(4):1284–8. doi: 10.1002/anie.201410063 (PMC4464531; doi:10.1002/anie.201410063)
Supplement: Supplementary file 1 [file anie0054-1284-sd1.pdf]

Angewandte Chemie
Eine Zeitschrift der Gesellschaft Deutscher Chemiker

Supporting Information

© Wiley-VCH 2015

69451 Weinheim, Germany

Total Syntheses of Linear Polythiazole/Oxazole Plantazolicin A and Its Biosynthetic Precursor Plantazolicin B**

*Zoe E. Wilson, Sabine Fenner, and Steven V. Ley**

anie_201410063_sm_miscellaneous_information.pdf

Table of contents

		Experimental	NMR
1	General experimental details	2	
2	Numbering of compounds	2	
	2.1 Atom numbering of key residues and their abbreviations	3	
	2.2 Residue numbering employed for the natural products	3	
3	Synthesis of *N*-Boc-Thr^{A}-Thr^{B}-Ile^{7}-OMe **9**	3	
	3.1 *N*-Boc-Thr-Ile^{7}-OMe **23**	4	34
	3.2 *N*-HCl-Thr-Ile^{7}-OMe **39**	4	37
	3.3 *N*-Boc-Thr^{A}-Thr^{B}-Ile^{7}-OMe **9**	5	40
4	Synthesis of Boc-N-(CMe_2)-O-Thr-Thz^{4}-CO_2Et **8**	5	
	4.1 *N*-Boc-Thr-N(Me)OMe **40**	5	43
	4.2 Boc-N-(CMe_2)-O-Thr-N(Me)OMe **24**	6	46
	4.3 Boc-N-(CMe_2)-O-Thr-Thl^{4}-CO_2Et **25**	6	49
	4.4 Boc-*N*-(CMe_2)-*O*-Thr-Thz^{4}-CO_2Et **8**	7	52
5	Synthesis of *N*-Boc-Arg^{1}(Boc)$_2$-Thz^{2}-CO_2Me **4b** and *N*-Me$_2$-Arg^{1}(Boc)$_2$-Thz^{2}-CO_2Me **4a**	8	
	5.1 *N*-Boc-Arg^{1}(Boc)$_2$-N(Me)OMe **26**	8	55
	5.2 *N*-Boc-Arg^{1}(Boc)$_2$-Thl-CO_2Me **27**	8	58
	5.3 *N*-Boc-Arg^{1}(Boc)$_2$-Thz^{2}-CO_2Me **4b**	9	61
	5.4 *N*-Me$_2$-Arg^{1}(Boc)$_2$-Thz^{2}-CO_2Me **4a**	10	64
6	Synthesis of Boc-N-(CMe_2)-O-Thr-Thz^{4}-5-MeOxz^{5}-5-MeOxz^{6}-Ile^{7}-OMe **5**	11	
	6.1 Boc-*N*-(CMe_2)-O-Thr^{A}-Thz^{4}-Thr^{B}-Thr^{C}-Ile^{7}-OMe **29**	11	70
	6.2 Boc-*N*-(CMe_2)-*O*-Thr-Thz^{4}-5-MeOxz^{5}-5-MeOxz^{6}-Ile^{7}-OMe **5**	12	73
7	Synthesis of *N*-Me$_2$-Arg^{1}(Boc)$_2$-Thz^{2}-5-MeOxz^{3}-Thz^{4}-5-MeOxz^{5}-5-MeOxz^{6}-Ile^{7}-OMe **2a**	13	
	7.1 *N*-Me$_2$-Arg^{1}(Boc)$_2$-Thz^{2}-Thr-Thz^{4}-5-MeOxz^{5}-5-MeOxz^{6}-Ile^{7}-OMe **30a**	13	76
	7.2 *N*-Me$_2$-Arg^{1}(Boc)$_2$-Thz^{2}-5-MeOxz^{3}-Thz^{4}-5-MeOxz^{5}-5-MeOxz^{6}-Ile^{7}-OMe **2a**	14	79
8	Synthesis of *N*-Boc-Arg^{1}(Boc)$_2$-Thz^{2}-5-MeOxz^{3}-Thz^{4}-5-MeOxz^{5}-5-MeOxz^{6}-Ile^{7}-OMe **2b**	15	
	8.1 *N*-Boc-Arg^{1}(Boc)$_2$-Thz^{2}-Thr-Thz^{4}-5-MeOxz^{5}-5-MeOxz^{6}-Ile^{7}-OMe **30b**	15	82
	8.2 *N*-Boc-Arg^{1}(Boc)$_2$-Thz^{2}-5-MeOxz^{3}-Thz^{4}-5-MeOxz^{5}-5-MeOxz^{6}-Ile^{7}-OMe **2b**	16	85
9	Synthesis of *N*-Boc-Ile^{8}-Oxz^{9}-Oxz^{10}-Oxz^{11}-Oxz^{12}-CO_2Me **6**	17	
	9.1 General procedure A:	17	
	9.2 General procedure B:	17	
	9.3 *N*-Boc-Ile^{8}-Ser-OMe **31**	18	88
	9.4 *N*-Boc-Ile^{8}-Oxz^{9}-CO_2Me **32**	18	89
	9.5 *N*-Boc-Ile^{8}-Oxz^{9}-Ser-OMe **34**	18	90
	9.6 *N*-Boc-Ile^{8}-Oxz^{9}-Oxz^{10}-CO_2Me **10**	19	93
	9.7 *N*-Boc-Ser^{A}-Ser^{B}-OMe **11**	19	96
	9.8 *N*-Boc-Ile^{8}-Oxz^{9}-Oxz^{10}-Ser^{A}-Ser^{B}-OMe **33**	20	97
	9.9 *N*-Boc-Ile^{8}-Oxz^{9}-Oxz^{10}-Oxz^{11}-Oxz^{12}-CO_2Me **6**	21	100
10	Synthesis of *N*-Boc-Ile^{8}-Oxz^{9}-Oxz^{10}-Oxz^{11}-Oxz^{12}-L-*allo*-Thr-Phe^{14}OTMSE **3**	22	
	10.1 *N*-Boc-Phe^{14}OTMSE **36**	22	103
	10.2 *N*-Boc-L-*allo*-Thr-Phe^{14}OTMSE 7	22	106
	10.3 *N*-Boc-Ile^{8}-Oxz^{9}-Oxz^{10}-Oxz^{11}-Oxz^{12}-L-*allo*-Thr-Phe^{14}OTMSE **3**	23	109
11	Synthesis of Plantazolicin A **1a**	25	
	11.1 *N*-Me$_2$-Arg^{1}(Boc)$_2$-Thz^{2}-5-MeOxz^{3}-Thz^{4}-5-MeOxz^{5}-5-MeOxz^{6}-Ile^{7}-Ile^{8}-Oxz^{9}-Oxz^{10}-Oxz^{11}-Oxz^{12}-L-*allo*-Thr-Phe^{14}OTMSE **37a**	25	112
	11.2 *N*-Me$_2$-Arg^{1}(Boc)$_2$-Thz^{2}-5-MeOxz^{3}-Thz^{4}-5-MeOxz^{5}-5-MeOxz^{6}-Ile^{7}-Ile^{8}-Oxz^{9}-Oxz^{10}-Oxz^{11}-Oxz^{12}-5-MeOxl^{13}-Phe^{14}OTMSE **38a**	26	112
	11.3 Plantazolicin A **1a**	27	115
12	Synthesis of Plantazolicin B **1b**	28	
	12.1 *N*-Boc$_2$-Arg^{1}(Boc)$_2$-Thz^{2}-5-MeOxz^{3}-Thz^{4}-5-MeOxz^{5}-5-MeOxz^{6}-Ile^{7}-Ile^{8}-Oxz^{9}-Oxz^{10}-Oxz^{11}-Oxz^{12}-L-*allo*-Thr-Phe^{14}OTMSE **37b**	28	118
	12.2 *N*-Boc$_2$-Arg^{1}(Boc)$_2$-Thz^{2}-5-MeOxz^{3}-Thz^{4}-5-MeOxz^{5}-5-MeOxz^{6}-Ile^{7}-Ile^{8}-Oxz^{9}-Oxz^{10}-Oxz^{11}-Oxz^{12}-5-MeOxl^{13}-Phe^{14}OTMSE **38b**	29	118
	12.3 Plantazolicin B **1b**	30	121
13	References	32	
14	NMR of synthesized compounds	33	
15	Comparison of synthesized and natural plantazolicin A	124	

1. General Details

All reactions were carried out under argon atmosphere using oven-dried glassware, and were monitored using a combination of TLC, NMR spectroscopy and HRMS. Unless otherwise stated, reagents were obtained from commercial sources and used without further purification. Amino acids were all of the natural (L) enantiomeric form unless otherwise stated. Solvents were freshly distilled over calcium hydride and lithium aluminium hydride (tetrahydrofuran and diethyl ether) or calcium hydride (dichloromethane, methanol, hexane, toluene, ethyl acetate and 40-60 petroleum ether). Additional anhydrous solvents were obtained from commercial sources and used directly (chloroform, *N,N*-dimethylformamide). Boc-Arg(Boc)$_2$-OH is protected on the Nα, Nω and Nδ positions of the arginine.

Thin layer chromatography (TLC) was carried out using 0.25 mm thick glass backed Merck TLC Silica gel 60 F_{254} plates which were visualised using ultraviolet radiation and aqueous acidic ammonium molybdate (VII) solution, potassium permanganate solution or ninhydrin solution. Preparatory Thin Layer Chromatography was carried out using Analtech 20 x 20 cm UNIPLATE™ Silica gel GF (preparative layer with UV254) plates of either 500, 1000 or 2000 microns depth and were visualised using UV. Flash column chromatography was carried out using high-purity grade silica gel (Merck grade 9385) with a pore size 60 Å and 230–400 mesh particle size).

Infrared Spectroscopy was recorded using a PerkinElmer Spectrum One FT-IR spectrometer using Universal ATR sampling accessories. Absorbances were recorded in the range 4000-650 cm^{-1}.

Nuclear Magnetic Resonance Spectroscopy (NMR) spectra were recorded using either a 400 MHz DPX-400 Dual Spectrometer, a 500 MHz Dual $^{13}C/^{1}H$ Cyroprobe Spectrometer or a 600 MHz Avance 600 BBI spectrometer as indicated. Unless otherwise stated, all samples were run at room temperature in deuterated solvent, with chemical shift (δ) reported to the nearest 0.01 (^{1}H)/0.1 (^{13}C) ppm , relative to the residual protic solvent; δ($CDCl_3$) = 7.26 (^{1}H)/77.16 (^{13}C) ppm, δ(MeOD) = 3.31 (^{1}H)/49.1 (^{13}C) ppm or δ(d_6-DMSO) 2.50 (^{1}H)/39.51 (^{13}C) ppm. All carbon NMRs were run with broadband proton decoupling. The ^{13}C NMR spectrum of synthetic natural products plantazolicin A **1a** and plantazolicin B **1b** and compound **38b** were obtained using a Uniform Driven Equilibrium Fourier Transform (UDEFT) sequence with a 360ms acquisition time, with a 5s d1 (relaxation delay).[1] Multiplicity of a signal in ^{1}H NMR is indicated by: s = singlet, d = doublet, t = triplet or, q = quartet, quint = quintet, m = multiplet, or a combination thereof. Multiplets are reported as the range of ppm values covered by the signals, otherwise the centre of the signal is given. Coupling constants, *J*, are quoted in Hz and recorded to the nearest 0.1 Hz. Assignments were confirmed using Distortionless Enhanced Polarisation Transfer NMR (DEPT 135) and two dimensional NMR (^{1}H–^{1}H Correlation Spectroscopy (COSY), Heteronuclear Single Quantum Coherence (HSQC) or Heteronuclear multiple-quantum correlation spectroscopy (HMQC) and Heteronuclear Multiple Bond Correlation (HMBC)) experiments gave information used to assign both the ^{1}H NMR and ^{13}C NMR spectra, and are provided in the supporting information when the compound is not literature known.

High Resolution Mass Spectrometry (HRMS) was performed using either a Waters Micromass LCT Premier spectrometer, or by Mr. Paul Skelton using a Bruker Bioapex 47e FTICR spectrometer, using positive Electron Spray Ionisation (ESI+). Masses are given in m/z-units, and the reported values all lie within Δ5 ppm of calculated values.

Melting points (mp) were measured using a Stanford Research Systems OptiMelt automated melting point system using a gradient of 1 °C/min, and are uncorrected.

Specific Optical Rotation was recorded on a Perkin-Elmer Model 343 digital polarimeter, using a Na/Hal lamp set at 589 nm and with a path length of 100 mm. All [α_D] values were measured using spectroscopy grade solvent at the specified concentration (in gcm^{-3}) and temperature, with units of $10^{-1}cm^2g^{-1}$.

High Pressure Liquid Chromatography was run on an Agilent Technologies 1100 Series HPLC with manual collection monitoring at 260 and 254 nm, using a Thermo Scientific Betasil C18 column (250 mm x 10 mm; pore size: 100 Å, particle size: 5 µm) at a flow rate of 4 mL min^{-1} in 10 mM aqueous ammonium bicarbonate with acetonitrile with the following solvent gradient:

Time	10 mM aq. NH_4HCO_3 (%)	Acetonitrile (%)
0 min	40	60
6 min	40	60
26 min	30	70
27 min	5	95
31 min	5	95
32 min	40	60
35 min	0	0

2. Numbering of compounds

The compound numbering system used is based on IUPAC conventions. Each amino acid is numbered individually, starting from the carboxyl group and proceeding up the side chain. Position labels thus refer first to the individual amino acid, and then to the position on it, with the atom to be defined (C or H) underlined. Non-amino acid derived functionality is numbered based on the position from which it branches. The entire branch is thus given the label of the position from which it begins, appended with a prime, and each position within the chain is further denoted by a lowercase letter.

2.1 Atom numbering of key residues and their abbreviations

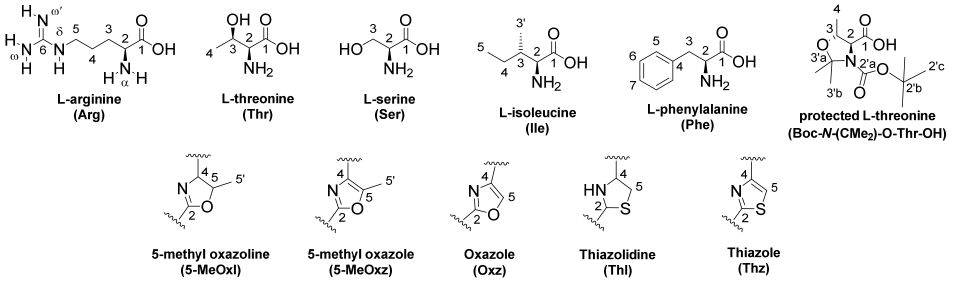

2.2 Residue numbering employed for the natural products

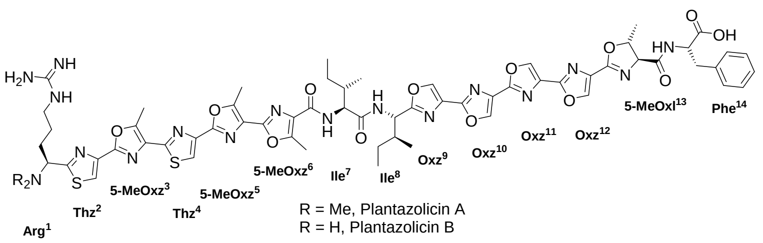

3. Synthesis of *N*-Boc-Thr^A-Thr^B-Ile^7-OMe 9

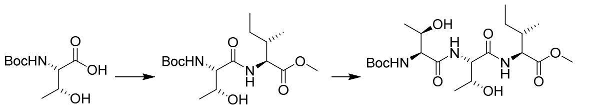

14 23 9

3.1. *N*-Boc-Thr-Ile^7-OMe 23

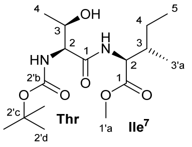

Diisopropylethylamine (15.9 mL, 91.2 mmol) was added to a solution of Boc-Thr-OH (10.00 g, 45.6 mmol) and Ile-OMe.HCl (8.28 g, 45.6 mmol) in dichloromethane (140 mL) and the reaction mixture stirred at room temperature for 2 min before 1-hydroxybenzotriazole hydrate (HOBt) (7.40 g, 54.7 mmol) was added. After a further 2 min at room temperature *N*-(3-dimethylaminopropyl)-*N′*-ethylcarbodiimide hydrochloride (EDCI) (10.50 g, 54.7 mmol) was added and the reaction mixture stirred at room temperature for 17 h. Saturated ammonium chloride (200 mL) was added and the reaction mixture extracted with dichloromethane (3 x 200 mL). The combined organic extracts were washed with aqueous hydrochloric acid (200 mL, 3M), saturated sodium bicarbonate (200 mL) and saturated sodium chloride (200 mL) and then dried over magnesium sulfate and the solvent was removed *in vacuo*. The resulting oil was purified by flash chromatography using 40-60-petroleum ether:diethyl ether (1:1) eluent to afford the *title compound* **23** (15.61 g, 45.1 mmol, 99%) as a white foam.

R*f* = 0.46 [40-60 Petroleum ether:diethyl ether = 1:1]; **$[\alpha]_D^{25.0}$** -98.9(c 1.0, $CHCl_3$); **IR** (neat/cm^{-1}) 3327, 2969, 2935, 2879, 1742, 1657, 1513, 1367, 1164; **^{1}H-NMR** (600 MHz, $CDCl_3$) δ = 7.18 (d, *J* = 8.7 Hz, 1H, Ile^7-C2NH), 5.61 (d, *J* = 7.9 Hz, 1H, Thr-C2NH), 4.48 (dd, *J* = 8.7, 5.0 Hz, 1H, Ile^7-C2H), 4.26 (qt, *J* = 5.9, 2.8 Hz, 1H, Thr-C3H), 4.08 (dd, *J* = 7.7, 2.7 Hz, 1H, Thr-C2H), 3.81 – 3.77 (m, 1H, Thr-C3OH), 3.68 (s, 3H, Ile^7-C1′aH_3), 1.87 (dtt, *J* = 11.4, 6.2, 3.2 Hz, 1H, Ile^7-C3H), 1.41 – 1.33 (m, 10H, 3 x Thr-C2′dH_3 and Ile^7-C4*H*H), 1.18 – 1.10 (m, 4H, Thr-C4H_3 and Ile^7-C4*H*H), 0.85 (t, *J* = 7.5 Hz, 6H, Ile^7-C5H_3 and Ile^7-C3′aH_3); **^{13}C-NMR** (151 MHz, $CDCl_3$) δ = 172.1 (quat., Ile^7-C1), 171.5 (quat., Thr-C1), 156.5 (quat., Thr-C2′b), 80.3 (quat., Thr-C2′c), 66.8 (CH, Thr-C3), 57.9 (CH, Thr-C2), 56.7 (CH, Ile^7-C2), 52.1 (CH_3, Ile^7-C1′a), 37.5 (CH, Ile^7-C3), 28.3 (CH_3, 3 x Thr-C2′d), 25.0 (CH_2, Ile^7-C4), 18.0 (CH_3, Thr-C4), 15.6 (CH_3, Ile^7-C5 or Ile^7-C3′a), 11.5 (CH_3, Ile^7-C5 or Ile^7-C3′a); **HRMS (ESI)** found: 369.1988 ($[MNa]^+$ $C_{16}H_{30}O_6N_2Na$ requires 369.1996).

3.2. *N*-HCl-Thr-Ile^7-OMe 39

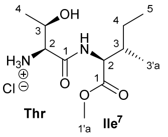

23 (7.81 g, 22.6 mmol) was taken up in 1,4-dioxane (75 mL) and anhydrous hydrochloric acid (28.2 mL, 4 M in 1,4-dioxane, 112.8 mmol) was added. The reaction mixture was stirred at room temperature for 23 h before the solvent was removed *in vacuo*. The reaction mixture was twice dissolved in ethanol (100 mL) and the solvent removed *in vacuo* to afford a white foam **39** which was used directly in the next step without further purification.

^{1}H-NMR (600 MHz, $CDCl_3$) δ = 8.35 (s, 1H, Ile^7-NH), 8.04 (s, 3H, Thr-C2NH_3Cl), 4.59 – 4.43 (m, 1H, Thr-C2H), 4.39 (t, *J* = 5.5 Hz, 1H, Ile-C2H), 4.19 (s, 1H, Thr-C3H), 3.74 – 3.57 (m, 3H, Ile^7-C1′aH_3), 1.97 (q, *J* = 7.2, 6.5 Hz, 1H, Ile^7-C3H), 1.59 – 1.40 (m, 4H, Thr-C4H_3 and Ile^7-C4*H*H), 1.30 (dq, *J* = 15.2, 7.9 Hz, 1H, Ile^7-C4*H*H), 0.95 (d, *J* = 6.6 Hz, 3H, Ile^7-C3′aH_3), 0.89 (t, *J* = 7.3 Hz, 3H, Ile^7-C5H_3). **^{13}C-NMR** (151 MHz, $CDCl_3$) δ = 171.7 (quat., Ile^7-C2′a), 167.8 (quat., Thr-C1), 67.1 (CH, Thr-C3), 58.5 (CH, Thr-C2), 58.1 (CH, Ile^7-C2), 52.2 (CH_3, Ile^7-C1′a), 36.8 (CH, Ile^7-C3), 25.5 (CH_2, Ile^7-C4), 19.2 (CH_3, Thr-C4), 15.7 (CH_3, Ile^7-C3′a), 11.8 (CH_3, Ile^7-C5).

3.3. *N*-Boc-Thr^{A}-Thr^{B}-Ile^{7}-OMe 9

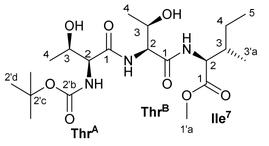

39 was taken up in dichloromethane (70 mL) and diisopropyl ethylamine (7.9 mL, 45.2 mmol) added. The reaction mixture was stirred at room temperature for 5 min before HOBt (3.66 g, 27.1 mmol) was added. After a further 5 minutes at room temperature EDCI (5.20 g, 27.1 mmol) was added and the reaction mixture was stirred at room temperature for 18 h before saturated ammonium chloride (100 mL) was added. The reaction mixture was extracted with dichloromethane (3 x 100 mL) and the combined organic extracts washed with aqueous hydrochloric acid (100 mL, 3M), saturated sodium bicarbonate (100 mL) and saturated sodium chloride (100 mL) and was dried over magnesium sulfate before the solvent was removed *in vacuo* to give an oil which was purified *via* flash chromatography using diethyl ether eluent to afford the *title compound* **9** (8.03 g, 17.9 mmol, 79%) as a white foam.

$\mathbf{R_f}$ = 0.08 [diethyl ether]; $[\alpha]_D^{25.0}$ -44.1(c 1.0, $CHCl_3$); **IR** (neat/cm^{-1}) 3308, 2973, 2937, 1742, 1719, 1645, 1513, 1367, 1163; **^{1}H-NMR** (600 MHz, $CDCl_3$) δ = 7.60 (d, J = 8.3 Hz, 1H, Thr^{B}-C2NH), 7.56 (d, J = 8.3 Hz, 1H, Ile^{7}-C2NH), 5.85 (d, J = 8.4 Hz, 1H, Thr^{A}-C2NH), 4.56 (dd, J = 8.4, 3.9 Hz, 1H, Thr^{B}-C2H), 4.47 – 4.41 (m, 1H, Ile^{7}-C2H), 4.28 – 4.23 (m, 1H, Thr^{A}-C2H), 4.22 – 4.11 (m, 3H, Thr^{A}-C3H, Thr^{B}-C3H, Thr$^{A/B}$-C3OH), 4.02 (d, J = 41.3 Hz, 1H, Thr$^{A/B}$-C3OH), 3.64 (s, 3H, Ile^{7}-C1′aH$_3$), 1.83 (dq, J = 12.0, 5.8 Hz, 1H, Ile^{7}-C3H), 1.36 (s, 10H, 3 x Thr^{A}-C2′cH$_3$ and Ile^{7}-C4*H*H), 1.21 – 1.04 (m, 7H, Ile^{7}-C4*H*H, Thr^{A}-C4H$_3$, Thr^{B}-C4H$_3$), 0.85 – 0.78 (m, 6H, Ile^{7}-C5H$_3$, Ile^{7}-C3′aH$_3$); **^{13}C-NMR** (151 MHz, $CDCl_3$) δ = 172.1 (quat., Ile^{7}-C1), 171.7 (quat., Thr^{A}-C1), 170.5 (quat., Thr^{B}-C1), 156.2 (quat., Thr^{A}-C2′b), 80.2 (quat., Thr^{A}-C2′c), 67.6 (CH, Thr$^{A/\ B}$-C2), 67.3 (CH, Thr$^{A/B}$-C2), 59.0 (CH, Thr^{A}-C3), 57.9 (CH, Thr^{B}-C3), 56.9 (CH, Ile^{7}-C2), 52.0 (CH_3, Ile-C1′a), 37.3 (CH, Ile^{7}-C3), 30.3 (CH_3, from 3 x Thr^{A}-C2′d), 28.3 (CH_3, from 3 x Thr^{A}-C2′d), 25.1 (CH_2, Ile^{7}-C4), 18.5 (CH_3, Thr$^{A/B}$-C4), 18.4 (CH_3, Thr$^{A/B}$-C4), 15.5 (CH_3, Ile^{7}-C5 or Ile^{7}-C3′a), 11.5 (CH_3, Ile^{7}-C5 or Ile^{7}-C3′a); **HRMS** (ESI) found: 448.2645 ($[M+H]^+$ $C_{20}H_{38}O_8N_3$ requires 448.2659).

4. Synthesis of Boc-N-(CMe_2)-O-Thr-Thz^{4}-CO_2Et 8

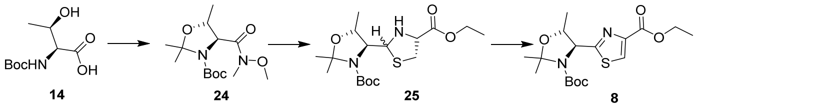

4.1 *N*-Boc-Thr-N(Me)OMe 40

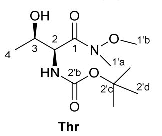

Boc-Thr-OH (10.0 g, 46.0 mmol) and *N*,*O*-dimethylhydroxylamine hydrochloride (4.45 g, 46.0 mmol) were taken up in dichloromethane (250 mL) and diisopropylethylamine (15.9 mL, 91.0 mmol) was added and the reaction mixture stirred for 2 min before the addition of HOBt (7.40 g, 55.0 mmol). After a further 10 min stirring at room temperature, EDCI (10.49 g, 55.0 mmol) was added and the reaction mixture stirred at room temperature for 22 h. Saturated ammonium chloride (200 mL) was added and the reaction mixture extracted with dichloromethane (3 x 200 mL). The combined organic extracts were washed with aqueous hydrochloric acid (200 mL, 3M), saturated sodium bicarbonate solution (200 mL), and saturated sodium chloride (200 mL) before drying over magnesium sulfate and removing the solvent *in vacuo* to afford the *title compound* **40** as a colourless oil, which was placed directly into the next step without further purification.

^{1}H-NMR (600 MHz, $CDCl_3$) δ = 5.61 – 5.46 (m, 1H, Thr-C2NH), 4.70 – 4.54 (m, 1H, Thr-C2H), 4.05 (q, J = 6.7 Hz, 1H, Thr-C3H), 3.73 (s, 3H, Thr-C1′bH$_3$), 3.18 (s, 3H, Thr-NC1′aH$_3$), 3.12 (s, 1H, Thr-C3OH), 1.40 (s, 9H, Thr-C2′dH$_3$), 1.18 (d, J = 6.5 Hz, 3H, Thr-C4H$_3$). **^{13}C-NMR** (151 MHz, $CDCl_3$) δ = 172.3 (quat., Thr-C1), 156.1 (quat., Thr-C2′b), 79.8 (quat., Thr-C2′c), 67.7(CH, Thr-C3), 61.7 (CH_3, Thr-C1′b), 54.2 (CH, Thr-C2), 32.0 (CH_3, Thr-C1′a), 28.4 (3 x CH_3, Thr-C2′d), 19.4 (CH_3, Thr-C4). The ^{1}H and ^{13}C NMR data obtained was in agreement with that reported in the literature.[2]

4.2. Boc-N-(CMe$_2$)-O-Thr-N(Me)OMe 24

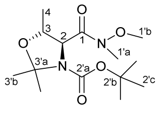

Pyridinium *para*-toluene sulphonate (2.31 g, 9.2 mmol) was added to a solution of **40** (11.96 g, 46.0 mmol) and 2,2-dimethoxy propane (89.2 mmol, 460.0 mmol) in tetrahydrofuran (250 mL) and the reaction mixture heated to reflux for 18 h. The solvent was removed *in vacuo*, water (200 mL) added and the reaction mixture extracted with ethyl acetate (3 x 200 mL). The combined organic extracts were washed with saturated sodium chloride (250 mL) and the solvent removed *in vacuo* with the resultant oil purified *via* flash chromatography using gradient elution with 40-60 petroleum ether:diethyl ether (2:1 to 1:1) eluent to afford the *title compound* **24** (11.84 g, 39.2 mmol, 86%) as a colourless oil.

R_f = 0.42 [40-60 Petroleum ether:diethyl ether = 1:1]; **$[\alpha]_D^{25.0}$** = -10.1(c 1.4, $CHCl_3$) (literature[2] $[\alpha]_D^{20.0}$ -9.81(c 1.4, $CHCl_3$)); **^{1}H-NMR** (600 MHz, $CDCl_3$) δ = 4.24 (d, J = 6.9 Hz, 0.4H, Thr-C2H), 4.14 (d, J = 6.9 Hz, 0.6H, Thr-C2H), 3.88 (dp, J = 25.1, 6.2 Hz, 1H, Thr-C3H), 3.54 (s, 1.2H, Thr-C1′bH$_3$), 3.49 (s, 1.8H, Thr-C1′bH$_3$), 2.97 (s, 3H, Thr-C1′aH$_3$), 1.39 (s, 2H, from 2 x Thr-C3′bH$_3$), 1.37 – 1.31 (m, 4H, from 2 x Thr-C3′bH$_3$), 1.22 (s, 3H, from Thr-C2′cH$_3$), 1.16 – 1.10 (m, 9H, Thr-C4H$_3$ and 6H from Thr-C2′cH$_3$). **^{13}C-NMR** (151 MHz, $CDCl_3$) δ = 170.7 (quat., Thr-C1), 170.0 (quat., Thr-C1), 151.5 (quat., Thr-C2′a), 150.8 (quat., Thr-C2′a), 94.5 (quat., Thr-C3′a), 94.0 (quat., Thr-C3′a_), 79.8 (quat., Thr-C2′b), 79.6 (quat., Thr-C2′b), 74.2 (CH, Thr-C3), 73.8 (CH, Thr-C3), 62.94 (CH, Thr-C2), 62.86 (CH, Thr-C2), 60.8 (CH_3, Thr-C1′b), 32.0 (CH_3, Thr-C1′a), 28.07 (CH_3, Thr-C2′c), 28.06 (CH_3, Thr-C2′c), 27.9 (CH_3, Thr-C2′c), 26.6 (CH_3, Thr-C2′c), 24.8 (CH_3, Thr-C3′b), 23.7 (CH_3, Thr-C3′b), 19.2 (CH_3, Thr-C4), 19.1 (CH_3, Thr-C4). The data obtained was in agreement with that reported in the literature.[2]

4.3. Boc-N-(CMe$_2$)-O-Thr-Thl^4-CO$_2$Et 25

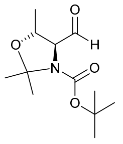

41

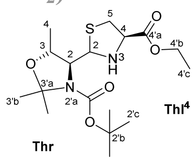

25

Diisobutylaluminium hydride (11.3 mL, 1M in dichloromethane, 11.3 mmol) was added to a solution of **24** (1.7 g, 5.6 mmol) in dichloromethane (70 mL) at -78 °C and the reaction mixture stirred at this temperature for 30 min. The reaction mixture was removed from the ice bath and quenched by addition of saturated sodium potassium tartrate (100 mL) and stirred at room temperature for 2 h. The reaction mixture was extracted with dichloromethane (3 x 100 mL) and the combined organic extracts were washed with saturated sodium chloride (100 mL) and dried over magnesium sulfate before the solvent was removed *in vacuo* to afford a crude yellow oil (**41**) which was taken up in toluene (35 mL) and methanol (35 mL) and a solution of cysteine ethyl ester hydrochloride (1.04 g, 5.6 mmol) and potassium bicarbonate (0.50 g, 5.0 mmol) in water (35 mL) was added. The biphasic reaction mixture was stirred vigorously at room temperature for 15 h before the toluene and methanol was removed *in vacuo*. Water (10 mL) was added and the mixture extracted with ethyl acetate (3 x 50 mL). The combined organic extracts were washed with saturated sodium chloride (50 mL) and were dried over magnesium sulfate before the solvent was removed *in vacuo*. The resultant crude oil was

purified using flash chromatography with 40-60 petroleum ether: diethyl ether (1:1) eluent to afford the *title compound* **25** (1.75 g, 4.7 mmol, 83%) a colourless oil consisting of two isomers in a 3:2 ratio.

R_f = 0.34 [40-60 petroleum ether:diethyl ether = 1:1]; **$[\alpha]_D^{26.9}$** = -70.1(*c* 1, $CHCl_3$); **IR** (neat/cm^{-1}) 3321, 2979, 2937, 1740, 1693, 1453, 1366, 1255, 1175, 1088, 1031; **^{1}H-NMR** (500 MHz, DMSO-D_6, temperature = 373 K) δ = 5.08 (t, *J* = 7.5 Hz, 1H, Thl^4-2H$^{\#}$), 4.80 (dd, *J* = 11.2, 3.5 Hz, 0.66H, Thl^4-2H*), 4.24 – 4.14 (m, 4H, Thl^4-C4′bH$_2$ and Thr-3H*), 4.11 (t, *J* = 6.7 Hz, 1H, Thl^4-4H$^{\#}$), 4.07 (qd, *J* = 6.3, 3.6 Hz, 1H, Thr-3H$^{\#}$), 3.92 (t, *J* = 3.9 Hz, 0.66H, Thr-2H*), 3.88 – 3.80 (m, 0.66H, Thl^4-4H*), 3.77 (dd, *J* = 7.0, 3.5 Hz, 1H, Thr-2H$^{\#}$), 3.37 (br. s, 0.66H, Thl^4-N3H*), 3.25 – 3.15 (m, 1.66H, Thl^4-5*HH*), 2.94 – 2.89 (m, 1H, Thl^4-5*H*H$^{\#}$), 2.87 – 2.81 (m, 0.66H, Thl^4-5*H*H*), 2.51 – 2.47 (m, 1H, Thl^4-N3H$^{\#}$), 1.56 (s, 2H, Thr-3′bCH$_3$*), 1.53 (s, 3H, Thr-3′bCH$_3$$^{\#}$), 1.50 (s, 2H, Thr-3′bCH$_3$*), 1.49 (s, 3H, Thr-3′bCH$_3$$^{\#}$), 1.47 – 1.45 (m, 6H, Thr-2′CH$_3$*), 1.44 (d, *J* = 1.0 Hz, 9H, Thr-2′CH$_3$$^{\#}$), 1.32 (dd, *J* = 6.3, 0.9 Hz, 2H, Thr-4CH$_3$*), 1.28 (dd, *J* = 6.4, 0.9 Hz, 3H, Thr-4CH$_3$$^{\#}$), 1.26 – 1.20 (m, 5H, Thl^4-4′cCH$_3$); **^{13}C-NMR** (126 MHz, DMSO, temperature = 373 K) δ = 170.5 (quat., Thl^4-C4′a$^{\#}$), 170.1 (quat., Thl^4-C4′a*), 151.6 (quat., Thr-C2′a*), 151.4 (quat., Thr-C2′a$^{\#}$), 93.7 (quat., Thr-C3′a*), 93.3 (quat., Thr-C3′a$^{\#}$), 79.2 (quat., Thr-C2′b*), 78.9 (quat., Thr-C2′b$^{\#}$), 73.0 (CH, Thr-C3$^{\#}$), 72.8 (CH, Thr-C3*), 70.9 (CH, Thl^4-C2*), 70.7 (CH, Thl^4-C2$^{\#}$), 66.1 (CH, Thr-C2$^{\#}$), 64.6 (CH, Thl^4-C4*), 64.5 (CH, Thr-C2*), 63.8 (CH, Thl^4-C4$^{\#}$), 60.2 (CH_2, Thl^4-C4′b*), 60.0 (CH_2, Thl^4-C4′b$^{\#}$), 36.2 (CH_2, Thl^4-C5*), 36.0 (CH_2, Thl^4-C5$^{\#}$), 28.0 (CH_3, Thr-C2′c), 27.7 (CH_3, Thr-C2′c), 27.54 (CH_3, Thr-C2′c *), 27.51 (CH_3, Thr-C2′c $^{\#}$), 27.2 (CH_3, Thr-C2′c), 26.0 (CH_3, Thr-C2′c), 21.1 (CH_3, Thr-C4$^{\#}$), 20.7 (CH_3, Thr-C4*), 13.3 (CH_3, Thl^4-C4′c$^{\#}$), 13.3 (CH_3, Thl^4-C4′c*); **HRMS** (ESI) found: 375.1963 ($[M+H]^+$ $C_{17}H_{31}N_2O_5S$ requires 375.1954).

* = signals arising solely from the minor isomer, # = signals arising solely from the major isomer

4.4. Boc-*N*-(CMe_2)-*O*-Thr-Thz^4-CO_2Et 8

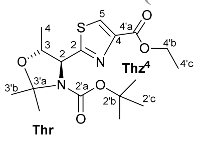

25 (0.90 g, 2.4 mmol) was taken up in toluene (15 mL) and activated manganese dioxide (4.17 g, 48.0 mmol) added before heating to 80 °C for 20 h. The reaction mixture was filtered through a plug of silica, eluting with ethyl acetate (100 mL) and the solvent removed *in vacuo*. The resultant yellow oil was purified by flash chromatography using 40-60 petroleum ether:diethyl ether (1:1) eluent to afford the *title compound* **8** (0.52 g, 1.4 mmol, 59%) as a white foam.

R_f = 0.2 [40-60 petroleum ether:diethyl ether = 1:1]; **$[\alpha]_D^{25.0}$** = -51.9(*c* 0.33, $CHCl_3$) (literature[3] $[\alpha]_D^{32}$ -51.0(c 0.3, $CHCl_3$)); **^{1}H-NMR** (600 MHz, $CDCl_3$) δ = 8.14 (s, 1H, Thz^4-C5H), 4.82 – 4.77 (m, 1H, Thr-C2H), 4.42 (q, *J* = 7.1 Hz, 2H, Thz^4-C4′bH$_2$), 4.18 – 4.15 (s, 1H, Thr-C3H), 1.78 – 1.63 (m, 6H, 2 x Thr-C3′bH$_3$), 1.53 – 1.48 (m, 3H, from 3 x Thr-C2′cH$_3$), 1.44 (dd, *J* = 12.1, 5.5 Hz, 3H, Thr-C4H$_3$), 1.39 (t, *J* = 7.1 Hz, 3H, Thz^4-C4′cH$_3$), 1.19 (s, 6H, from 3 x Thr-C2′cH$_3$); **^{13}C-NMR** (151 MHz, $CDCl_3$) δ = 173.3 (quat., Thz^4-C2), 161.2 (quat., Thz^4-C4′a), 151.3 (quat., Thr-C2′a), 146.8 (quat., Thz^4-C4), 127.2 (CH, Thz^4-C5), 95.3 (quat., Thr-C3′a), 80.7 (quat., Thr-C2′b), 77.8 (CH., Thr-C3), 65.9 (CH, Thr-C2), 61.4 (CH_2, Thz^4-C4′b), 28.1 (CH_3, Thr-C2′c), 26.5 (CH_3, Thr-C3′b), 25.9 (CH_3, Thr-C3′b), 17.9 (CH_3, Thr-C4), 14.3 (CH_3, Thz^4-C4′c). The data obtained was in agreement with that reported in the literature.[3]

5. Synthesis of *N*-Boc-Arg^1(Boc)$_2$-Thz^2-CO$_2$Me 4b and *N*-Me$_2$-Arg^1(Boc)$_2$-Thz^2-CO$_2$Me 4a

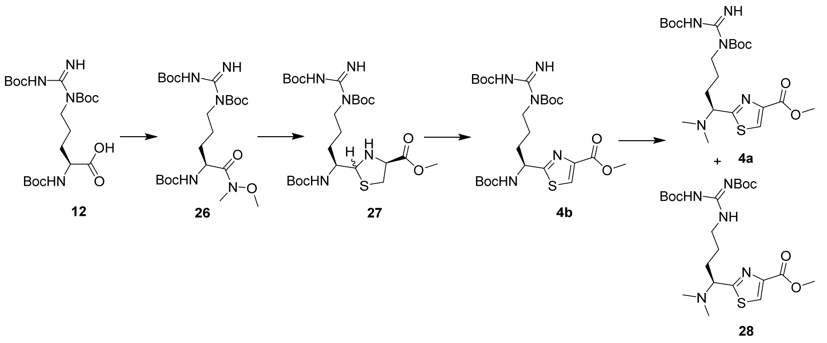

5.1. *N*-Boc-Arg^1(Boc)$_2$-N(Me)OMe 26

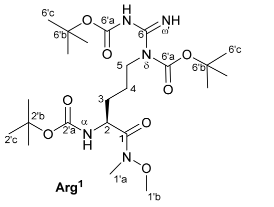

Diisopropyl ethylamine (0.76 mL, 4.4 mmol) was added to a solution of Boc-Arg(Boc)$_2$-OH (1.00 g, 2.2 mmol) and *N,O*-dimethylhydroxylamine hydrochloride (0.21 g, 2.2 mmol) in dichloromethane (20 mL) and the reaction mixture stirred for 2 minutes before HOBt (0.35 g, 2.6 mmol) was added. After a further 2 minutes at room temperature EDCI (0.50 g, 2.6 mmol) was added and the reaction mixture stirred at room temperature for 17 h. Saturated ammonium chloride (20 mL) was added and the reaction mixture was extracted with dichloromethane (3 x 20 mL) and the combined organic extracts were washed with aqueous hydrochloric acid (20 mL, 3M), saturated sodium bicarbonate (20 mL), then sodium chloride (20 mL) before drying over magnesium sulfate and removing the solvent *in vacuo* to give the *title compound* **26** (1.08 g, 2.1 mmol, 96%) as a colourless foam.

R$_f$ = 0.29 [40-60 petroleum ether:ethyl acetate = 1:1]; **[α]$_D$$^{26.1}$**+10.1(c 1, CHCl$_3$); **IR** (neat/cm^{-1}) 3389, 2978, 1709, 1607, 1506, 1271, 1249, 1143; **^{1}H-NMR** (600 MHz, CDCl$_3$) δ = 9.30 (s, 1H, Arg^{1}NωH or Arg^1-Nω′H), 9.11 (s, 1H, Arg^{1}NωH or Arg^1-Nω′H), 5.23 (d, J = 9.2 Hz, 1H, Arg^1-NαH), 4.59 (s, 1H, Arg^1-C2H), 3.82 (t, J = 7.1 Hz, 2H, Arg^1-C5H$_2$), 3.70 (s, 3H, Arg^1-C1′bH$_3$), 3.13 (s, 3H, Arg^1-C1′aH$_3$), 1.70 – 1.52 (m, 2H, Arg^1-C4*H*H and Arg^1-C3*H*H), 1.53 – 1.42 (m, 11H, Arg^1-C4*H*H and Arg^1-C3*H*H and 3 x CH$_3$ from Arg^1-C2′cH$_3$ and Arg^1-C6′cH$_3$), 1.41 (s, 9H, 3 x CH$_3$ from Arg^1-C2′cH$_3$ and Arg^1-C6′cH$_3$), 1.35 (s, 9H, 3 x CH$_3$ from Arg^1-C2′cH$_3$ and Arg^1-C6′cH$_3$); **^{13}C-NMR** (151 MHz, CDCl$_3$) δ = 172.8 (quat., Arg^1-C1), 163.7 (quat., Arg^1-C6′a), 160.5 (quat., Arg^1-C6), 155.4 (quat., Arg^1-C2′a), 154.9 (quat., Arg^1-C6′a), 83.5 (quat., Arg^1-C2′b or Arg^1-C6′b), 79.3 (quat., Arg^1-C2′b or Arg^1-C6′b), 78.5 (quat., Arg^1-C2′b or Arg^1-C6′b), 61.5 (CH$_3$, Arg^1-C1′b), 50.4 (CH, Arg^1-C2), 44.2 (CH$_2$, Arg^1-C5), 32.0 (CH$_3$, Arg^1-C1′a), 29.9 (CH$_2$, Arg^1-C3), 28.3 (CH$_3$, Arg^1-C2′c or Arg^1-C6′c), 28.2 (CH$_3$, Arg^1-C2′c or Arg^1-C6′c), 27.9 (CH$_3$, Arg^1-C2′c or Arg^1-C6′c), 24.8 (CH$_2$, Arg^1-C4); **HRMS** (ESI) found: 540.3029 ([M+Na]$^+$ C$_{26}$H$_{43}$N$_5$O$_8$Na requires 540.3009).

5.2. *N*-Boc-Arg^1(Boc)$_2$-Thl-CO$_2$Me 27

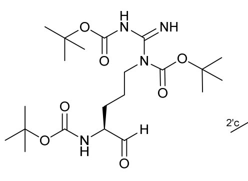

42

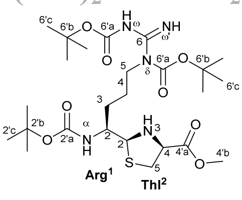

27

A solution of **26** (1.13 g, 2.2 mmol) in dichloromethane (40 mL) was cooled to -78 °C and diisobutylaluminium hydride (4.4 mL, 1 M in dichloromethane, 4.4 mmol) was added and the reaction mixture stirred at this temperature for 1 h before a saturated solution of sodium potassium tartrate (40 mL) was added and the reaction mixture stirred vigorously at room temperature for 1 h. The reaction mixture was extracted with dichloromethane (3 x 50 mL) and the combined organic extracts washed with a saturated solution of sodium chloride (50 mL) and dried over magnesium sulfate before the solvent was removed *in vacuo* to afford **42** as a white foam. This was directly taken up methanol (40 mL) and a solution of cysteine methyl ester hydrochloride (0.37 g, 2.2 mmol) and potassium bicarbonate (0.20 g, 2.0 mmol) in water (20 mL) was added. The resulting mixture was stirred vigorously for 41.5 h before the solvent was removed *in vacuo*. Water (50 mL) was added, the reaction mixture was extracted with ethyl acetate (3 x 200 mL) and the combined organic extracts were washed with saturated sodium chloride (200 mL) before the solvent was removed *in vacuo*. The resulting crude oil was purified using flash chromatography using 40-60 petroleum ether: ethyl acetate (1:1) eluent to afford the *title compound* **27** (0.98 g, 1.7 mmol, 78%) as a colourless foam.

R_f = 0.33 [40-60 petroleum ether:ethyl acetate = 1:1]; **$[\alpha]_D^{23.7}$** = -25.5(*c* 1, $CHCl_3$); **IR** (neat/cm^{-1}) 3381, 2977, 1709, 1607, 1505, 1366, 1271, 1248, 1144; **^{1}H-NMR** (600 MHz, $CDCl_3$) δ = 9.22 (s, 1H, Arg^1-NωH or Arg^1-Nω′H), 9.03 (s, 1H, Arg^1-NωH or Arg^1-Nω′H), 5.18 (d, *J* = 9.9 Hz, 0.33H, Arg^1-NαH*), 5.09 (d, *J* = 9.9 Hz, 0.66H, Arg^1-NαH$^\#$), 4.55 (s, 0.66H, Thl^2-C2H$^\#$), 4.54 – 4.43 (m, 0.33H, Thl^2-C2H*), 3.97 (tt, *J* = 9.6, 4.0 Hz, 1H, Arg^1-C2H), 3.90 – 3.62 (m, 3.66H, Arg^1-C5H$_2$, Thl^2-C4H and Arg^1-C2H$^\#$), 3.60 (s, 2H, Thl^2-C4′bH$_3$$^\#$), 3.57 (s, 1H, Thl^2-C4′bH$_3$*), 3.51 – 3.38 (m, 0.33H, Arg^1-C2H*), 3.10 - 3.01 (m, 1H, Thl^2-C5*H*H), 2.81 (dd, *J* = 10.6, 6.1 Hz, 0.33H, Thl^2-C5*H*H*), 2.72 – 2.60 (m, 0.33H, Thl^2-N3H*), 2.51 (t, *J* = 10.0 Hz, 0.66H, Thl^2-C5*H*H$^\#$), 2.44 – 2.32 (m, 0.66H, Thl^2-N3H$^\#$), 1.58 – 1.49 (m, 2H, Arg^1-C4H$_2$), 1.40 – 1.21 (m, 29H, 3 x Arg^1-C2′cH$_3$, 6 x Arg^1-C6′cH$_3$ and Arg^1-C3H$_2$); **^{13}C-NMR** (151 MHz, $CDCl_3$) δ = 172.0 (quat.,Thl^2-C4′a*), 170.9 (quat.,Thl^2-C4′a$^\#$), 163.5 (quat., Arg^1-C6′a$^\#$), 163.4 (quat., Arg^1-C6′a*), 160.5 (quat., Arg^1-C6′a*), 160.4 (quat., Arg^1-C6′a$^\#$), 156.2 (quat., Arg^1-C2′a*), 155.9 (quat., Arg^1-C2′a$^\#$), 154.79 (quat., Arg^1-C6*), 154.76 (quat., Arg^1-C6$^\#$), 83.5 (quat., Arg^1-C6′b$^\#$), 83.5 (quat., Arg^1-C6′b*), 79.3 (quat., Arg^1-C2′b$^\#$), 78.6 (quat., Arg^1-C2′b*), 78.4 (quat., Arg^1-C6′b*), 78.3 (quat., Arg^1-C6′b$^\#$), 74.2 (CH, Thl^2-C2$^\#$), 72.2 (CH, Thl^2-C2*), 65.1 (CH, Thl^2-C4$^\#$), 64.0 (CH, Arg^1-C2*), 55.9(CH, Thl^2-C4*), 52.2 (CH_2, Thz^2-C4′b$^\#$), 52.1 (CH_2, Thz^2-C4′b*), 51.7 (CH, Arg^1-C2$^\#$), 44.3 (CH_2, Arg^1-C5*), 44.1 (CH_2, Arg^1-C5$^\#$), 37.1 (CH_2, Thl^2-C5$^\#$), 37.0 (CH_2, Thl^2-C5*), 31.7 (CH_2, Arg^1-C3$^\#$), 28.24 (CH_3, 3 x Arg^1-C2′c*), 28.19 (CH_3, 3 x Arg^1-C2′c$^\#$), 28.1 (CH_3, 3 x Arg^1-C6′c), 28.0 (CH_2, Arg^1-C3*), 27.8 (CH_3, 3 x Arg^1-C6′c); **HRMS** (ESI) found: 576.3096 ($[M+H]^+$ $C_{25}H_{46}N_5O_8S$ requires 576.3067).

* = signals arising solely from the minor isomer, # = signals arising solely from the major isomer

5.3. *N*-Boc-Arg^1(Boc)$_2$-Thz^2-CO_2Me 4b

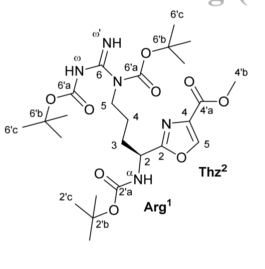

27 (0.98 g, 1.7 mmol) was taken up in toluene (40 mL) and manganese dioxide (2.96 g, 34.0 mmol) was added. The reaction mixture was heated to 80 °C for 15 h before cooling to room temperature and filtering through a pad of silica, eluting with ethyl acetate (200 mL). The solvent was removed *in vacuo* The resulting crude oil was purified using flash chromatography using 40-60 petroleum ether: ethyl acetate (2:1) eluent to afford the *title compound* **4b** (0.46 g, 0.8 mmol, 48%) as a colourless foam.

R_f = 0.49 [40-60 petroleum ether:ethyl acetate = 1:1]; **$[\alpha]_D^{27.5}$** = +2.0(*c* 1, $CHCl_3$); **IR** (neat/cm^{-1}) 3380, 2980, 1710, 1608, 1503, 1367, 1245, 1144, 726; **H-NMR** (600 MHz, $CDCl_3$) δ = 9.36 (s, 1H,

Arg^1-NωH or Arg^1-Nω′H), 9.18 (s, 1H, Arg^1-NωH or Arg^1-Nω′H), 8.09 (s, 1H, Thz^2-C5H), 6.26 (s, 1H, Arg^1-NαH), 5.05 (s, 1H, Arg^1-C2H), 4.03 (q, *J* = 7.7, 7.1 Hz, 1H, Arg^1-C5*H*H), 3.92 (s, 3H, Thz^2-C4′bH$_3$), 3.77 – 3.70 (m, 1H, Arg^1-C5*H*H), 2.12 (s, 1H, Arg^1-C3*H*H), 2.01 (s, 1H, Arg^1-C3*H*H), 1.72 (dq, *J* = 16.8, 7.2 Hz, 2H, Arg^1-C4H$_2$), 1.53 – 1.42 (m, 27H, 3 x Arg^1-C2′cH$_3$ and 6 x Arg^1-C6′cH$_3$); **^{13}C-NMR** (151 MHz, CDCl$_3$) δ = 175.1 (quat., Thz^2-C2), 163.5 (quat., Arg^1-C6′a), 161.8 (quat.,Thz^2-C4′a), 160.7 (quat., Arg^1-C6), 155.5 (quat., Arg^1-C2′a), 154.8 (quat., Arg^1-C6′a), 146.8 (quat., Thz^2-C4), 127.4 (CH, Thz^2-C5), 83.9 (quat., Arg^1-C6′b), 79.9 (quat., Arg^1-C2′b), 78.9 (quat., Arg^1-C6′b), 53.6 (CH, Arg^1-C2), 52.3 (CH$_3$, Thz^2-C4′b), 44.1 (CH$_2$, Arg^1-C5), 30.6 (CH$_2$, Arg^1-C3), 28.4 (CH$_3$, from 3 x Arg^1-C2′c and 6 x Arg^1-C6′c), 28.3 (CH$_3$, from 3 x Arg^1-C2′c and 6 x Arg^1-C6′c), 28.0 (CH$_3$, from 3 x Arg^1-C2′c and 6 x Arg^1-C6′c), 25.2 (CH$_2$, Arg^1-C4); **HRMS** (ESI) found: 594.2599 ([M+Na]$^+$ $C_{25}H_{41}N_5O_8SNa$ requires 594.2573).

5.4. *N*-Me$_2$-Arg^1(Boc)$_2$-Thz^2-CO$_2$Me 4a

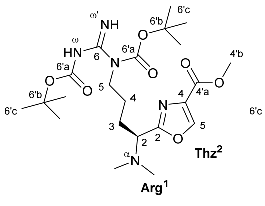

4a

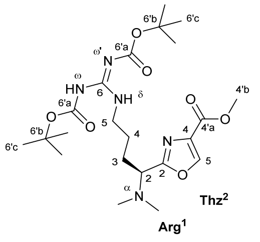

28

4b (0.80 g, 1.4 mmol) was taken up in dioxane (20 mL) and anhydrous hydrochloric acid (20 mL) was added. The reaction mixture was stirred at room temperature for 1 h before the solvent was removed *in vacuo*. Ethanol (2 x 20 mL) and methanol (20 mL) were added successively, removing the solvent in vacuo after each addition to give a colourless foam. This was taken up in methanol (45 mL) and formaldehyde (0.61 mL, 37% in water, 7.5 mmol) was added and the reaction mixture was stirred at room temperature for 1h before sodium cyanoborohydride (0.29 g, 4.7 mmol) was added and the reaction mixture stirred for a further 15.5 h before the solvent was removed *in vacuo*. Saturated sodium bicarbonate (45 mL) was added and the reaction mixture was extracted with ethyl acetate (3 x 45 mL). The combined organic extracts were washed with saturated sodium chloride (45 mL) and dried over magnesium sulfate before the solvent was removed in vacuo. The crude oil was taken up in dichloromethane (20 mL) and diisopropylethylamine (0.8 mL, 4.5 mmol) and di-*tert*-butyl dicarbonate (1.0 mL, 4.5 mmol) was added. The reaction mixture was stirred at room temperature for 48 h before a further portion of diisopropylethylamine (0.8 mL, 4.5 mmol) and di-*tert*-butyl dicarbonate (1.0 mL, 4.5 mmol) were added. After a further 48 hours stirring at room temperature water (20 mL) was added and the reaction mixture was extracted with dichloromethane (3 x 20 mL). The combined organic extracts were washed with saturated sodium chloride before drying over magnesium sulfate and removing the solvent in vacuo. The resulting crude oil was purified using flash chromatography using ethyl acetate eluent to afford the *title compound* **4a** (0.26 g, 0.5 mmol, 35%) as a colourless oil and its regioisomer **28** (0.095 g, 0.2 mmol, 13%) as a colourless foam.

Characterisation for 4a: R_f = 0.34 [ethyl acetate]; **$[\alpha]_D^{27.4}$** = +4.6(*c* 1, CHCl$_3$); **IR** (neat/cm^{-1}) 3381, 2976, 1711, 1609, 1271, 1246, 1143, 1094; **H-NMR** (600 MHz, CDCl$_3$) δ = 9.32 (s, 1H, Arg^1-NωH or Arg^1-Nω′H), 9.15 (s, 1H, Arg^1-NωH or Arg^1-Nω′H), 8.15 (s, 1H, Thz^2-C5H), 3.93 (s, 3H, Thz^2-C4′bH$_3$), 3.91 – 3.86 (m, 3H, Arg^1-C2H and Arg^1-C5H$_2$), 2.31 (s, 6H, Arg^1-NαCH$_3$), 1.93 (td, *J* = 12.0, 11.2, 5.6 Hz, 1H, Arg^1-C3*H*H), 1.82 (dddd, *J* = 13.4, 11.0, 8.0, 5.2 Hz, 1H, Arg^1-C3*H*H), 1.66 (ddt, *J* = 17.7, 11.8, 6.5 Hz, 1H, Arg^1-C4*H*H), 1.59 – 1.50 (m, 1H, Arg^1-C4*H*H), 1.47 (s, 9H, 3 x Arg^1-C6′cH$_3$), 1.45 (s, 9H, 3 x Arg^1-C6′cH$_3$). **^{13}C-NMR** (151 MHz, CDCl$_3$) δ = 173.9 (quat., Thz^2-C2), 163.8 (quat., Arg^1-C6′a), 162.0 (quat.,Thz^2-C4′a), 160.5 (quat., Arg^1-C6), 154.9 (quat., Arg^1-C6′a), 146.1 (quat., Thz^2-C4), 128.0 (CH, Thz^2-C5), 83.6 (quat., Arg^1-C6′b), 78.5 (quat., Arg^1-C6′b), 66.7 (CH, Arg^1-C2), 52.3 (CH$_3$, Thz^2-C4′b), 44.4 (CH$_2$, Arg^1-C5), 42.1 (CH$_3$, 2 x Arg^1-NαC), 30.3 (CH$_2$, Arg^1-C3), 28.3 (CH$_3$, 3 x Arg^1-C6′c), 28.0 (CH$_3$, 3 x Arg^1-C6′c), 25.6 (CH$_2$, Arg^1-C4); **HRMS** (ESI) found: 500.2568 ([M+H]$^+$ $C_{22}H_{37}N_5O_8S$ requires 500.2543).

Characterisation for 28: R$_f$ = 0.20 [ethyl acetate]; **[α]$_D$$^{23.4}$** = -3.4(*c* 1, $CHCl_3$); **IR** (neat/cm^{-1}) 3329, 2978, 2933, 1720, 1637, 1611, 1326, 1156, 1131; **H-NMR** (600 MHz, $CDCl_3$) *δ* = 11.47 (s, 1H, Arg^1-NδH), 8.32 – 8.26 (m, 1H, Arg^1-NωH), 8.17 (s, 1H, Thz^2-C5H), 3.95 (s, 3H, Thz^2-C4′bH$_3$), 3.89 (dd, *J* = 8.2, 6.1 Hz, 1H, Arg^1-C2H), 3.46 – 3.38 (m, 2H, Arg^1-C5H$_2$), 2.30 (s, 6H, 2 x Arg^1-NαCH$_3$), 1.99 (ddt, *J* = 13.5, 11.1, 5.6 Hz, 1H, Arg^1-C3*H*H), 1.85 (dddd, *J* = 13.5, 10.7, 8.2, 5.1 Hz, 1H, Arg^1-C3*H*H), 1.70 – 1.62 (m, 1H, Arg^1-C4*H*H), 1.57 – 1.52 (m, 1H, Arg^1-C4*H*H), 1.49 (s, 9H, 3 x Arg^1-C6′cH$_3$), 1.48 (s, 9H, 3 x Arg^1-C6′cH$_3$).**^{13}C-NMR** (151 MHz, $CDCl_3$) *δ* = 173.3 (quat., Thz^2-C2), 163.5 (quat., Arg^1-C6′a), 162.0 (quat.,Thz^2-C4′a), 156.1 (quat., Arg^1-C6), 153.2 (quat., Arg^1-C6′a), 146.1 (quat., Thz^2-C4), 128.1 (CH, Thz^2-C5), 83.0 (quat., Arg^1-C6′b), 79.2 (quat., Arg^1-C6′b), 66.8 (CH, Arg^1-C2), 52.4 (CH_3, Thz^2-C4′b), 42.1 (CH_3, 2 x Arg^1-NαC), 40.6 (CH_2, Arg^1-C5), 30.4 (CH_2, Arg^1-C4) 28.2(CH_3, 3 x Arg^1-C6′c), 28.0 (CH_3, 3 x Arg^1-C6′c), 25.0 (CH_2, Arg^1-C3); **HRMS** (ESI) found: 522.2341 ([M+Na]$^+$ $C_{22}H_{37}N_5O_6SNa$ requires 522.2357).

6. Synthesis of Boc-N-(CMe_2)-O-Thr-Thz^4-5-MeOxz^5-5-MeOxz^6-Ile^7-OMe 5

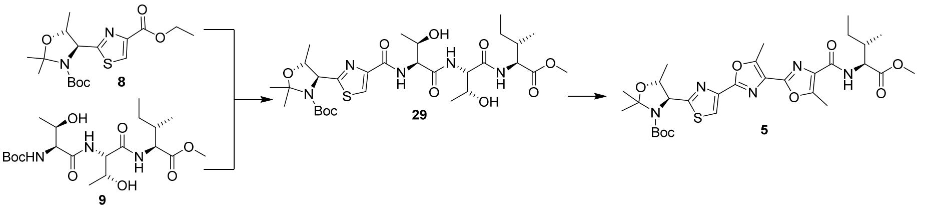

6.1. Boc-N-(CMe_2)-O-Thr^A-Thz^4-Thr^B-Thr^C-Ile^7-OMe 29

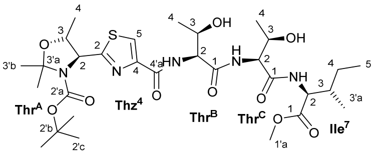

8 (1.00 g, 2.7 mmol) was taken up in methanol (60 mL) and water (40 mL), lithium hydroxide monohydrate (0.23 g, 5.4 mmol) was added and the reaction mixture stirred at room temperature for 3 hours before the reaction mixture was acidified to pH 2 using aqueous hydrochloric acid (3M) and the methanol was removed *in vacuo*. The residual water was extracted with ethyl acetate (3 x 50 mL) and the combined organic were washed with saturated sodium chloride (40 mL) and dried over magnesium sulfate before the solvent was removed *in vacuo* to give the free acid as a white solid which was used directly in the next step.

Anhydrous hydrochloric acid (15 mL, 4 M in dioxane) was added to a solution of **9** (1.21 g, 2.7 mmol) in dioxane (15 mL) and the reaction mixture stirred at room temperature for 30 minutes before the solvent was removed *in vacuo*. The oil was taken up twice in ethanol (30 mL), then in methanol (30 mL), removing the solvent *in vacuo* after each addition to give the hydrochloric acid salt as a white foam which was used directly in the next step.

The crude acid and amine were combined and taken up in dichloromethane (32 mL) and diisopropylethylamine (0.94 mL, 5.4 mmol), HOBt (0.44 g, 3.2 mmol) and EDCI (0.62 g, 3.2 mmol) were added in that order. The reaction mixture was stirred at room temperature for 17.5 hours before saturated ammonium chloride (30 mL) was added and the reaction mixture extracted with dichloromethane (3 x 30 mL). The combined organic extracts were washed with aqueous hydrochloric acid (30 mL, 3M), saturated sodium bicarbonate (30 mL) and saturated sodium chloride (30 mL) before drying over magnesium sulfate and the solvent was removed *in vacuo*. The resulting oil was purified *via* flash chromatography using ethyl acetate eluent to afford the *title compound* **29** (1.28 g, 1.9 mmol, 71%) as a white foam.

R$_f$ = 0.28 [ethyl acetate]; **[α]$_D$$^{25.0}$** -47.9(*c* 1.0, $CHCl_3$); **IR** (neat/cm^{-1}) 3316, 2975, 1743, 1699, 1645, 1537, 1365, 1260, 1135, 856, 732; **^{1}H-NMR** (600 MHz, $CDCl_3$) *δ* = 8.30 – 8.06 (m, 2H, Thz^4-C5H and Thr^B-N2H), 7.67 (d, *J* = 8.3 Hz, 1H, Thr^C-NH), 7.41 (d, *J* = 8.5 Hz, 1H, Ile^7-NH), 4.88 (s, 1H, Thr^B-C2H), 4.70 (d, *J* = 56.2 Hz, 1H, Thr^A-C2H), 4.56 (dd, *J* = 8.5, 2.9 Hz, 1H, Thr^C-C2H), 4.53 (dd, *J* = 8.5, 5.3 Hz, 1H, Ile^7-C2H), 4.45 – 4.32 (m, 2H, Thr^{B+C}-C3H), 4.15 (p, *J* = 6.2 Hz, 1H, Thr^A-C3H), 3.83 (s, 2H, Thr^{A+B}-C3OH), 3.71 (s, 3H, Ile^7-C1′aH_3), 1.92 – 1.83 (m, 1H, Ile^7-C3H), 1.66 (t, *J* = 12.2 Hz, 6H, 2 x Thr-C3′bH_3), 1.52 – 1.34 (m, 7H, 3H from 3 x Thr-C2′cH_3, Ile^7-C4*H*H and Thr^A-C4H_3), 1.26 – 1.07 (m, 12H, 6H from 3 x Thr-C2′cH_3, Thr^B-C4H_3, Thr^C-C4H_3 and Ile^7-C4H*H*), 0.88 (d, *J* = 6.9 Hz, 3H, Ile^7-C3′aH_3), 0.85 (t, *J* = 7.4 Hz, 3H, Ile^7-C5H_3). **^{13}C-NMR** (151 MHz, $CDCl_3$) *δ* = 172.9 (quat., Thz^4-C4′a), 172.3(quat., Ile^7-C1), 170.5 (quat., Thr^C-C1), 170.4 (quat., Thr^B-C1), 161.5 (quat., Thz^4-C4), 152.4 (quat., Thr^A-C2′a), 152.3 (quat., Thr^A-C2′a), 151.2 (quat., Thr^A-C2′a), 148.6 (quat., Thz^4-C2), 148.5 (quat., Thz^4-C2), 148.5 (quat., Thz^4-C2), 148.3 (quat., Thz^4-C2), 124.5 (CH, Thz^4-C5), 95.3 (quat., Thr^A-C3′a), 94.9 (quat., Thr^A-C3′a), 81.3 (quat., Thr^A-C2′b), 80.6 (quat., Thr^A-C2′b), 77.9 (CH, Thr^A-C3), 67.0 (CH, Thr^{A+B}-C3), 65.9 (CH, Thr^A-C2), 65.6 (CH, Thr^A-C2), 57.9 (CH, Thr^C-C2), 57.4 (CH, Thr^C-C2), 56.9 (CH, Thr^B-C2), 52.2 (CH, Ile^7-C2), 37.5 (CH, Ile^7-C3), 28.3 (CH_3, Thr^A -C4), 28.1 (CH_3, Thr^B-C4), 26.5 (CH_3, Thr^A-C3′b), 26.3 (CH_3, Thr^A-C3′b), 25.8 (CH_3, Thr^A-C3′b), 25.2 (CH_2, Ile^7-C4), 18.8 (CH_3, Thr^C-C4), 18.2 (CH_3, from 3 x Thr^A-C2′c), 17.9 (CH_3, from 3 x Thr^A-C2′c), 15.5 (CH_3, Ile^7-C3′a), 11.5 (CH_3, Ile^7-C5); **HRMS** (ESI) found: 694.3093 ($[M+Na]^+$ $C_{30}H_{49}O_{10}N_5SNa$ requires 694.3092).

6.2. Boc-*N*-(CMe_2)-*O*-Thr-Thz^4-5-MeOxz^5-5-MeOxz^6-Ile^7-OMe 5

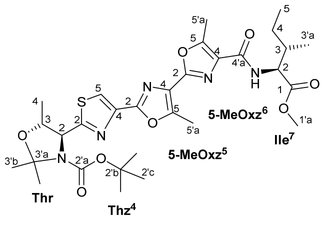

Deoxo-Fluor®[4] (0.44 mL, 50% in toluene, 0.99 mmol) was added dropwise to a solution of **29** (0.30 g, 0.45 mmol) in dichloromethane (10 mL) at -20 °C and the reaction mixture stirred at this temperature for 2.5 hours before 1,8-diazabicyclo[5.4.0]undec-7-ene (0.26 mL, 1.35 mmol) then bromotrichloromethane (0.10 mL, 1.35 mmol) was added and the reaction mixture warmed to 0 °C and stirred at this temperature for for 110 hours with further portions of 1,8-diazabicyclo[5.4.0]undec-7-ene (0.26 mL, 1.35 mmol) then bromotrichloromethane (0.10 mL, 1.35 mmol) added at time = 14 hours, 22 hours, 38 hours, 48 hours, 63 hours, 74 hours, 87 hours. The reaction mixture was quenched by addition of saturated sodium bicarbonate (15 mL) and warming to room temperature before extracting with ethyl acetate (3 x 20 mL). The combined organic extracts were washed with a saturated solution of sodium chloride (10 mL) and dried over magnesium sulfate and the reaction mixture filtered through a plug of silica before the solvent was removed *in vacuo*. This resulting oil was purified by flash chromatography using 40-60-petroleum ether:diethyl ether (1:1) as eluent to afford the *title compound* **5** (0.18 g, 0.28 mmol, 64%) as a colourless foam.

R$_f$ = 0.30 [40-60 petroleum ether:ethyl acetate = 1:1]; **[α]$_D$$^{25.0}$** -5.6(*c* 1.0, $CHCl_3$); **IR** (neat/cm^{-1}) 3406, 2975, 1741, 1705, 1673, 1630, 1509, 1365, 1135, 755; **^{1}H-NMR** (600 MHz, $CDCl_3$) *δ* = 8.06 (s, 1H, Thz^4-C5H), 7.42 (d, *J* = 9.0 Hz, 1H, Ile^7-NH), 4.91 – 4.73 (m, 1H, Thr-C2H), 4.68 (dd, *J* = 9.0, 5.5 Hz, 1H, Ile^7-C2H), 4.29 – 4.11 (m, 1H, Thr-C3H), 3.71 (s, 3H, Ile^7-C1′aH_3), 2.75 (s, 3H, 5-MeOxz^5-C5′aH3), 2.68 (s, 3H, 5-MeOxz^6-C5′aH3), 1.97 (ddd, *J* = 14.1, 10.2, 5.1 Hz, 1H, Ile^7-C3H), 1.67 (s, 6H, 2 x Thr-C3′bH_3), 1.55 – 1.46 (m, 1H, Ile^7-C4*H*H), 1.46 – 1.35 (m, 6H, Thr-C4H_3 and 3H from Thr-C2′cH_3), 1.29 – 1.19 (m, 1H, Ile^7-C4H*H*), 1.18 – 1.11 (m, 6H, from Thr-C2′cH_3), 0.94 (d, *J* = 6.9 Hz, 3H, Ile^7-C3′aH_3), 0.92 (t, *J* = 7.4 Hz, 3H, Ile^7-C5H_3); **^{13}C-NMR** (151 MHz, $CDCl_3$) *δ* = 174.2 (quat., Thz^4-C4), 172.1 (quat., Ile^7-C1), 161.5 (quat., 5-MeOxz^6-C4′a), 155.9 (quat., 5-MeOxz^6-C2), 153.1 (quat., 5-MeOxz^6-C5), 152.8 (quat., 5-MeOxz^5-C2), 151.3 (quat., Thr-C2′a), 150.5 (quat., 5-MeOxz^5-C5), 142.4 (quat., Thz^4-C2), 129.7 (quat., 5-MeOxz^6-C4), 125.8 (quat., 5-MeOxz^5-C4), 120.2 (CH, Thz^4-C5), 95.3 (quat., Thr-C3′a), 80.7 (quat., Thr-C2′b), 77.8 (CH, Thr-C3), 65.9 (CH, Thr-C2),

56.0 (CH, Ile^{7}-C2), 52.0 (CH_3, Ile^{7}-C1′a), 37.9 (CH, Ile^{7}-C3), 28.1 (CH_3, Thr-C2′c), 26.5 and 25.8 (CH_3, 2 x Thr-C3′b), 25.2 (CH_2, Ile^{7}-C4), 17.9 (CH_3, Thr-C4), 15.5 (CH_3, Ile^{7}-C3′a), 11.9 (CH_3, 5-MeOxz^{5}-C5′a), 11.8 (CH_3, 5-MeOxz^{6}-C5′a), 11.5 (CH_3, Ile^{7}-C5); **HRMS** (ESI) found: 632.2777 ($[M+H]^+$ $C_{30}H_{42}O_8N_5S$ requires 632.2754).

7. Synthesis of *N*-Me$_2$-Arg^{1}(Boc)$_2$-Thz^{2}-5-MeOxz^{3}-Thz^{4}-5-MeOxz^{5}-5-MeOxz^{6}-Ile^{7}-OMe 2a

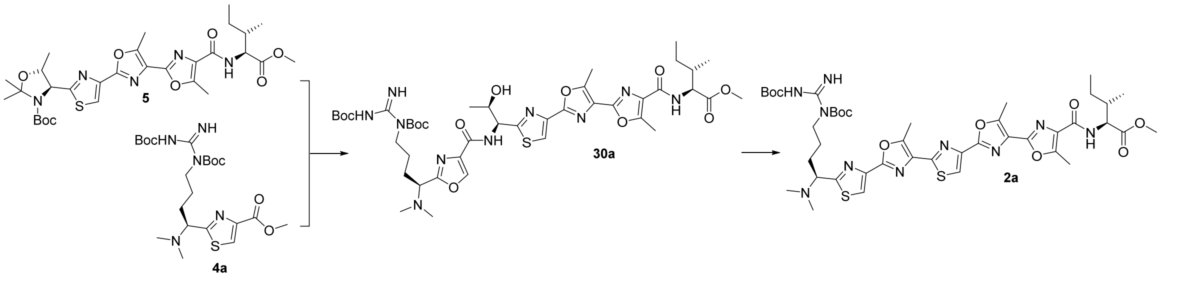

7.1. *N*-Me$_2$-Arg^{1}(Boc)$_2$-Thz^{2}-Thr-Thz^{4}-5-MeOxz^{5}-5-MeOxz^{6}-Ile^{7}-OMe 30a

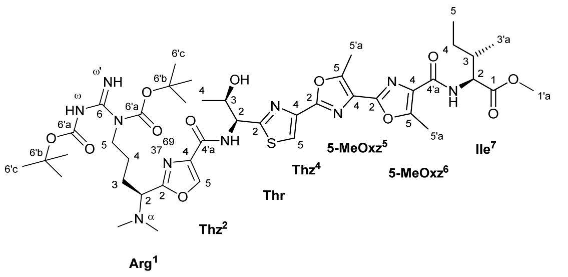

4a (0.087 g, 0.17 mmol) was taken up in tetrahydrofuran (4 mL) and the reaction mixture was cooled to 0 °C before aqueous lithium hydroxide (4 mL, 1M) and the reaction mixture was stirred at this temperature for 1.5 h before neutralising at 0 °C using aqueous hydrochloric acid (15 drops, 3M). The reaction mixture was warmed to room temperature and extracted with ethyl acetate (3 x 10 mL) and the combined organics were washed with saturated sodium chloride (5 mL) and were dried over magnesium sulfate before the solvent was removed *in vacuo* to give crude acid as a colourless foam which was used directly in the next step.

5 (0.11 g, 0.17 mmol) was taken up in dioxane (4 mL), anhydrous hydrochloric acid (4 mL, 4M) was added and the reaction mixture stirred at room temperature for 1h. The solvent was removed *in vacuo* and then ethanol (2 x 10 mL) and methanol (10 mL) were added successively, removing the solvent *in vacuo* after each addition to give the amine hydrochloride salt as colourless foam which was used directly in the next step.

The crude acid and amine and 1-[bis(dimethylamino)methylene]-1*H*-1,2,3-triazolo[4,5-b]pyridinium 3-oxide hexafluorophosphate (HATU) (0.071 g, 0.19 mmol) were taken up in dichloromethane (3 mL) and *N*,*N*-dimethylformamide (0.3 mL) and the reaction mixture was cooled to 0 °C before diisopropyl ethylamine (0.089 mL, 0.51 mmol) was added. The reaction mixture was warmed to room temperature and stirred for 22 h before water (5 mL) and ethyl acetate (5 mL) was added and the reaction mixture was neutralised with aqueous hydrochloric acid (4 drops, 3M) and extracted with ethyl acetate (3 x 10 mL). The combined organics were washed with saturated sodium chloride (5 mL) and were dried over magnesium sulfate and the solvent was removed *in vacuo*. The resulting oil was purified by preparative thin layer chromatography using ethyl acetate as eluent to afford the *title compound* **30a** (0.099 g, 0.10 mmol, 61%) as a colourless foam.

R$_f$ = 0.56 [ethyl acetate]; **[α]$_D^{26.4}$** -11.6(*c* 1.0, $CHCl_3$); **IR** (neat/cm^{-1}) 33869 2973, 1713, 1668, 1608, 1509, 1271, 1250, 1144; **^{1}H-NMR** (600 MHz, $CDCl_3$) δ = 9.31 (s, 1H, Arg^1-NωH or Arg^1-Nω'H), 9.19 (s, 1H, Arg^1-NωH or Arg^1-Nω'H), 8.28 (d, *J* = 9.0 Hz, 1H, Thr-NH), 8.13 (s, 1H, Thz^4-C5H), 8.06 (s, 1H, Thz^2-C5H), 7.47 (d, *J* = 9.0 Hz, 1H, Ile^7-NH), 5.44 (dd, *J* = 9.0, 1.9 Hz, 1H, Thr-C2H), 4.81 (qd, *J* = 6.3, 1.9 Hz, 1H, Thr-C3H), 4.72 (dd, *J* = 9.0, 5.5 Hz, 1H, Ile^7-C2H), 4.00 – 3.87 (m, 3H, Arg^1-C2H and Arg^1-C5H_2), 3.76 (s, 3H, Ile^7-C1′aH_3), 3.68 (s, 1H, Thr-C3OH), 2.79 (s, 3H, 5-MeOxz^5-C5′aH_3), 2.73 (s, 3H, 5-MeOxz^6-C5′aH_3), 2.33 (s, 6H, Arg^1-NαCH_3), 2.07 – 1.98 (m, 1H, Ile^7-C3H), 1.96 – 1.82 (m, 2H, Arg^1-C3H_2), 1.72 – 1.58 (m, 2H, Arg^1-C4H_2), 1.58 – 1.51 (m, 1H, Ile^7-C4*H*H), 1.46 (s, 9H, 3 x Arg^1-C2′cH_3), 1.44 (s, 9H, 3 x Arg^1-C2′cH_3), 1.37 (d, *J* = 6.3 Hz, 3H, Thr-C4H_3), 1.28 (ddd, *J* = 13.8, 6.9, 2.0 Hz, 1H, Ile^7-C4*H*H), 0.99 (d, *J* = 6.8 Hz, 3H, Ile^7-C3′aH_3), 0.96 (t, *J* = 7.4 Hz, 3H, Ile^7-C5H_3); **^{13}C-NMR** (151 MHz, $CDCl_3$) δ = 172.2 (quat., Ile^7-C1), 172.1 (quat., Thz^2-C4'a), 165.7 (quat., 5-MeOxz^5-C2), 163.7 (quat., 5-MeOxz^6-C4′a), 161.54 (quat., 5-MeOxz^6-C2), 161.50 (quat., Arg^1-C6′a), 160.5 (quat., Thz^2-C2), 155.8 (quat., Arg^1-C6), 154.9 (quat., Arg^1-C6′a), 153.2 (quat., 5-MeOxz^6-C5), 152.8 (quat., 5-MeOxz^5-C5), 148.5 (quat. Thz^4-C4), 142.4 (quat., Thz^2-C4), 129.7 (quat., 5-MeOxz^5-C4), 125.8, (quat., 5-MeOxz^6-C4), 124.3 (CH, Thz^4-C5), 121.0 (CH, Thz^2-C5), 83.7, 78.7 (quat., 2 x Arg^1-C6′b), 68.3 (CH, Thr-C3), 66.0 (CH, Arg^1-C2), 56.1 (CH, Ile^7-C2), 55.0 (CH, Thr-C2), 52.1 (CH_3, Ile^7-C1′a), 44.2(CH_2, Arg^1-C5), 41.8 (CH_3, 2 x Arg^1-NαC), 37.9 (CH, Ile^7-C3), 29.5 (CH_2, Arg^1-C3), 28.3 and 28.0 (CH_3, 6 x Arg^1-C6′c), 25.7 (CH_2, Arg^1-C4), 25.3 (CH_2, Ile^7-C4), 19.5 (CH_3, Thr-C4), 15.6 (CH_3, Ile^7-C3′a), 11.9 and 11.8 (CH_3, 5-MeOxz$^{5\text{ and }6}$-C5′a), 11.5 (CH_3, Ile^7-C5); **HRMS** (ESI) found: 959.4146 ($[M+H]^+$ $C_{43}H_{63}O_{11}N_{10}S_2$ requires 959.4119).

7.2. *N*-Me$_2$-Arg^1(Boc)$_2$-Thz^2-5-MeOxz^3-Thz^4-5-MeOxz^5-5-MeOxz^6-Ile^7-OMe 2a

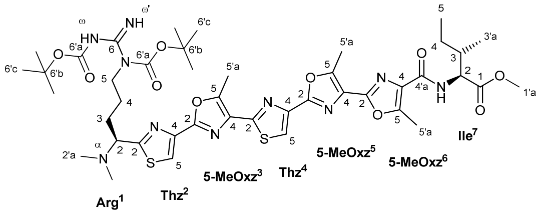

30a (0.019 g, 0.02 mmol) was taken up in dichloromethane (1 mL) and was cooled to -20 °C before Deoxo-Fluor® (0.2 mL, 0.1 M in dichloromethane, 0.02 mmol) was added. The reaction mixture was stirred at -20 °C for 2 h before 1,8-diazabicyclo[5.4.0]undec-7-ene (0.009 mL, 0.059 mmol) then bromotrichloromethane (0.006 mL, 0.059 mmol) was added and the reaction mixture warmed to 0 °C and stirred at this temperature for 20 h before saturated sodium bicarbonate (5 mL) was added and the reaction mixture was extracted with ethyl acetate (3 x 10 mL). The combined organics were washed with saturated sodium chloride (10 mL) and were dried over magnesium sulfate and the solvent was removed *in vacuo*. The resulting oil was purified by preparative thin layer chromatography using dichloromethane:methanol (19:1) as eluent to afford the *title compound* **2a** (0.013 g, 0.014 mmol, 69%) as a colourless foam.

R$_f$ = 0.47 [dichloromethane:methanol = 19:1]; **[α]$_D^{26.4}$** +13.4(*c* 1.0, $CHCl_3$); **IR** (neat/cm^{-1}) 3390, 2972, 2933, 1712, 1672, 1608, 1508, 1272, 1250, 1145; **^{1}H-NMR** (600 MHz, $CDCl_3$) δ = 9.35 (s, 1H, Arg^1-NωH or Arg^1-Nω'H), 9.18 (s, 1H, Arg^1-NωH or Arg^1-Nω'H), 8.11 (s, 1H, Thz^4-C5H), 8.03 (s, 1H, Thz^2-C5H), 7.48 (d, *J* = 9.0 Hz, 1H, Ile^7-N2H), 4.73 (dd, *J* = 9.0, 5.5 Hz, 1H, Ile^7-C2H), 4.03 – 3.97 (m, 1H, Arg^1-C2H), 3.93 (t, *J* = 7.4 Hz, 2H, Arg^1-C5H_2), 3.77 (s, 3H, Ile^7-C1′aH_3), 2.93 (s, 3H, 5-MeOxz^3-C5′aH_3), 2.82 (s, 3H, 5-MeOxz$^{5\text{ or }6}$-C5′aH_3), 2.74 (s, 3H, 5-MeOxz$^{5\text{ or }6}$-C5′aH_3), 2.36 (s, 6H, 2 x Arg-NαCH_3), 2.05 – 1.95 (m, 2H, Arg^1-C3*H*H and Ile^7-C3H), 1.95 – 1.86 (m, 1H, Arg^1-C3*H*H), 1.77 – 1.68 (m, 1H, Arg^1-C4*H*H), 1.63 – 1.53 (m, 1H, Arg^1-C4*H*H), 1.49 (s, 9H, 3 x Arg^1-C6′cH_3), 1.47 (s, 9H, , 3 x Arg^1-C6′cH_3), 1.33 – 1.23 (m, 2H, Ile^7-C4H_2), 1.00 (d, *J* = 6.8 Hz, 3H, Ile^7-C3′aH_3), 0.97 (t, *J* = 7.4 Hz, 3H, Ile^7-C5H_3); **^{13}C-NMR** (151 MHz, $CDCl_3$) δ = 174.6 (quat., Thz^2-C2), 172.2 (quat., Ile^7-C1), 163.8 (quat., Arg^1-C6′a), 162.4 (quat., Thz^4-C2), 161.6 (quat., 5-MeOxz^6-C4′a), 160.5 (quat., Arg^1-C6), 156.2 (quat., 5-MeOxz^5-C2), 155.7 (quat., 5-MeOxz^3-C2), 155.0 (quat.,

5-MeOxz^{6}-C2), 153.2 (quat., 5-MeOxz$^{5\text{ or }6}$-C5), 152.9 (quat., Arg^{1}-C6′a), 150.4 (quat., 5-MeOxz$^{5\text{ or }6}$-C5), 148.1 (quat., 5-MeOxz^{3}-C5), 143.5 (quat., Thz^{4}-C4), 142.4 (quat., Thz^{2}-C4), 130.8 (quat., 5-MeOxz^{3}-C4), 129.7 and 125.8 (quat., 5-MeOxz$^{5\text{ and }6}$-C4), 120.6 (CH, Thz^{2}-C5), 120.3 (CH, Thz^{4}-C5), 83.6 and 78.6 (quat., 2 x Arg^{1}-C6′b), 66.7 (CH, Arg^{1}-C2), 56.1 (CH, Ile^{7}-C2), 52.1 (CH_3, Ile^{7}-C1′a), 44.4 (CH_2, Arg^{1}-C5), 42.1 (CH_3, 2 x Arg^{1}-NαC), 37.9 (CH, Ile^{7}-C3), 30.4 (CH_2, Arg^{1}-C3), 28.3 and 28.0 (CH_3, 6 x Arg^{1}-C6′c), 25.6 (CH_2, Arg^{1}-C4), 25.3 (CH_2, Ile^{7}-C4), 15.6 (CH_3, Ile^{7}-C3′a), 12.2, 12.0 and 11.8 (CH_3, 5-MeOxz$^{3,\,5\text{ and }6}$-C5′a), 11.5 (CH_3, Ile^{7}-C5); **HRMS** (ESI) found: 939.3850 ($[M+H]^+$ $C_{43}H_{59}O_{10}N_{10}S_2$ requires 939.3852).

8. Synthesis of *N*-Boc-Arg^{1}(Boc)$_2$-Thz^{2}-5-MeOxz^{3}-Thz^{4}-5-MeOxz^{5}-5-MeOxz^{6}-Ile^{7}-OMe 2b

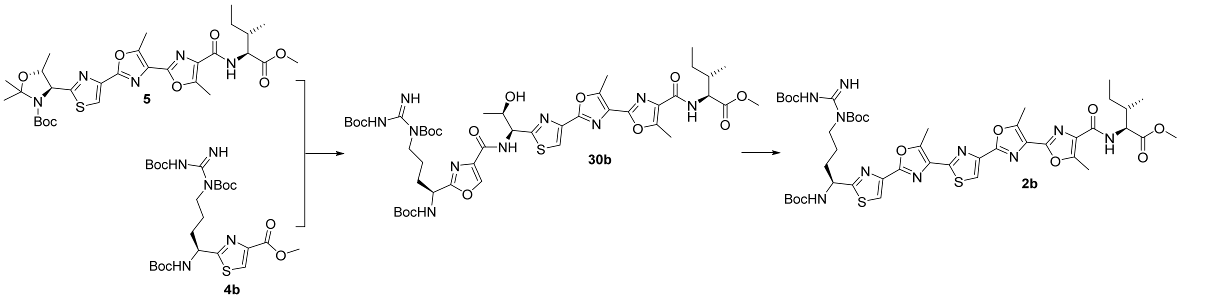

8.1. *N*-Boc-Arg^{1}(Boc)$_2$-Thz^{2}-Thr-Thz^{4}-5-MeOxz^{5}-5-MeOxz^{6}-Ile^{7}-OMe 30b

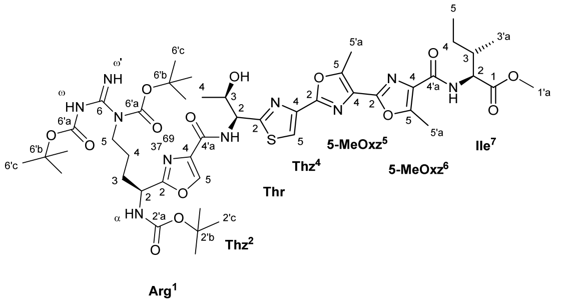

A solution of **4b** (0.114 g, 0.2 mmol) in tetrahydrofuran (4 mL) was cooled to 0 °C and aqueous lithium hydroxide (4 mL, 1M) was added. The reaction mixture stirred at 0 °C for 1.5 h before the reaction mixture was neutralised with aqueous hydrochloric acid (~40 drops, 3M) before warming to room temperature. The reaction mixture was extracted with ethyl acetate (3 x 10 mL) and the combined organic extracts were washed with saturated sodium chloride (5 mL) and dried over magnesium sulfate before the solvent was removed *in vacuo* to give the crude acid as a white solid which was used directly in the next step.

5 (0.13 g, 0.2 mmol) was taken up in dioxane (4 mL) and hydrochloric acid (4 mL, 4M in dioxane/water) was added. The reaction mixture was stirred at room temperature for 1 h before the solvent was removed *in vacuo*. The oil was taken up in ethanol (2 x 10 mL), then in methanol (10 mL), removing the solvent *in vacuo* after each addition to give the hydrochloric acid salt of the amine as a white foam which was used directly in the next step.

The crude acid and amine were combined and taken up in dichloromethane (4 mL) and *N*,*N*-dimethylformamide (0.4 mL) and HATU (0.084 g, 0.22 mmol) added before the reaction mixture cooled to 0 °C and diisopropyl ethylamine (0.11 mL, 0.6 mmol) was added. The reaction mixture was warmed to room temperature and stirred for 22 hours before neutralising with aqueous hydrochloric acid (5 drops, 3M). The reaction mixture was extracted with ethyl acetate (3 x 10 mL) and the combined organic extracts were washed with saturated sodium chloride (5 mL) before drying over

magnesium sulfate and removing the solvent *in vacuo*. The resulting oil was purified by preparative thin layer chromatography using ethyl acetate as eluent to afford the *title compound* **30b** (0.14 g, 0.13 mmol, 66%) as a colourless foam.

R$_f$ = 0.64 [ethyl acetate]; **[α]$_D^{27.5}$** -20.9(*c* 1.0, CHCl$_3$); **IR** (neat/cm^{-1}) 3386, 2974, 1713, 1677, 1611, 1512, 1274, 1251, 1148; **^{1}H-NMR** (600 MHz, CDCl$_3$) *δ* = 9.35 (s, 1H, Arg^1-NωH or Arg^1-Nω′H), 9.20 (s, 1H, Arg^1-NωH or Arg^1-Nω′H), 8.20 (d, *J* = 9.1 Hz, 1H, Thr-NH), 8.10 – 8.00 (m, 2H, Thz$^{2\text{ and }4}$-C5H), 7.46 (d, *J* = 9.0 Hz, 1H, Ile^7-NH), 6.32 (s, 1H, Arg^1-NαH), 5.42 (d, *J* = 9.0 Hz, 1H, Thr-C2H), 5.02 (s, 1H, Arg^1-C2H), 4.81 (d, *J* = 6.8 Hz, 1H, Thr-C3H), 4.71 (dd, *J* = 8.9, 5.5 Hz, 1H, Ile^7-C2H), 4.10 – 3.98 (m, 1H, Arg^1-C5*H*H), 3.85 – 3.77 (m, 1H, Arg^1-C5*H*H), 3.75 (s, 3H, Ile^7-C1′aH$_3$), 3.63 (dt, *J* = 8.9, 5.1 Hz, 1H, Thr-C3OH), 2.78 (s, 3H, 5-MeOxz^5-C5′aH3), 2.72 (s, 3H, 5-MeOxz^6-C5′aH3), 2.16 – 2.05 (m, 1H, Arg^1-C3*H*H), 2.05 – 1.95 (m, 2H, Arg^1-C3*H*H and Ile^7-C3H), 1.76 (td, *J* = 14.2, 6.6 Hz, 2H, Arg^1-C4H$_2$), 1.60 – 1.51 (m, 1H, Ile^7-C4*H*H), 1.51 – 1.39 (m, 27H, 3 x Arg^1-C2′cH$_3$ and 6 x Arg^1-C6′cH$_3$), 1.36 (d, *J* = 6.4 Hz, 3H, Thr-C4H$_3$), 1.33 – 1.21 (m, 1H, Ile^7-C4*H*H), 1.02 – 0.97 (m, 3H, Ile^7-C3′aH$_3$), 0.96 (t, *J* = 7.4 Hz, 3H, Ile^7-C5H$_3$); **^{13}C-NMR** (151 MHz, CDCl$_3$) *δ* = 175.2 and 172.3 (quat., Thz$^{2\text{ and }4}$-C4), 172.2 (quat., Ile^7-C1), 163.6 (quat., Arg^1-C6′a), 161.7 (quat., 5-MeOxz^6-C4′a), 161.6 (quat.,Thz^2-C4′a), 161.0 (quat., Arg^1-C6), 156.0 (quat., Arg^1-C6′a), 155.7 (quat., Arg^1-C2′a), 155.0 (quat., 5-MeOxz^6-C2), 153.4 (quat., 5-MeOxz^6-C5), 153.0 (quat., 5-MeOxz^5-C2), 150.7 (quat., 5-MeOxz^5-C5), 149.1 and 142.5 (quat., Thz$^{2\text{ and }4}$-C2), 129.9 (quat., 5-MeOxz^6-C4), 126.0 (quat., 5-MeOxz^5-C4), 124.1 and 121.2 (CH, Thz$^{2\text{ and }4}$-C5), 84.1 (quat., Arg^1-C6′b), 80.1 (quat., Arg^1-C2′b), 79.1 (quat., Arg^1-C6′b), 68.4 (CH, Thr-C3), 56.3 (CH, Ile^7-C2), 55.0 (CH, Thr-C2), 53.6 (CH, Arg^1-C2), 52.2 (CH$_3$, Ile^7-C1′a), 44.1 (CH$_2$, Arg^1-C5), 38.1 (CH, Ile^7-C3), 30.2 (CH$_2$, Arg^1-C3), 29.8 (CH$_2$, Ile^7-C4), 28.6, 28.4 and 28.2 (CH$_3$, 3 x Arg^1-C2′c and 6 x Arg^1-C6′c), 25.5 (CH$_2$, Arg^1-C4), 19.6 (CH$_3$, Thr-C4), 15.7 (CH$_3$, Ile^7-C3′a), 12.1 and 12.0 (CH$_3$, 5-MeOxz$^{5\text{ and }6}$-C5′a), 11.7 (CH$_3$, Ile^7-C5); **HRMS** (ESI) found: 1031.4288 ([M+H]$^+$ $C_{46}H_{67}O_{13}N_{10}S_2$ requires 1031.4325).

8.2. *N*-Boc-Arg^1(Boc)$_2$-Thz^2-5-MeOxz^3-Thz^4-5-MeOxz^5-5-MeOxz^6-Ile^7-OMe 2b

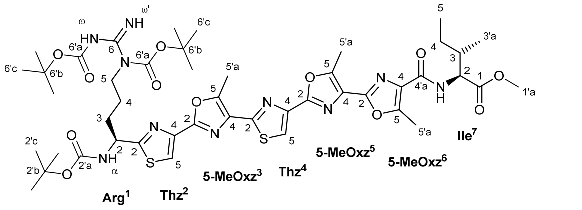

Deoxo-Fluor® (0.15 mL, 0.05M in toluene, 0.041 mmol) was added to a solution of **30b** (0.042 g, 0.041 mmol) in dichloromethane (2 mL) at -20 °C and the reaction mixture stirred at this temperature for 2 hours before 1,8-diazabicyclo[5.4.0]undec-7-ene (0.024 mL, 0.12 mmol) then bromotrichloromethane (0.019 mL, 0.12 mmol) was added and the reaction mixture warmed to 0 °C and stirred at this temperature for 15 hours. The reaction mixture was quenched by addition of a saturated solution of sodium bicarbonate (5 mL) and warming to room temperature before extracting with ethyl acetate (3 x 5 mL). The combined organic extracts were washed with a saturated solution of sodium chloride (5 mL) and dried over magnesium sulfate and the solvent was removed *in vacuo*. This resulting oil was purified by preparative thin layer chromatography using 40-60-petroleum ether:ethyl acetate (1:2) eluent to afford the *title compound* **2b** (0.038 g, 0.0037 mmol, 92%) as a colourless foam.

R$_f$ = 0.40 [ethyl acetate]; **[α]$_D^{26.6}$** +6.1(*c* 1.0, CHCl$_3$); **IR** (neat/cm^{-1}) 3381, 2964, 2930, 1712, 1609, 1509, 1273, 1253, 1148; **^{1}H-NMR** (600 MHz, CDCl$_3$) *δ* = 9.39 (s, 1H, Arg^1-NωH or Arg^1-Nω′H), 9.21 (s, 1H, Arg^1-NωH or Arg^1-Nω′H), 8.11 (s, 1H, Thz^4-C5H), 7.96 (s, 1H, Thz^2-C5H), 7.48 (d, *J* = 9.0 Hz, 1H, Ile^7-NH), 6.31 (d, *J* = 8.7 Hz, 1H, Arg^1-NαH), 5.14 (q, *J* = 7.8 Hz, 1H, Arg^1-C2H), 4.73 (dd, *J* = 9.0, 5.2 Hz, 1H, Ile^7-C2H), 4.11 – 4.06 (m, 1H, Arg^1-C5*H*H), 3.83 – 3.78 (m, 1H, Arg^1-C5*H*H), 3.77 (s, 3H, Ile^7-C1′aH$_3$), 2.92 (s, 3H, 5-MeOxz^3-C5′aH$_3$), 2.82 (s, 3H, 5-MeOxz^5-C5′aH$_3$), 2.74 (s, 3H, 5-MeOxz^6-C5′aH$_3$), 2.25 – 2.18 (m, 1H, Arg^1-C3*H*H), 2.06 (dd, *J* = 13.6, 7.8 Hz, 1H,

Arg[1]-C3HH), 2.01 (ddd, J = 9.0, 6.8, 2.0 Hz, 1H, Ile[7]-C3H), 1.78 (dq, J = 13.6, 6.8 Hz, 2H, Arg[1]-C4H_2), 1.63 – 1.52 (m, 1H, Ile[7]-C4HH), 1.51 (s, 9H, 3 x Arg[1]-C6′cH_3), 1.50 (s, 9H, 3 x Arg[1]-C6′cH_3), 1.48 (s, 9H, 3 x Arg[1]-C2′cH_3), 1.33 – 1.23 (m, 1H, Ile[7]-C4HH), 0.99 (d, J = 6.8 Hz, 3H, Ile[7]-C3′aH_3), 0.97 (t, J = 7.4 Hz, 3H, Ile[7]-C5H_3); **^{13}C-NMR** (151 MHz, $CDCl_3$) δ = 176.0 (quat., Thz[2]-C2), 172.2 (quat., Ile[7]-C1), 163.5 (quat., Arg[1]-C6′a), 162.4 (quat., Thz[4]-C2), 161.6 (quat., 5-MeOxz[6]-C4′a), 160.8 (quat., Arg[1]-C6), 156.2 (quat., 5-MeOxz[5]-C2), 155.6 (2 x quat., Arg[1]-C2′a and 5-MeOxz[3]-C2), 154.9 (quat., 5-MeOxz[3 or 6]-C2), 153.2 (quat., 5-MeOxz[6]-C5), 152.9 (quat., Arg[1]-C6′a), 150.4 (quat., 5-MeOxz[5]-C5), 148.1 (quat., 5-MeOxz[3]-C5), 143.5 (quat., Thz[4]-C4), 143.0 (quat., Thz[2]-C4), 130.8 (quat., 5-MeOxz[3]-C4), 129.7 (quat., 5-MeOxz[6]-C4), 125.8 (quat., 5-MeOxz[5]-C4), 120.3 (CH, Thz[4]-C5), 120.1 (CH, Thz[2]-C5), 83.9 (quat., Arg[1]-C6′b), 79.9 (quat., Arg[1]-C2′b), 78.9 (quat., Arg[1]-C6′b), 56.1 (CH, Ile[7]-C2), 53.7 (CH, Arg[1]-C2), 52.1 (CH_3, Ile[7]-C1′a), 44.2 (CH_2, Arg[1]-C5), 37.9 (CH, Ile[7]-C3), 30.7 (CH_2, Arg[1]-C3), 28.4 (CH_3, 3 x Arg[1]-C2′c), 28.3 (CH_3, 3 x Arg[1]-C6′c), 28.0 (CH_3, 3 x Arg[1]-C6′c), 25.3 (CH_2, Ile[7]-C4 and Arg[1]-C4), 15.6 (CH_3, Ile[7]-C3′a), 12.2, 12.0 and 11.8 (CH_3, 5-MeOxz[3, 5 and 6]-C5′a), 11.5 (CH_3, Ile[7]-C5); **HRMS** (ESI) found: 1011.4082 ($[M+H]^+$ $C_{46}H_{63}O_{12}N_{10}S_2$ requires 1011.4063).

9. Synthesis of *N*-Boc-Ile[8]-Oxz[9]-Oxz[10]-Oxz[11]-Oxz[12]-CO_2Me 6

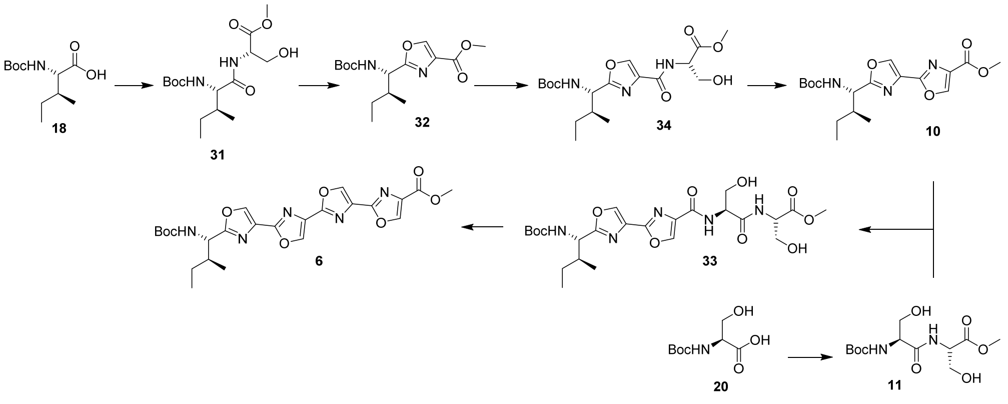

9.1. General procedure A:

To a solution of carboxylic acid (1.0 eq) and amine hydrochloric acid salt (1.0 eq) in dichloromethane was added diisopropylethylamine (2.0 eq), HOBt (1.2 eq) and EDCI (1.2 eq). The reaction mixture was stirred at room temperature for 20 h after which it was quenched with saturated aqueous ammonium chloride. The aqueous layer was extracted with dichloromethane (3 x), the combined organic layers were washed successively with aqueous hydrochloric acid (0.5 M), saturated sodium bicarbonate and saturated sodium chloride and then were dried over sodium sulfate and concentrated *in vacuo*.

9.2. General procedure B:

Deoxo-Fluor® (1.1 eq) was added to a solution of β-hydroxy amide (1.0 eq) in dichloromethane at -20 °C. After 30 min at this temperature, bromotrichloromethane (3.6 eq) then 1,8-diazabicyclo[5.4.0]undec-7-ene (3.6 eq) were added and the reaction mixture was warmed to 2-3 °C and stirred at that temperature for 8 h, after which it was quenched with saturated sodium bicarbonate. The reaction mixture was extracted with dichloromethane (3 x), the combined organic layers were washed with saturated sodium chloride, dried over Na_2SO_4 and concentrated *in vacuo*.

9.3. *N*-Boc-Ile^{8}-Ser-OMe 31

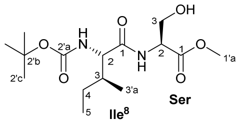

According to general procedure A, *N*-Boc-Ile-OH **18** (11.56 g, 50.0 mmol) and Ser-OMe·HCl **19** (7.77 g, 50.0 mmol) were coupled in dichloromethane (170 mL). The resultant crude oil was purified by flash chromatography using *n*-hexane:ethyl acetate (1:1 → 1:3) eluent to afford the *title compound* **31** (15.19 g, 45.7 mmol, 91%) as a colorless foam.

R$_f$ = 0.28 [*n*-hexanes:ethyl acetate = 1:1.5]; $[a]_D^{28.4}$ = +9.9 (*c* = 1.00, $CHCl_3$); **IR** (neat)/cm^{-1} 3321, 2962, 1743, 1647, 1520, 1241, 1160, 1021; **^{1}H-NMR** ($CDCl_3$, 500 MHz) δ = 7.06 (d, *J* = 7.5 Hz, 1H, Ser-NH), 5.28 (d, *J* = 8.2 Hz, 1H, Ile^{8}-NH), 4.67 (dt, *J* = 7.3, 3.5 Hz, 1H, Ser-C2H), 4.02 – 3.86 (m, 3H, Ile^{8}-C2H, Ser-C3H_2), 3.76 (s, 3H, Ser-C1′H_3), 3.26 (s, 1H, Ser-C3OH), 1.92 – 1.73 (m, 1H, Ile^{8}-C3H), 1.65 – 1.49 (m, 1H, Ile^{8}-C4*H*H), 1.42 (s, 3H, Ile^{8}-C2′cH_3), 1.24 – 1.07 (m, 1H, Ile^{8}-C4H*H*), 0.95 (d, *J* = 6.8 Hz, 3H, Ile^{8}-C3′H_3), 0.90 (t, *J* = 7.4 Hz, 3H, Ile^{8}-C5H_3); **^{13}C-NMR** ($CDCl_3$, 126 MHz) δ = 172.1 (quat., Ile-C1), 170.7 (quat., Ser-C1), 156.2 (quat., Ile^{8}-C2′a), 80.2 (quat., Ile^{8}-C2′b), 62.7 (CH_2, Ser-C3), 59.5 (CH, Ile^{8}-C2), 54.7 (CH, Ser-C2), 52.6 (CH_3, Ser-C1′a), 37.1 (CH, Ile^{8}-C3), 28.3 (CH_3, Ile^{8}-C2′c), 24.8 (CH_2, Ile^{8}-C4), 15.4 (CH_3, Ile^{8}-C3′a), 11.2 (CH_3, Ile^{8}-C5); **HRMS** (ESI) found: 355.1850 ($[M+ Na]^+$ $C_{15}H_{28}N_2O_6Na$ requires 355.1840). The data obtained was in agreement with that reported in the literature.[5]

9.4. *N*-Boc-Ile^{8}-Oxz^{9}-CO_2Me 32

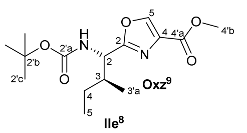

According to general procedure B, **31** (9.97 g, 30.0 mmol) was cyclized in dichloromethane (270 mL). The crude product was purified by flash chromatography using *n*-hexane:ethyl acetate (5:1) eluent to afford the *title compound* **32** (7.59 g, 24.3 mmol, 81%) as a colorless foam.

R$_f$ = 0.20 [*n*-hexanes:ethyl acetate = 3:1]; $[a]_D^{28.3}$ = -26.2 (*c* = 1.00, $CHCl_3$); **IR** (neat)/cm^{-1} 3323, 2972, 1728, 1698, 1530, 1328, 1232, 1152, 1105; **^{1}H-NMR** ($CDCl_3$, 400 MHz) δ = 8.17 (s, 1H, Oxz^{9}-C5H), 5.30 (d, *J* = 7.7 Hz, 1H, Ile^{8}-NH), 4.89 – 4.80 (m, 1H, Ile^{8}-C2H), 3.90 (s, 3H, Oxz^{9}-C4′bH_3), 2.00 – 1.87 (m, 1H, Ile^{8}-C3H), 1.51 – 1.31 (m, 1H, Ile^{8}-C4*H*H), 1.42 (s, 9H, Ile^{8}-C2′cH_3), 1.17 (dd, *J* = 13.5, 7.0 Hz, 1H, Ile^{8}- C4*H*H), 0.90 (t, *J* = 7.3 Hz, 3H, Ile^{8}-C5H_3), 0.85 (d, *J* = 6.6 Hz, 3H, Ile^{8}-C3′aH_3); **^{13}C-NMR** ($CDCl_3$, 100 MHz) δ = 165.1 (quat., Oxz^{9}-C2), 161.6 (quat., Oxz^{9}-C4′a), 155.2 (quat., Ile^{8}-C2′a), 143.7 (CH, Oxz^{9}-C5), 133.2 (quat., Oxz^{9}-C4), 80.0 (quat., Ile^{8}-C2′b), 53.3 (CH, Ile^{8}-C2), 52.2 (CH_3, Oxz^{9}-C4′b), 39.5 (CH, Ile^{8}-C3), 28.3 (CH_3, Ile^{8}-C2′c), 25.0 (CH_2, Ile^{8}-C4), 15.1 (CH_3, Ile^{8}-C3′a), 11.3 (CH_3, Ile^{8}-C5); **HRMS** (ESI) found: 313.1750 ($[M+H]^+$ $C_{15}H_{25}N_2O_5$ requires 313.1758). The data obtained was in agreement with that reported in the literature.[5]

9.5. *N*-Boc-Ile^{8}-Oxz^{9}-Ser-OMe 34

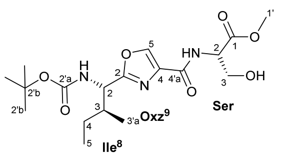

Lithium hydroxide monohydrate (0.73 g, 17.3 mmol) was added to a solution of methyl ester **32** (3.61 g, 11.6 mmol) in methanol (25 mL), tetrahydrofuran (25 mL) and water (5 mL) at 0 °C. The reaction mixture was stirred at room temperature overnight before it was acidified to pH 2-4 using aqueous hydrochloric acid (3 M) and the solvent was removed *in vacuo*. Ethyl acetate (25 mL) and water (25 mL) were added and the aqueous layer was extracted with EtOAc (3 x 25 mL). The combined organic layers were washed with saturated sodium chloride (25 mL), dried over Na_2SO_4 and the solvent removed *in vacuo* to afford the crude free acid (3.37 g, 11.3 mmol), which was directly coupled with Ser-OMe·HCl **19** (1.76 g, 11.3 mmol), in dichloromethane (40.0 mL) according to general procedure A. The crude oil was purified by flash chromatography using *n*-hexane:ethyl acetate (1:1 → 1:2) eluent to afford the *title compound* **34** (3.80 g, 9.5 mmol, 84%) as a colorless foam.

$\mathbf{R_f}$ = 0.31 [*n*-Hexane:ethyl acetate = 1:2]; $[a]_D^{27.6}$ = -12.6 (*c* = 1.00, $CHCl_3$); **IR** (neat)/cm^{-1} 3320, 2967, 1661, 1599, 1509, 1366, 1248, 1165, 871; **^{1}H-NMR** ($CDCl_3$, 500 MHz) δ = 8.10 (s, 1H, Oxz^9-C5H), 7.76 (d, *J* = 7.3 Hz, 1H, Ser-NH), 5.43 (d, *J* = 9.1 Hz, 1H, Ile^8-NH), 4.86 – 4.76 (m, 2H, Ile^8-C2H, Ser-C2H), 4.14 – 4.05 (m, 1H, Ser-C3*H*H), 4.01 (dd, *J* = 11.3, 3.3 Hz, 1H, Ser-C3*H*H), 3.80 (s, 3H, Ser-C1′H$_3$), 1.96 – 1.83 (m, 1H, Ile^8-C3H), 1.53 – 1.39 (m, 4H, Ile^8-C4*H*H, Ile^8-C2′cH$_3$), 1.23 – 1.11 (m, 1H, Ile^8-C4*H*H), 0.91 (t, *J* = 7.3 Hz, 3H, Ile^8-C5H$_3$), 0.84 (d, *J* = 7.0 Hz, 3H, Ile^8-C3′aH$_3$); **^{13}C-NMR** ($CDCl_3$, 100 MHz) δ = 170.5 (quat., Ser-C1), 163.9 (quat., Oxz^9-C2), 160.8 (quat., Oxz^9-C4′a), 155.4 (quat., Ile^8-C2′a), 141.3 (CH, Oxz^9-C5), 135.4 (quat., Oxz^9-C4), 80.4 (quat., Ile^8-C2′b), 63.1 (CH_2, Ser-C3), 54.5 (CH, Ser-C2), 53.4 (CH, Ile^8-C2), 52.7 (CH_3, Ser-C1′a), 39.2 (CH, Ile^8-C3), 28.3 (CH_3, Ile^8-C2′c), 25.1 (CH_2, Ile^8-C4), 15.2 (CH_3, Ile^8-C3′a), 11.3 (CH_3, Ile^8-C5); **HRMS** (ESI) found: 422.1891 ($[M+H]^+$ $C_{18}H_{30}N_3O_7$ requires 422.1898).

9.6. *N*-Boc-Ile^8-Oxz^9-Oxz^{10}-CO_2Me 10

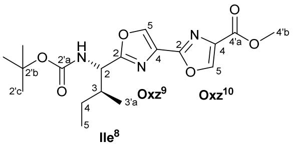

According to general procedure B, **34** (3.80 g, 9.5 mmol) was cyclized in dichloromethane (120 mL). The crude oil was purified by flash chromatography using *n*-hexane:ethyl acetate (3:1 → neat ethyl acetate) eluent to afford the *title compound* **10** (2.80 g, 7.4 mmol, 78%) as a colorless foam.

$\mathbf{R_f}$ = 0.10 [*n*-hexane:ethyl acetate = 1:3]; $[a]_D^{25.0}$ = -34.0 (*c* = 1.00, $CHCl_3$); **IR** (neat)/cm^{-1} 3141, 2959, 1721, 1690, 1509, 1247, 1147, 1001, 870, 729; **^{1}H-NMR** ($CDCl_3$, 500 MHz) δ = 8.30 (s, 1H, Oxz^9-C5H), 8.29 (s, 1H, Oxz^{10}-C5H), 5.30 (d, *J* = 9.1 Hz, 1H, Ile^8-NH), 4.90 (dd, *J* = 9.2, 5.8 Hz, 1H, Ile^8-C2H), 3.94 (s, 3H, Oxz^{10}-C4′bH$_3$), 2.02 – 1.92 (m, 1H, Ile^8-C3H), 1.53 – 1.43 (m, 1H, Ile^8-C4*H*H), 1.43 (s, 3H, Ile^8-C2′cH$_3$), 1.27 – 1.14 (m, 1H, Ile^8-C4*H*H), 0.92 (t, *J* = 7.4 Hz, 3H, Ile^8-C5H$_3$), 0.89 (d, *J* = 6.8 Hz, 3H, Ile^8-C′3H$_3$); **^{13}C-NMR** ($CDCl_3$, 126 MHz) δ = 165.5 (quat., Oxz^9-C2), 161.3 (quat., Oxz^{10}-C4′a), 155.7 (quat., Oxz^{10}-C2), 155.2 (quat., Ile^8-C2′a), 143.7 (CH, Oxz^{10}-C5), 139.3 (CH, Oxz^9-C5), 134.3 (quat., Oxz^{10}-C4), 129.6 (quat., Oxz^9-C4), 80.1 (quat., Ile^8-C2′b), 53.4 (CH, Ile^8-C2), 52.3 (CH_3, Oxz^{10}-C4′b), 39.5 (CH, Ile^8-C3), 28.3 (CH_3, Ile^8-C2′c), 25.1 (CH_2, Ile^8-C4), 15.2 (CH_3, Ile^8-C5), 11.4 (CH_3, Ile^8-C3′a); **HRMS** (ESI) found: 402.1629 ($[M+Na]^+$ $C_{18}H_{25}N_3O_6Na$ requires 402.1636).

9.7. *N*-Boc-Ser^A-Ser^B-OMe 11

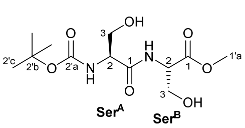

According to general procedure A, Boc-Ser-OH **20** (3.08 g, 15.0 mmol) and Ser-OMe·HCl **19** (2.33 g, 15.0 mmol) were coupled in dichloromethane (60 mL) with an amended work up. After 16 h the reaction mixture was quenched with saturated ammonium chloride (60 mL). The reaction mixture was extracted with dichloromethane (3 x 60 mL), the combined organic layers were washed with aqueous hydrochloric acid (60 mL, 0.5 M), saturated sodium bicarbonate (60 mL) and saturated sodium chloride (60 mL). The combined aqueous layers were then extracted with ethyl acetate (4 x 60 mL). The combined organic extracts were dried over sodium sulfate and concentrated *in vacuo* to yield the *title compound* **11** (4.06 g, 13.2 mmol, 88%) as a colorless solid, mp 63.7 °C (lit m.p. 62-64 °C)[6].

R_f = 0.60 [dichloromethane:methanol = 9:1]; $[a]_D^{25.3}$ = +5.3 (*c* = 1.00, $CHCl_3$); **IR** (neat)/cm^{-1} 3325, 2978, 1513, 1465, 1392, 1367, 1227, 1161, 1057, 854; **^{1}H-NMR** ($CDCl_3$, 500 MHz) δ = 7.53 (d, *J* = 6.7 Hz, 1H, Ser^B-NH), 5.77 (d, *J* = 7.2 Hz, 1H, Ser^A-NH), 4.68 – 4.61 (m, 1H, Ser^B-C2H), 4.24 (s, 1H, Ser^A-C2H), 4.02 (dd, *J* = 11.2, 3.7 Hz, 1H, Ser^A-C3H*H*), 3.96 (dd, *J* = 3.6, 1.5 Hz, 2H, Ser^B-C3H_2), 3.78 (s, 3H, Ser^B-C1′aH_3), 3.71 (dd, *J* = 10.8, 5.5 Hz, 1H, Ser^A-C3*H*H), 1.44 (s, 3H, Ser^A-C2′cH_3); **^{13}C-NMR** ($CDCl_3$, 126 MHz) δ = 171.5 (quat., Ser^A-C1), 170.9 (quat., Ser^B-C1), 156.0 (quat., Ser^A-C2′a), 80.7 (quat., Ser^A-C2′b), 63.0 (CH, Ser^A-C3), 62.4 (CH_2, Ser^B-C3), 55.6 (CH, Ser^A-C2), 55.0 (CH, Ser^B-C2), 52.9 (CH_3, Ser^B-C1′a), 28.3 (CH_3, Ser^A-C2′c); **HRMS** (ESI) found: 307.1504 ($[M+H]^+$ $C_{12}H_{23}N_2O_7$ requires 307.1505). The data obtained was in agreement with that reported in the literature.[6]

9.8. *N*-Boc-Ile^8-Oxz^9-Oxz^{10}-Ser^A-Ser^B-OMe 33

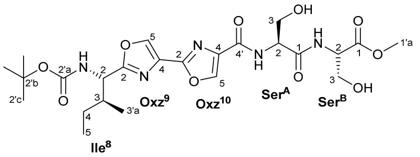

Lithium hydroxide monohydrate (0.25 g, 6.0 mmol) was added to a solution of methyl ester **34** (0.76 g, 2.0 mmol) in methanol (15 mL), tetrahydrofuran (25 mL) and water (2.5 mL) at 0 °C. The reaction mixture was stirred at room temperature for 2 h, then it was acidified to between pH 2 – 4 with aqueous hydrochloric acid (3 M) and the solvent was removed *in vacuo*. Ethyl acetate (25 mL) and water (25 mL) were added and the reaction mixture extracted with ethyl acetate (3 x 25 mL). The combined organic layers were dried over sodium sulfate and the solvent removed *in vacuo* to give crude acid which was used in the coupling with no further purification.

Hydrochloric acid (5.9 mL, 4 M in 1,4-dioxane) was added to **11** (0.65 g, 2.1 mmol) at 0 °C. The reaction mixture was stirred at room temperature for 3.5 h before the solvent was removed *in vacuo* to give amine hydrochloric acid salt which was used in the coupling with no further purification.

According to general procedure A, the crude acid and amine hydrochloric acid amine were coupled in dichloromethane (25 mL). The crude oil was purified by flash chromatography using dichloromethane:methanol (19:1) eluent to afford the *title compound* **33** (0.67 g, 1.2 mmol, 61%) as a colorless foam.

R_f = 0.11 [dichloromethane:methanol = 19:1]; $[a]_D^{25.0}$ = -4.0 (*c* = 0.30, $CHCl_3$); **IR** (neat)/cm^{-1} 3313, 2963, 1741, 1687, 1661, 1515, 1172, 1100, 1050, 972; **^{1}H-NMR** ($CDCl_3$, 500 MHz) δ = 8.21 (s, 1H, Oxz^{10}-C5H), 8.18 (s, 1H, Oxz^9-C5H), 8.07 (d, *J* = 7.9 Hz, 1H, Ser^A-NH), 7.61 (d, *J* = 7.6 Hz, 1H, Ser^B-NH), 5.72 (d, *J* = 9.3 Hz, 1H, Ile^8-NH), 4.91 (dd, *J* = 9.3, 6.4 Hz, 1H, Ile^8-C2H), 4.81 (dt, *J* = 8.0, 4.7 Hz, 1H, Ser^A-C2H), 4.72 (dt, *J* = 7.5, 3.3 Hz, 1H, Ser^B-C2H), 4.21 (dd, *J* = 11.3, 3.5 Hz, 1H, Ser^A-C3*H*H), 4.00 (d, *J* = 3.5 Hz, 2H, Ser^B-C3H_2), 3.93 (dd, *J* = 11.6, 5.2 Hz, 1H, Ser^A-C3H*H*), 3.78

(s, 3H, Ser^{B}-C1′aH$_3$), 2.03 – 1.94 (m, 1H, Ile^{8}-C3H), 1.55 – 1.46 (m, 1H, Ile^{8}-C4*H*H), 1.44 (s, 3H, Ile^{8}-C2′cH$_3$), 1.29 – 1.18 (m, 1H, Ile^{8}-C4*H*H), 0.93 (t, *J* = 7.4 Hz, 3H, Ile^{8}-C5H$_3$), 0.90 (d, *J* = 6.7 Hz, 3H, Ile^{8}-C3′aH$_3$); **^{13}C-NMR** ($CDCl_3$, 126 MHz) δ = 170.8 (quat., Ser^{B}-C1), 170.5 (quat., Ser^{A}-C1), 166.1 (quat., Oxz^{9}-C2), 161.0 (quat., Oxz^{10}-C4′a), 155.4 (quat., Ile^{8}-C2′a), 155.0 (quat., Oxz^{10}-C2), 141.6 (CH, Oxz^{10}-C5), 139.2 (CH, Oxz^{9}-C5), 136.4 (quat., Oxz^{10}-C4), 129.5 (quat., Oxz^{9}-C4), 80.1 (quat., Ile^{8}-C2′b), 62.7 (CH_2, Ser^{A}-C3), 62.6 (CH_2, Ser^{B}-C3), 55.1 (CH, Ser^{B}-C2), 54.4 (CH, Ser^{A}-C2), 53.4 (CH, Ile^{8}-C2), 52.9 (CH_3, Ser^{B}-C1′a), 39.4 (CH, Ile^{8}-C3), 28.3 (CH_3, Ile^{8}-C2′c), 25.1 (CH_2, Ile^{8}-C4), 15.2 (CH_3, Ile^{8}-C3′a), 11.3 (CH_3, Ile^{8}-C5); **HRMS** (ESI) found: 576.2279 ([M+Na]$^{+}$ $C_{24}H_{35}N_5O_{10}Na$ requires 576.2276).

9.9. *N*-Boc-Ile^{8}-Oxz^{9}-Oxz^{10}-Oxz^{11}-Oxz^{12}-CO_2Me 6

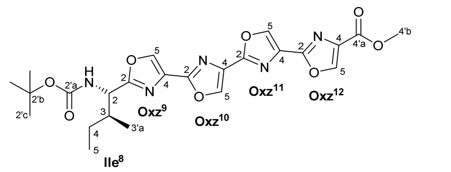

Deoxo-Fluor® (0.33 mL, 0.9 mmol) was added to a solution of **33** (0.22 g, 0.4 mmol) in dichloromethane (15 mL) at -20 °C. After 30 min at this temperature, bromotrichloromethane (0.31 mL, 3.1 mmol) then 1,8-diazabicyclo[5.4.0]undec-7-ene (0.46 mL, 3.1 mmol) were added and the reaction mixture was warmed to 0 °C and stirred at that temperature for 16 h. Further bromotrichloromethane (0.31 mL, 3.1 mmol) then 1,8-diazabicyclo[5.4.0]undec-7-ene (0.46 mL, 3.1 mmol) was added and the reaction mixture was stirred at 0 °C for a further 8 h. The reaction mixture was quenched with saturated sodium bicarbonate (15 mL) before extracting with dichloromethane (3 x 15 mL). The combined organic layers were washed with saturated sodium chloride (15 mL) and dried over sodium sulfate before the solvent was removed *in vacuo*. The crude oil was purified by flash chromatography using dichloromethane:ethyl acetate (5:1 → 3:1) eluent to afford the *title compound* **6** (0.16 g, 0.31 mmol, 77%) as a colorless foam.

R$_f$ = 0.20 [dichloromethane:ethyl acetate = 5:1]; $[a]_D^{28.6}$ = -34.0 (*c* = 0.20, $CHCl_3$); **IR** (neat)/cm^{-1} 3378, 3176, 3141, 2961, 1726, 1693, 1514, 1246, 1121, 1102, 975, 724; **^{1}H-NMR** ($CDCl_3$, 500 MHz) δ = 8.45 (s, 1H, Oxz^{11}-C5H), 8.42 (s, 1H, Oxz^{10}-C5H), 8.33 (s, 1H, Oxz^{12}-C5H), 8.32 (s, 1H, Oxz^{9}-C5H), 5.33 (d, *J* = 9.1 Hz, 1H, Ile^{8}-NH), 4.92 (dd, *J* = 9.1, 5.8 Hz, 1H, Ile^{8}-C2H), 3.96 (s, 3H, Oxz^{12}-C4′bH$_3$), 2.04 – 1.94 (m, 1H, Ile^{8}-C3H), 1.54 – 1.46 (m, 1H, Ile^{8}-C4*H*H), 1.44 (s, 3H, Ile^{8}-C2′cH$_3$), 1.29 – 1.17 (m, 1H, Ile^{8}-C4*H*H), 0.93 (t, *J* = 7.4 Hz, 3H, Ile^{8}-C5H$_3$), 0.90 (d, *J* = 6.8 Hz, 3H, Ile^{8}-C′3H$_3$); **^{13}C-NMR** ($CDCl_3$, 126 MHz) δ = 165.7 (quat., Oxz^{9}-C2), 161.3 (quat., Oxz^{12}-C4′a), 156.2 (quat., Oxz^{11}-C2), 155.8 (quat., Oxz^{10}-C2), 155.3 (quat, Oxz^{12}-C2), 155.2 (quat., Ile^{8}-C2′a), 143.9 (CH, Oxz^{12}-C5), 139.5 (CH, Oxz^{11}-C5), 139.3 (CH, Oxz^{9}-C5), 139.2 (CH, Oxz^{10}-C5), 134.4 (quat., Oxz^{12}-C4), 130.9 (quat., Oxz^{11}-C4), 130.8 (quat., Oxz^{10}-C4), 129.6 (quat., Oxz^{9}-C4), 80.1 (quat., Ile^{8}-C2′b), 53.4 (CH, Ile^{8}-C2), 52.4 (CH_3, Oxz^{12}-C4′b), 39.5 (CH, Ile^{8}-C3), 28.3 (CH_3, Ile^{8}-C2′c), 25.1 (CH_2, Ile^{8}-C4), 15.2 (CH_3, Ile^{8}-C3′a), 11.4 (CH_3, Ile^{8}-C5); **HRMS** (ESI) found: 536.1749 ([M+Na]$^{+}$ $C_{24}H_{27}N_5O_8Na$ requires 536.1752).

10. Synthesis of *N*-Boc-Ile^{8}-Oxz^{9}-Oxz^{10}-Oxz^{11}-Oxz^{12}-L-*allo*-Thr-Phe^{14}OTMSE 3

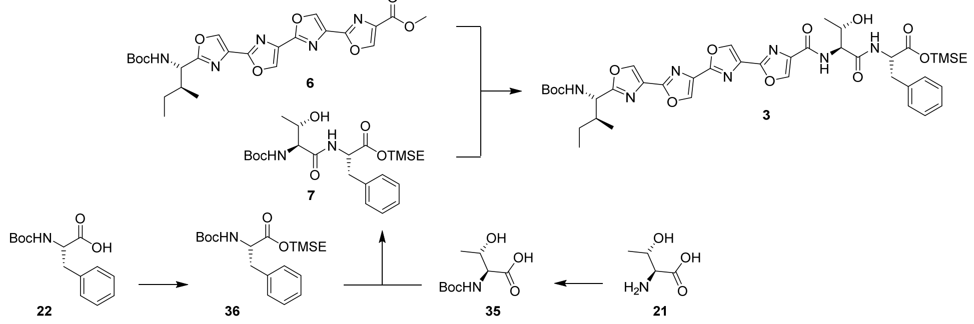

10.1. *N*-Boc-Phe^{14}OTMSE 36

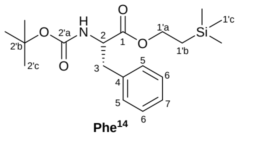

A solution of *N*-Boc-Phe-OH **22** (2.00 g, 7.5 mmol), *N*-(3-dimethylaminopropyl)-*N′*-ethylcarbodiimide hydrochloride (1.51 g, 7.9 mmol) and trimethylsilylethanol (1.13 mL, 7.9 mmol) in dichloromethane (16 mL) was cooled to 0 °C and 4-(dimethylamino)pyridine (0.092 g, 0.8 mmol) was added. The reaction mixture was warmed to room temperature and stirred for 18 h before diluting with dichloromethane (30 mL) and washing successively with water (20 mL), aqueous hydrochloric acid (20 mL, 3M), saturated sodium bicarbonate (20 mL) and saturated sodium chloride (20 mL). The combined aqueous layers were extracted with dichloromethane (3 x 20 mL) and the combined organic layers dried over magnesium sulfate before the solvent was removed *in vacuo*. The crude oil was purified by flash chromatography using 40-60 petroleum ether:ethyl acetate (9:1) as eluent to afford the title compound **36** (2.05 g, 5.6 mmol, 74%) as a colourless oil.

R*f* = 0.35 [40-60-petroleum ether:ethyl acetate = 9:1]; $[a]_D^{26.2}$ = +22.0 (*c* = 1.00, $CHCl_3$); **IR** (neat)/cm^{-1} 3371, 2955, 1714, 1497, 1249, 1168, 1053, 858, 836, 698; **^{1}H-NMR** ($CDCl_3$, 500 MHz) δ = 7.32 – 7.21 (m, 3H, 2 x Phe^{14}-C6H, Phe^{14}-C7H), 7.14 (d, *J* = 6.9 Hz, 2H, 2 x Phe^{14}-C5H), 4.97 (d, *J* = 8.6 Hz, 1H, Phe^{14}-NH), 4.54 (d, *J* = 6.9 Hz, 1H, Phe^{14}-C2H), 4.18 (dt, *J* = 9.2, 7.5 Hz, 2H, Phe^{14}-C1′aH_2), 3.11 (dd, *J* = 13.7, 5.7 Hz, 1H, Phe^{14}-C3H*H*), 3.04 (dd, *J* = 13.6, 6.0 Hz, 1H, Phe^{14}-C3*H*H), 1.41 (s, 3H, Phe^{14}-C2′cH_3), 0.95 (ddd, *J* = 10.1, 7.2, 1.7 Hz, 2H, Phe^{14}-C1′bH_2), 0.03 (s, 3H, Phe^{14}-C1′cH_3); **^{13}C-NMR** ($CDCl_3$, 126 MHz) δ = 171.9 (quat., Phe^{14}-C1), 155.1 (quat., Phe^{14}-C2′a), 136.2 (quat., Phe^{14}-C4), 129.4 (CH, Phe^{14}-C5), 128.5 (CH, Phe^{14}-C6), 126.9 (CH, Phe^{14}-C7), 79.8 (quat., Phe^{14}-C2′b), 63.7 (CH_2, Phe^{14}-C1′a), 54.5 (CH, Phe^{14}-C2), 38.4 (CH_2, Phe^{14}-C3), 28.3 (CH_3, Phe^{14}-C2′c), 17.3 (CH_2, Phe^{14}-C1′b), -1.6 (CH_3, Phe^{14}-C1′c); **HRMS** (ESI) found: 388.1905 ($[M+Na]^+$ $C_{19}H_{31}NO_4SiNa$ requires 388.1915). The data obtained was in agreement with that reported in the literature.[7]

10.2. *N*-Boc-L-*allo*-Thr-Phe^{14}OTMSE 7

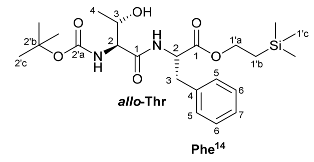

Sodium bicarbonate (0.53 g, 6.3 mmol) was added to a solution of L-*allo*-Thr-OH **21** (0.50 g, 4.2 mmol) in water (8 mL) and the reaction mixture was stirred at room temperature for 15 min before methanol (8 mL) then di-*tert*-butyl dicarbonate (1.45 mL, 6.3 mmol) were added and the reaction mixture was stirred at room temperature for 15.5 h. The solvent was removed *in vacuo* and hydrochloric acid (10 mL, 1M) was added before extracting with ethyl acetate (3 x 10 mL). The combined organic extracts were washed with saturated sodium chloride (10 mL) and dried over magnesium sulfate and the solvent was removed *in vacuo* to afford crude *N*-Boc-*allo*-threonine which was used directly in the coupling.

36 (1.53 g, 4.2 mmol) was dissolved in anhydrous hydrochloric acid (20 mL, 4M) and was stirred at room temperature for 2 h before the solvent was removed *in vacuo*. The crude was taken up in ethanol (2 x 20 mL) and methanol (20 mL), removing the solvent *in vacuo* after each addition to afford crude Phe-OTMSE.HCl, which was used directly in the coupling.

HATU (1.76 g, 4.62 mmol) was added to a solution of the crude *N*-Boc-*allo*-threonine and crude Phe-OTMSE·HCl in dichloromethane (60 mL) and the reaction mixture was cooled to 0 °C before diisopropylamine (2.2 mL, 12.6 mmol) was added. The reaction mixture was warmed to room temperature and was stirred at this temperature for 16 h. Water (60 mL) was added and the reaction mixture neutralized with aqueous hydrochloric acid (10 drops, 3M) before extracting with dichloromethane (3 x 60 mL). The combined organic layers were washed with saturated sodium chloride (60 mL) and were dried over magnesium sulfate before concentrating *in vacuo*. The crude oil was purified by flash chromatography using 40-60 petroleum ether:ethyl acetate (2:1) as eluent to afford the title compound **7** (1.58 g, 3.4 mmol, 81%) as a colourless solid, mp = 83.5 °C.

R_f = 0.18 [40-60 petroleum ether:ethyl acetate = 2:1]; $[a]_D^{26.9}$ = +0.5 (*c* = 1.00, $CHCl_3$); **IR** (neat)/cm^{-1} 3283, 2957, 1741, 1692, 1653, 1525, 1170, 1042, 1016, 858, 835, 679; **^{1}H-NMR** ($CDCl_3$, 500 MHz) δ = 7.33 – 7.22 (m, 3H, Phe^{14}-2 x C6H, C7H), 7.14 (dd, *J* = 6.8, 1.6 Hz, 2H, 2 x Phe^{14}-C5H), 6.57 (d, *J* = 7.8 Hz, 1H, Phe^{14}-NH), 5.38 (d, *J* = 8.1 Hz, 1H, Thr-NH), 4.80 (q, *J* = 7.0 Hz, 1H, Phe^{14}-C2H), 4.27 – 4.14 (m, 2H, Phe^{14}-C1′aH_2), 3.96 (t, *J* = 6.3 Hz, 1H, Thr-C2H), 3.83 (p, *J* = 6.3 Hz, 1H, Thr-C3H), 3.15 (dd, *J* = 14.0, 5.5 Hz, 1H, Phe^{14}-C3H*H*), 3.06 (dd, *J* = 14.0, 7.0 Hz, 1H, Phe^{14}-C3*H*H), 1.44 (s, 3H, Thr-2′cH_3), 1.23 (d, *J* = 6.3 Hz, 3H, Thr-C4H_3), 1.01 – 0.93 (m, 2H, Phe^{14}-C1′bH_2), 0.04 (s, 9H, Phe^{14}-C1′cH_3); **^{13}C-NMR** ($CDCl_3$, 126 MHz) δ = 171.5 (quat., Phe^{14}-C1), 171.0 (quat., Thr-C1), 155.8 (quat., Thr-C2′a), 135.6 (quat., Phe^{14}-C4), 129.2 (CH, Phe^{14}-C5), 128.7 (CH, Phe^{14}-C6), 127.3 (CH, Phe^{14}-C7), 80.3 (quat., Thr-C2′b), 69.5 (CH, Thr-C3), 64.3 (CH_2, Phe^{14}-C1′a), 58.5 (CH, Thr-C2), 53.5 (CH, Phe^{14}-C2), 37.6 (CH_2, Phe^{14}-C3), 28.3 (CH_3, Thr-C2′c), 19.7 (CH_3, Thr-C4), 17.4 (CH_2, Phe^{14}-C1′b), -1.6 (CH_3, Phe^{14}-C1′c); **HRMS** (ESI) found: 468.2636 ($[M+H]^+$ $C_{23}H_{40}N_2O_6Si$ requires 468.2650).

10.3 *N*-Boc-Ile^8-Oxz^9-Oxz^{10}-Oxz^{11}-Oxz^{12}-L-*allo*-Thr-Phe^{14}OTMSE 3

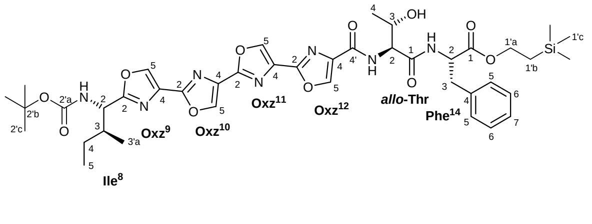

Hydrochloric acid (1 mL, 4M in 1,4-dioxane) was added to dipeptide **7** (0.038 g, 0.07 mmol) at 0 °C and the reaction mixture was stirred at room temperature for 30 min. The solvent volatiles was removed *in vacuo* to afford the amine as the hydrochloric acid salt which was used directly in the next step.

Lithium hydroxide monohydrate (0.021 g, 0.5 mmol) was added to a solution of methyl ester **6** (0.051 g, 0.1 mmol) in chloroform (9 mL), methanol (3 mL) and water (1 mL) at 0 °C. The reaction mixture was stirred at 65 °C for 48 h, over which time further portions of lithium hydroxide monohydrate (5 x 0.021 g, 0.5 mmol) were added before acidified to pH 2 - 4 with aqueous hydrochloric acid (3M) and the solvent was removed *in vacuo*. Ethyl acetate (10 mL) and water (10 mL) were added and the aqueous layer was extracted with ethyl acetate (4 x 20 mL). The combined organic extracts were washed with saturated sodium chloride (20 mL) and dried over sodium sulfate before the solvent was removed *in vacuo* to afford the free acid which was used directly in the next step.

The crude amine (0.07 mmol) and the crude acid (0.1 mmol) were combined and taken up in dichloromethane (4 mL) and *N*,*N*-dimethylformamide (0.14 mL). HATU (0.030 g, 0.08 mmol) was added and the suspension was cooled to 0 °C before diisopropylethylamine (0.038 mL, 0.22 mmol) was added. The reaction was stirred at room temperature for 18 h before quenching with saturated ammonium chloride (5 mL). The reaction mixture was extracted with dichloromethane (3 x 10 mL) and the combined organic layers were washed successively with hydrochloric acid (10 mL, 0.5M), water (10 mL), saturated sodium bicarbonate (10 mL) and saturated sodium chloride (10 mL) and were dried over sodium sulfate before the solvent was removed *in vacuo*. The crude oil was purified by flash chromatography using *n*-hexane:ethyl acetate (1:1 → neat ethyl acetate) as eluent to afford the *title compound* **3** (0.046 g, 0.054 mmol, 77%) as an off-white solid.

R_f = 0.20 [*n*-Hexane:ethyl acetate = 1:2]; $[a]_D^{28.5}$ = -12.9 (*c* = 1.00, $CHCl_3$); **IR** (neat)/cm^{-1} 3340, 2968, 1689, 1648, 1516, 1249, 1169, 1121, 1104, 977, 915, 835; **^{1}H-NMR** ($CDCl_3$, 500 MHz) δ = 8.46 (s, 1H, Oxz^{11}-C5H), 8.37 (s, 1H, Oxz^{10}-C5H), 8.32 (s, 1H, Oxz^{9}-C5H), 8.26 (s, 1H, Oxz^{12}-C5H), 7.76 (d, *J* = 8.4 Hz, 1H, Thr-NH), 7.20 – 7.11 (m, 3H, 2 x Phe^{14}-C6H, Phe^{14}-C7H), 7.11 – 7.07 (m, 2H, 2 x Phe^{14}-C5H), 6.85 (d, *J* = 8.0 Hz, 1H, Phe^{14}-NH), 5.34 (d, *J* = 9.4 Hz, 1H, Ile^{8}-NH), 4.92 (dd, *J* = 9.1, 6.0 Hz, 1H, Ile^{8}-C2H), 4.82 (ddd, *J* = 8.1, 7.1, 5.4 Hz, 1H, Phe^{14}-C2H), 4.50 (dd, *J* = 8.5, 5.4 Hz, 1H, Thr-C2H), 4.30 – 4.16 (m, 2H, Phe^{14}-C1′aH_2), 3.99 (s, 1H, Thr-C3H), 3.89 (s, 1H, Thr-C3OH), 3.17 (dd, *J* = 14.0, 5.5 Hz, 1H, Phe^{14}-C3H*H*), 3.05 (dd, *J* = 14.0, 7.1 Hz, 1H, Phe^{14}-C3*H*H), 2.03 – 1.95 (m, 1H, Ile^{8}-C3H), 1.55 – 1.47 (m, 1H, Ile^{8}-C4*H*H), 1.44 (s, 9H, Ile^{8}-2′cH_3), 1.30 (d, *J* = 6.4 Hz, 3H, Thr-C4H_3), 1.27 – 1.17 (m, 1H, Ile^{8}-C4*H*H), 0.99 (ddd, *J* = 9.9, 7.1, 1.3 Hz, 3H, Ile^{8}-C5H_3), 0.93 (t, *J* = 7.4 Hz, 2H, Phe^{14}-C1′bH_2), 0.91 (d, *J* = 6.8 Hz, 3H, Ile^{8}-C3′aH_3), 0.05 (s, 9H, Phe^{14}-C1′cH_3); **^{13}C-NMR** ($CDCl_3$, 126 MHz) δ = 171.5 (quat., Phe^{14}-C1), 170.2 (quat., Thr-C1), 165.7 (quat., Oxz^{9}-C2), 160.5 (quat., Oxz^{12}-C4′a), 156.3 (quat., Oxz^{11}-C2), 156.0 (quat., Oxz^{10}-C2), 155.3 (quat., Ile^{8}-C2′a), 154.5 (quat., Oxz^{12}-C2), 141.6 (CH, Oxz^{12}-C5), 139.4 (CH, Oxz$^{10 \text{ and } 11}$-C5), 139.3 (CH, Oxz^{9}-C5), 136.6 (quat., Oxz^{12}-C4), 135.5 (quat., Phe^{14}-C4), 130.9 (quat., Oxz^{10}-C4), 130.7 (quat., Oxz^{11}-C4), 129.6 (quat., Oxz^{9}-C4), 129.1 (CH, Phe^{14}-C5), 128.6 (CH, Phe^{14}-C6), 127.1 (CH, Phe^{14}-C7), 80.1 (quat., Ile^{8}-C2′b), 69.1 (CH, Thr-C3), 64.3 (CH_2, Phe^{14}-C1′a), 58.5 (CH, Thr-C2), 53.44 (CH, Phe^{14}-C2), 53.38 (CH, Ile^{8}-C2), 39.5 (CH, Ile^{8}-C3), 37.4 (CH_2, Phe^{14}-C3), 28.3 (CH_3, Ile^{8}-C2′c), 25.1 (CH_2, Ile^{8}-C4), 19.8 (CH_3, Thr-C4), 17.4 (CH_2, Phe^{14}-C1′b), 15.2 (CH_3, Ile^{8}-C3′a), 11.4 (CH_3, Ile^{8}-C5), -1.6 (CH_3, Phe^{14}-C1′c); **HRMS** (ESI) found: 848.3615 ($[M+H]^+$ $C_{41}H_{54}N_7O_{11}Si$ requires 848.3645).

11. Synthesis of Plantazolicin A 1a

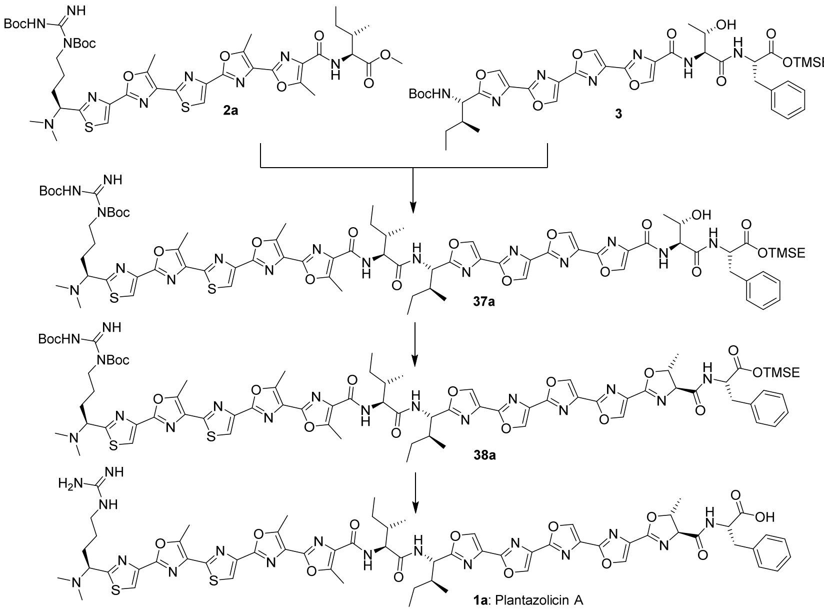

11.1. *N*-Me$_2$-Arg^1(Boc)$_2$-Thz^2-5-MeOxz^3-Thz^4-5-MeOxz^5-5-MeOxz^6-Ile^7-Ile^8-Oxz^9-Oxz^{10}-Oxz^{11}-Oxz^{12}-L-*allo*-Thr-Phe^{14}OTMSE 37a

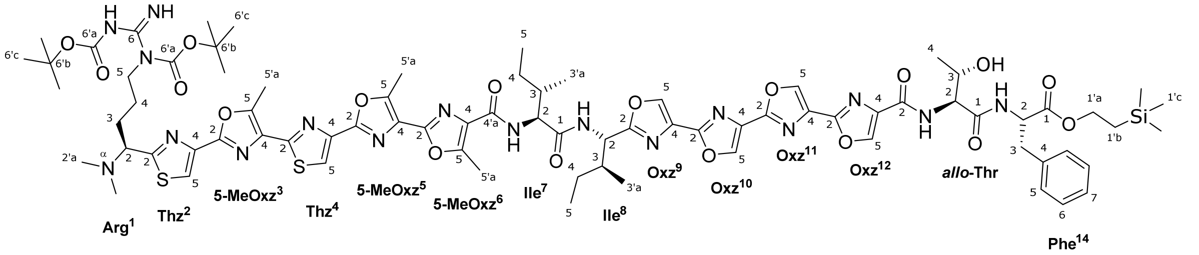

2a (0.038 g, 0.041 mmol) was taken up in tetrahydrofuran (2 mL) and was cooled to 0 °C and aqueous lithium hydroxide (2 mL, 1M) was added. The reaction mixture was stirred at 0 °C for 2.25 h before it was neutralised at 0 °C using aqueous hydrochloric acid (~30 drops, 3M). The reaction mixture was warmed to room temperature and was extracted with ethyl acetate (3 x 10 mL) before the combined organics were washed with saturated sodium chloride (5 mL) and dried over magnesium sulfate before the solvent was removed *in vacuo* to afford the crude acid, which was used directly in the coupling.

3 (0.035g, 0.041 mmol) was cooled to 0 °C and anhydrous hydrochloric acid (1 mL, 4M in dioxane) was added. The reaction mixture was stirred at 0 °C for 5 min then warmed to room temperature for a further 30 min. The solvent was removed *in vacuo* and then ethanol (2 x 10 mL) and methanol (10 mL) were added successively, removing the solvent *in vacuo* after each addition to give the crude amine as the hydrochloride salt, which was used directly in the coupling.

The crude amine and crude acid were taken up in dichloromethane (3 mL) and *N,N*-dimethylformamide (0.3 mL) and HATU (0.017 g, 0.045 mmol) was added before cooling the reaction mixture to 0 °C. Diisopropylethylamine (0.021 mL, 0.12 mmol) was added and the reaction mixture warmed to room temperature and stirred for 16 h. The reaction mixture was neutralised using aqueous hydrochloric acid (1 drop, 3M) before water (5 mL) was added and the reaction mixture extracted with ethyl acetate (3 x 10 mL). The combined organic layers were washed with saturated sodium chloride (5 mL) and were dried over magnesium sulfate before the solvent was removed *in*

vacuo. The resulting oil was purified by preparative thin layer chromatography twice using dichloromethane:methanol (19:1 (R_f = 0.21) then 9:1 (R_f = 0.49)) eluent to afford *crude title compound* **37a** (0.037 g, ≤0.022 mmol) as a colourless foam, which was used directly in the next step without further purification. **HRMS** (ESI) found: 1654.6642 ($[M+H]^+$ $C_{78}H_{99}N_{17}O_{18}S_2Si$ requires 1654.6642).

11.2. *N*-Me$_2$-Arg^1(Boc)$_2$-Thz^2-5-MeOxz^3-Thz^4-5-MeOxz^5-5-MeOxz^6-Ile^7-Ile^8-Oxz^9-Oxz^{10}-Oxz^{11}-Oxz^{12}-5-MeOxl^{13}-Phe^{14}OTMSE 38a

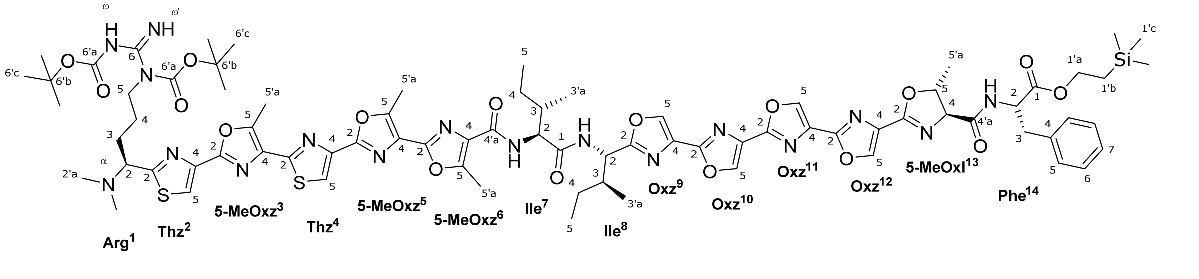

37a (0.037 g, ≤0.022 mmol) was taken up in dichloromethane (3 mL) and was cooled to -20 °C before a solution of Deoxo-Fluor® (0.24 mL, 0.1M in dichloromethane, 0.024 mmol) was added. The reaction mixture was stirred at -20 °C for 24 h before saturated sodium bicarbonate (5 mL) was added and the reaction mixture was extracted with dichloromethane (3 x 10 mL). The combined organic extracts were washed with saturated sodium chloride (5 mL) and dried over magnesium sulfate before the solvent was removed *in vacuo*. The resulting crude was purified by preparatory thin layer chromatography using dichloromethane:methanol (19:1) eluent to afford the *title compound* **38a** (0.029 g, 0.018 mmol, 43%) as a colourless foam.

R_f = 0.49 [dichloromethane:methanol = 19:1] ; $[a]_D^{23.3}$ = -2.1 (*c* = 0.5, $CHCl_3$); **IR** (neat)/cm^{-1} 3369, 2924, 1710, 1663, 1645, 1610, 1510, 1251, 1448, 1098, 977, 807; **^{1}H-NMR** ($CDCl_3$, 500 MHz) δ = 9.34 (s, 1H, Arg^1-NωH or Arg^1-Nω′H), 9.18 (s, 1H, Arg^1-NωH or Arg^1-Nω′H), 8.50 (s, 1H, Oxz^{11}-C5H), 8.44 (s, 1H, Oxz^{10}-C5H), 8.34 (s, 1H, Oxz^9-C5H), 8.21 (s, 1H, Oxz^{12}-C5H), 8.11 (s, 1H, Thz^4-C5H), 8.03 (s, 1H, Thz^2-C5H), 7.49 (d, *J* = 8.9 Hz, 1H, Ile^7-NH), 7.15 – 7.13 (m, 3H, 2 x Phe^{14}-C6H, Phe^{14}-C7H), 7.09 – 7.00 (m, 3H, 2 x Phe^{14}-C5H, Phe-NH), 6.80 (d, *J* = 8.6 Hz, 1H, Ile^8-NH), 5.24 (dd, *J* = 8.6, 6.2 Hz, Ile^8-C2H), 4.82 (ddd, *J* = 8.7, 7.4, 5.9 Hz, 1H, Phe^{14}-C2H), 4.75 – 4.70 (m, 1H, 5-MeOxl^{13}-C5H), 4.46 (dd, *J* = 8.9, 7.5 Hz, 1H, Ile^7-C2H), 4.30 (d, *J* =7.6 Hz, 1H, 5-MeOxl^{13}-C4H), 4.28 – 4.14 (m, 2H, Phe^{14}-C1′aH$_2$), 4.04 – 3.87 (s, 3H, Arg^1-C2H and Arg^1-C5H$_2$), 3.13 (dd, *J* = 13.7, 5.9 Hz, Phe^{14}-C3H*H*), 2.99 (dd, *J* = 13.7, 7.4 Hz, Phe^{14}-C3*H*H), 2.92 (s, 3H, 5-MeOxz^3-C5′aH3), 2.81 (s, 3H, 5-MeOxz^5-C5′aH3), 2.74 (s, 3H, 5-MeOxz^6-C5′aH3), 2.35 (s, 6H, 2 x Arg-NαCH$_3$), 2.09 – 1.98 (m, 4H, Arg^1-C3H$_2$, Ile^7-C3H and Ile^8-C3H), 1.67 – 1.57 (m, 2H, Arg^1-C4H$_2$), 1.53 (d, *J* = 6.2 Hz, 3H, C5′aH$_3$), 1.50 – 1.42 (m, 20H, 6 x Arg^1-C6′cH$_3$ and Ile^7-C4H$_2$), 1.37 – 1.25 (m, 2H, Ile^8-C4H$_2$), 1.02 – 0.80 (m, 14H, Ile^7-C3′aH$_3$, Ile^7-C5H$_3$, Ile^8-C3′aH$_3$, Ile^8-C5H$_3$ and Phe^{14}-C1′bH$_2$), 0.04 (s, 9H, Phe^{14}-C1′cH$_3$); **^{13}C-NMR** ($CDCl_3$, 126 MHz) δ =175.4 (quat., Ile^7-C1), 174.5 (quat., Thz^2-C2), 171.1 (quat., Phe^{14}-C1), 170.8 (quat., 5-MeOxz^6-C4′a), 170.4 (quat., 5-MeOxl^{13}-C4′a), 164.8 (quat., Oxz^9-C2), 163.8 (quat., Arg^1-C6′a), 162.4 (quat., Thz^4-C2), 162.0 (quat., 5-MeOxz^6-C2), 160.5 (quat., Arg^1-C6), 158.5 (quat., 5-MeOxl^{13}-C2), 156.22 (quat., 5-MeOxz^3-C2), 156.16 (quat., Oxz^{10}-C2), 155.9 (quat., Oxz^{11}-C2), 155.5 (quat., Oxz^{12}-C2), 154.9 (quat., Arg^1-C6′a), 153.2 (quat., 5-MeOxz^6-C5), 153.1 (quat., 5-MeOxz^5-C2), 150.5 (quat., 5-MeOxz^5-C5), 148.1 (quat., 5-MeOxz^3-C5), 143.5 (quat., Thz^4-C4), 142.4 (quat., Thz^2-C4), 141.4 (CH, Oxz^{12}-C5), 139.5 (CH, Oxz^{11}-C5), 139.4 (CH, Oxz^9-C5), 139.3 (CH, Oxz^{10}-C5), 135.9 (quat., Phe^{14}-C4), 131.6 (quat., Oxz^{12}-C4), 131.0 (quat., Oxz^{11}-C4), 130.8 (quat., 5-MeOxz^6-C4), 130.0 (quat., Oxz^9-C4), 129.7(quat., 5-MeOxz^3-C4 and

Oxz^{10}-C4), 129.6 (CH, Phe^{14}-C7), 129.3 (CH, Phe^{14}-C6), 128.3 (CH, Phe^{14}-C5), 126.9 (quat., 5-MeOxz^{5}-C4), 120.6 (CH, Thz^{2}-C5), 120.3 (CH, Thz^{4}-C5), 83.7 (quat., Arg^{1}-C6′b), 80.3 (CH, 5-MeOxl^{13}-C5H), 78.6 (quat., Arg^{1}-C6′b), 74.9 (CH, 5-MeOxl^{13}-C4H), 66.70 (CH, Arg^{1}-C2), 63.9 (CH_2, Phe^{14}-C1′a), 57.5 (CH, Ile^{7}-C2), 53.0 (CH, Phe^{14}-C2), 51.9 (CH, Ile^{8}-C2), 44.4 (CH_2, Arg^{1}-C5), 42.1 (CH_3, 2 x Arg^{1}-NαC), 38.9 (CH, Ile^{8}-C3), 38.2 (CH_2, Phe^{14}-C3), 36.6 (CH, Ile^{7}-C3), 28.3 (CH_3, 3 x Arg^{1}-C6′c), 28.0 (CH_3, 3 x Arg^{1}-C6′c), 27.2 (CH_2, Arg^{1}-C3), 25.5 (CH_2, Arg^{1}-C4), 25.2 (CH_2, Ile^{7}-C4), 24.9 (CH_2, Ile^{8}-C4), 21.7 (CH_3, 5-MeOxl^{13}-C5′a), 17.4 (CH_2, Phe^{14}-C1′b), 15.7, 15.2 (CH_3, Ile^{7+8}-C3′a), 12.3 (CH_3, 5-MeOxz^{3}-C5′a), 12.0 (CH_3, 5-MeOxz^{5}-C5′a), 11.8 (CH_3, 5-MeOxz^{6}-C5′a), 11.3, 11.2 (CH_3, Ile^{7+8}-C5), -1.5 (CH_3, Phe^{14}-C1′c); **HRMS** (ESI) found: 1636.6599 ($[M+H]^+$ $C_{78}H_{98}N_{17}O_{17}S_2Si$ requires 1636.6537).

11.3. Plantazolicin A (*N*-Me$_2$-Arg^{1}-Thz^{2}-5-MeOxz^{3}-Thz^{4}-5-MeOxz^{5}-5-MeOxz^{6}-Ile^{7}-Ile^{8}-Oxz^{9}-Oxz^{10}-Oxz^{11}-Oxz^{12}-5-MeOxl^{13}-Phe^{14}OH) 1a

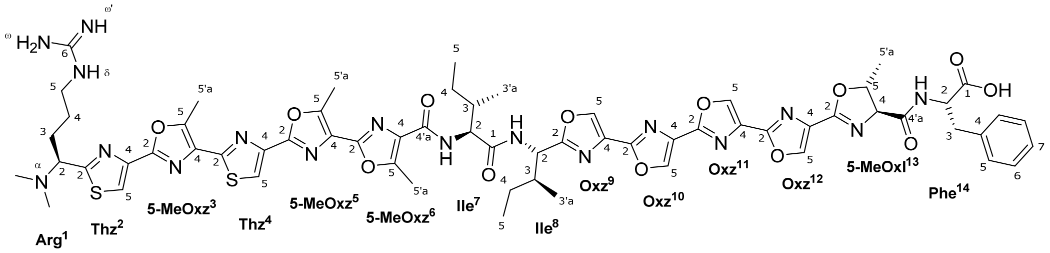

Trifluoroacetic acid (0.5 mL) was added to **38a** (8.5 mg, 5.2 x 10^{-3} mmol) and the reaction mixture was stirred at room temperature for 2 h before the solvent was removed *in vacuo*. Ethanol (2 x 2 mL) then methanol (2 mL) were added successively, removing the solvent *in vacuo* after each addition. The resulting crude was purified by HPLC to afford the *title compound* **1a** (4.1 mg, 3.1 x 10^{-3} mmol, 59%) as a colourless amorphous solid.

R$_t$ = 10.936 minutes (**R$_t$ natural sample*** = 10.921 minutes); $[a]_D^{26.4}$ = -1.6 (*c* = 0.1, 80% MeCN in H_2O)) (literature[8] $[\alpha]_D^{23}$ -1.2 (c 0.1, MeCN/H_2O)); **IR** (neat)/cm^{-1} 3357, 2923, 2853, 1659, 1632, 1540, 1488, 1425, 1411, 1104, 803, 698; **^{1}H-NMR**# (DMSO-d$_6$, 500 MHz) δ = 9.10 (s, 1H, Oxz^{11}-C5H), 9.08 (s, 1H, Oxz^{10}-C5H), 8.97 (s, 1H, Oxz^{9}-C5H), 8.83 (s, 1H, Oxz^{12}-C5H), 8.81 (d, *J* = 7.5 Hz, 1H, Ile^{8}-NH), 8.65 (br. s., 1H, Arg^{1}-NδH), 8.46 (s, 1H, Thz^{4}-C5H), 8.42 (s, 1H, Thz^{2}-C5H), 7.91 (d, *J* = 9.4 Hz, 1H, Ile^{7}-NH), 7.80 (br. s., 3H, Arg^{1}-NωH_2 and Arg^{1}-Nω′H), 7.12 (d, *J* = 7.2 Hz, 1H, Phe^{14}-NH), 7.08 – 6.94 (m, 5H, 2 x Phe^{14}-C5H, 2 x Phe^{14}-C6H, Phe^{14}-C7H), 4.94 (t, *J* = 7.8 Hz, 1H, Ile^{8}-C2H), 4.67 – 4.58 (m, 1H, 5-MeOxl^{13}-C5H), 4.48 (t, *J* = 8.7 Hz, 1H, Ile^{7}-C2H), 4.25 (d, *J* = 7.7 Hz, 1H, 5-MeOxl^{13}-C4H), 4.17 (q, *J* = 5.7 Hz, 1H, Phe^{14}-C2H), 3.96 (t, *J* = 7.1 Hz, 1H, Arg^{1}-C2H), 3.19 – 3.13 (m, 2H, Arg^{1}-C5H_2), 3.04 (dd, *J* = 13.1, 5.4 Hz, 1H, Phe^{14}-C3H*H*), 2.94 (dd, *J* = 13.1, 5.4 Hz, 1H, Phe^{14}-C3*H*H), 2.83 (s, 3H, 5-MeOxz^{3}-C5′aH_3), 2.75 (s, 3H, 5-MeOxz^{5}-C5′aH_3), 2.65 (s, 3H, 5-MeOxz6-C5′aH_3), 2.29 (s, 6H, 2 x Arg-NαCH_3), 2.12 – 1.91 (m, 3H, Arg^{1}-C3H*H*, Ile^{7}-C3H and Ile^{8}-C3H), 1.93 – 1.84 (m, 1H, Arg^{1}-C3H*H*), 1.63 – 1.54 (m, 3H, Arg^{1}-C4H_2 and Ile^{A}-C4H*H*), 1.49 – 1.41 (m, 1H, Ile^{B}-C4H*H*), 1.47 (d, *J* = 6.2 Hz, 3H, 5-MeOxl^{13}-C5′aH_3), 1.32 – 1.24 (m 1H, Ile^{A}-C4H*H*), 1.09 (dt, *J* = 14.6, 7.7 Hz, 1H, Ile^{B}-C4H*H*), 0.97 – 0.79 (m, 12H, Ile^{7}-C3′aH_3, Ile^{7}-C5H_3, Ile^{8}-C3′aH_3, Ile^{8}-C5H_3); **^{13}C-NMR**# ($CDCl_3$, 126 MHz) δ = 174.0 (quat., Phe^{14}-C1), 173.9 (quat., Thz^{2}-C2), 171.2 (quat., Ile^{7}-C1), 169.0 (quat., 5-MeOxl^{13}-C4′a), 164.6 (quat., Oxz^{9}-C2), 161.7 (quat., Thz^{4}-C2), 160.6 (quat., 5-MeOxz^{6}-C4′a), 157.4 (quat., 5-MeOxl^{13}-C2), 157.3 (quat., 5-MeOxz^{6}-C2), 155.7 (quat., Oxz^{10}-C2), 155.4 (quat., 5-MeOxz^{3}-C2 and Oxz^{11}-C2), 155.3 (quat., 5-MeOxz^{5}-C2), 155.0 (quat., Oxz^{12}-C2), 152.6 (quat., 5-MeOxz^{6}-C5), 150.8 (quat., 5-MeOxz^{5}-C5), 147.7 (quat., 5-MeOxz^{3}-C5), 142.9 (quat., Thz^{4}-C4), 142.8 (CH, Oxz^{12}-C5), 141.2 (quat., Thz^{2}-C4), 141.1 (CH, Oxz^{11}-C5), 140.94 (CH, Oxz^{10}-C5), 140.90 (CH, Oxz^{9}-C5), 138.4 (quat., Phe^{14}-C4), 130.8 (quat., Oxz^{12}-C4),

130.1 (quat., Oxz^{11}-C4), 130.0 (quat., 5-MeOxz^{3}-C4 and Oxz^{10}-C4), 129.7 (quat., Arg^{1}-C6), 129.5 (CH, Phe^{14}-C5), 129.3 (quat., 5-MeOxz^{6}-C4), 128.8 (quat., Oxz^{9}-C4), 127.6 (CH, Phe^{14}-C6), 125.7 (CH, Phe^{14}-C7), 125.1 (quat., 5-MeOxz^{5}-C4), 122.6 (CH, Thz^{4}-C5), 121.7 (quat., Thz^{2}-C5), 79.8 (CH, 5-MeOxl^{13}-C5), 74.6 (CH, 5-MeOxl^{13}-C4), 65.6 (CH, Arg^{1}-C2), 57.0 (CH, Ile^{7}-C2), 54.7 (CH, Phe^{14}-C2), 51.7 (CH, Ile^{8}-C2), 41.5 (CH_3, 2 x Arg^{1}-NαC), 40.5 (CH_2, Arg^{1}-C5), 37.5 (CH_2, Phe^{14}-C3), 37.1 (CH, Ile^{8}-C3), 37.0 (CH, Ile^{7}-C3), 28.7 (CH_2, Arg^{1}-C3), 26.0 (CH_2, Arg^{1}-C4), 25.1 (CH_2, Ile^{8}-C4), 24.5 (CH_2, Ile^{7}-C4), 21.3 (CH_3, 5-MeOxl^{13}-C5′a), 15.5 (CH_3, Ile^{7}-C3′a), 15.4 (CH_3, Ile^{8}-C3′a), 11.8 (CH_3, 5-MeOxz^{3}-C5′a), 11.7 (CH_3, 5-MeOxz^{5}-C5′a), 11.4 (CH_3, 5-MeOxz^{6}-C5′a), 10.9 (CH_3, Ile^{7+8}-C5); **HRMS** (ESI) found: 1336.4786 ($[M+H]^+$ $C_{63}H_{70}N_{17}O_{13}S_2$ requires 1336.4780).

* Provided by Professor Douglas Mitchell (University of Illinois at Urbana-Campaign), Molohon *et al.*, *ACS Chem. Biol.*, **2011**, *6*, 1037 – 1313

\# Comparison of ^{1}H and ^{13}C data to previously reported values is reported later in the supporting information.

12. Synthesis of Plantazolicin B 1b

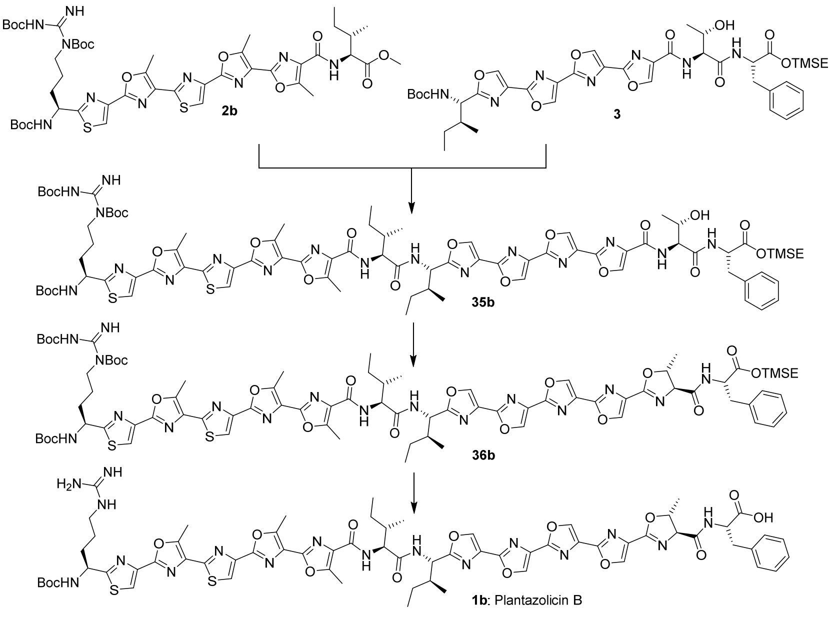

12.1. *N*-Boc$_2$-Arg^{1}(Boc)$_2$-Thz^{2}-5-MeOxz^{3}-Thz^{4}-5-MeOxz^{5}-5-MeOxz^{6}-Ile^{7}-Ile^{8}-Oxz^{9}-Oxz^{10}-Oxz^{11}-Oxz^{12}-L-*allo*-Thr-Phe^{14}OTMSE 37b

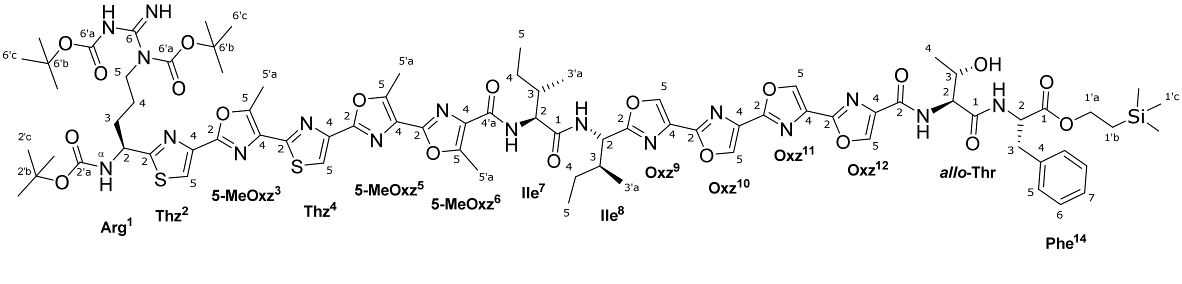

2b (0.047 g, 0.041 mmol) was taken up in tetrahydrofuran (2 mL) and cooled to 0 °C before aqueous lithium hydroxide (2 mL, 1M) was added. The reaction mixture was stirred at this temperature for 2.25 h before carefully neutralising at 0 °C using aqueous hydrochloric acid (~25 drops, 3M) before warming to room temperature. The reaction mixture was extracted with ethyl acetate (3 x 10 mL) and

the combined organic extracts washed with saturated sodium chloride (5 mL) and dried over magnesium sulfate before the solvent was removed *in vacuo* and the resulting crude acid used directly in the coupling.

3 (0.035 g, 0.041 mmol) was cooled to 0 °C and anhydrous hydrochloric acid (1 mL, 4M in dioxane) was added. The reaction mixture was stirred at 0 °C for 5 min then warmed to room temperature for a further 30 min before the solvent was removed *in vacuo*. Ethanol (2 x 10 mL) and methanol (10 mL) were added successively, removing the solvent *in vacuo* after each addition to afford crude amine as the hydrochloride salt which was used directly in the coupling.

The crude acid and crude amine were taken up in dichloromethane (3 mL) and *N*,*N*-dimethylformamide (0.3 mL) and HATU (0.017 g, 0.045 mmol) was added before cooling the reaction mixture to 0 °C. Diisopropylethylamine (0.021 mL, 0.12 mmol) was added and the reaction mixture warmed to room temperature and stirred for 16 h. The reaction mixture was neutralised with aqueous hydrochloric acid (1 drop, 3M) and water (5 mL) was added before extracting with ethyl acetate (3 x 10 mL). The combined organic extracts were washed with saturated sodium chloride (5 mL) and were dried over magnesium sulfate before the solvent was removed *in vacuo*. The resulting oil was purified by preparative thin layer chromatography twice using dichloromethane:methanol (19:1 (R_f = 0.39) then 9:1 (R_f = 0.53)) eluent to afford *crude title compound* **37b** (0.026 g, ≤0.015 mmol) as a colourless foam, which was used directly in the next step without further purification. **HRMS** (ESI) found: 1726.6846 ($[M+H]^+$ $C_{81}H_{104}N_{17}O_{20}S_2Si$ requires 1726.6854).

12.2. *N*-Boc$_2$-Arg^1(Boc)$_2$-Thz^2-5-MeOxz^3-Thz^4-5-MeOxz^5-5-MeOxz^6-Ile^7-Ile^8-Oxz^9-Oxz^{10}-Oxz^{11}-Oxz^{12}-5-MeOxl^{13}-Phe^{14}OTMSE 38b

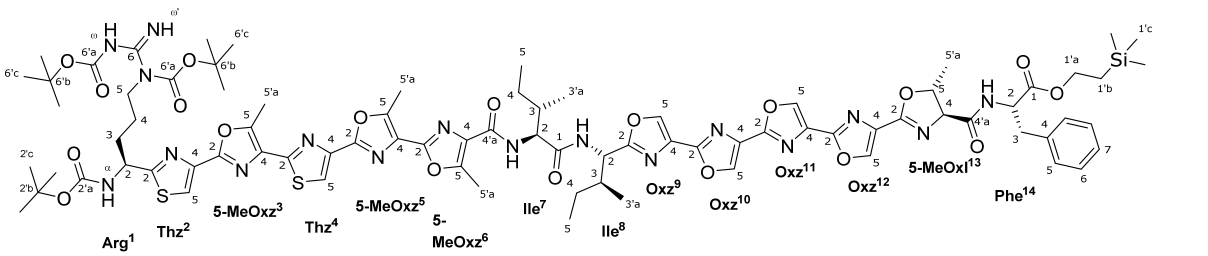

37b (0.026 g, ≤0.015 mmol) was taken up in dichloromethane (2 mL) and was cooled to – 20 °C and a solution of Deoxo-Fluor® (0.17 mL, 1M in dichloromethane, 0.017 mmol) was added and the reaction mixture was stirred at -20 °C for 17 hours. Saturated sodium bicarbonate (5 mL) was added and the reaction mixture warmed to room temperature before extracting with dichloromethane (3 x 10 mL). The combined organic extracts were washed with saturated sodium chloride (5 mL) and dried over magnesium sulfate before the solvent was removed *in vacuo*. The resulting crude was purified by preparatory thin layer chromatography twice using dichloromethane:methanol (9:1 then 19:1) eluent to afford the *title compound* **38b** (0.025g, 0.014 mmol, 35%) as a colourless foam.

R_f = 0.52/0.22 [dichloromethane:methanol = 9:1/19:1]; $[a]_D^{23.4}$ = -5.7 (*c* = 0.5, $CHCl_3$); **IR** (neat)/cm^{-1} 3361, 2960, 2923, 2849, 1711, 1685, 1647, 1516, 1260, 1095, 1016, 799; **^{1}H-NMR** ($CDCl_3$, 500 MHz) δ = 9.39 (s, 1H, Arg^1-NωH or Arg^1-Nω'H), 9.21 (s, 1H, Arg^1-NωH or Arg^1-Nω'H), 8.50 (s, 1H, Oxz^{11}-C5H), 8.44 (s, 1H, Oxz^{10}-C5H), 8.34 (s, 1H, Oxz^9-C5H), 8.21 (s, 1H, Oxz^{12}-C5H), 8.11 (s, 1H, Thz^4-C5H), 7.96 (s, 1H, Thz^2-C5H), 7.49 (d, *J* =8.9 Hz, 1H, Ile^7-NH), 7.19 – 7.12 (m, 3H, 2 x Phe^{14}-C6H, C7H), 7.09 – 7.03 (m, 2H, 2 x Phe^{14}-C5H), 7.03 (d, *J* =8.6 Hz, 1H, Phe^{14}-NH), 6.78 (d, *J* =8.7 Hz, 1H, Ile^8-NH), 6.31 (d, *J* =6.8 Hz, 1H, Arg^1-NαH), 5.26 – 5.24 (m, 1H, Ile^8-C2H), 5.13 (br. s, 1H, Arg^1-C2H), 4.87 – 4.79 (m, 1H, Phe^{14}-C2H), 4.78 – 4.69 (m, 1H, 5-MeOxl^{13}-C5H), 4.47 (dd, *J* =8.9,

7.5 Hz, 1H, Ile^{7}-C2H), 4.31 (d, J =7.6 Hz, 1H, 5-MeOxl^{13}-C4H), 4.29 – 4.14 (m, 2H, Phe^{14}-C1′aH$_2$), 4.13 – 4.04 (m, 1H, Arg^{1}-C5*H*H), 3.83 – 3.75 (s, 1H, Arg^{1}-C5*H*H), 3.14 (dd, J =13.7, 5.9 Hz, 1H, Phe^{14}-C3H*H*), 3.00 (dd, J =13.7, 7.4 Hz, 1H, Phe^{14}-C3*H*H), 2.92 (s, 3H, 5-MeOxz^{3}-C5′aH3), 2.81 (s, 3H, 5-MeOxz^{5}-C5′aH$_3$), 2.75 (s, 3H, 5-MeOxz^{6}-C5′aH$_3$), 2.13 – 1.97 (m, 4H, Arg^{1}-C3H$_2$, Ile^{7}-C3H and Ile^{8}-C3H), 1.84 – 1.74 (m, 2H, Arg^{1}-C4H$_2$), 1.54 (d, J =6.2 Hz, 3H, 5-MeOxl^{13}-C5′aH$_3$), 1.52 – 1.40 (m, 27H, 6 x Arg^{1}-C6′cH$_3$ and , 3 x Arg^{1}-C2′cH$_3$), 1.36 – 1.33 (m, 4H, Ile^{7}-C4H$_2$, Ile^{8}-C4H$_2$), 0.95 – 0.87 (m, 14H, Ile^{7}-C3′aH$_3$, Ile^{7}-C5H$_3$, Ile^{8}-C3′aH$_3$, Ile^{8}-C5H$_3$, Phe^{14}-C1′bH$_2$), 0.05 (s, 9H, Phe^{14}-C1′cH$_3$); **^{13}C-NMR** ($CDCl_3$, 126 MHz) δ = 176.0 (quat., Thz^{2}-C2), 175.4 (quat., Ile^{7}-C1), 171.1 (quat., Phe^{14}-C1), 170.8 (quat., 5-MeOxz^{6}-C4′a), 170.5 (quat., 5-MeOxl^{13}-C4′a), 164.8 (quat., Oxz^{9}-C2), 163.5 (quat., Arg^{1}-C6′a), 162.4 (quat., Thz^{4}-C2 and Arg^{1}-C2′a), 162.0 (quat., 5-MeOxz^{6}-C2), 160.8 (quat., Arg^{1}-C6), 158.5 (quat., 5-MeOxl^{13}-C2), 156.2 (quat., 5-MeOxz^{3}-C2), 156.2 (quat., Oxz^{10}-C2), 155.9 (quat., Oxz^{11}-C2), 155.6 (quat., Oxz^{12}-C2), 154.9 (quat., Arg^{1}-C6′a), 153.3 (quat., 5-MeOxz^{6}-C5), 153.1 (quat., 5-MeOxz^{5}-C2), 150.5 (quat., 5-MeOxz^{5}-C5), 148.1 (quat., 5-MeOxz^{3}-C5), 143.5 (quat., Thz^{4}-C4), 143.0 (quat., Thz^{2}-C4), 141.4 (CH, Oxz^{12}-C5), 139.5 (CH, Oxz^{11}-C5), 139.4 (CH, Oxz^{9}-C5), 139.3 (CH, Oxz^{10}-C5), 135.9 (quat., Phe^{14}-C4), 131.6 (quat., Oxz^{12}-C4), 131.0 (quat., Oxz^{11}-C4), 130.8 (quat., 5-MeOxz^{3}-C4 and Oxz^{10}-C4), 130.0 (quat., Oxz^{9}-C4), 129.7 (quat., 5-MeOxz^{6}-C4), 129.3 (CH, Phe^{14}-C5), 128.4 (CH, Phe^{14}-C6), 126.9 (CH, Phe^{14}-C7), 125.8 (quat., 5-MeOxz^{5}-C4), 120.3 (CH, Thz^{4}-C5), 120.1 (CH, Thz^{2}-C5), 83.9 (quat., Arg^{1}-C6′b), 80.3 (CH, 5-MeOxl^{13}-C5), 79.9 (quat., Arg^{1}-C2′b), 78.9 (quat., Arg^{1}-C6′b), 74.9 (CH, 5-MeOxl^{13}-C4), 63.9 (CH_2, Phe^{14}-C1′a), 57.5 (CH, Ile^{7}-C2), 53.7 (CH, Arg^{1}-C2), 53.0 (CH, Phe^{14}-C2), 51.9 (CH, Ile^{8}-C2), 44.2 (CH_2, Arg^{1}-C5), 38.9 (CH, Ile^{8}-C3), 38.2 (CH_2, Phe^{14}-C3), 36. 6 (CH, Ile^{7}-C3), 28.4 (CH_3, 3 x Arg^{1}-C2′c), 28.3 (CH_3, 3 x Arg^{1}-C6′c), 28.0 (CH_3, 3 x Arg^{1}-C6′c), 27.2 (CH_2, Arg^{1}-C3), 25.5 (CH_2, Arg^{1}-C4), 25.2 (CH_2, Ile^{7}-C4), 24.9 (CH_2, Ile^{8}-C4), 21.7 (CH_3, 5-MeOxl^{13}-C5′a), 17.4 (CH_2, Phe^{14}-C1′b), 15.7, 15.2 (CH_3, Ile^{7+8}-C3′a), 12.2 (CH_3, 5-MeOxz^{3}-C5′a), 12.0 (CH_3, 5-MeOxz^{5}-C5′a), 11.8 (CH_3, 5-MeOxz^{6}-C5′a), 11.3, 11.2 (CH_3, Ile^{7+8}-C5), -1.5 (CH_3, Phe^{14}-C1′c); **HRMS** (ESI) found: 1708.6772 ($[M+H]^+$ $C_{81}H_{102}N_{17}O_{19}S_2Si$ requires 1708.6749).

12.3. Plantazolicin B (NH_2-Arg^{1}-Thz^{2}-5-MeOxz^{3}-Thz^{4}-5-MeOxz^{5}-5-MeOxz^{6}-Ile^{7}-Ile^{8}-Oxz^{9}-Oxz^{10}-Oxz^{11}-Oxz^{12}-5-MeOxl^{13}-Phe^{14}OH) 1b

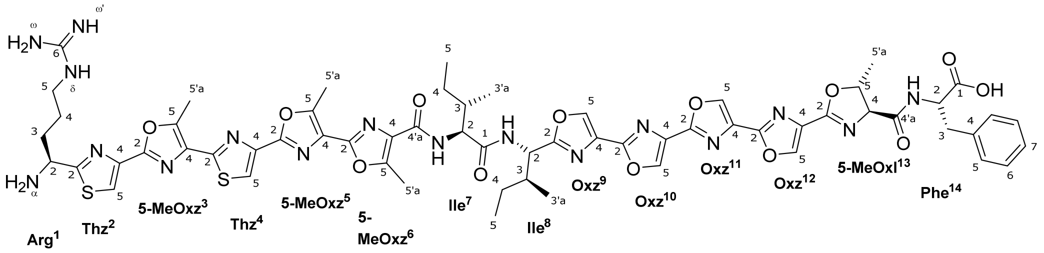

Trifluoroacetic acid (0.5 mL) was added to **38b** (8.8 mg, 5.6 x 10^{-3} mmol) and the reaction mixture was stirred at room temperature for 1 h before the solvent was removed *in vacuo*. Ethanol (4 x 2 mL) then methanol (2 x 2 mL) were added successively, removing the solvent *in vacuo* after each addition. The resulting crude was purified by HPLC to afford the *title compound* **1b** (4.3 mg, 3.3 x 10^{-3} mmol, 64%) as a colourless amorphous solid.

R_t = 5.412 minutes; $[a]_D^{24.7}$ = +0.8 (c = 0.4, 80% MeCN in H_2O); **IR** (neat)/cm^{-1} 3341, 2924, 2853, 1645, 1510, 1467, 1454, 1389, 1101, 979, 719; **^{1}H-NMR** (DMSO-d$_6$, 500 MHz) δ = 9.06 (s, 1H, Oxz^{11}-C5H), 8.98 (s, 1H, Oxz^{10}-C5H), 8.95 (s, 1H, Oxz^{9}-C5H), 8.80 (s, 1H, Oxz^{12}-C5H), 8.75 (s, 1H, Ile^{8}-NH), 8.73 (s, 1H, Arg^{1}-NδH), 8.43 (s, 1H, Thz^{4}-C5H), 8.23 (s, 1H, Thz^{2}-C5H), 7.92 (d, J = 9.6 Hz, 1H, Ile^{7}-NH), 7.73 (s, 3H, Arg^{1}-NωH$_2$ and Arg^{1}-Nω′H), 7.17 (s, 2H, Arg^{1}-NαH$_2$), 7.11 (d, J = 7.3 Hz, 1H, Phe^{14}-NH), 7.08 – 7.02 (m, 2H, 2 x Phe^{14}-C6H), 7.02 – 6.98 (m, 1H, Phe^{14}-C7H), 6.98 – 6.90

(m, 2H, 2 x Phe^{14}-C5H), 4.92 (t, J = 7.4 Hz, 1H, Ile^{8}-C2H), 4.62 – 4.50 (m, 1H, 5-MeOxl^{13}-C5H), 4.41 (t, J = 8.9 Hz, 1H, Ile^{7}-C2H), 4.21 – 4.13 (m, 3H, Arg^{1}-C2H, Phe^{14}-C2H and 5-MeOxl^{13}-C4H), 3.19 – 3.06 (m, 2H, Arg^{1}-C5H_2), 3.02 (dd, J = 13.2, 5.6 Hz, 1H, Phe^{14}-C3H*H*), 2.90 (dd, J = 13.2, 5.6 Hz, 1H, Phe^{14}-C3*H*H), 2.75 (s, 3H, 5-MeOxz^{3}-C5′aH_3), 2.72 (s, 3H, 5-MeOxz^{5}-C5′aH_3), 2.61 (s, 3H, 5-MeOxz^{6}-C5′aH_3), 2.09 – 1.97 (m, 4H, Arg^{1}-C3H_2, Ile^{7}-C3H and Ile^{8}-C3H), 1.67 – 1.60 (m, 1H, Arg^{1}-C4H*H*), 1.58 – 1.50 (m, 1H, Ile^{A}-C4H*H*), 1.47 – 1.38 (m, 5H, 5-MeOxl^{13}-C5′aH_3, Ile^{B}-C4H*H* and Arg^{1}-C4H*H*), 1.31 – 1.16 (m, 1H, Ile^{A}-C4H*H*), 1.05 (dt, J = 12.8, 6.4 Hz, 1H, Ile^{B}-C4H*H*), 0.89 – 0.75 (m, 12H, Ile^{7}-C3′aH_3, Ile^{7}-C5H_3, Ile^{8}-C3′aH_3, Ile^{8}-C5H_3); **^{13}C-NMR** ($CDCl_3$, 126 MHz) δ = 181.9 (quat., Thz^{2}-C2), 174.1 (quat., Phe^{14}-C1), 170.9 (quat., Ile^{7}-C1), 168.8 (quat., 5-MeOxl^{13}-C4′a), 164.3 (quat., Oxz^{9}-C2), 161.5 (quat., Thz^{4}-C2), 160.3 (quat., 5-MeOxz^{6}-C4′a), 157.2 (quat., 5-MeOxz^{6}-C2), 157.1 (quat., 5-MeOxl^{13}-C2), 155.4 (quat., Oxz^{10}-C2), 155.3 (quat., 5-MeOxz^{3}-C2), 155.2 (quat., Oxz^{11}-C2), 155.0 (quat., 5-MeOxz^{5}-C2), 154.8 (quat., Oxz^{12}-C2), 152.4 (quat., 5-MeOxz^{6}-C5), 150.5 (quat., 5-MeOxz^{5}-C5), 147.2 (quat., 5-MeOxz^{3}-C5), 142.7 (quat., Thz^{4}-C4), 142.6 (quat., Thz^{2}-C4), 141.4 (CH, Oxz^{12}-C5), 140.8 (CH, Oxz^{9}-C5), 140.7 (CH, Oxz^{11}-C5), 140.6 (CH, Oxz^{10}-C5), 138.1 (quat., Phe^{14}-C4), 130.5 (quat., Oxz^{12}-C4), 129.8 (quat., Oxz^{11}-C4), 129.7 (quat., Oxz^{10}-C4), 129.5 (CH, Phe^{14}-C5), 129.2 (quat., 5-MeOxz^{3}-C4), 129.1 (quat., Arg^{1}-C6), 129.0 (quat., 5-MeOxz^{6}-C4), 128.6 (quat., Oxz^{9}-C4), 127.3 (CH, Phe^{14}-C6), 125.5 (CH, Phe^{14}-C7), 124.9 (quat., 5-MeOxz^{5}-C4), 121.53 (quat., Thz^{2}-C5), 121.46 (CH, Thz^{4}-C5), 79.5 (CH, 5-MeOxl^{13}-C5), 74.4 (CH, 5-MeOxl^{13}-C4), 57.0 (CH, Ile^{7}-C2), 54.5 (CH, Phe^{14}-C2), 53.0 (CH, Arg^{1}-C2), 51.4 (CH, Ile^{8}-C2), 40.3 (CH_2, Arg^{1}-C5), 37.4 (CH_2, Phe^{14}-C3), 36.8 (CH, Ile^{8}-C3), 36.7 (CH, Ile^{7}-C3), 26.4 (CH_2, Arg^{1}-C3), 24.9 (CH_2, Arg^{1}-C4), 24.8 (CH_2, Ile^{8}-C4), 24.3 (CH_2, Ile^{7}-C4), 21.1 (CH_3, 5-MeOxl^{13}-C5′a), 15.2, 15.1 (CH_3, Ile^{7+8}-C3′a), 11.5 (CH_3, 5-MeOxz^{3}-C5′a), 11.4 (CH_3, 5-MeOxz^{5}-C5′a), 11.2 (CH_3, 5-MeOxz^{6}-C5′a), 10.7, 10.6 (CH_3, Ile^{7+8}-C5); **HRMS** (ESI) found: 1308.4482 ($[M+H]^+$ $C_{61}H_{66}N_{17}O_{13}S_2$ requires 1308.4467).

Summary table of NMR data for Plantazolicin B

Residue	Atom	^{1}H NMR (500 MHz, DMSO) δ	^{13}C NMR (500 MHz, DMSO) δ
Arg^{1}	NαH_2	7.17 (s, 2H)	
	C2H	4.21 – 4.13 (m, 1 out of 3H)	53.0 (CH)
	C3H_2	2.09 – 1.97 (m, 2 out of 4H)	26.4 (CH_2)
	C4H_2	1.67 – 1.60 (m, 1H) and 1.47 – 1.38 (m, 1 out of 5H)	24.9 (CH_2)
	C5H_2	3.19 – 3.06 (m, 2H)	40.3 (CH_2)
	C6		129.1 (quat.)
	NδH	8.73 (s, 1H)	
	NωH_2	7.73 (s, 2 out of 3H)	
	Nω′H	7.73 (s, 1 out of 3H)	
Thz^{2}	C2		181.9 (quat.)
	C4		142.6 (quat.)
	C5H	8.23 (s, 1H)	121.53 (quat.)
5-MeOxz^{3}	C2		155.3 (quat.)
	C4		129.2 (quat.)
	C5		147.2 (quat.)
	C5′aH_3	2.75 (s, 3H)	11.5 (CH_3)
Thz^{4}	C2		161.5 (quat.)
	C4		142.7 (quat.)
	C5H	8.43 (s, 1H)	121.46 (CH)
5-MeOxz^{5}	C2		155.0 (quat.)
	C4		124.9 (quat.)
	C5		150.5 (quat.)
	C5′aH_3	2.72 (s, 3H)	11.4 (CH_3)
5-MeOxz^{6}	C2		157.2 (quat.)
	C4		129.0 (quat.)
	C4′a		160.3 (quat.)
	C5		152.4 (quat.)

	C5′aH_3	2.61 (s, 3H)	11.2 (CH_3)
Ile[7]	NH	7.92 (d, *J* = 9.6 Hz, 1H)	
	C1		170.9 (quat.)
	C2H	4.41 (t, *J* = 8.9 Hz, 1H)	57.0 (CH)
	C3H	2.09 – 1.97 (m, 1 out of 4H)	36.7 (CH)
	C3′aH_3	0.89 – 0.75 (m, 3 out of 12H)	15.2 (CH_3) *or* 15.1 (CH_3)
	C4H_2	*Either* 1.58 – 1.50 (m, 1H) and 1.31 – 1.16 (m, 1H) *or* 1.47 – 1.38 (m, 1 out of 5H) and 1.05 (dt, *J* = 12.8, 6.4 Hz, 1H)	24.3 (CH_2)
	C5H_3	0.89 – 0.75 (m, 3 out of 12H)	10.7 (CH_3) *or* 10.6 (CH_3)
Ile[8]	NH	8.75 (s, 1H)	
	C2H	4.92 (t, *J* = 7.4 Hz, 1H)	51.4 (CH)
	C3H	2.09 – 1.97 (m, 1 out of 4H)	36.8 (CH)
	C3′aH_3	0.89 – 0.75 (m, 3 out of 12H)	15.2 *or* 15.1
	C4H_2	*Either* 1.58 – 1.50 (m, 1H) and 1.31 – 1.16 (m, 1H) *or* 1.47 – 1.38 (m, 1 out of 5H) and 1.05 (dt, *J* = 12.8, 6.4 Hz, 1H)	24.8 (CH_2)
	C5H_3	0.89 – 0.75 (m, 3 out of 12H)	10.7 *or* 10.6 (CH_3)
Oxz[9]	C2		164.3 (quat.)
	C4		128.6 (quat.)
	C5H	8.95 (s, 1H)	140.8 (CH)
Oxz[10]	C2		155.4 (quat.)
	C4		129.7 (quat.)
	C5H	8.98 (s, 1H)	140.6 (CH)
Oxz[11]	C2		155.2 (quat.)
	C4		129.8 (quat.)
	C5H	9.06 (s, 1H)	140.7 (CH)
Oxz[12]	C2		154.8 (quat.)
	C4		130.5 (quat.)
	C5H	8.80 (s, 1H)	141.4 (CH)
5-MeOxl[13]	C2		157.1 (quat.)
	C4H	4.21 – 4.13 (m, 1 out of 3H)	74.4 (CH)
	C4′a		168.8 (quat.)
	C5H	4.62 – 4.50 (m, 1H)	79.5 (CH)
	C5′aH_3	1.47 – 1.38 (m, 3 out of 5H)	21.1 (CH_3)
Phe[14]	NH	7.11 (d, *J* = 7.3 Hz, 1H)	
	C1		174.1 (quat.)
	C2H	4.21 – 4.13 (m, 1 out of 3H)	54.5 (CH)
	C3H_2	3.02 (dd, *J* = 13.2, 5.6 Hz, 1H) and 2.90 (dd, *J* = 13.2, 5.6 Hz, 1H)	37.4 (CH_2)
	C4		138.1 (quat.)
	C5H (2)	6.98 – 6.90 (m, 2H)	129.5 (CH)
	C6H (2)	7.08 – 7.02 (m, 2H)	127.3 (CH)
	C7H	7.02 – 6.98 (m, 1H)	125.5 (CH)

13. References

[1] M. Piotto, M. Bourdonneau, K. Elbayed, J. M. Wieruszeski, G. Lippens, *Magn Reson Chem* 2006, *44*, 943-947.

[2] M. Sawamura, Y. Nakayama, T. Kato, Y. Ito, *J Org Chem* 1995, *60*, 1727-1732.

[3] K. C. Nicolaou, B. S. Safina, M. Zak, S. H. Lee, M. Nevalainen, M. Bella, A. A. Estrada, C. Funke, F. J. Zécri, S. Bulat, *Journal of the American Chemical Society* 2005, *127*, 11159-11175.

[4] Deoxo-Fluor is a registered trade mark of Air Products and Chemicals, Inc. and was obtained from Sigma Aldrich

[5] S. Pena, L. Scarone, E. Manta, L. Stewart, V. Yardley, S. Croft, G. Serra, *Bioorg Med Chem Lett* 2012, *22*, 4994-4997.

[6] S. Yoshimura, Y. Shimonishi, *B Chem Soc Jpn* 1985, *58*, 2805-2811.

[7] S. Banala, P. Ensle, R. D. Sussmuth, *Angew Chem Int Edit* 2013, *52*, 9518-9523.

[8] B. Kalyon, S. E. Helaly, R. Scholz, J. Nachtigall, J. Vater, R. Borriss, R. D. Sussmuth, *Org Lett* 2011, *13*, 2996-2999.

14. NMRs of Synthesised Compounds

$CDCl_3$, 600 MHz

23

^1H NMR

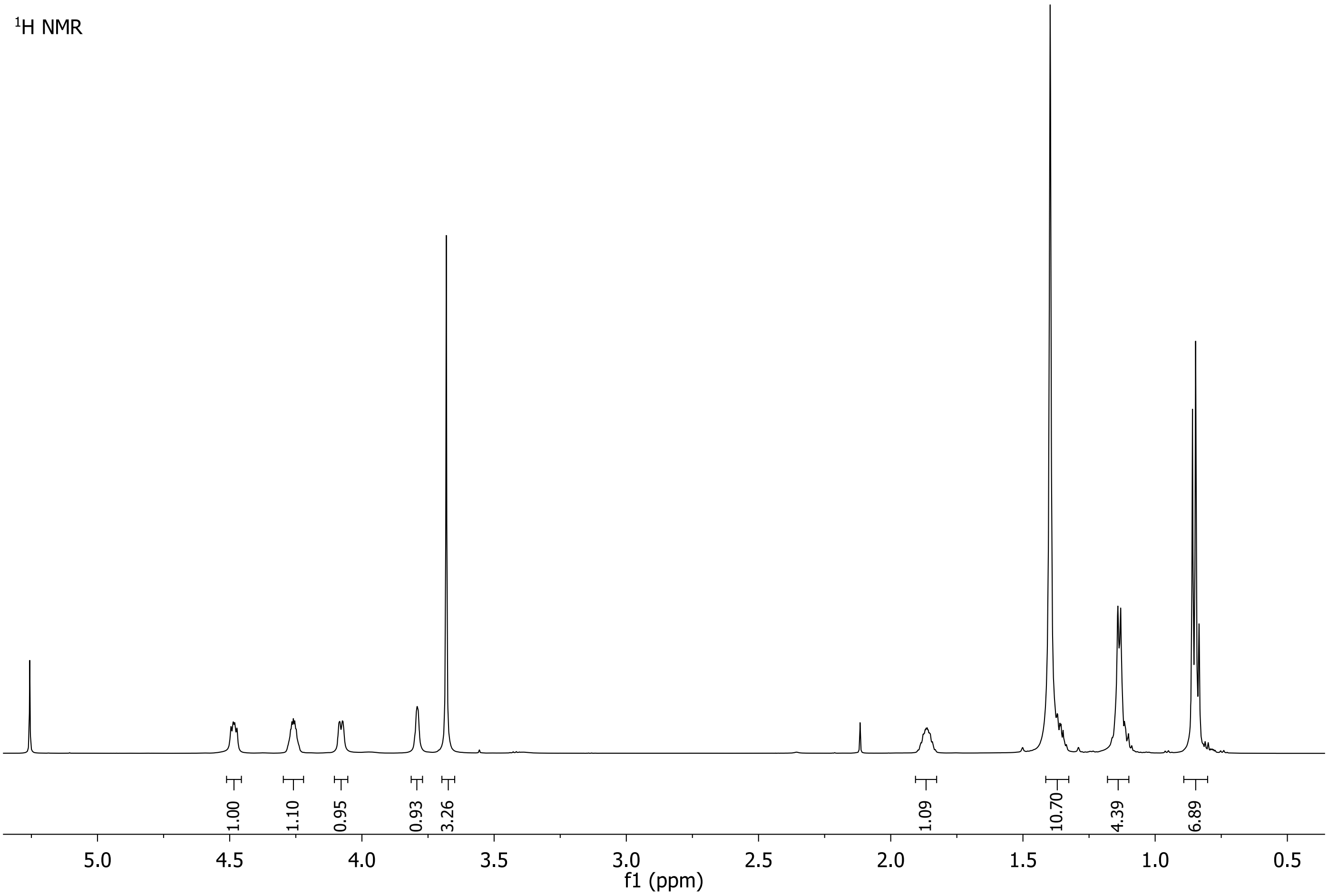

$CDCl_3$, 600 MHz

23

^{13}C NMR

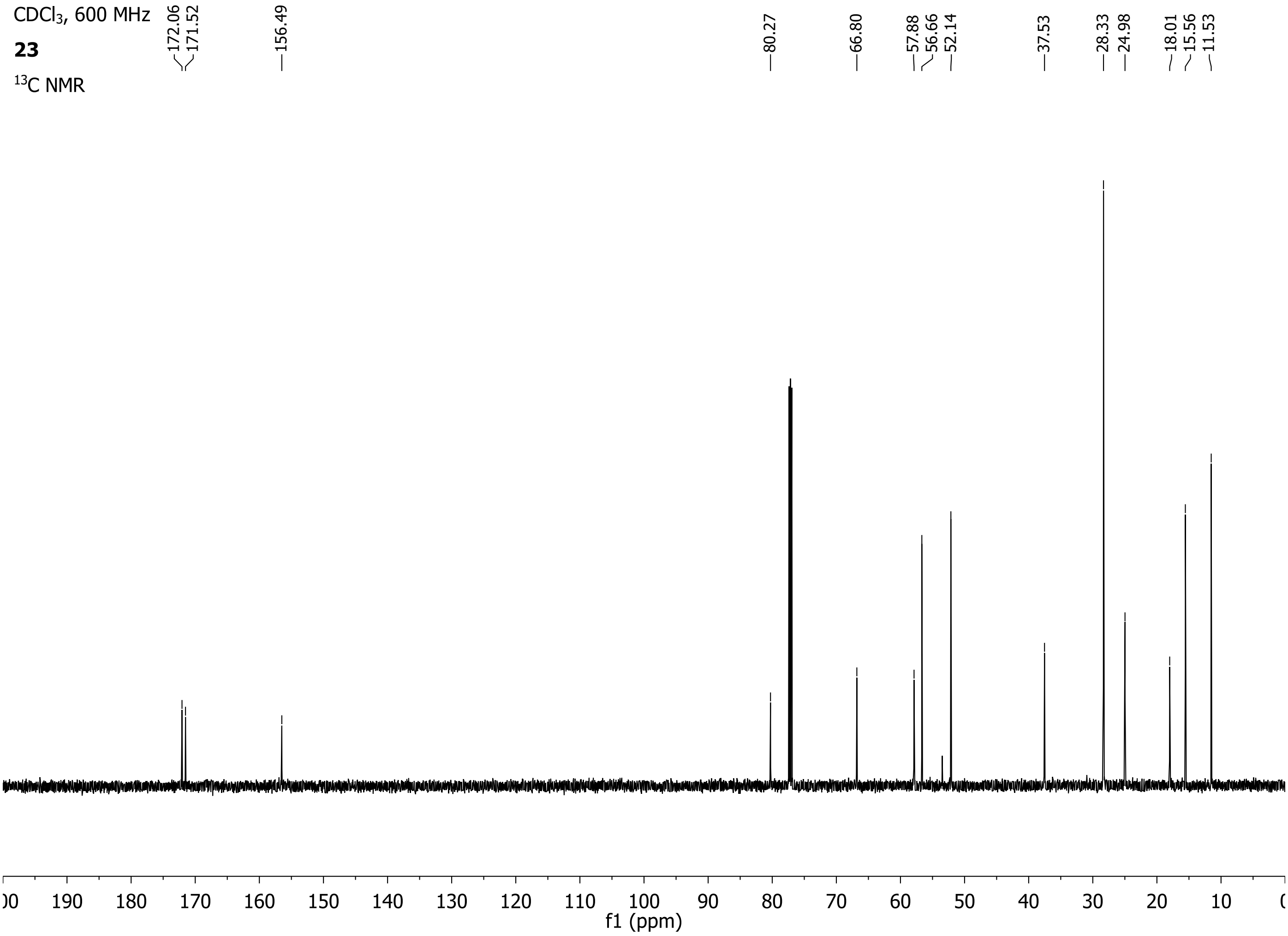

CDCl$_3$, 600 MHz

23

DEPT-135

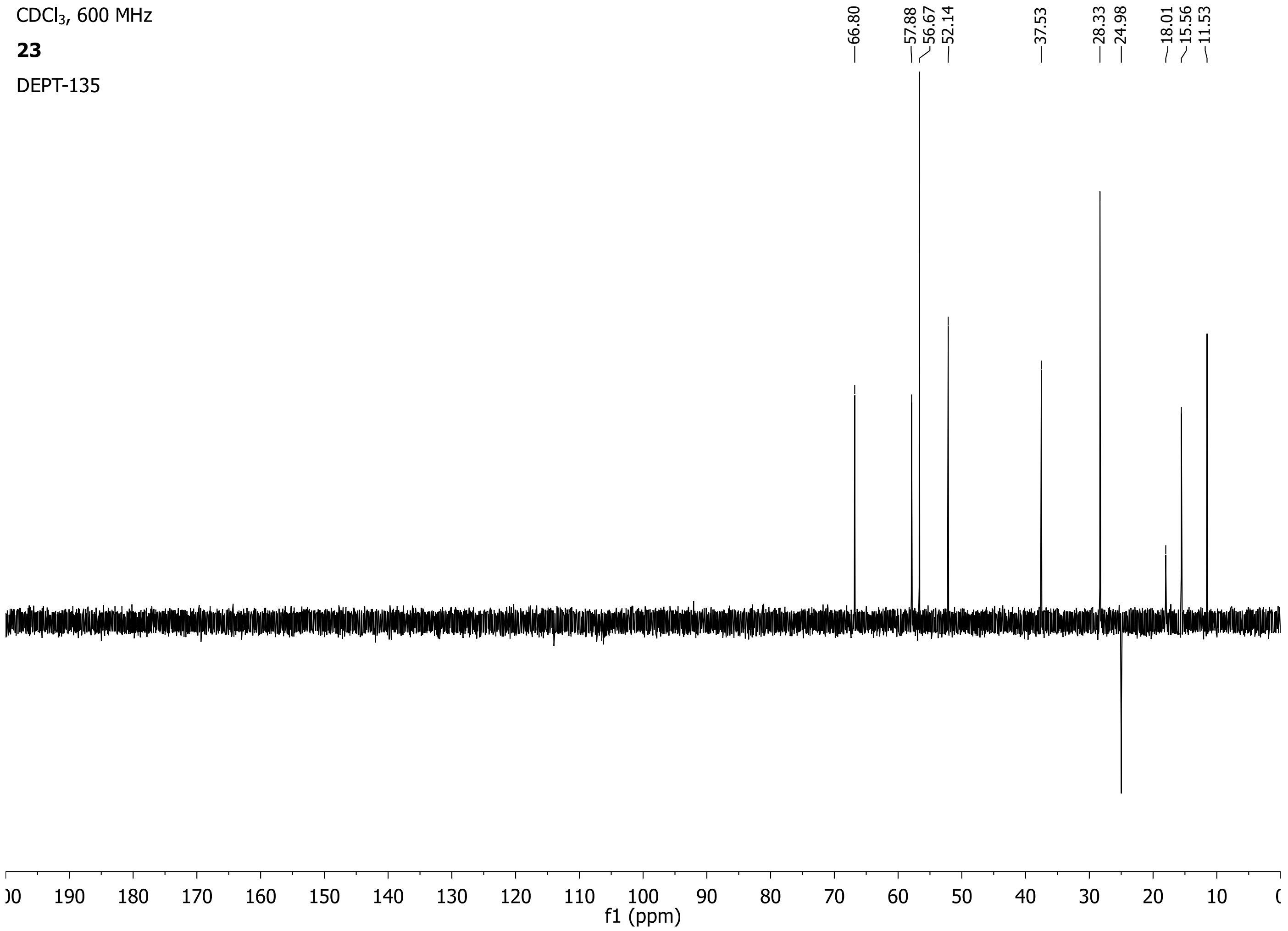

CDCl$_3$, 600 MHz

23

COSY

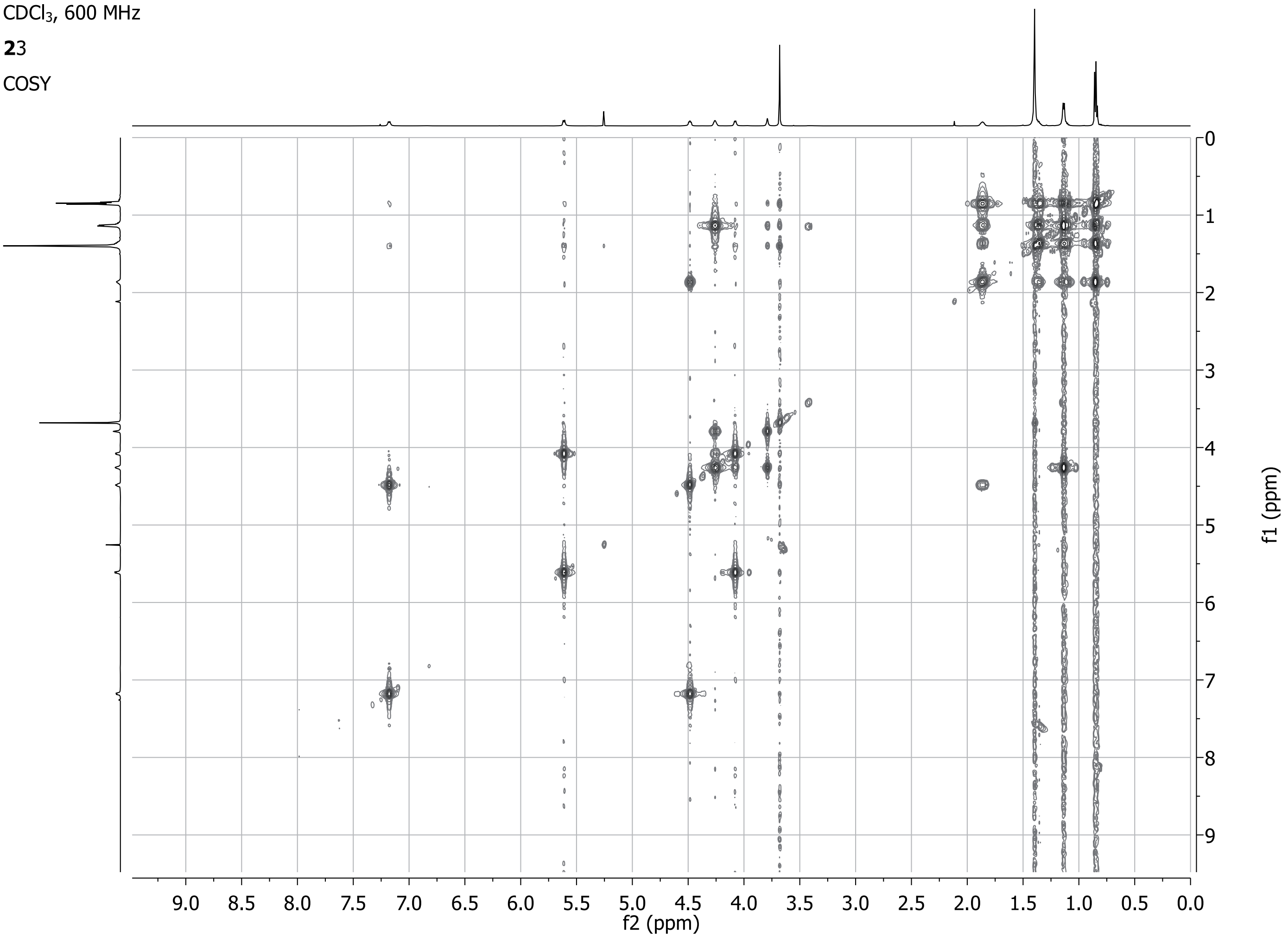

CDCl$_3$, 600 MHz

23

HMQC

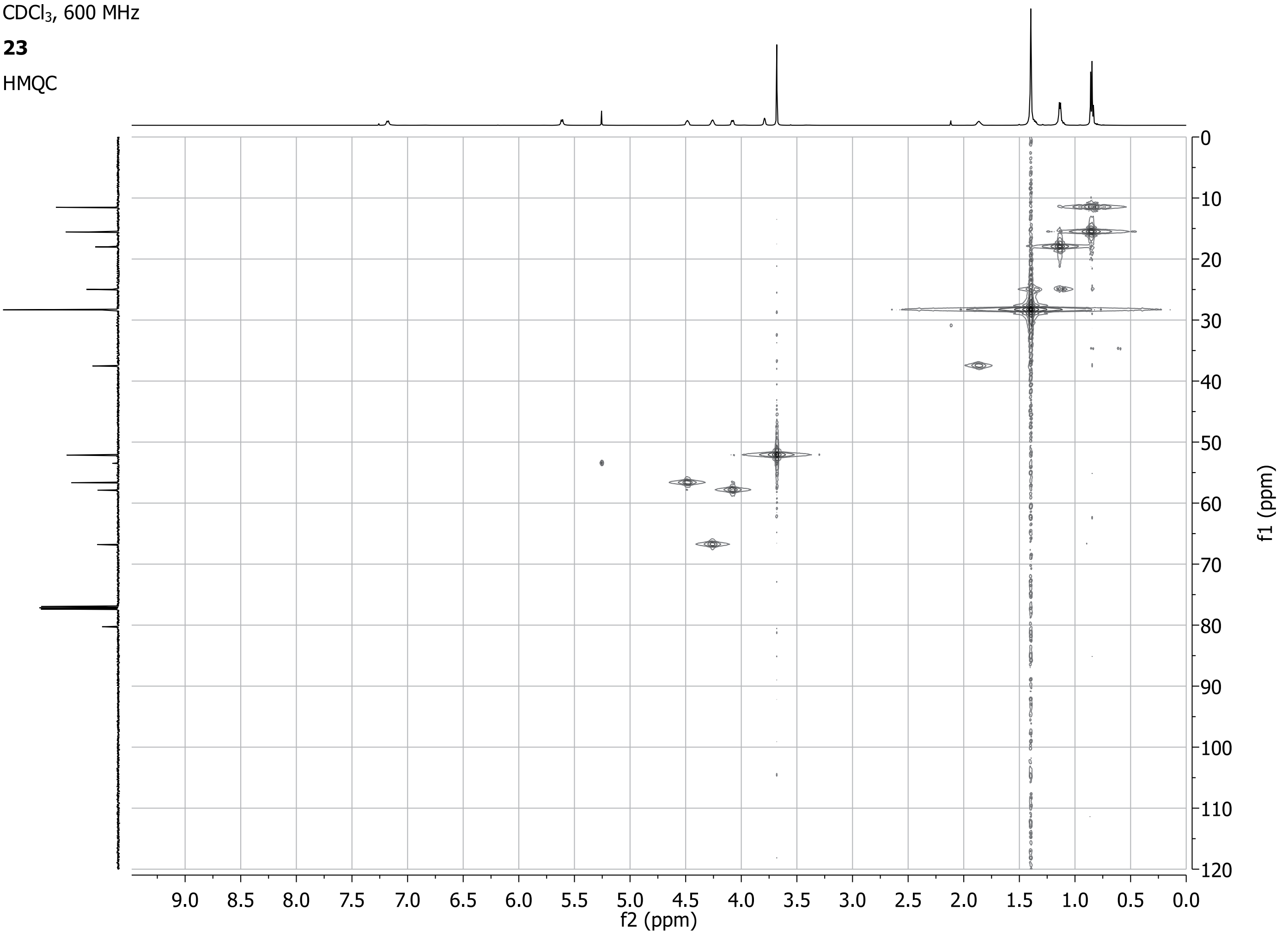

CDCl$_3$, 600 MHz

23

HMBC

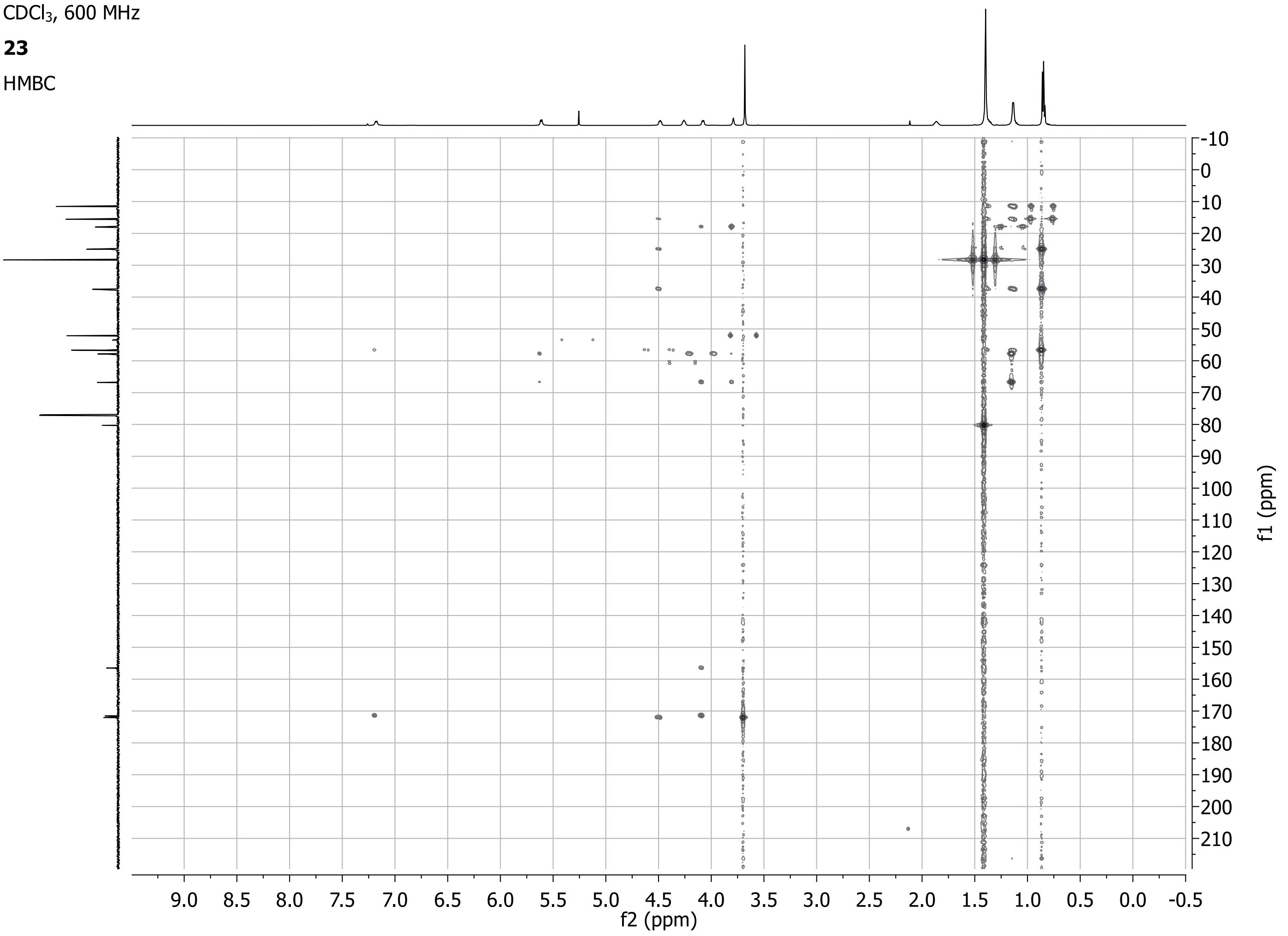

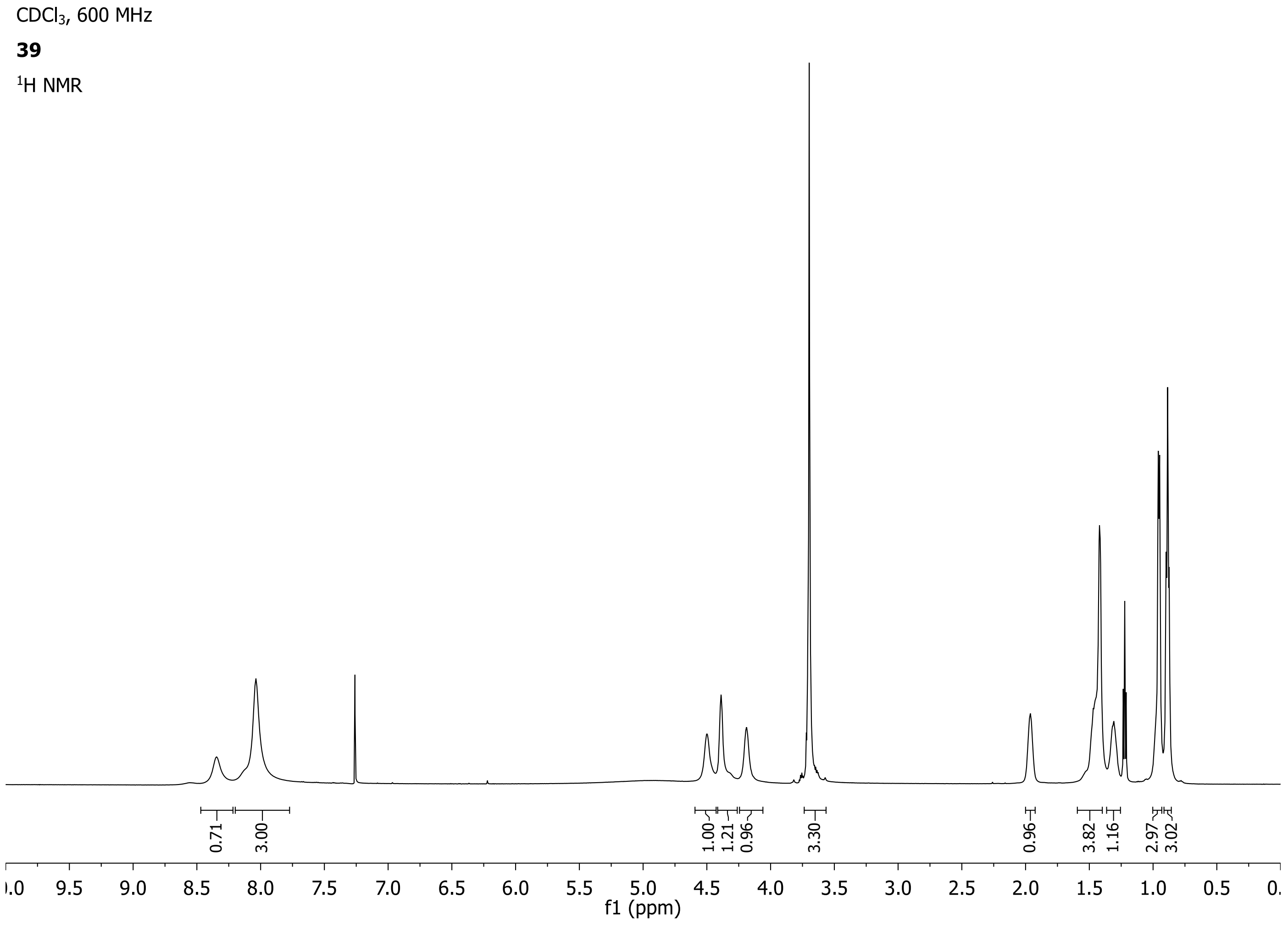
CDCl$_3$, 600 MHz
39
^{1}H NMR
0.71
3.00
1.00
1.21
0.96
3.30
0.96
3.82
1.16
2.97
3.02
9.5 9.0 8.5 8.0 7.5 7.0 6.5 6.0 5.5 5.0 4.5 4.0 3.5 3.0 2.5 2.0 1.5 1.0 0.5
f1 (ppm)

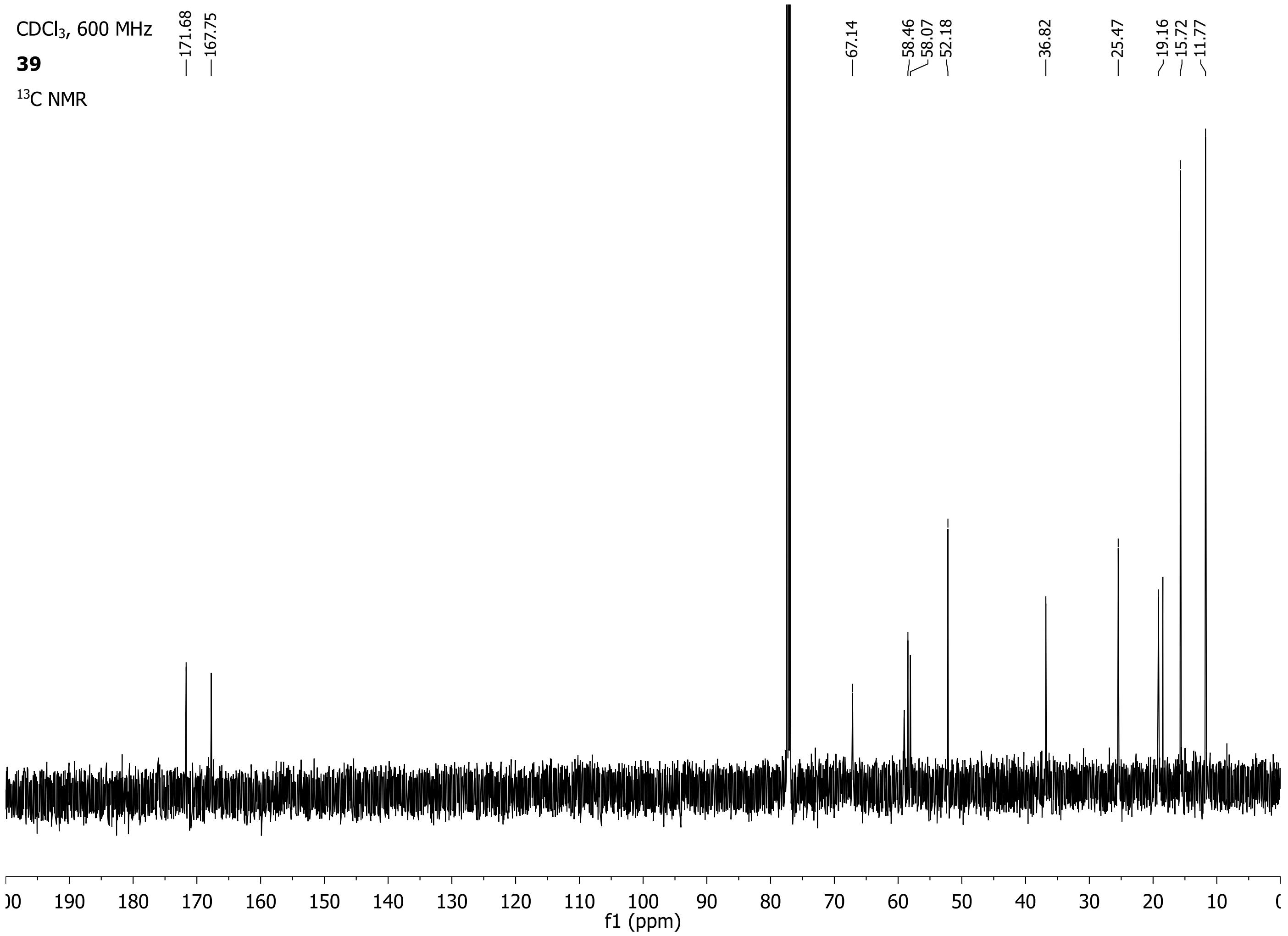
CDCl$_3$, 600 MHz
39
^{13}C NMR
171.68
167.75
67.14
58.46
58.07
52.18
36.82
25.47
19.16
15.72
11.77
190 180 170 160 150 140 130 120 110 100 90 80 70 60 50 40 30 20 10
f1 (ppm)

CDCl$_3$, 600 MHz

39

DEPT-135

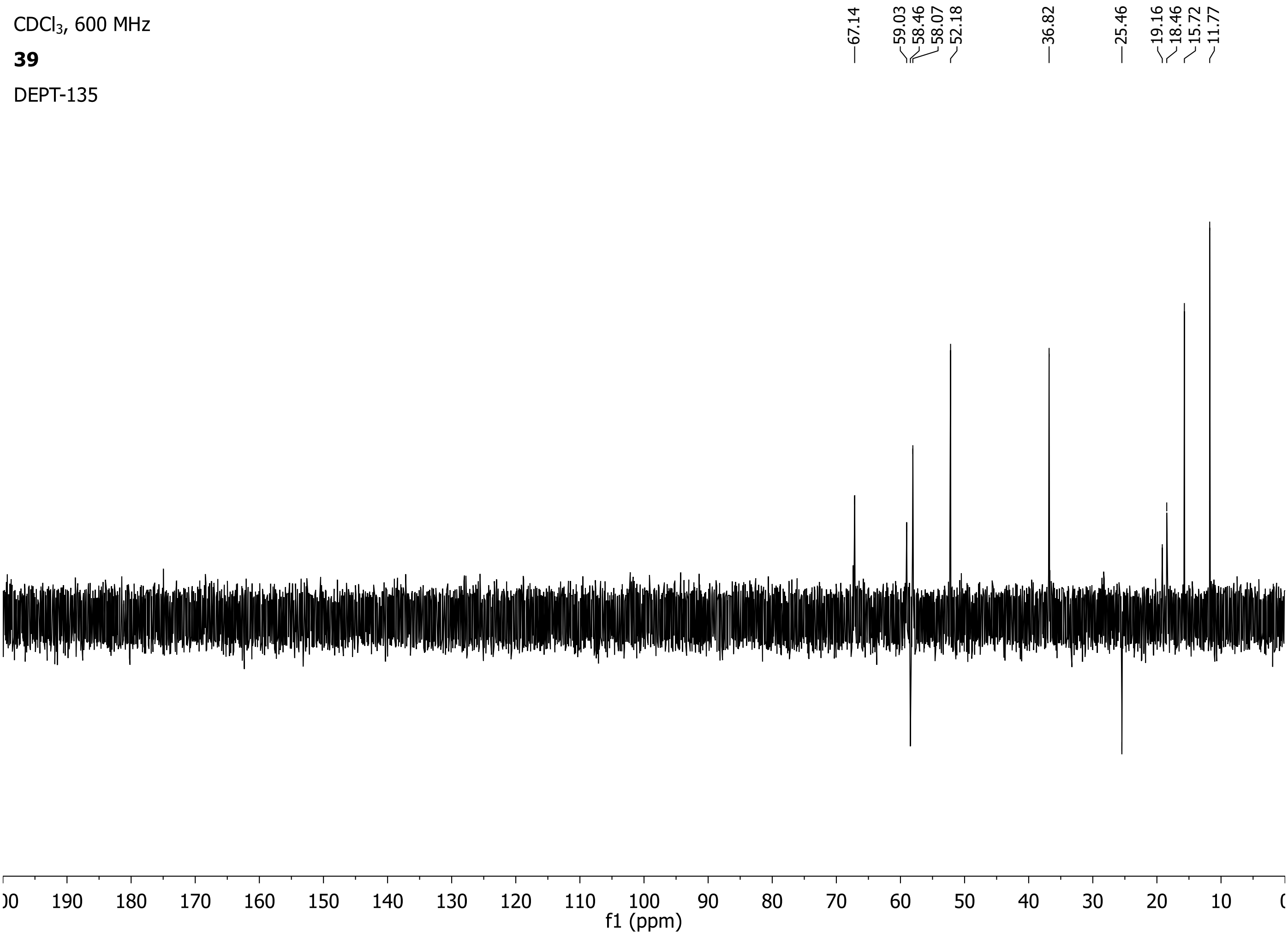

CDCl$_3$, 600 MHz

39

COSY

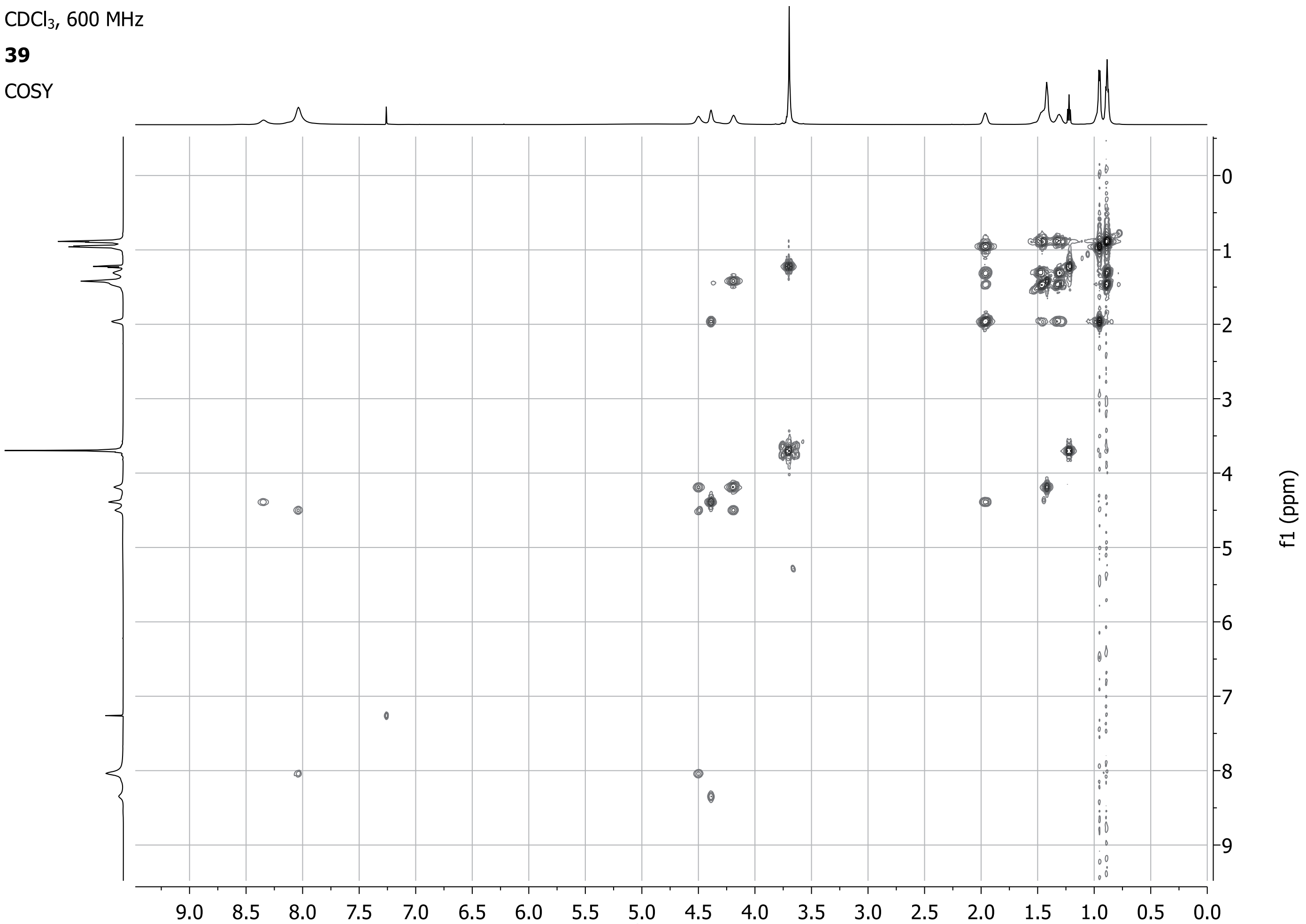

$CDCl_3$, 600 MHz

39

HMQC

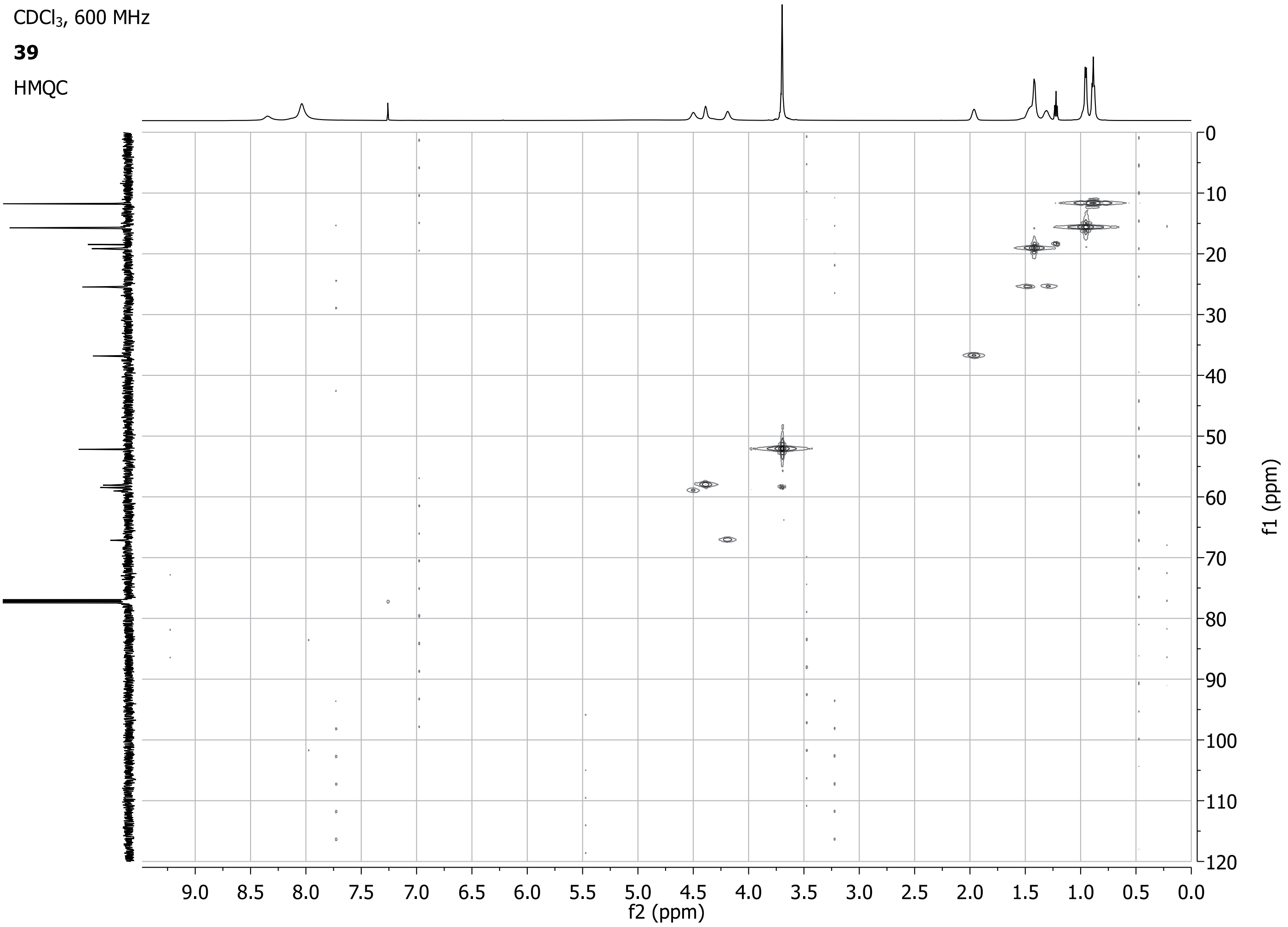

$CDCl_3$, 600 MHz

39

HMBC

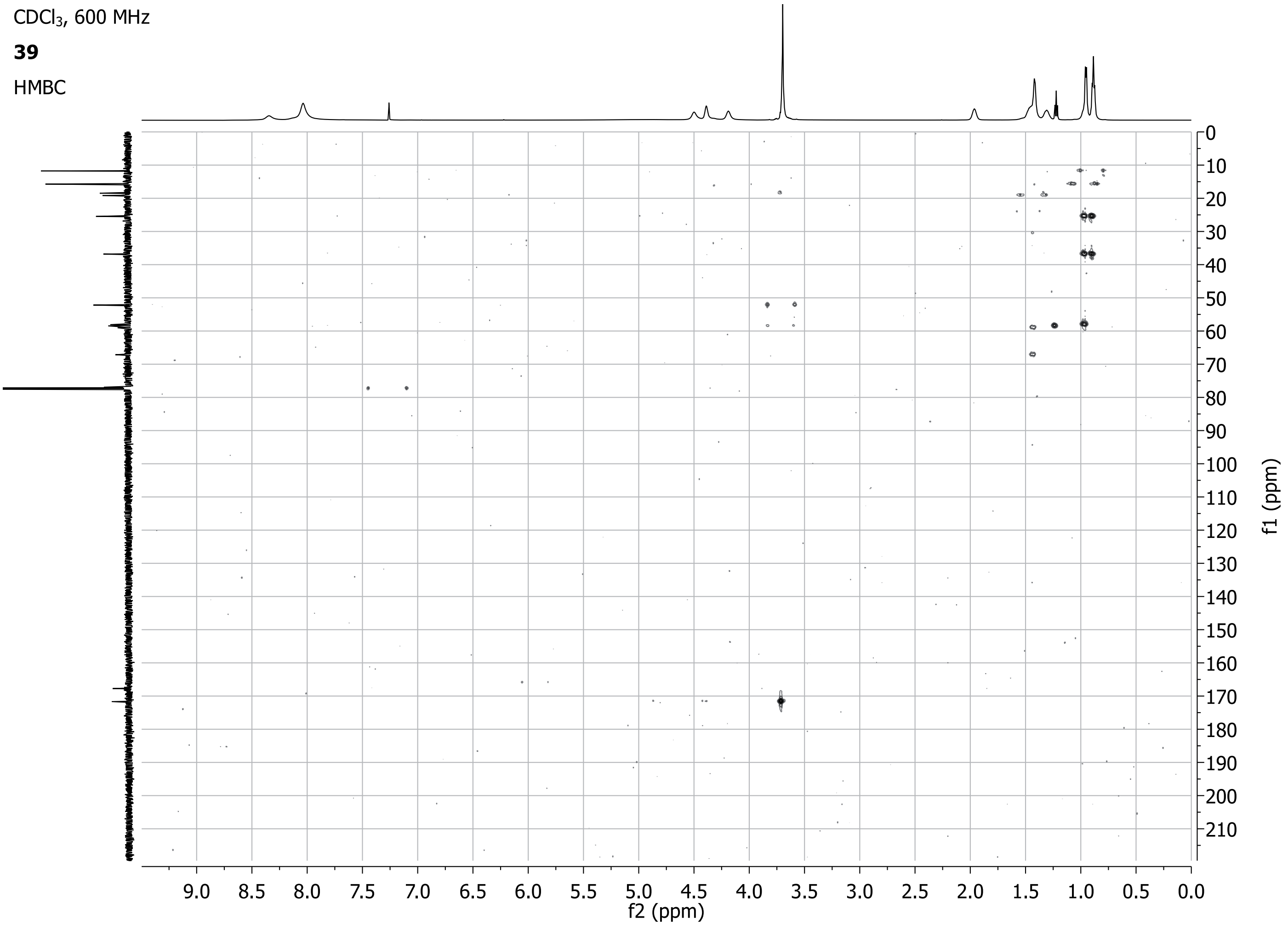

CDCl$_3$, 600 MHz

9

^{1}H NMR

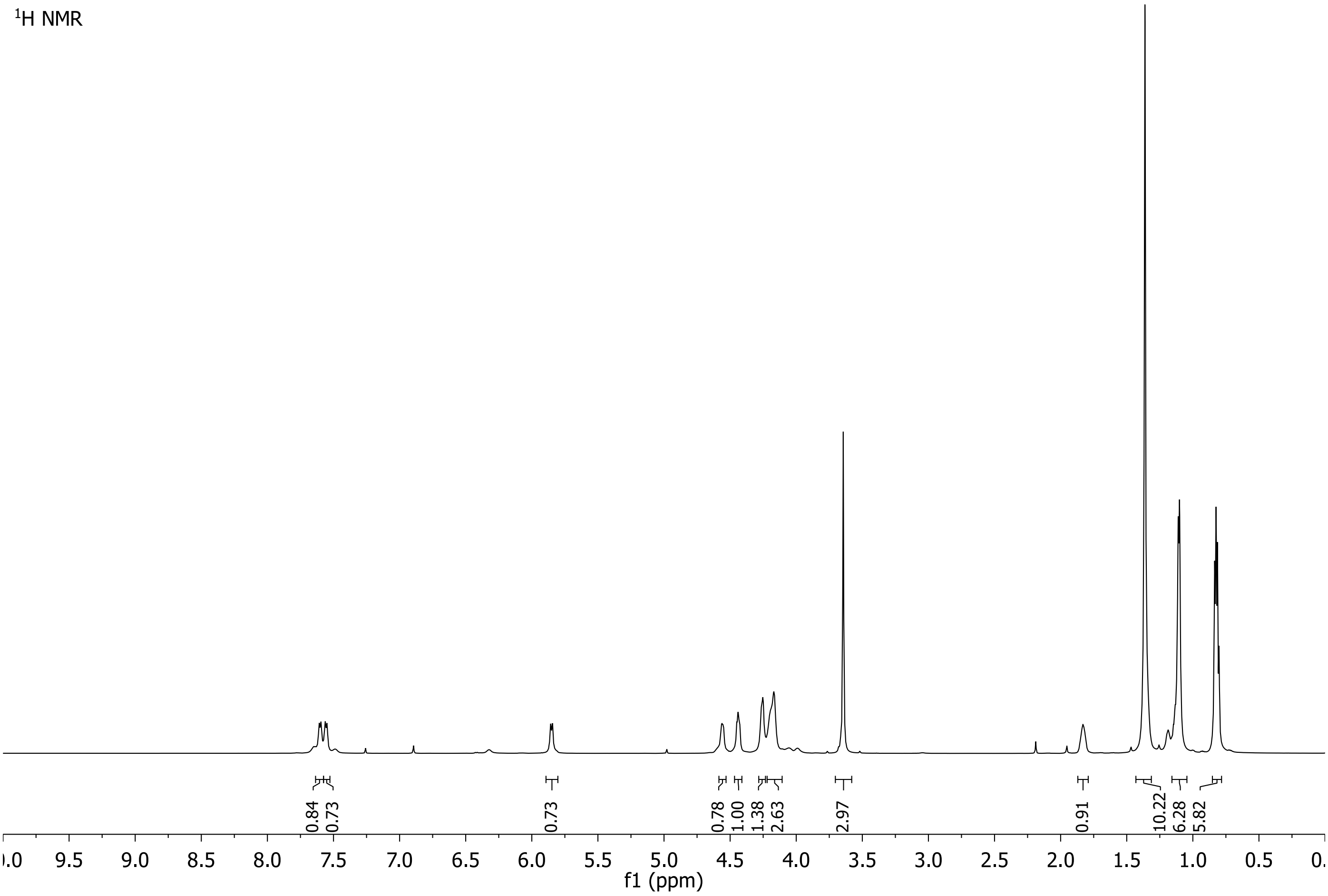

CDCl$_3$, 600 MHz

9

^{13}C NMR

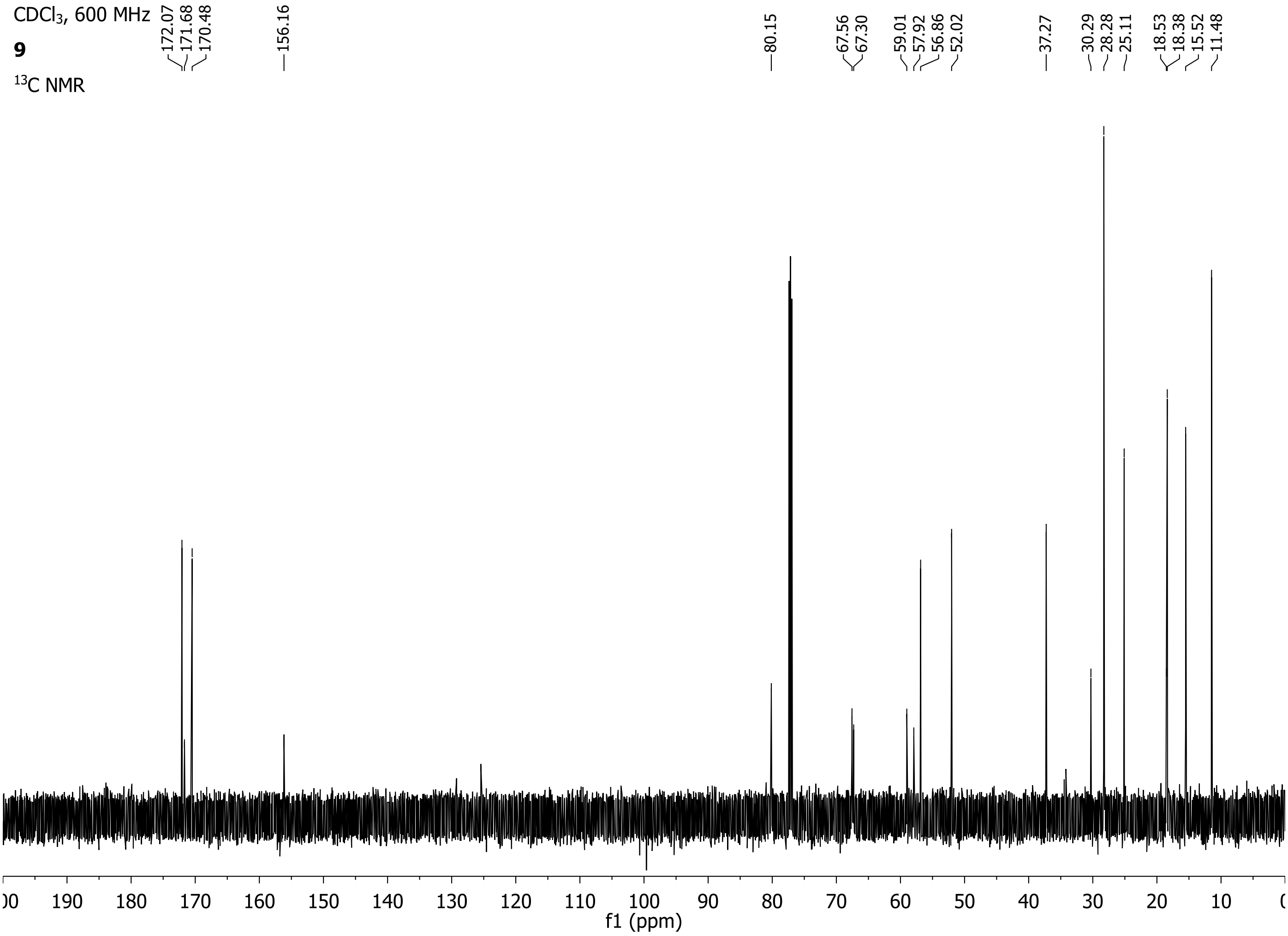

CDCl$_3$, 600 MHz

9

DEPT-135

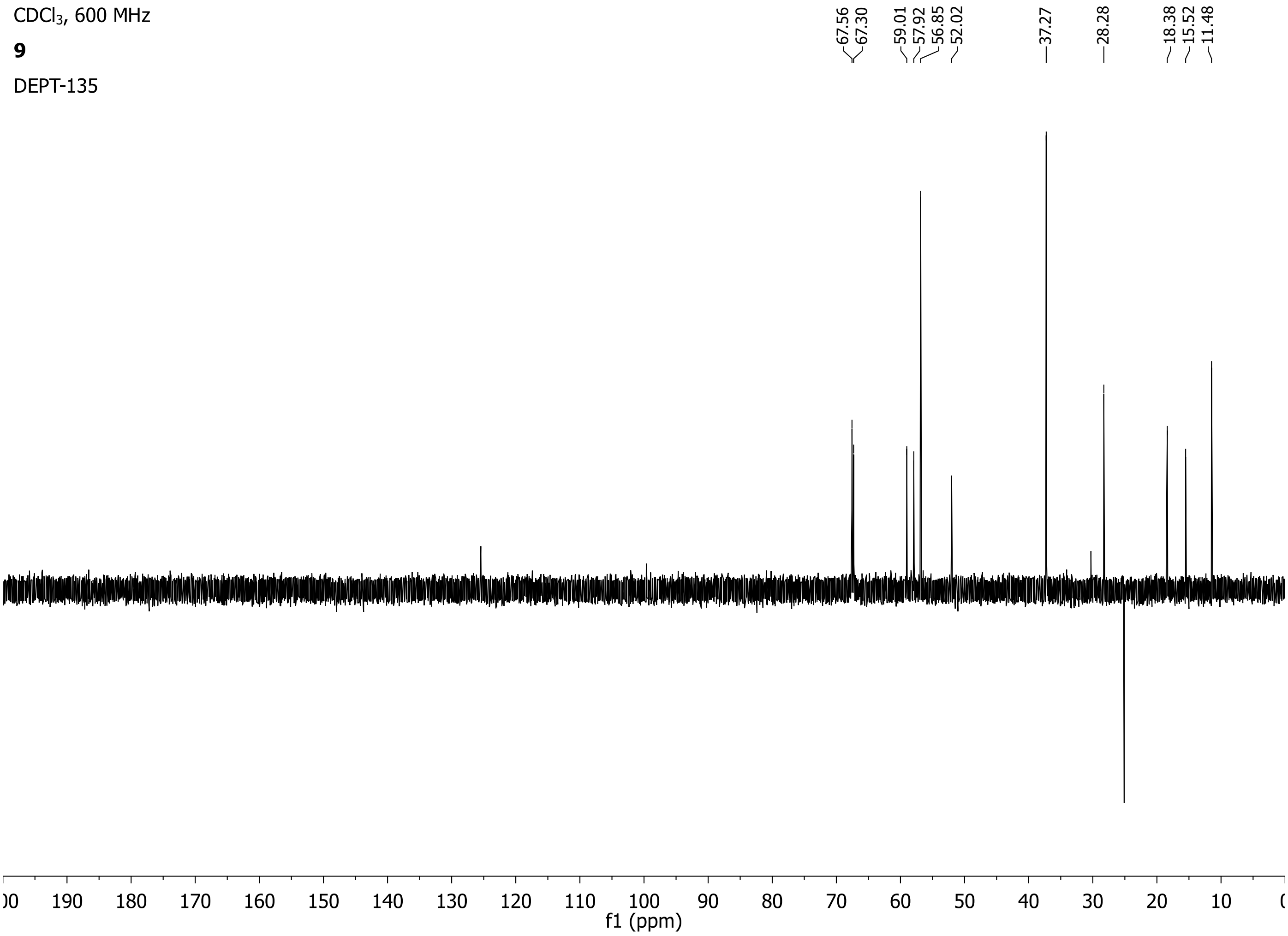

CDCl$_3$, 600 MHz

9

COSY

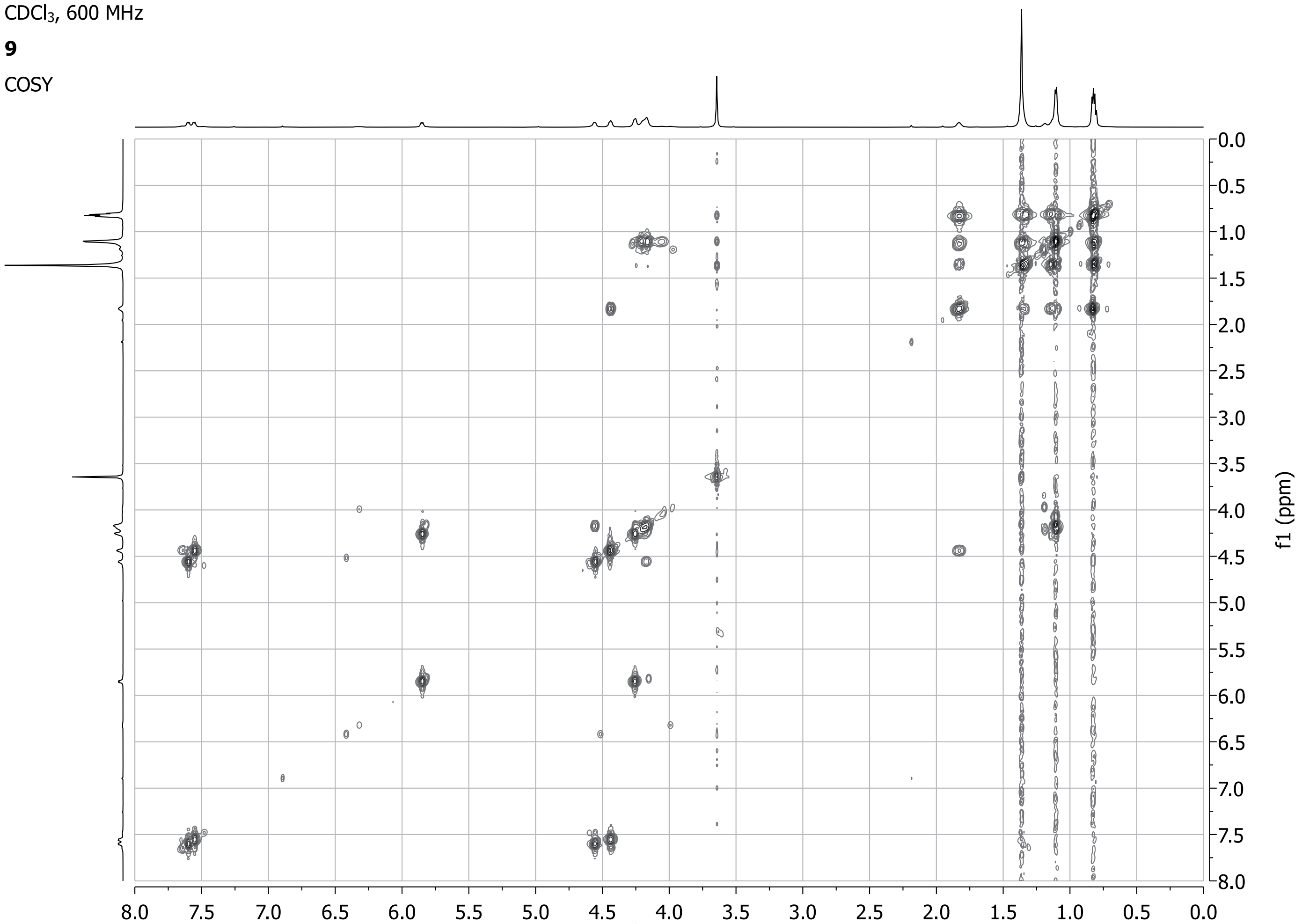

CDCl$_3$, 600 MHz

9

HMQC

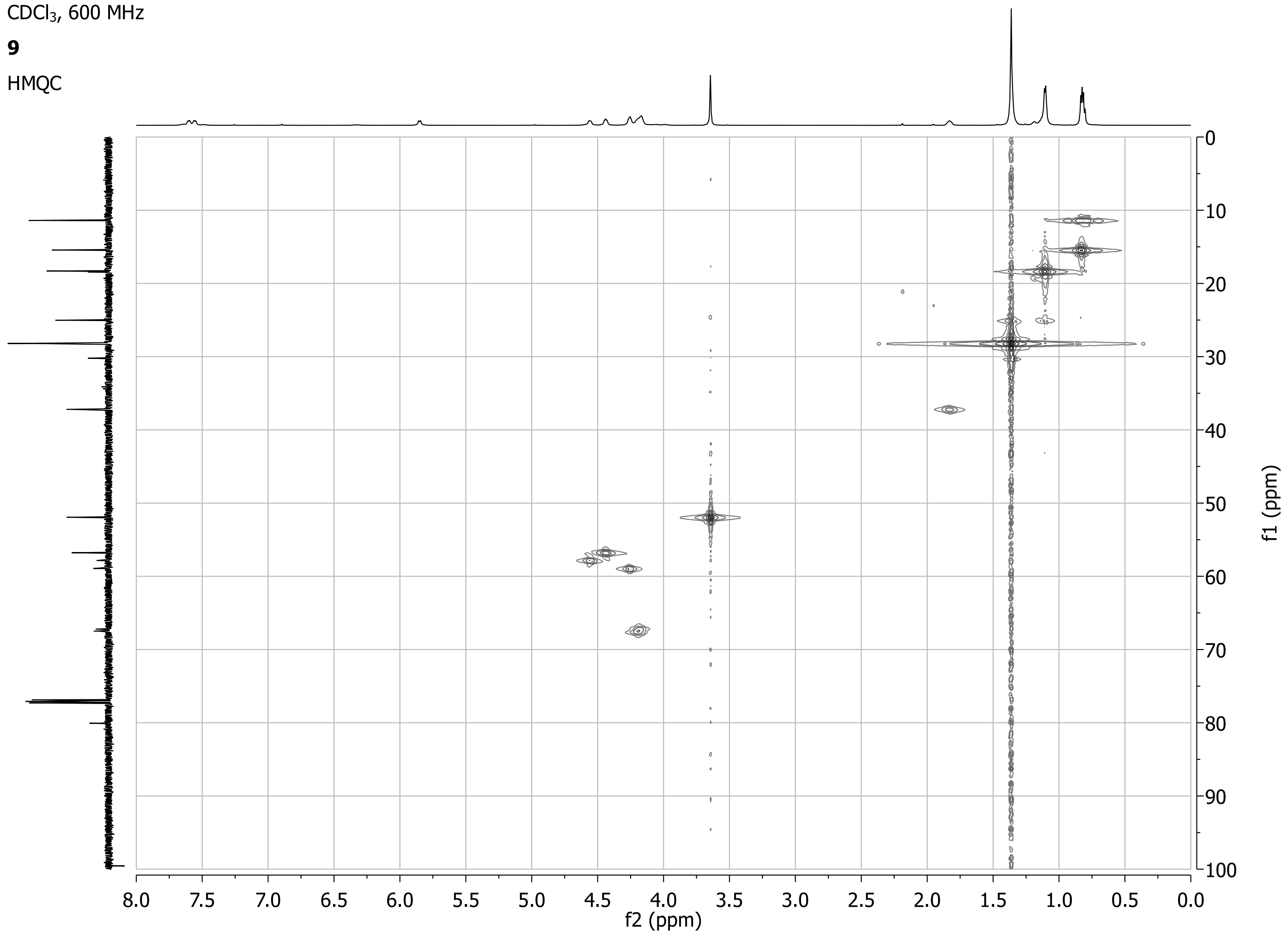

CDCl$_3$, 600 MHz

9

HMBC

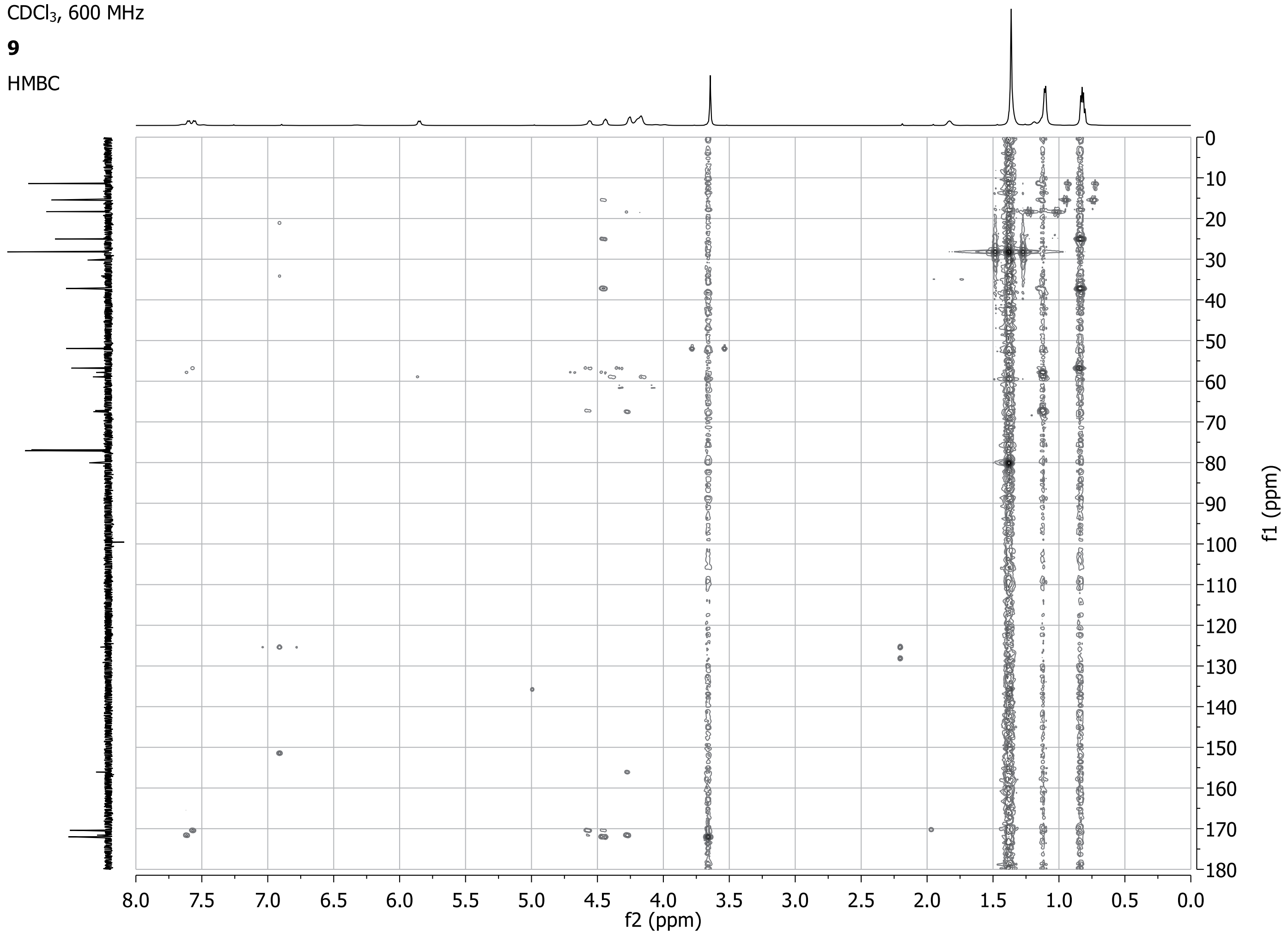

CDCl$_3$, 600 MHz

40

^{1}H NMR

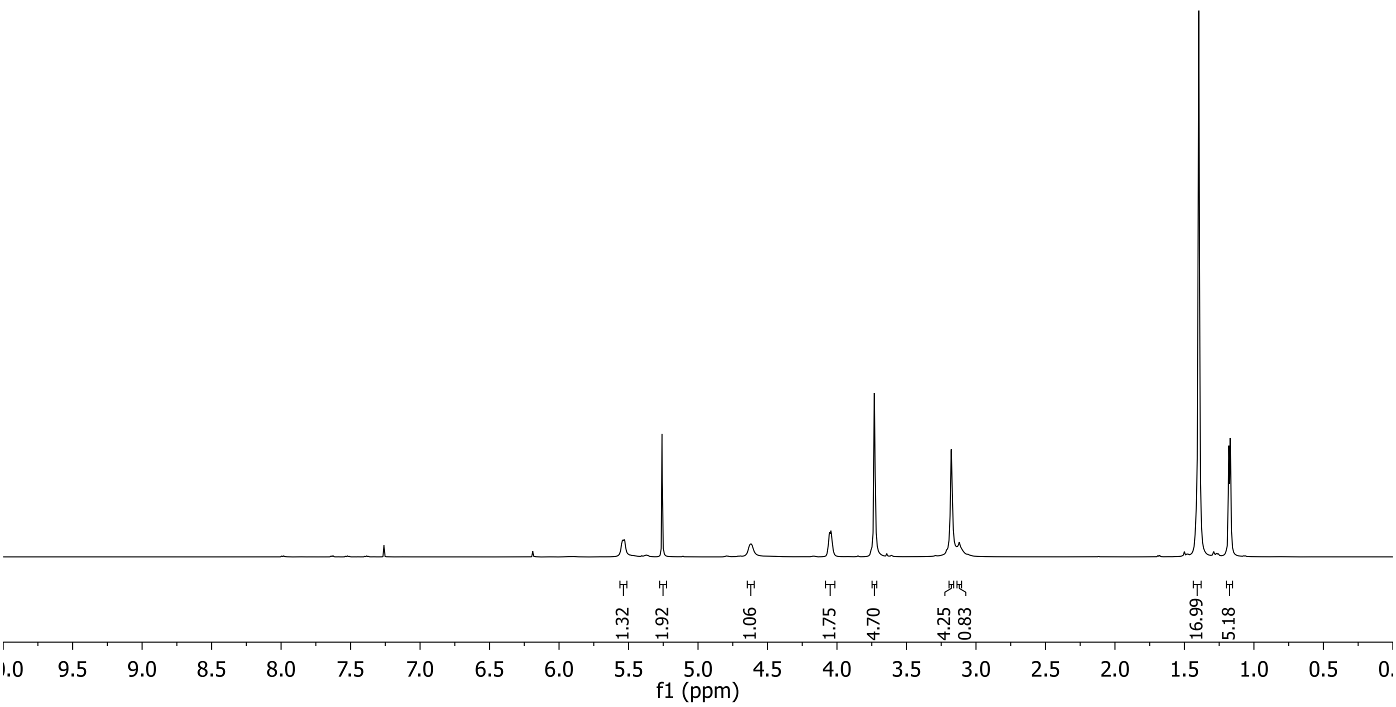

CDCl$_3$, 600 MHz

40

^{13}C NMR

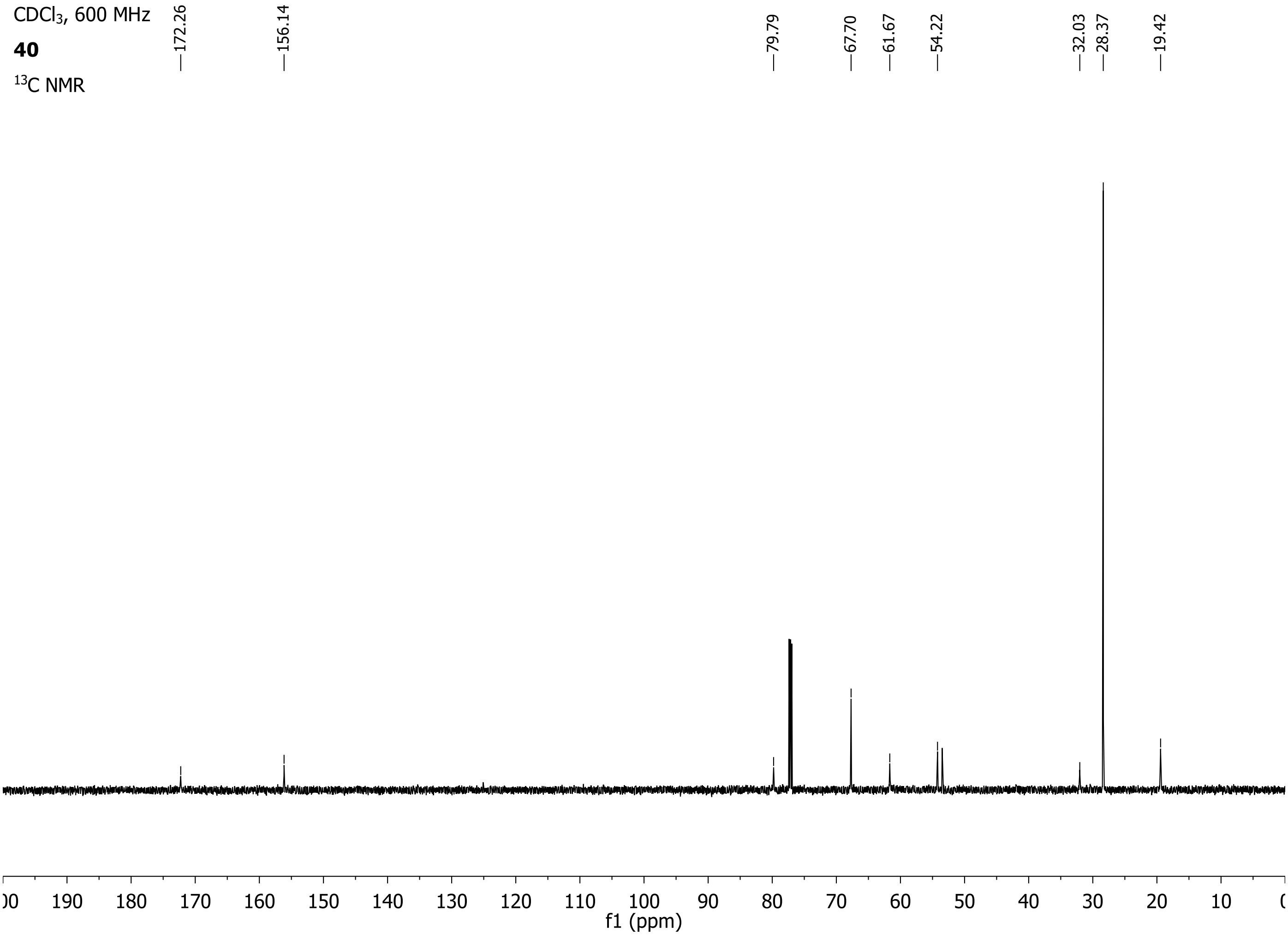

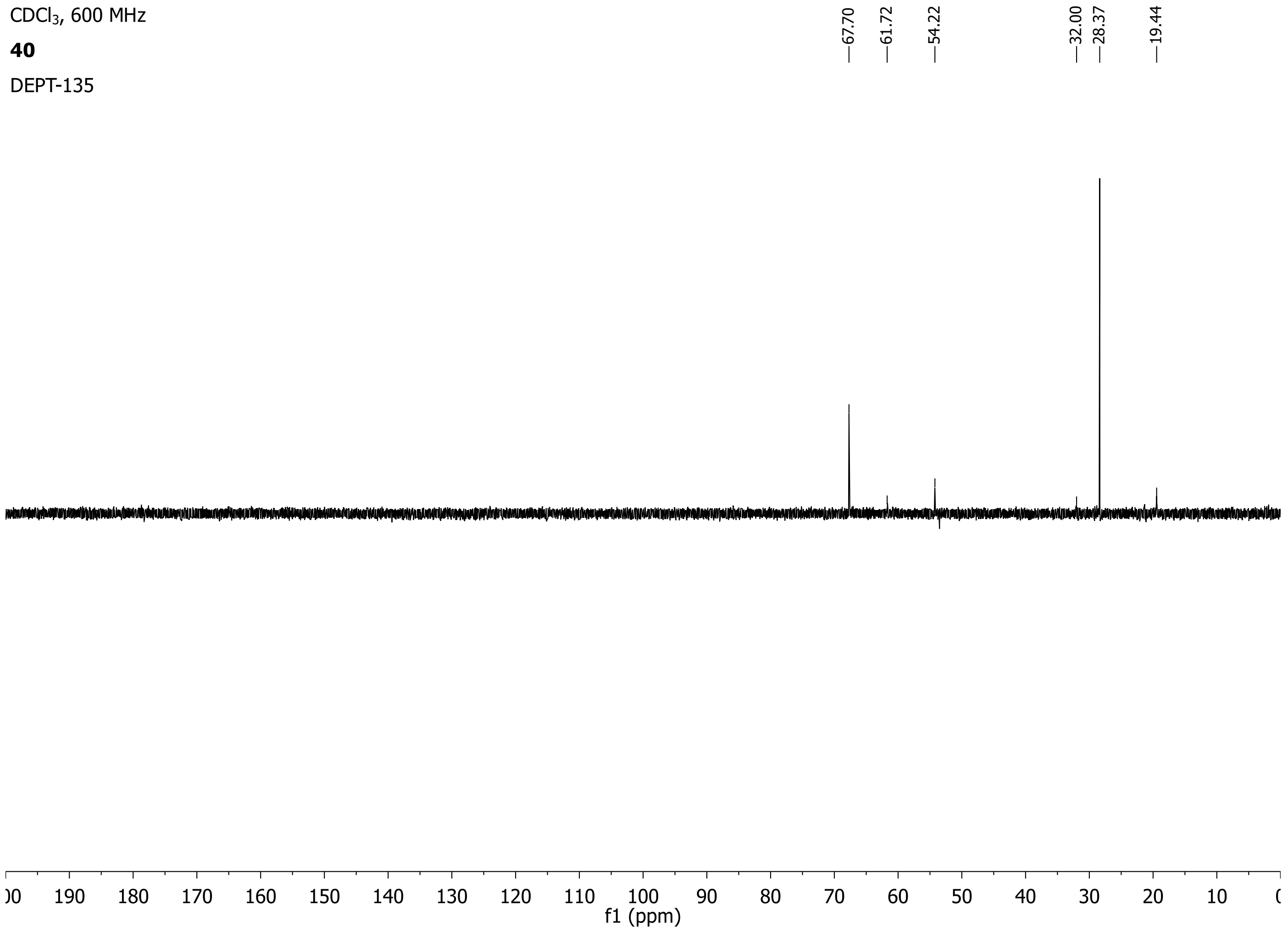
CDCl3, 600 MHz
40
DEPT-135
67.70
61.72
54.22
32.00
28.37
19.44
f1 (ppm)

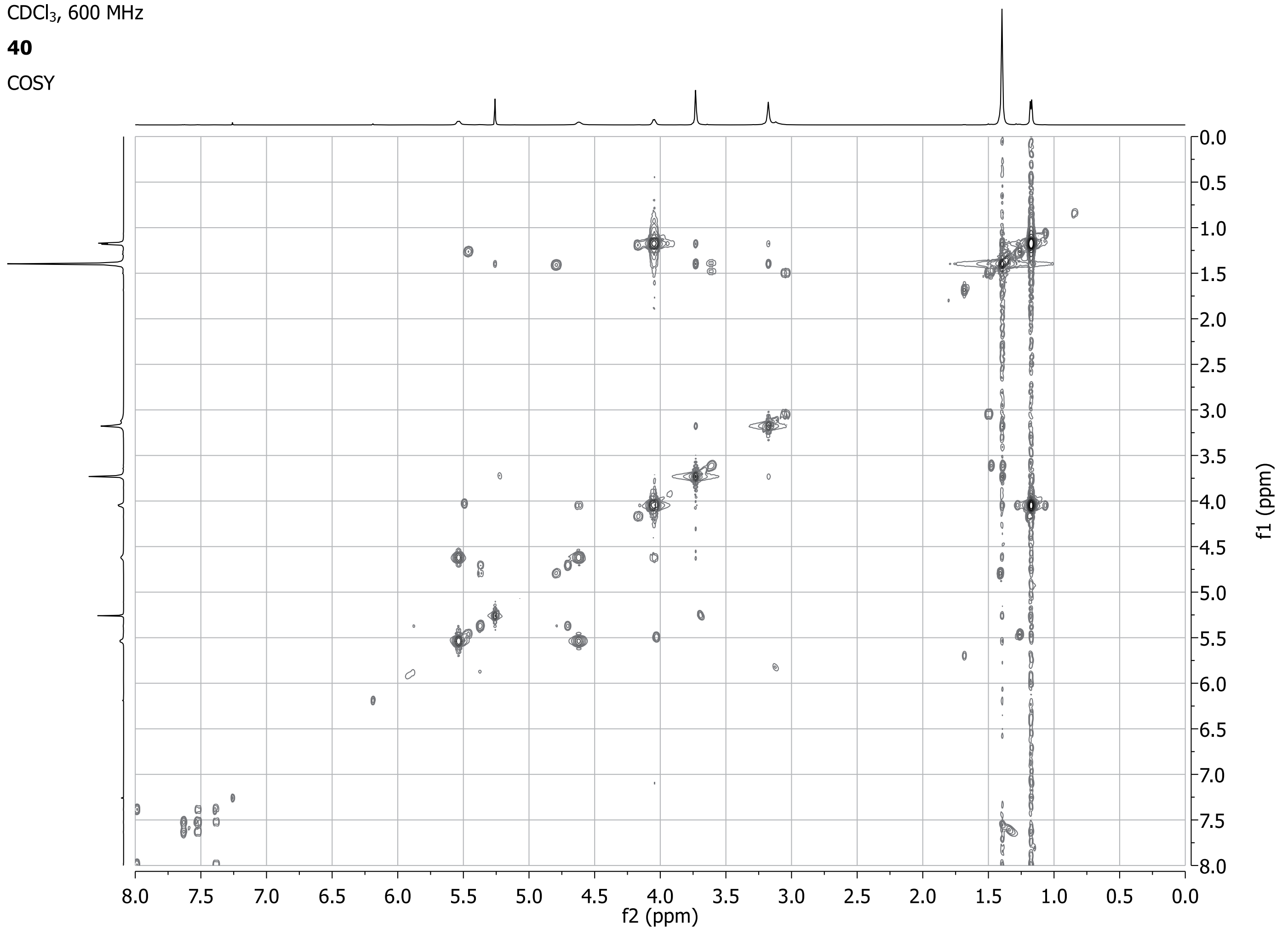
CDCl3, 600 MHz
40
COSY
f2 (ppm)
f1 (ppm)

$CDCl_3$, 600 MHz

40

HMQC

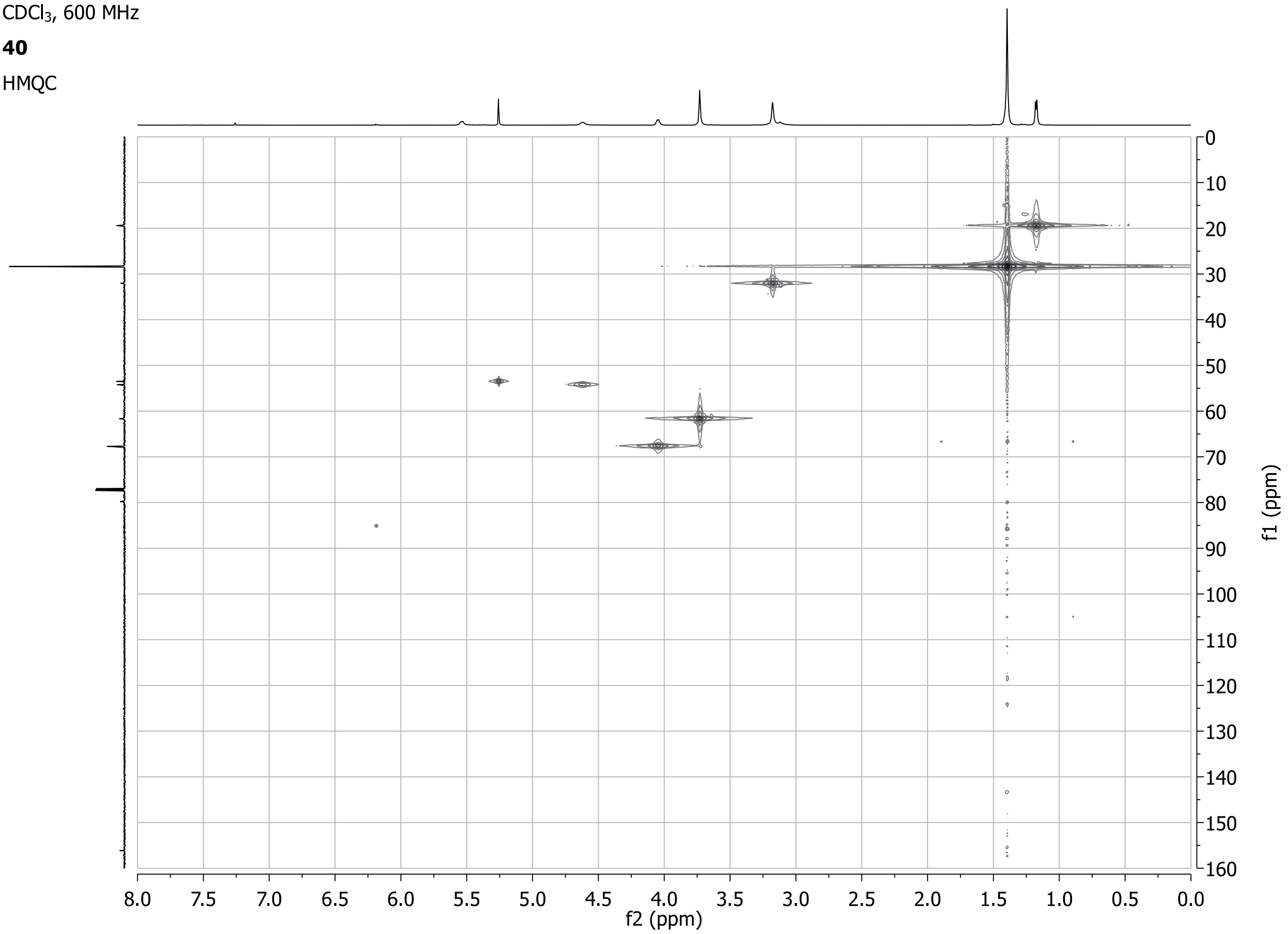

$CDCl_3$, 600 MHz

40

HMBC

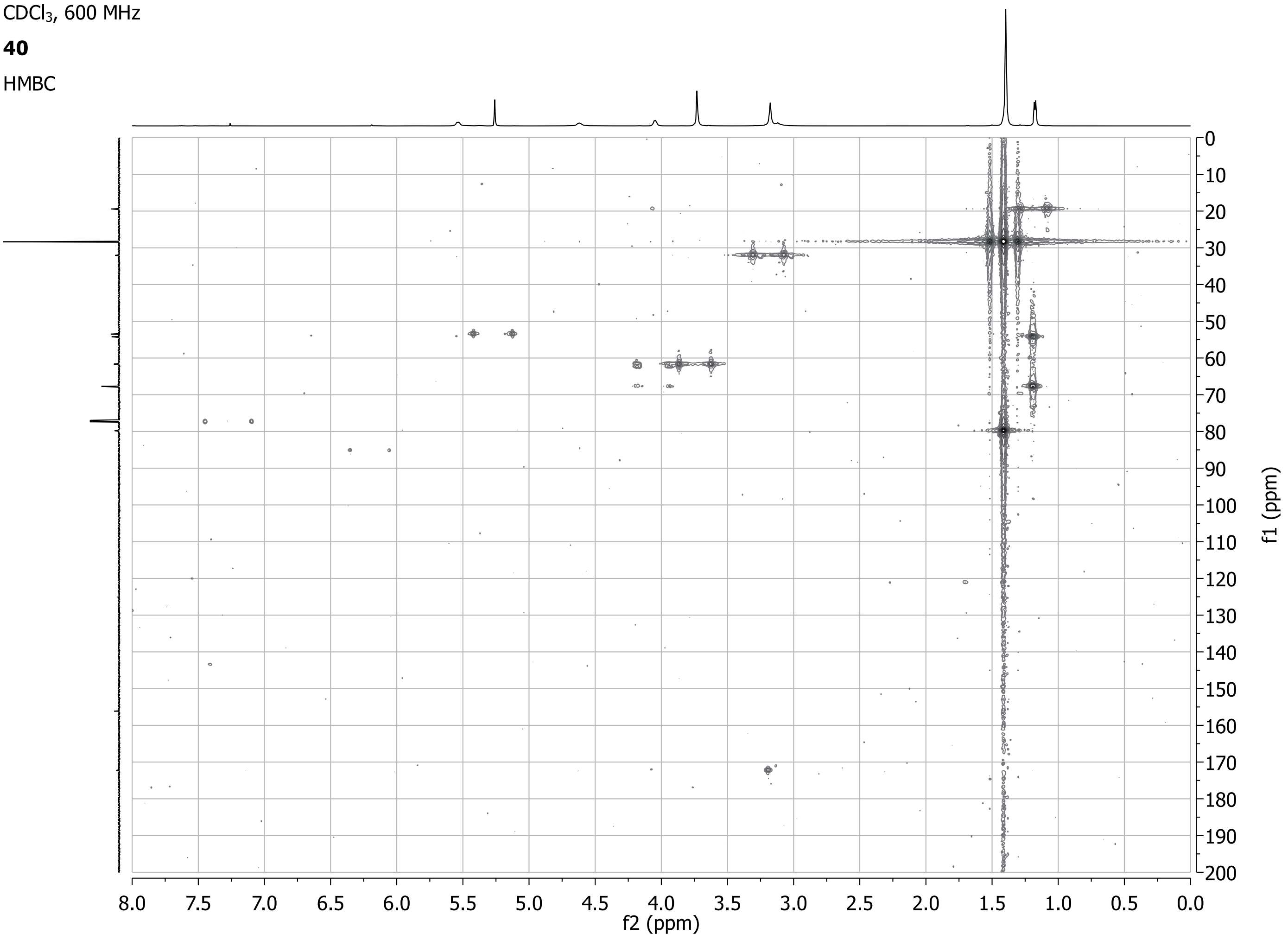

CDCl$_3$, 600 MHz

24

^{1}H NMR

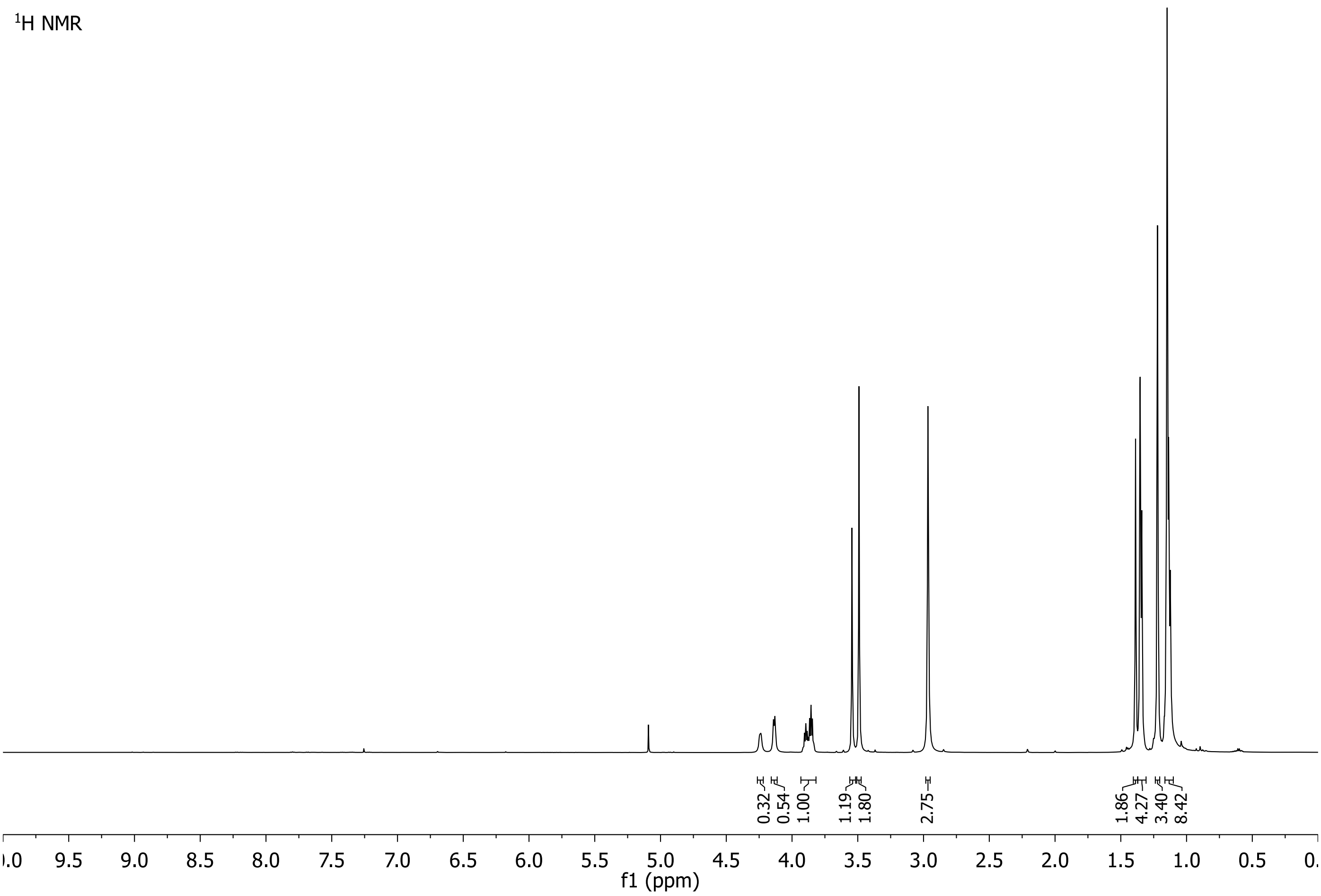

CDCl$_3$, 600 MHz

24

^{13}C NMR

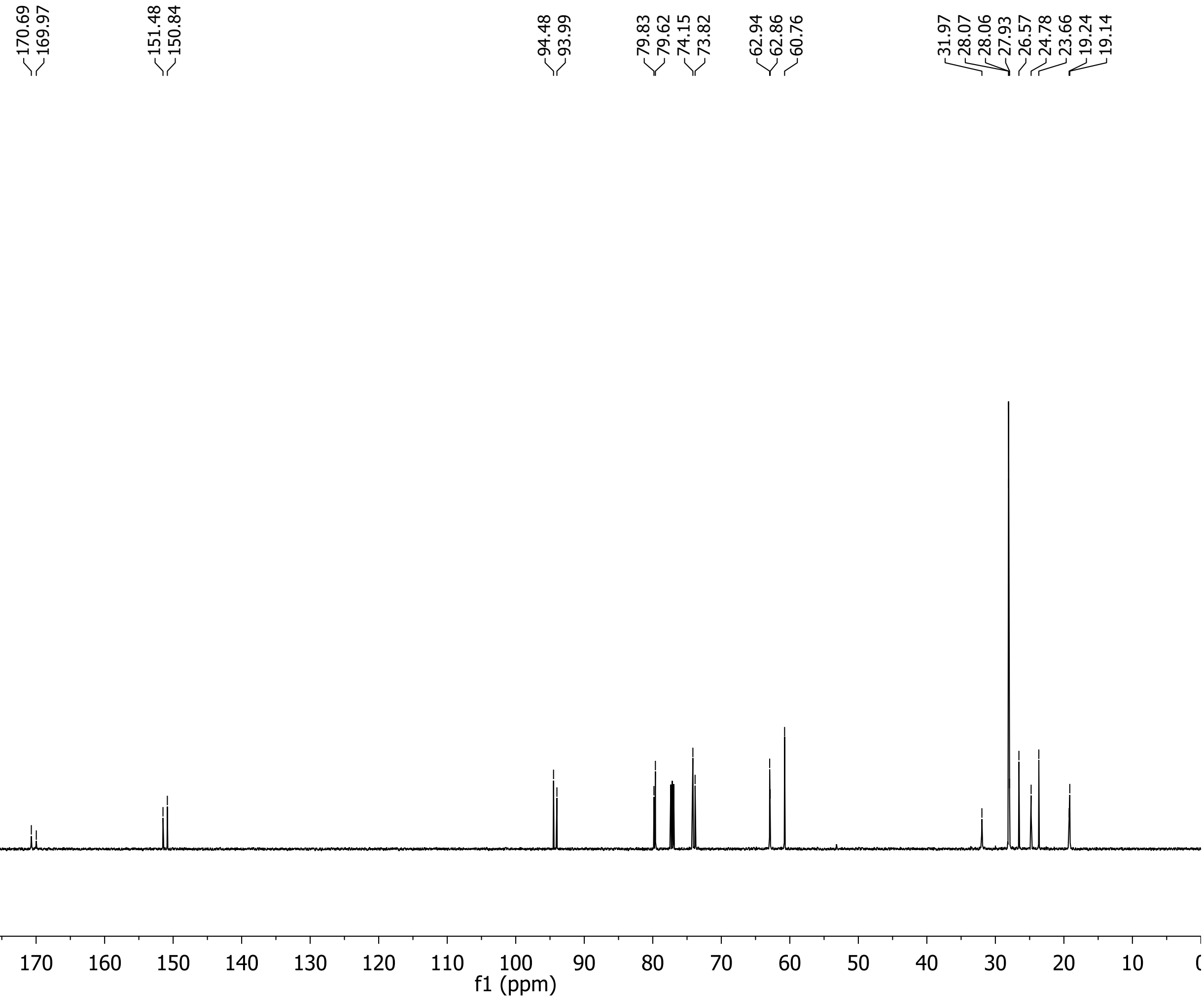

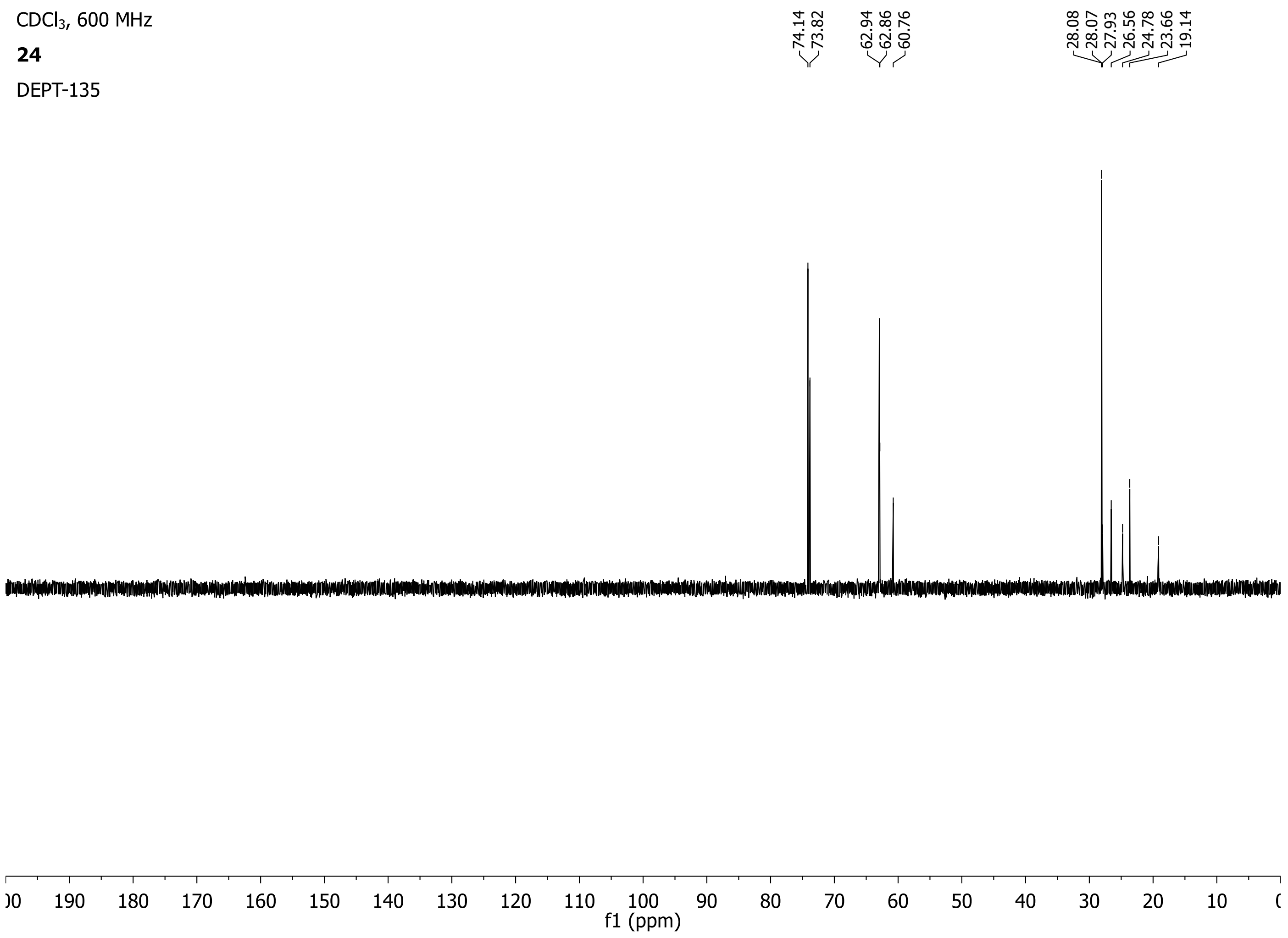
CDCl3, 600 MHz
24
DEPT-135
74.14
73.82
62.94
62.86
60.76
28.08
28.07
27.93
26.56
24.78
23.66
19.14
f1 (ppm)

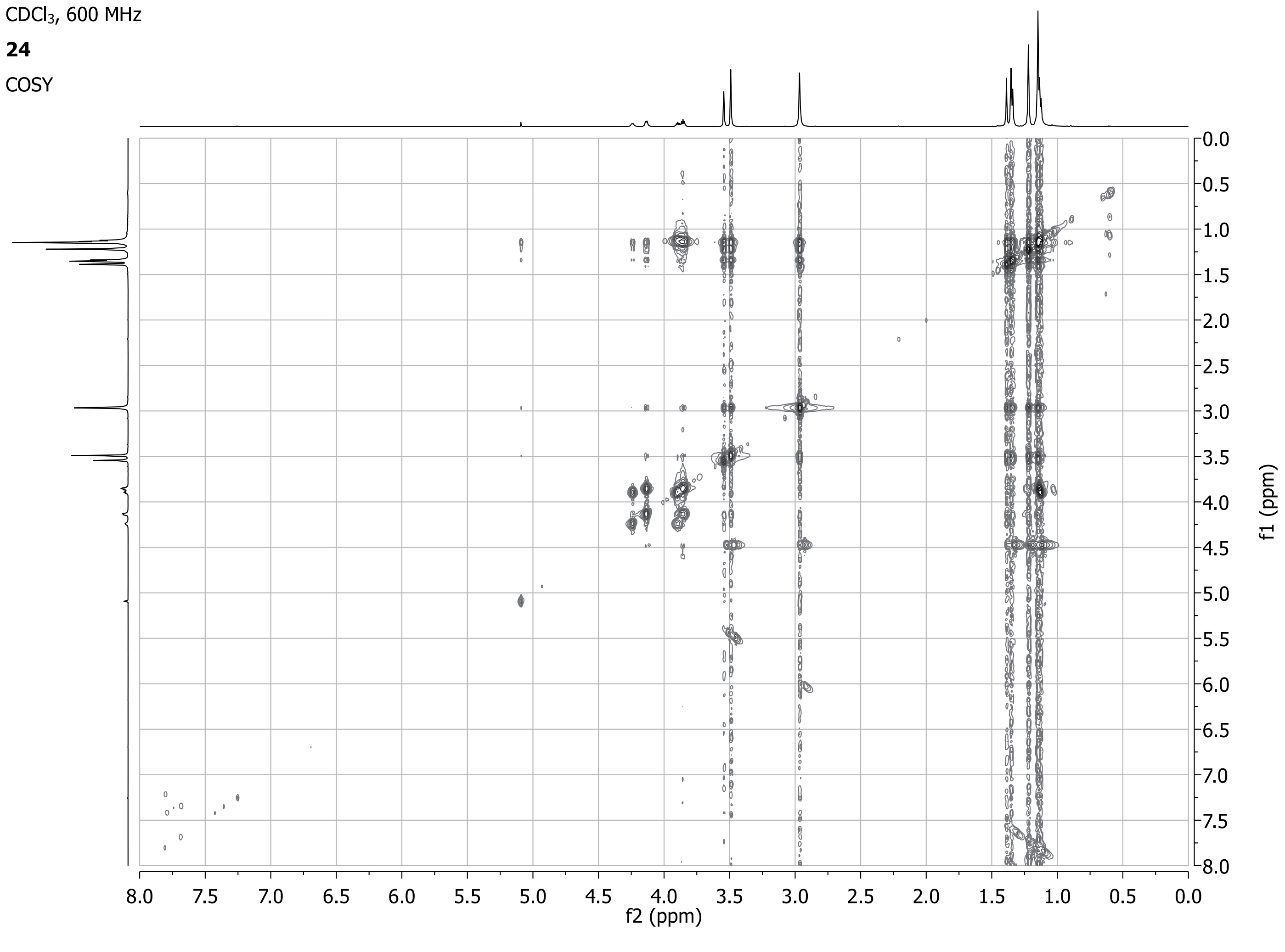
CDCl3, 600 MHz
24
COSY
f2 (ppm)
f1 (ppm)

CDCl$_3$, 600 MHz

24

HMQC

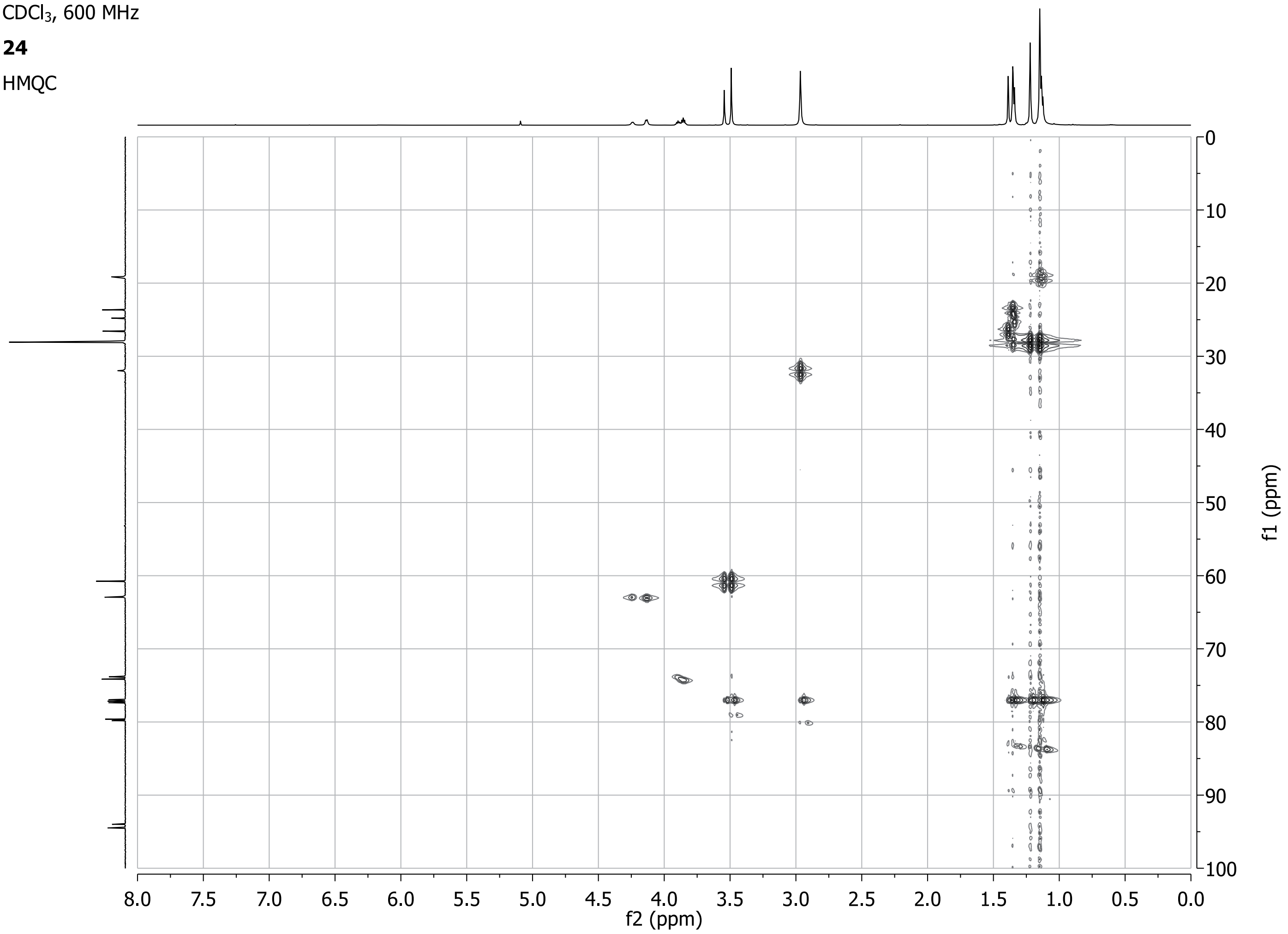

CDCl$_3$, 600 MHz

24

HMBC

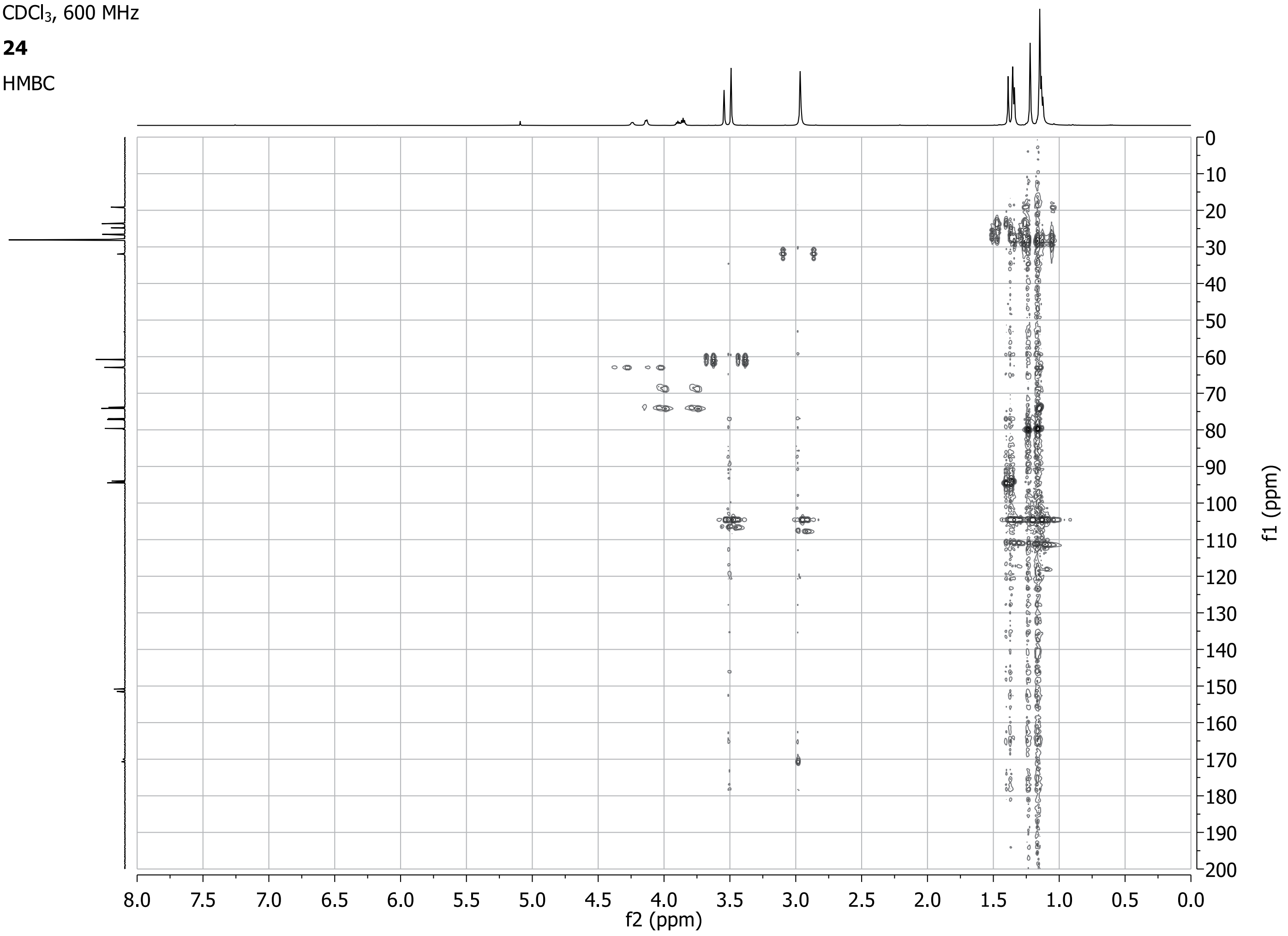

DMSO, 500 MHz
100 °C
25
^{1}H NMR

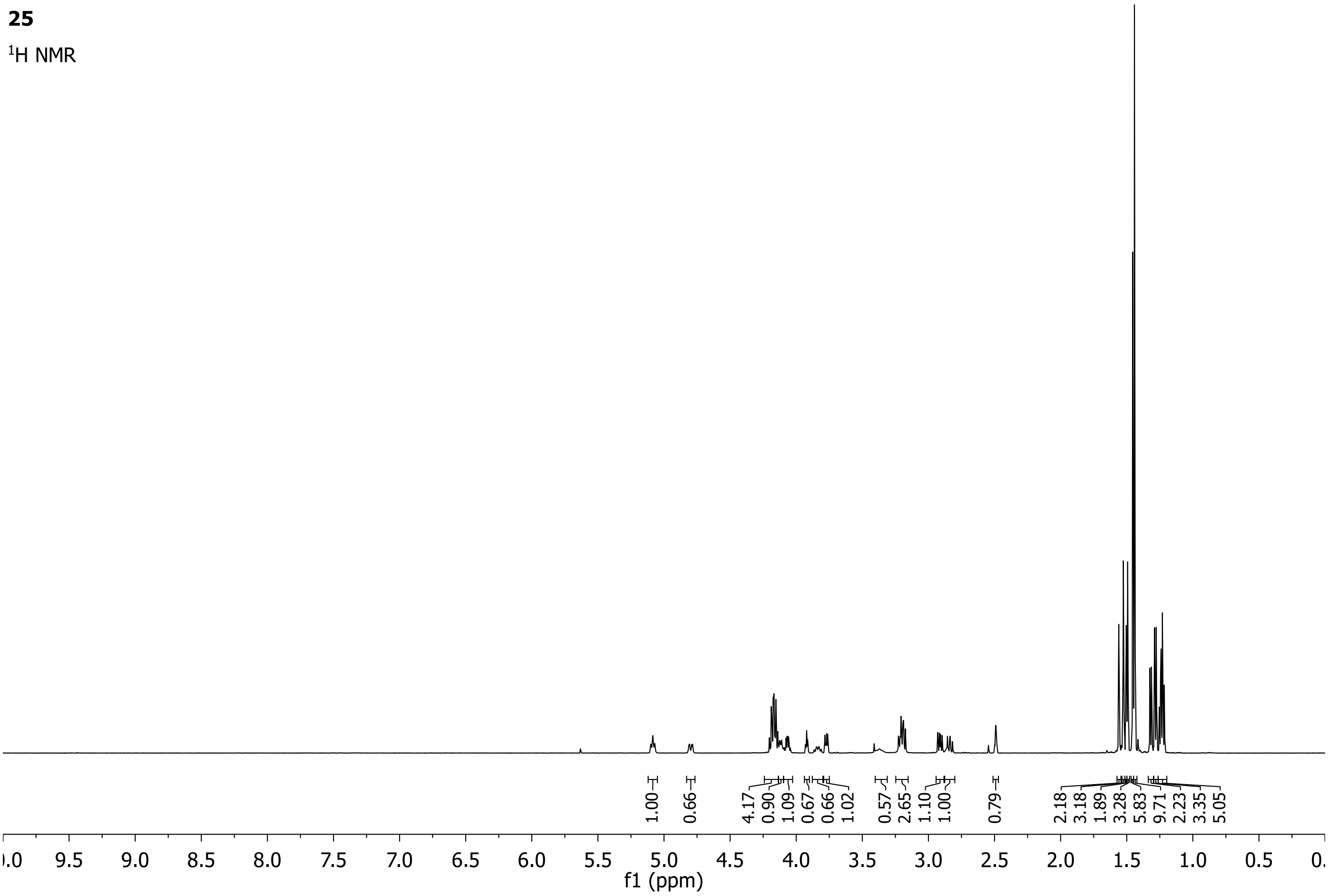

DMSO, 500 MHz
100 °C
25
^{13}C NMR

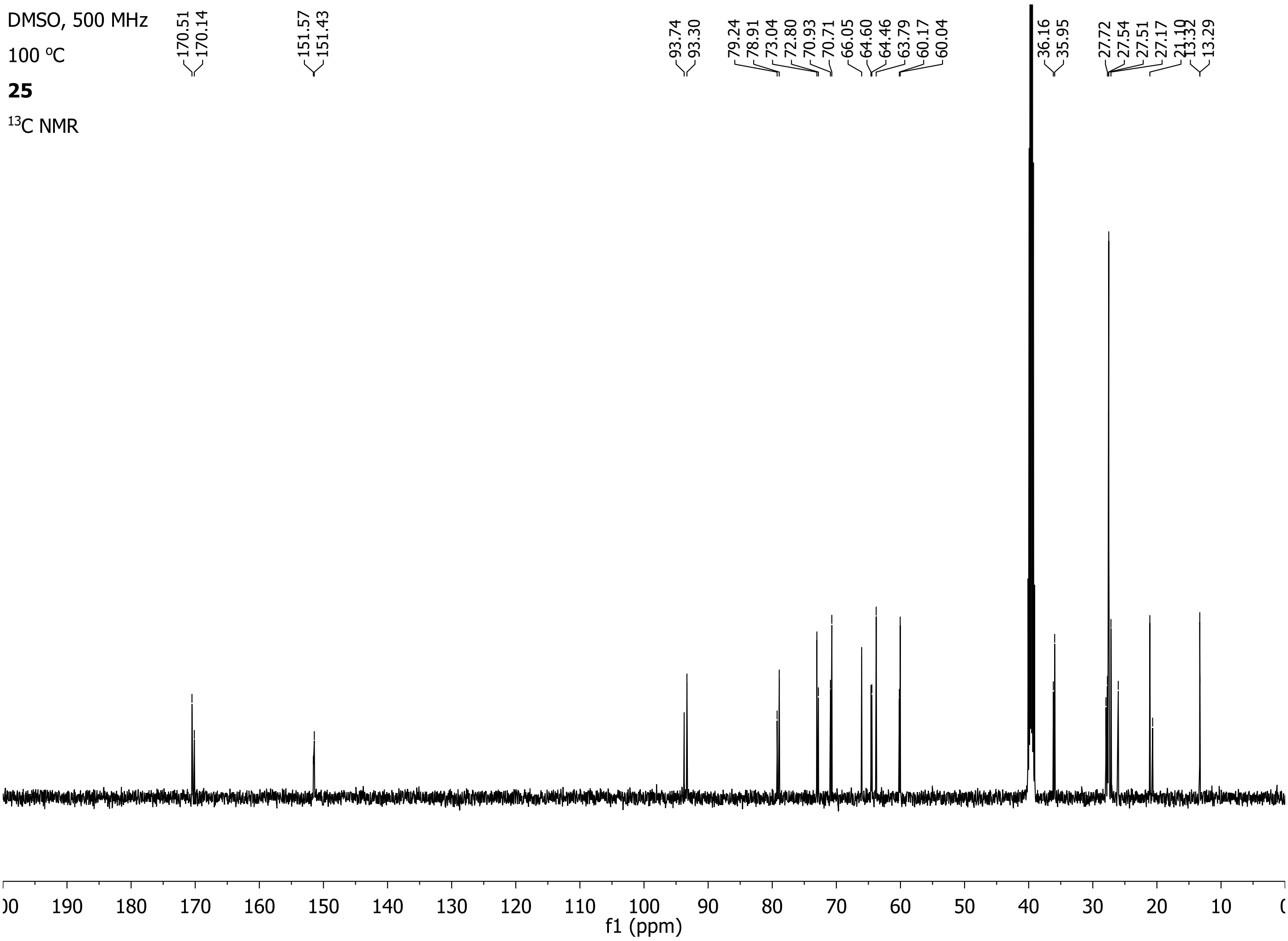

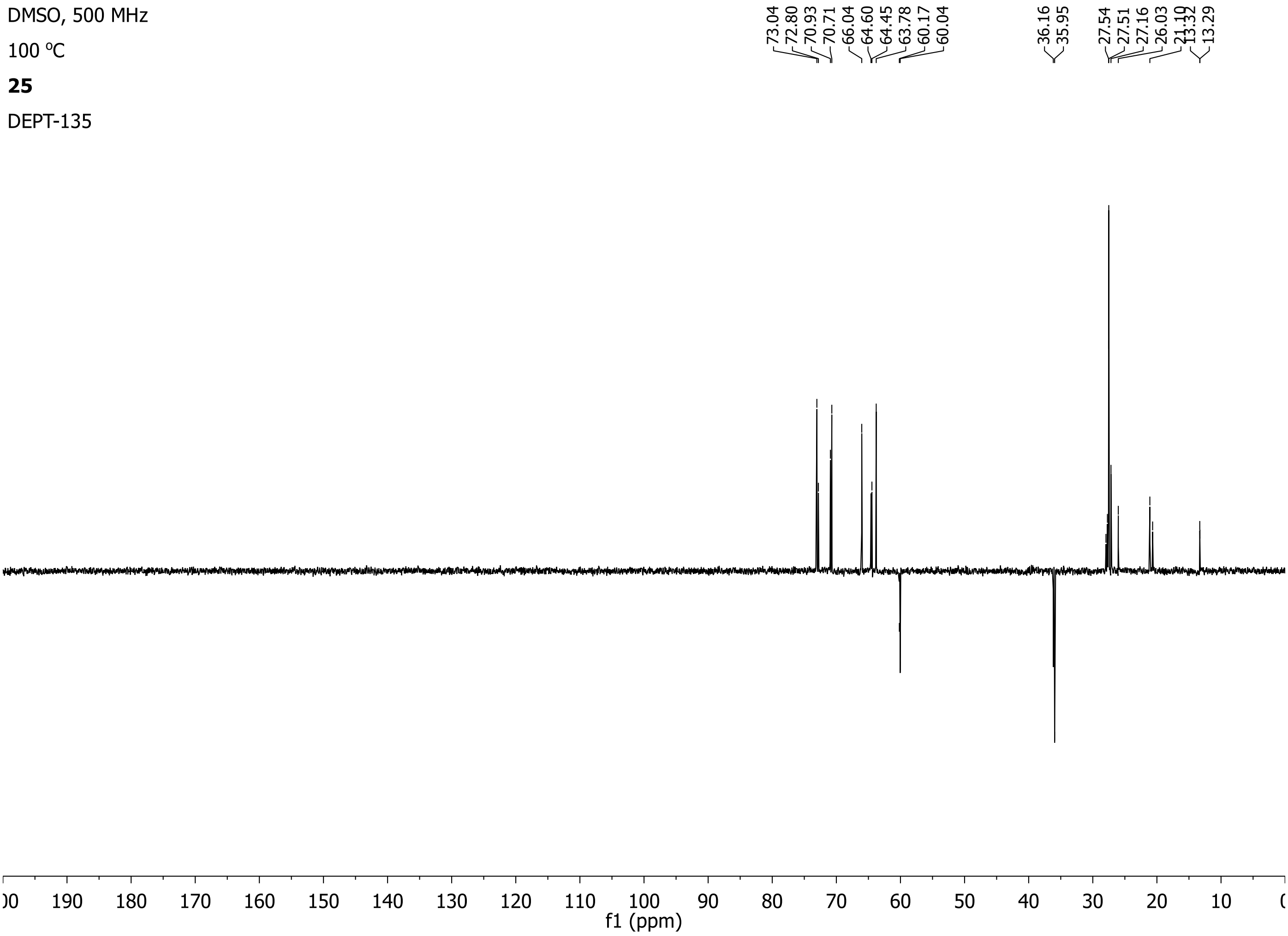
DMSO, 500 MHz
100 °C
25
DEPT-135
73.04
72.80
70.93
70.71
66.04
64.60
64.45
63.78
60.17
60.04
36.16
35.95
27.54
27.51
27.16
26.03
21.10
13.32
13.29
f1 (ppm)

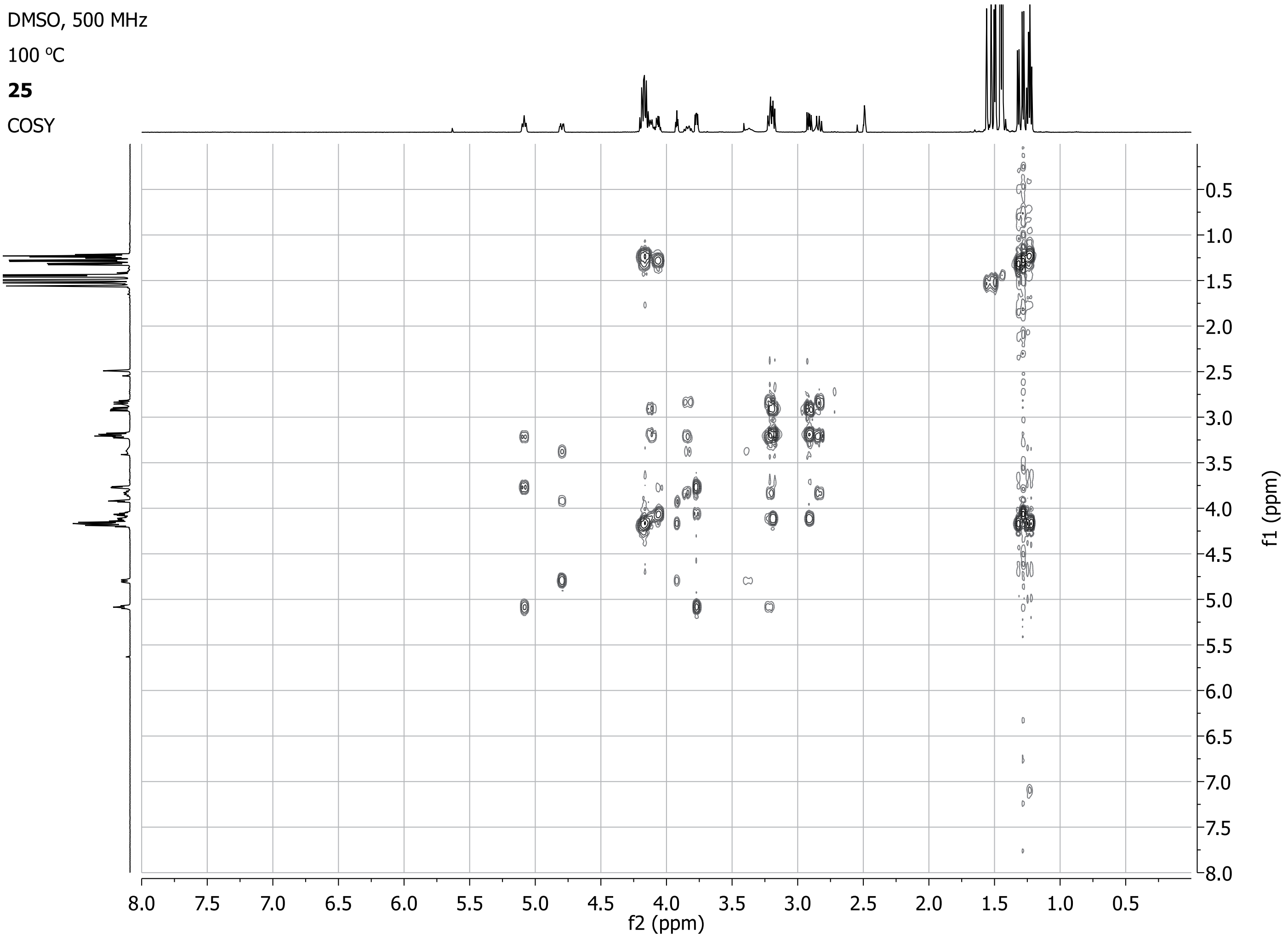
DMSO, 500 MHz
100 °C
25
COSY
f2 (ppm)
f1 (ppm)

DMSO, 500 MHz

100 °C

25

HMQC

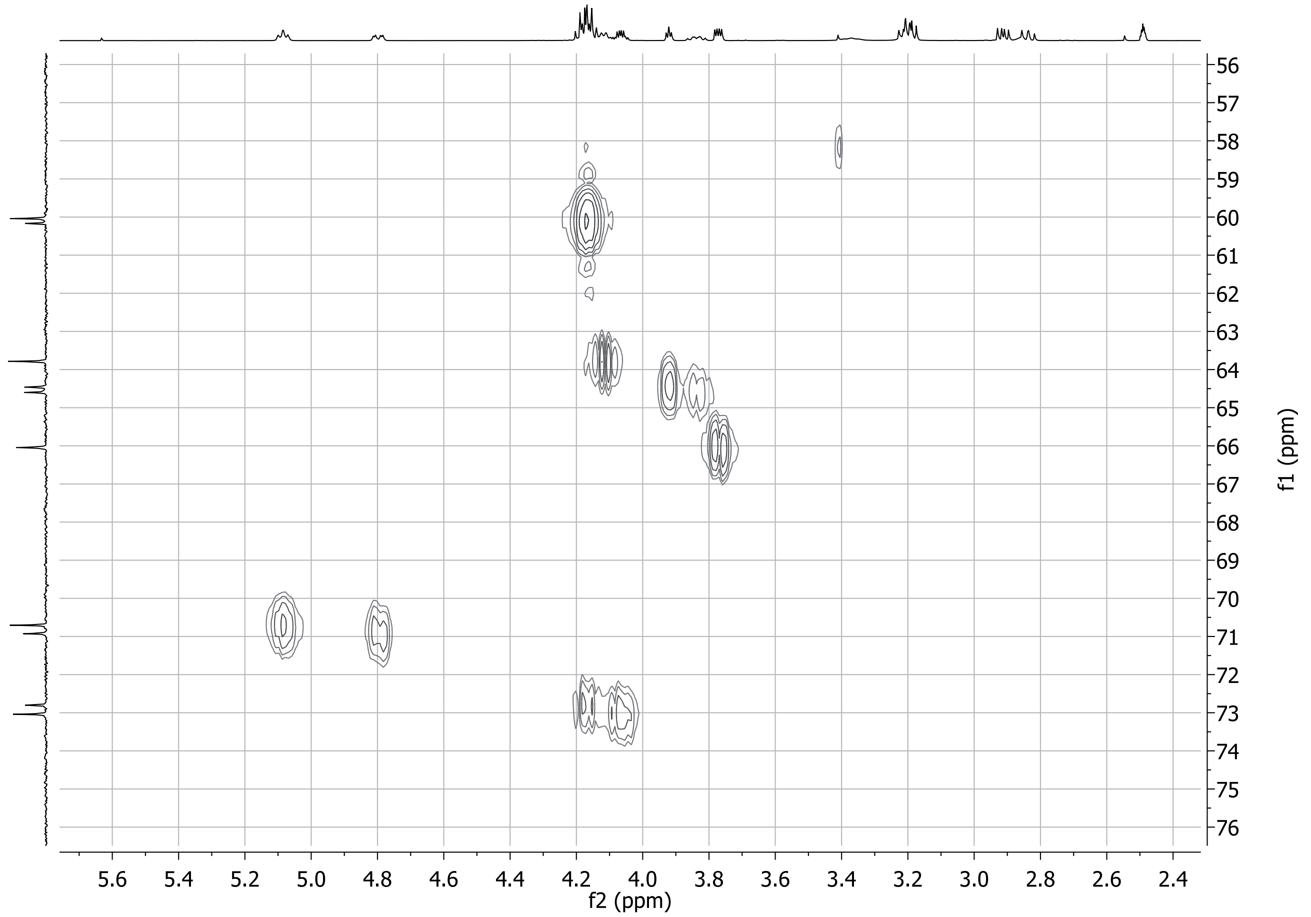

DMSO, 500 MHz

100 °C

25

HMBC

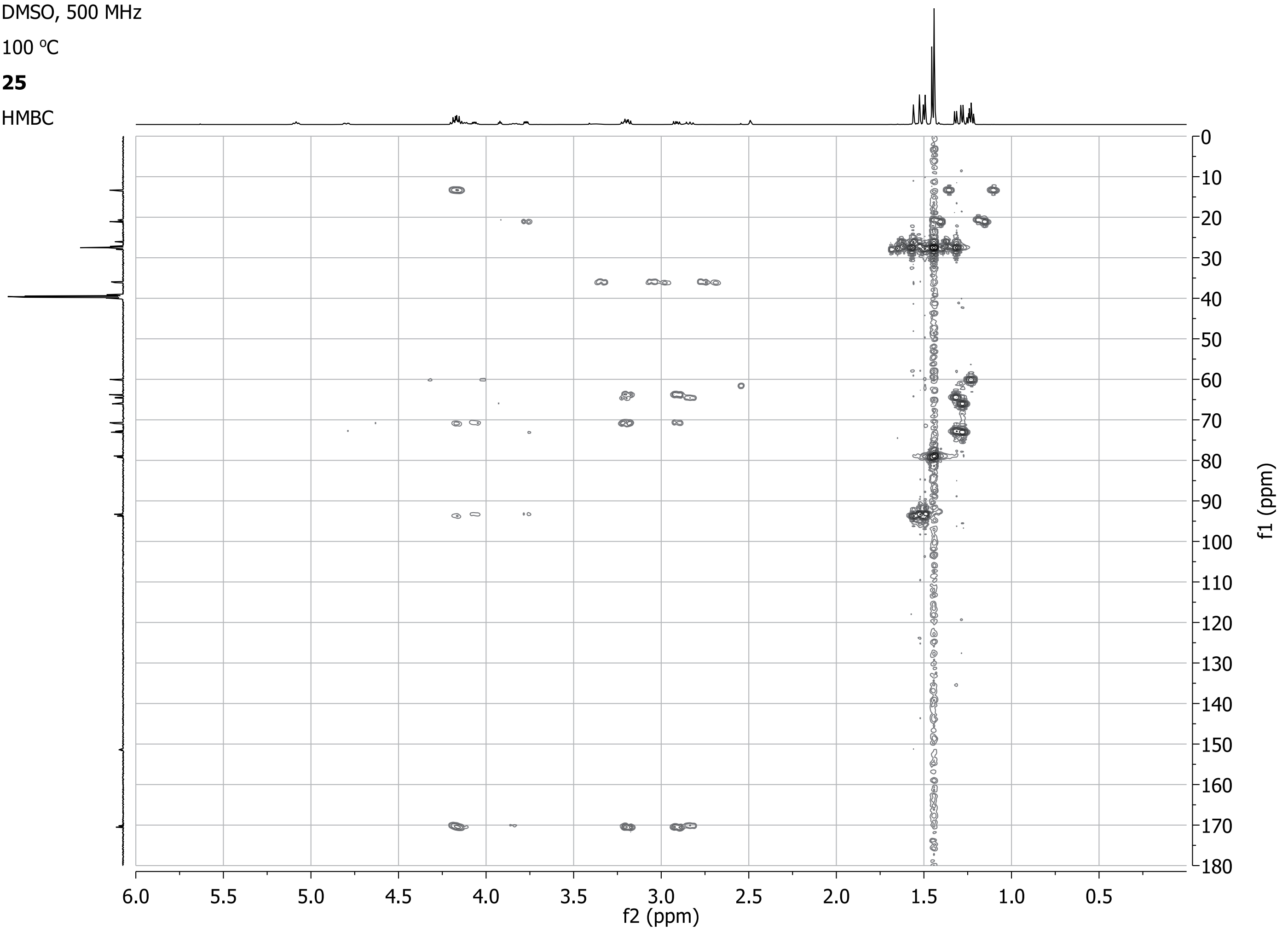

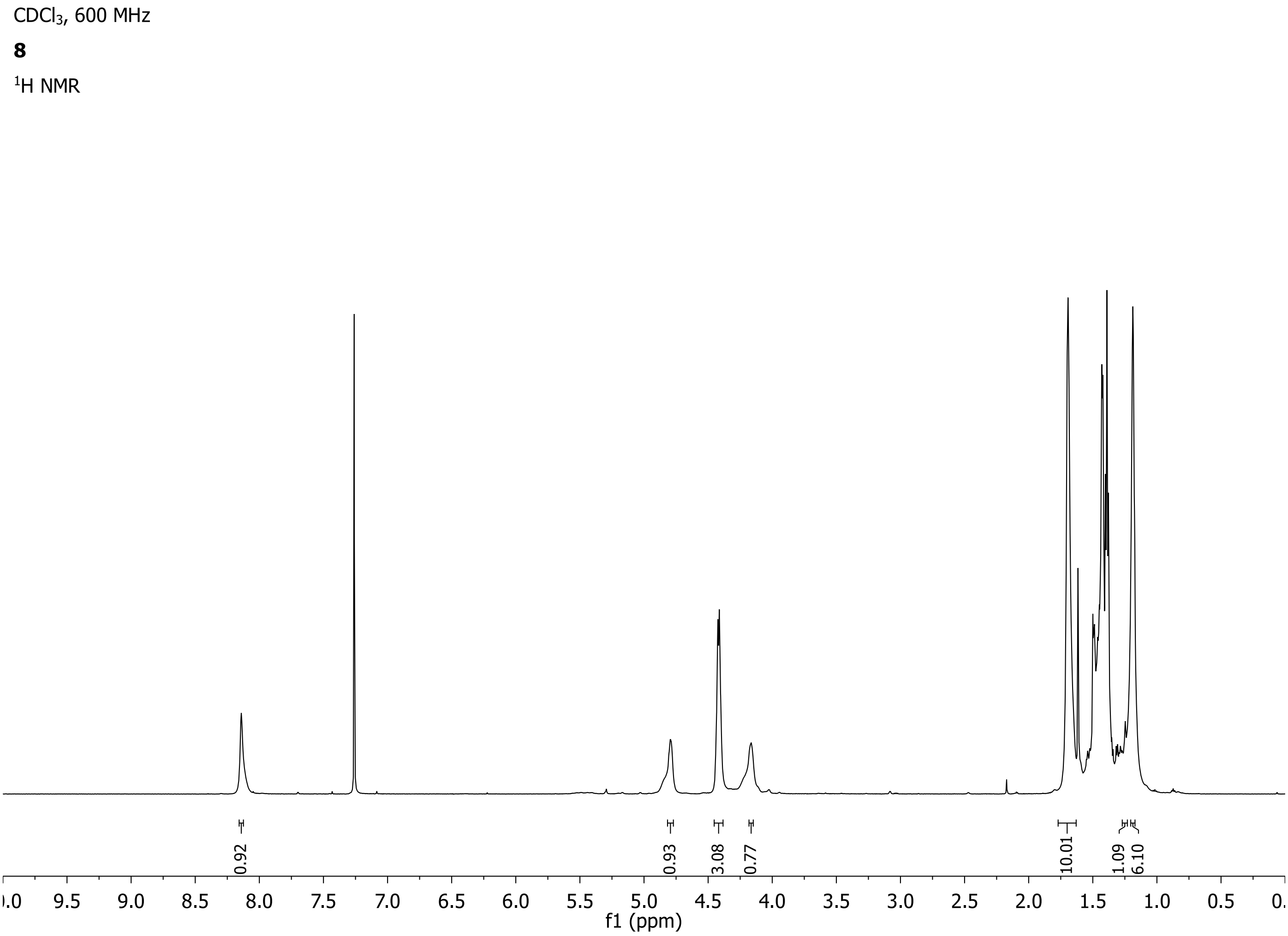
CDCl3, 600 MHz
8
1H NMR
0.92
0.93
3.08
0.77
10.01
1.09
6.10
f1 (ppm)

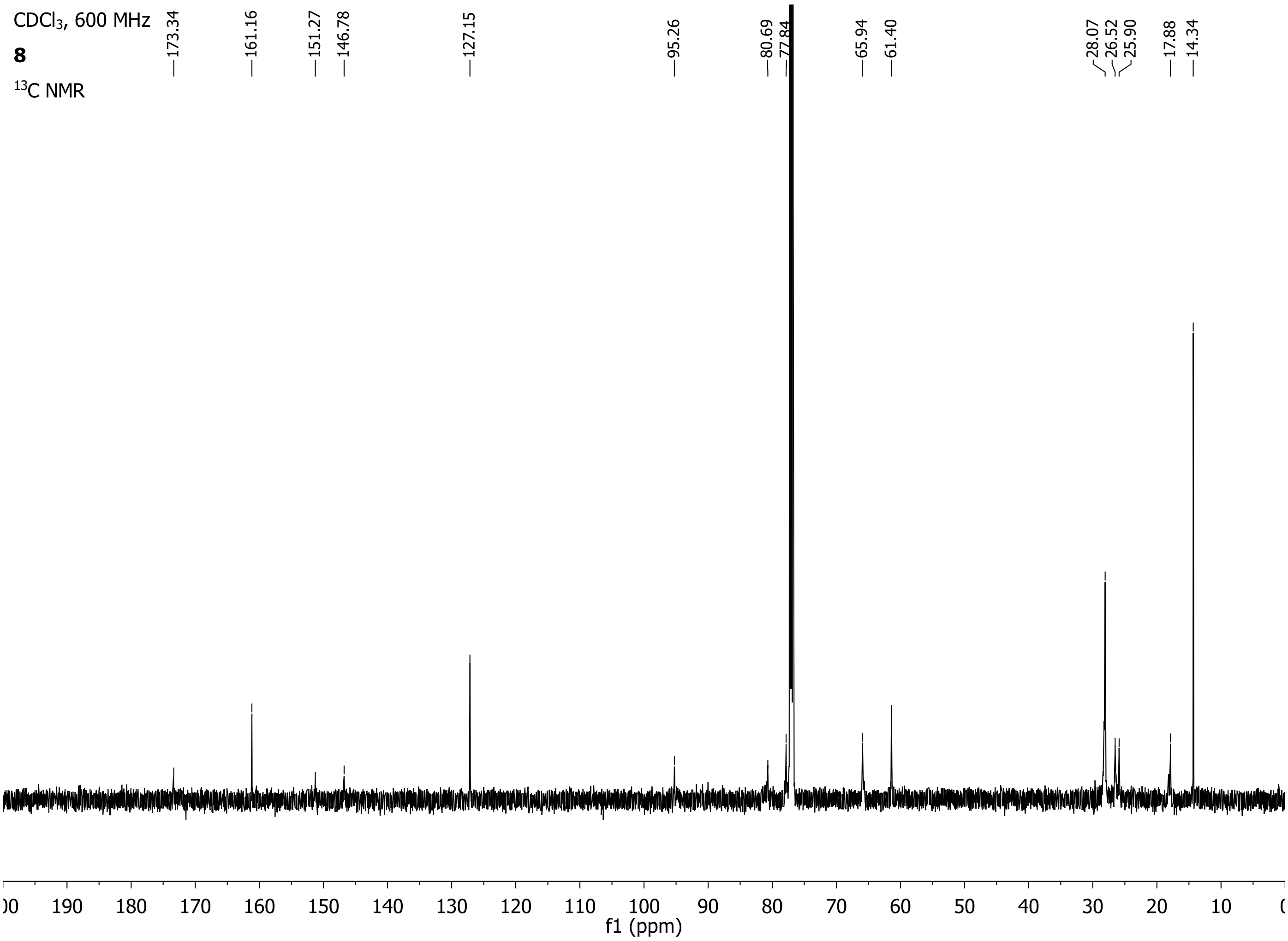
CDCl3, 600 MHz
8
13C NMR
173.34
161.16
151.27
146.78
127.15
95.26
80.69
77.84
65.94
61.40
28.07
26.52
25.90
17.88
14.34
f1 (ppm)

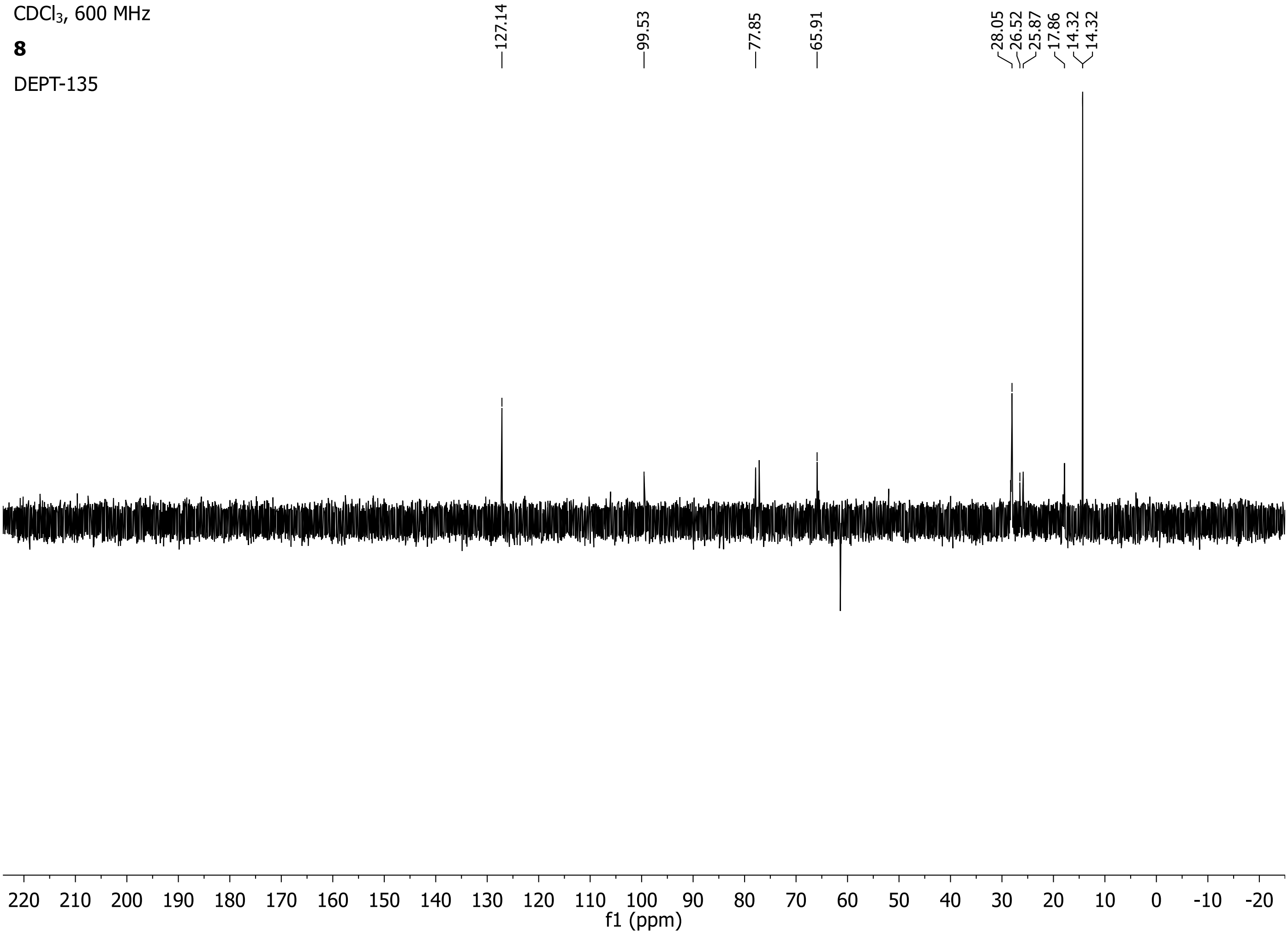

CDCl3, 600 MHz
8
DEPT-135
127.14
99.53
77.85
65.91
28.05
26.52
25.87
17.86
14.32
14.32
220 210 200 190 180 170 160 150 140 130 120 110 100 90 80 70 60 50 40 30 20 10 0 -10 -20
f1 (ppm)

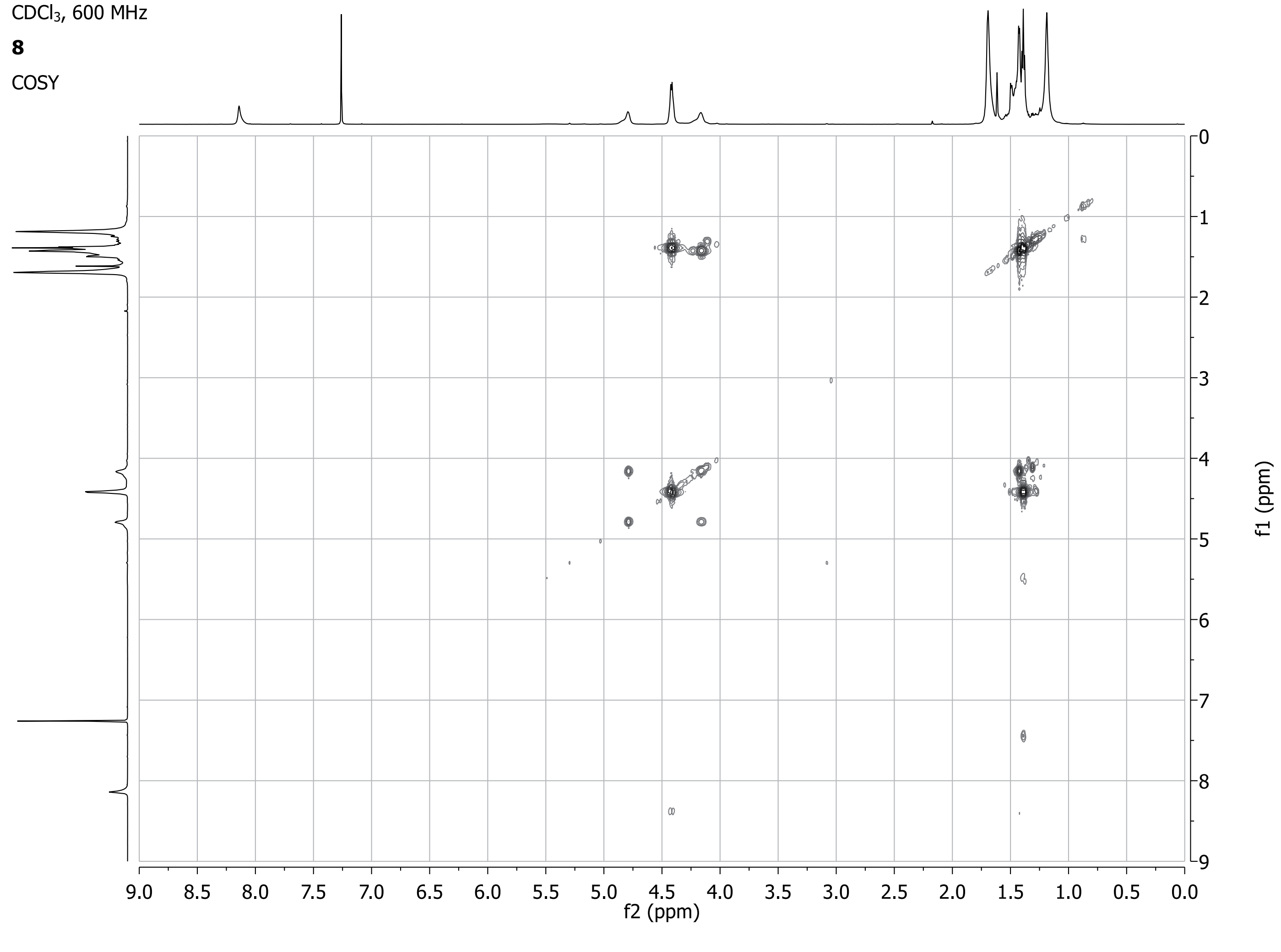

CDCl3, 600 MHz
8
COSY
9.0 8.5 8.0 7.5 7.0 6.5 6.0 5.5 5.0 4.5 4.0 3.5 3.0 2.5 2.0 1.5 1.0 0.5 0.0
f2 (ppm)
0 1 2 3 4 5 6 7 8 9
f1 (ppm)

CDCl$_3$, 600 MHz

8

HMQC

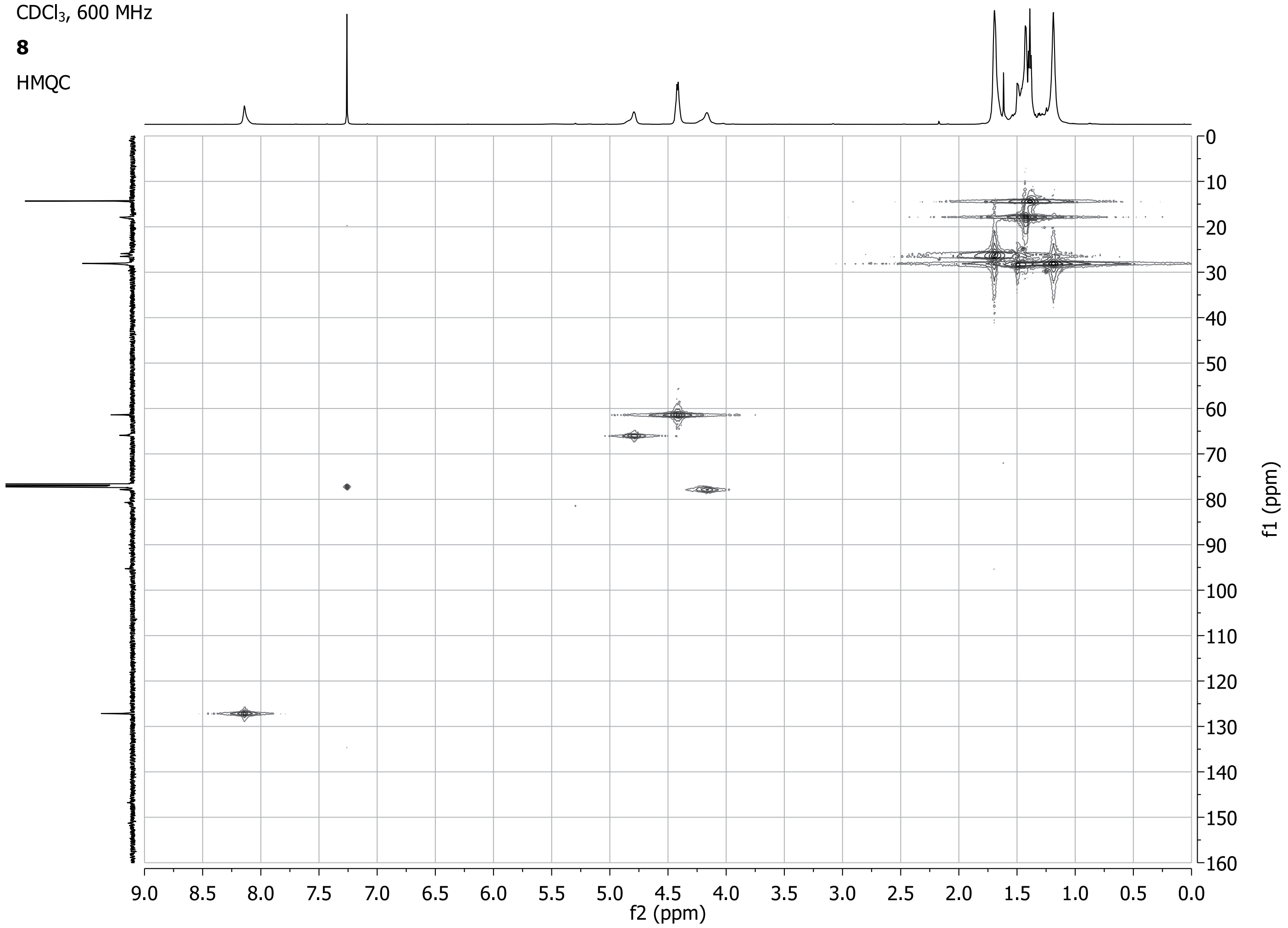

CDCl$_3$, 600 MHz

8

HMBC

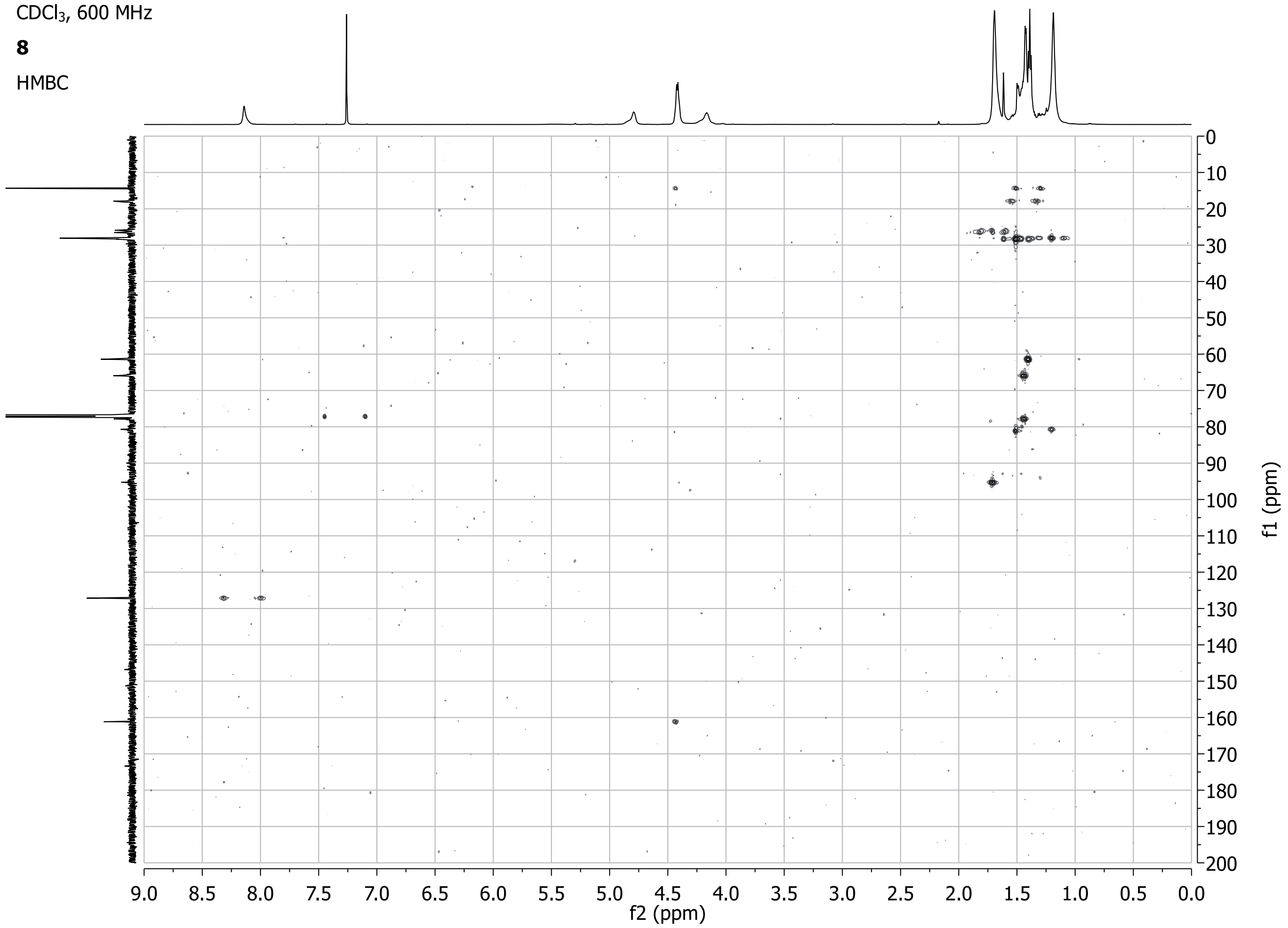

CDCl3, 600 MHz

26

^{1}H NMR

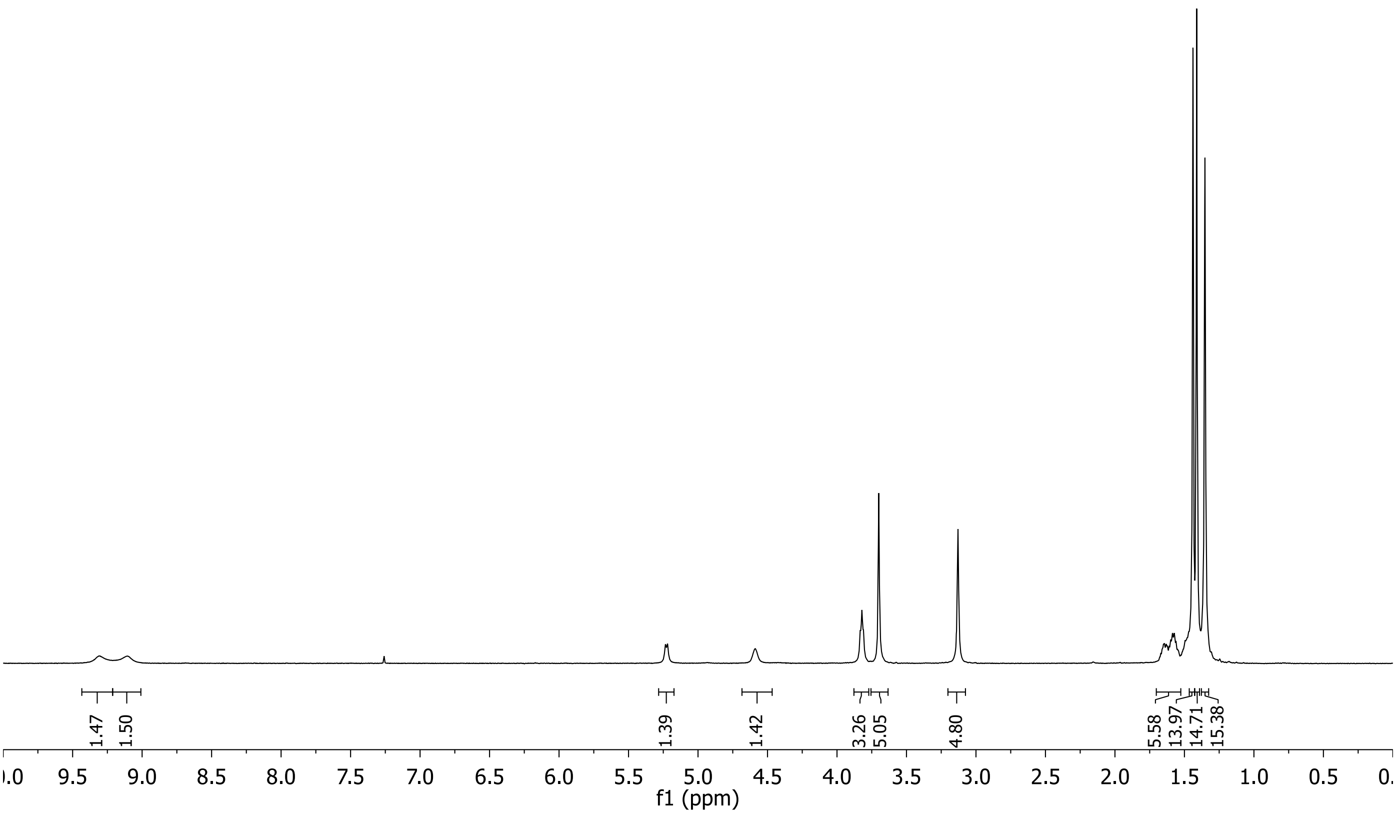

CDCl3, 600 MHz

26

^{13}C NMR

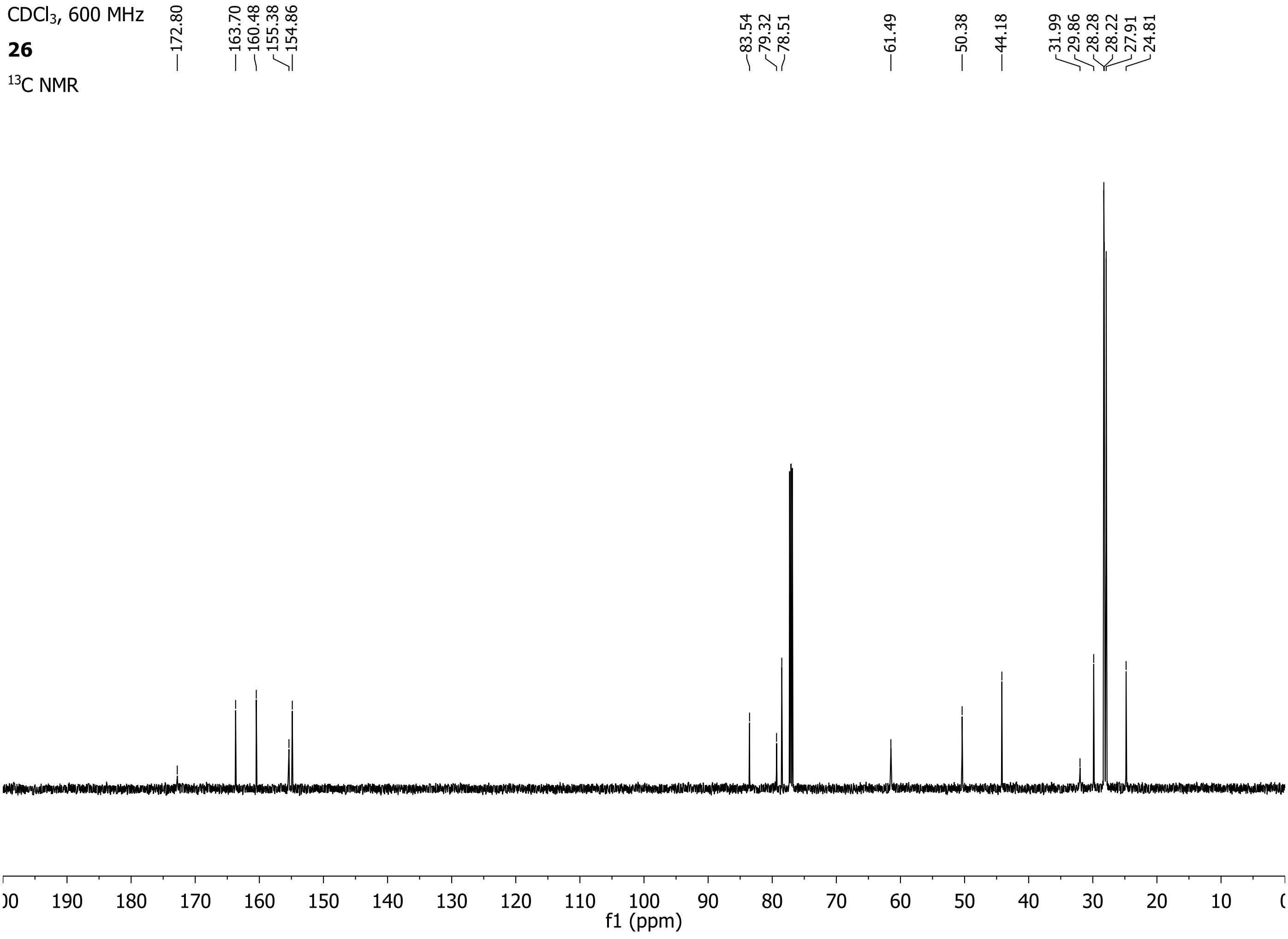

CDCl$_3$, 600 MHz

26

DEPT-135

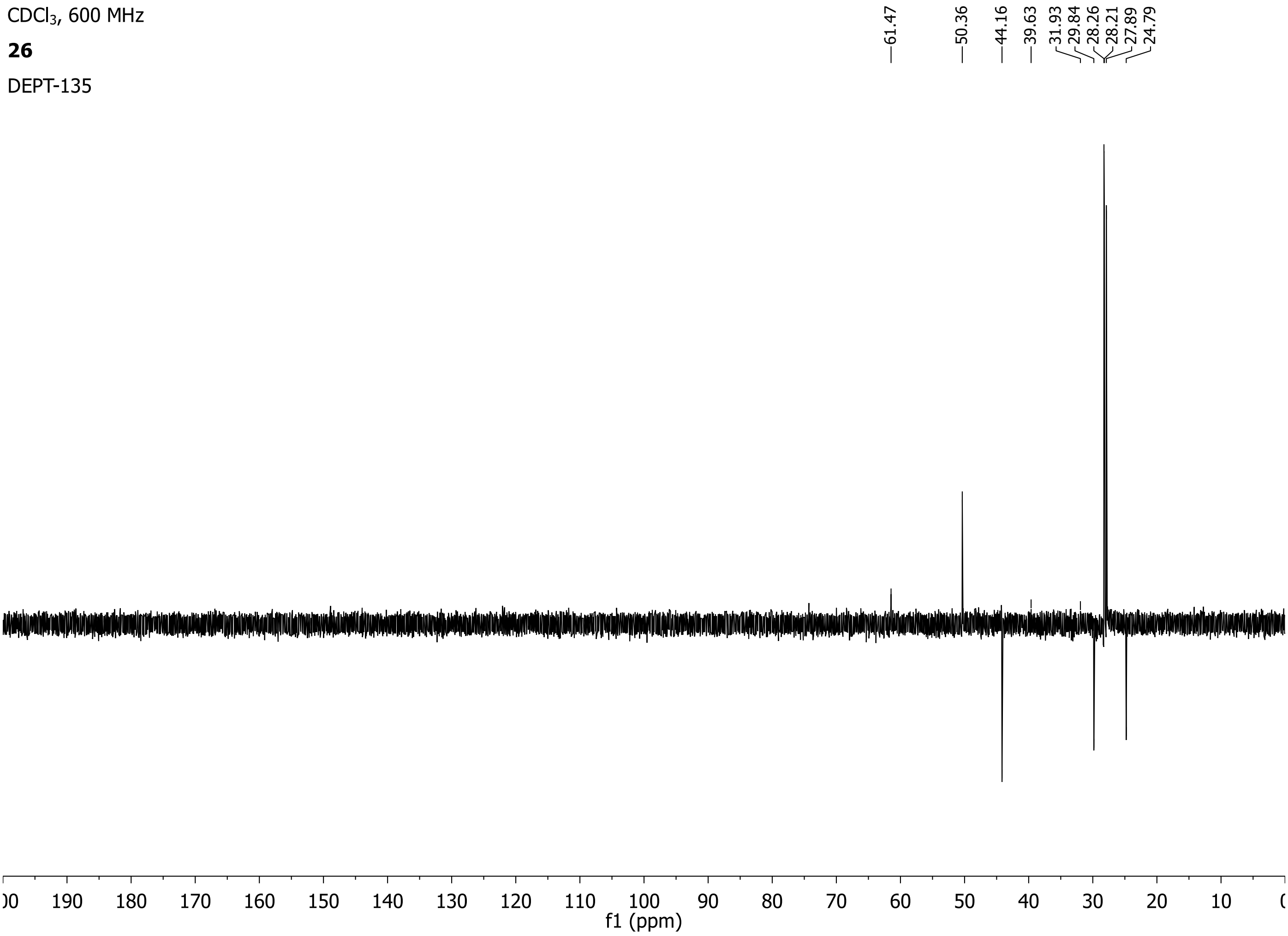

CDCl$_3$, 600 MHz

26

COSY

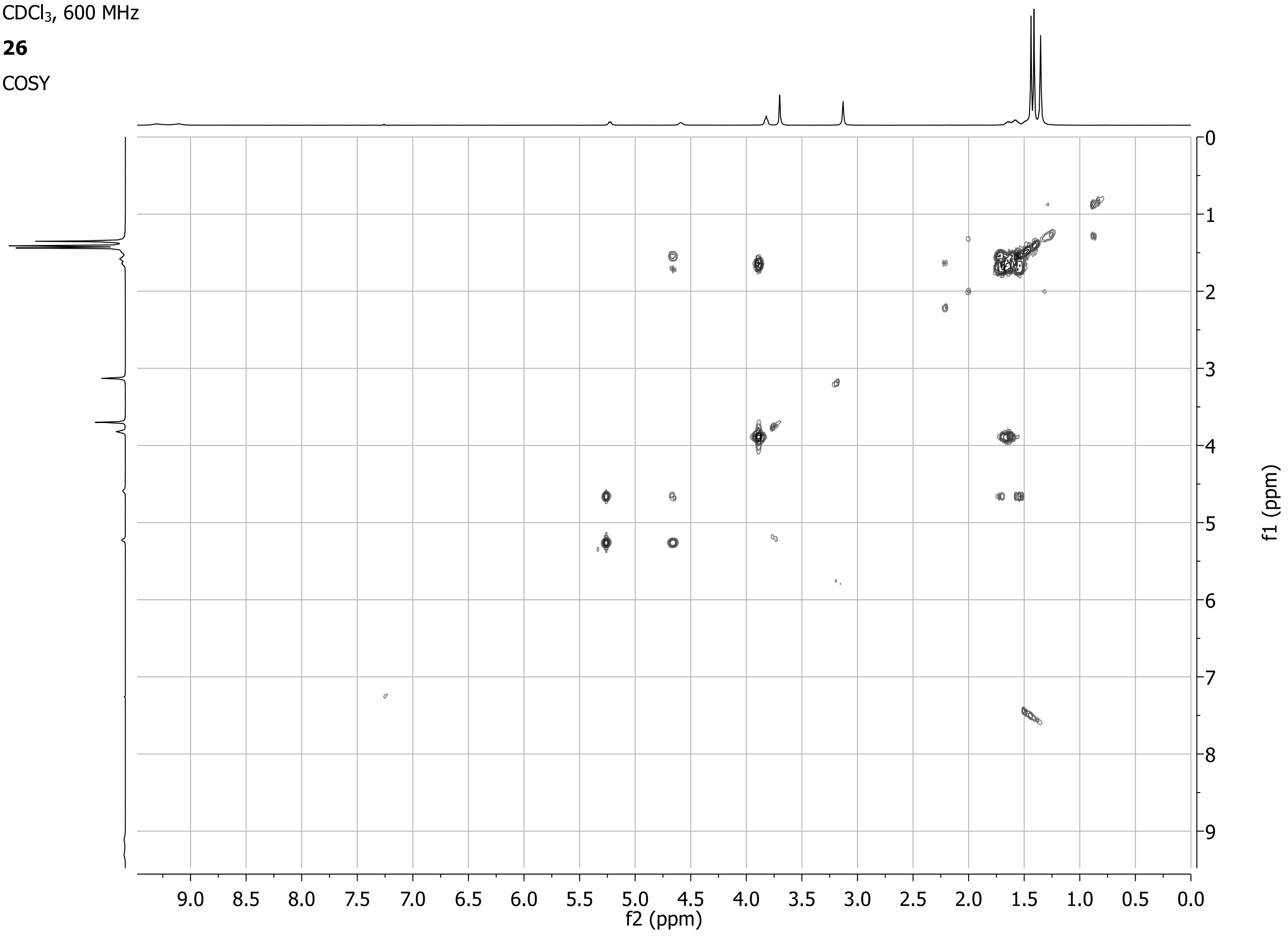

CDCl$_3$, 600 MHz

26

HMQC

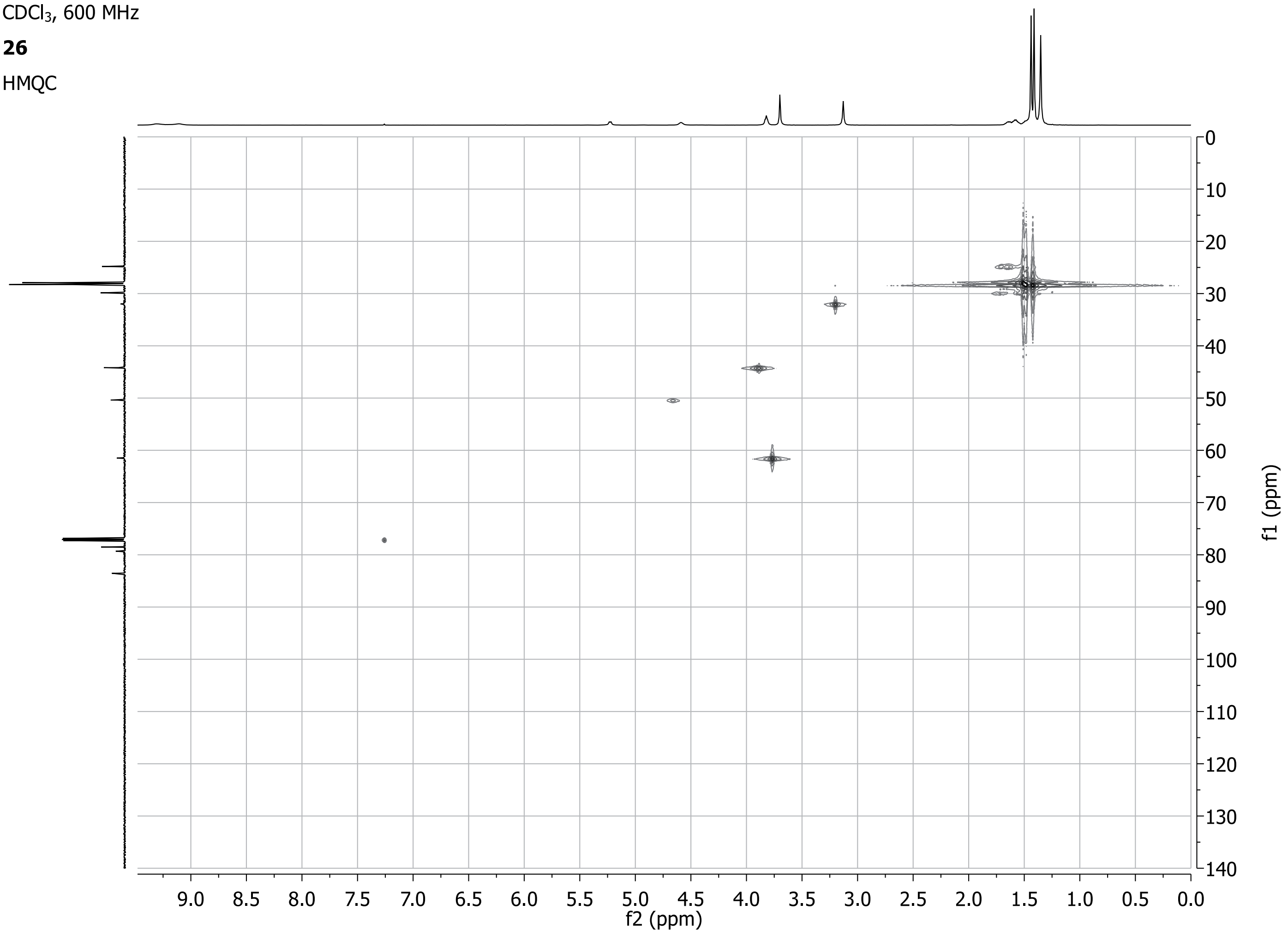

CDCl$_3$, 600 MHz

26

HMBC

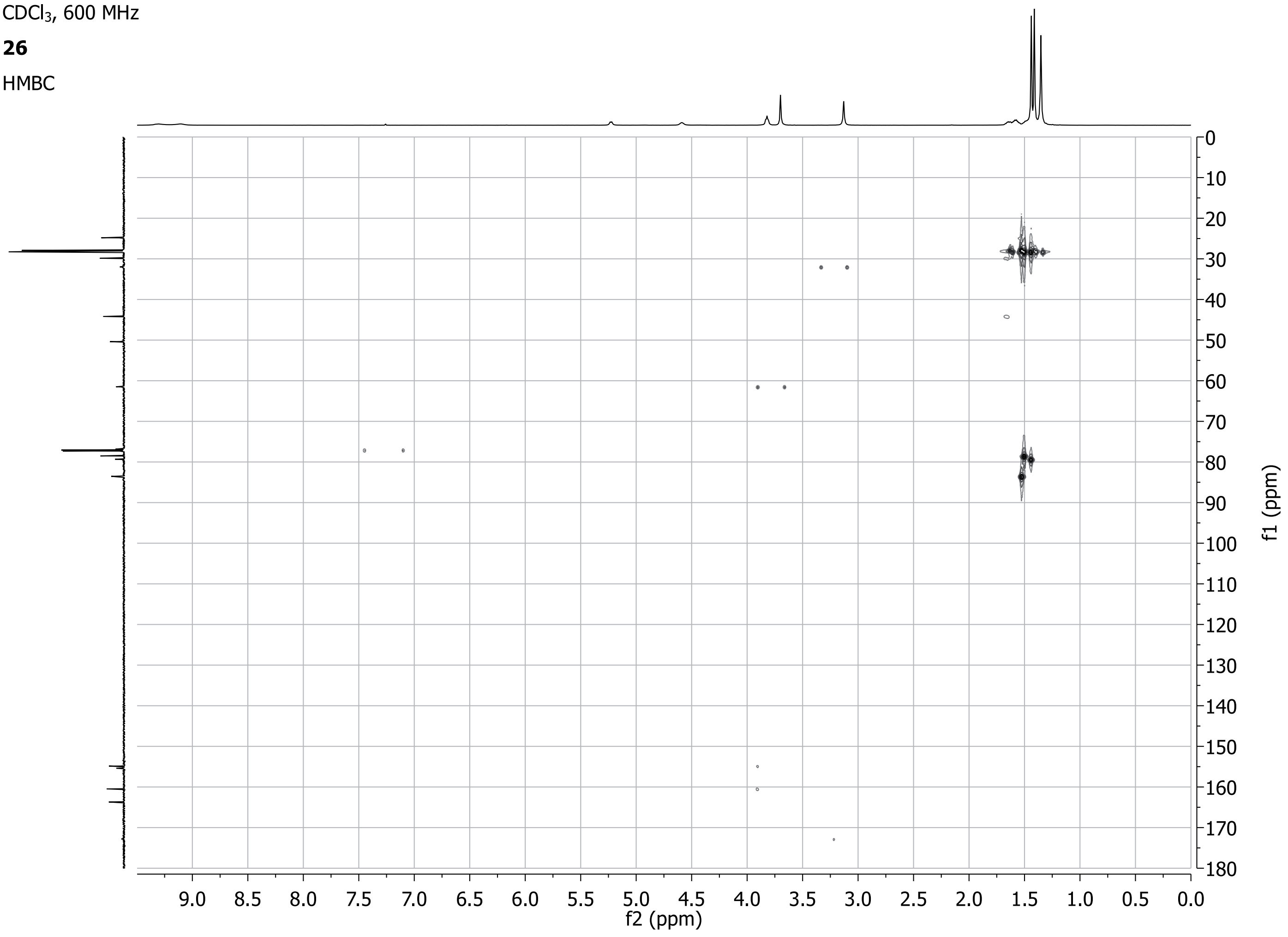

CDCl$_3$, 600 MHz

27

^{1}H NMR

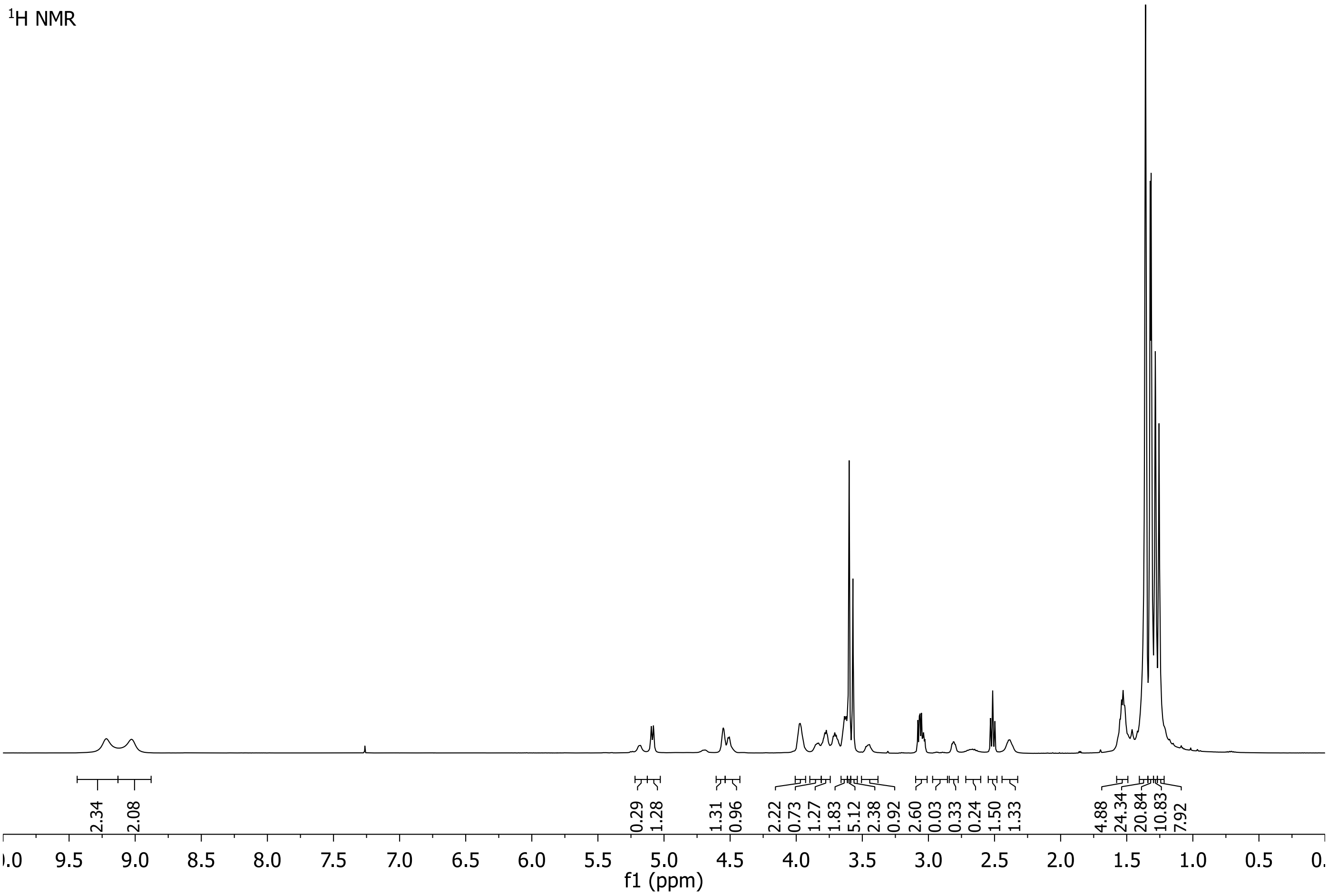

CDCl$_3$, 600 MHz

27

^{13}C NMR

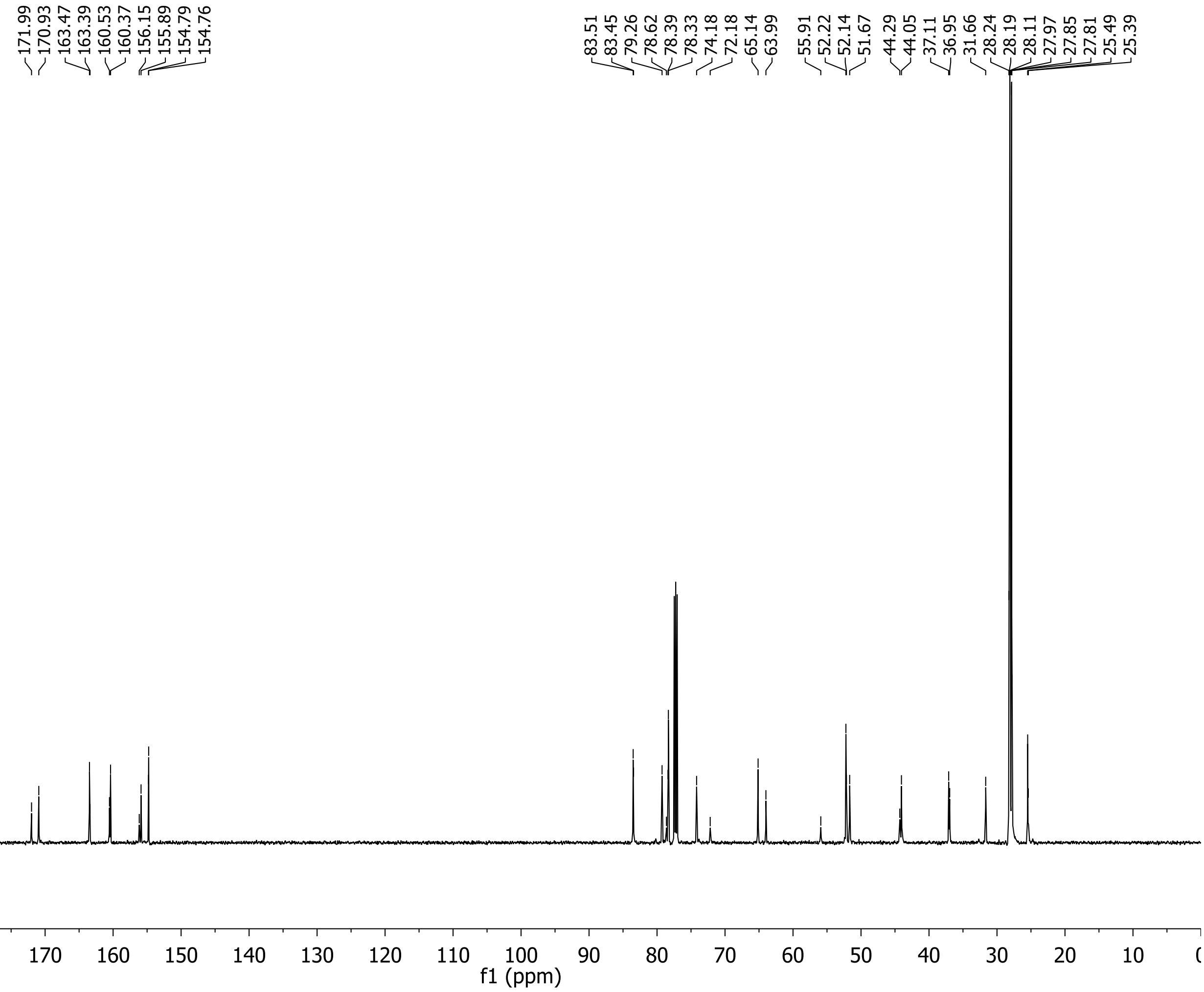

CDCl$_3$, 600 MHz

27

DEPT-135

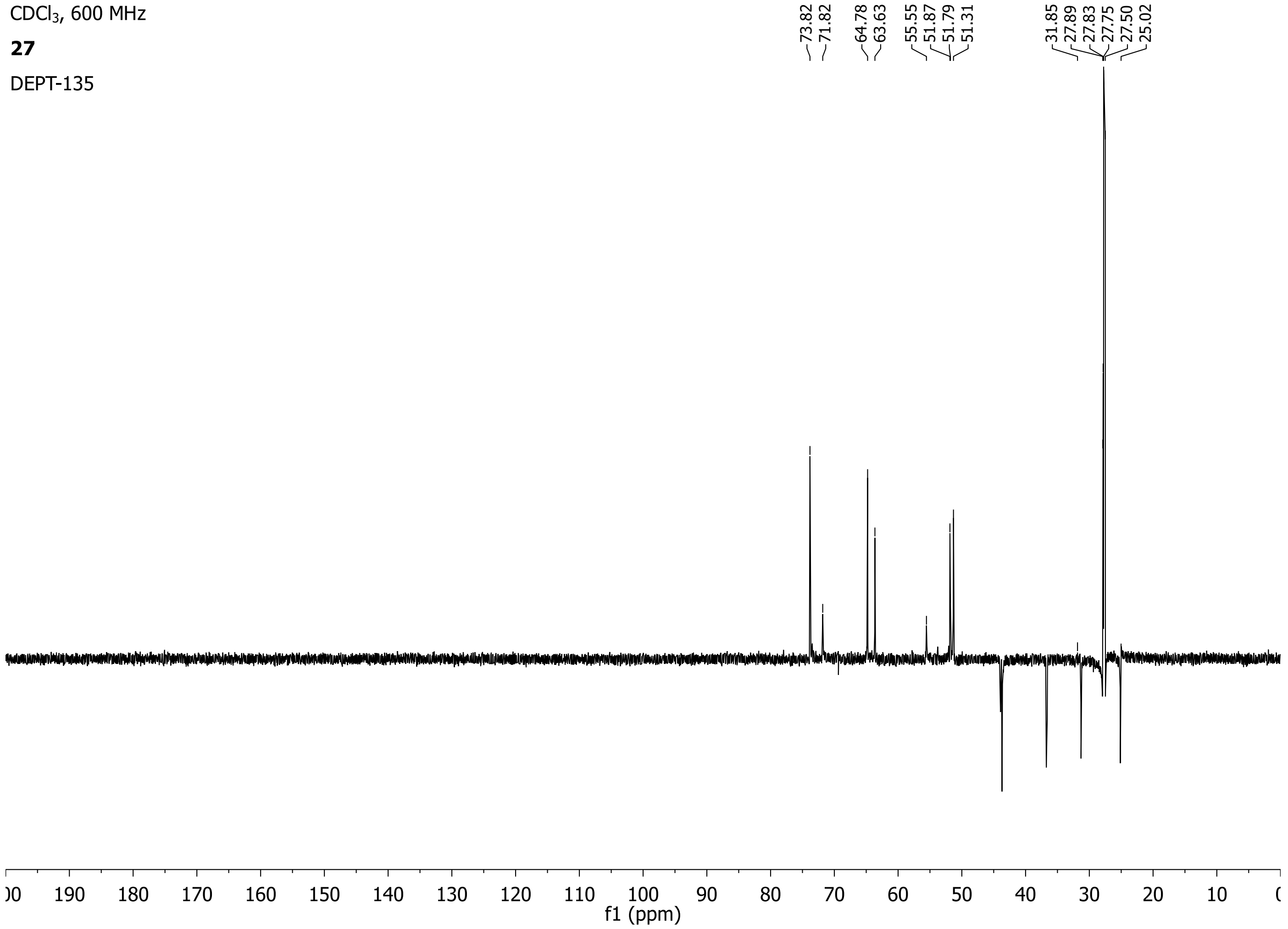

CDCl$_3$, 600 MHz

27

COSY

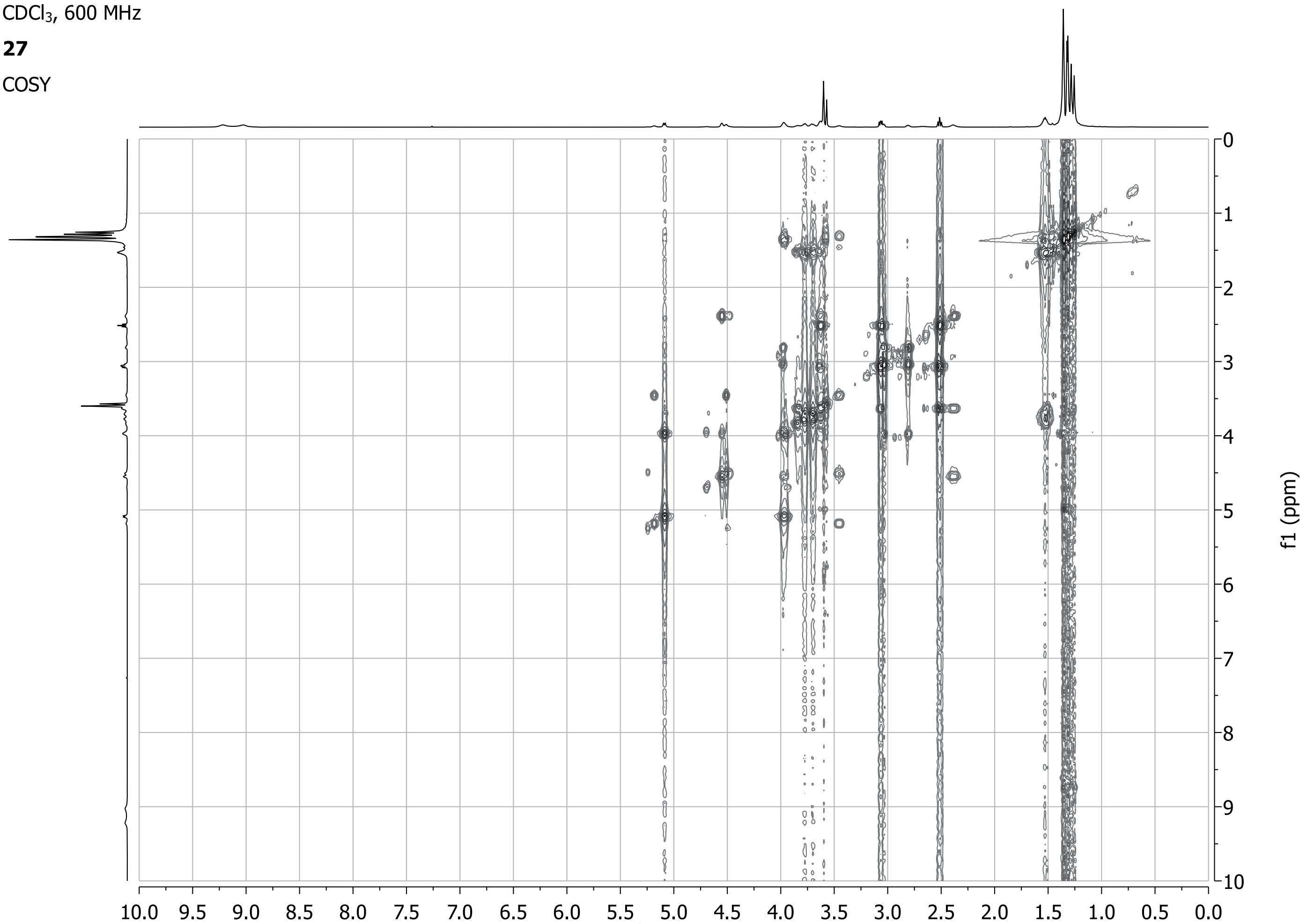

CDCl$_3$, 600 MHz

27

HMQC

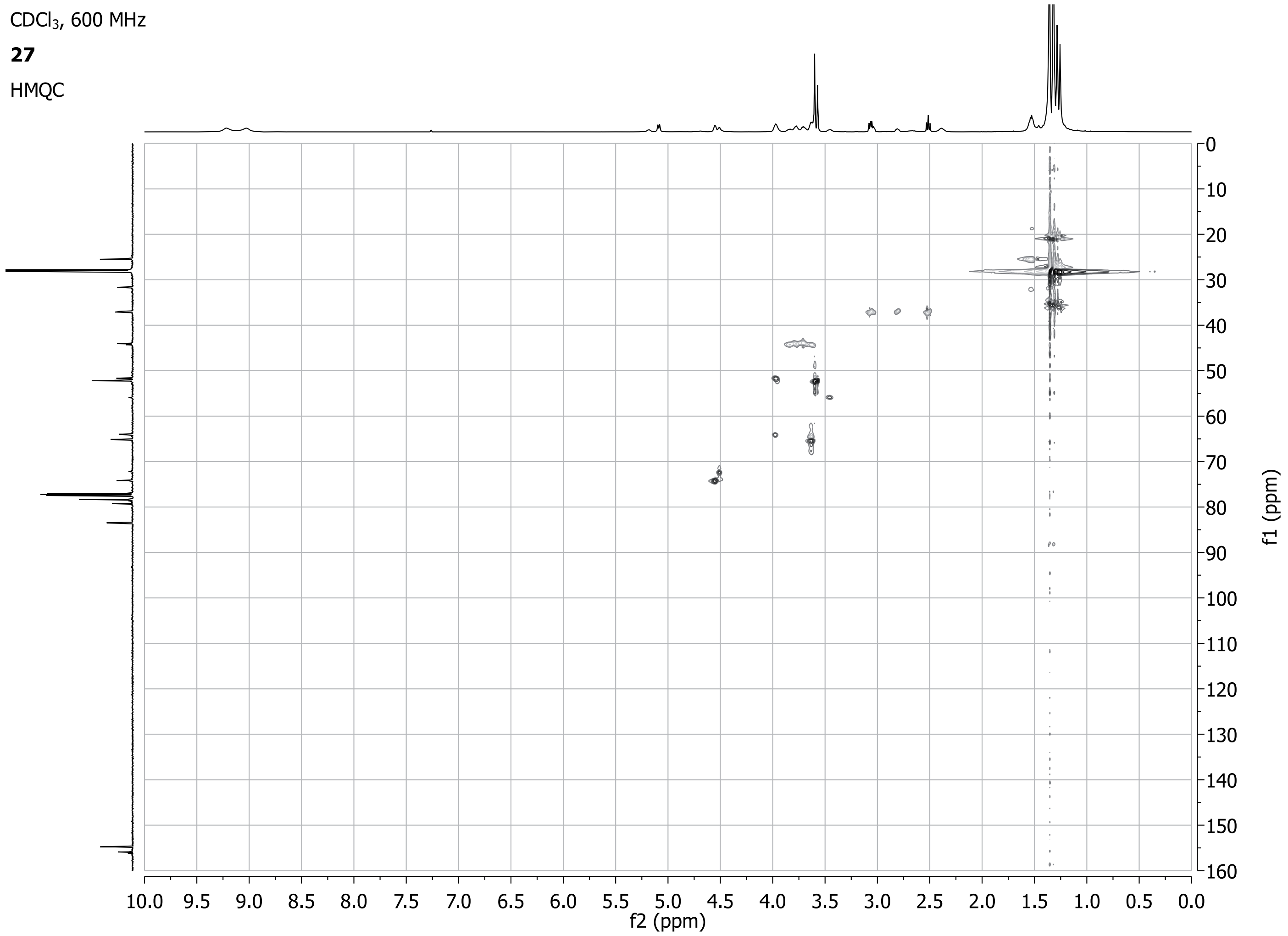

CDCl$_3$, 600 MHz

27

HMBC

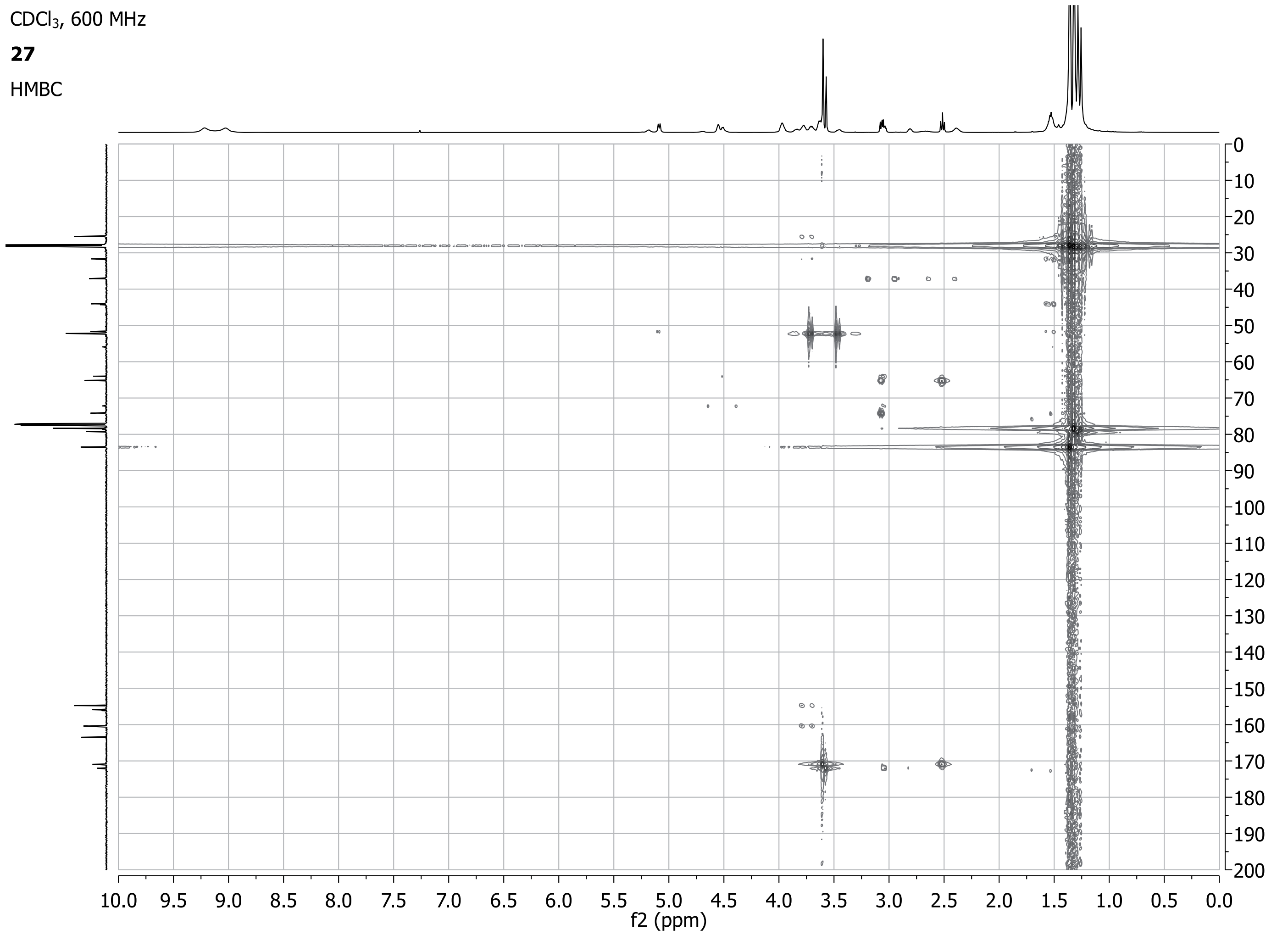

CDCl$_3$, 600 MHz

4b

^{1}H NMR

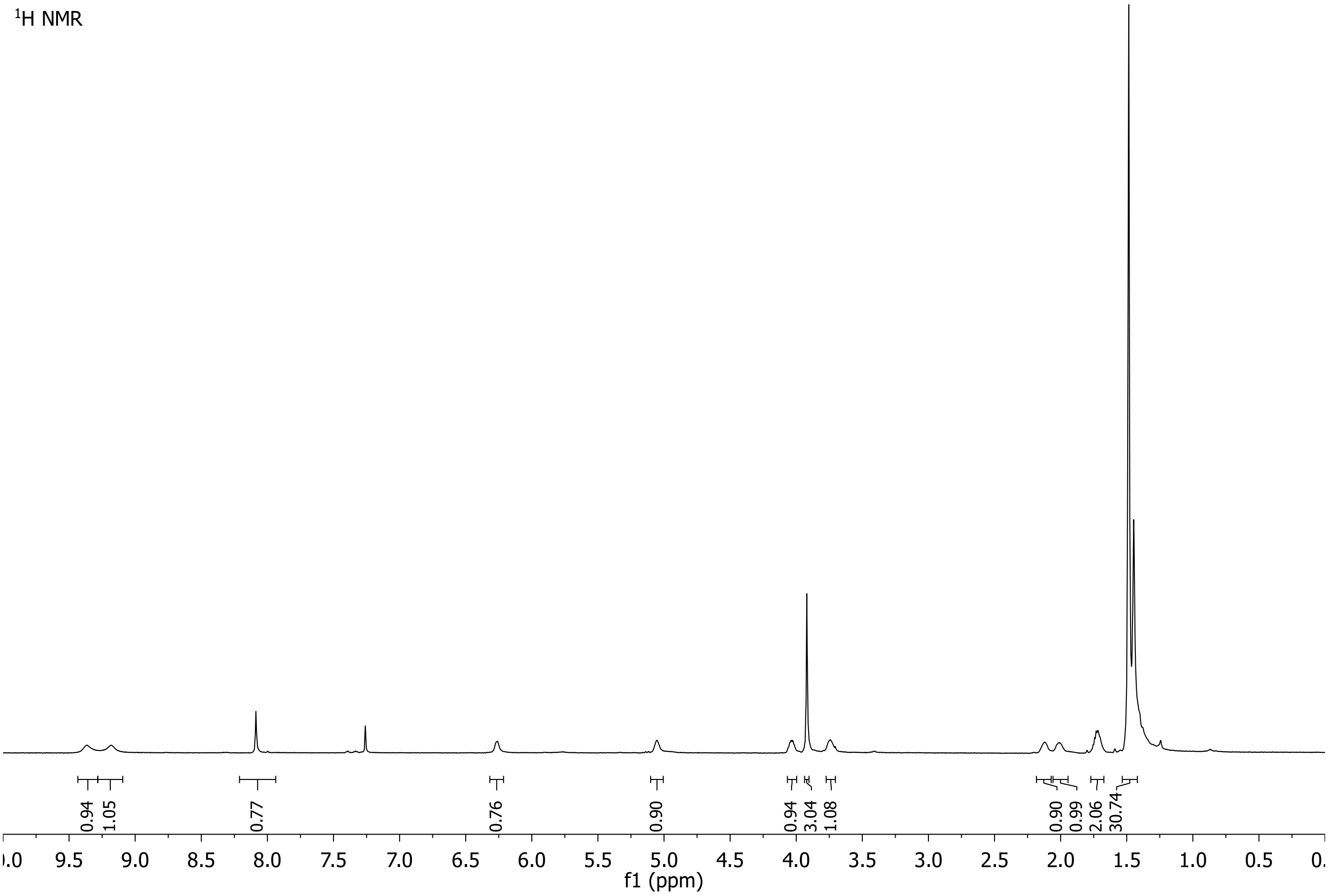

CDCl$_3$, 600 MHz

4b

^{13}C NMR

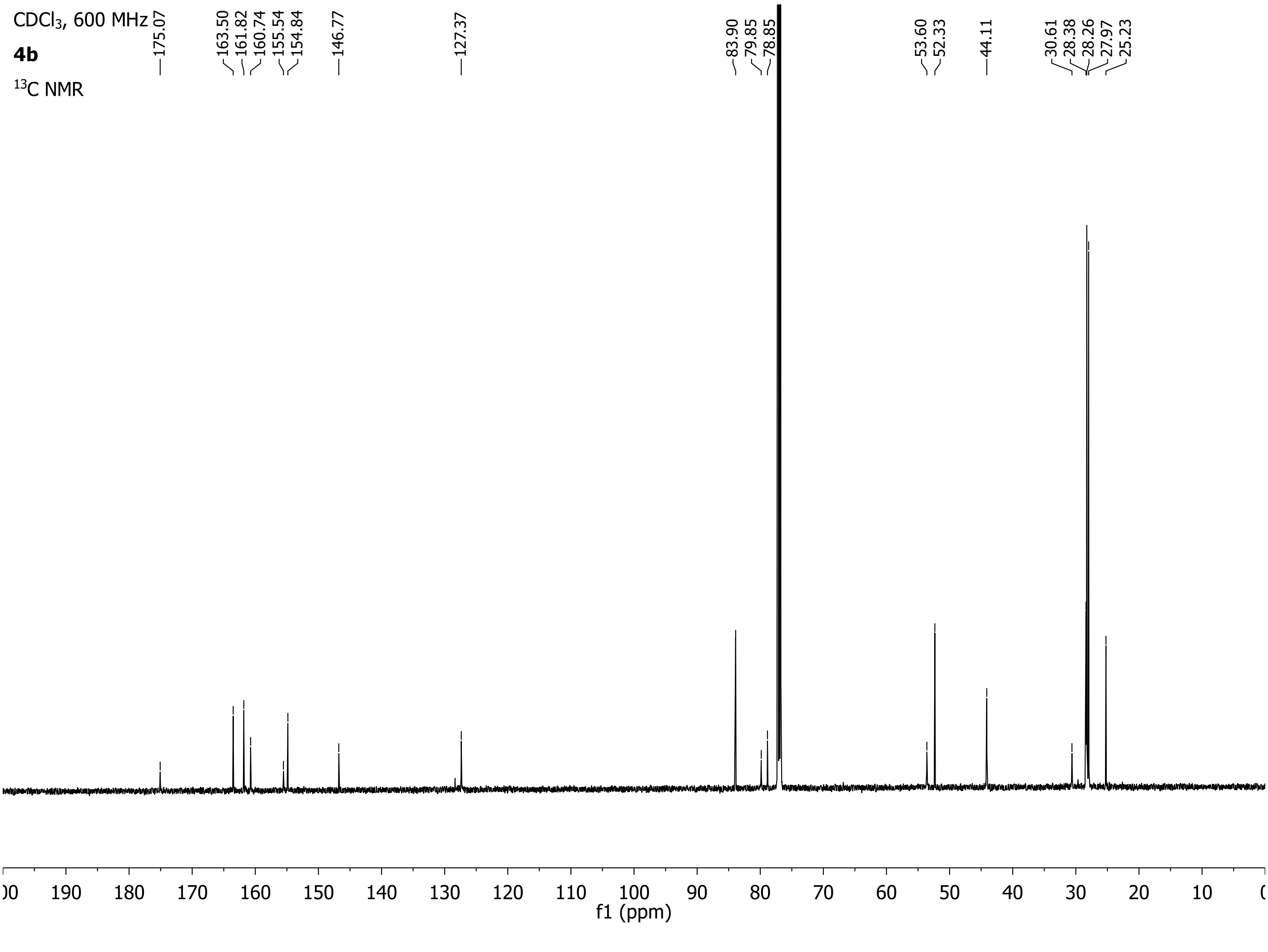

CDCl$_3$, 600 MHz

4b

DEPT-135

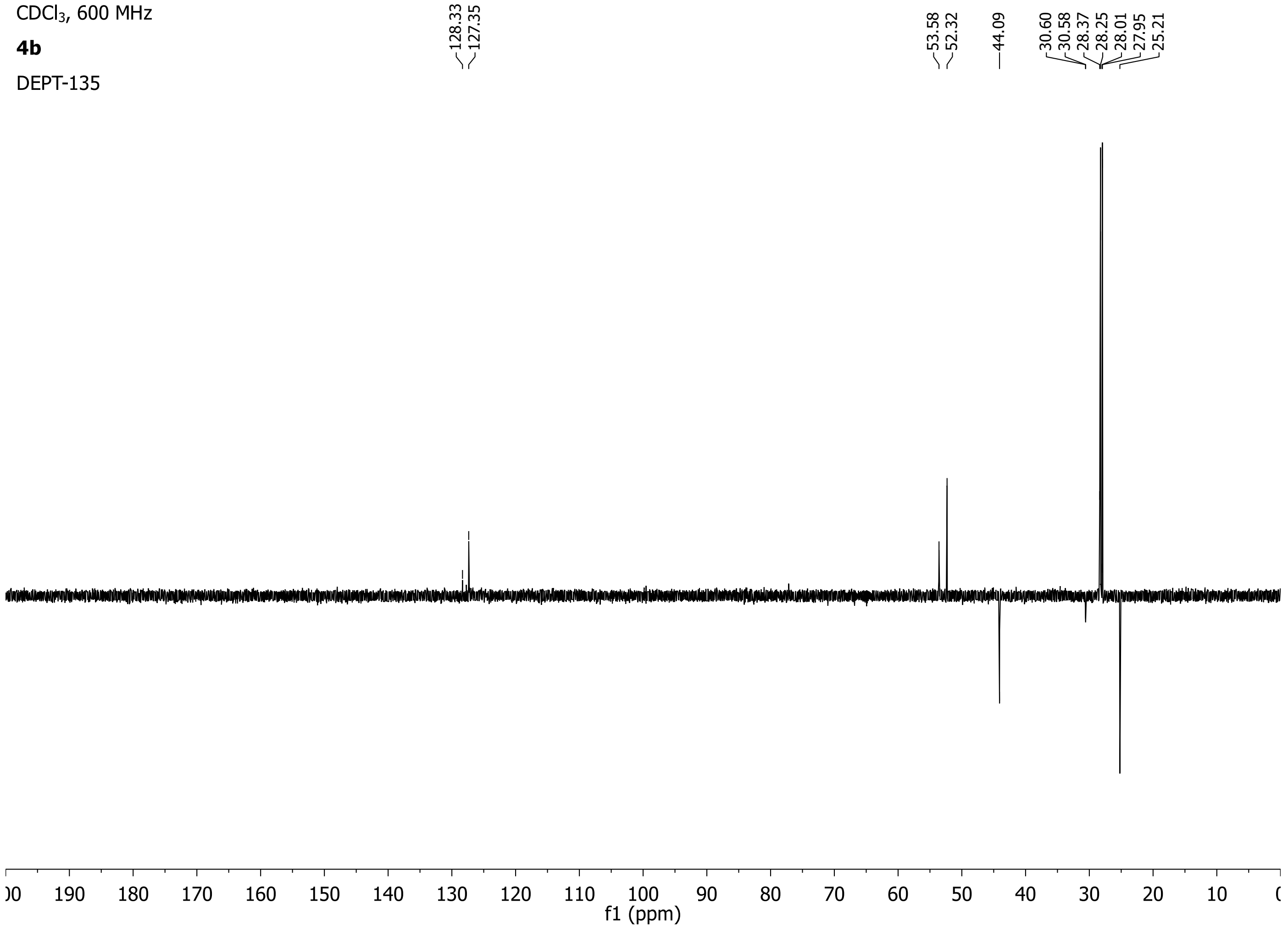

CDCl$_3$, 600 MHz

4b

COSY

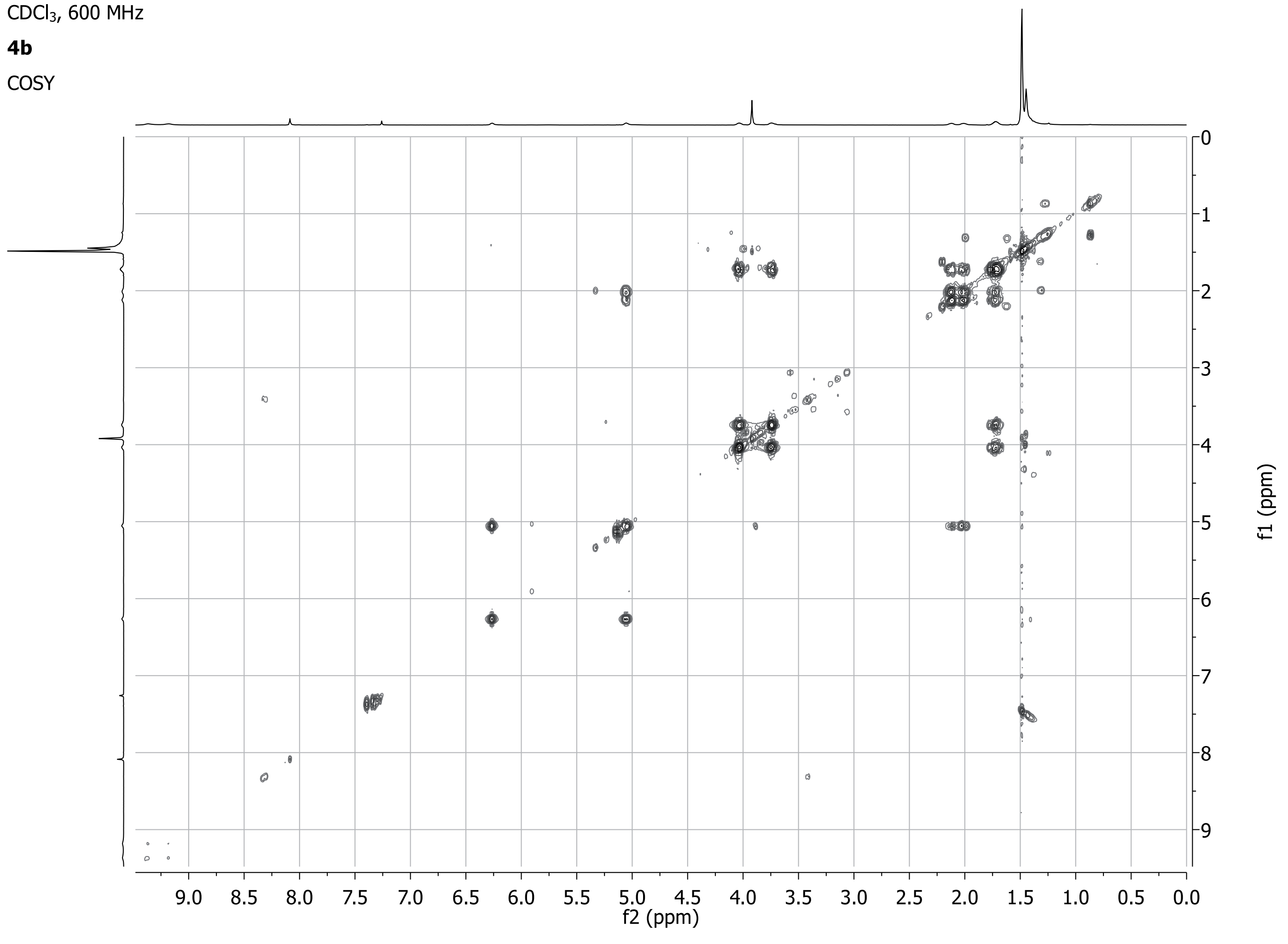

CDCl$_3$, 600 MHz

4b

HMQC

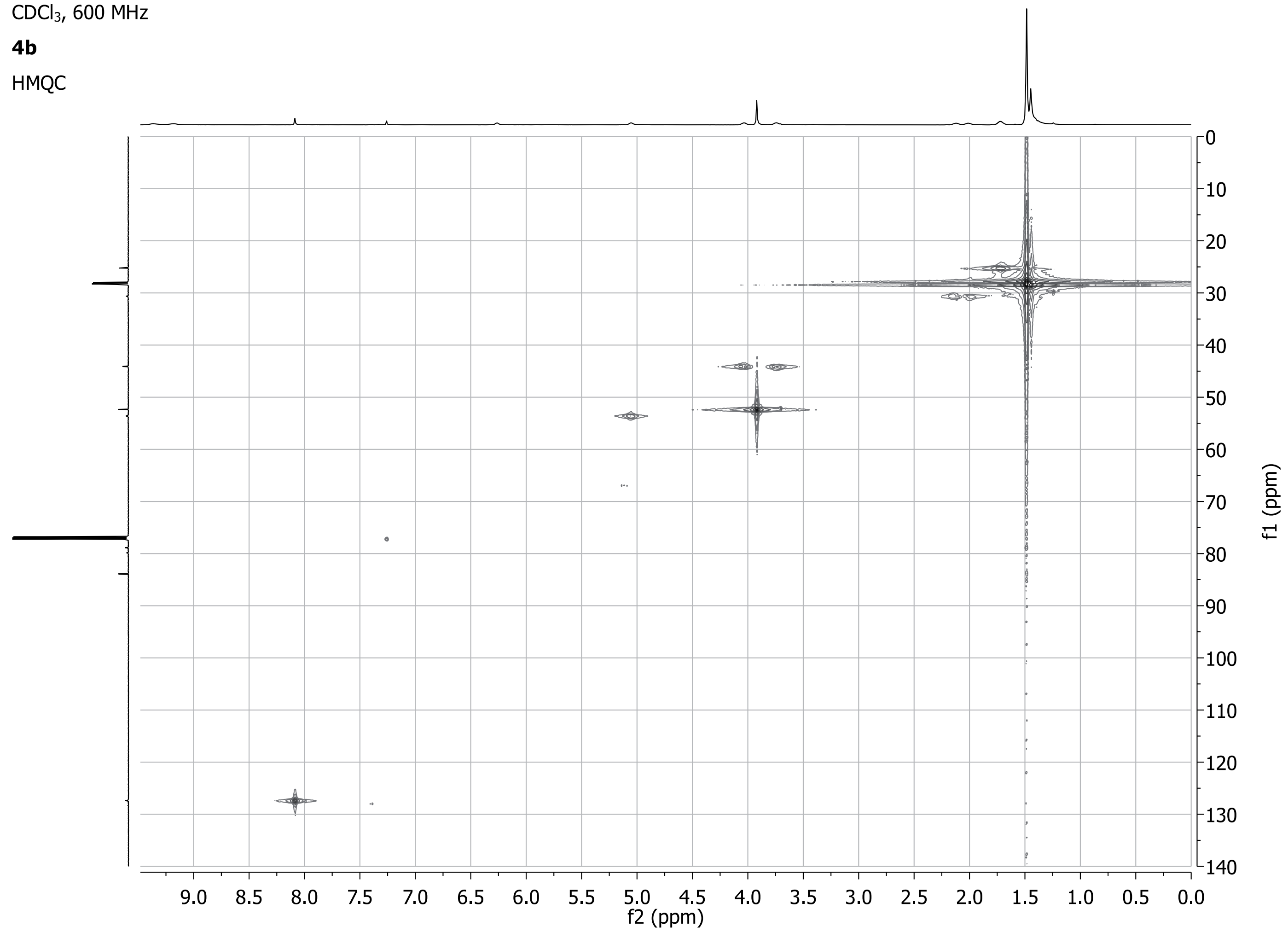

CDCl$_3$, 600 MHz

4b

HMBC

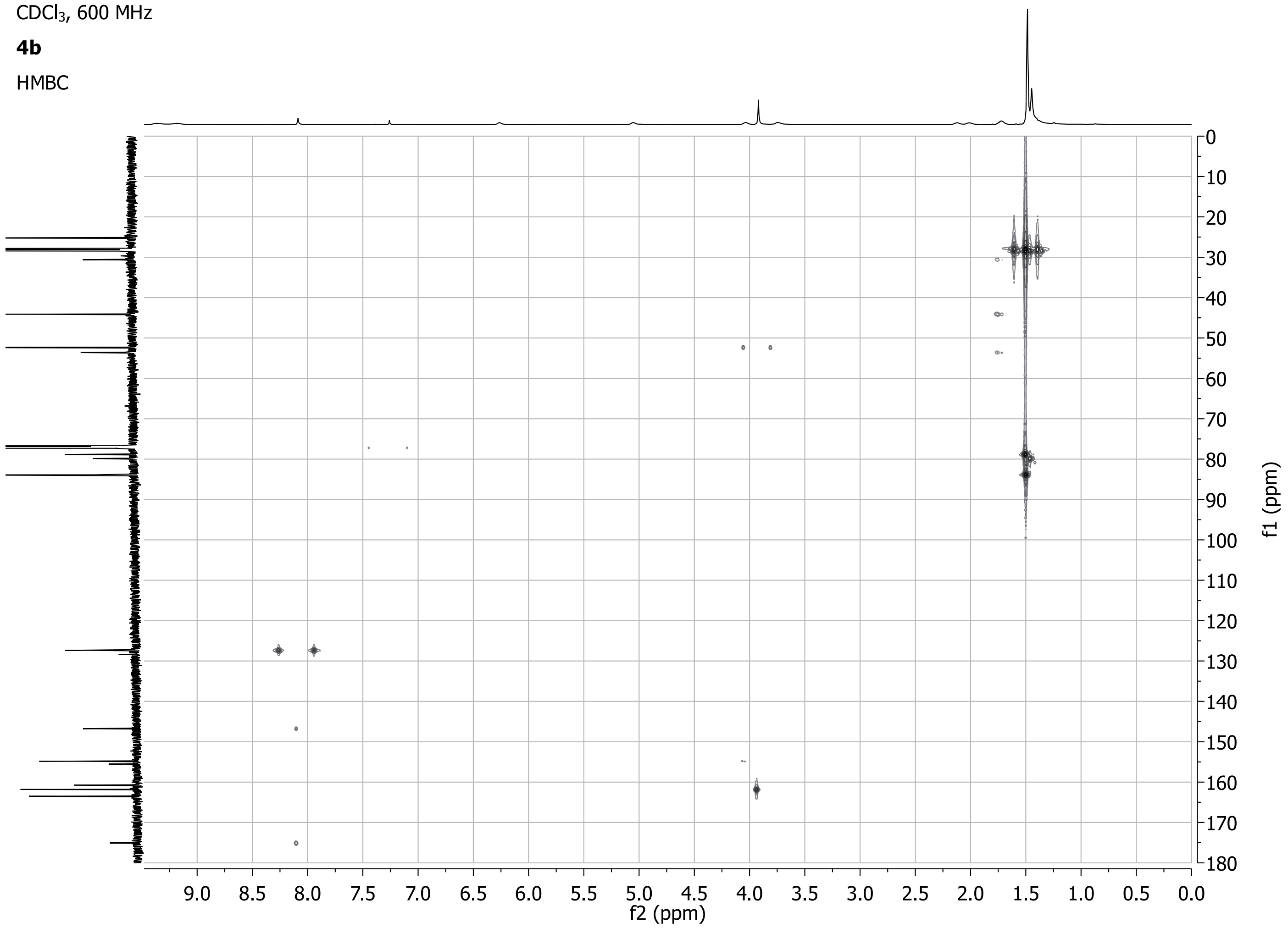

CDCl3, 600 MHz

4a

^1H NMR

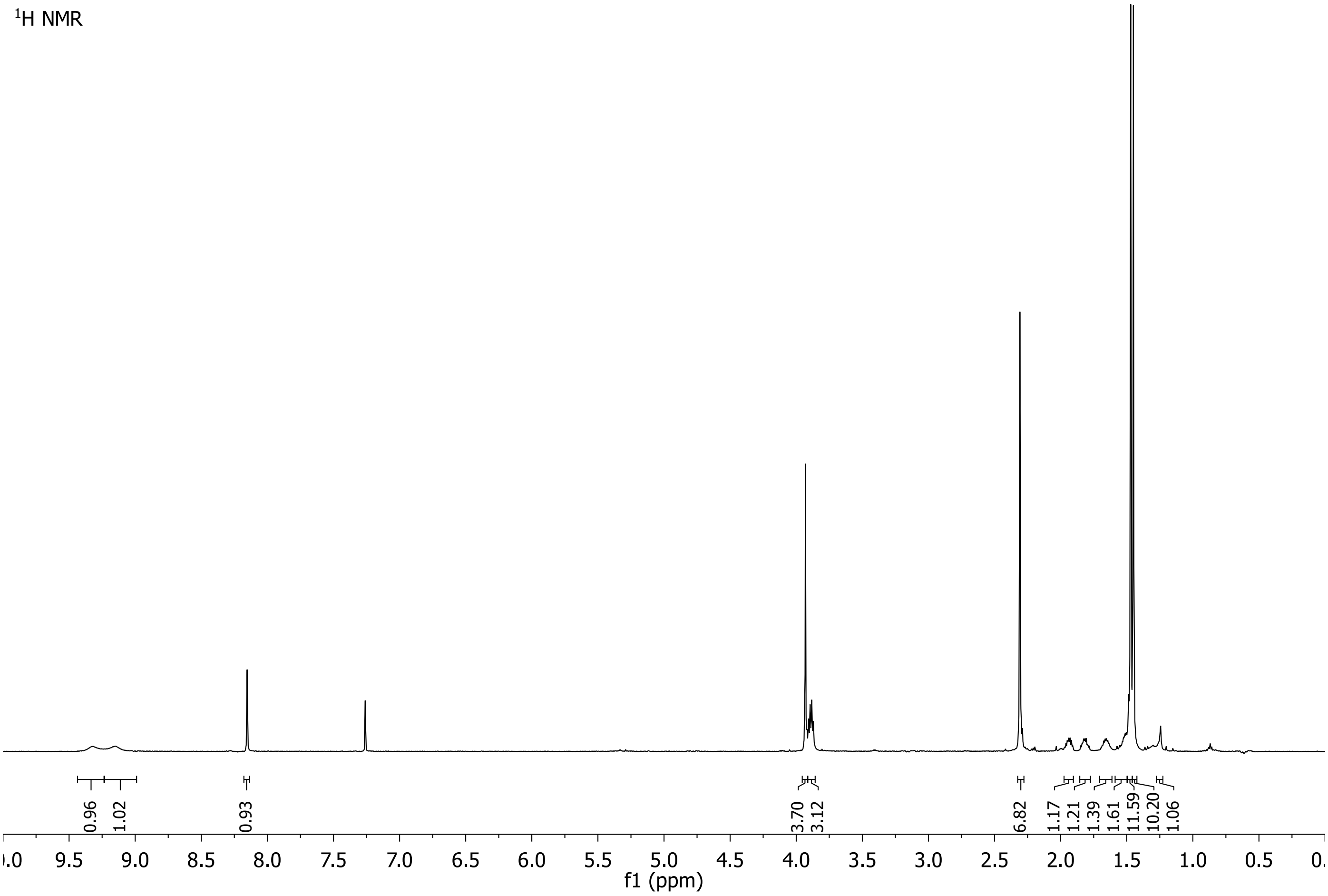

CDCl3, 600 MHz

4a

^{13}C NMR

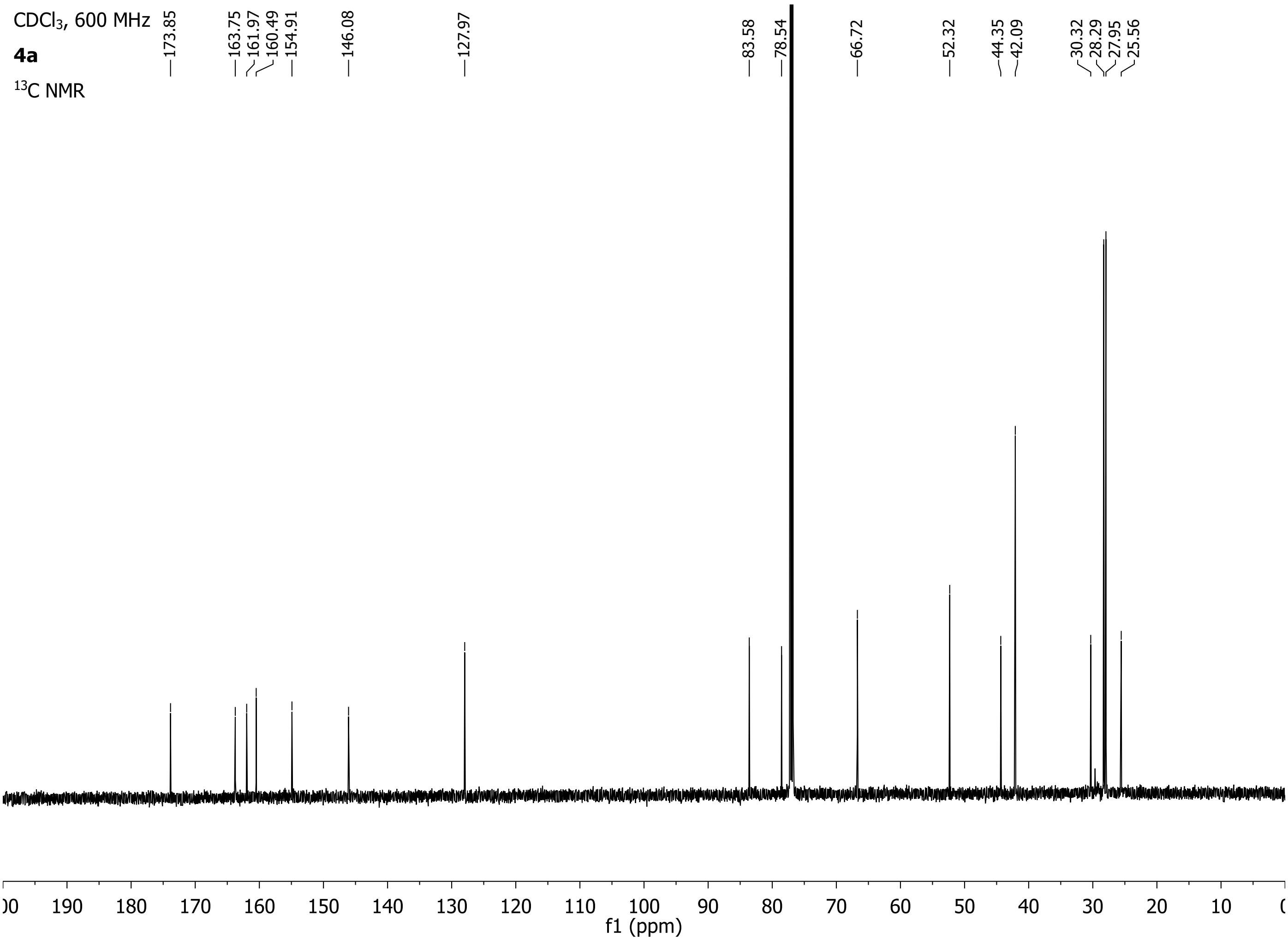

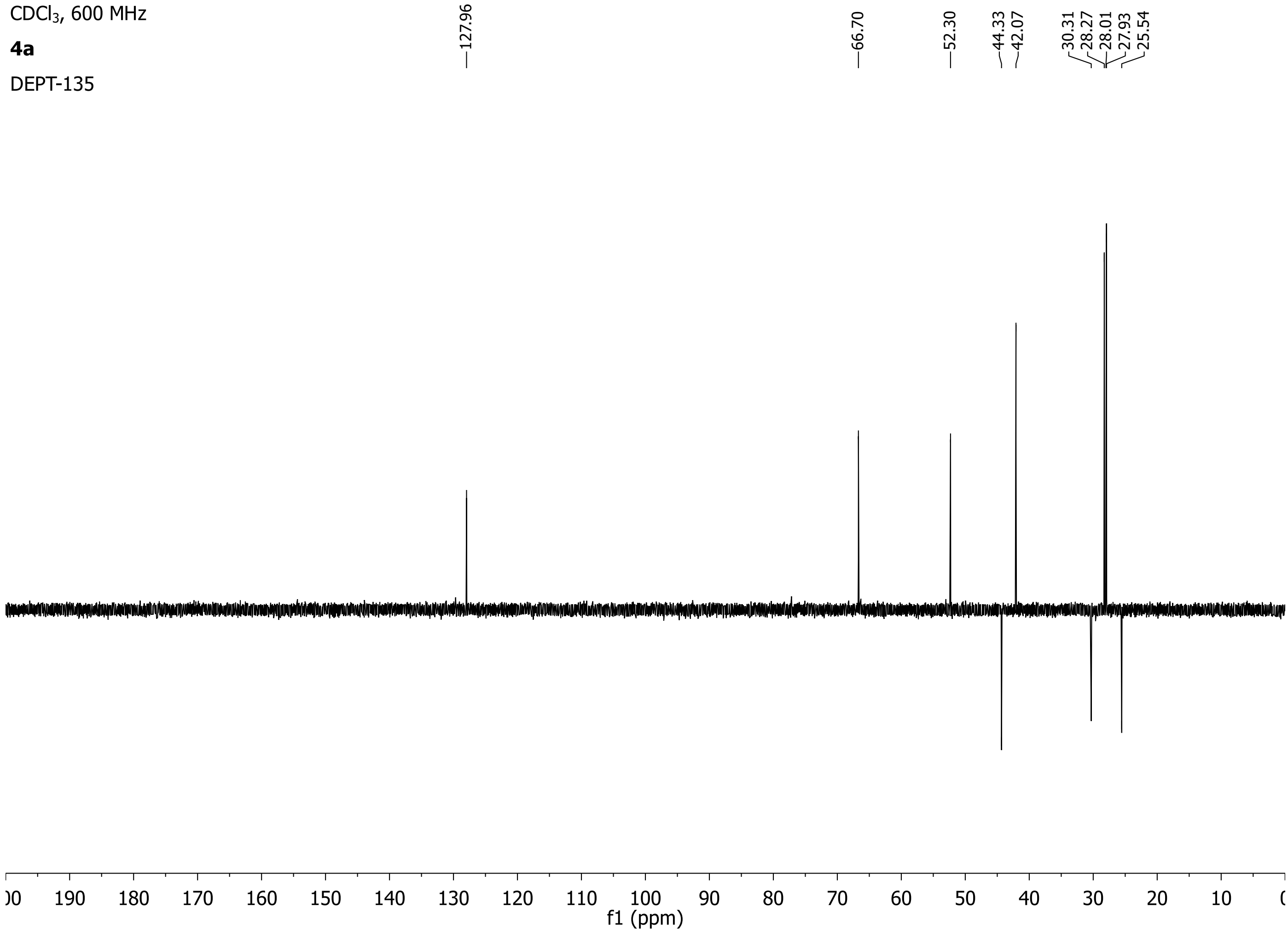
CDCl3, 600 MHz
4a
DEPT-135
127.96
66.70
52.30
44.33
42.07
30.31
28.27
28.01
27.93
25.54
f1 (ppm)

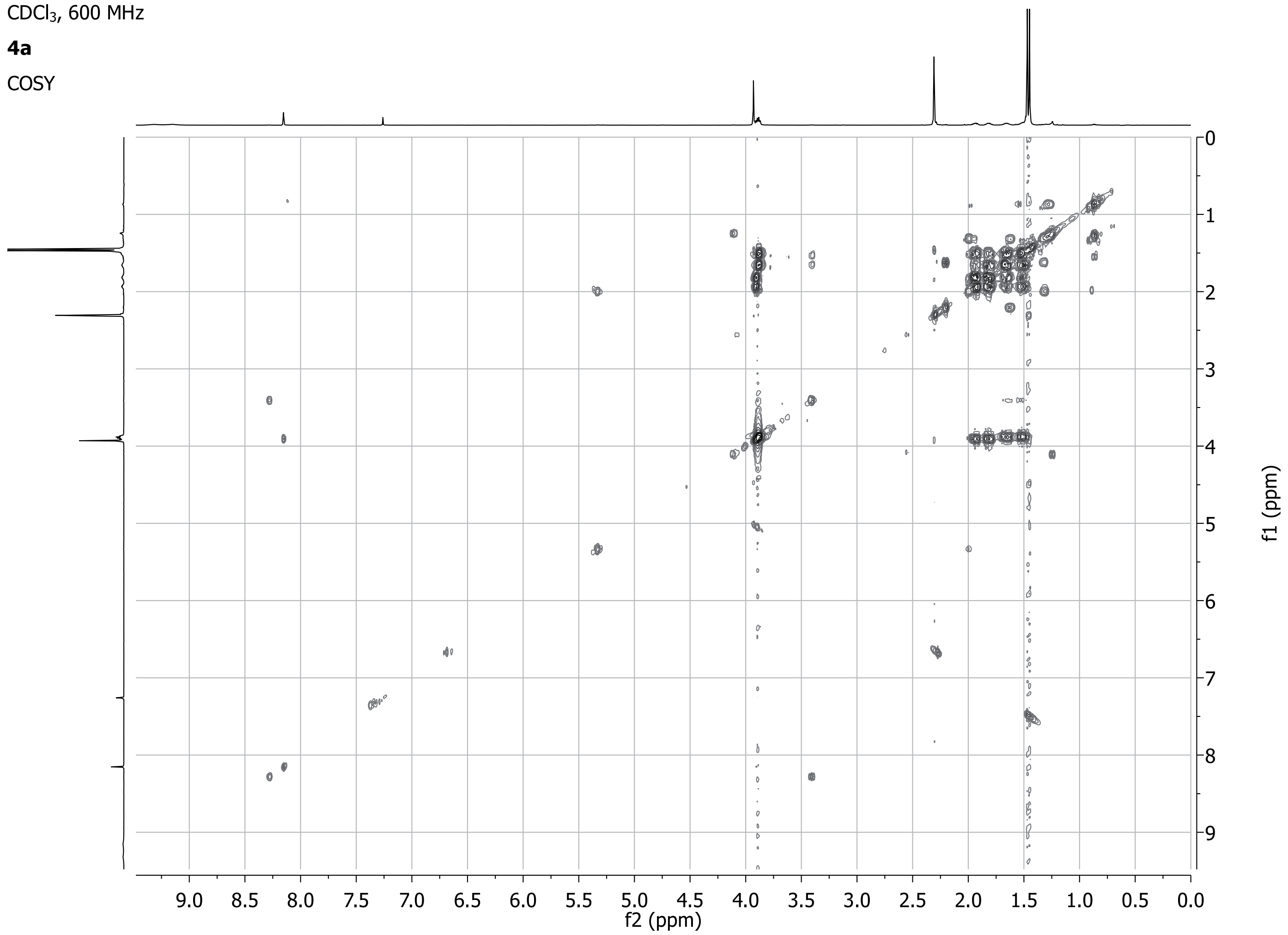
CDCl3, 600 MHz
4a
COSY
f2 (ppm)
f1 (ppm)

CDCl$_3$, 600 MHz

4a

HMQC

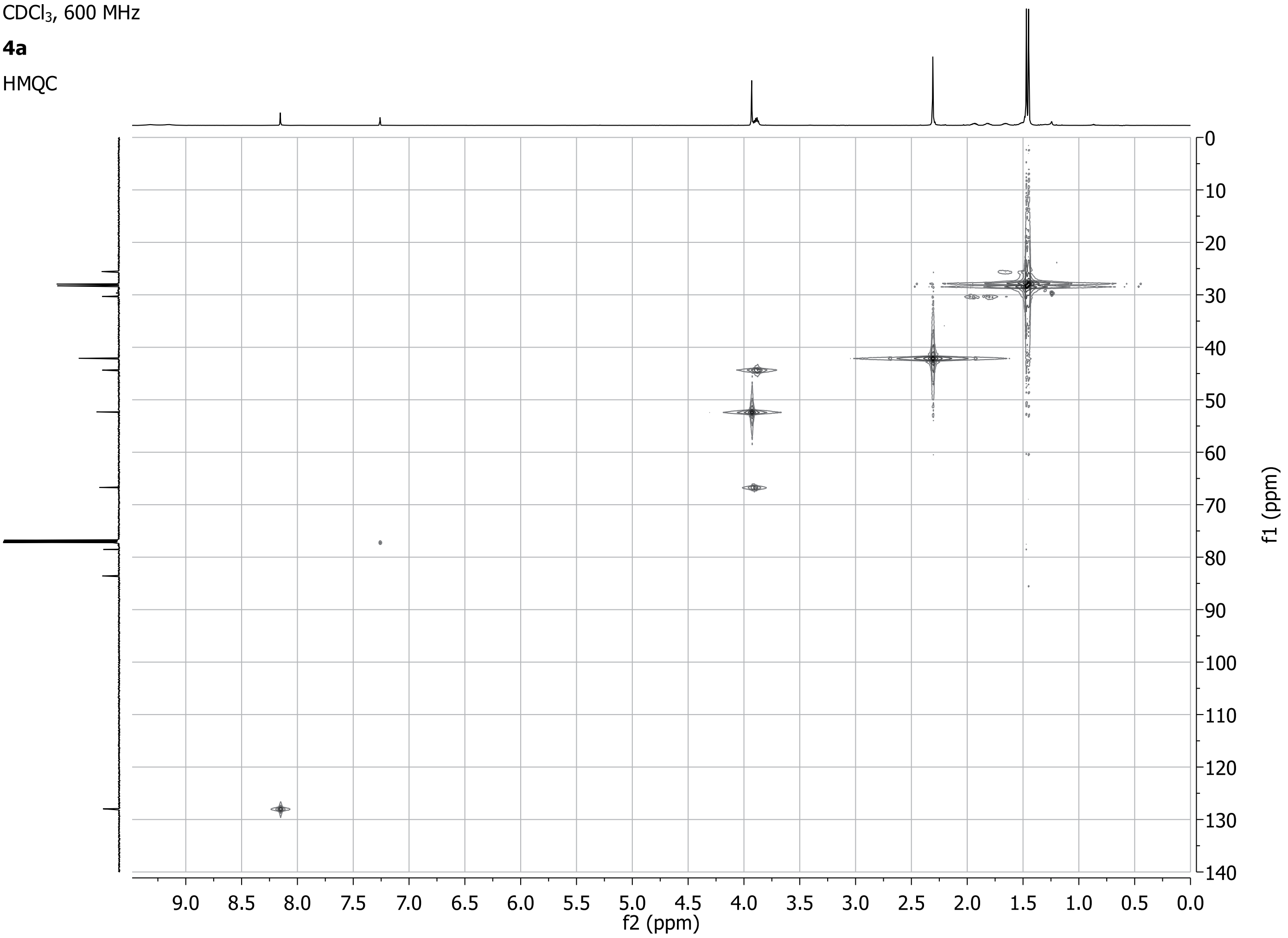

CDCl$_3$, 600 MHz

4a

HMBC

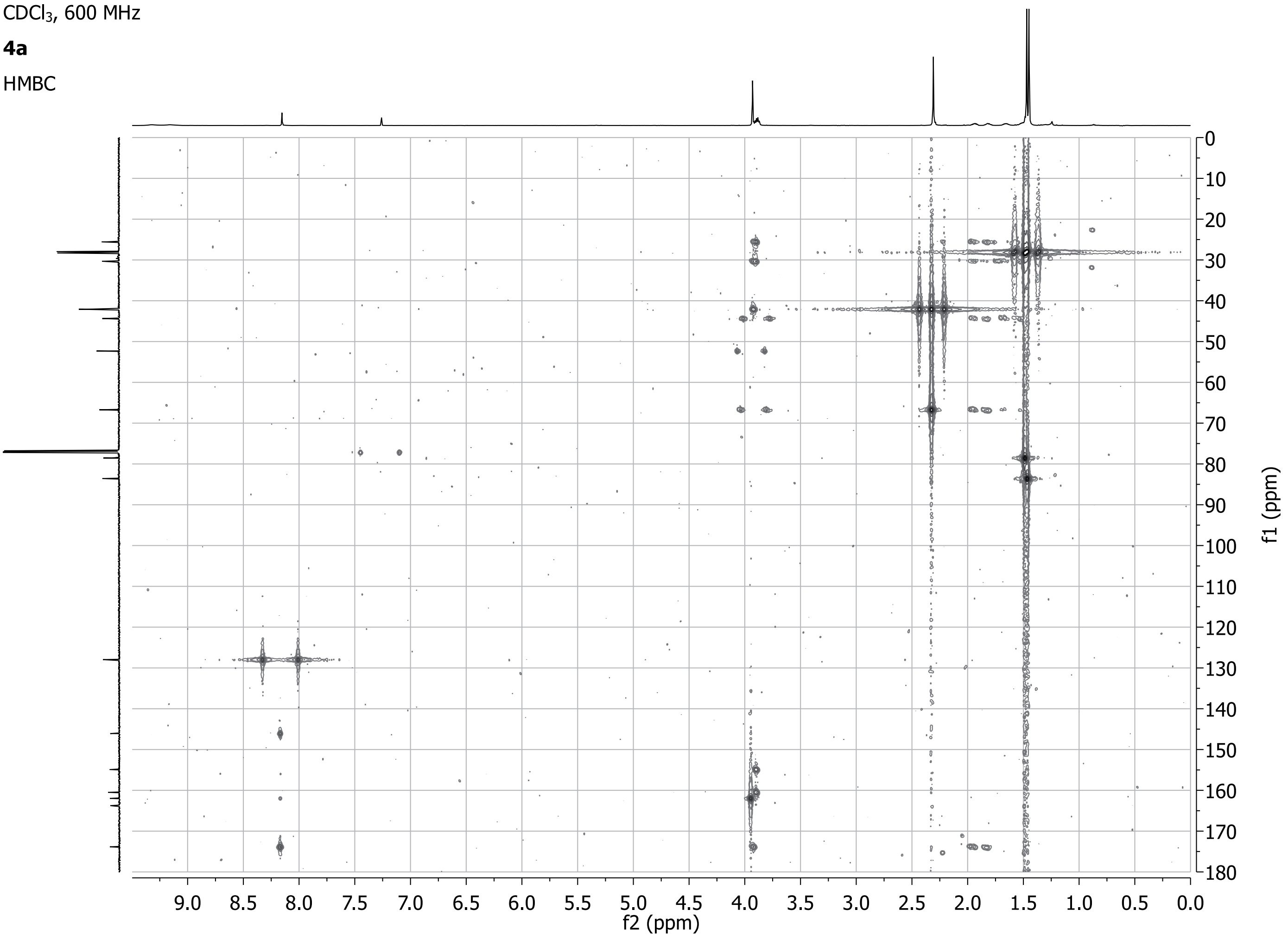

CDCl$_3$, 600 MHz

28

^{1}H NMR

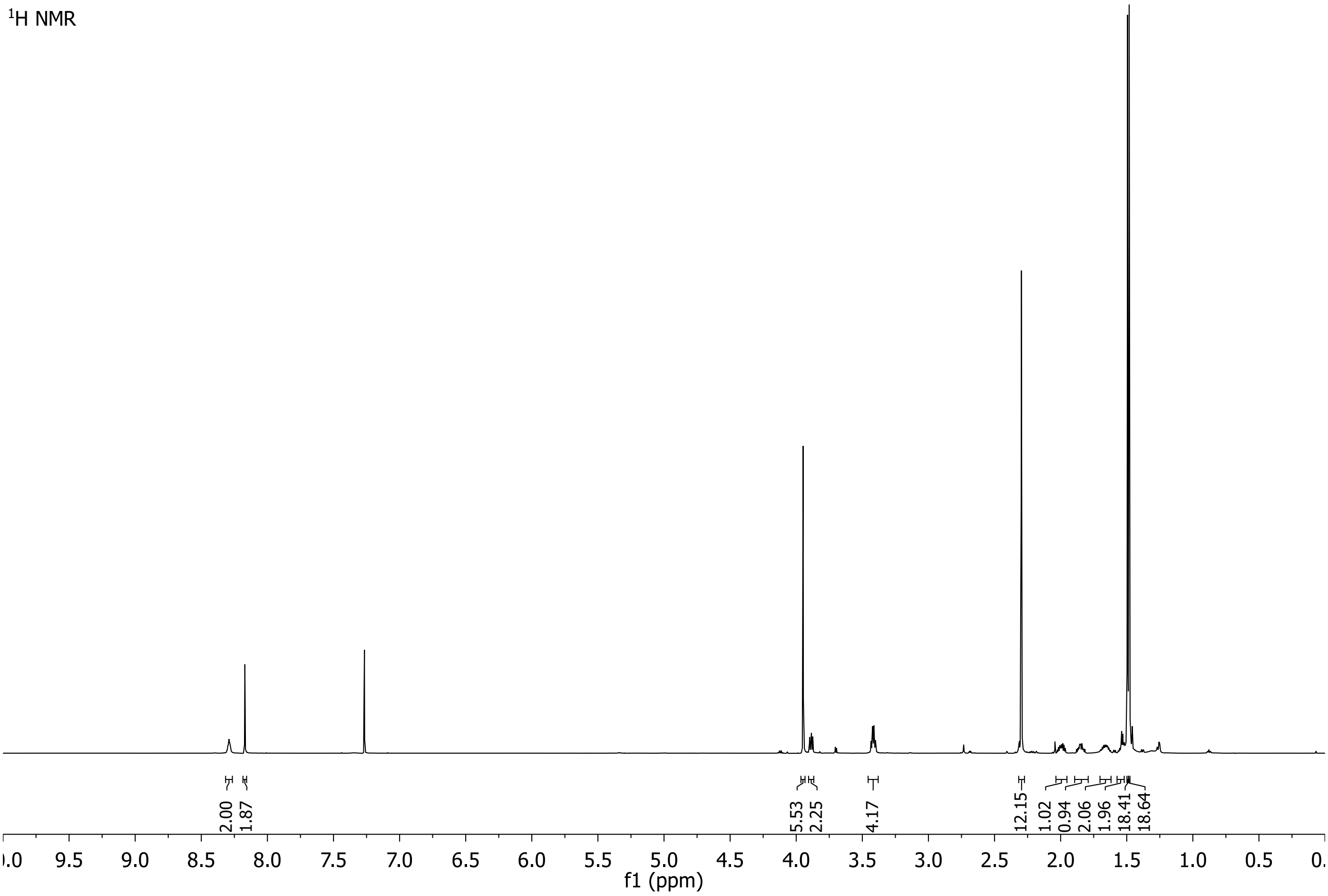

CDCl$_3$, 600 MHz

28

^{13}C NMR

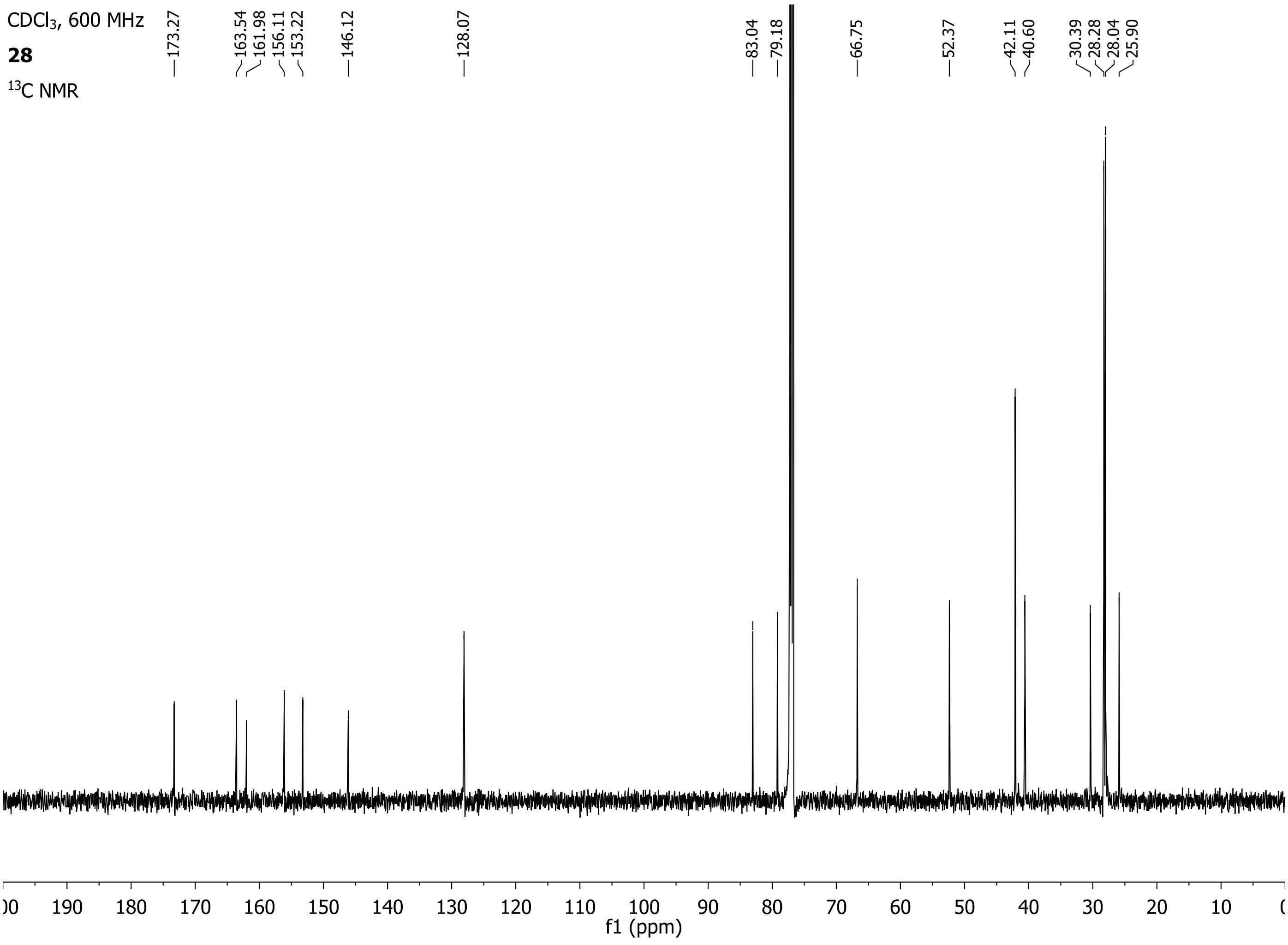

CDCl$_3$, 600 MHz

28

DEPT-135

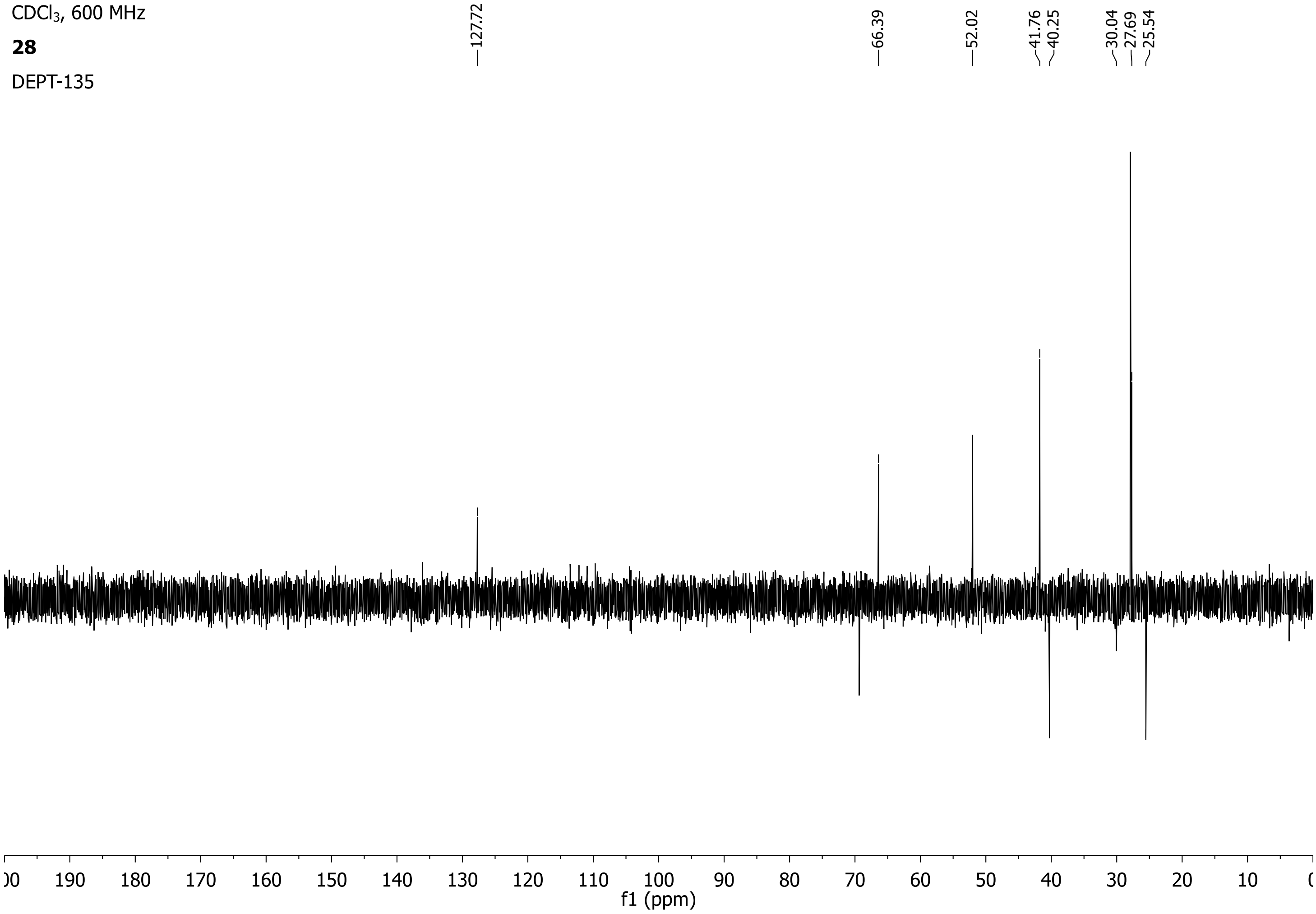

CDCl$_3$, 600 MHz

28

COSY

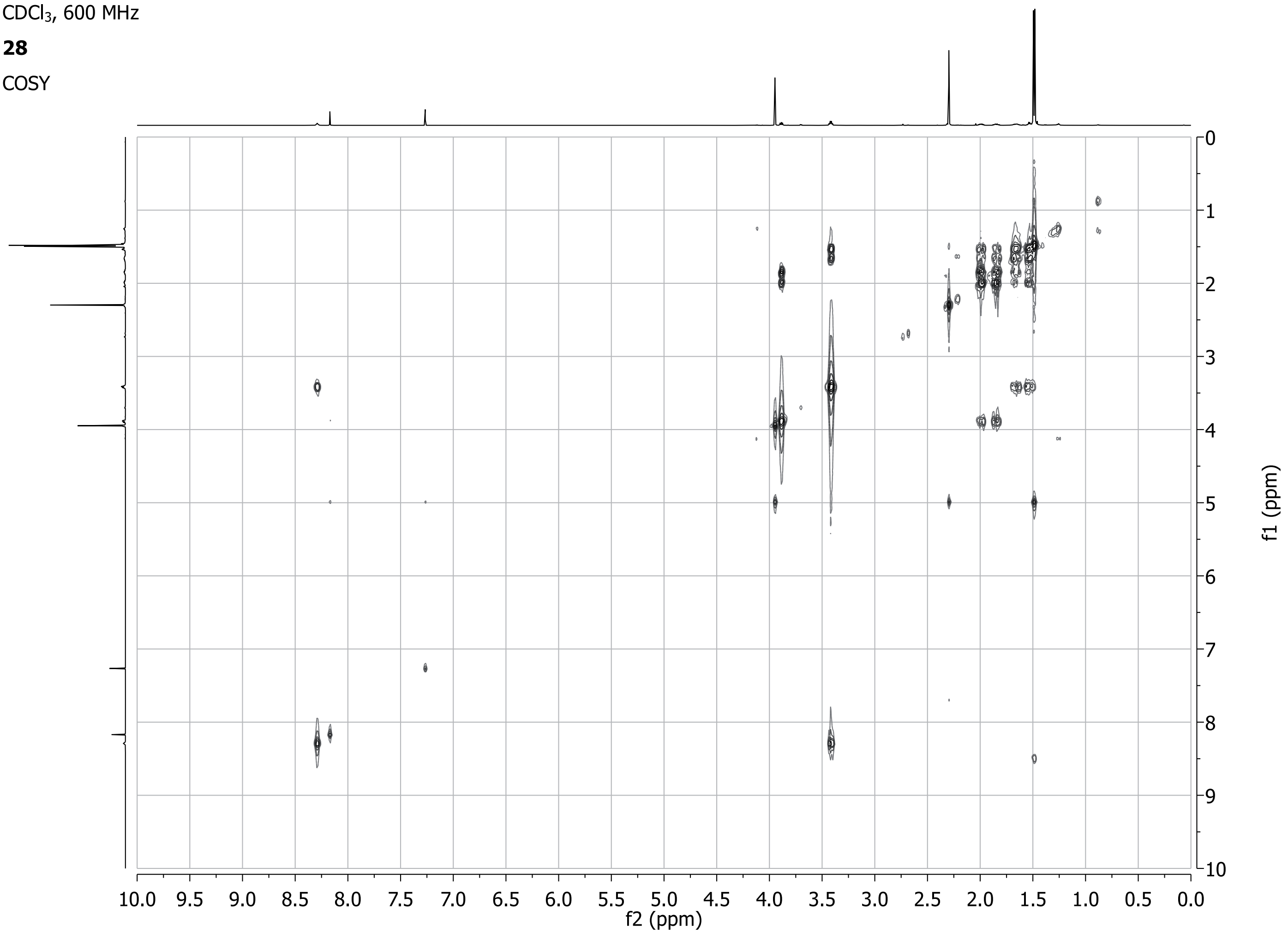

CDCl$_3$, 600 MHz

28

HMQC

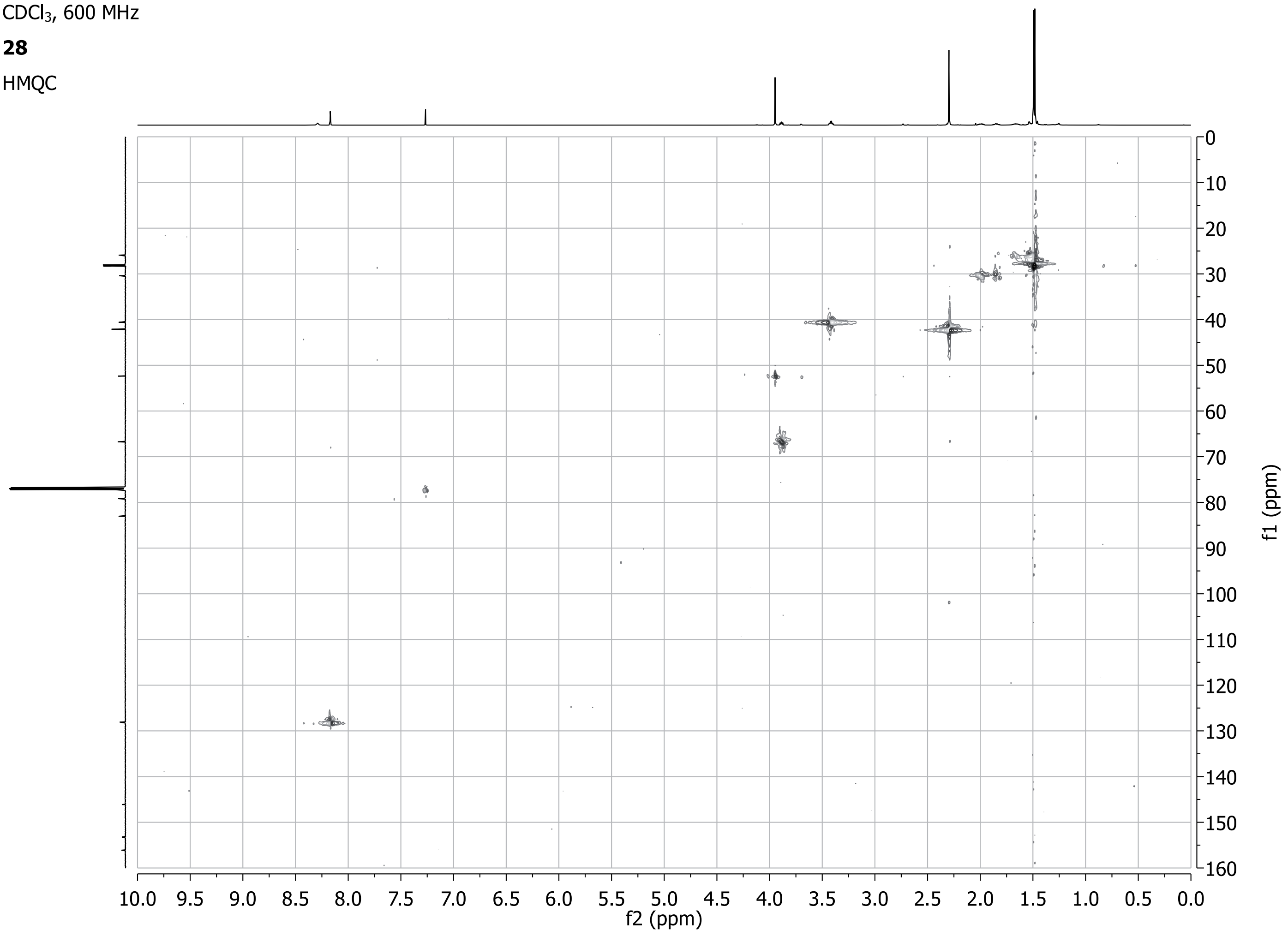

CDCl$_3$, 600 MHz

28

HMBC

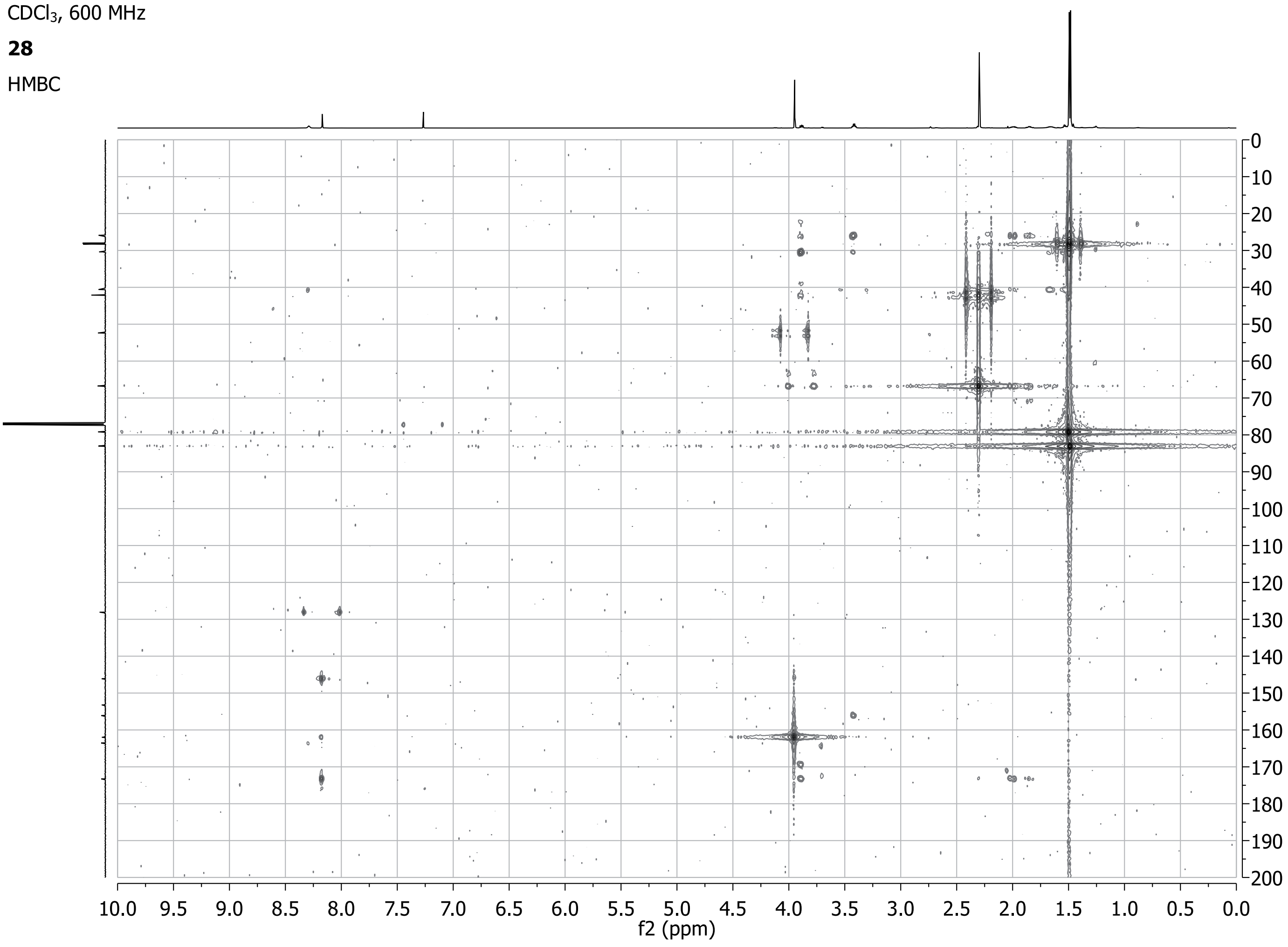

CDCl$_3$, 600 MHz

29

^{1}H NMR

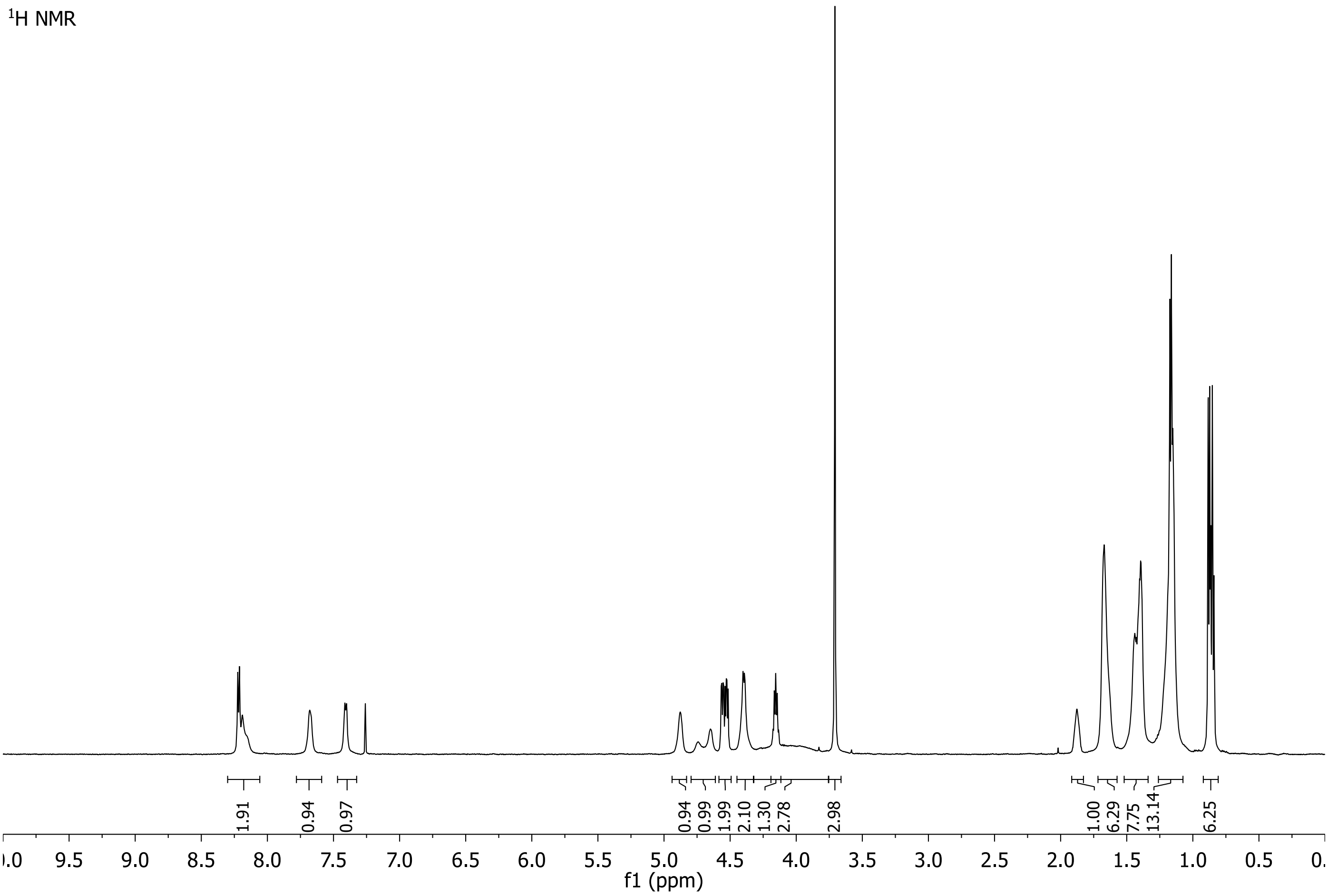

CDCl$_3$, 600 MHz

29

^{13}C NMR

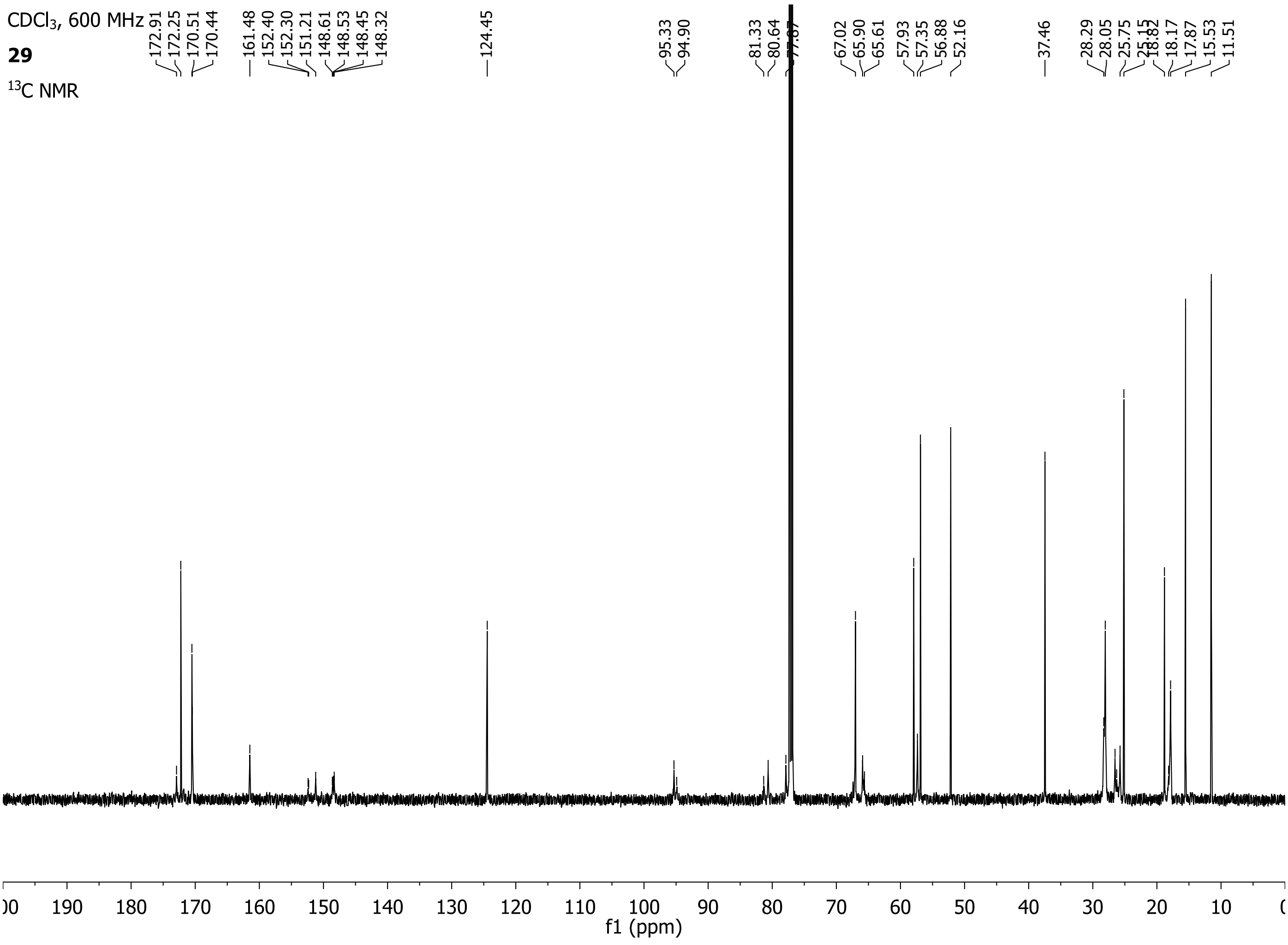

CDCl3, 600 MHz

29

DEPT-135

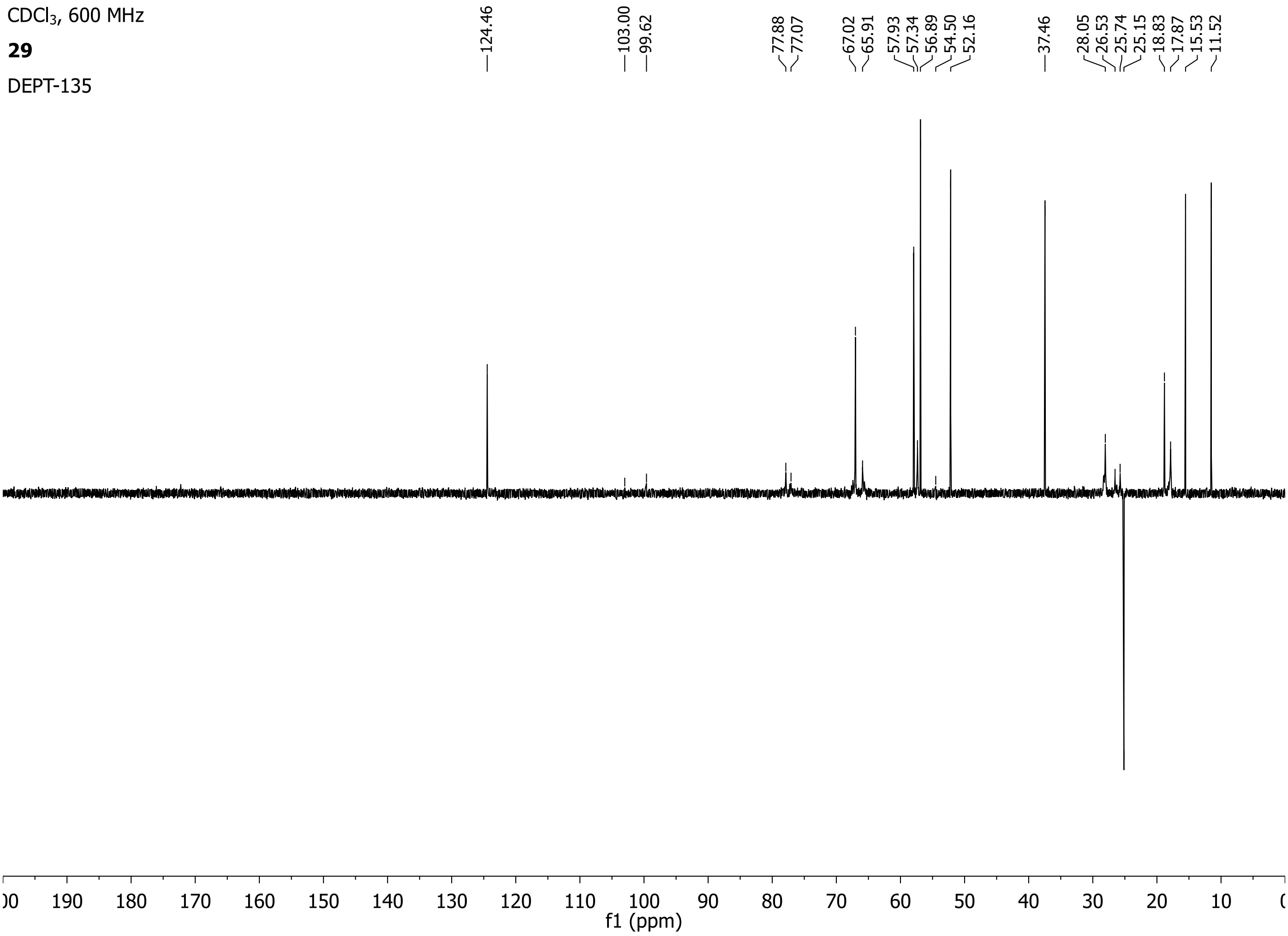

CDCl3, 600 MHz

29

COSY

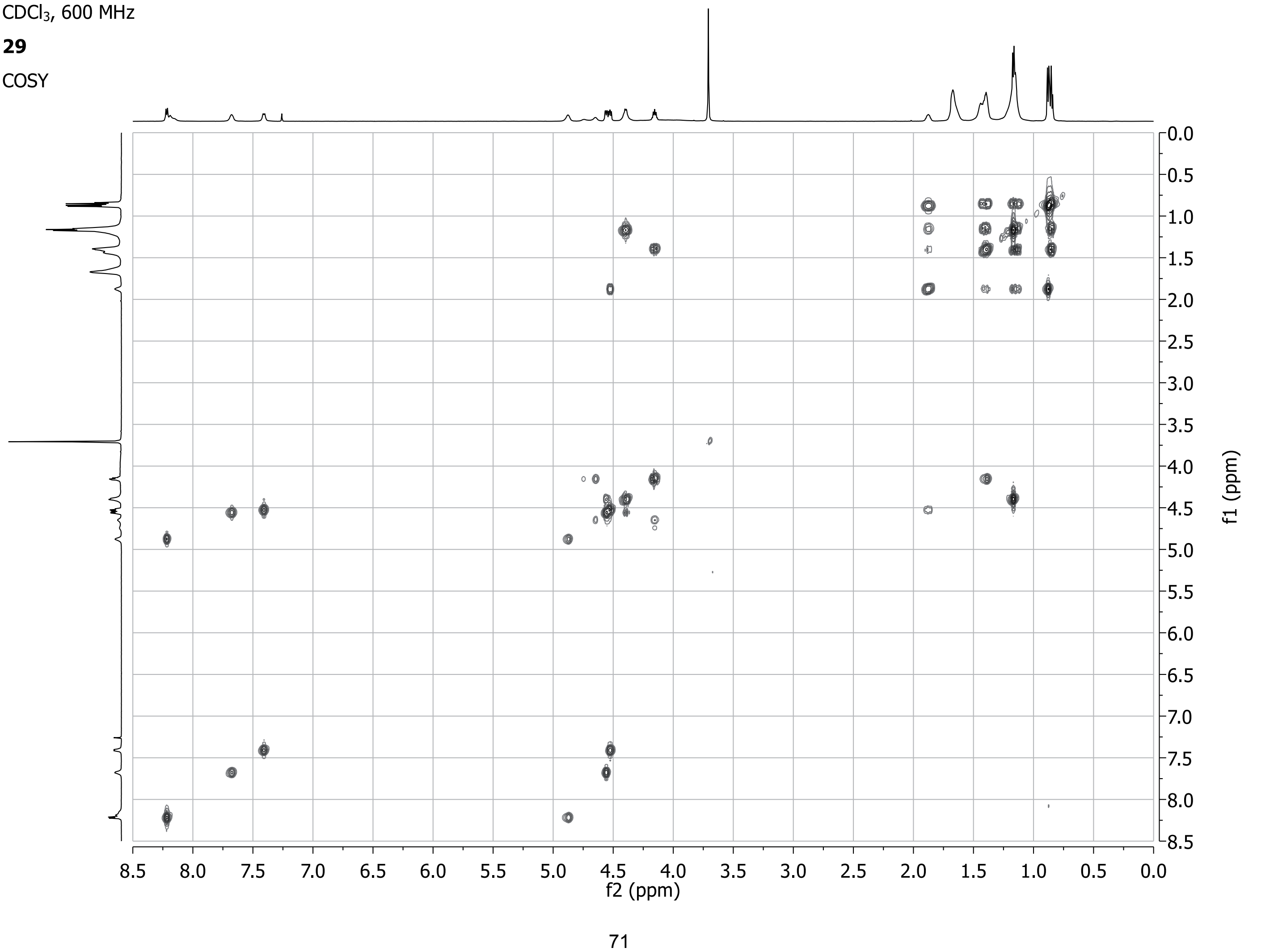

CDCl3, 600 MHz

29

HMQC

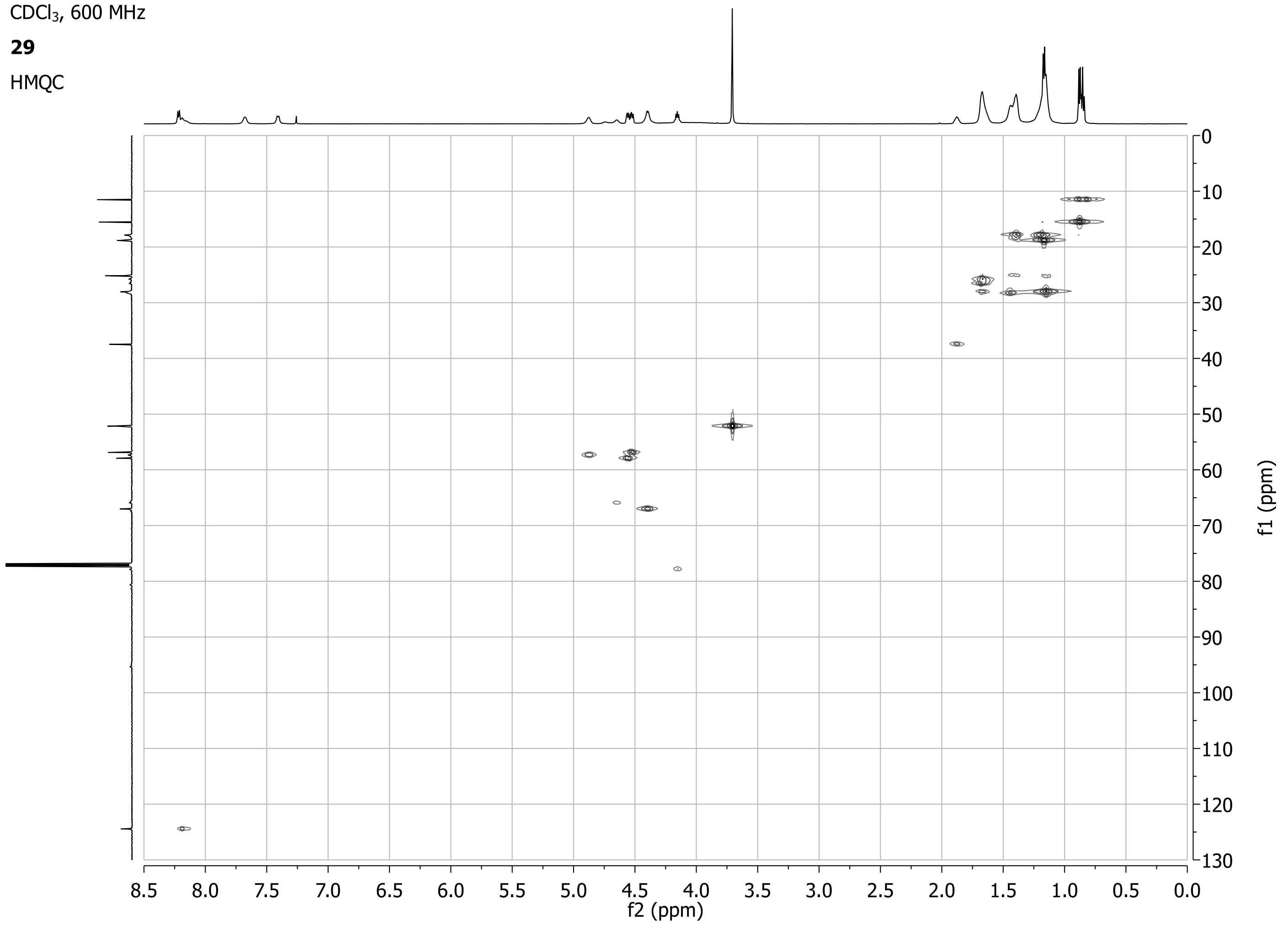

CDCl3, 600 MHz

29

HMBC

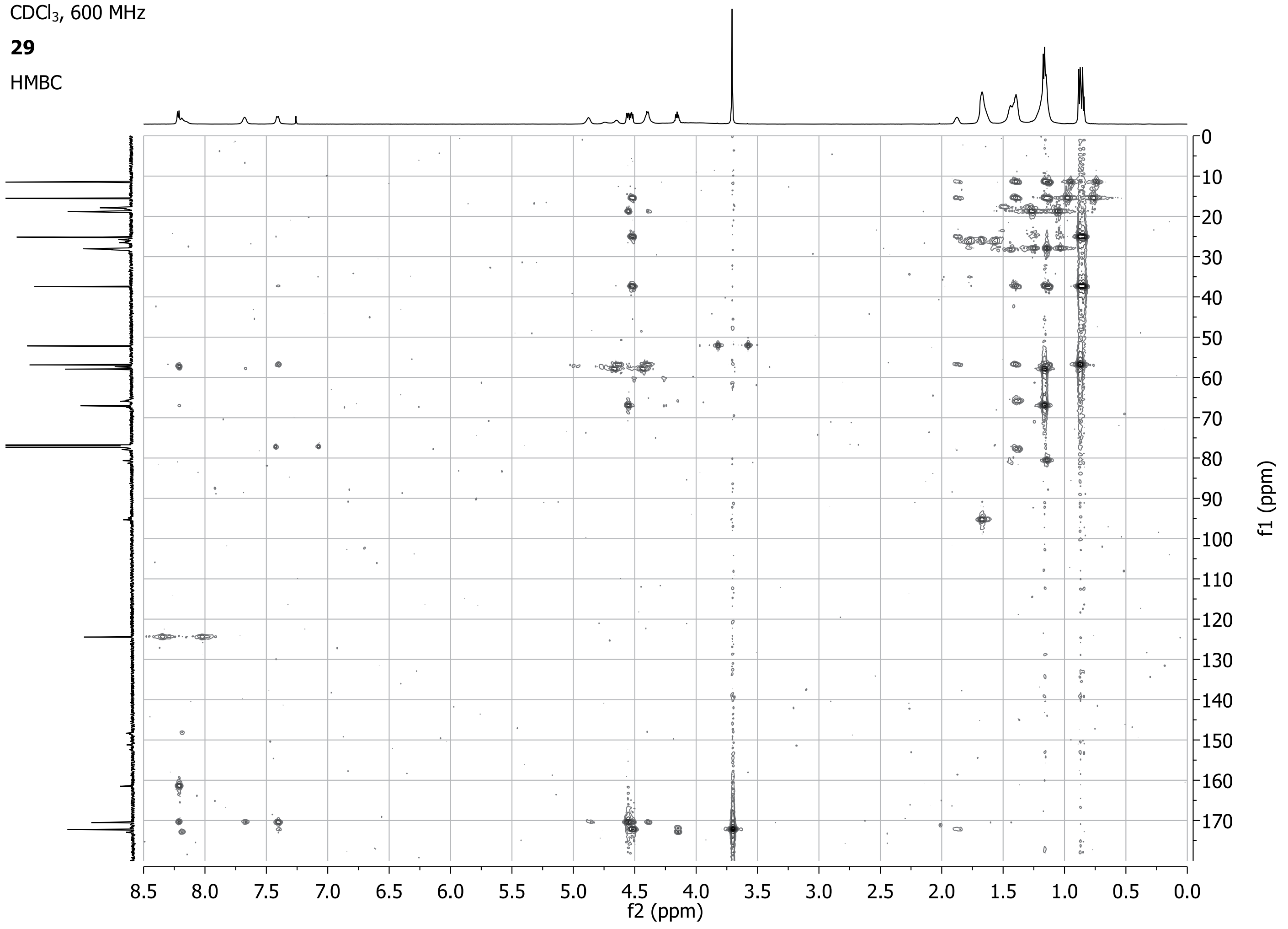

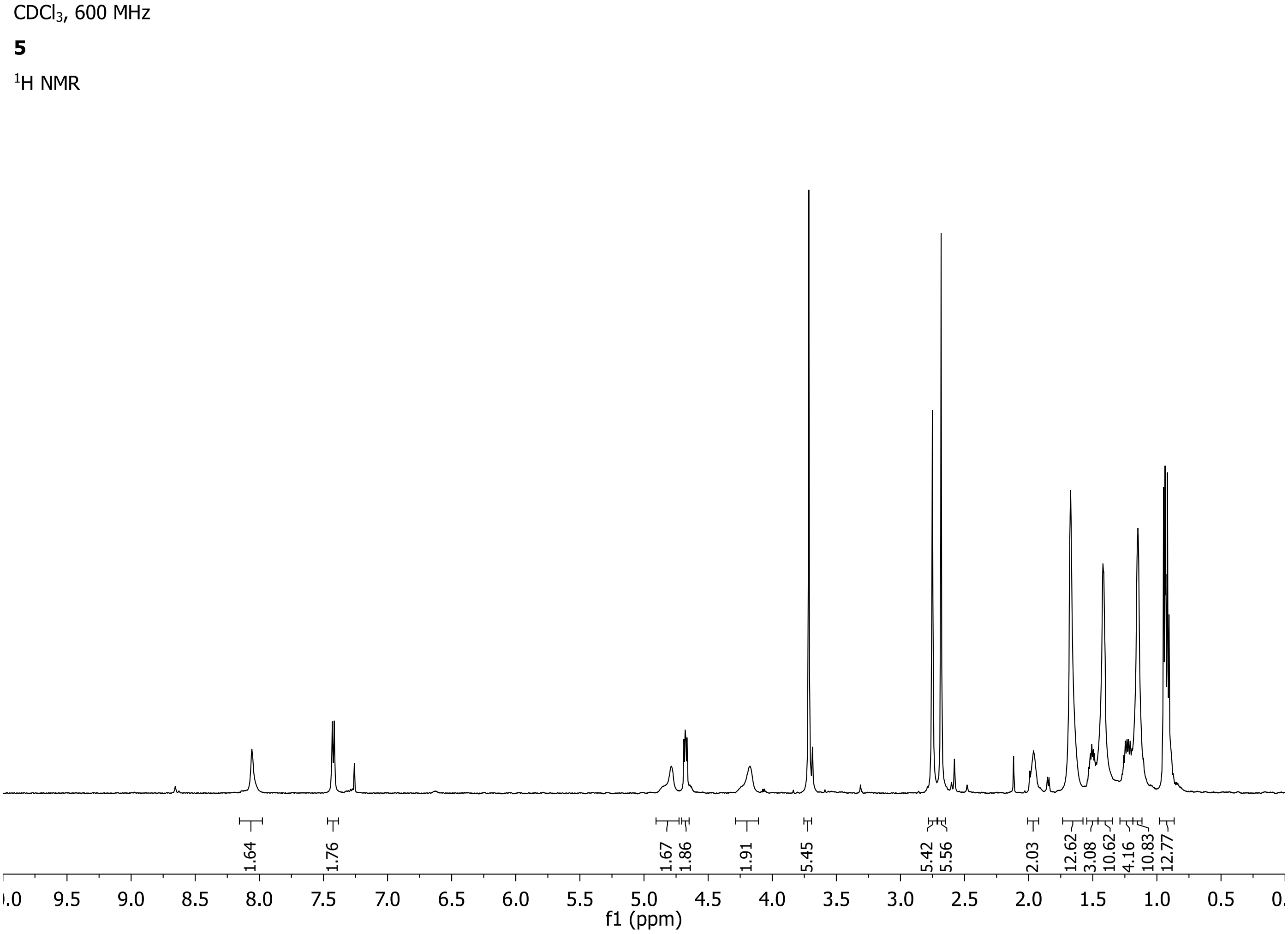
CDCl$_3$, 600 MHz
5
^{1}H NMR
1.64
1.76
1.67
1.86
1.91
5.45
5.42
5.56
2.03
12.62
3.08
10.62
4.16
10.83
12.77
9.5 9.0 8.5 8.0 7.5 7.0 6.5 6.0 5.5 5.0 4.5 4.0 3.5 3.0 2.5 2.0 1.5 1.0 0.5
f1 (ppm)

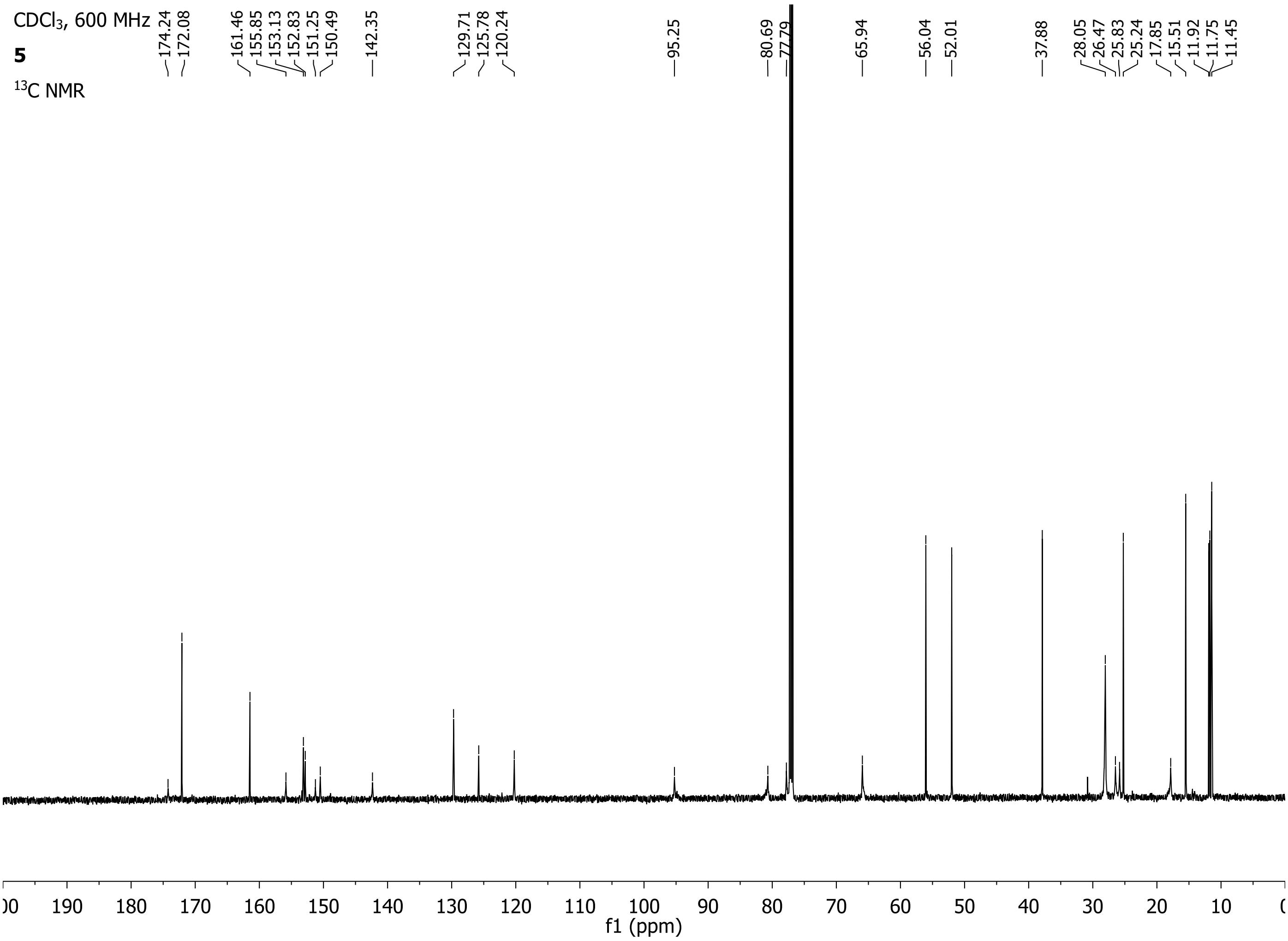
CDCl$_3$, 600 MHz
5
^{13}C NMR
174.24
172.08
161.46
155.85
153.13
152.83
151.25
150.49
142.35
129.71
125.78
120.24
95.25
80.69
77.79
65.94
56.04
52.01
37.88
28.05
26.47
25.83
25.24
17.85
15.51
11.92
11.75
11.45
190 180 170 160 150 140 130 120 110 100 90 80 70 60 50 40 30 20 10
f1 (ppm)

CDCl$_3$, 600 MHz
5
DEPT-135

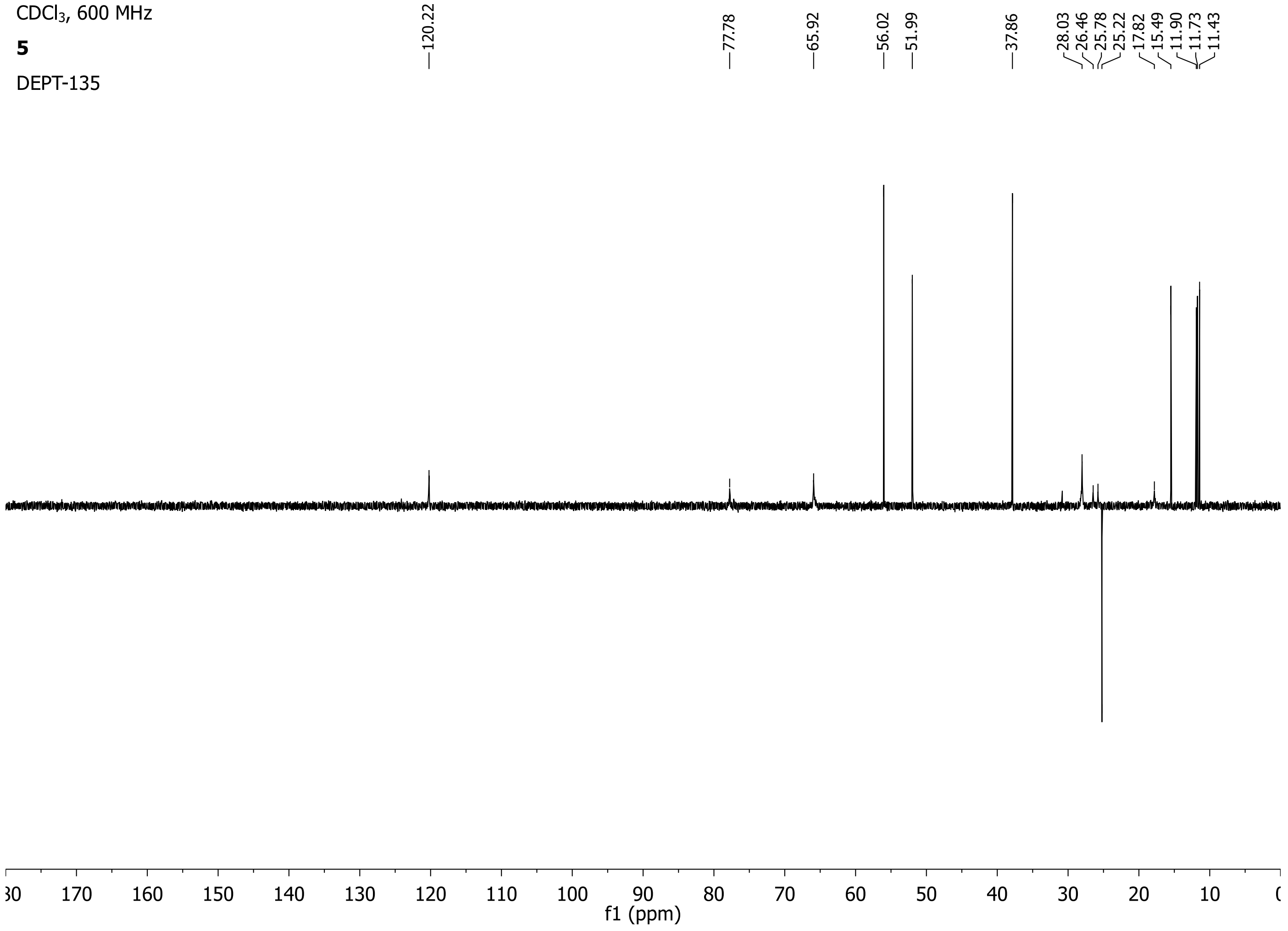

CDCl$_3$, 600 MHz
5
COSY

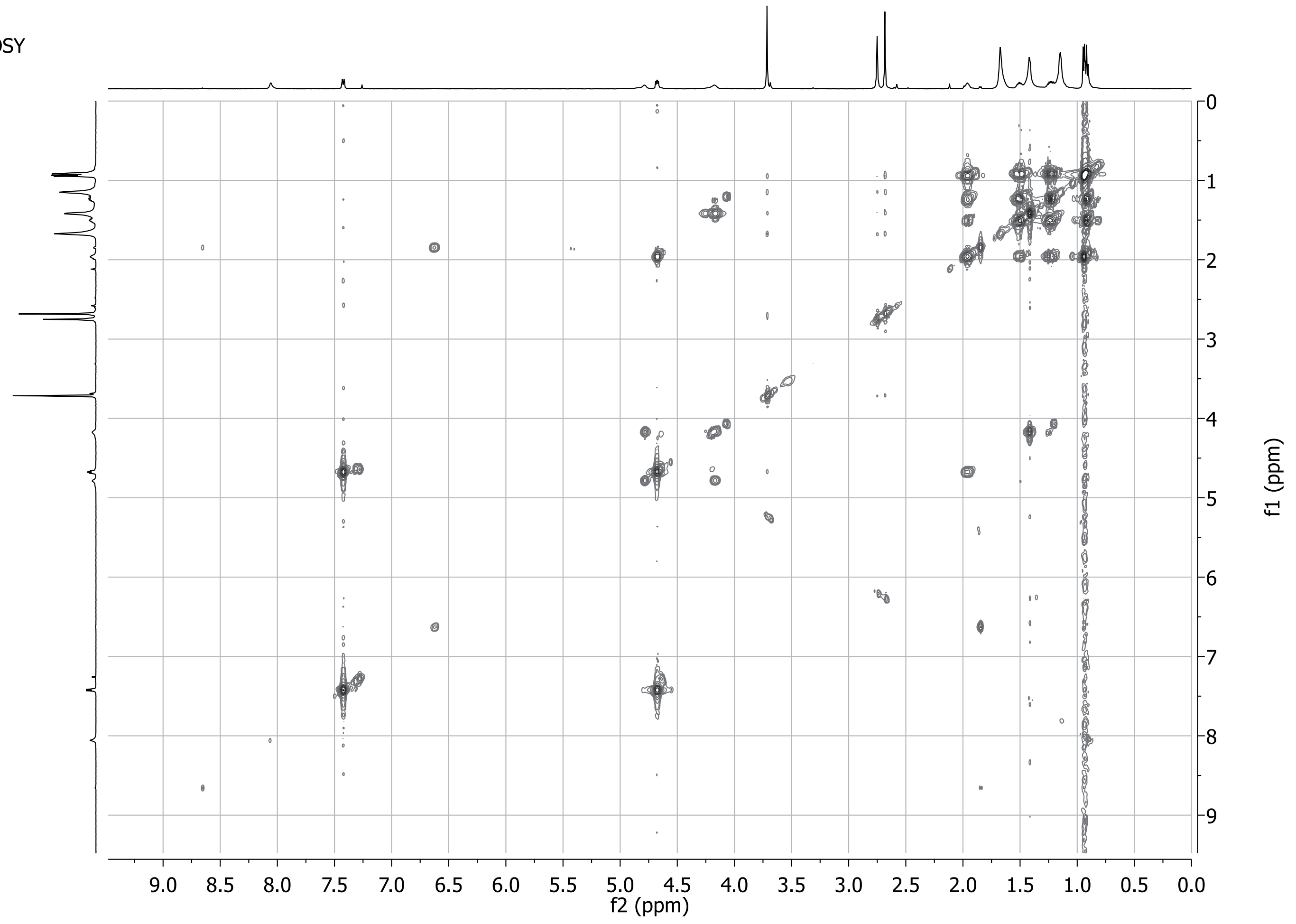

CDCl$_3$, 600 MHz

5

HMQC

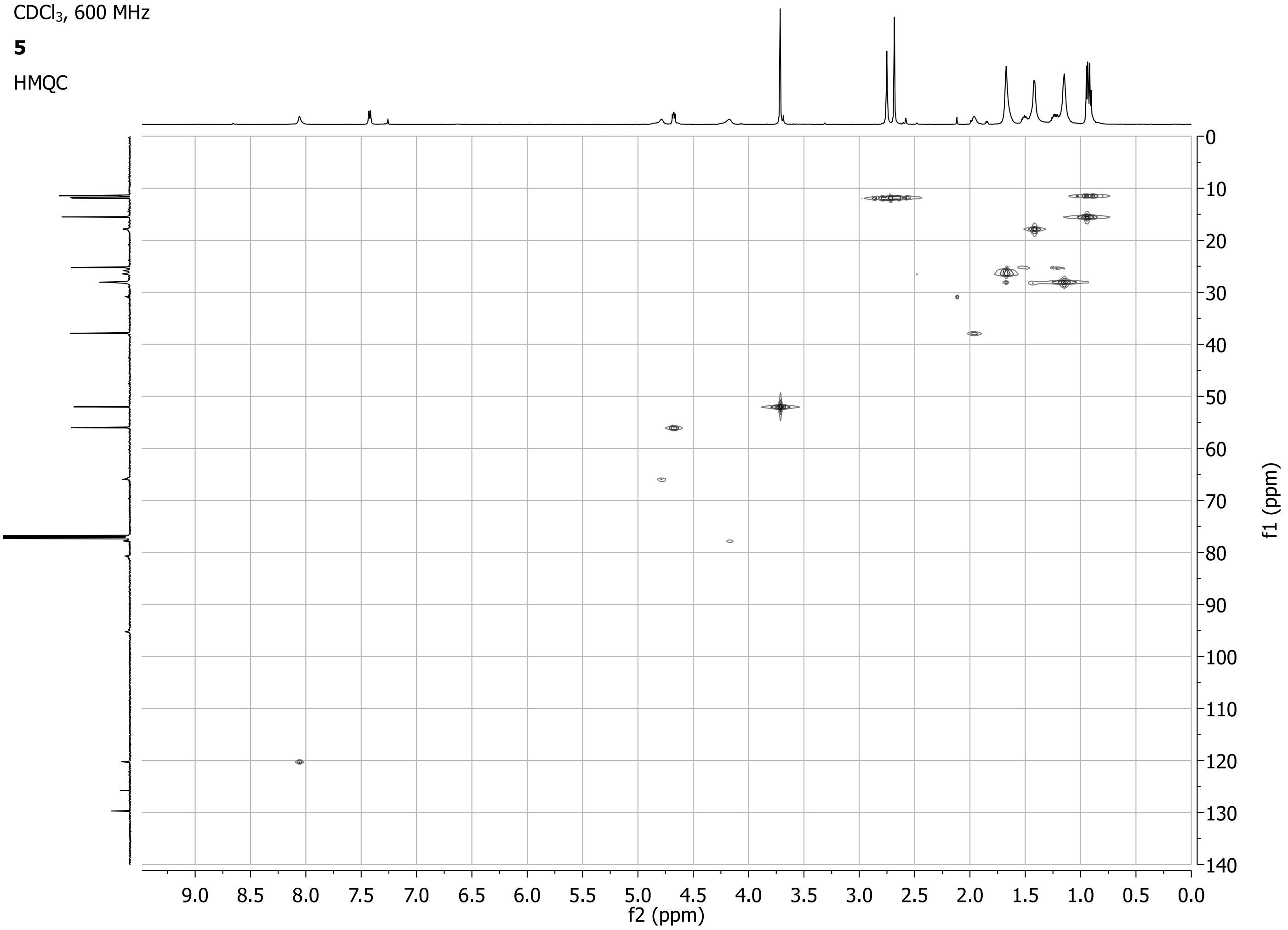

CDCl$_3$, 600 MHz

5

HMBC

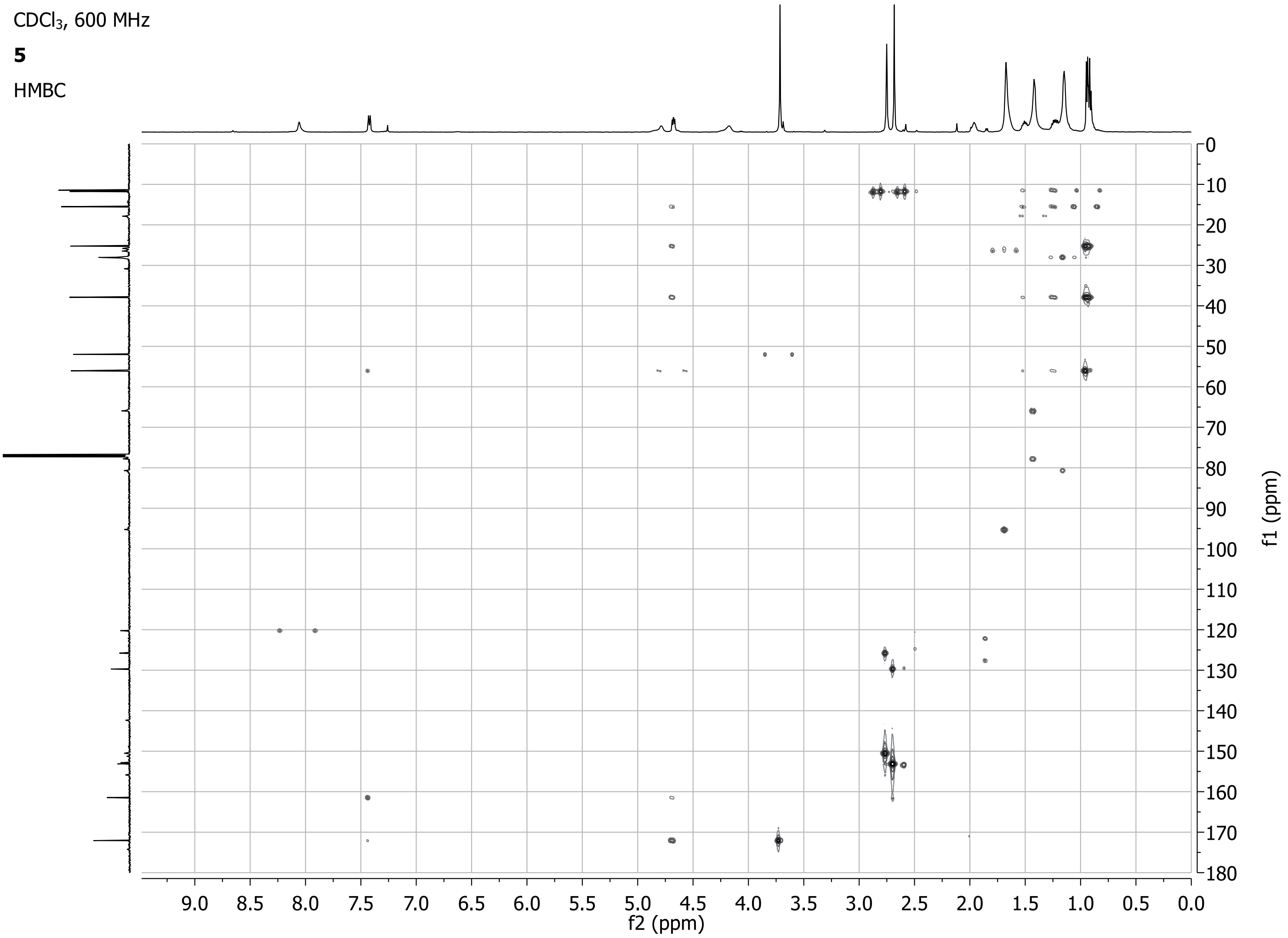

CDCl3, 600 MHz

30a

^{1}H NMR

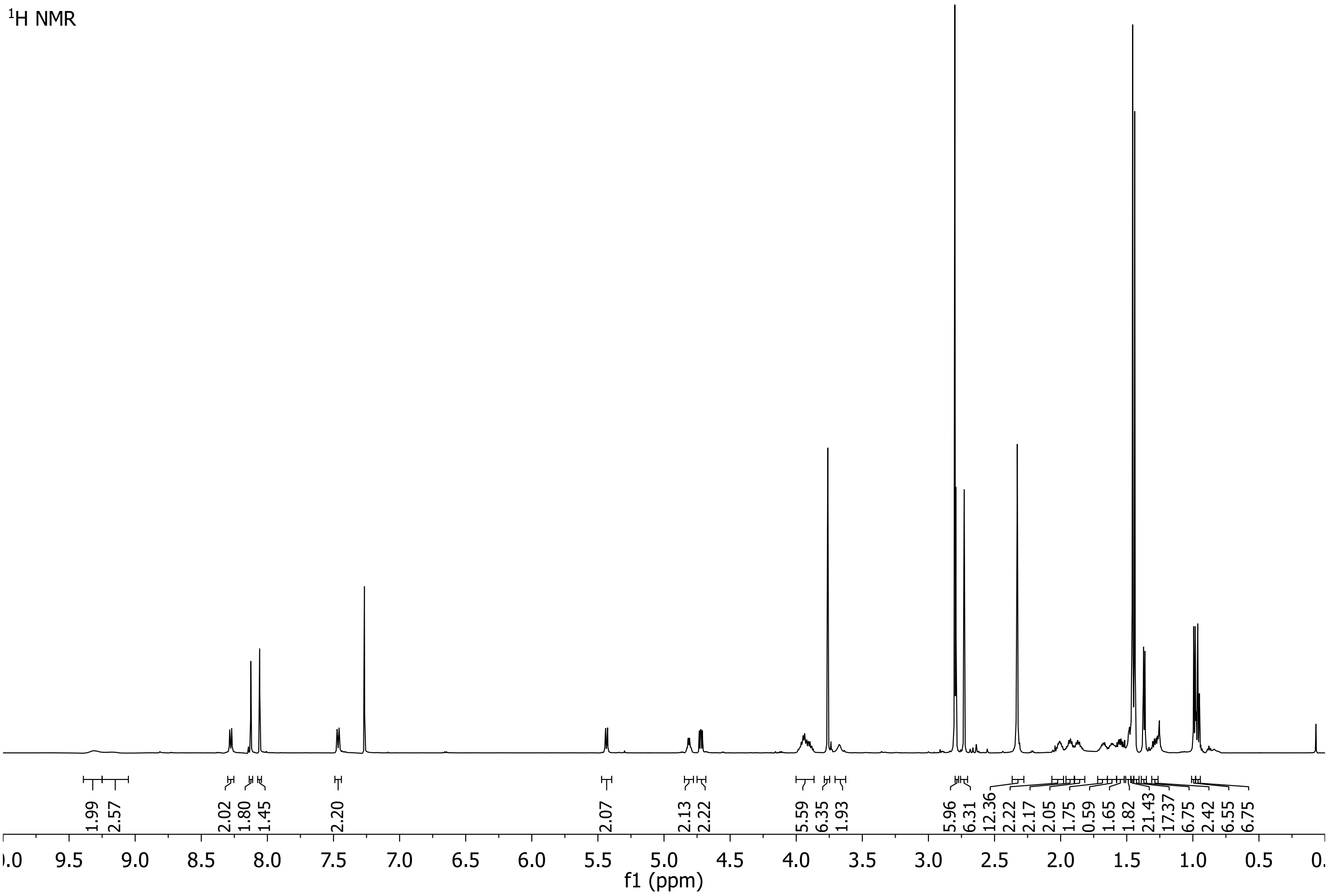

CDCl3, 600 MHz

30a

^{13}C NMR

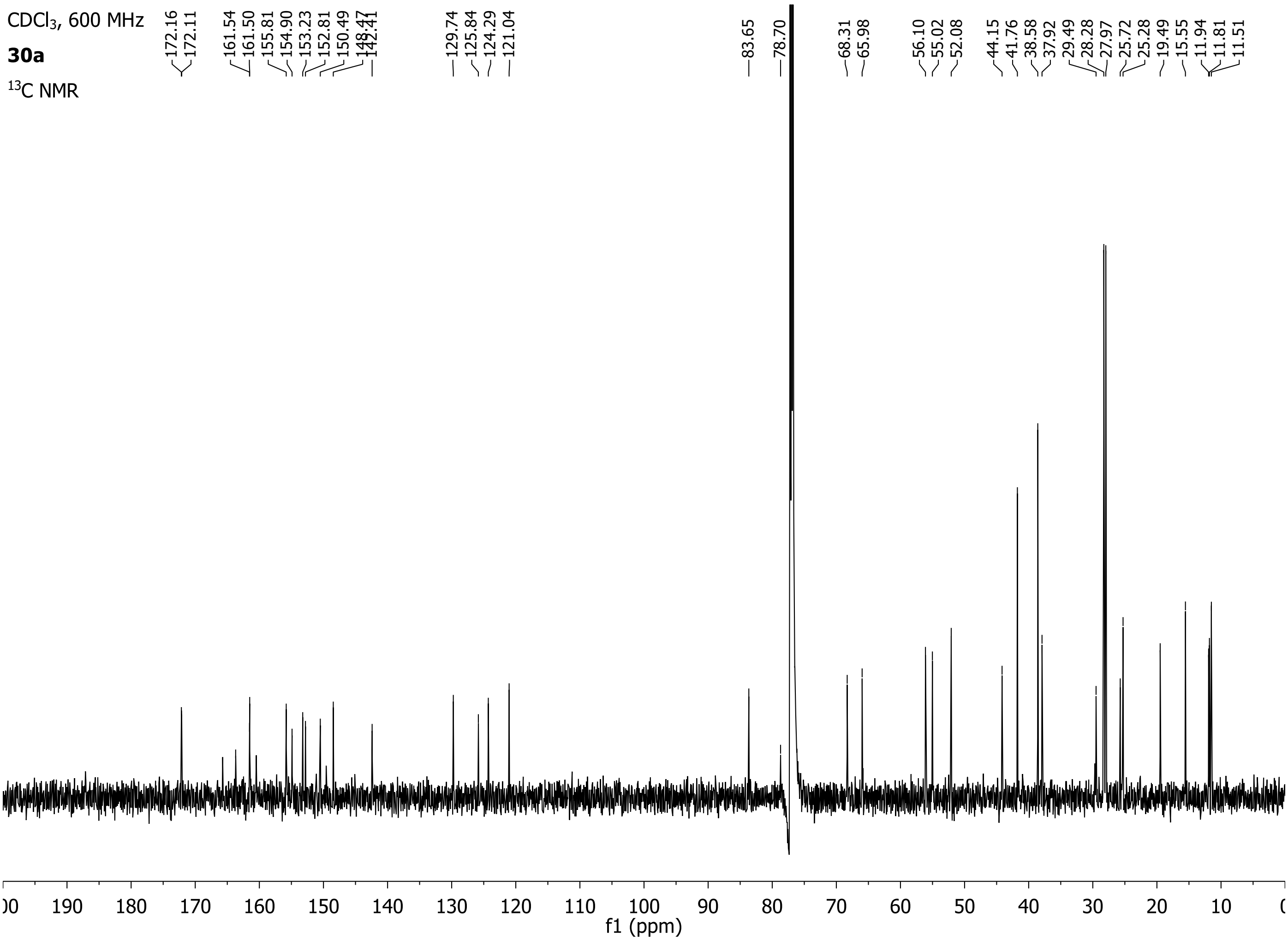

CDCl$_3$, 600 MHz

30a

DEPT-135

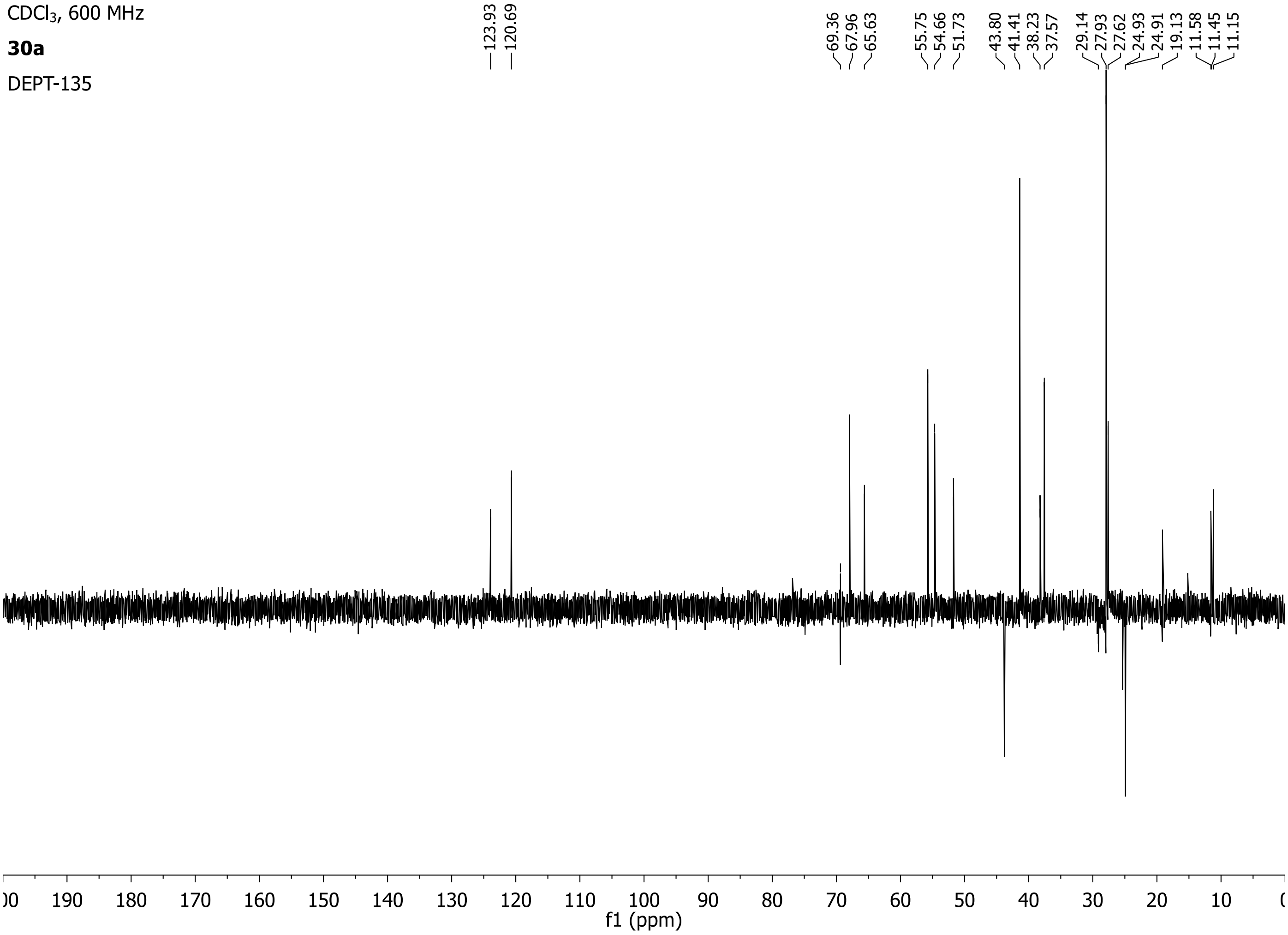

CDCl$_3$, 600 MHz

30a

COSY

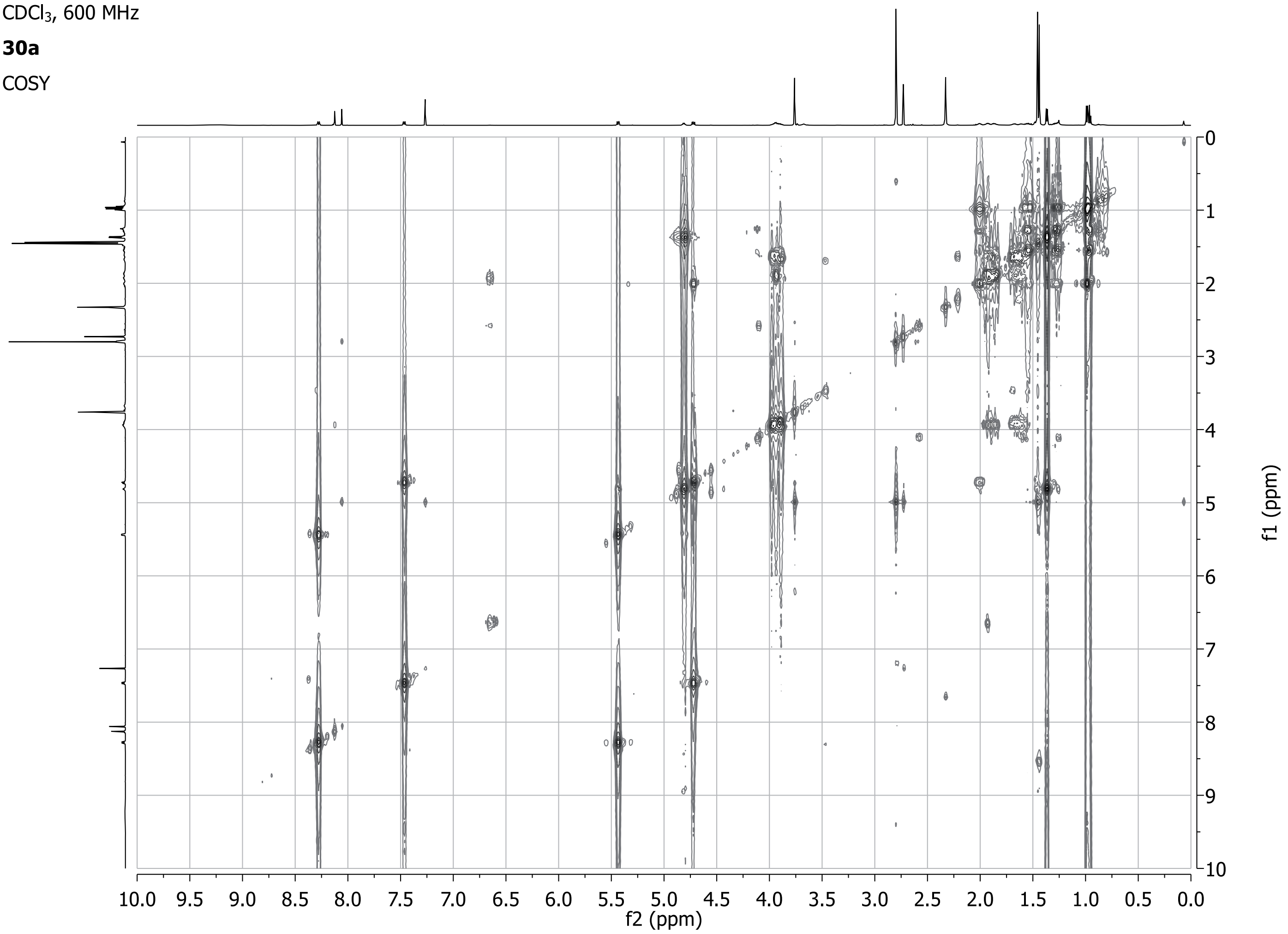

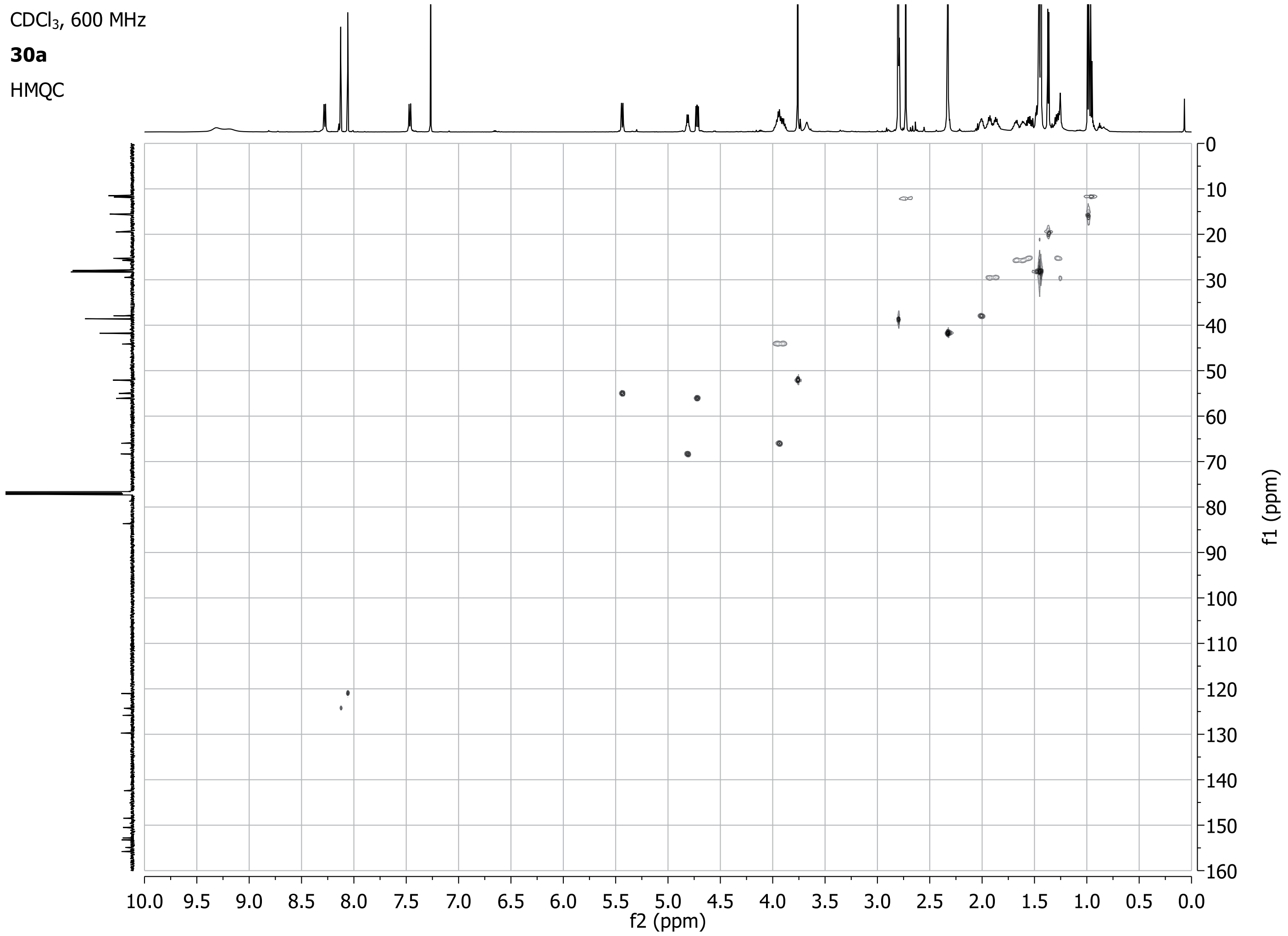
CDCl3, 600 MHz
30a
HMQC
f1 (ppm)
f2 (ppm)

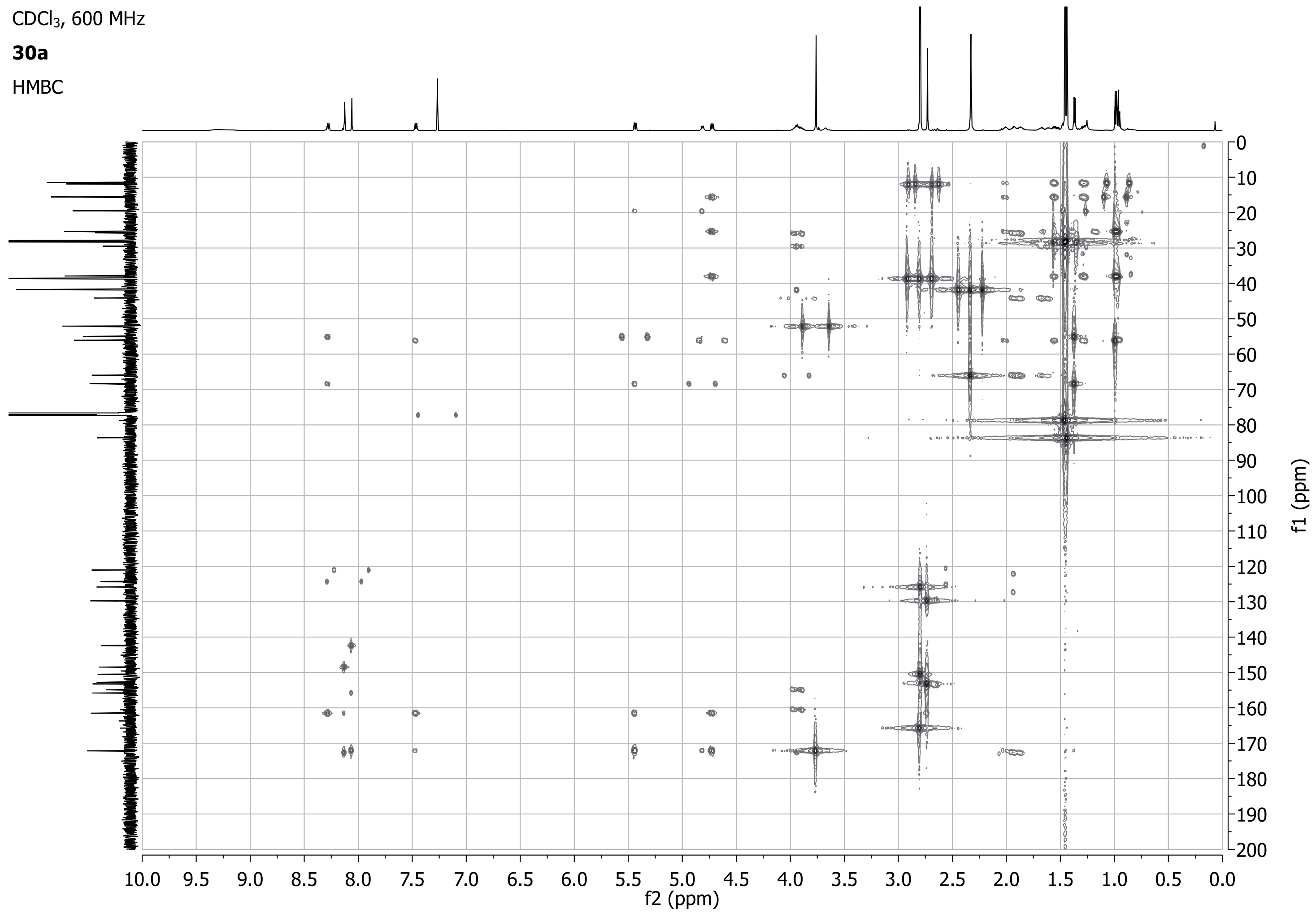
CDCl3, 600 MHz
30a
HMBC
f1 (ppm)
f2 (ppm)

CDCl3, 600 MHz

2a

^1H NMR

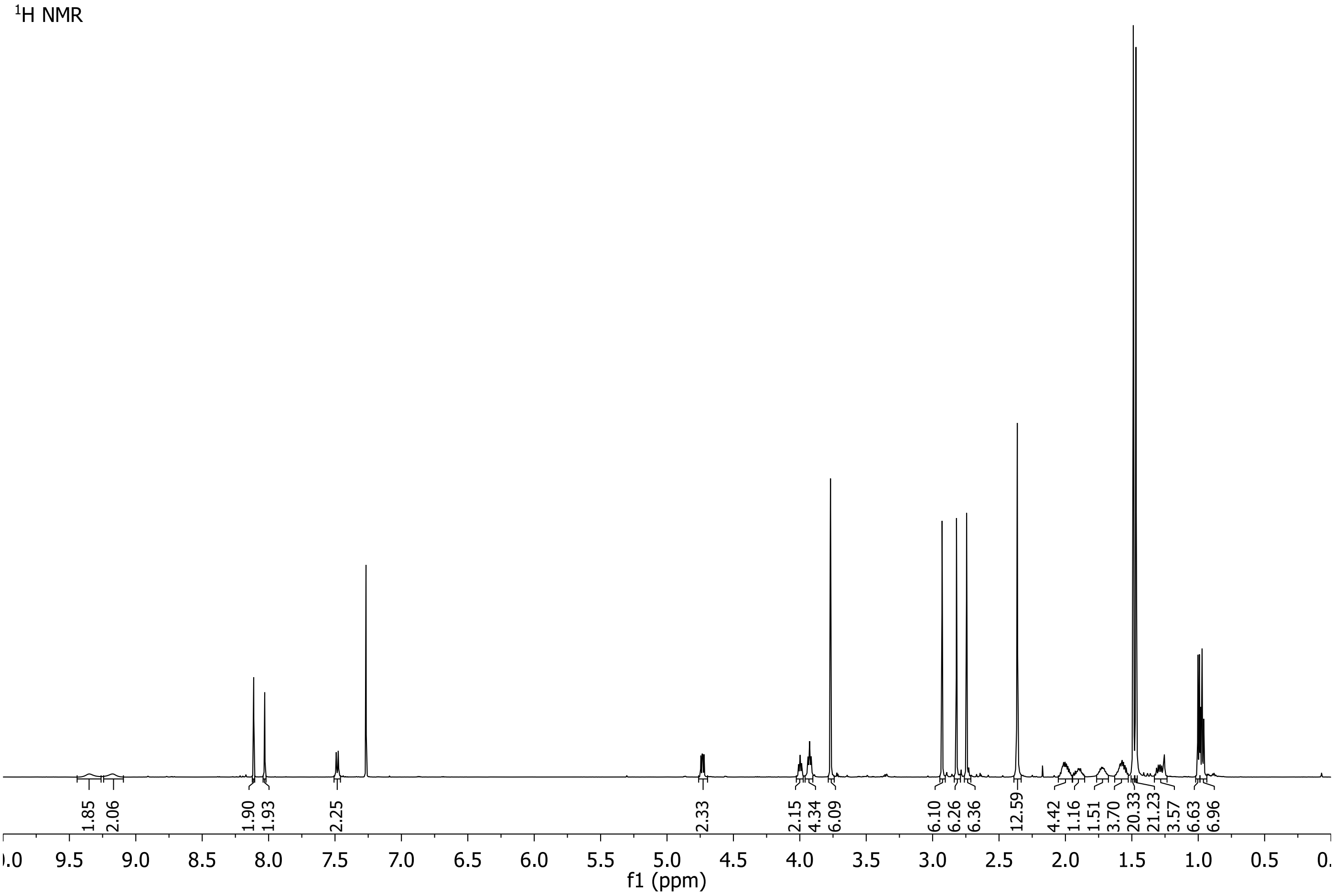

CDCl3, 600 MHz

2a

^{13}C NMR

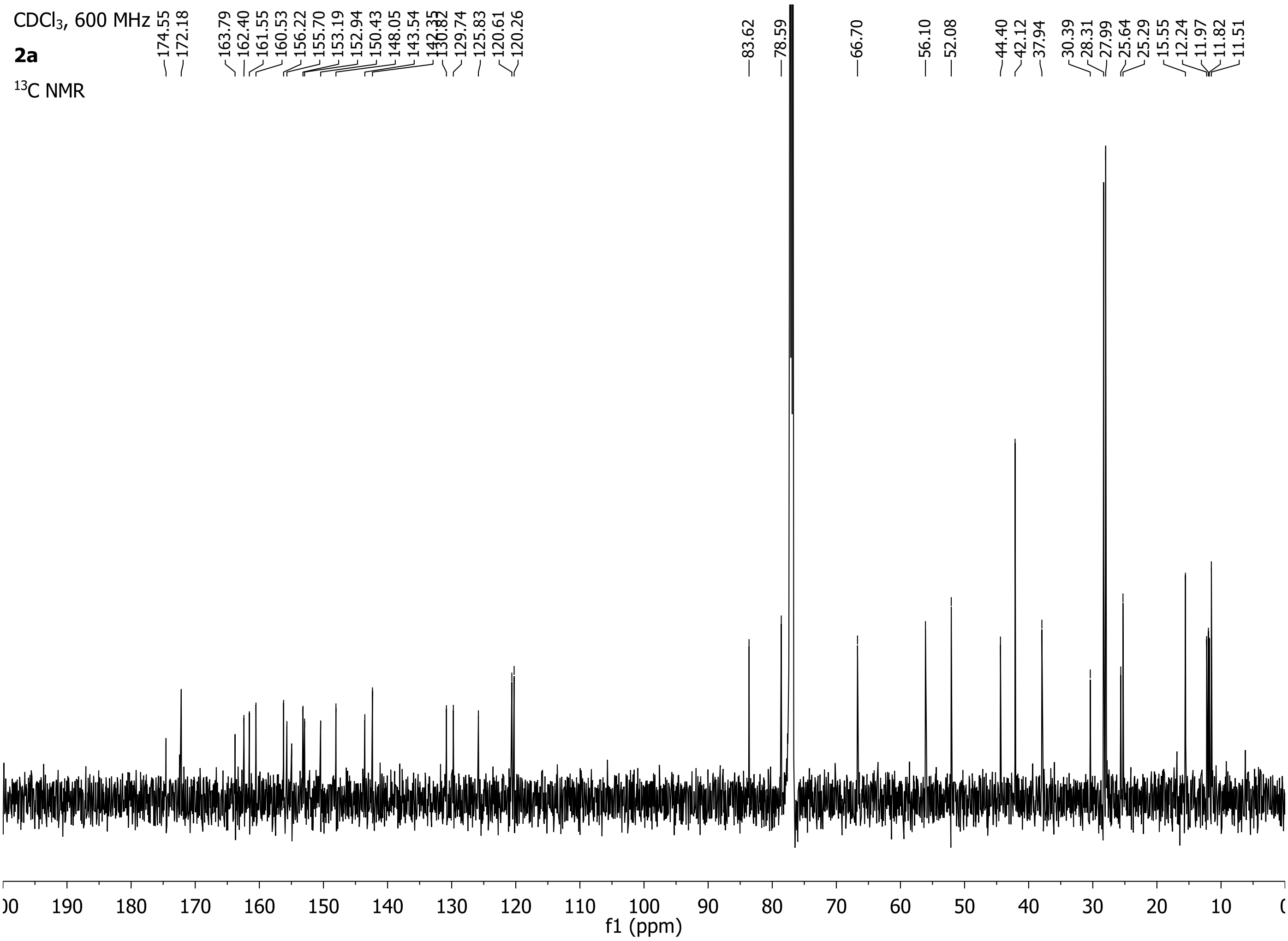

CDCl$_3$, 600 MHz

2a

DEPT-135

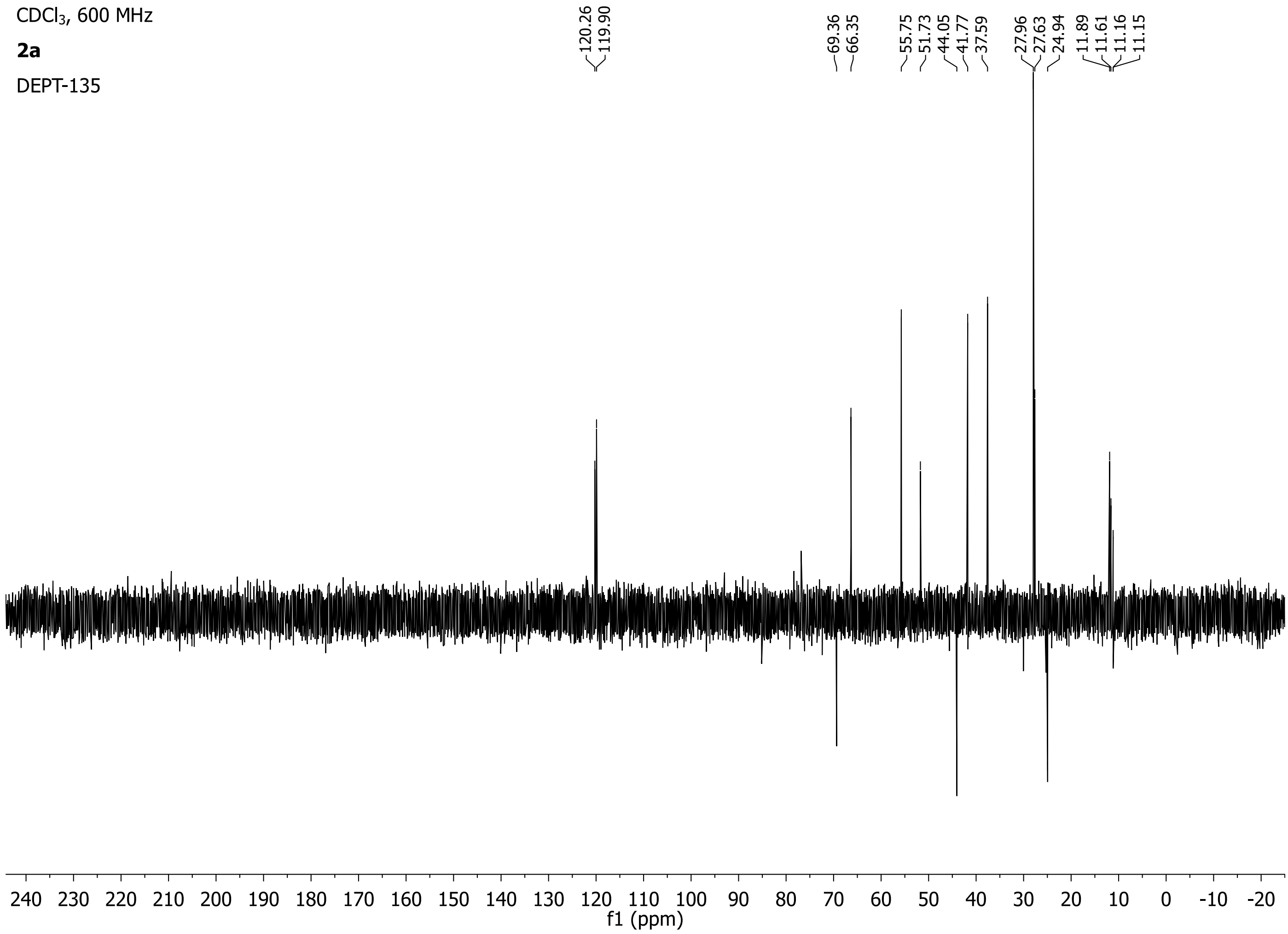

CDCl$_3$, 600 MHz

2a

COSY

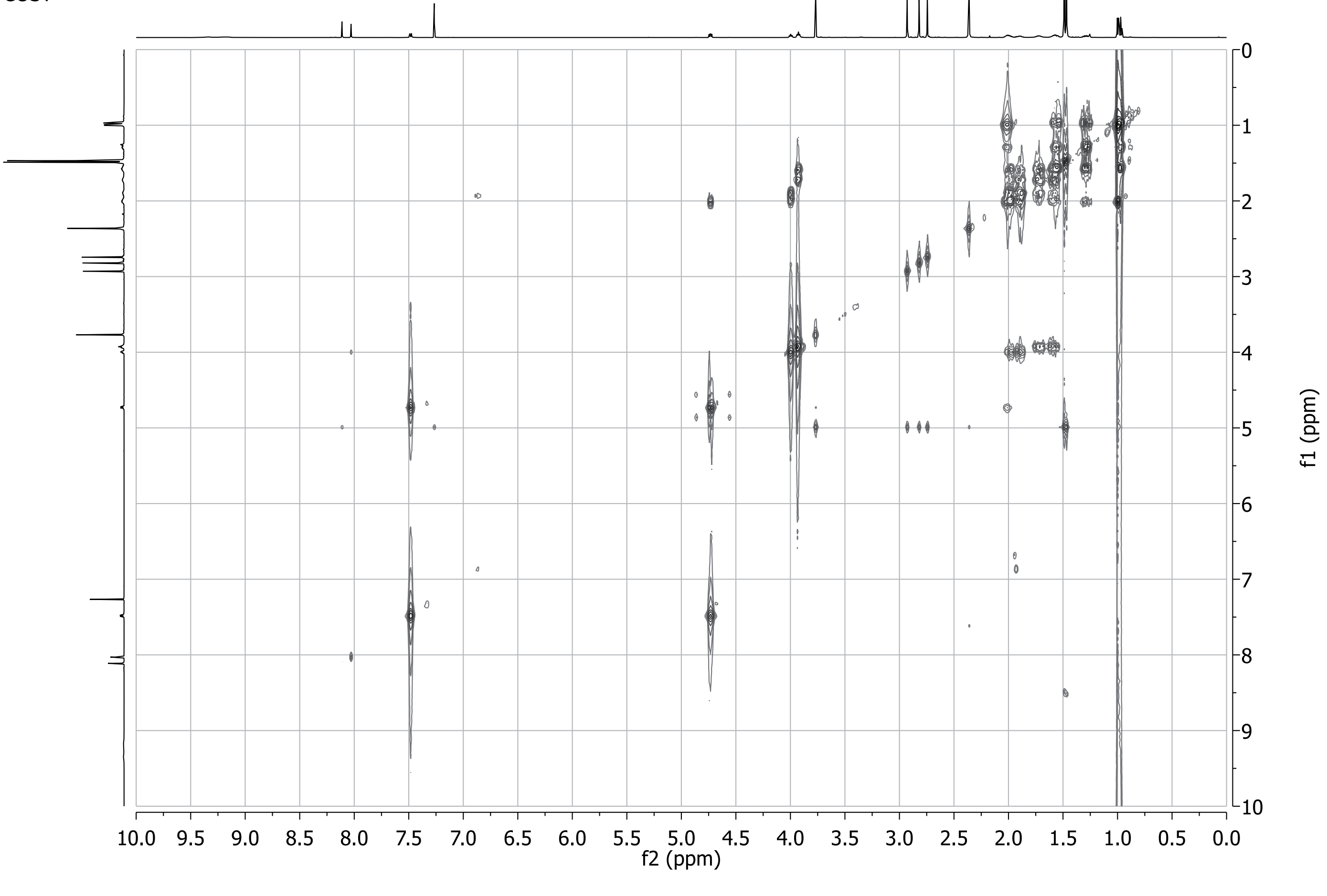

CDCl$_3$, 600 MHz

2a

HMQC

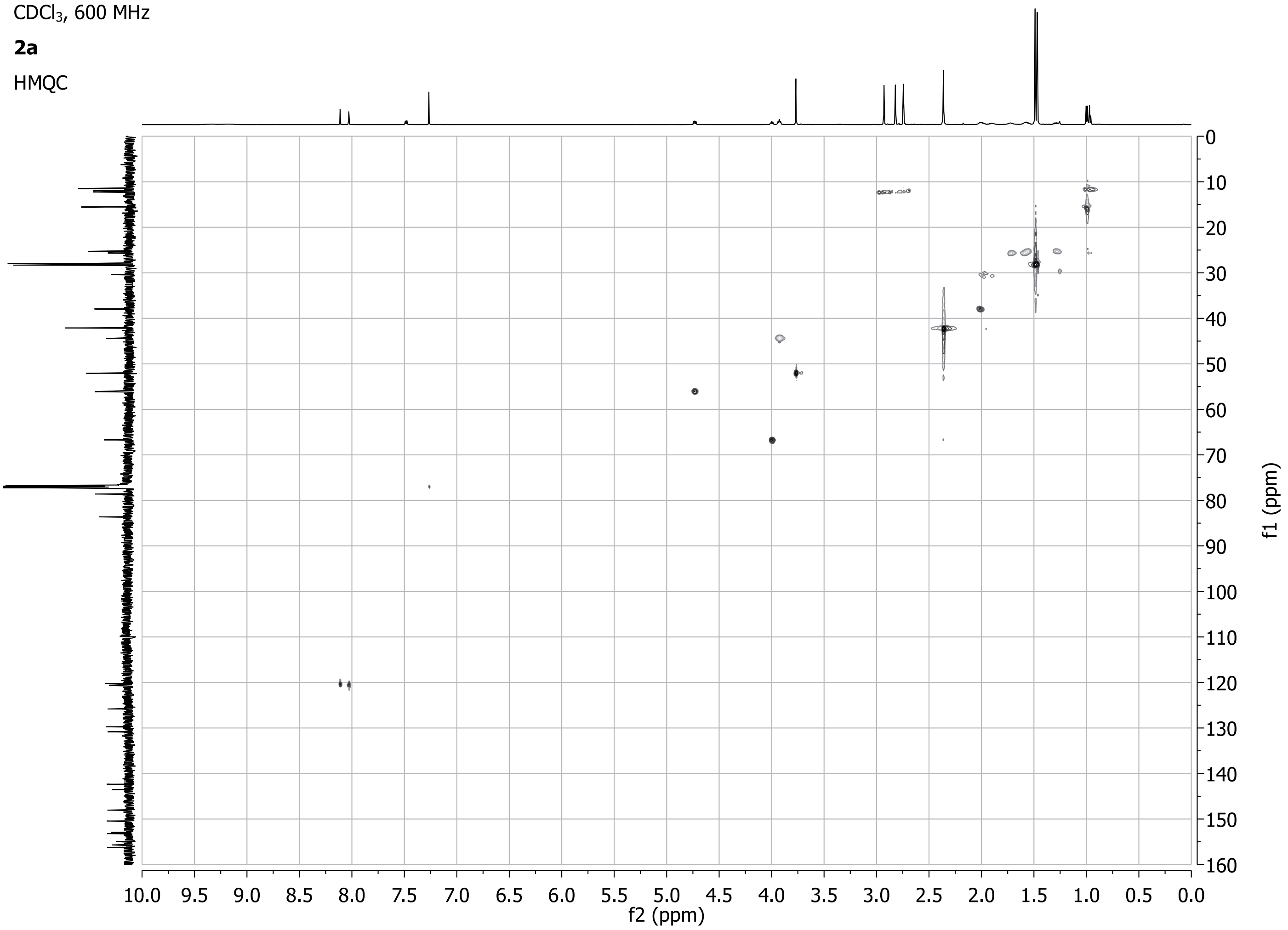

CDCl$_3$, 600 MHz

2a

HMBC

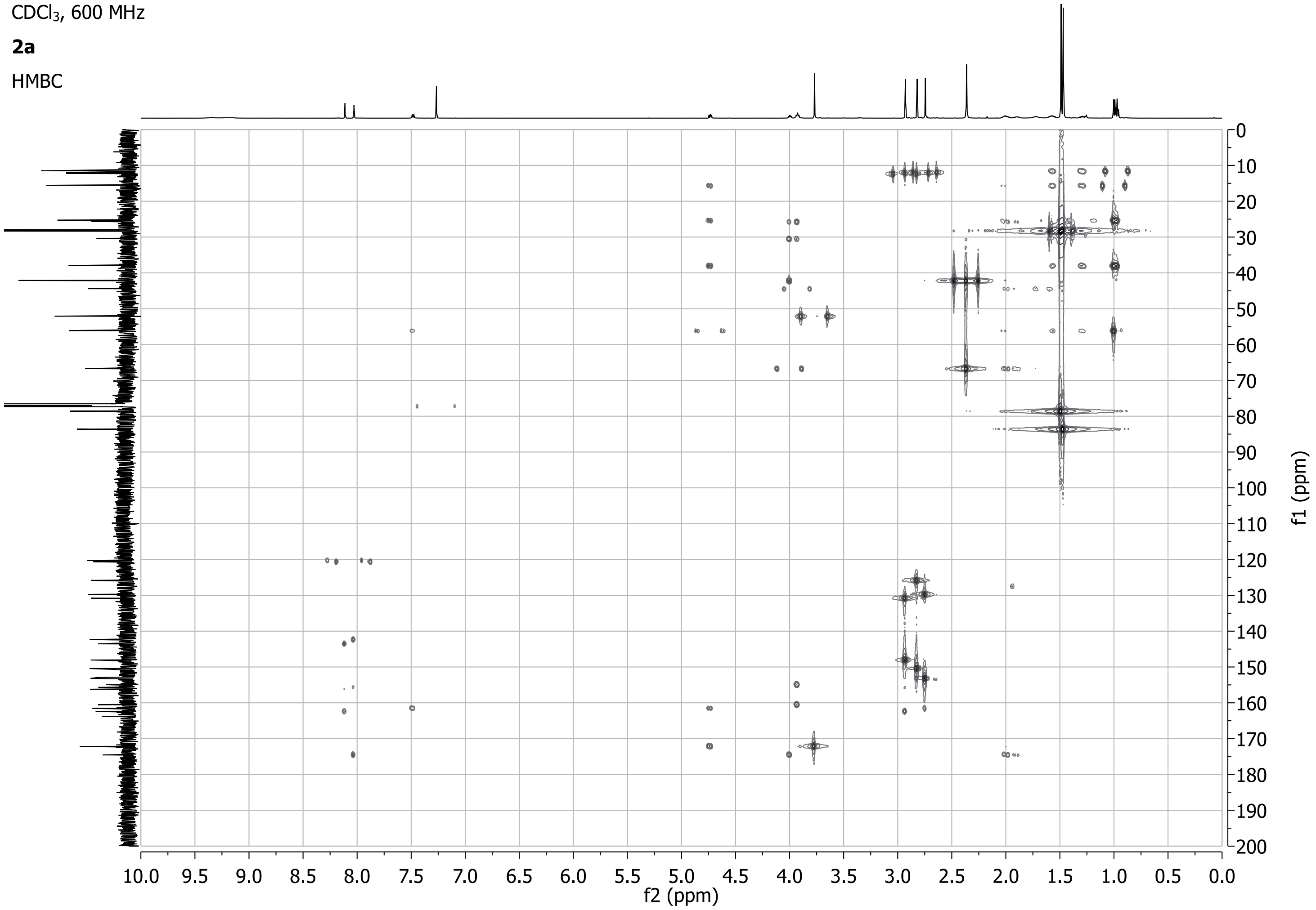

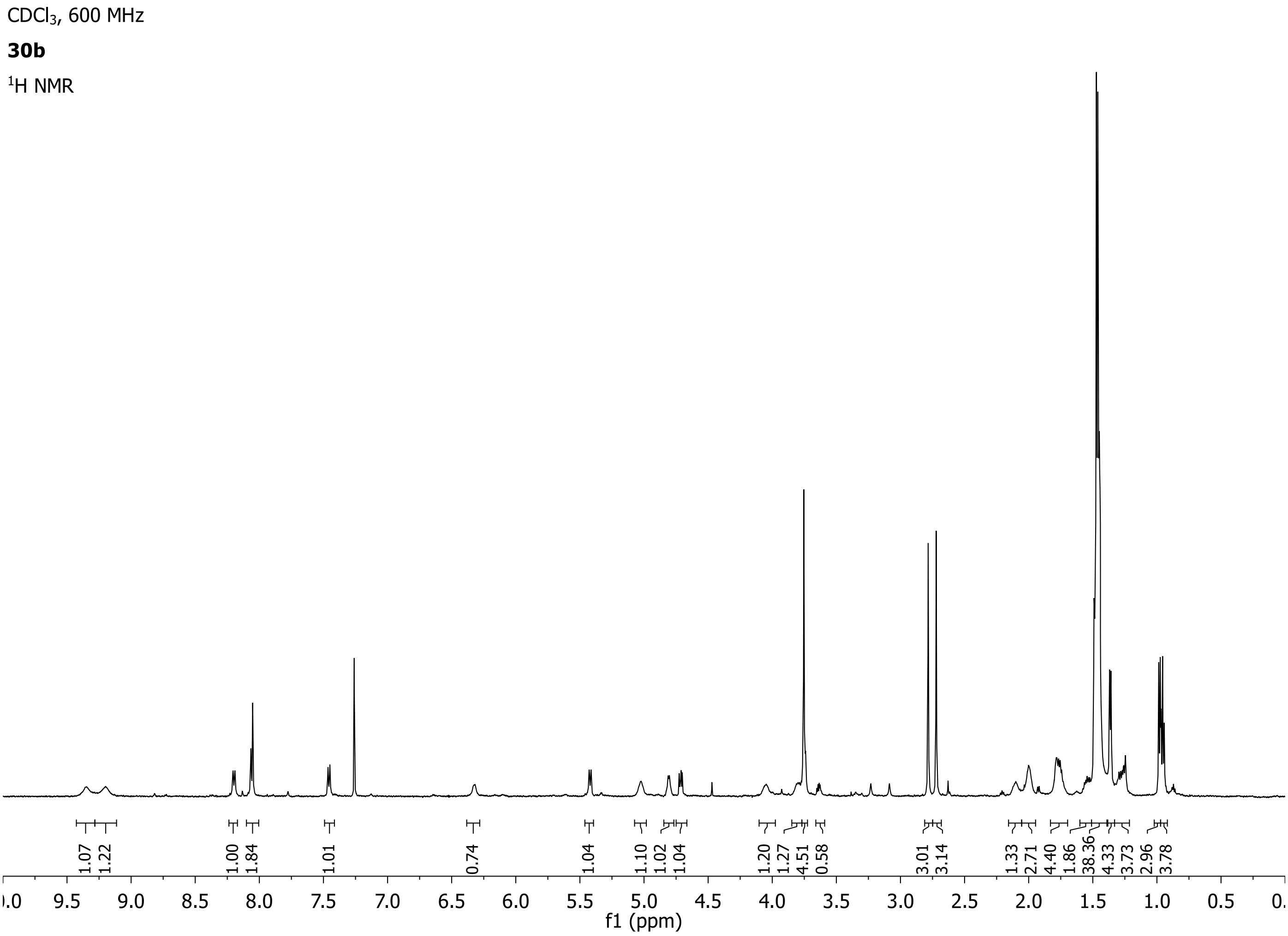
CDCl3, 600 MHz
30b
1H NMR
1.07
1.22
1.00
1.84
1.01
0.74
1.04
1.10
1.02
1.04
1.20
1.27
4.51
0.58
3.01
3.14
1.33
2.71
4.40
1.86
38.36
4.33
3.73
2.96
3.78
9.5 9.0 8.5 8.0 7.5 7.0 6.5 6.0 5.5 5.0 4.5 4.0 3.5 3.0 2.5 2.0 1.5 1.0 0.5
f1 (ppm)

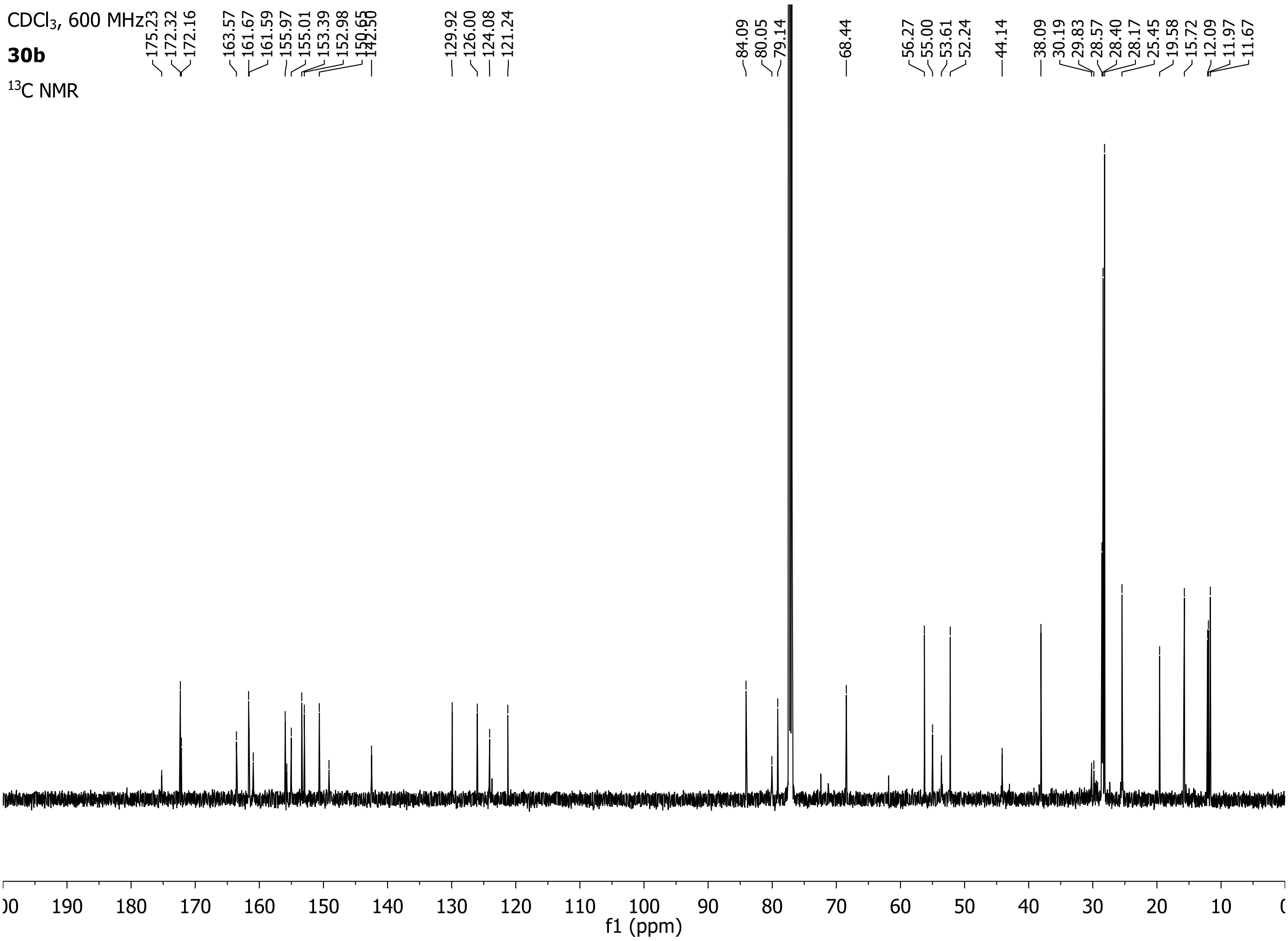
CDCl3, 600 MHz
30b
13C NMR
175.23
172.32
172.16
163.57
161.67
161.59
155.97
155.01
153.39
152.98
150.65
142.50
129.92
126.00
124.08
121.24
84.09
80.05
79.14
68.44
56.27
55.00
53.61
52.24
44.14
38.09
30.19
29.83
28.57
28.40
28.17
25.45
19.58
15.72
12.09
11.97
11.67
190 180 170 160 150 140 130 120 110 100 90 80 70 60 50 40 30 20 10
f1 (ppm)

CDCl$_3$, 600 MHz

30b

DEPT-135

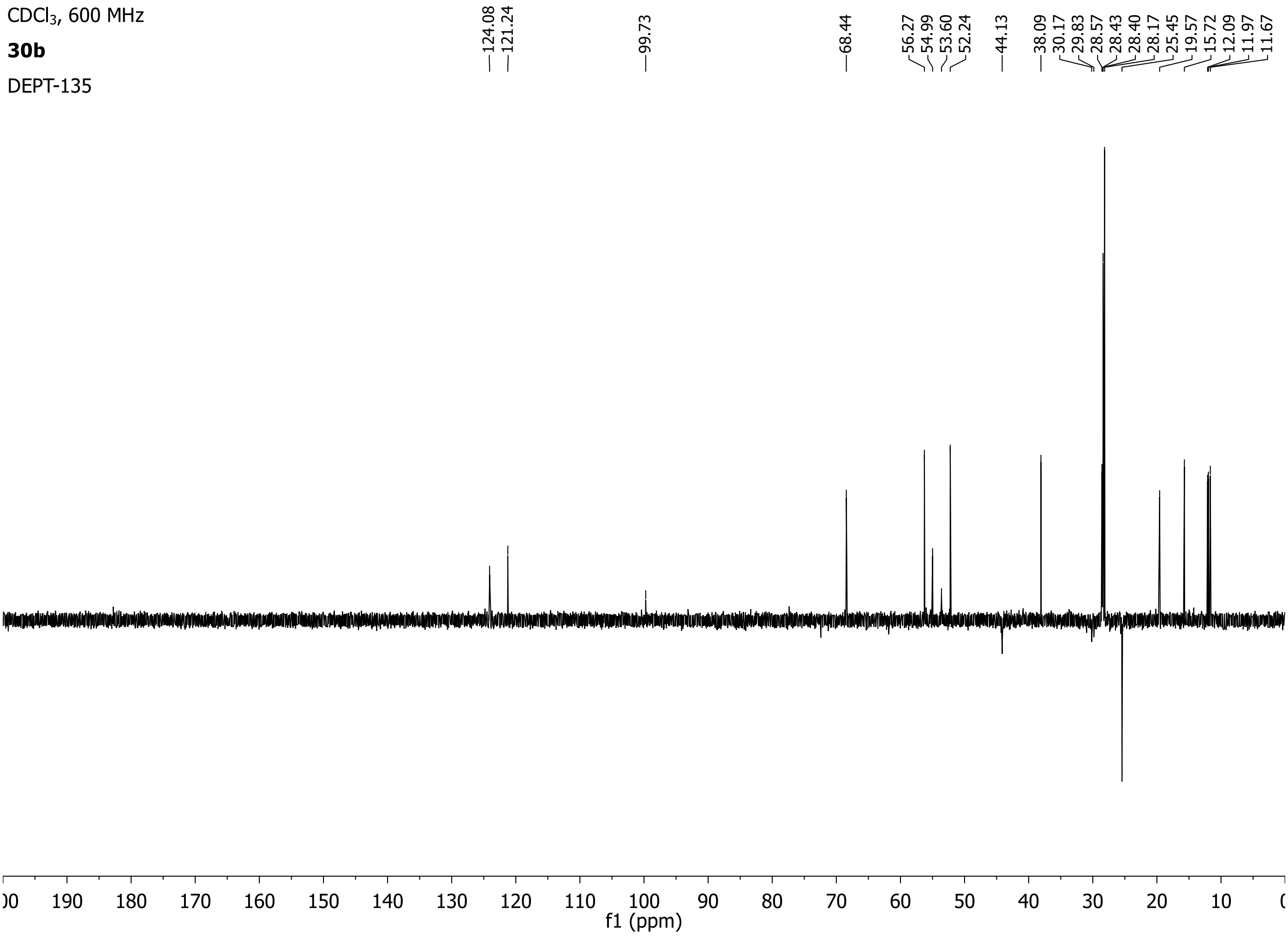

CDCl$_3$, 600 MHz

30b

COSY

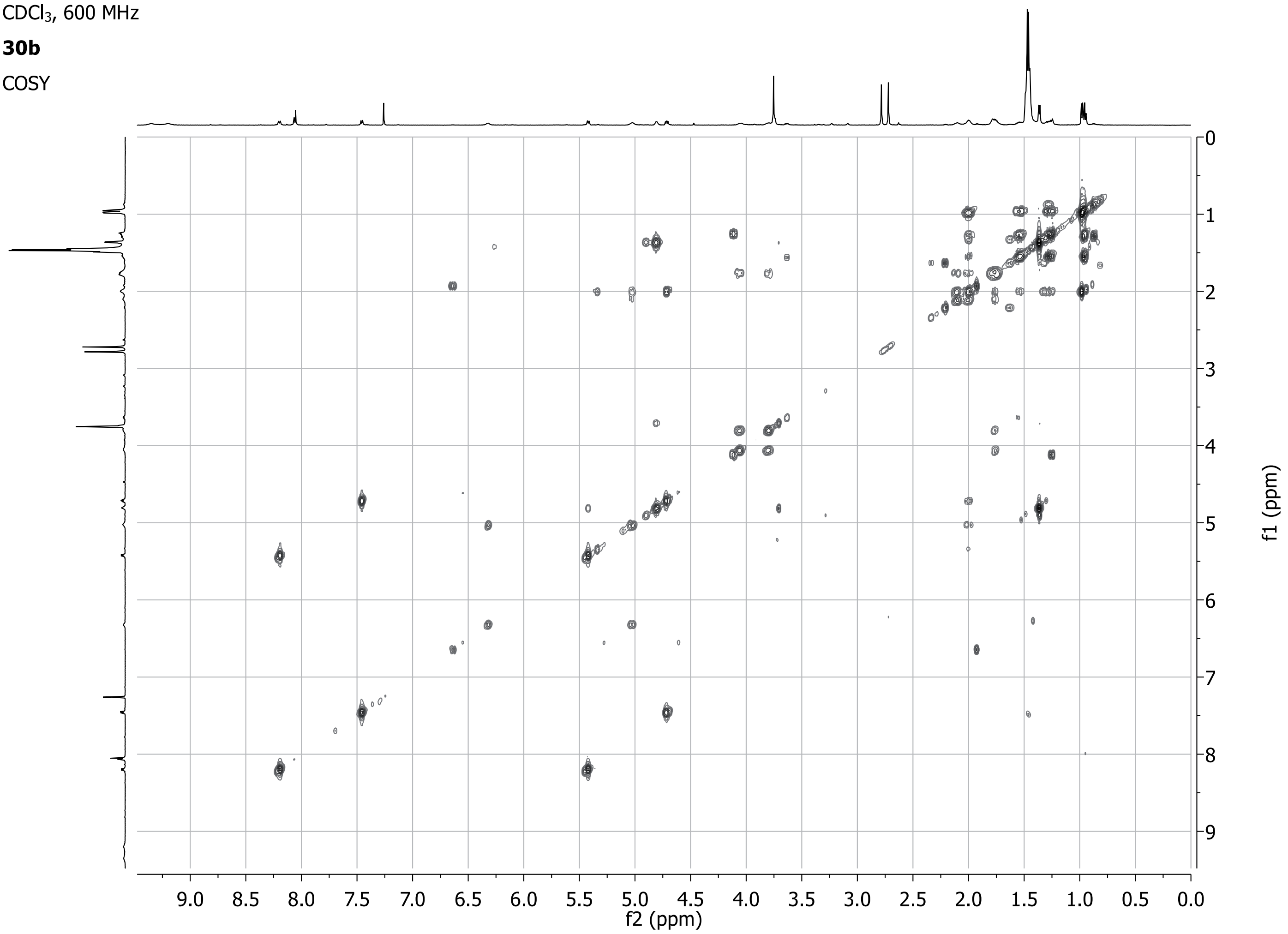

CDCl$_3$, 600 MHz

30b

HMQC

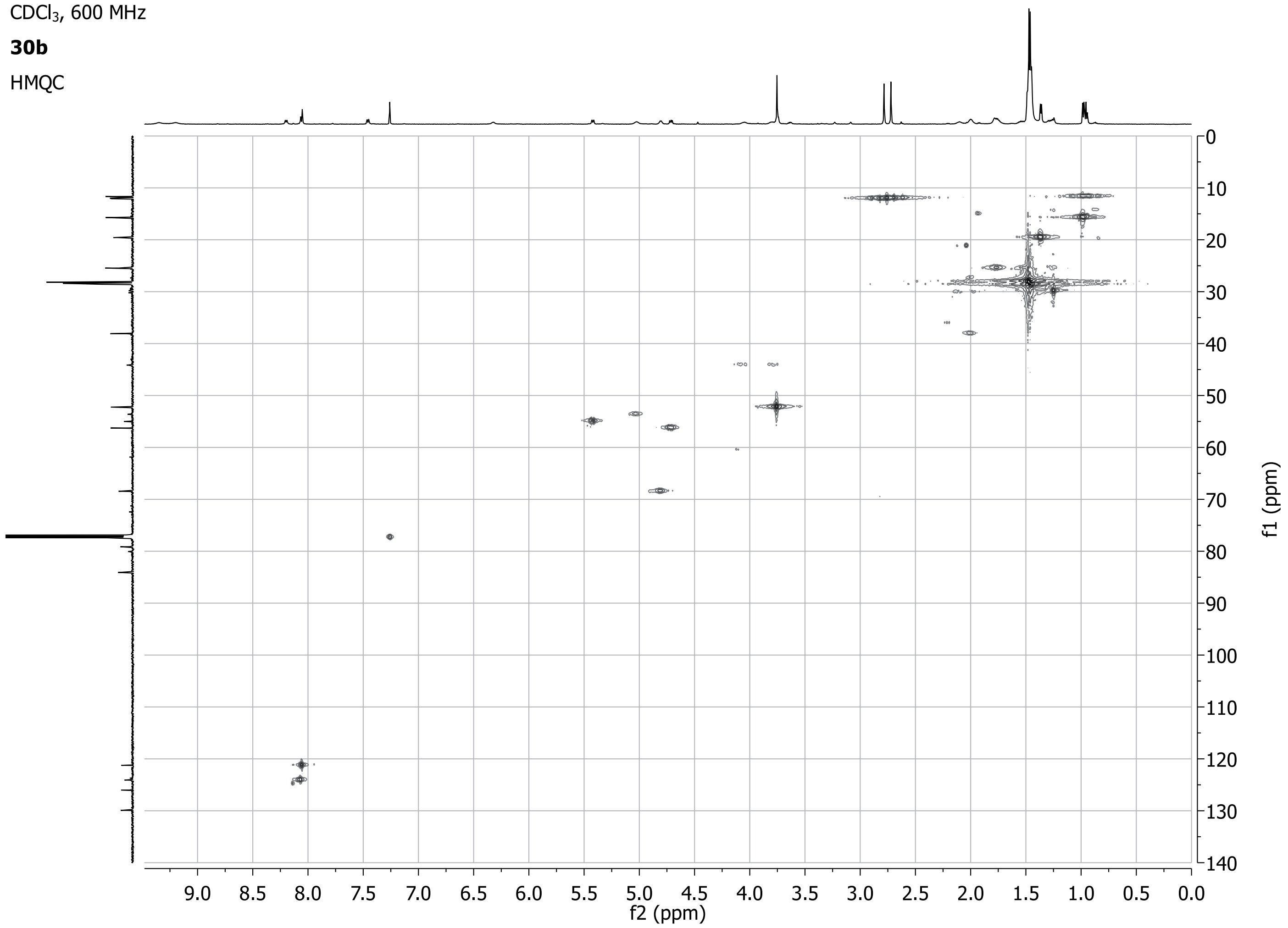

CDCl$_3$, 600 MHz

30b

HMBC

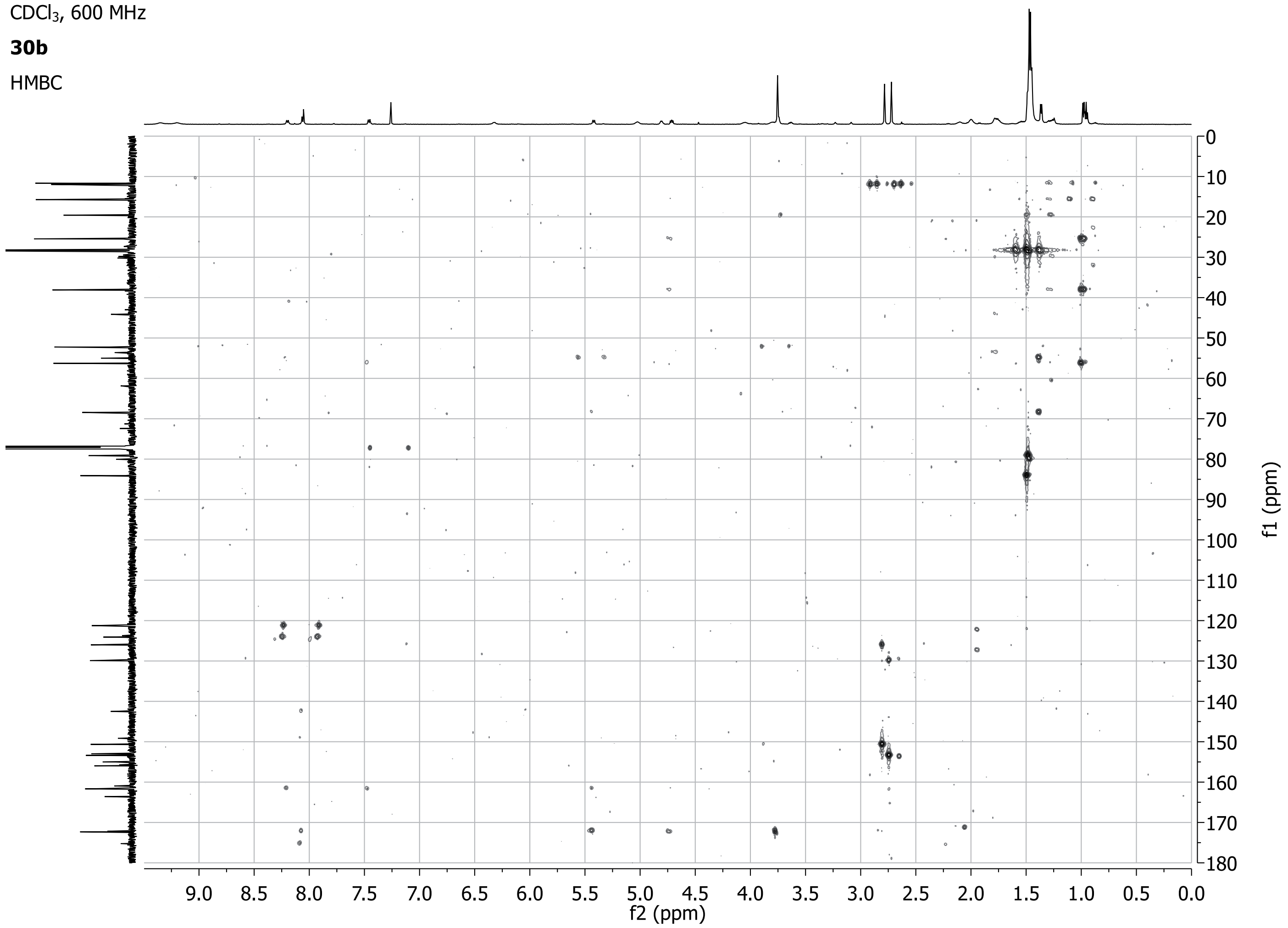

CDCl$_3$, 600 MHz

2b

^{1}H NMR

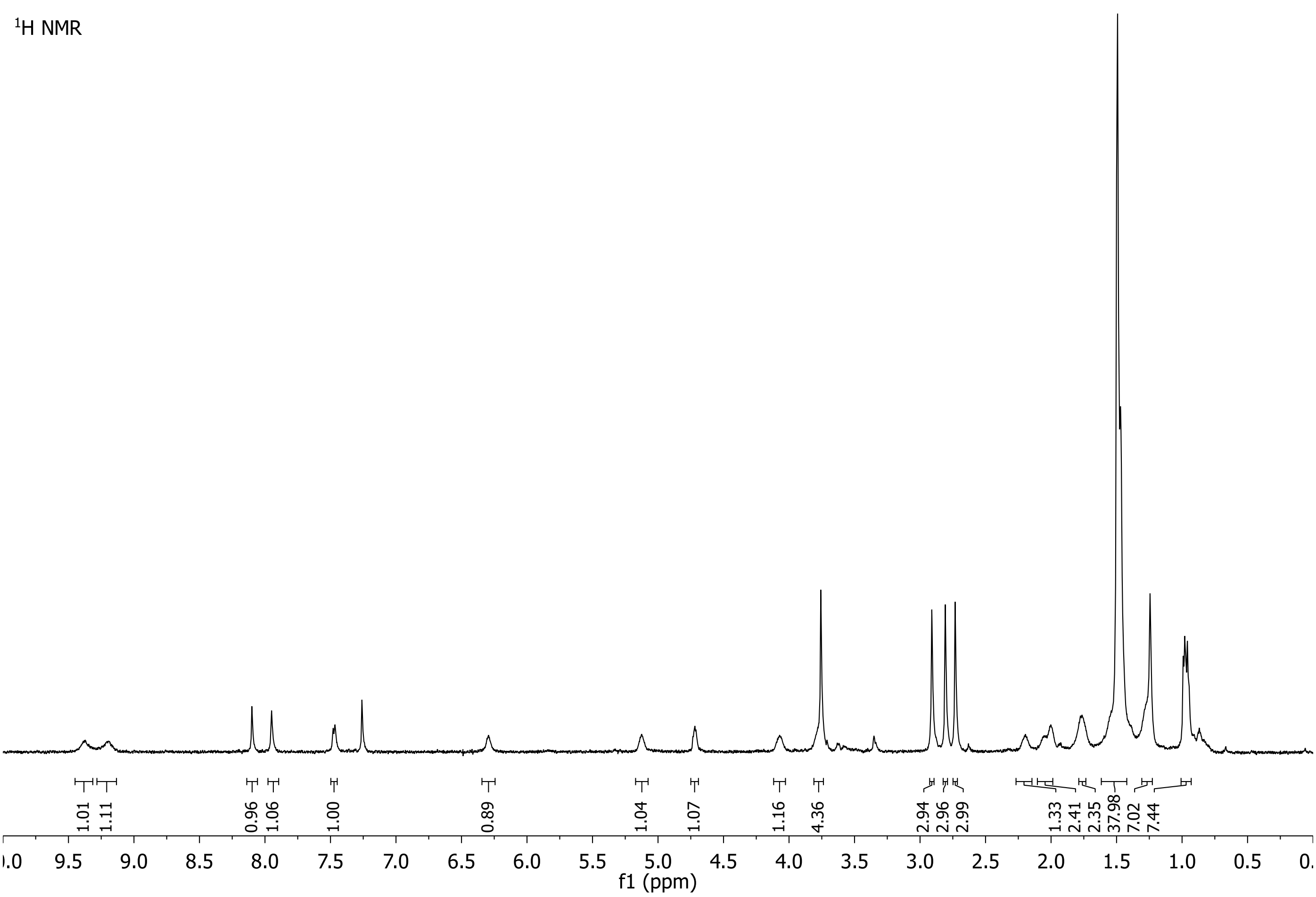

CDCl$_3$, 600 MHz

2b

^{13}C NMR

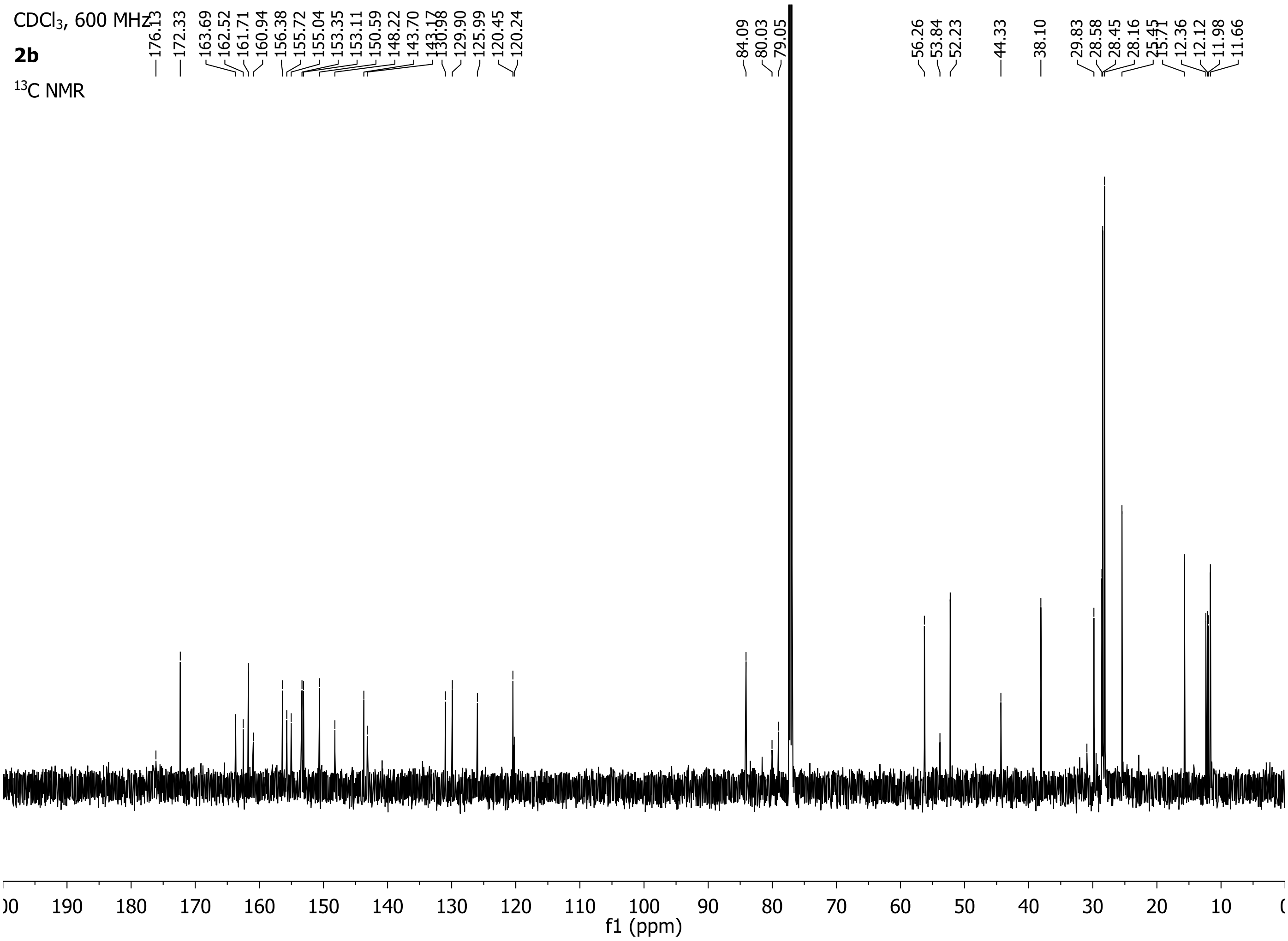

CDCl$_3$, 600 MHz

2b

DEPT-135

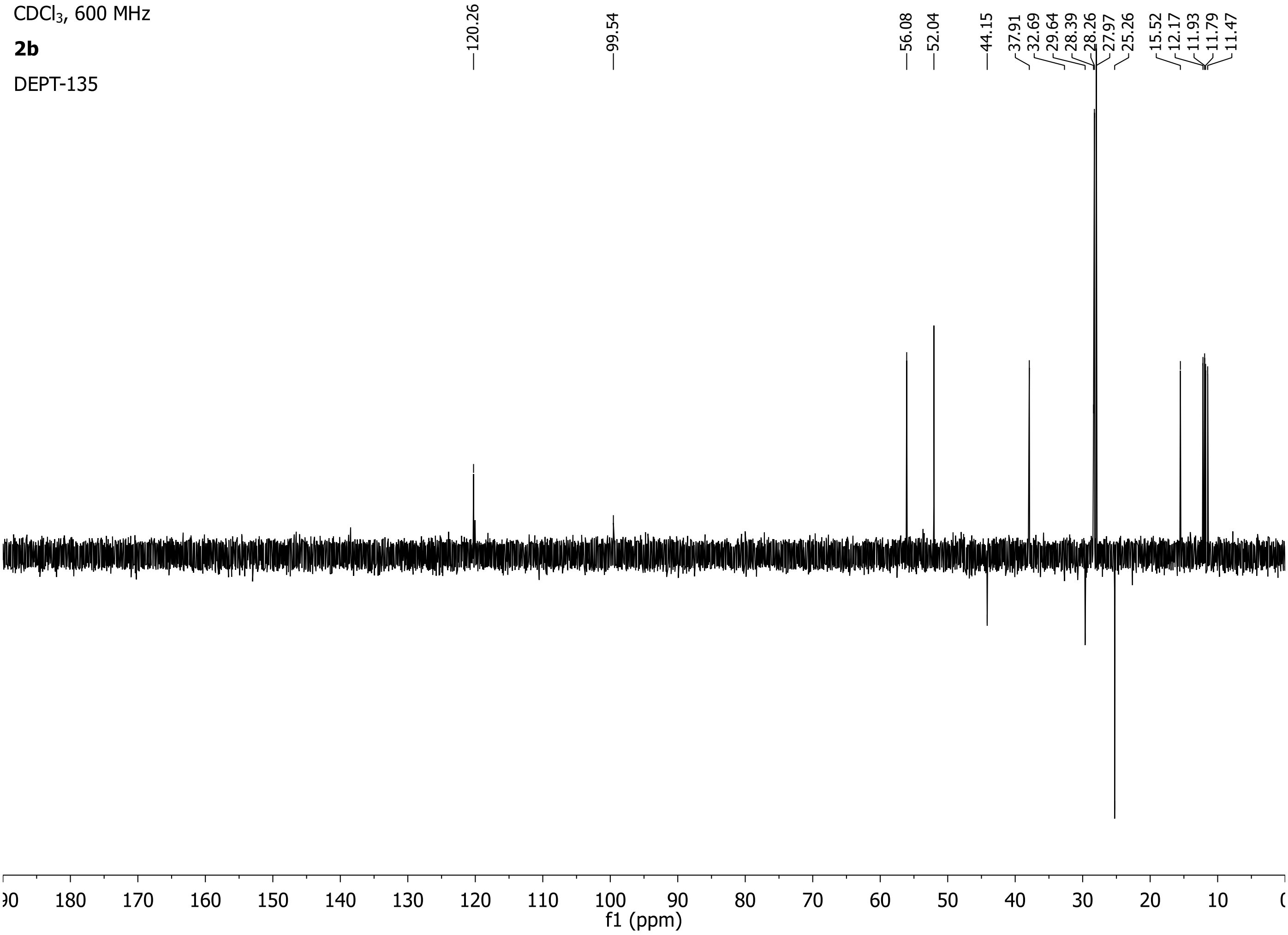

CDCl$_3$, 600 MHz

2b

COSY

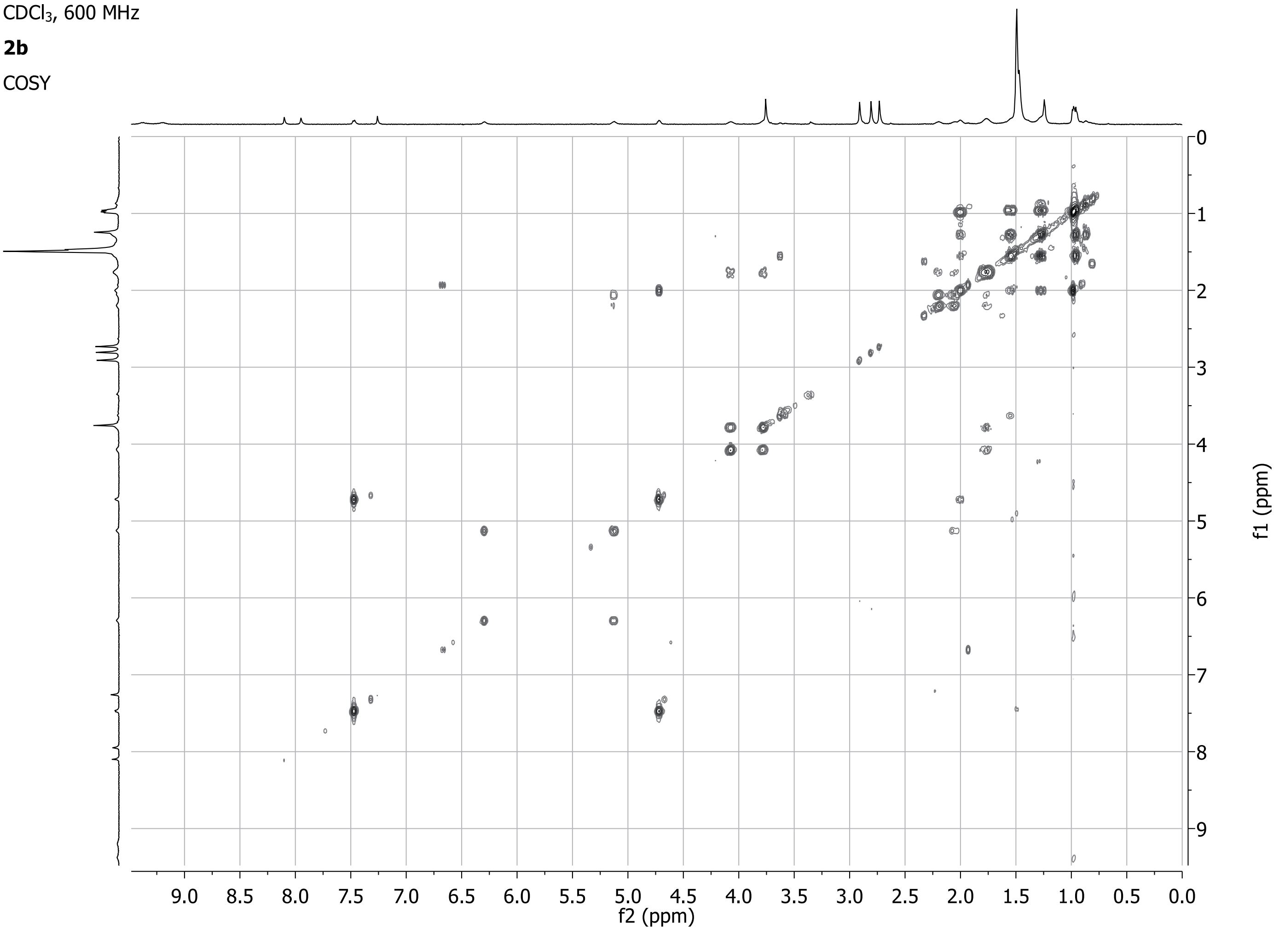

CDCl$_3$, 600 MHz

2b

HMQC

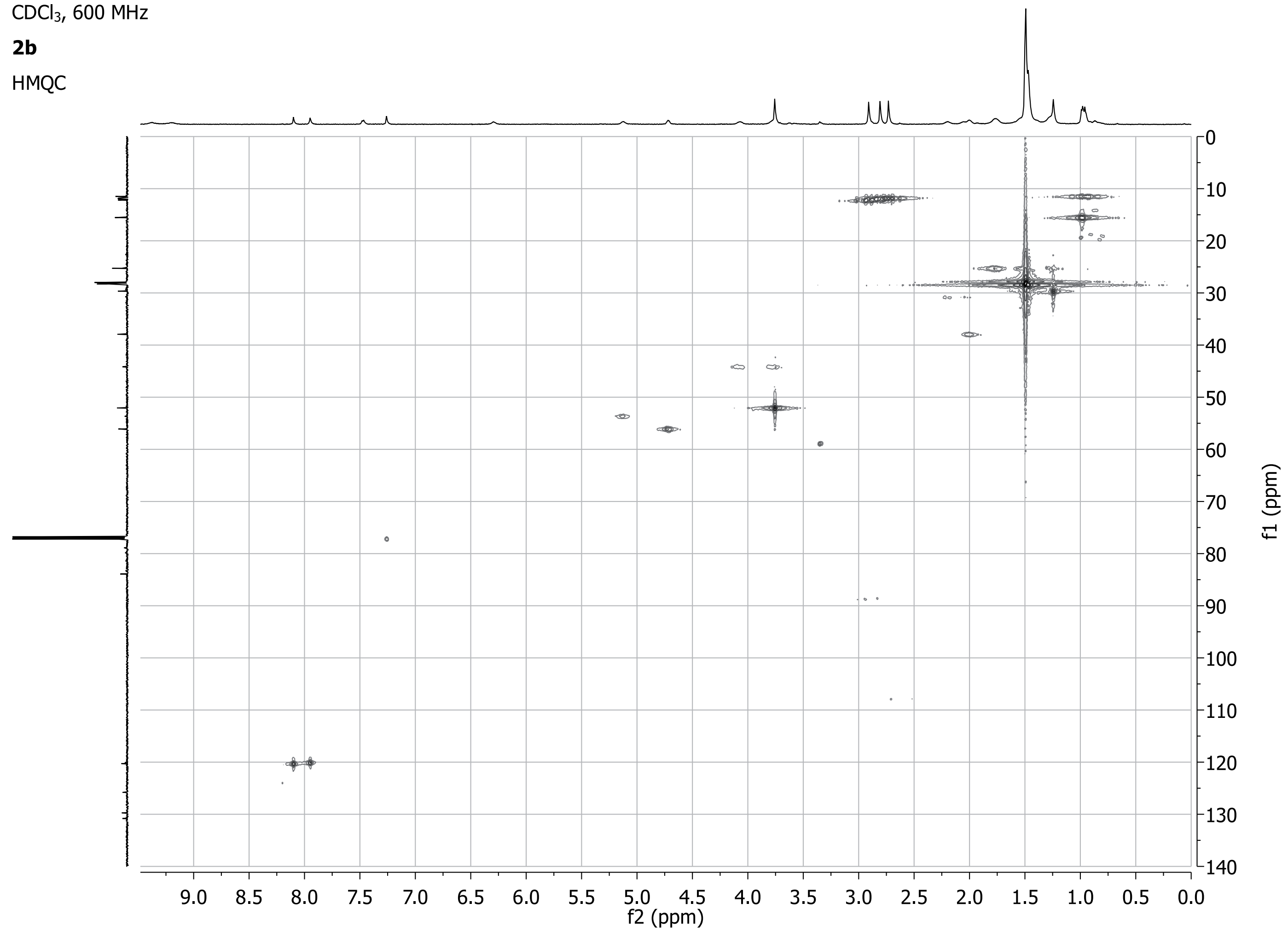

CDCl$_3$, 600 MHz

2b

HMBC

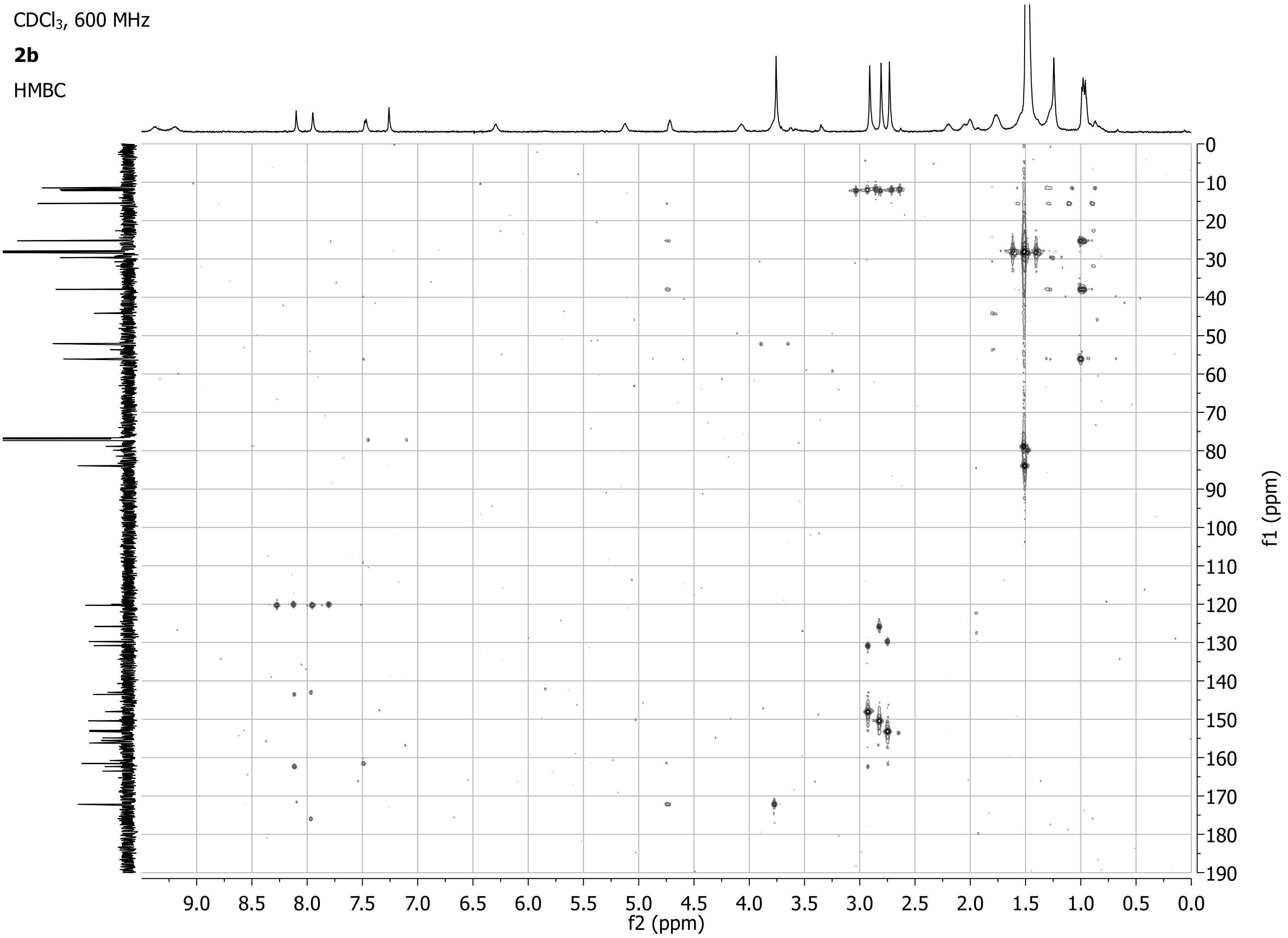

CDCl$_3$, 500 MHz

31

^{1}H NMR

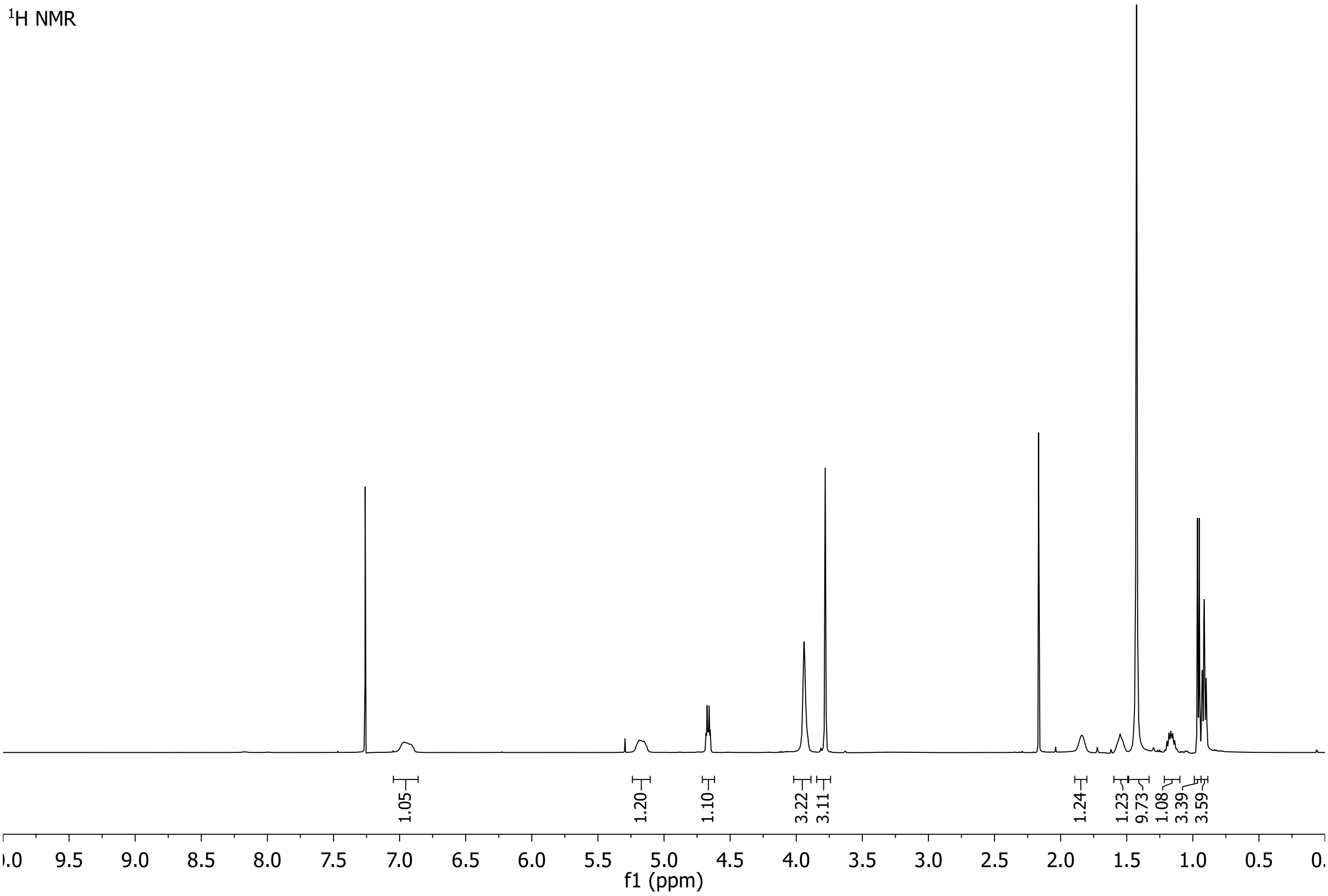

CDCl$_3$, 500 MHz

31

^{13}C NMR

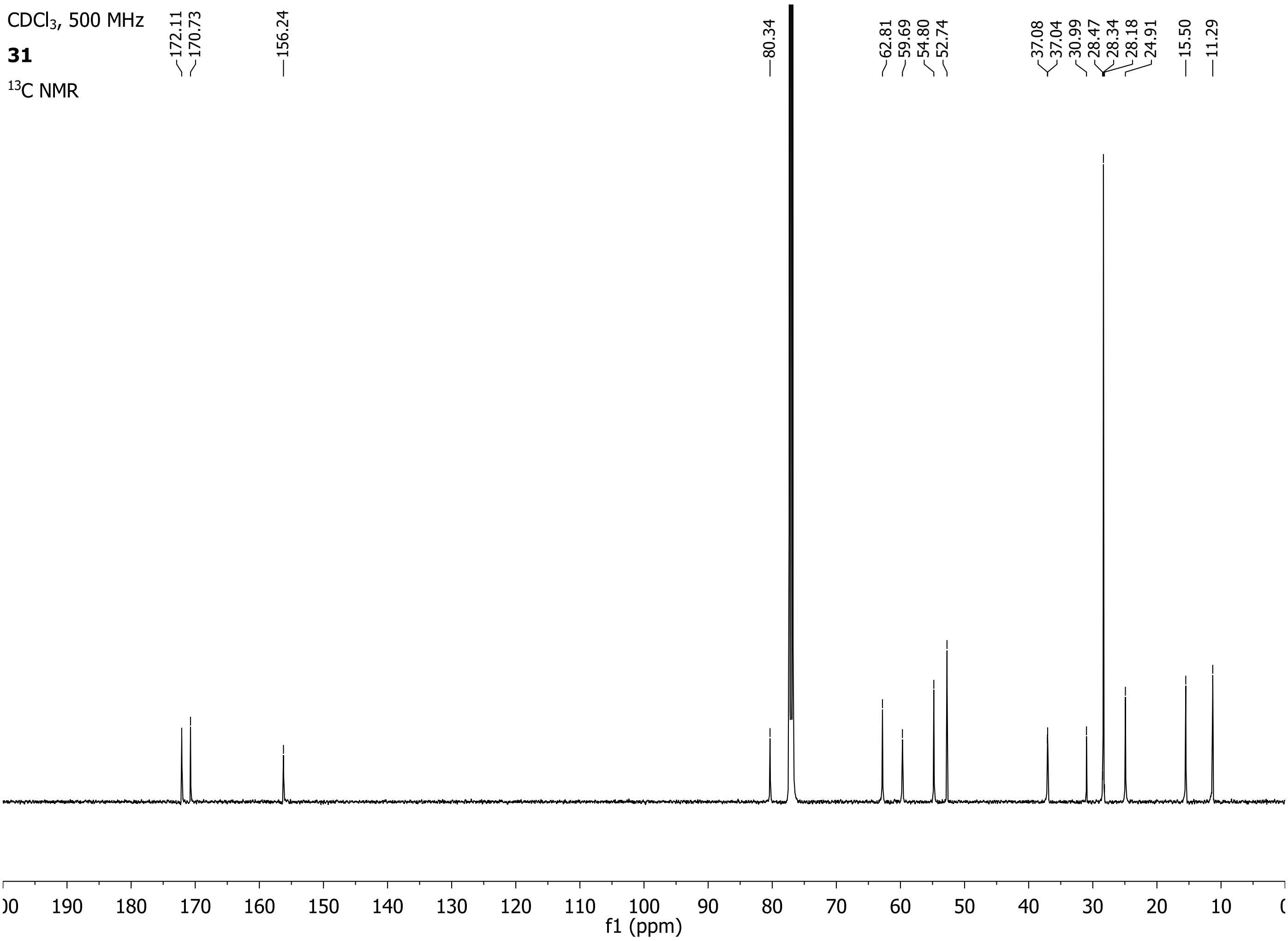

CDCl$_3$, 400 MHz

32

^{1}H NMR

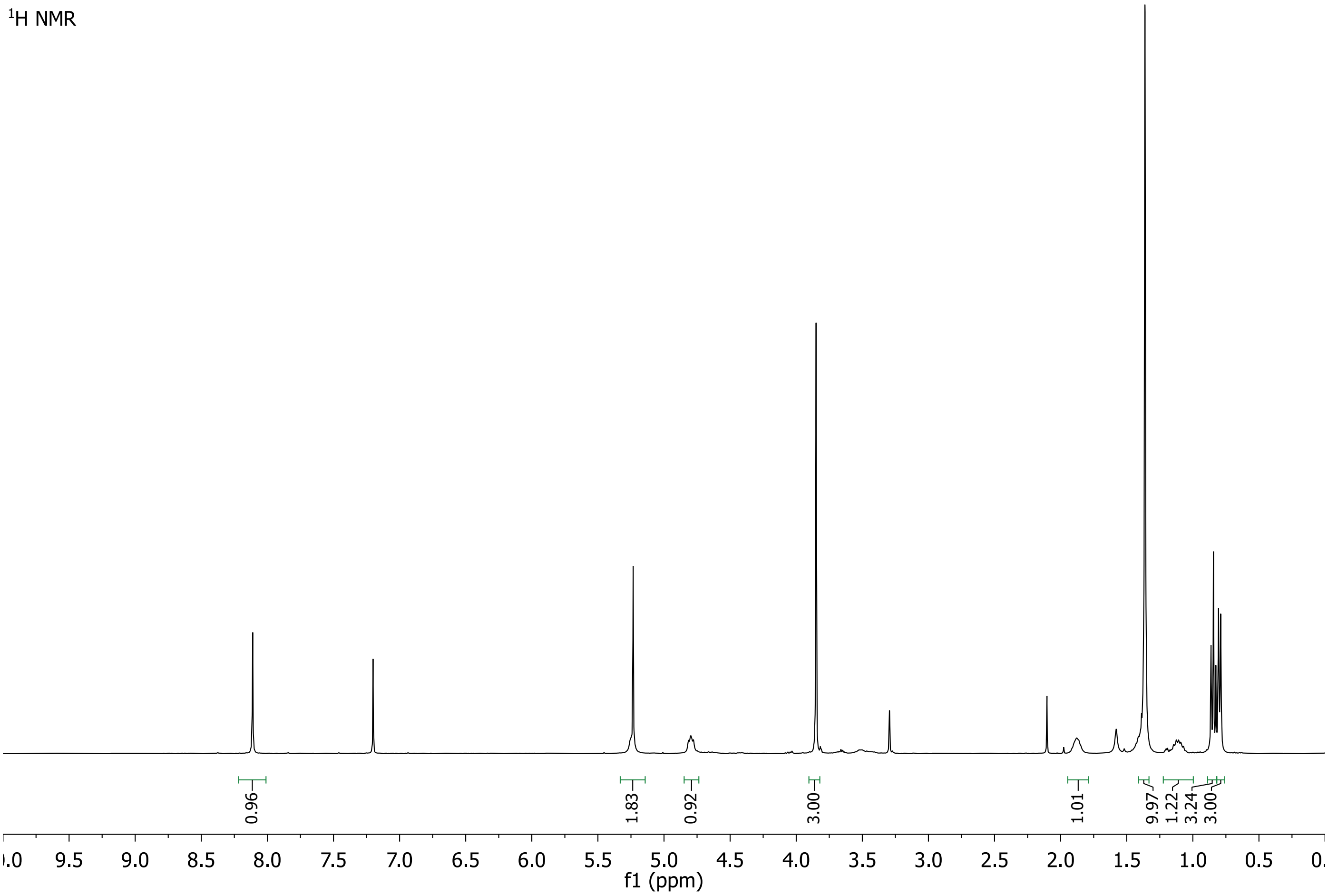

CDCl$_3$, 400 MHz

32

^{13}C NMR

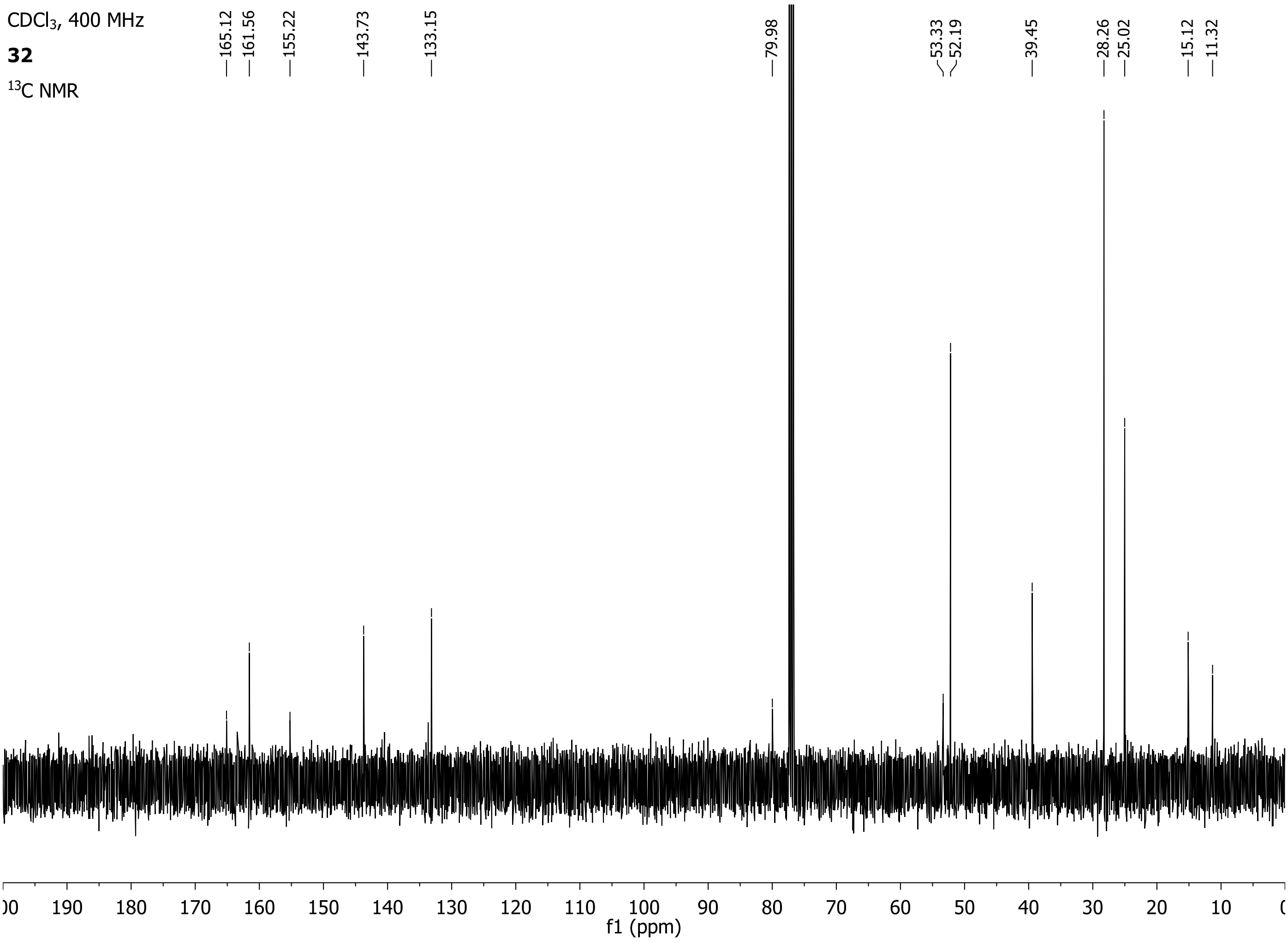

CDCl$_3$, 500 MHz

34

^{1}H NMR

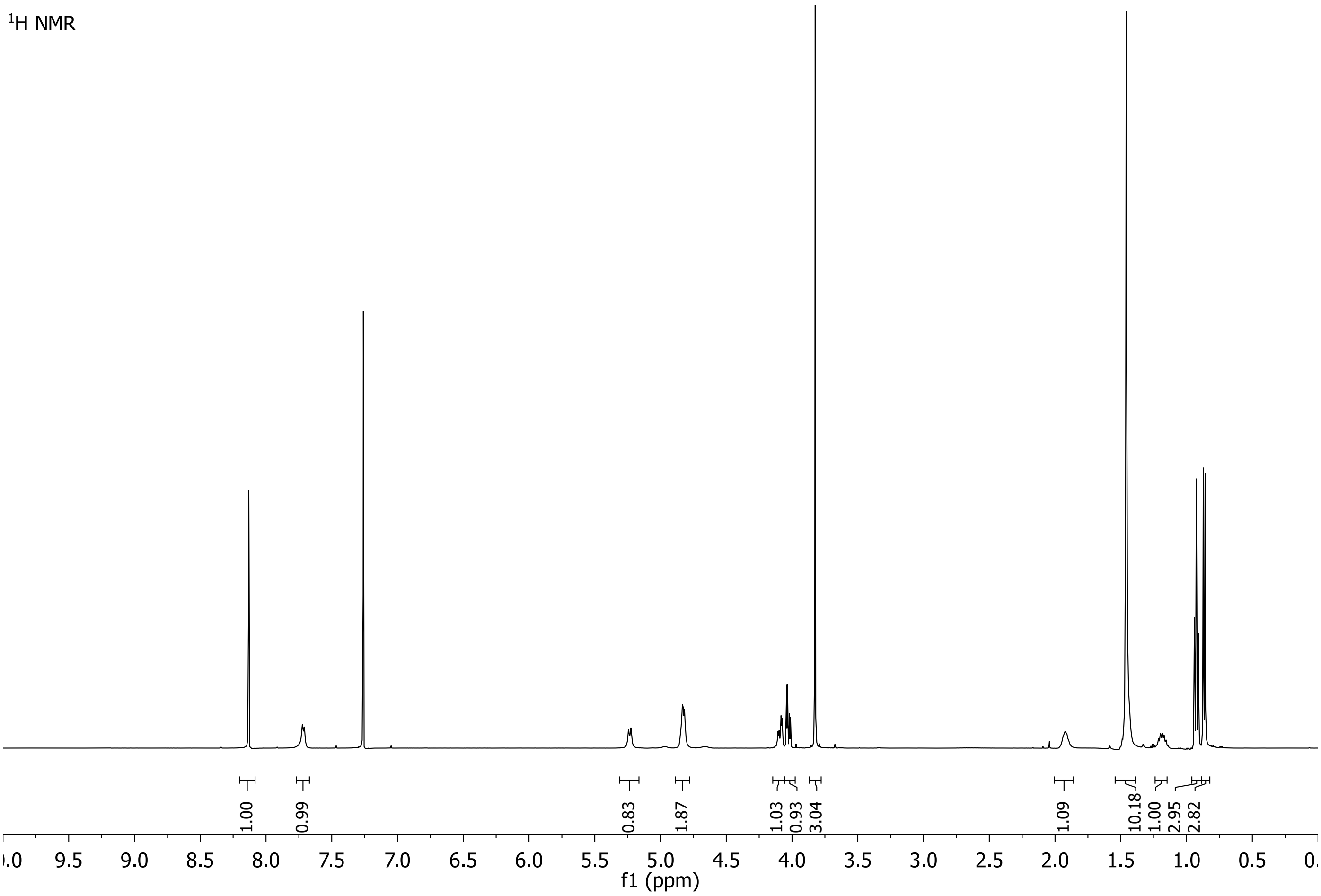

CDCl$_3$, 500 MHz

34

^{13}C NMR

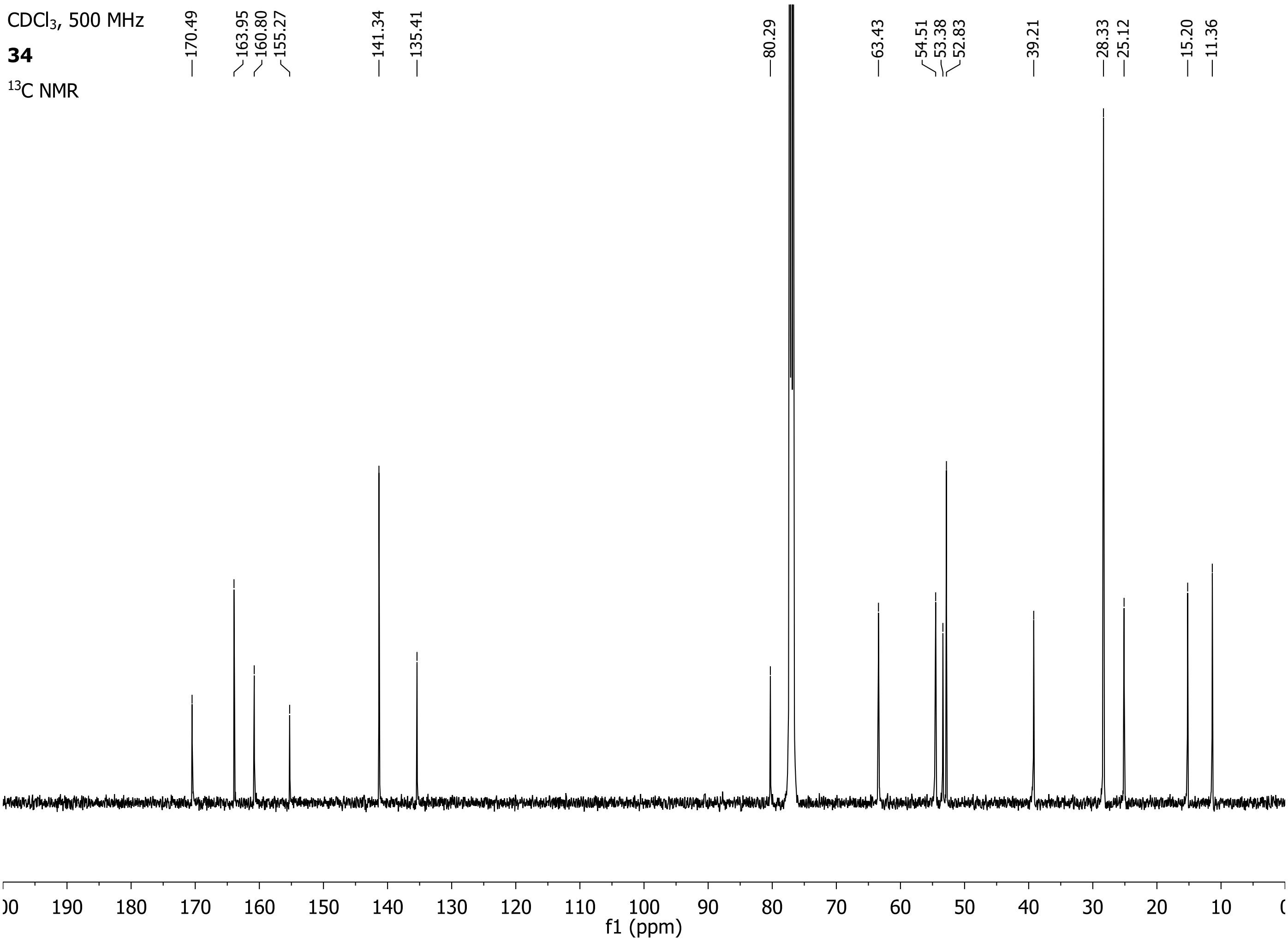

CDCl$_3$, 500 MHz

34

^{13}C NMR

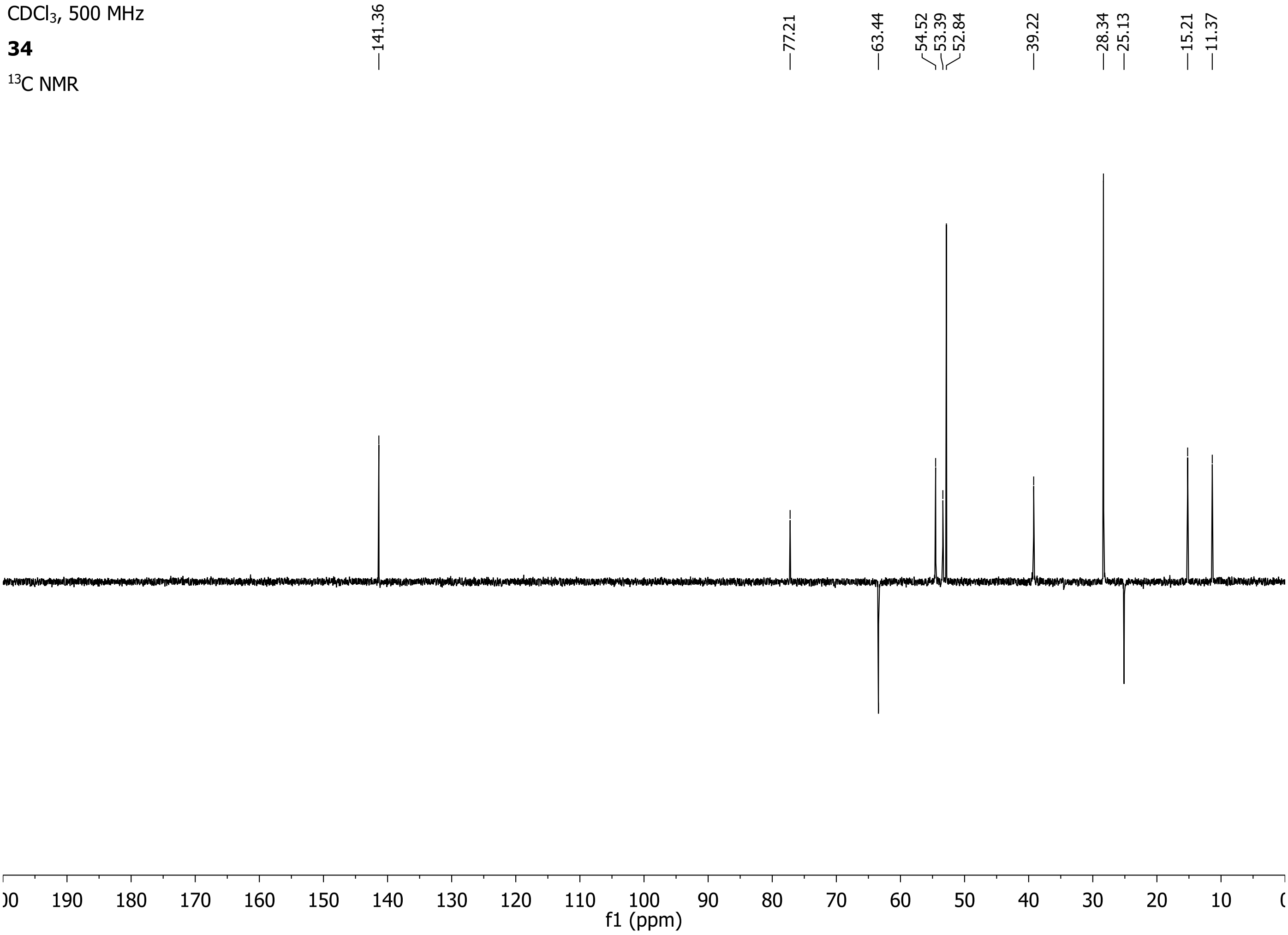

CDCl$_3$, 500 MHz

34

COSY

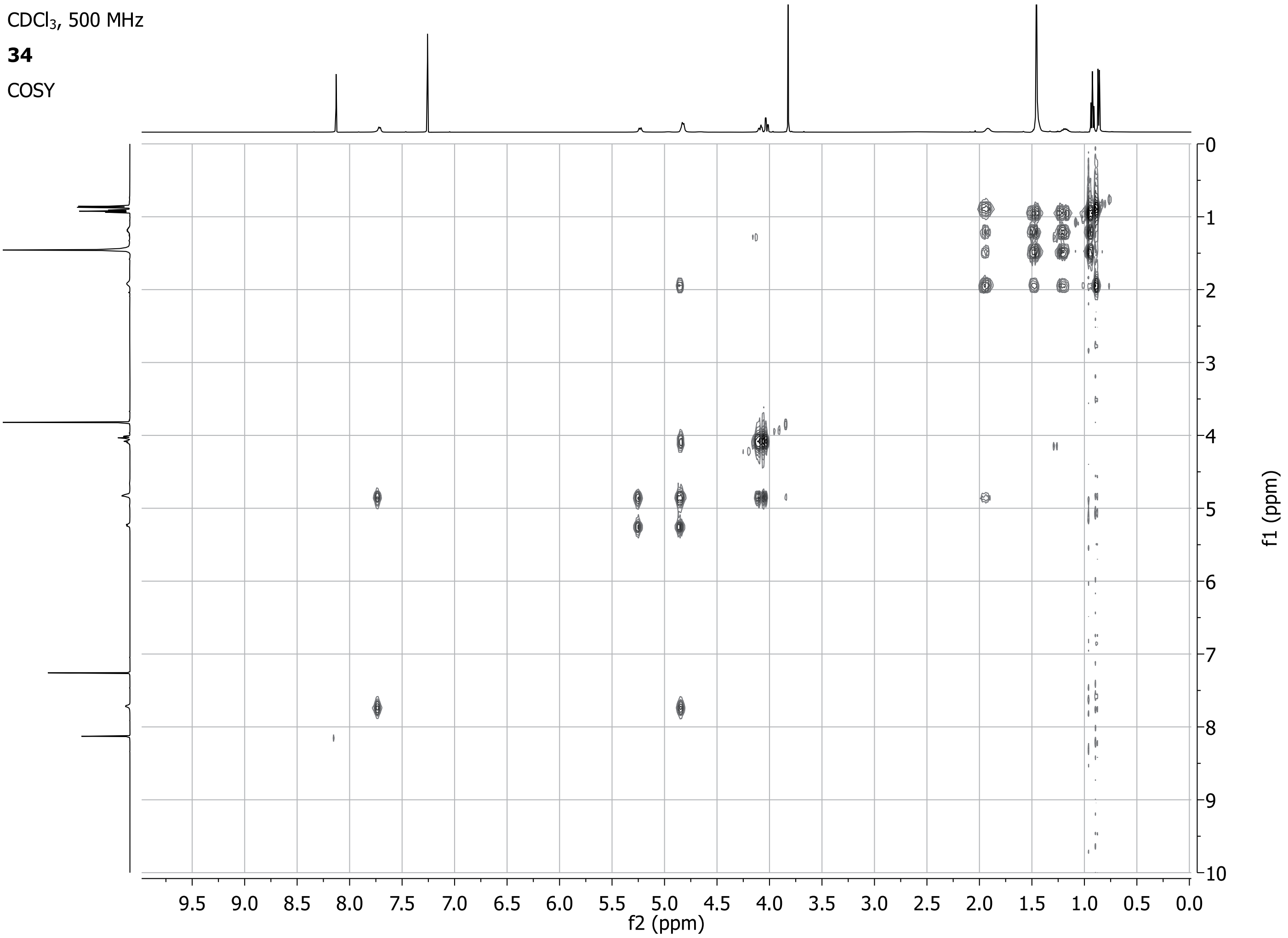

CDCl$_3$, 500 MHz

34

HSQC

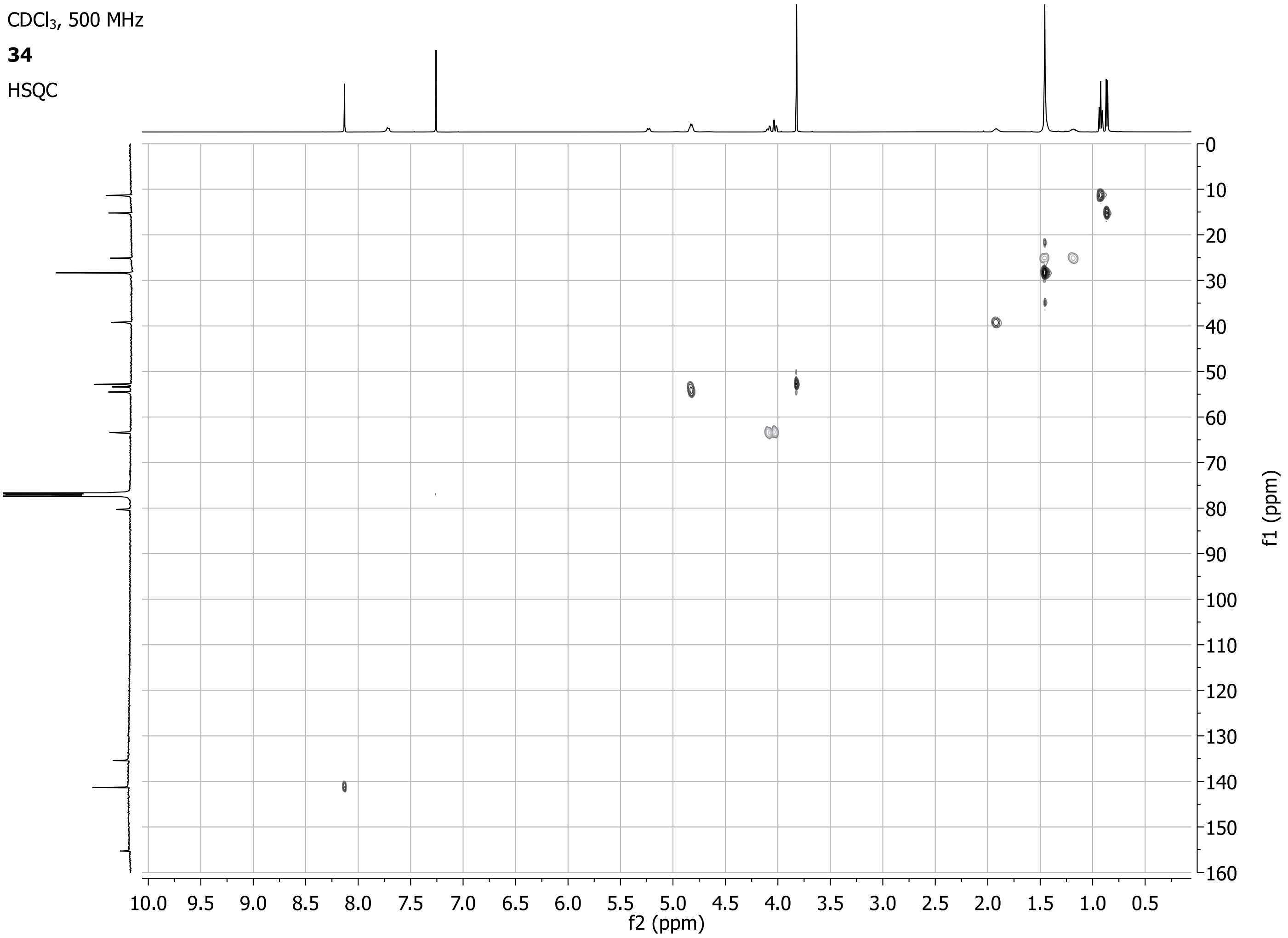

CDCl$_3$, 500 MHz

34

HMBC

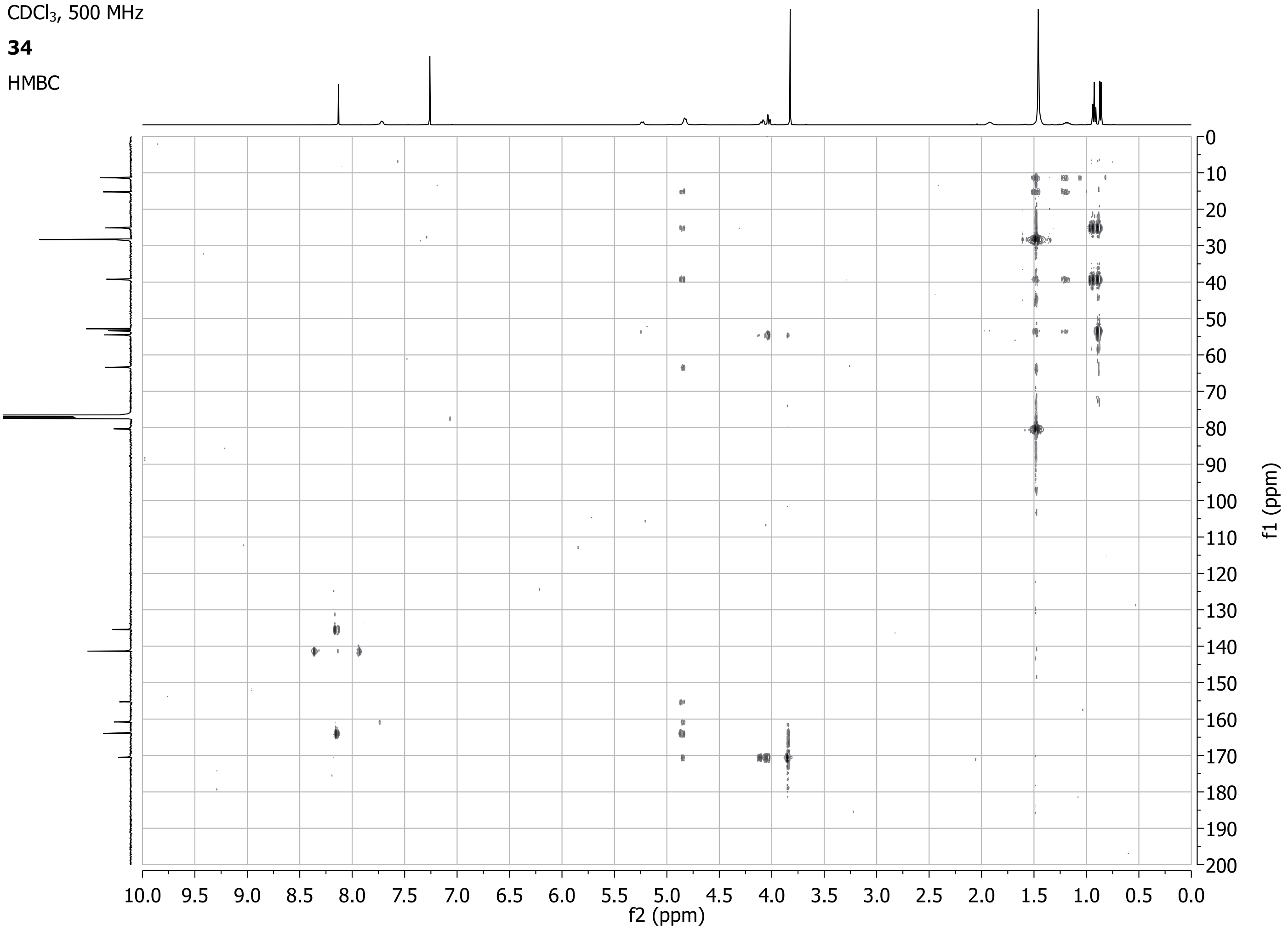

CDCl3, 500 MHz

10

^{1}H NMR

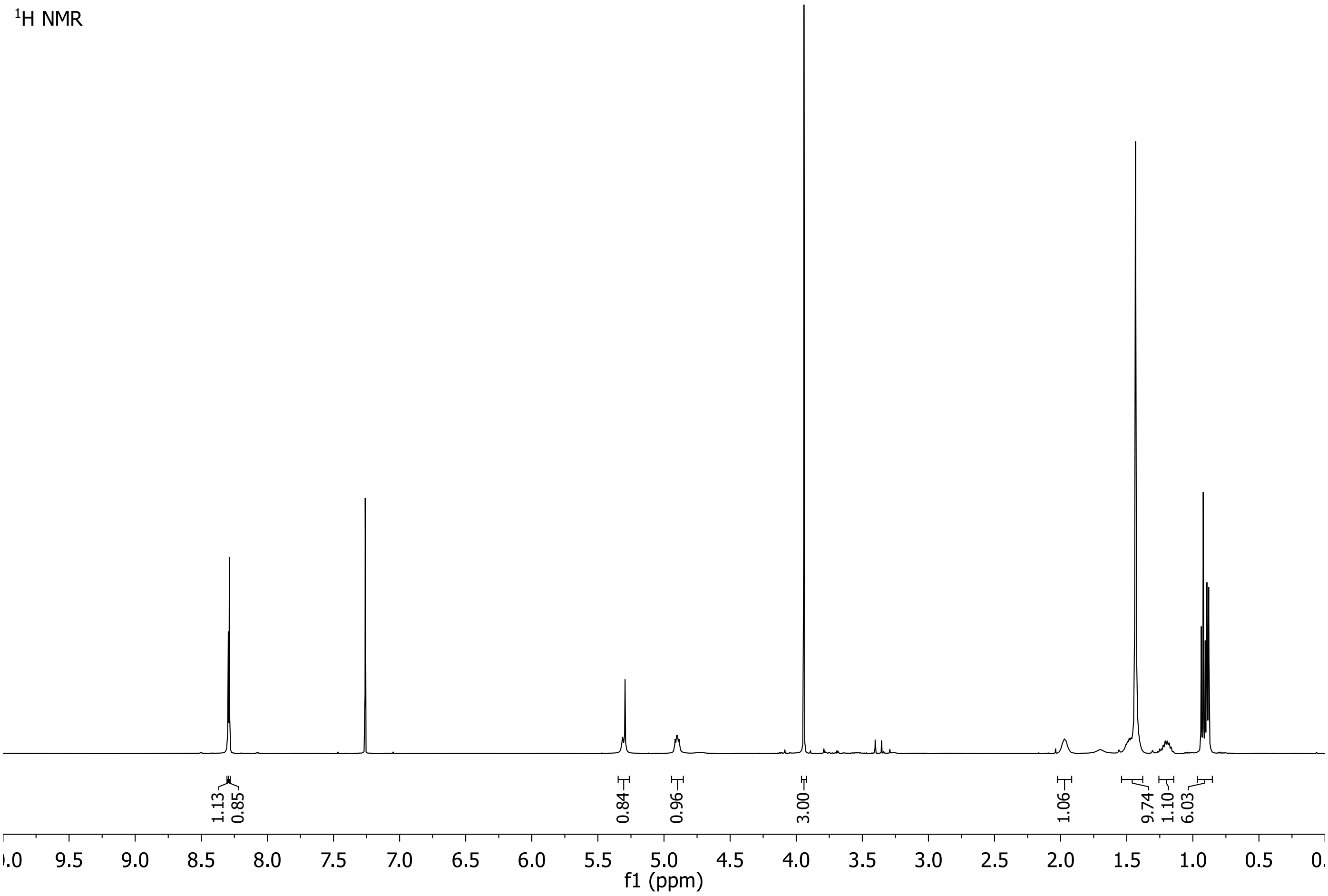

CDCl3, 500 MHz

10

^{13}C NMR

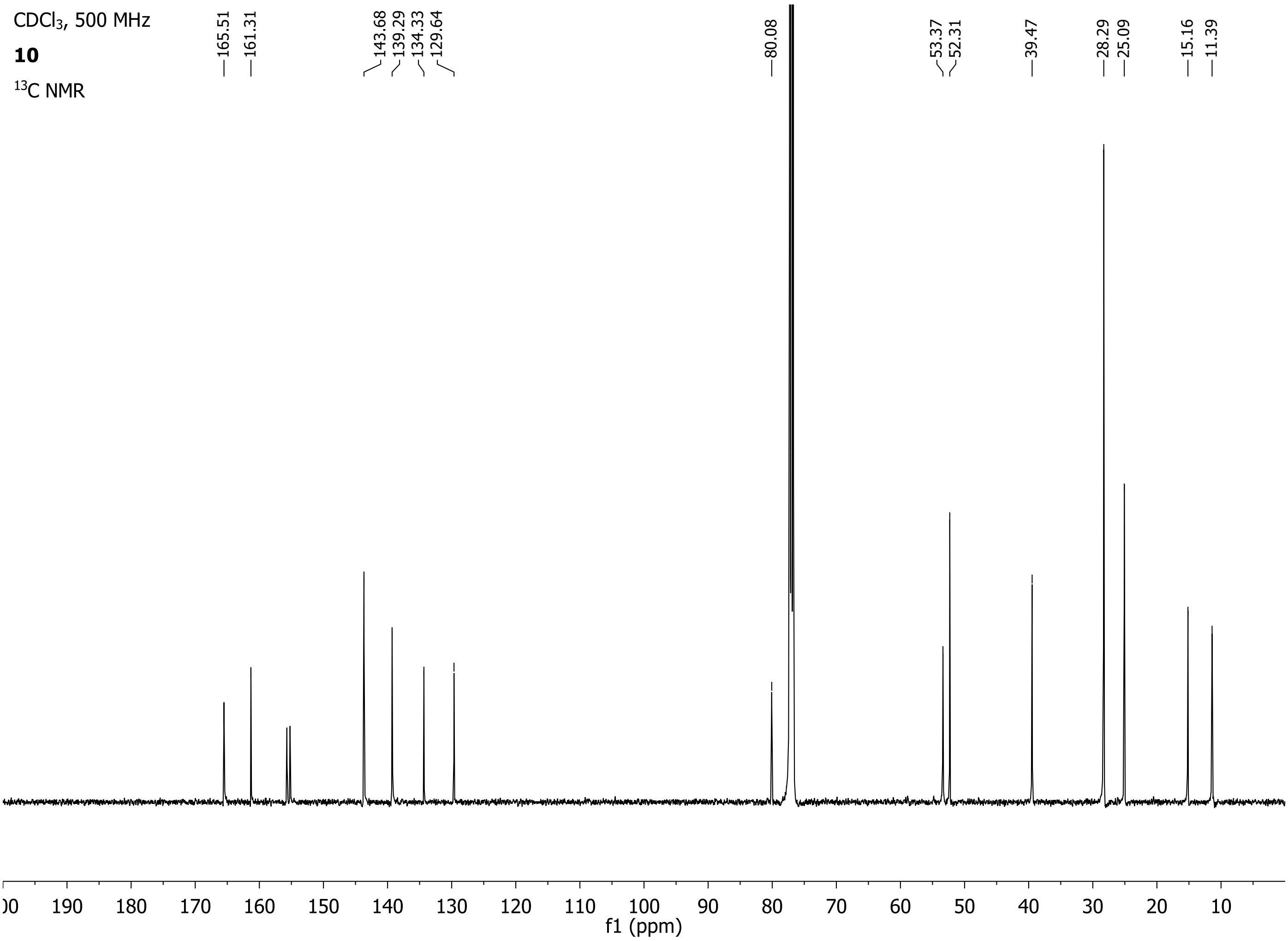

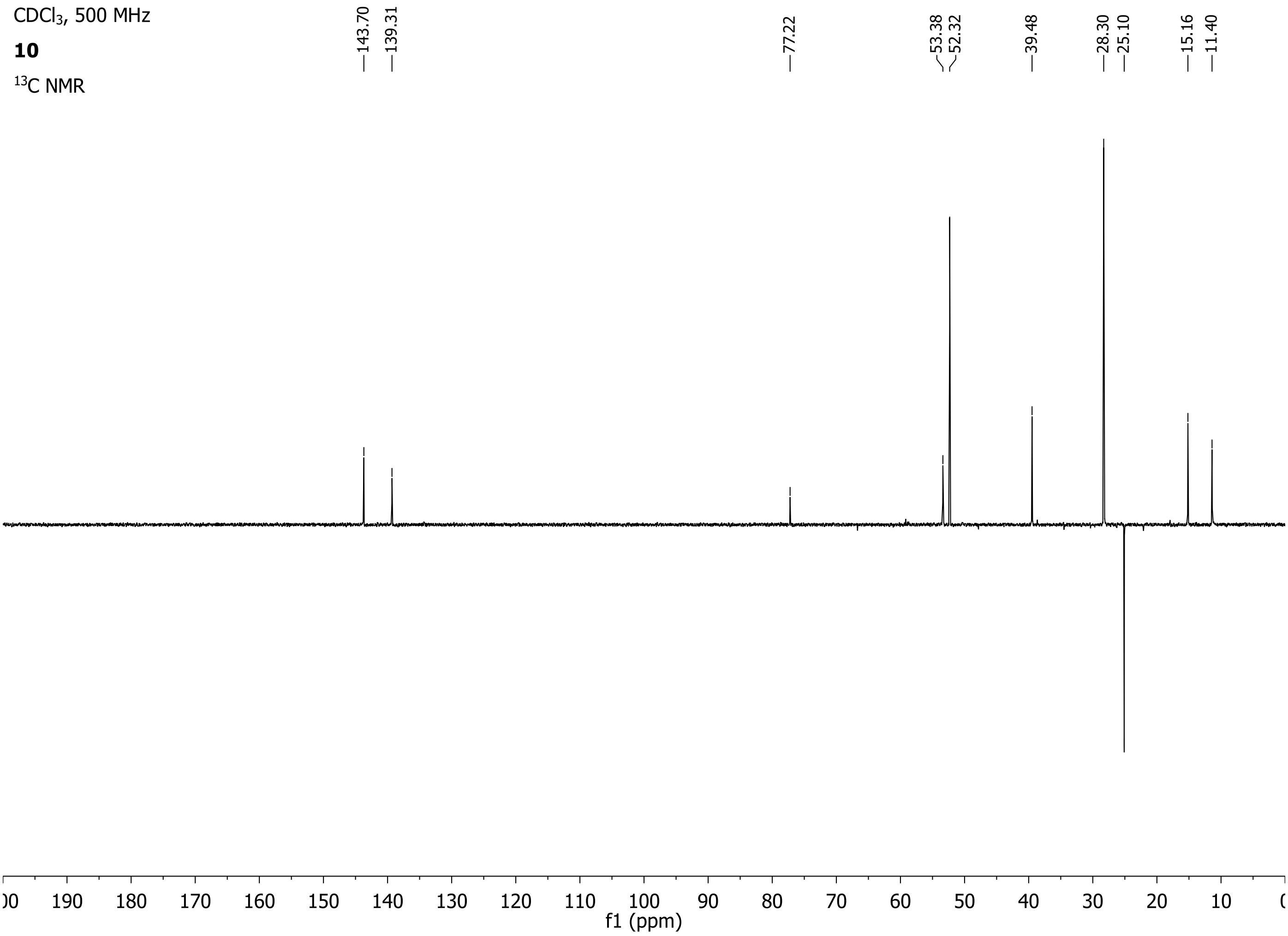
CDCl3, 500 MHz
10
13C NMR
143.70
139.31
77.22
53.38
52.32
39.48
28.30
25.10
15.16
11.40
f1 (ppm)

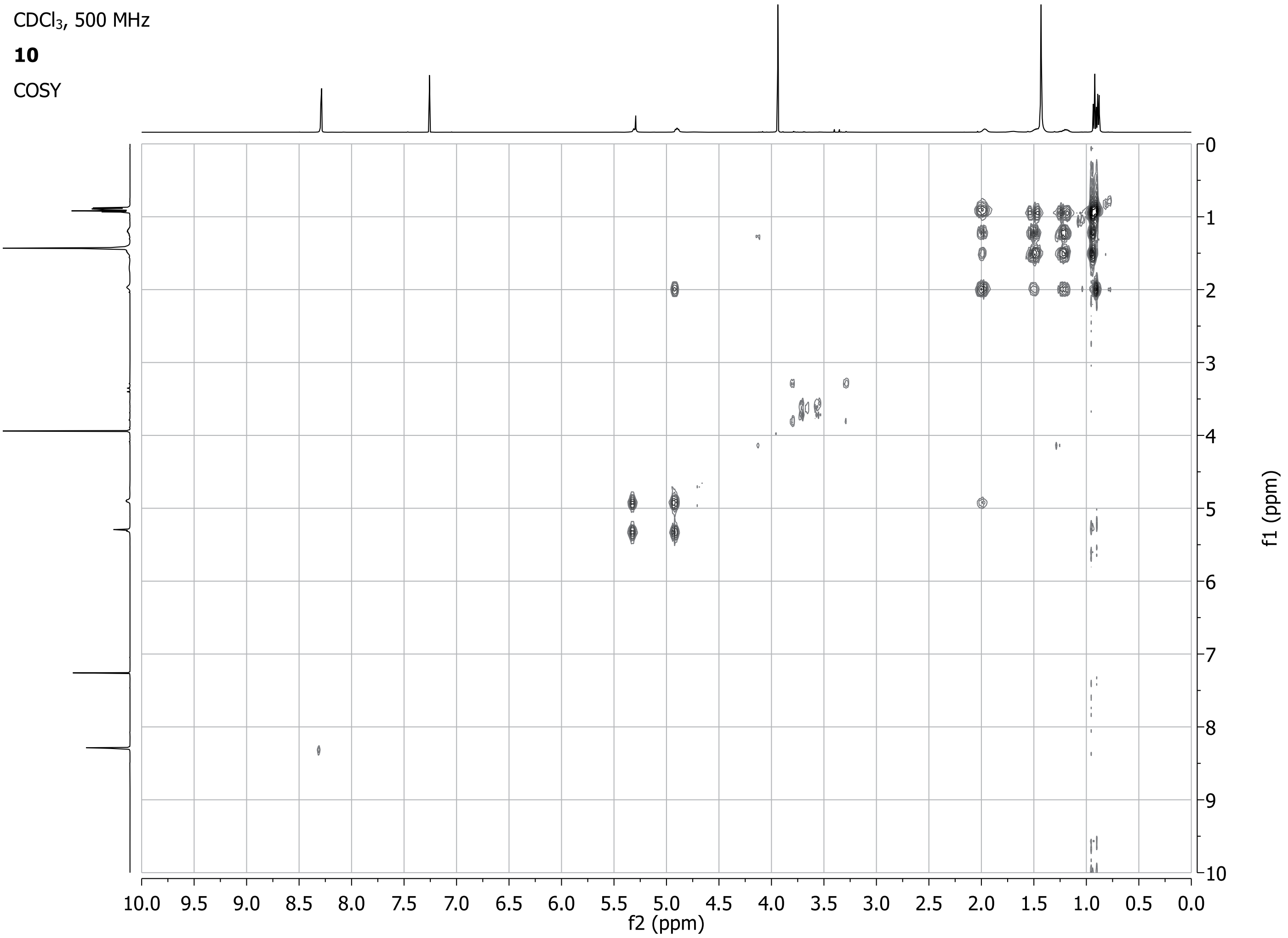
CDCl3, 500 MHz
10
COSY
f2 (ppm)
f1 (ppm)

CDCl$_3$, 500 MHz

10

HSQC

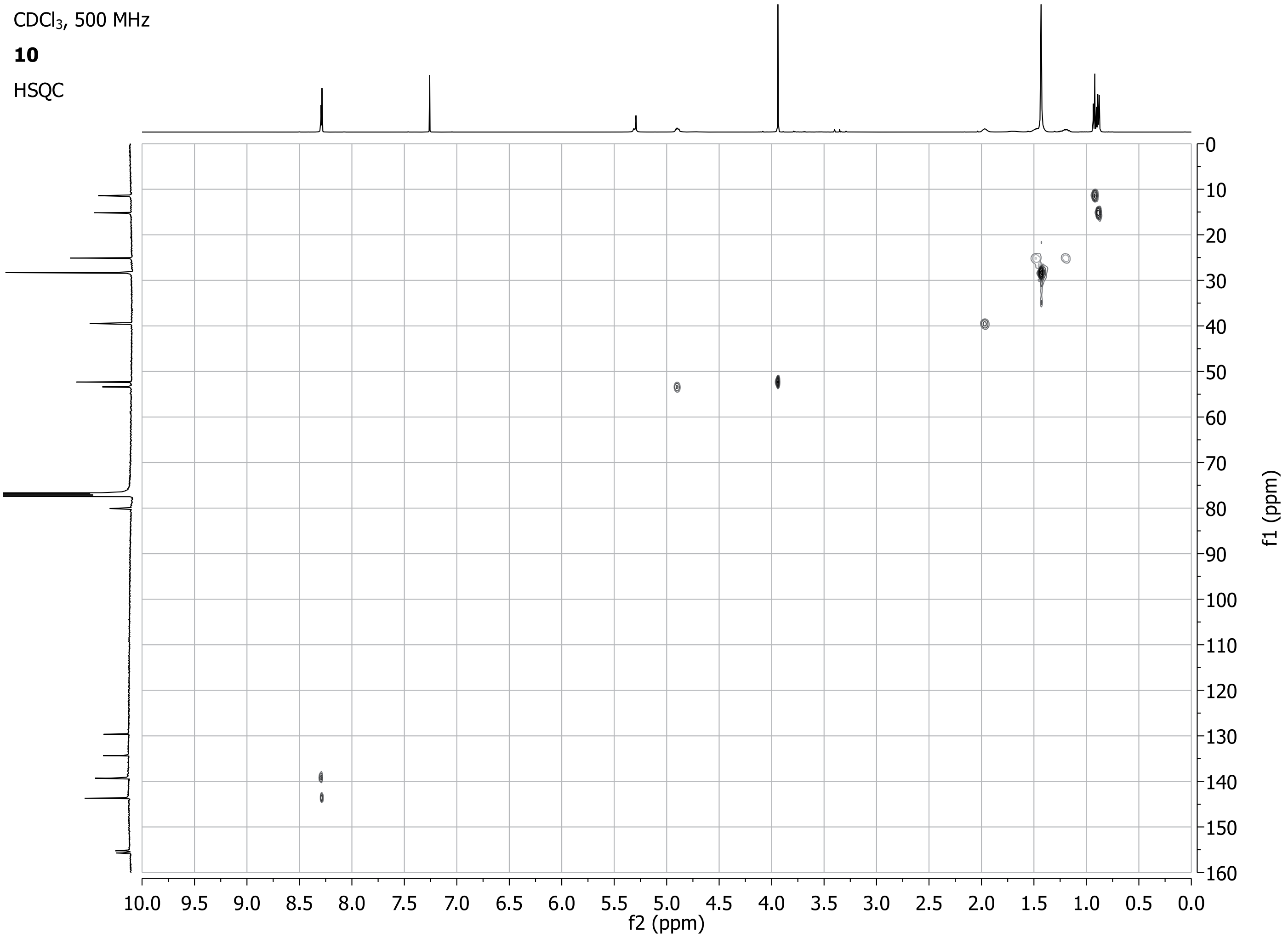

CDCl$_3$, 500 MHz

10

HMQC

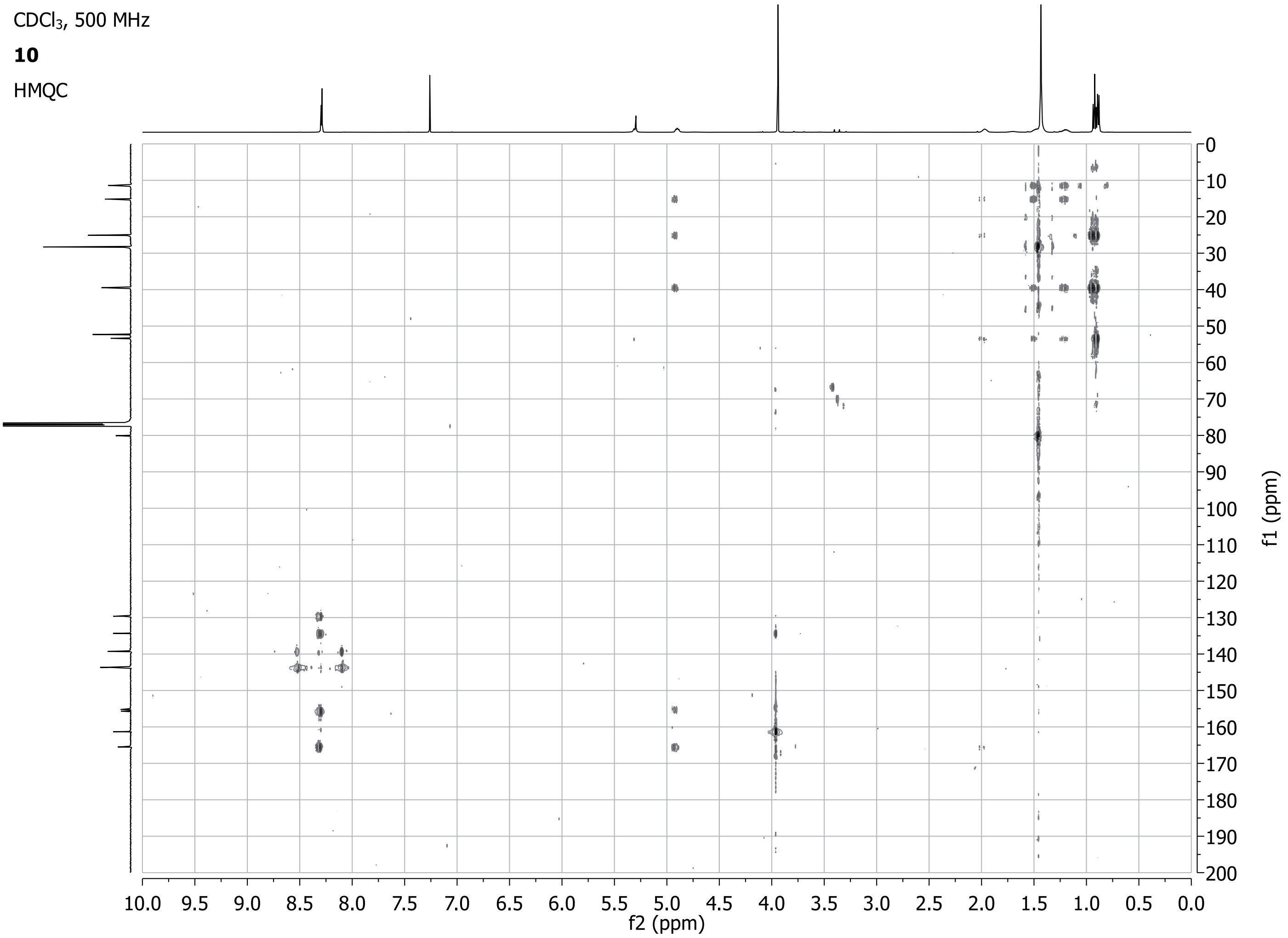

CDCl$_3$, 500 MHz

11

^{1}H NMR

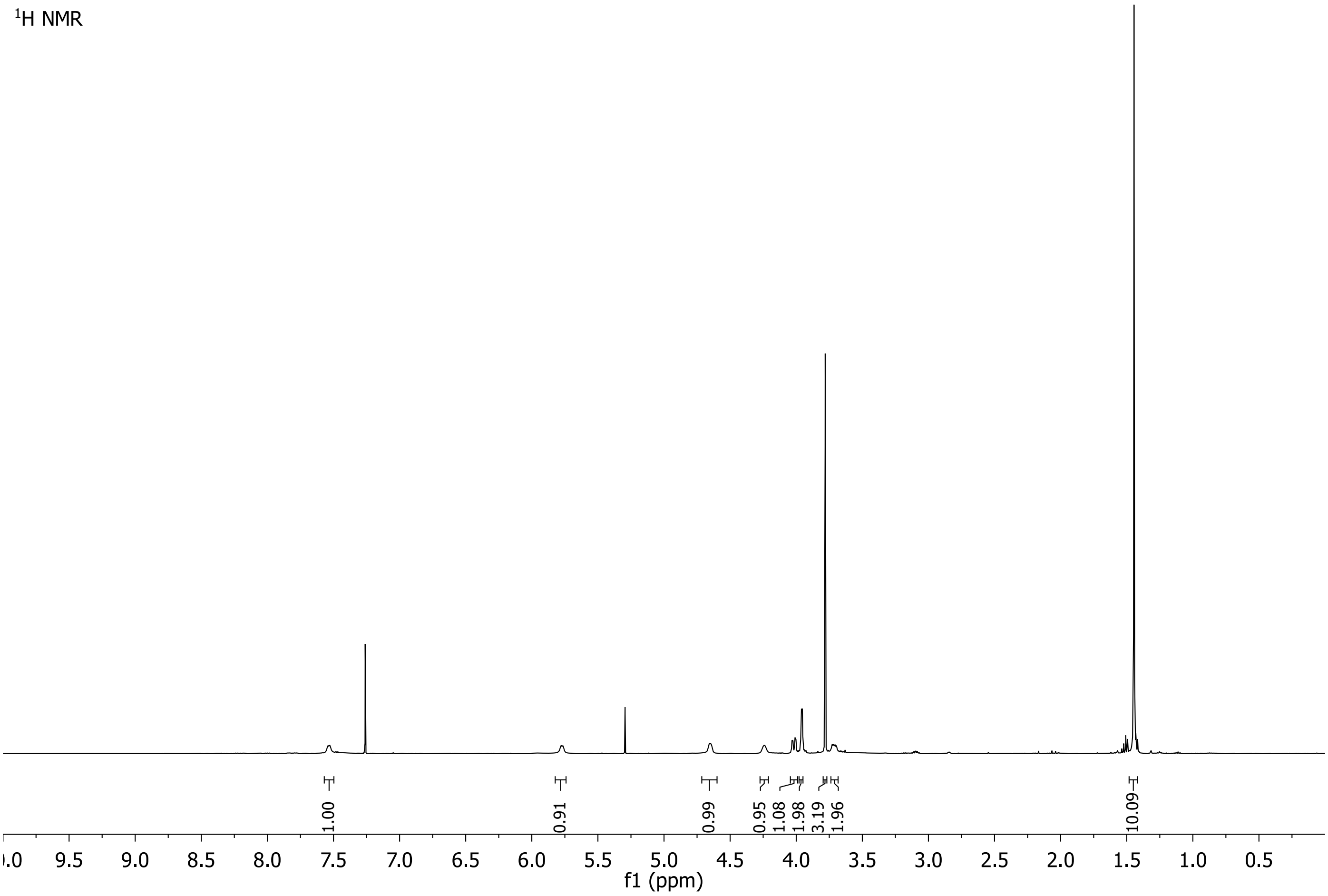

CDCl$_3$, 500 MHz

11

^{13}C NMR

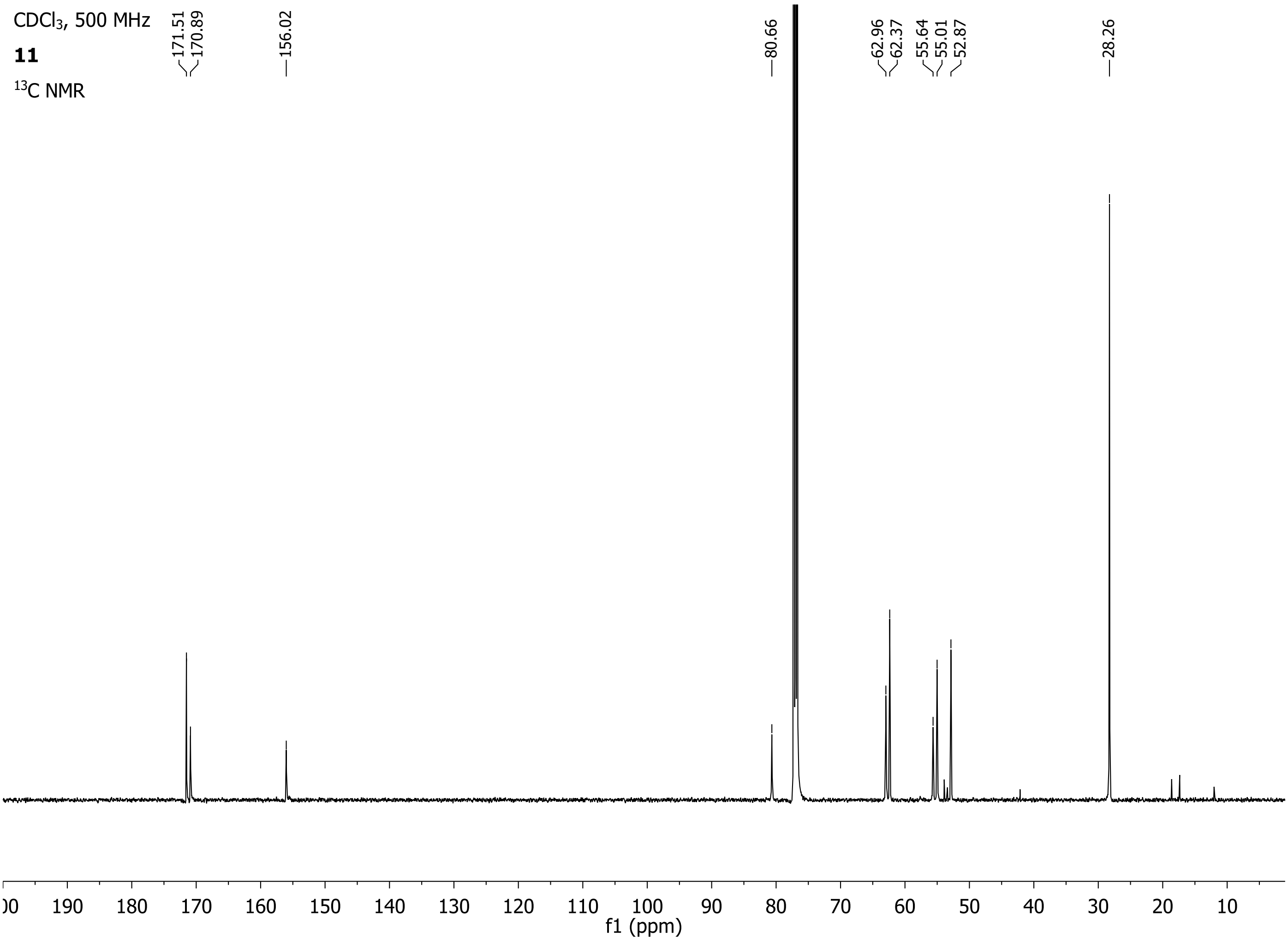

CDCl$_3$, 500 MHz

33

^{13}C NMR

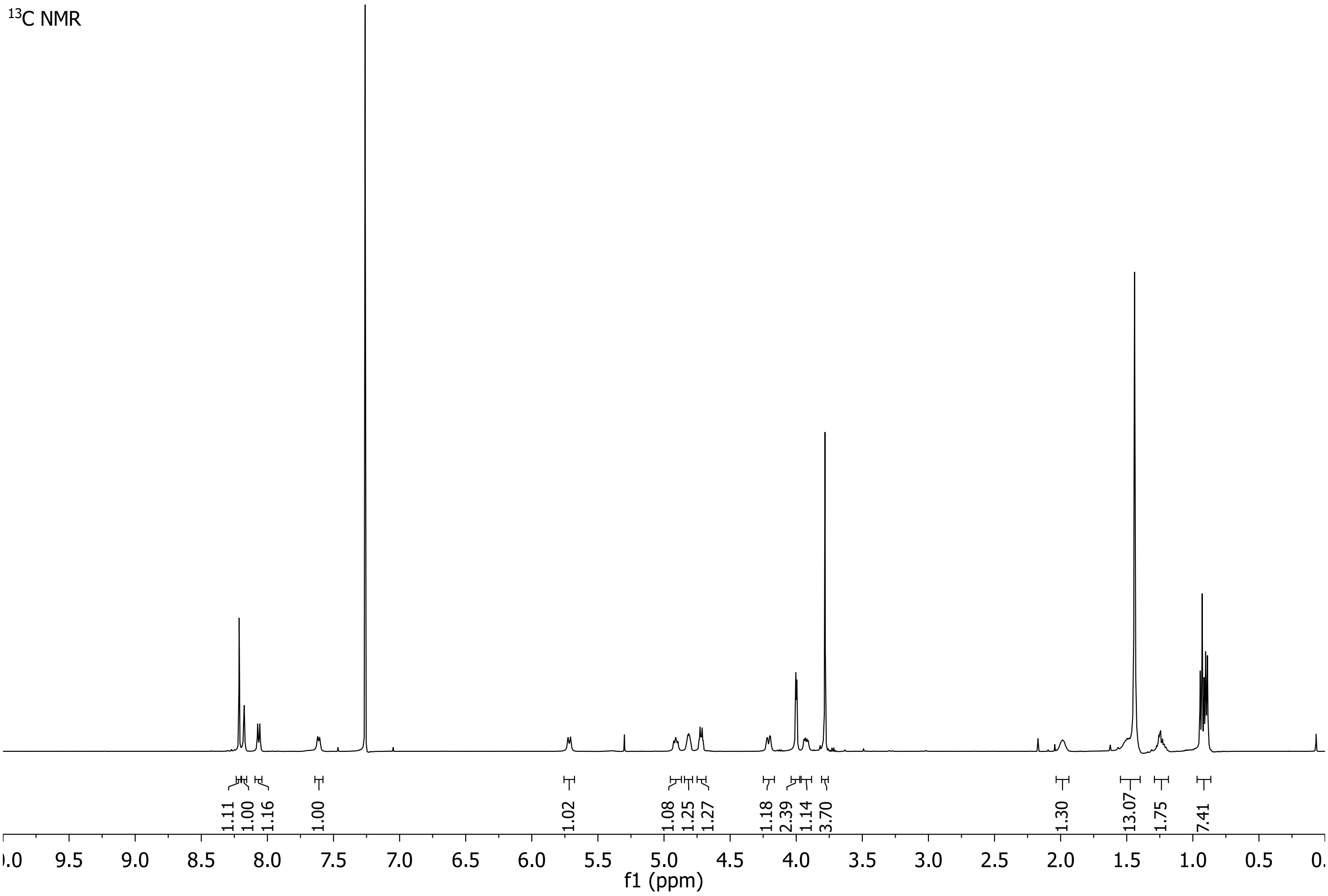

CDCl$_3$, 500 MHz

33

^{13}C NMR

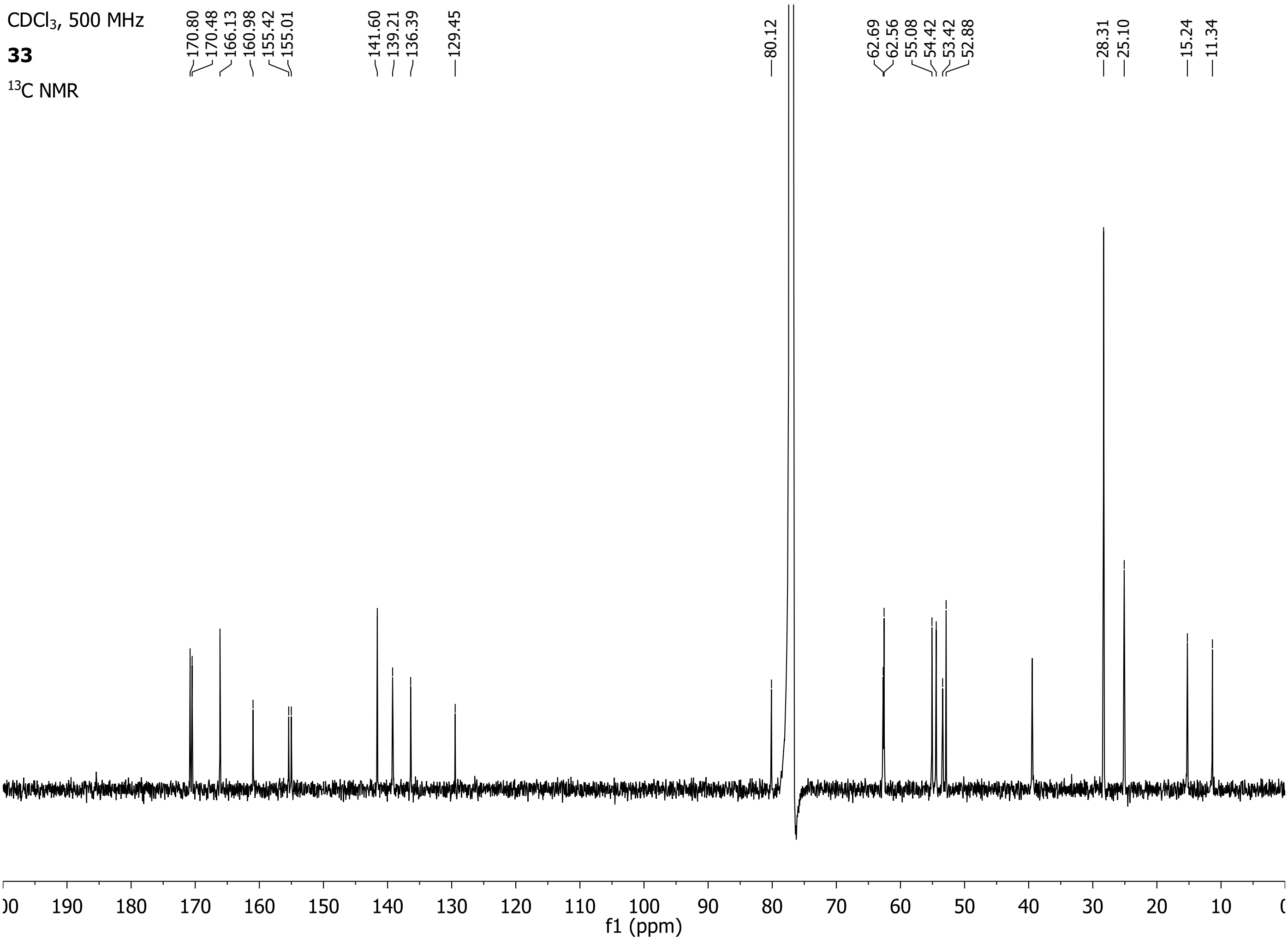

CDCl$_3$, 500 MHz

33

^{13}C NMR

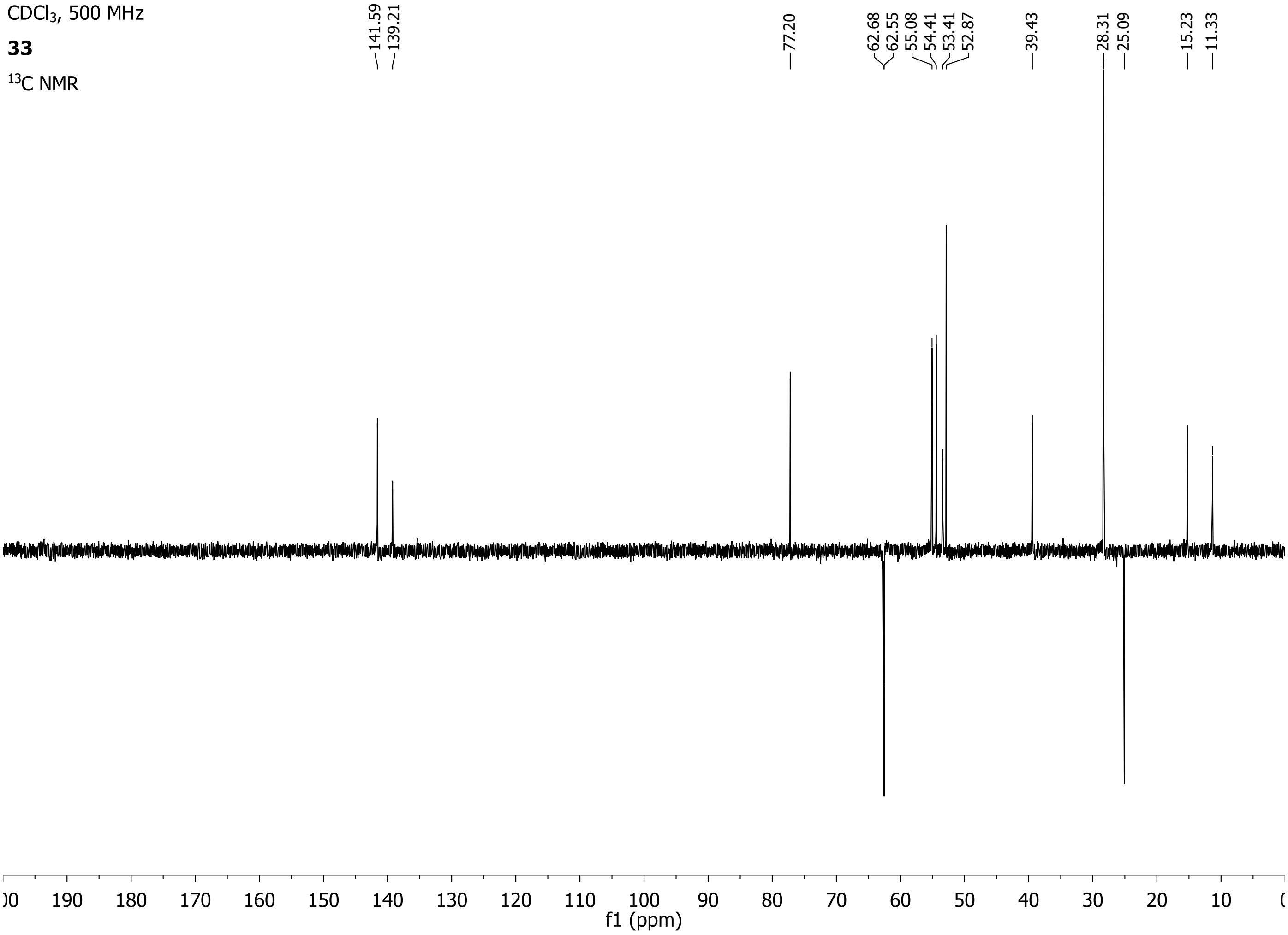

CDCl$_3$, 500 MHz

33

COSY

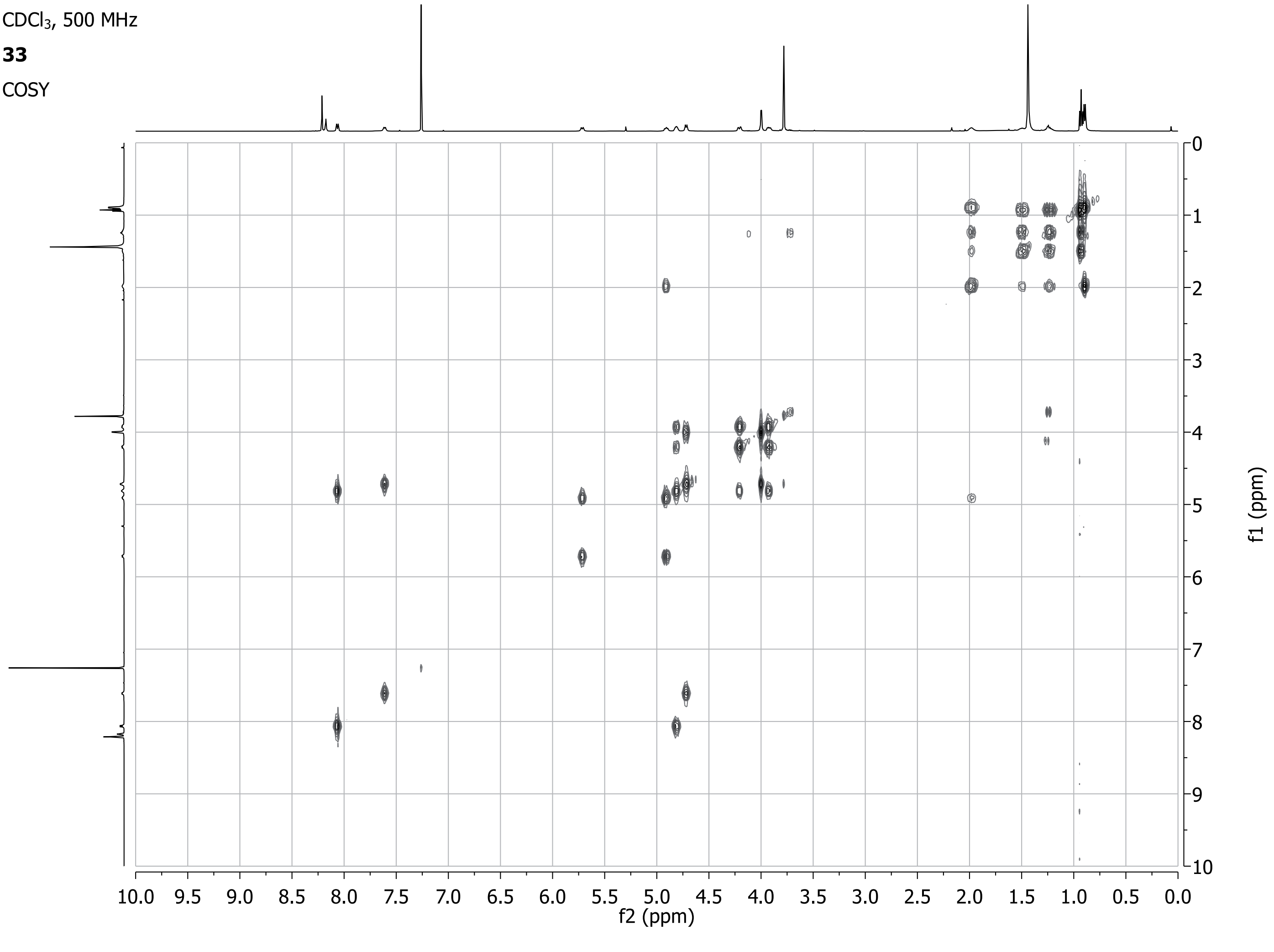

CDCl$_3$, 500 MHz

33

HSQC

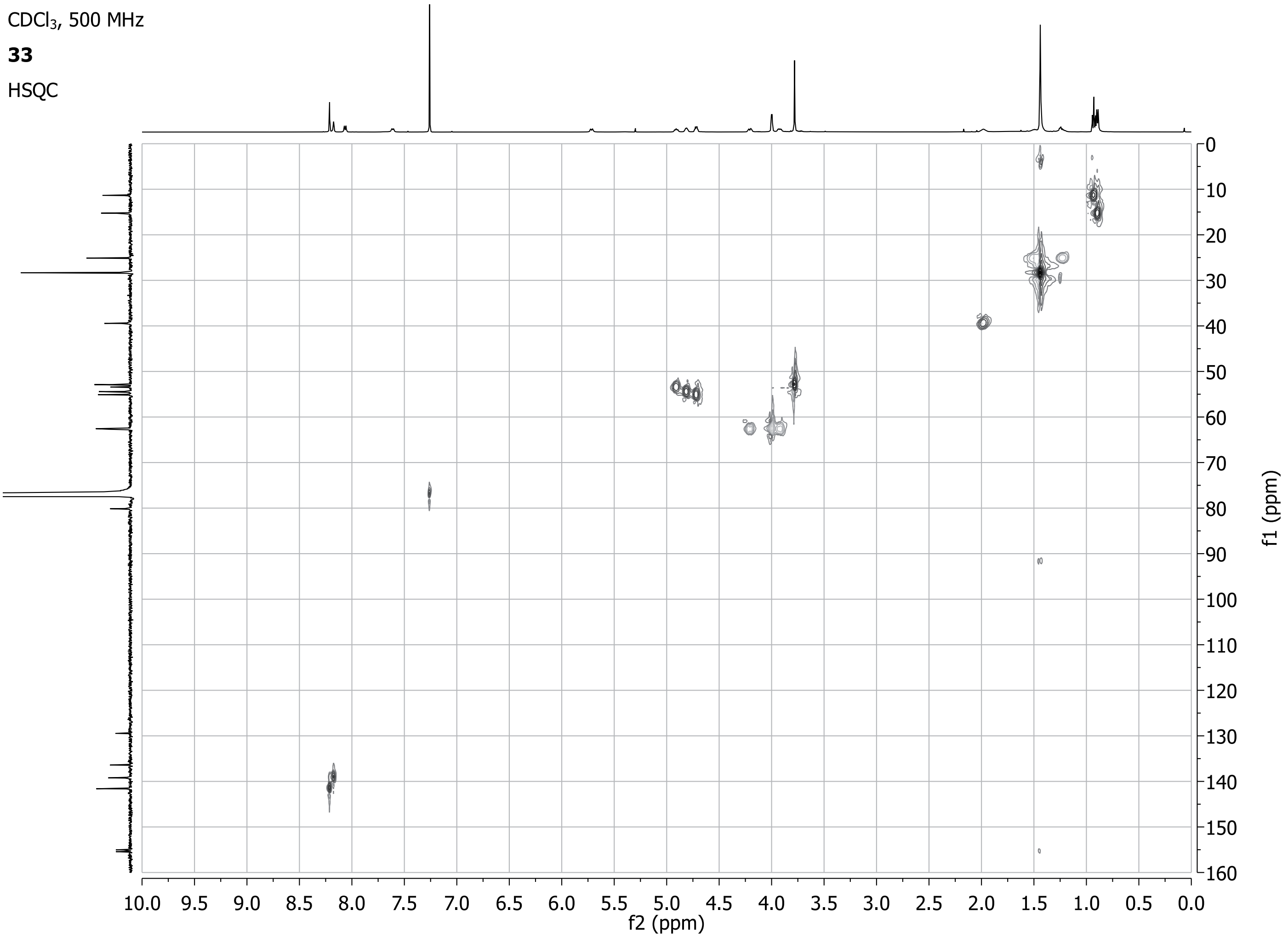

CDCl$_3$, 500 MHz

33

HMBC

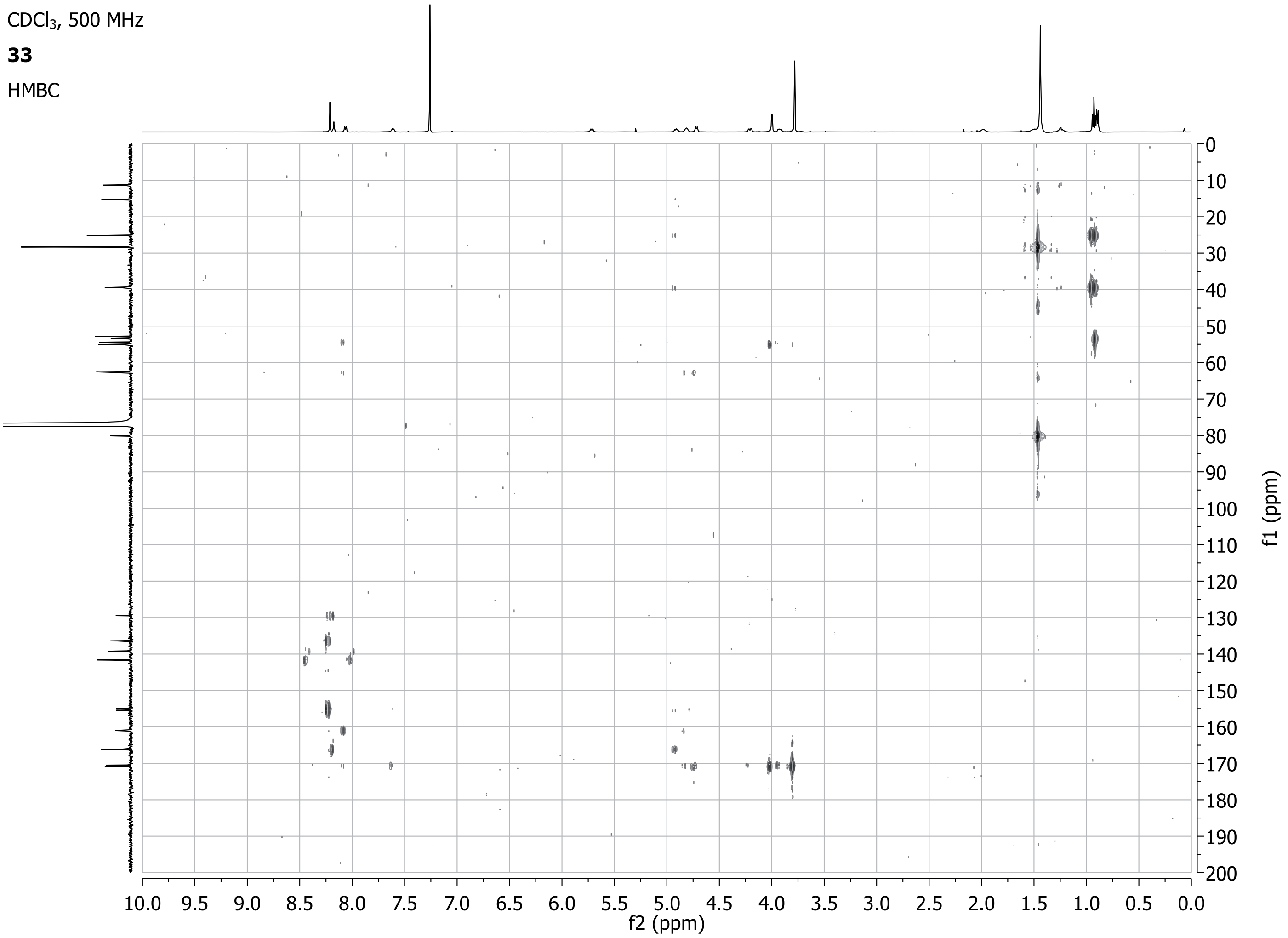

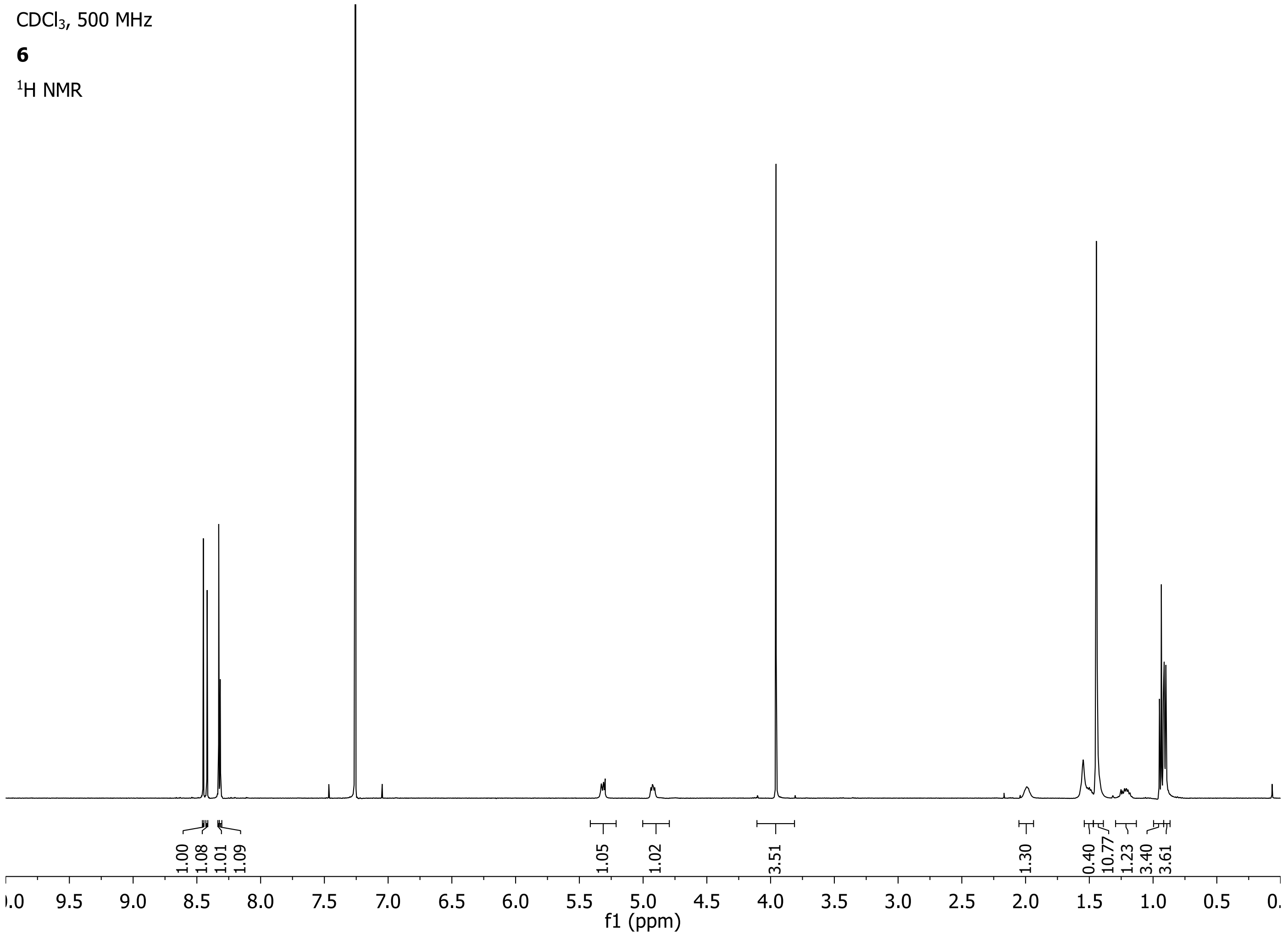
CDCl3, 500 MHz
6
1H NMR
1.00
1.08
1.01
1.09
1.05
1.02
3.51
1.30
0.40
10.77
1.23
3.40
3.61
f1 (ppm)

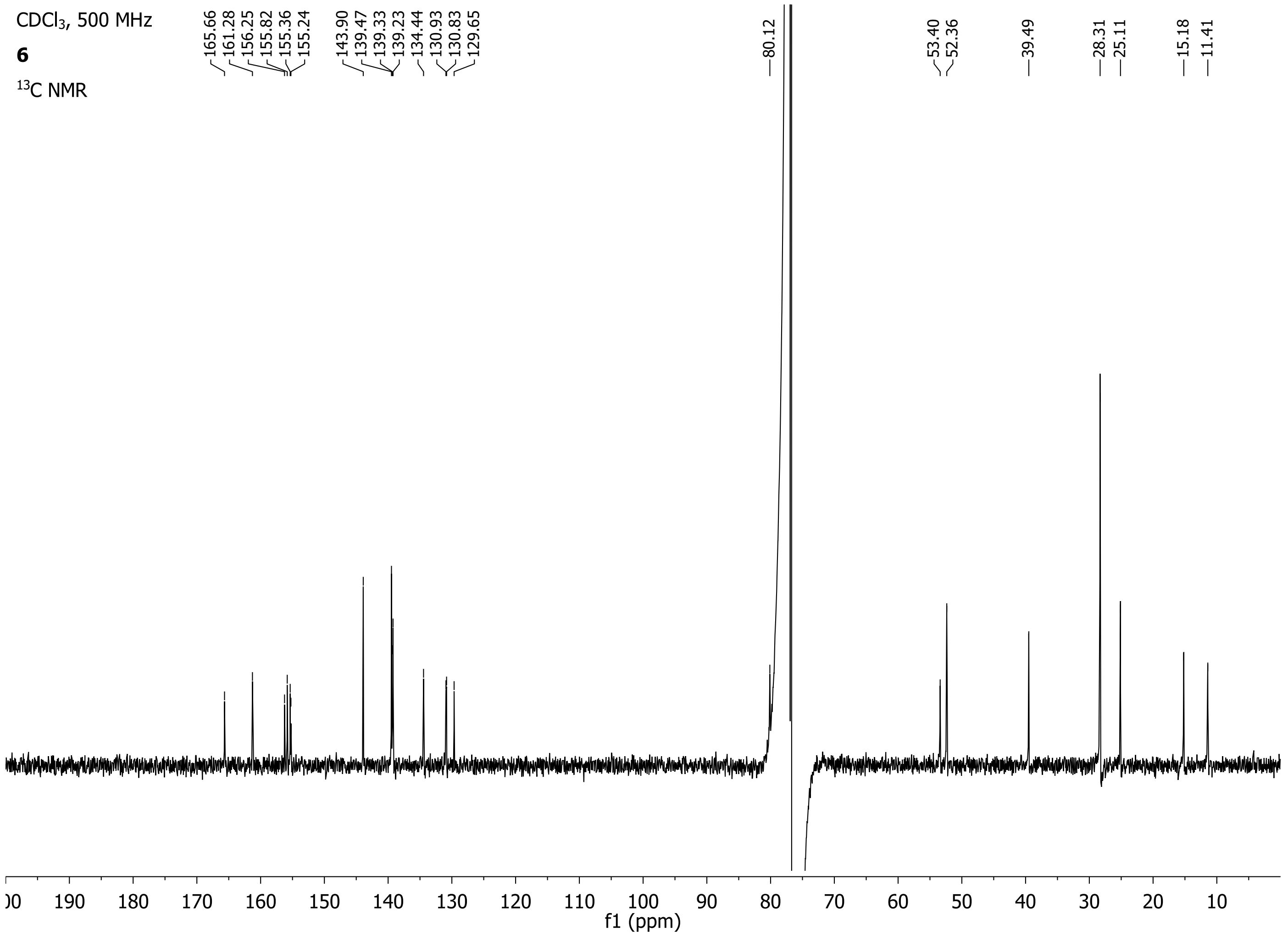
CDCl3, 500 MHz
6
13C NMR
165.66
161.28
156.25
155.82
155.36
155.24
143.90
139.47
139.33
139.23
134.44
130.93
130.83
129.65
80.12
53.40
52.36
39.49
28.31
25.11
15.18
11.41
f1 (ppm)

CDCl3, 500 MHz

6

^{13}C NMR

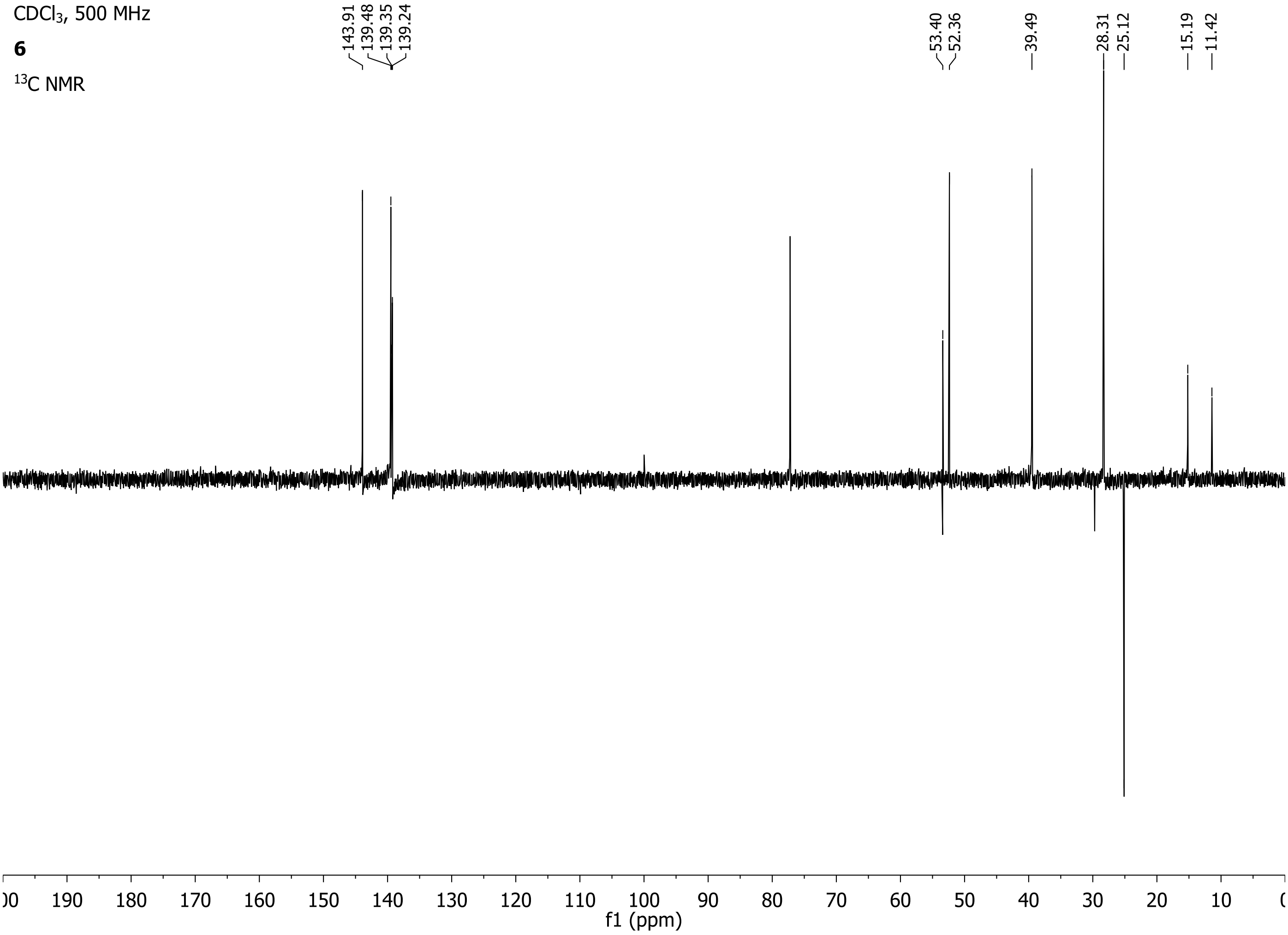

CDCl3, 500 MHz

6

COSY

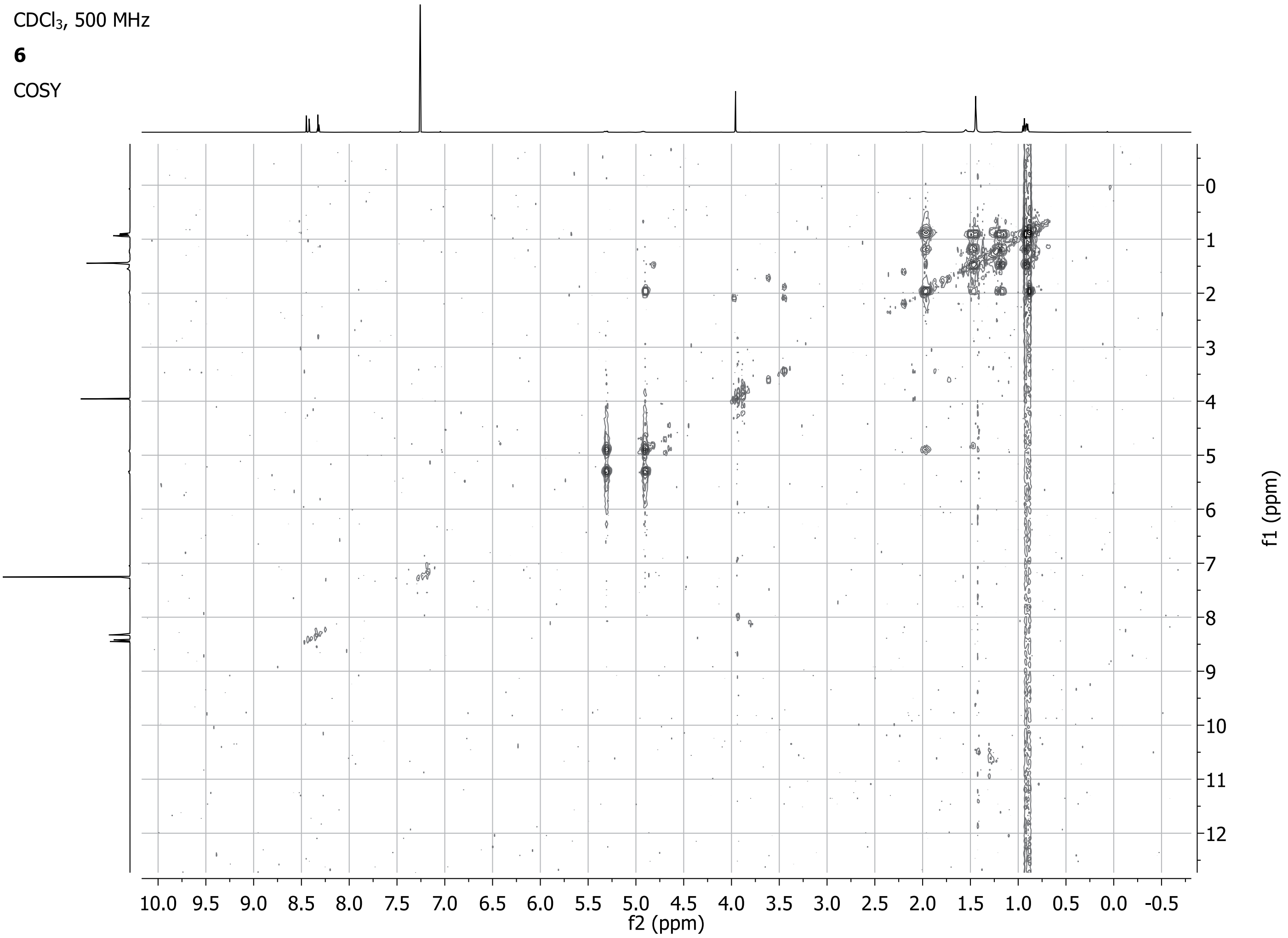

CDCl$_3$, 500 MHz

6

HSQC

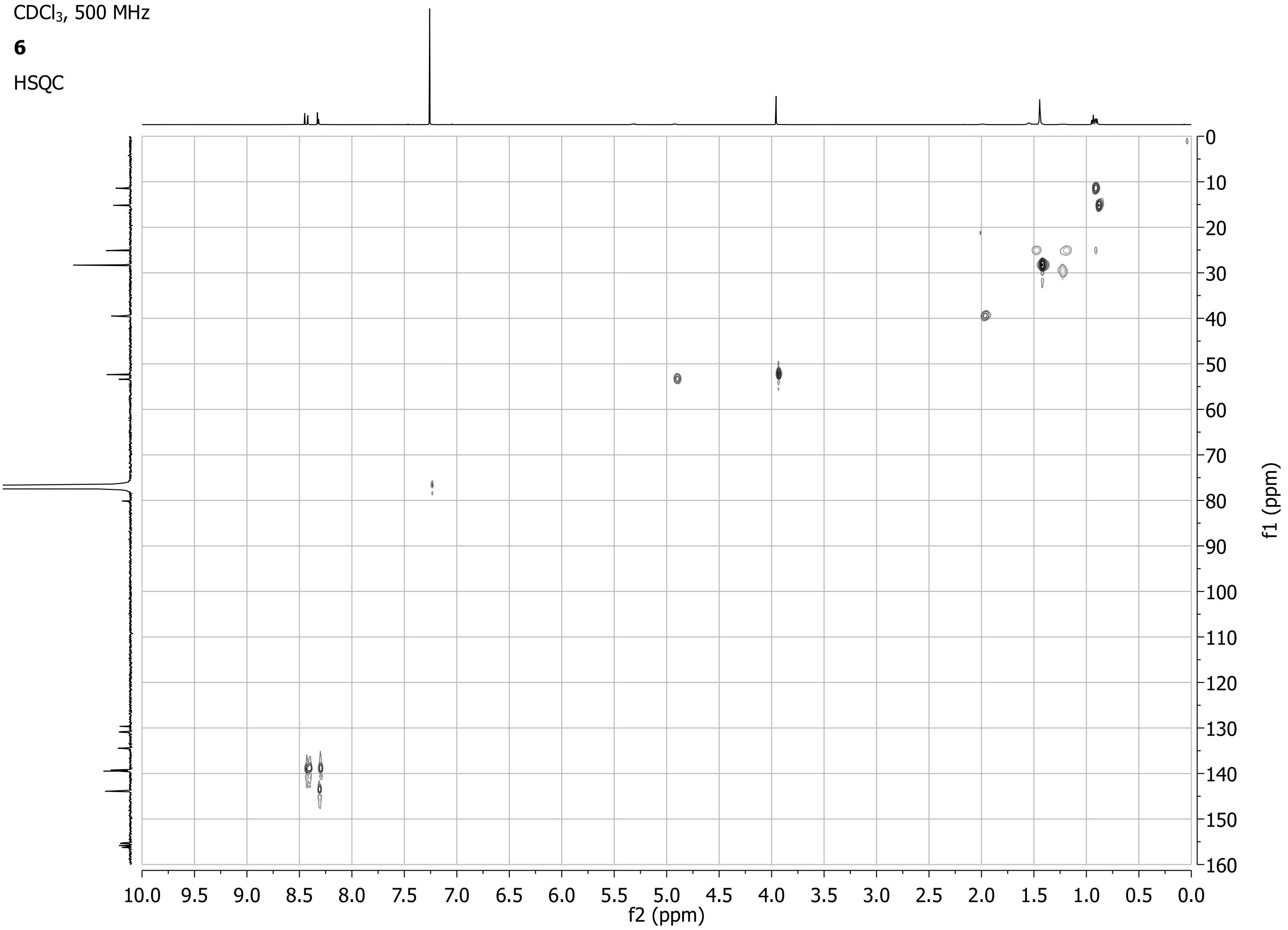

CDCl$_3$, 500 MHz

6

HMBC

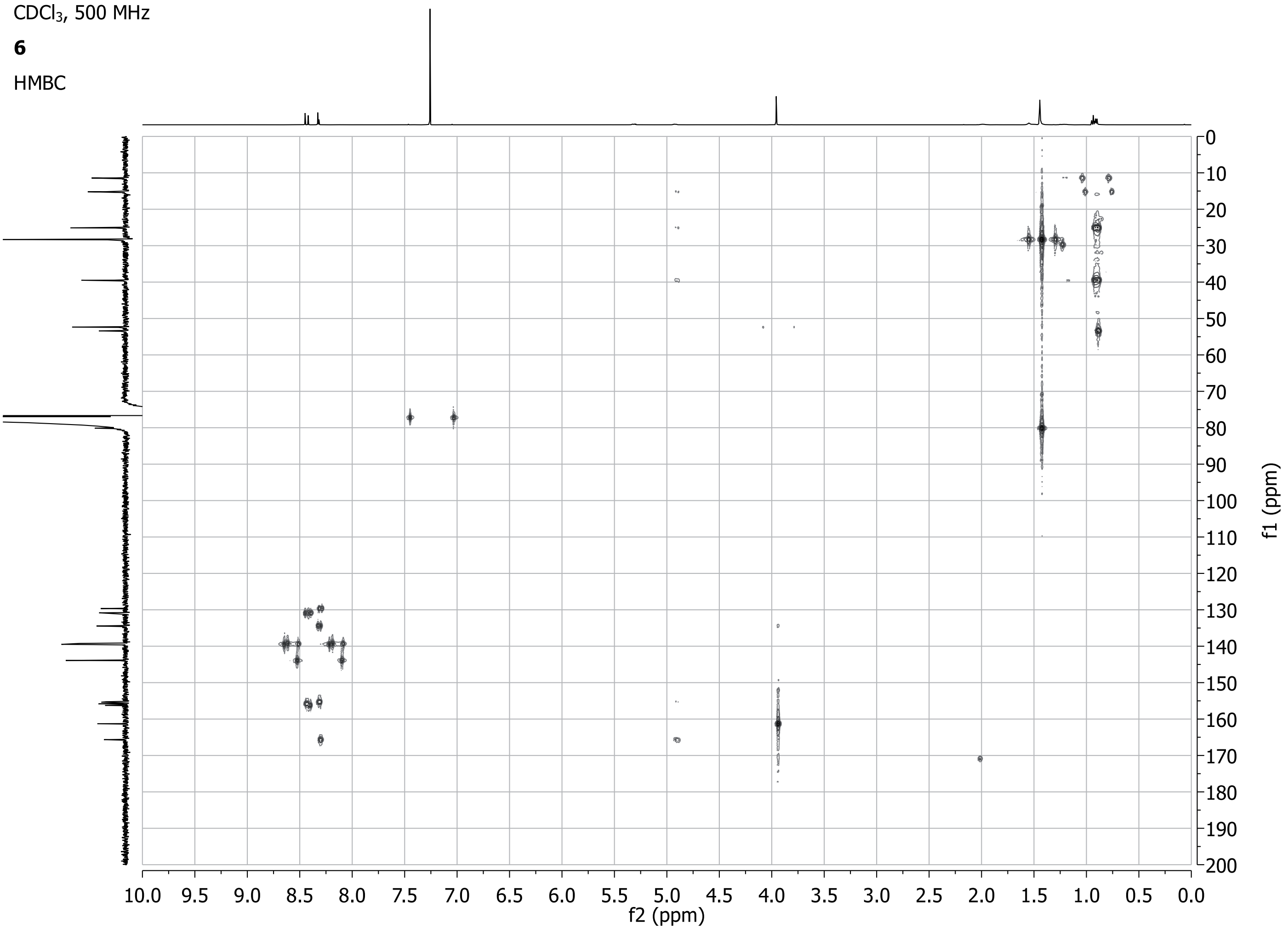

CDCl$_3$, 500 MHz

36

^{1}H NMR

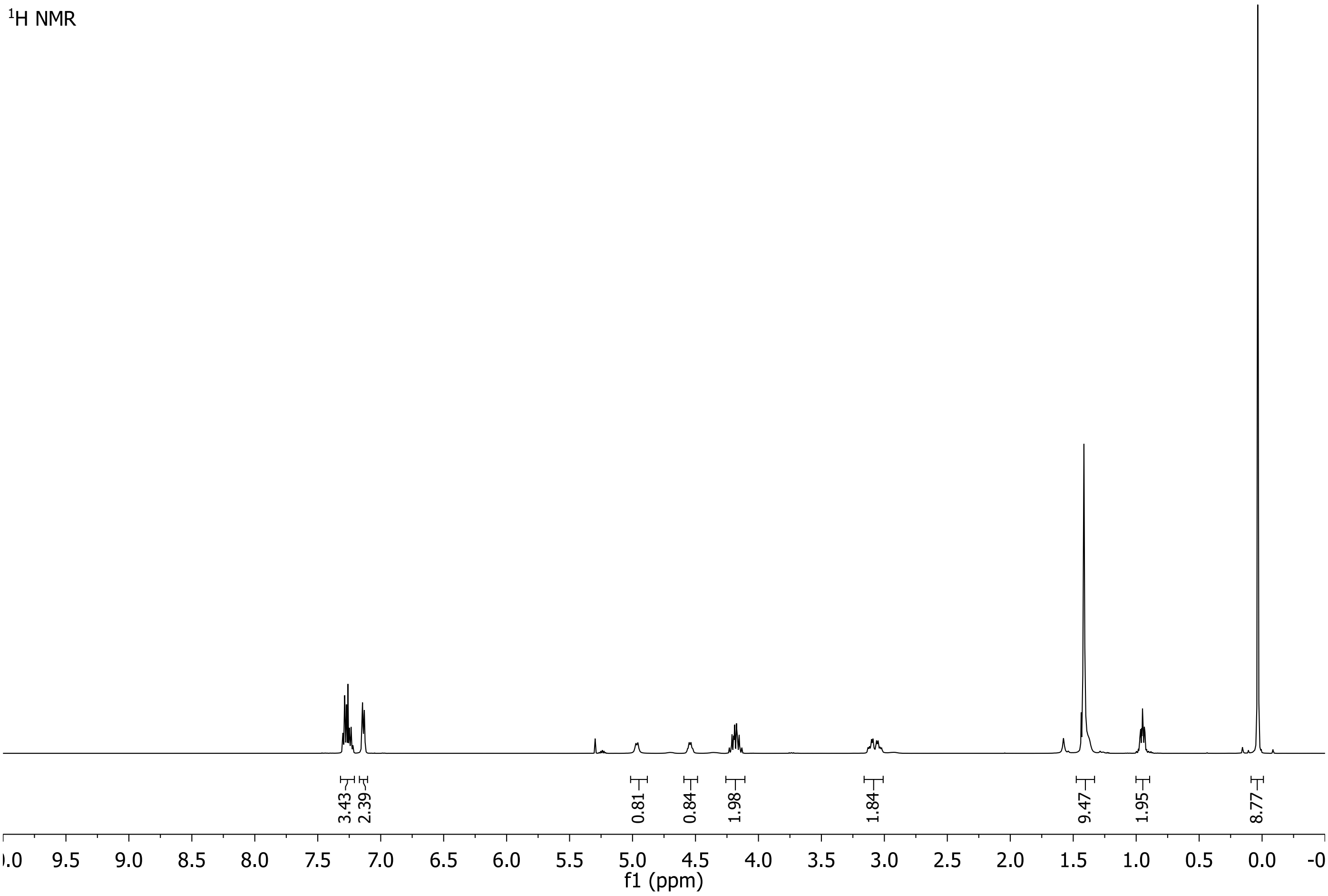

CDCl$_3$, 500 MHz

36

^{13}C NMR

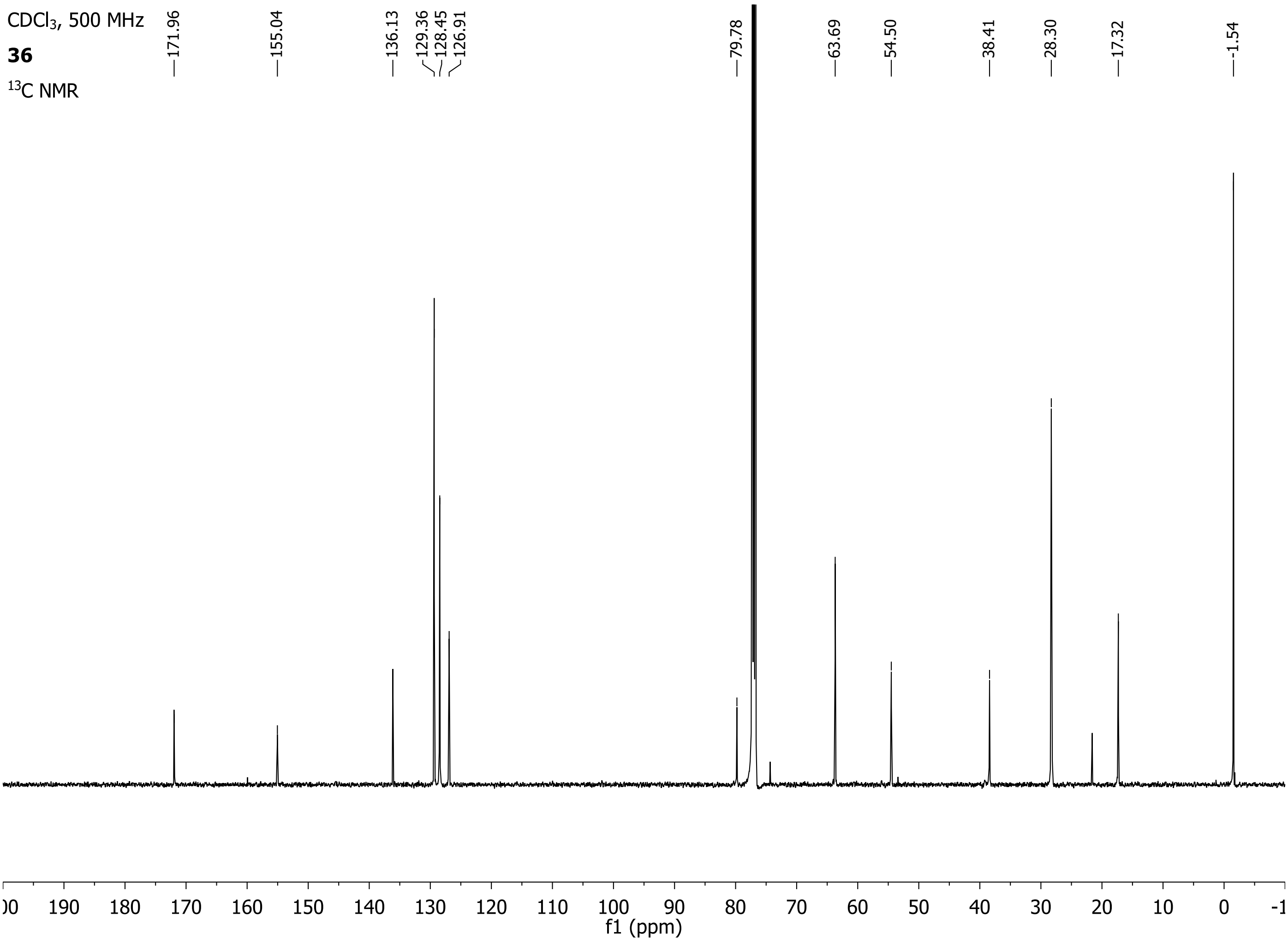

CDCl$_3$, 500 MHz

36

^{13}C NMR

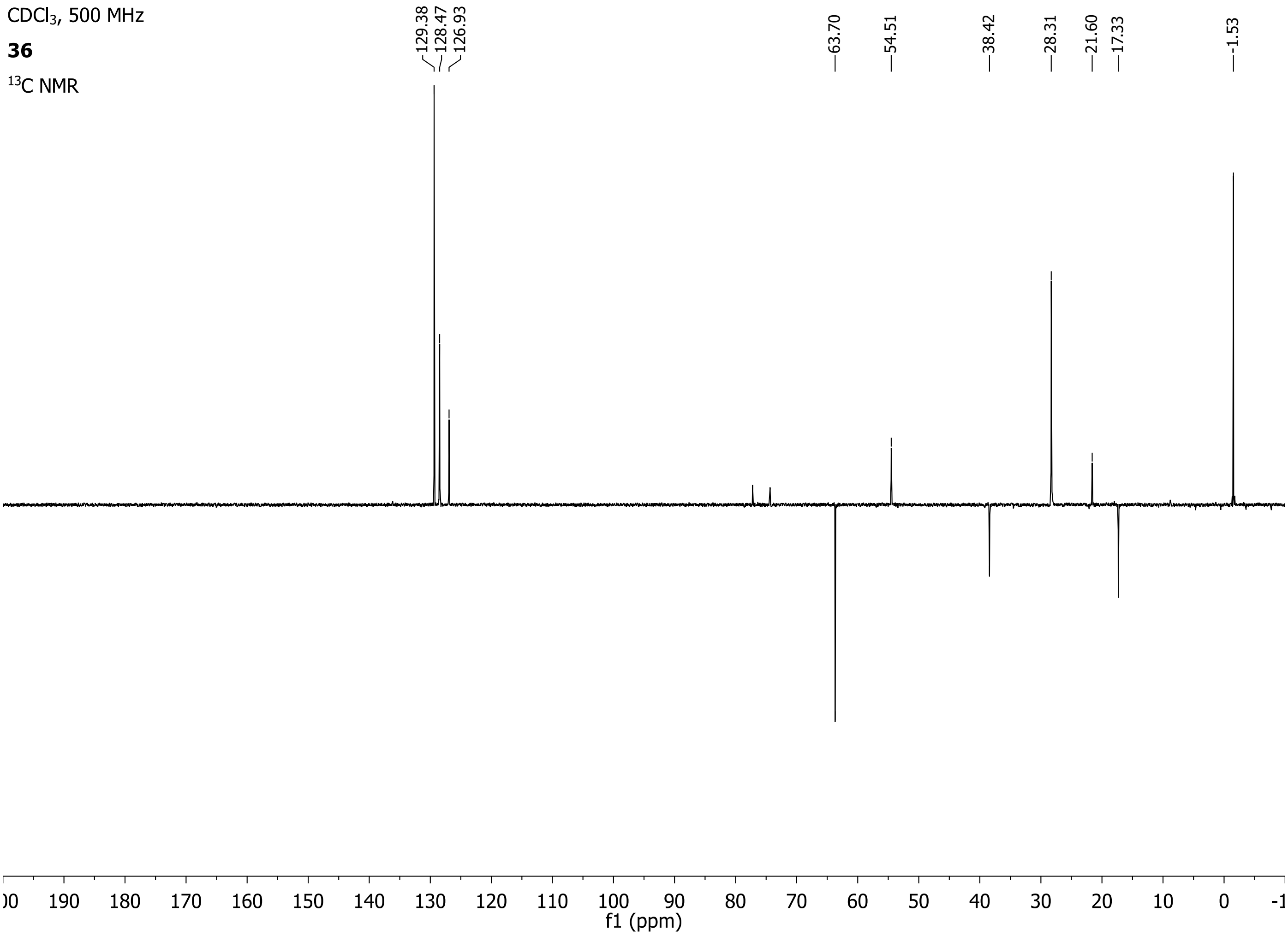

CDCl$_3$, 500 MHz

36

COSY

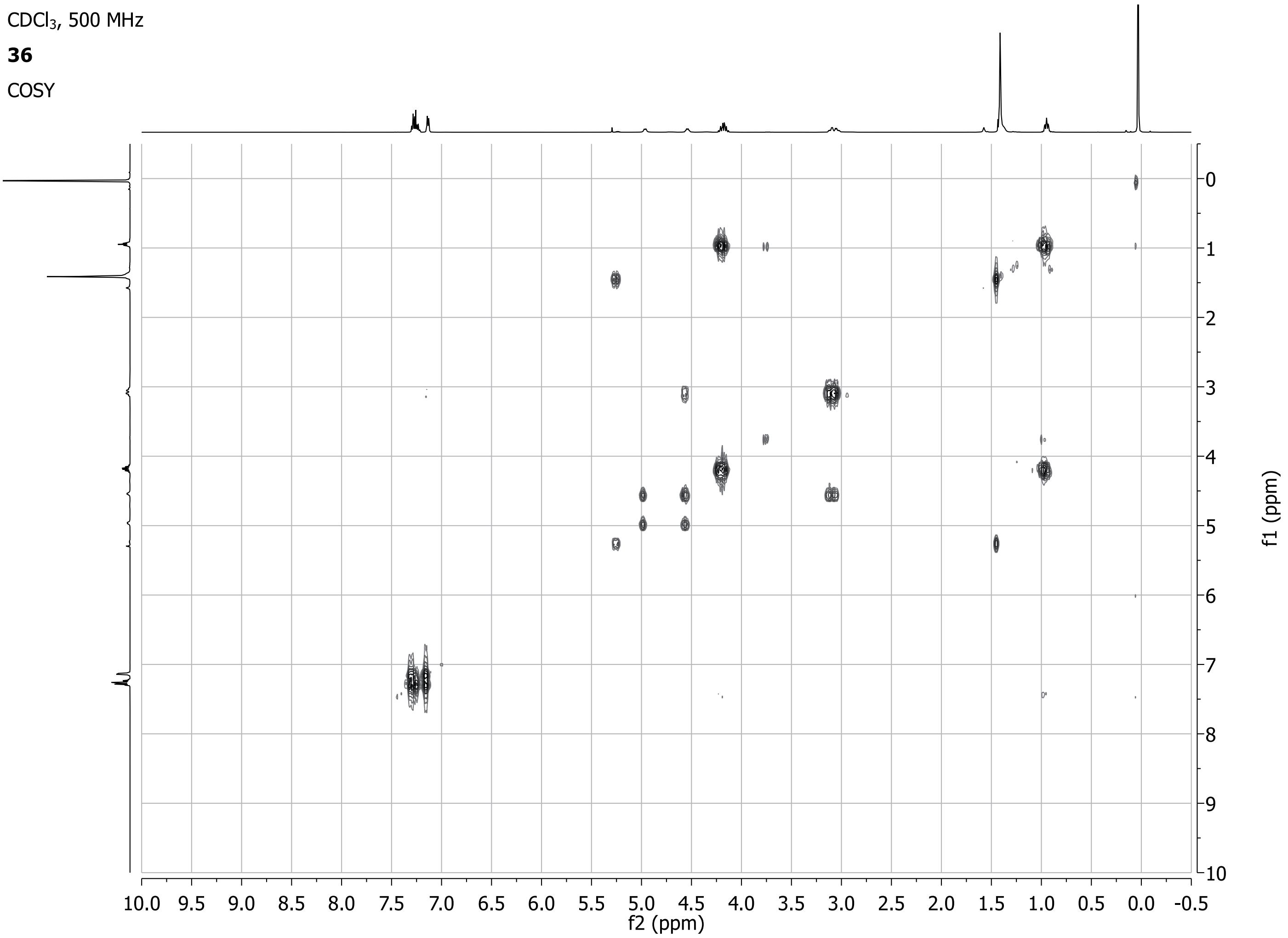

CDCl$_3$, 500 MHz

36

HSQC

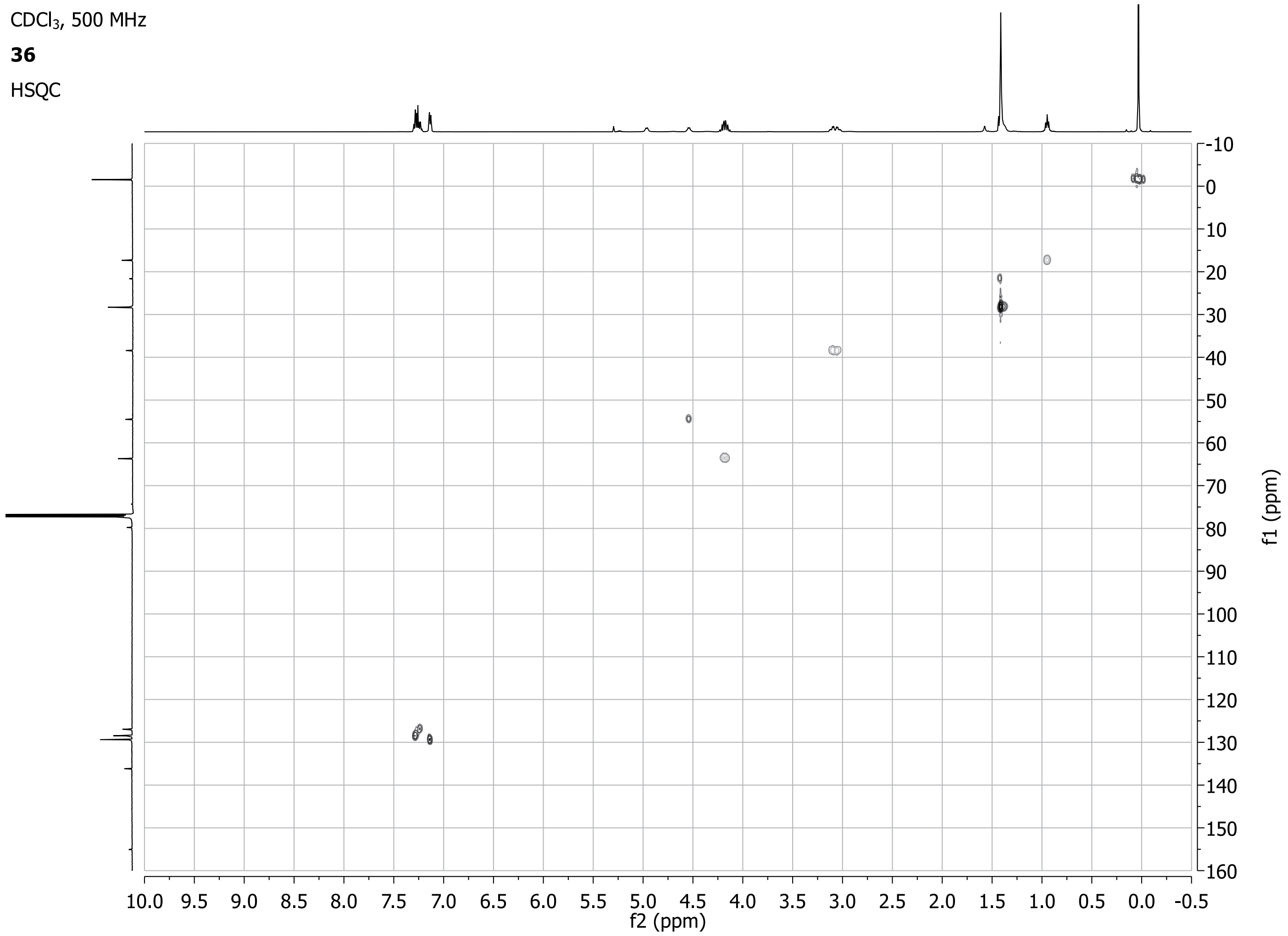

CDCl$_3$, 500 MHz

36

HMBC

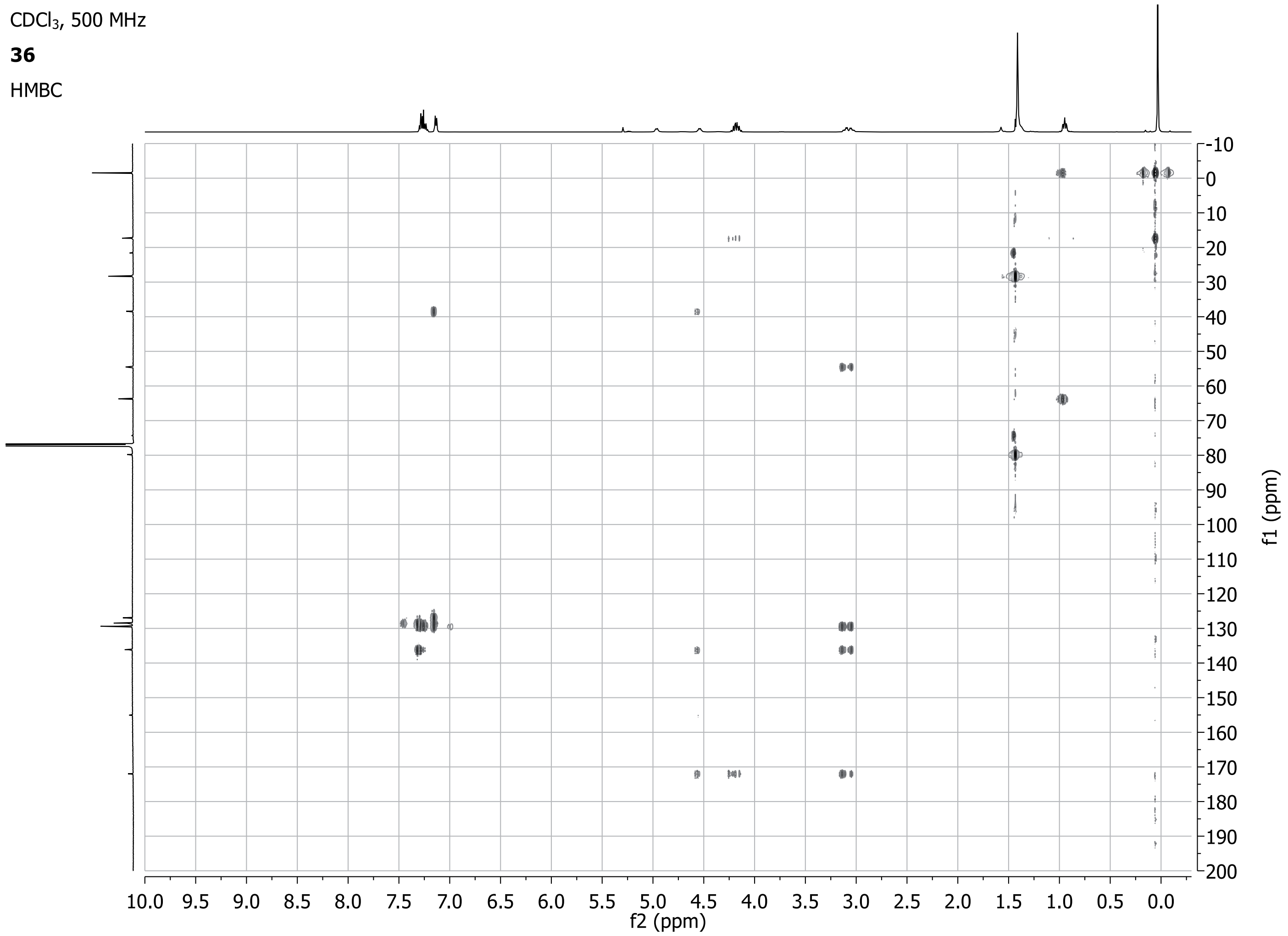

CDCl3, 500 MHz

7

^{1}H NMR

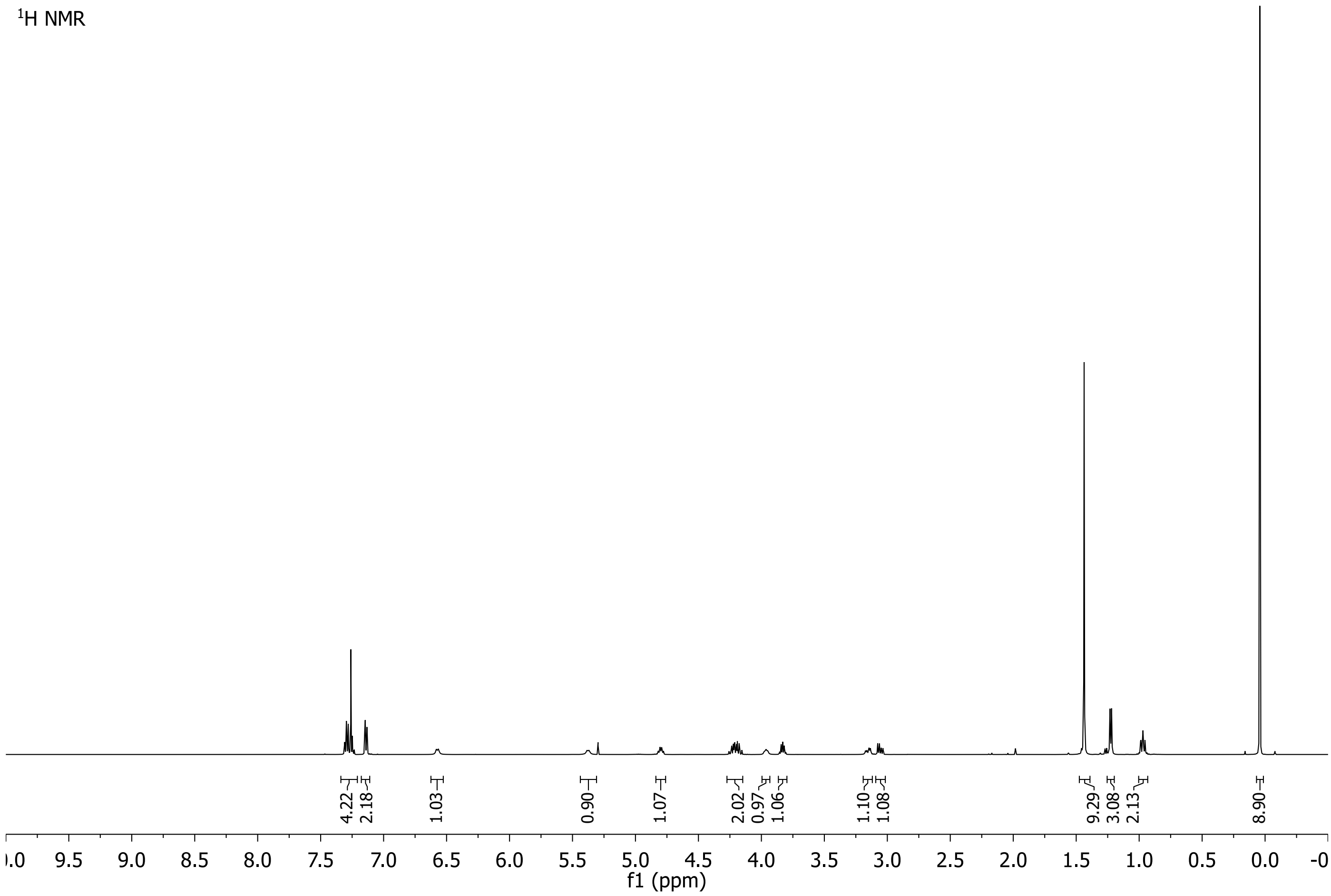

CDCl3, 500 MHz

7

^{13}C NMR

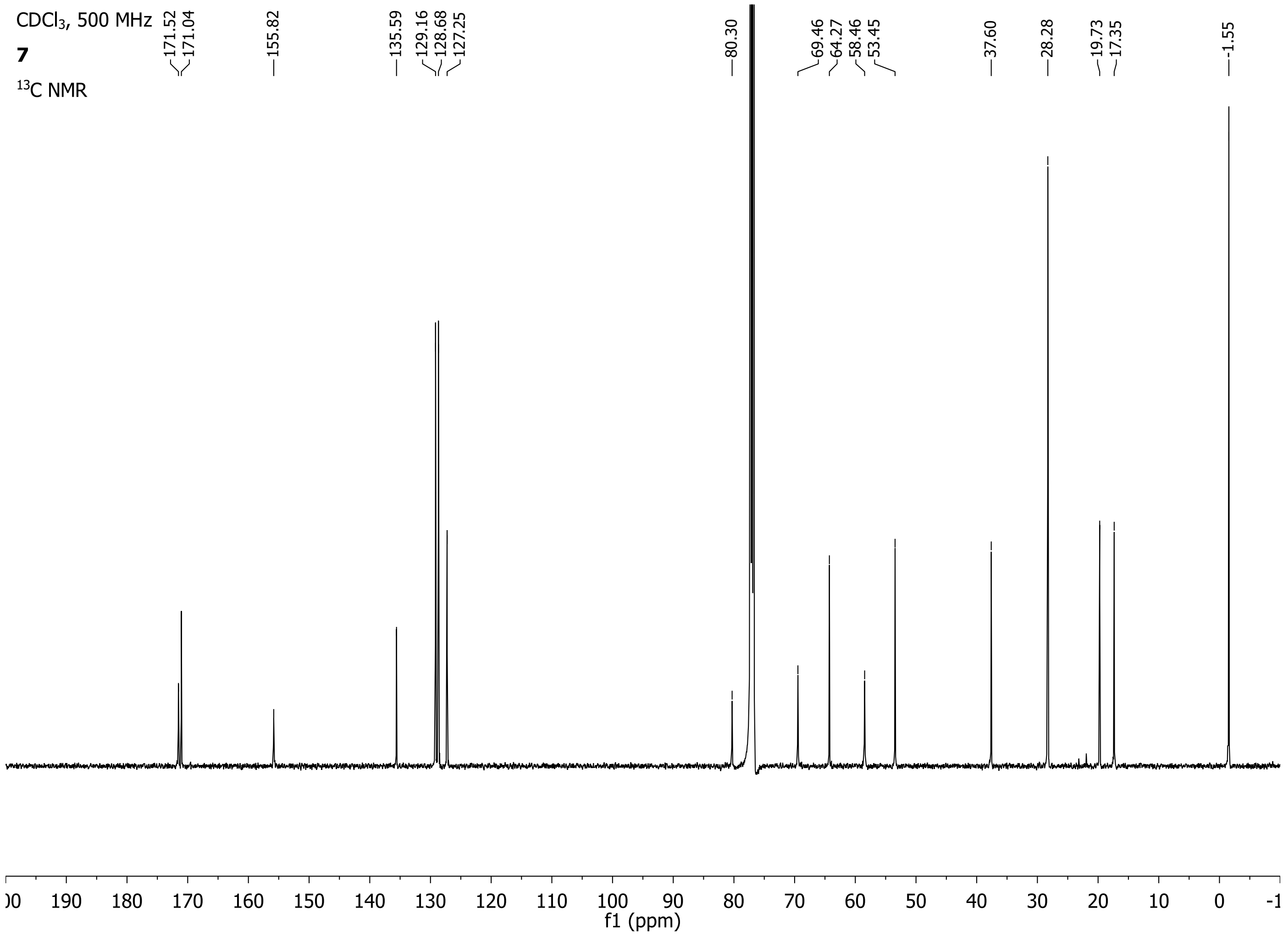

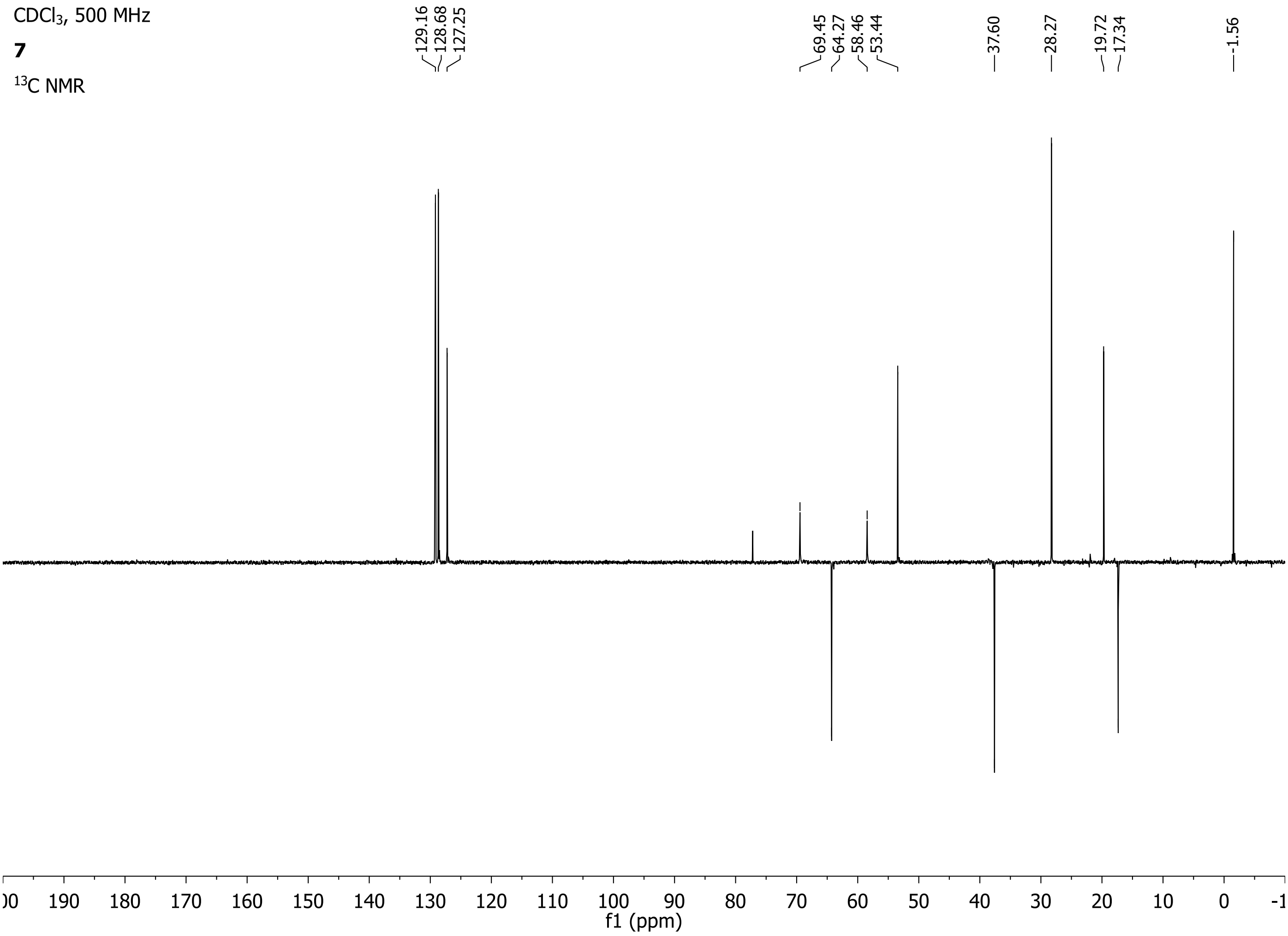
CDCl3, 500 MHz
7
13C NMR
129.16
128.68
127.25
69.45
64.27
58.46
53.44
37.60
28.27
19.72
17.34
-1.56
f1 (ppm)

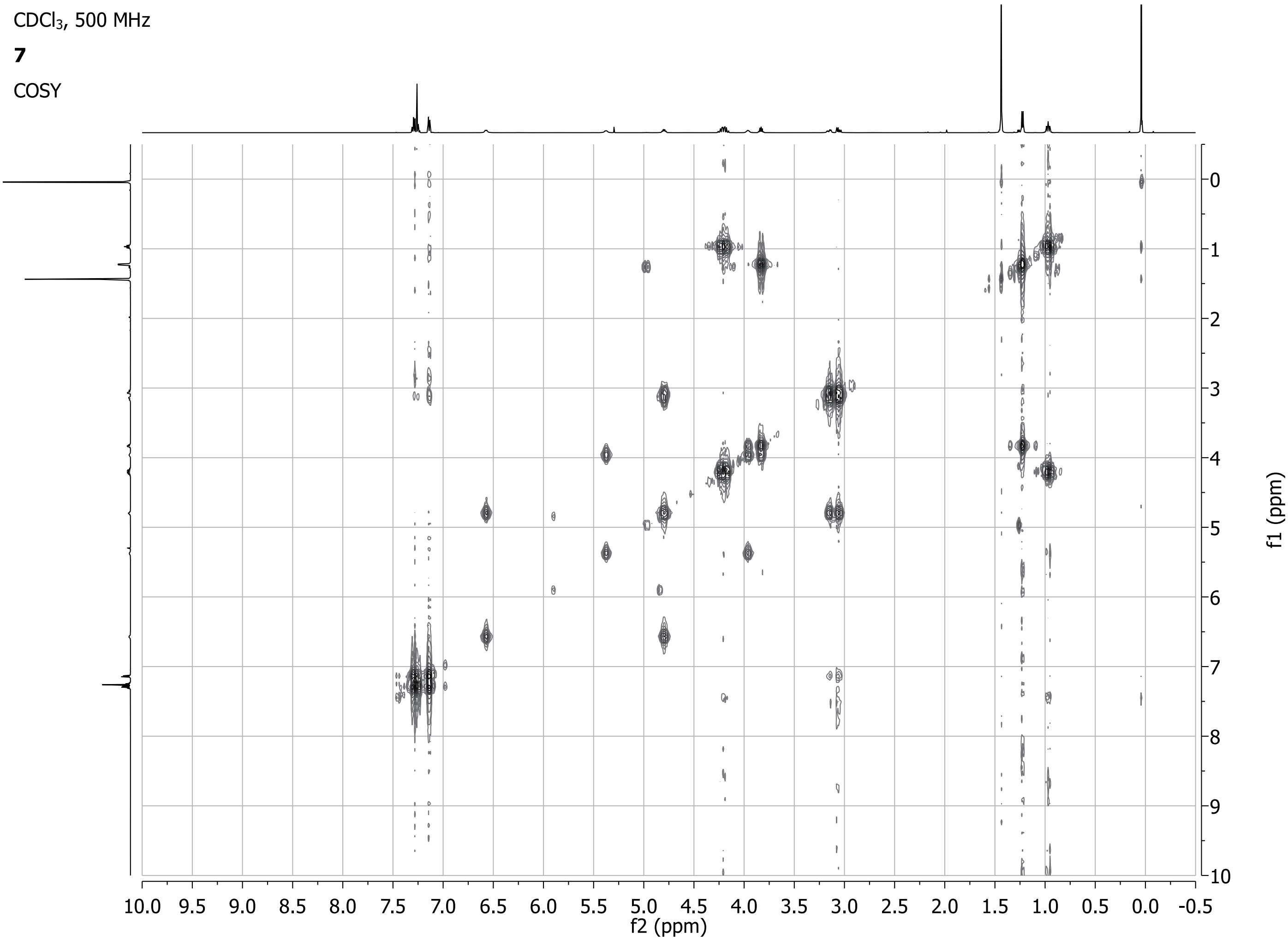
CDCl3, 500 MHz
7
COSY
f2 (ppm)
f1 (ppm)

CDCl$_3$, 500 MHz

7

HSQC

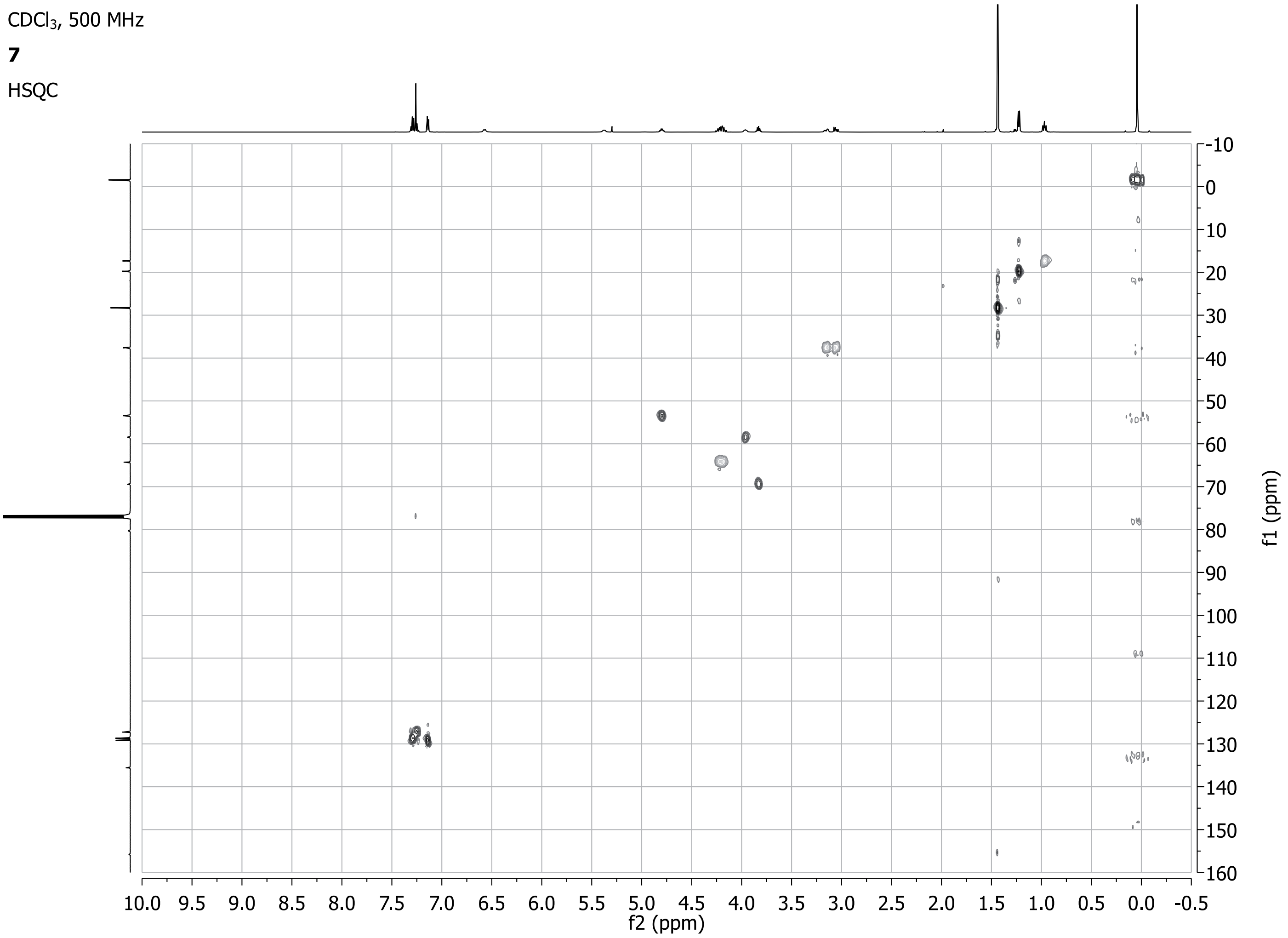

CDCl$_3$, 500 MHz

7

HMQC

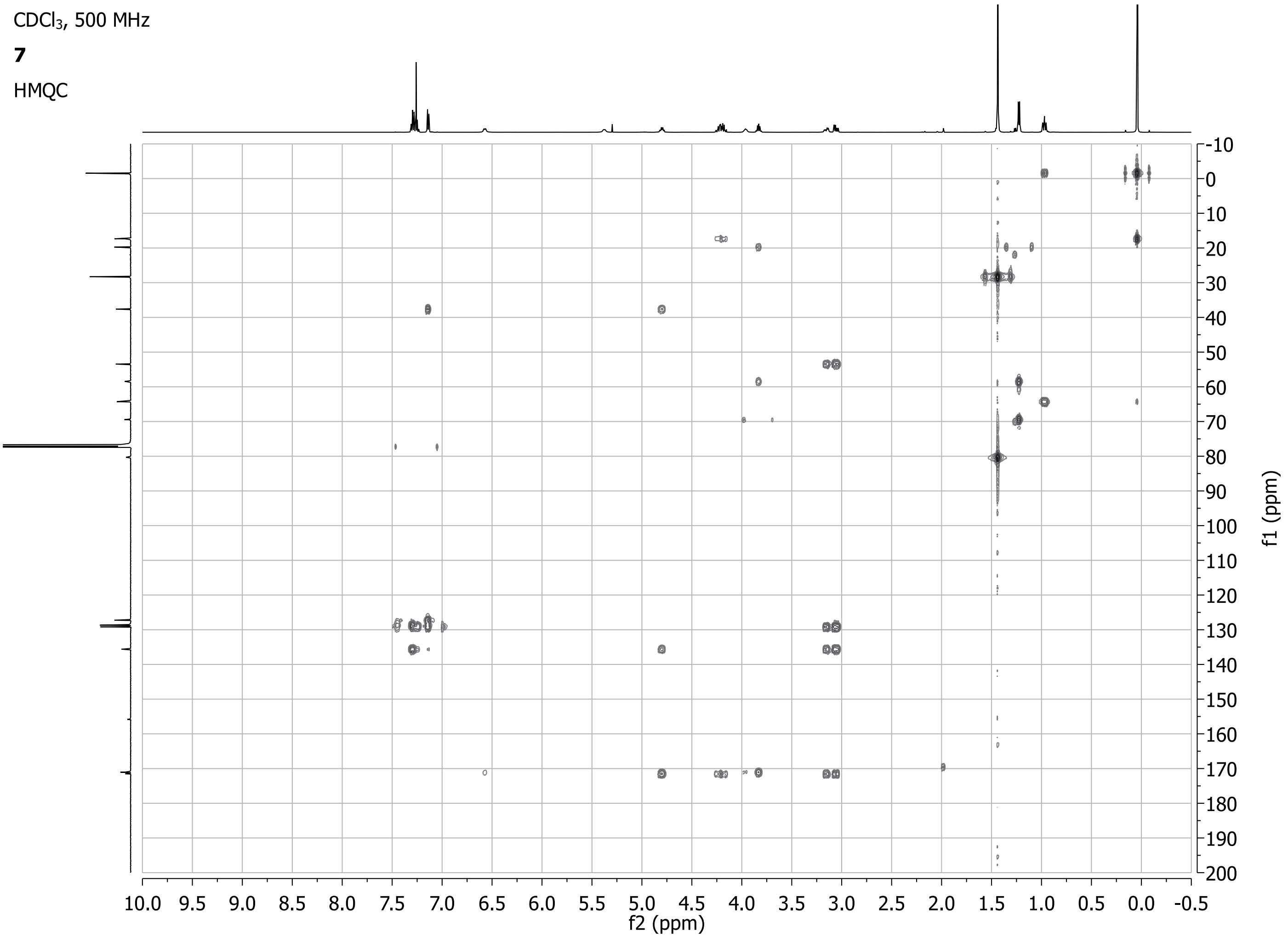

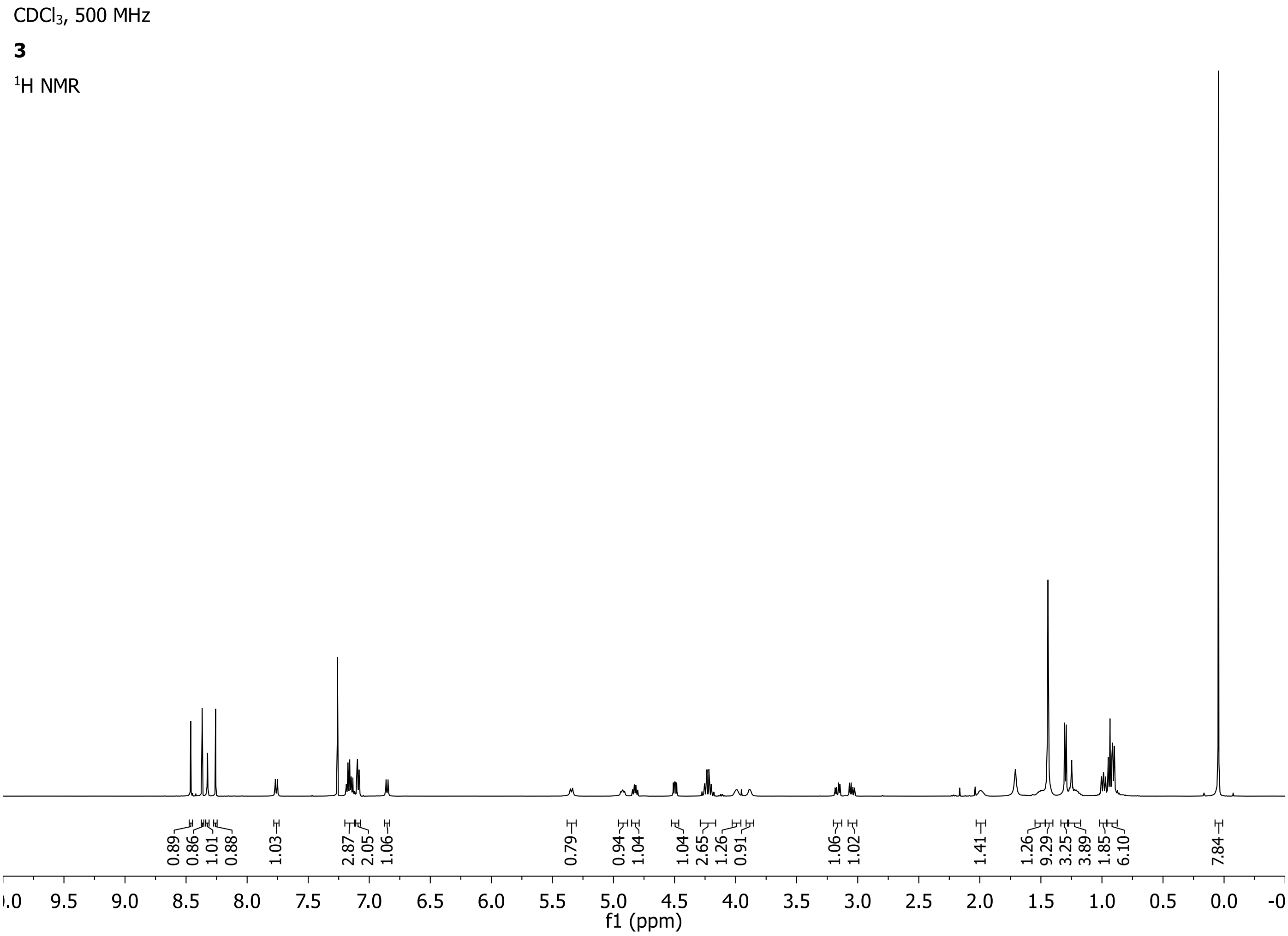
CDCl$_3$, 500 MHz
3
^{1}H NMR
0.89 0.86 1.01 0.88 1.03 2.87 2.05 1.06 0.79 0.94 1.04 1.04 2.65 1.26 0.91 1.06 1.02 1.41 1.26 9.29 3.25 3.89 1.85 6.10 7.84
f1 (ppm)

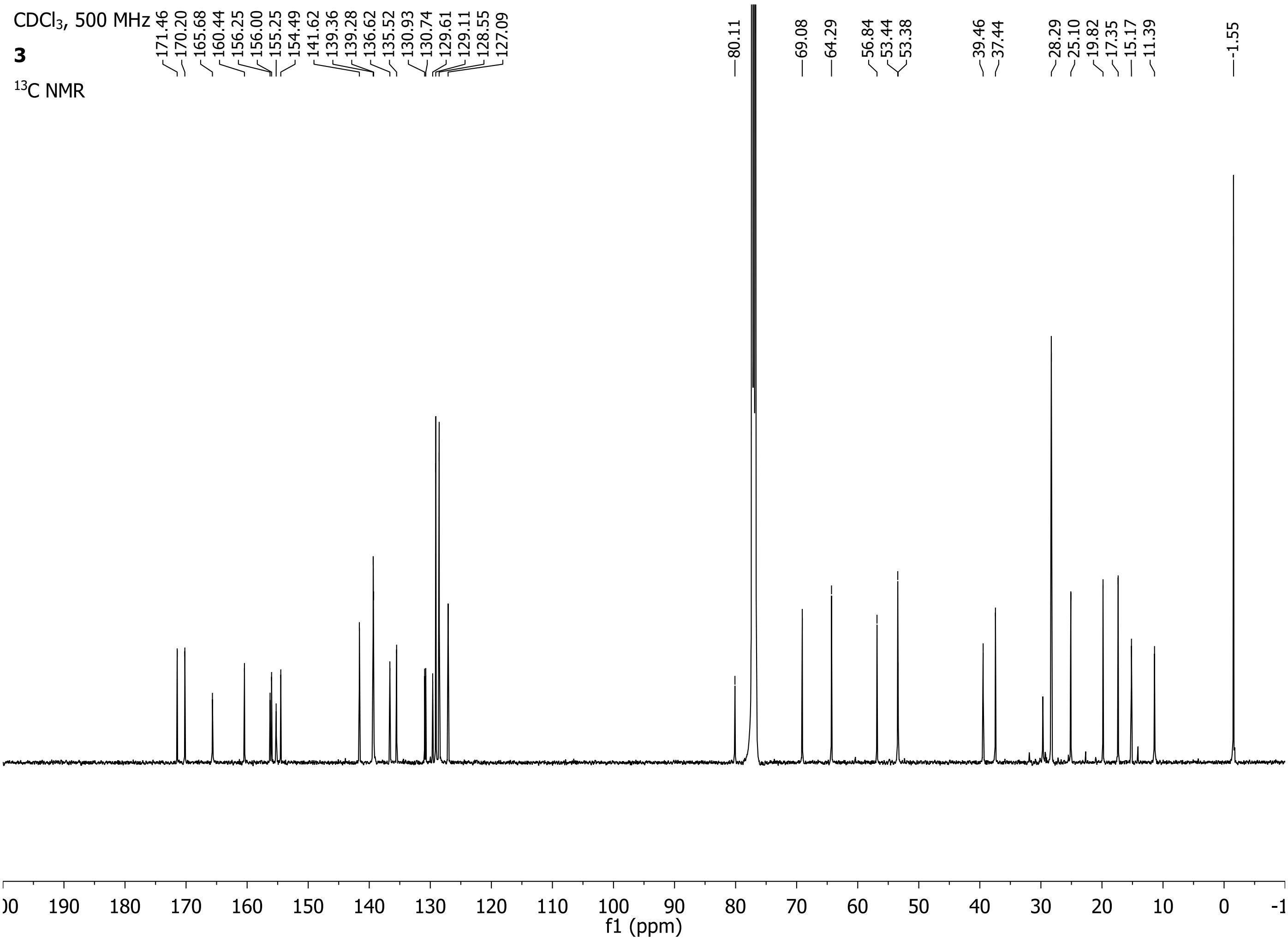
CDCl$_3$, 500 MHz
3
^{13}C NMR
171.46 170.20 165.68 160.44 156.25 156.00 155.25 154.49 141.62 139.36 139.28 136.62 135.52 130.93 130.74 129.61 129.11 128.55 127.09
80.11 69.08 64.29 56.84 53.44 53.38 39.46 37.44 28.29 25.10 19.82 17.35 15.17 11.39 -1.55
f1 (ppm)

CDCl3, 500 MHz

3

^{13}C NMR

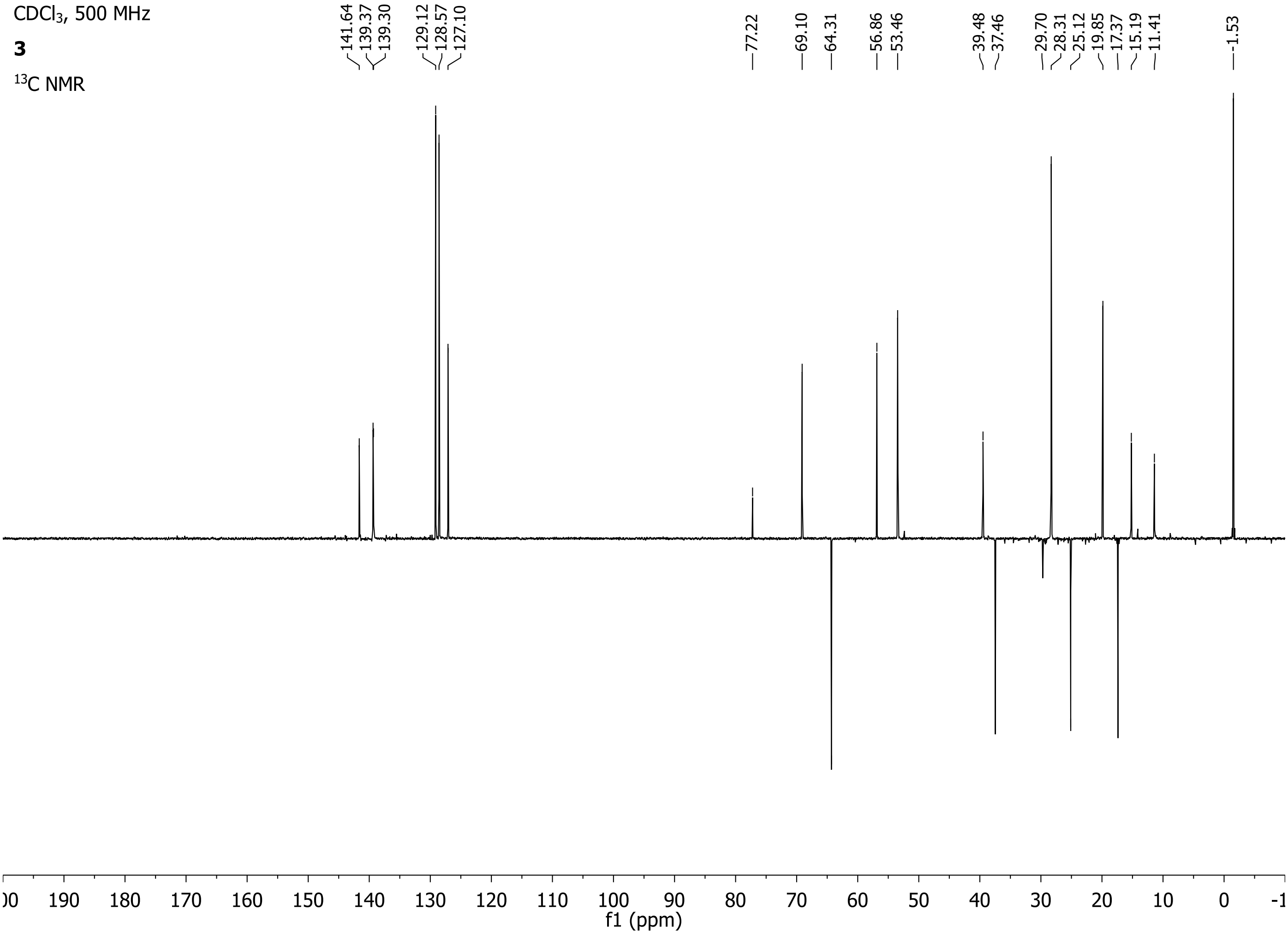

CDCl3, 500 MHz

3

COSY

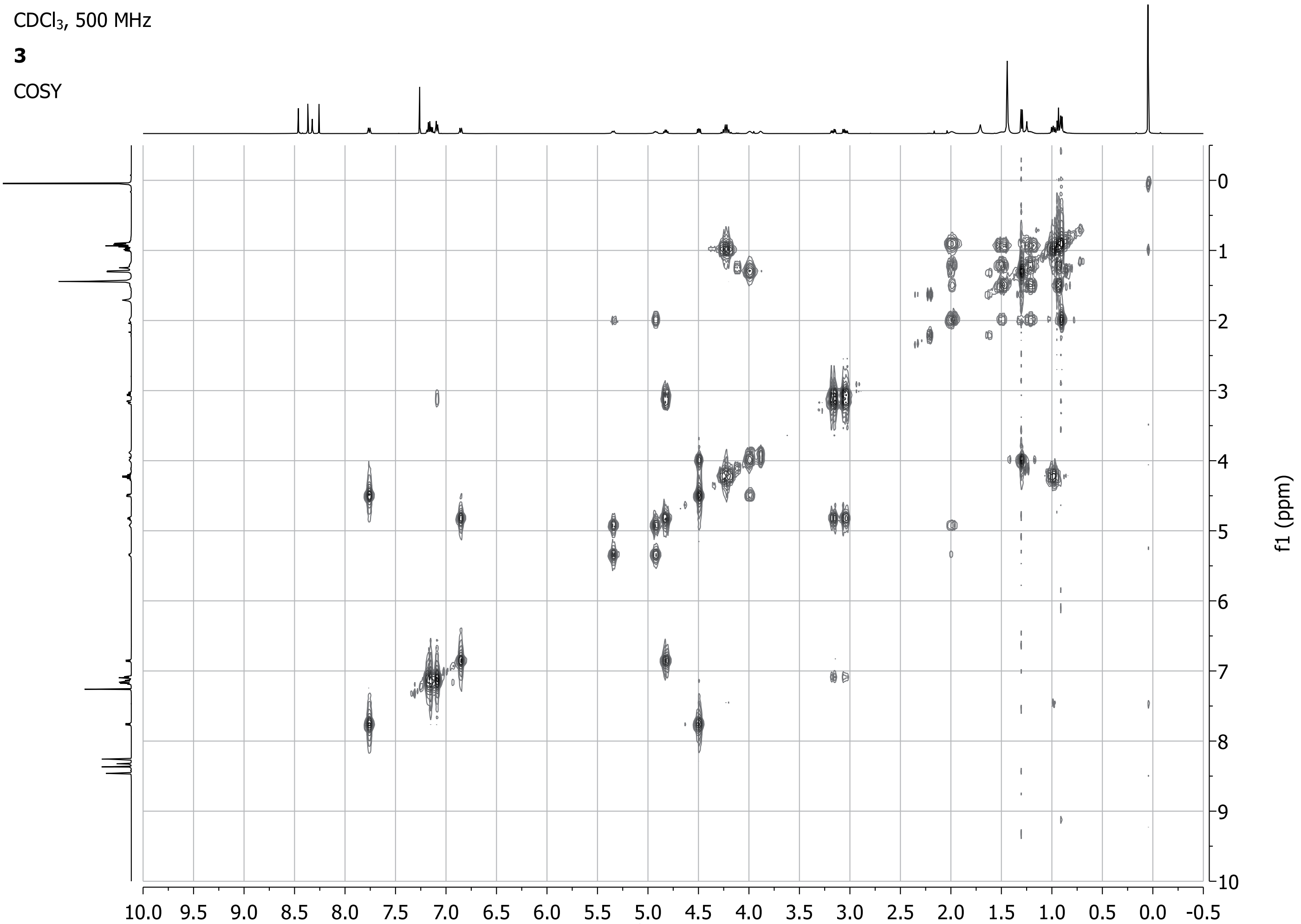

CDCl$_3$, 500 MHz

32

HSQC

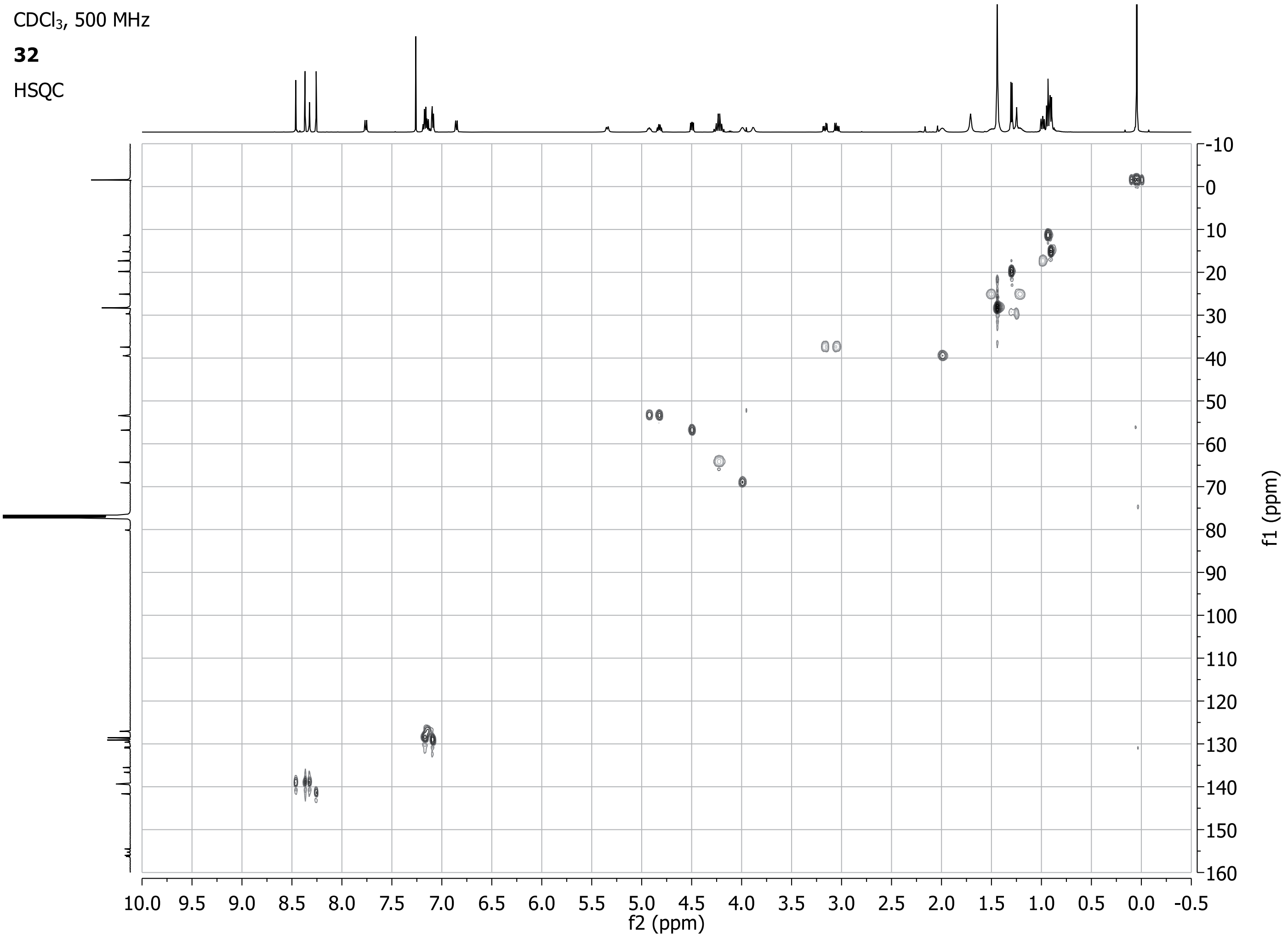

CDCl$_3$, 500 MHz

3

HMBC

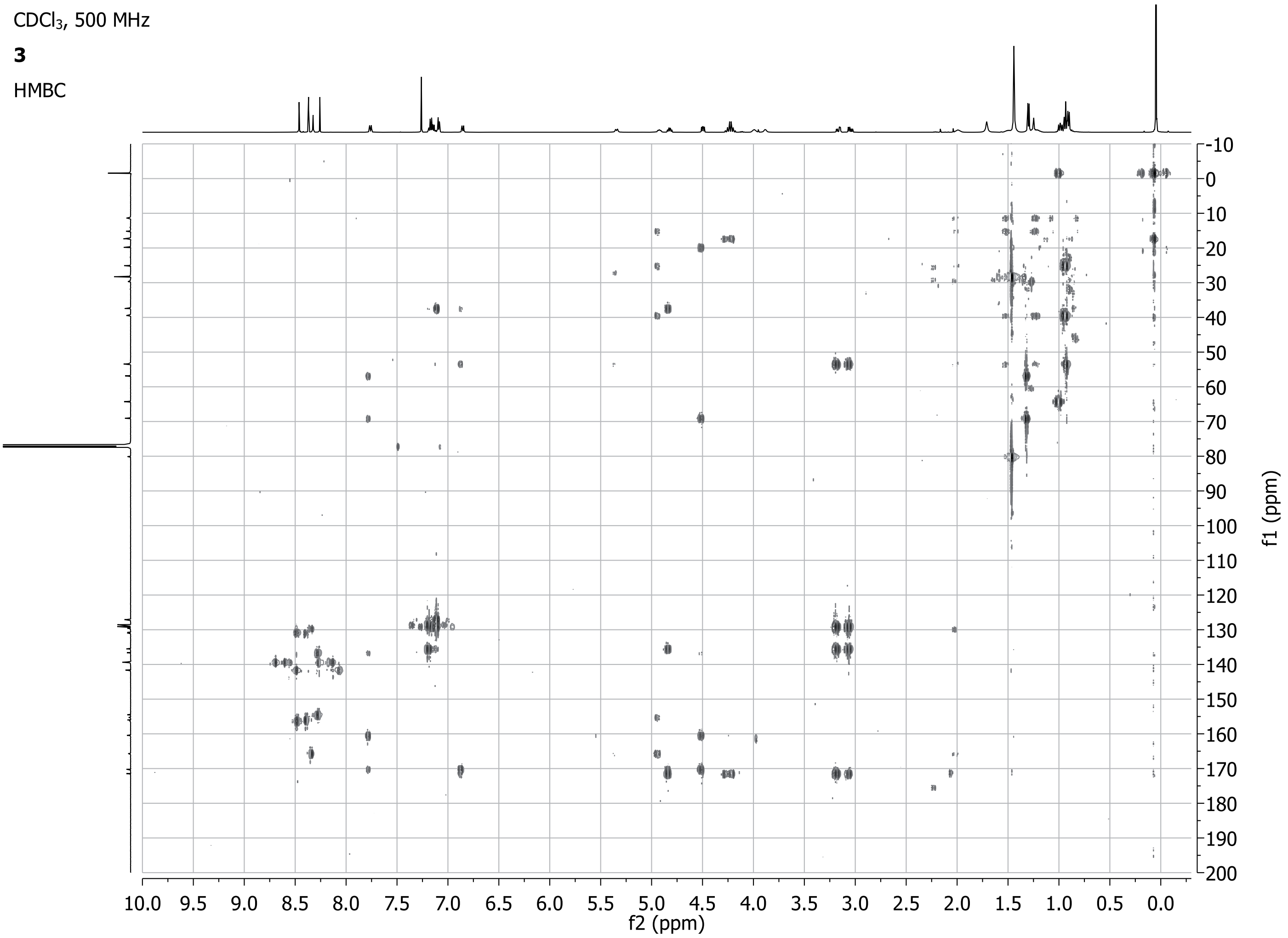

CDCl$_3$, 400 MHz

37a

^{1}H NMR

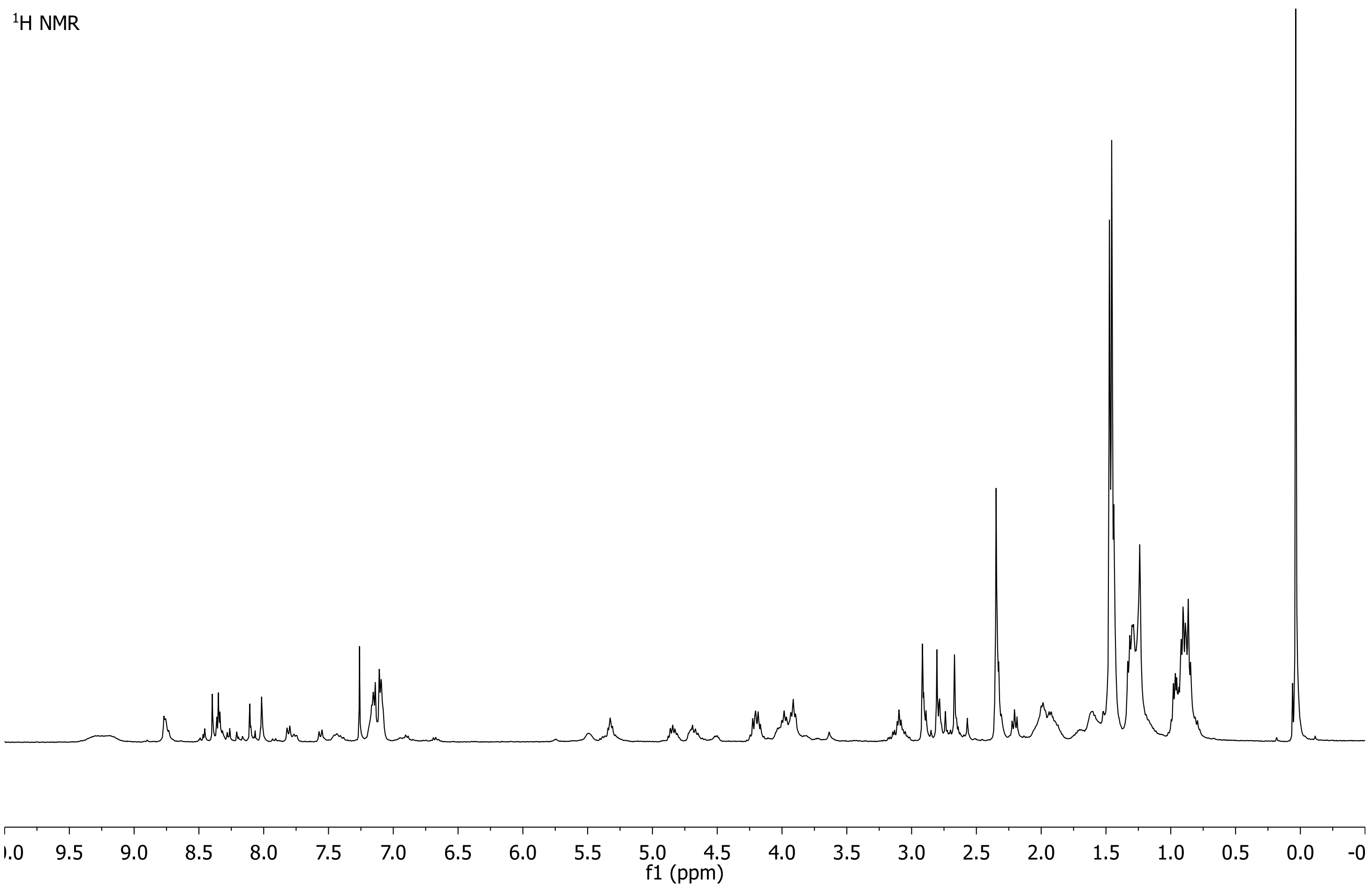

CDCl$_3$, 500 MHz

38a

^{1}H NMR

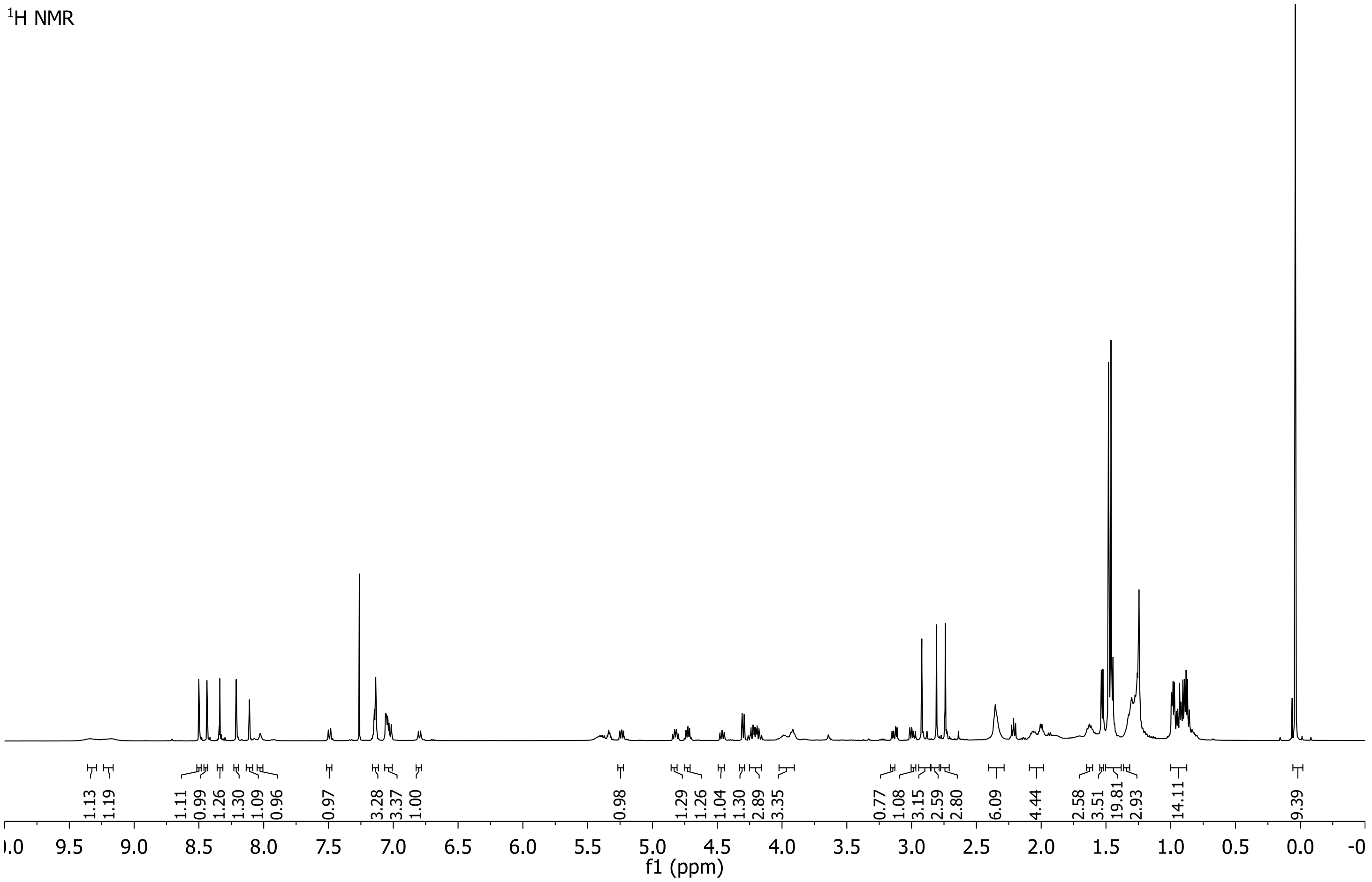

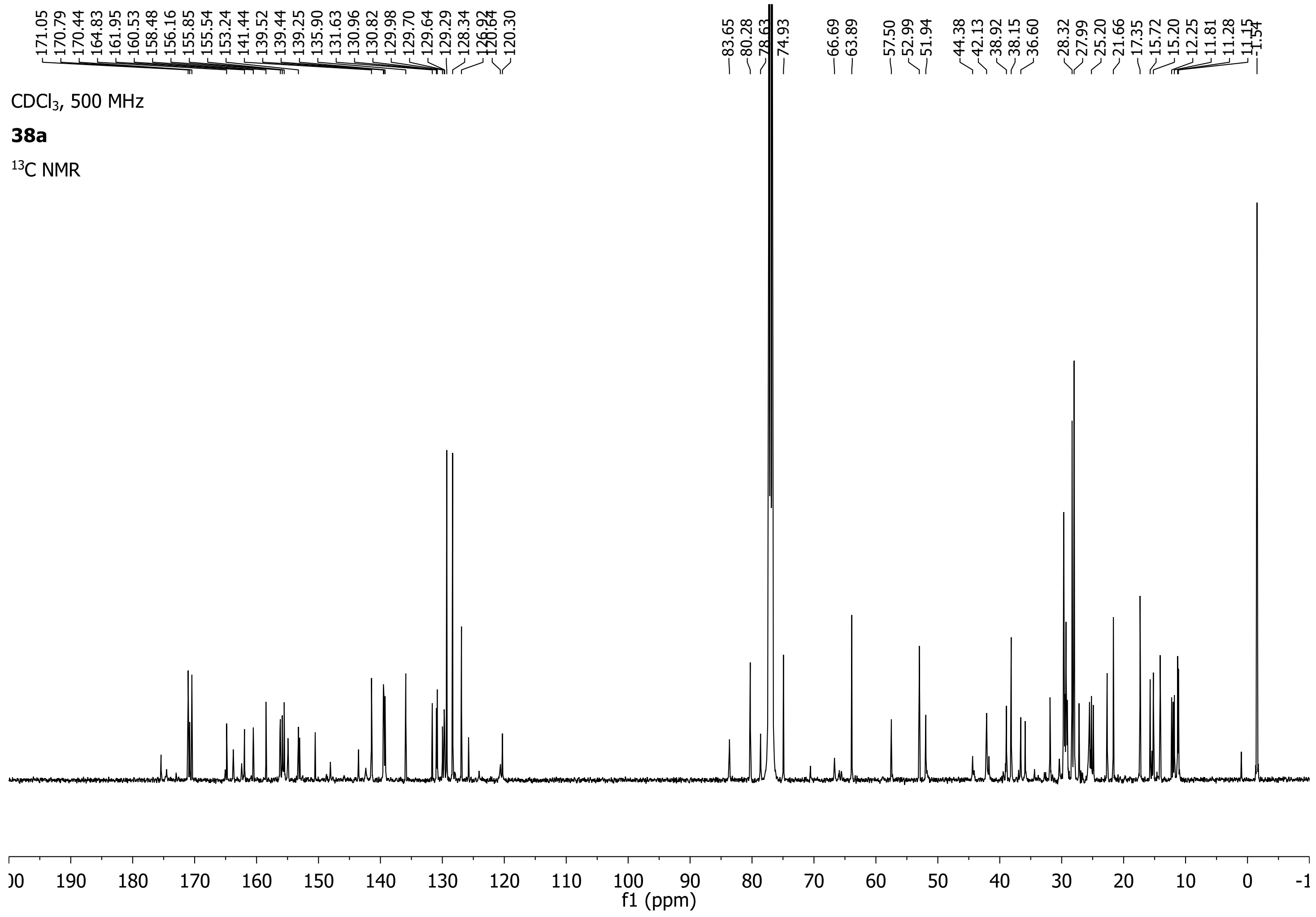
171.05
170.79
170.44
164.83
161.95
160.53
158.48
156.16
155.85
155.54
153.24
141.44
139.52
139.44
139.25
135.90
131.63
130.96
130.82
129.98
129.70
129.64
129.29
128.34
126.92
120.64
120.30
83.65
80.28
78.63
74.93
66.69
63.89
57.50
52.99
51.94
44.38
42.13
38.92
38.15
36.60
28.32
27.99
25.20
21.66
17.35
15.72
15.20
12.25
11.81
11.28
11.15
1.54
CDCl3, 500 MHz
38a
13C NMR
f1 (ppm)

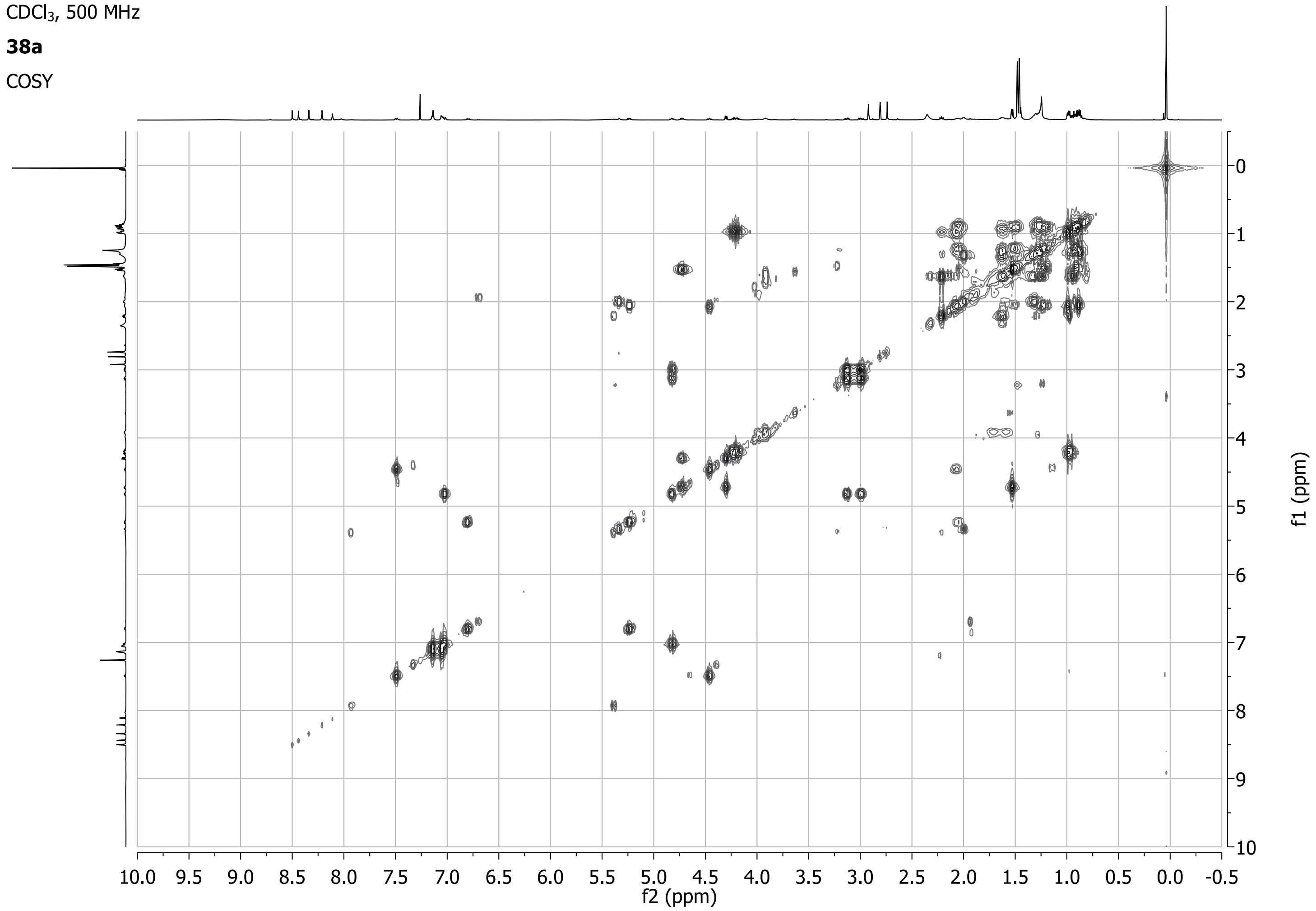
CDCl3, 500 MHz
38a
COSY
f1 (ppm)
f2 (ppm)

CDCl$_3$, 500 MHz

38a

HSQC

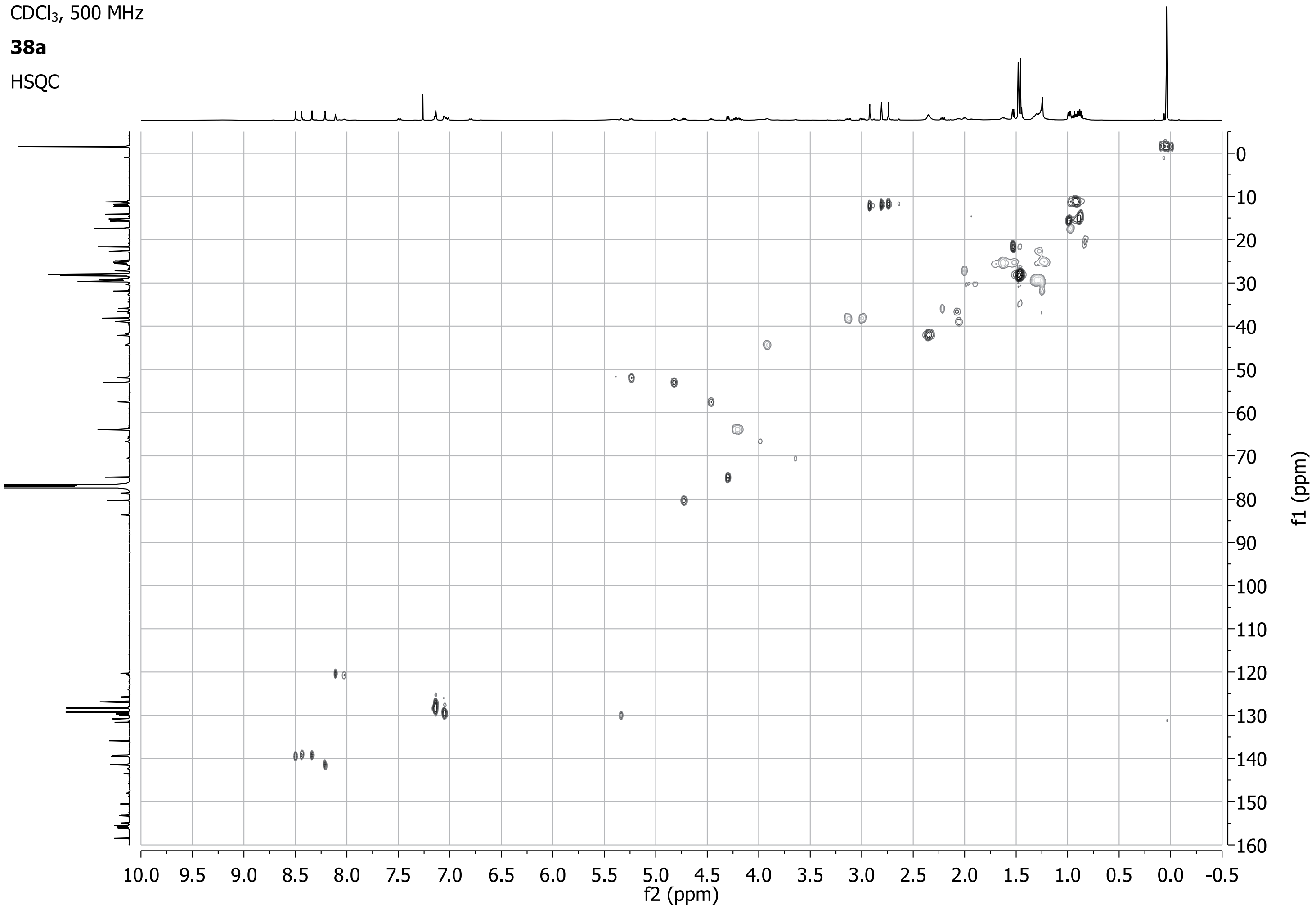

CDCl$_3$, 500 MHz

38a

HMBC

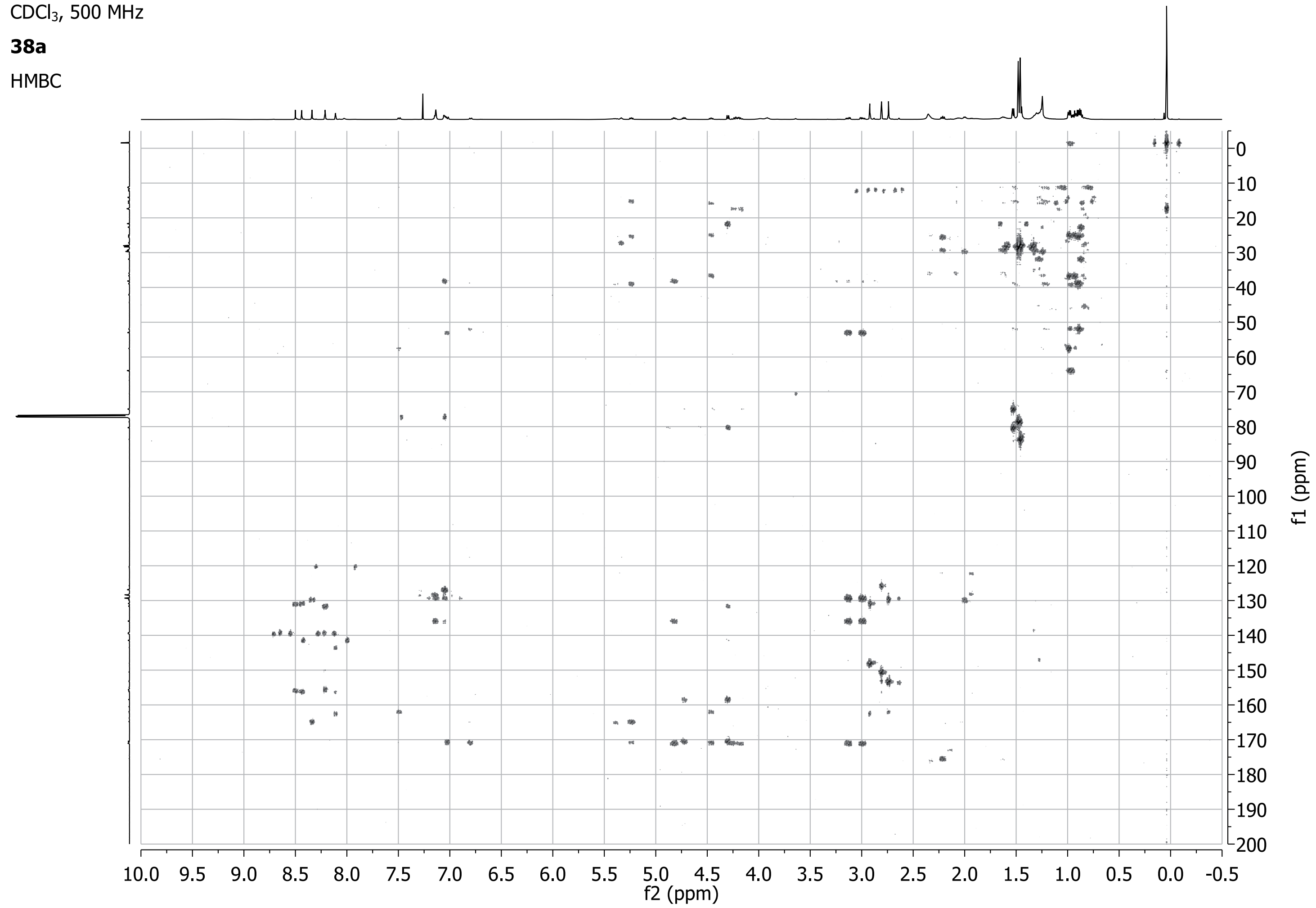

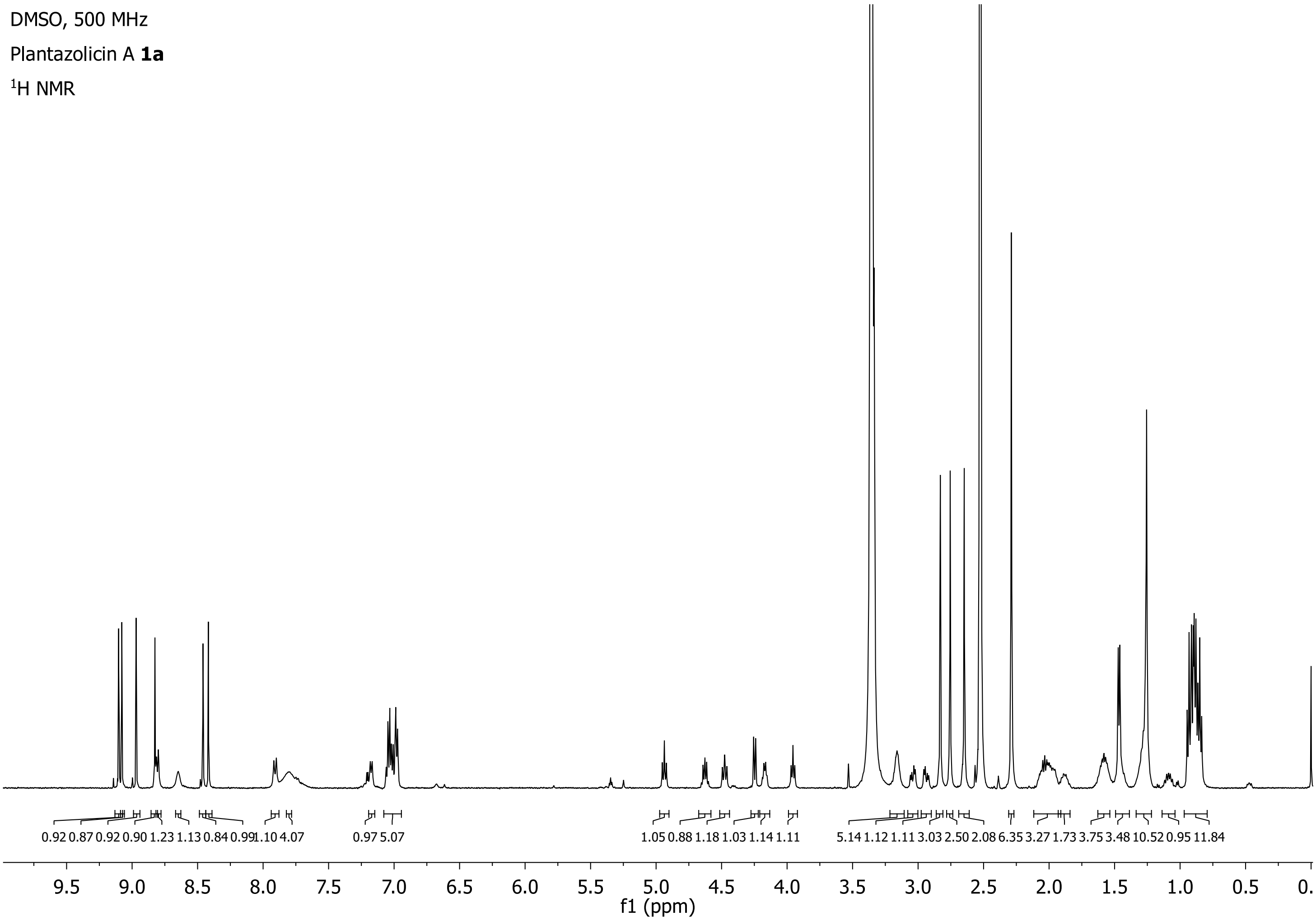
DMSO, 500 MHz
Plantazolicin A **1a**
^{1}H NMR
0.92 0.87 0.92 0.90 1.23 1.13 0.84 0.99 1.10 4.07
0.97 5.07
1.05 0.88 1.18 1.03 1.14 1.11
5.14 1.12 1.11 3.03 2.50 2.08 6.35 3.27 1.73 3.75 3.48 10.52 0.95 11.84
9.5 9.0 8.5 8.0 7.5 7.0 6.5 6.0 5.5 5.0 4.5 4.0 3.5 3.0 2.5 2.0 1.5 1.0 0.5
f1 (ppm)

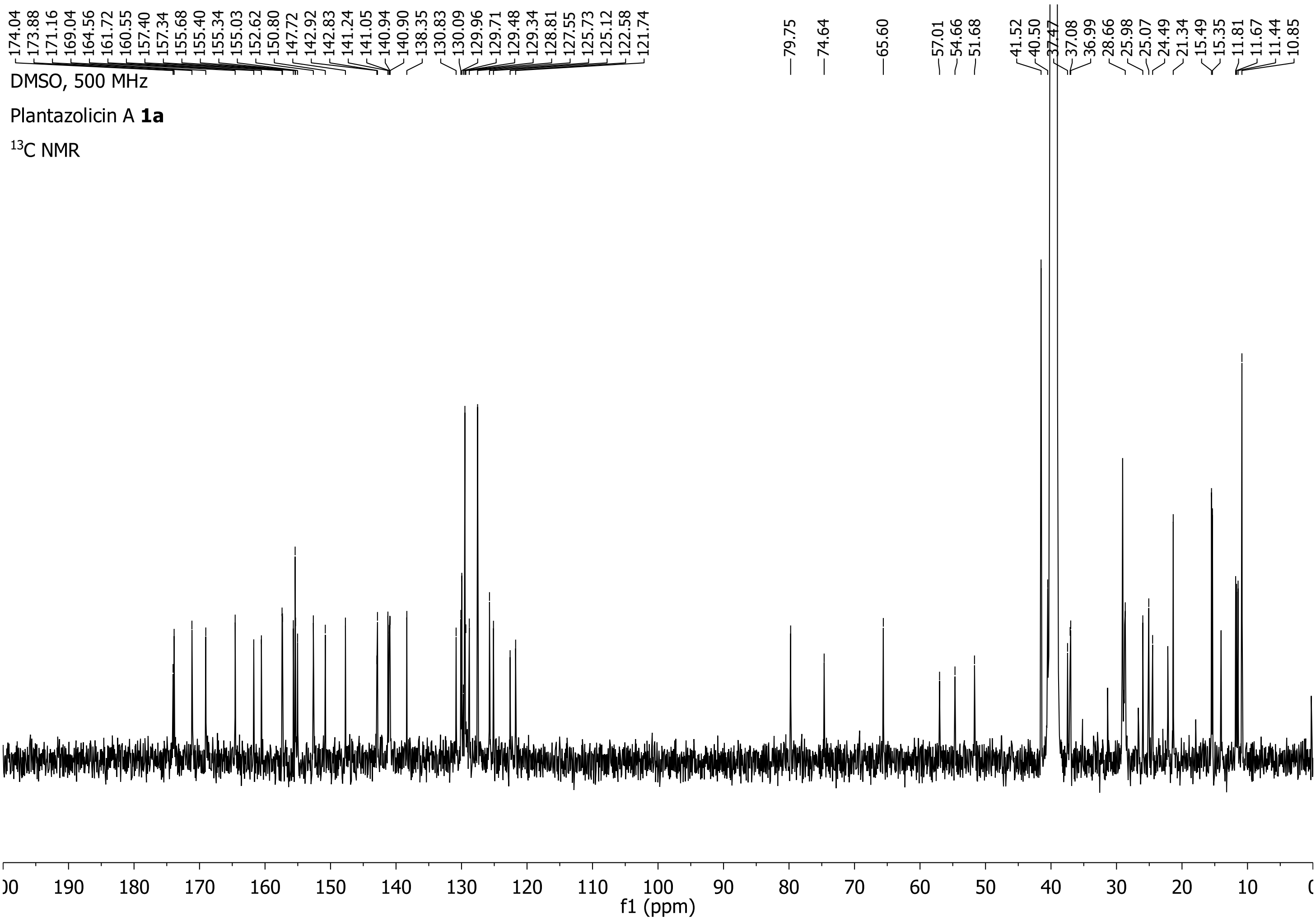
174.04 173.88 171.16 169.04 164.56 161.72 160.55 157.40 157.34 155.68 155.40 155.34 155.03 152.62 150.80 147.72 142.92 142.83 141.24 141.05 140.94 140.90 138.35 130.83 130.09 129.96 129.71 129.48 129.34 128.81 127.55 125.73 125.12 122.58 121.74
79.75 74.64 65.60 57.01 54.66 51.68 41.52 40.50 37.47 37.08 36.99 28.66 25.98 25.07 24.49 21.34 15.49 15.35 11.81 11.67 11.44 10.85
DMSO, 500 MHz
Plantazolicin A **1a**
^{13}C NMR
190 180 170 160 150 140 130 120 110 100 90 80 70 60 50 40 30 20 10
f1 (ppm)

DMSO, 500 MHz

Plantazolicin A **1a**

COSY

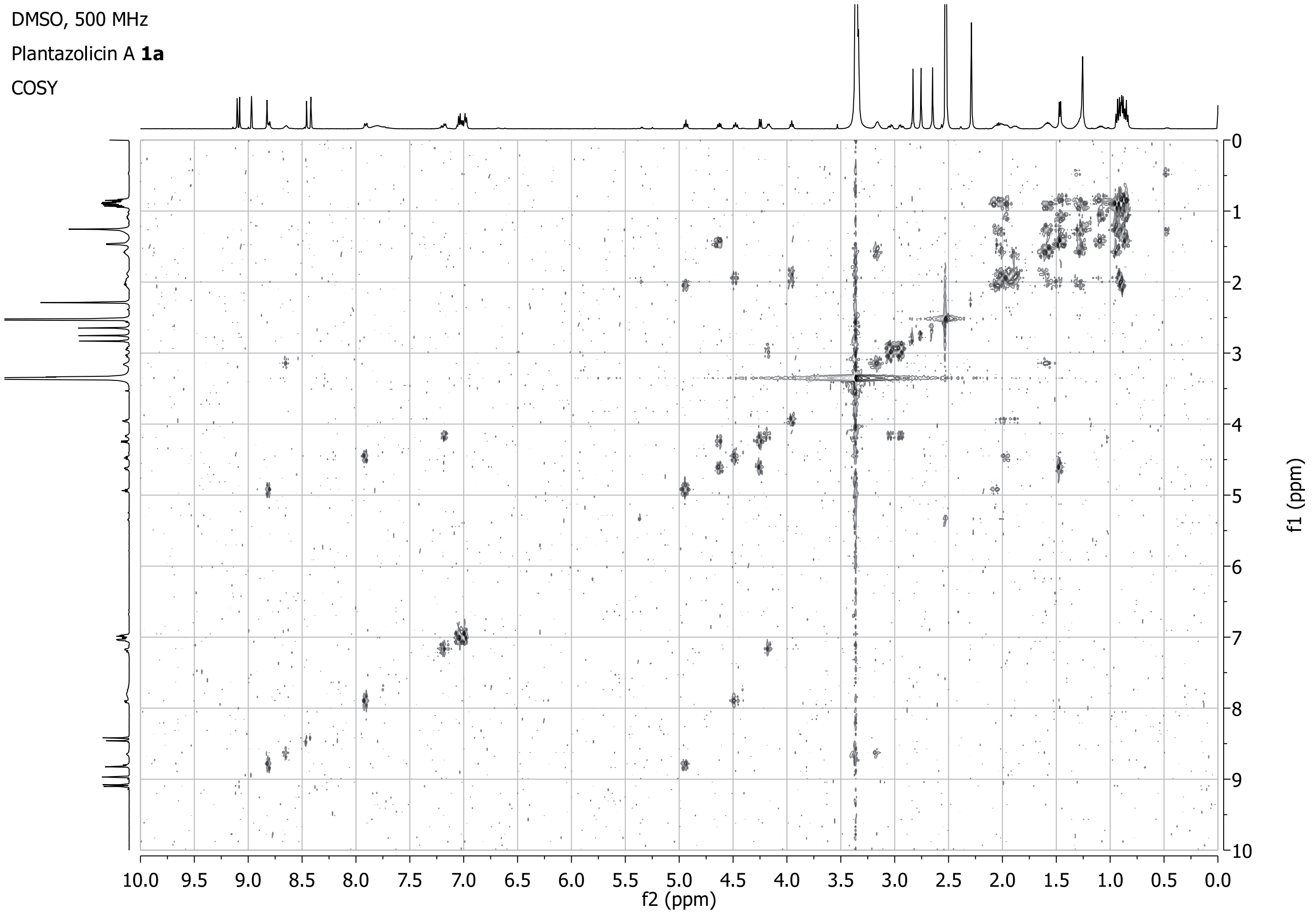

DMSO, 500 MHz

Plantazolicin A **1a**

HSQC

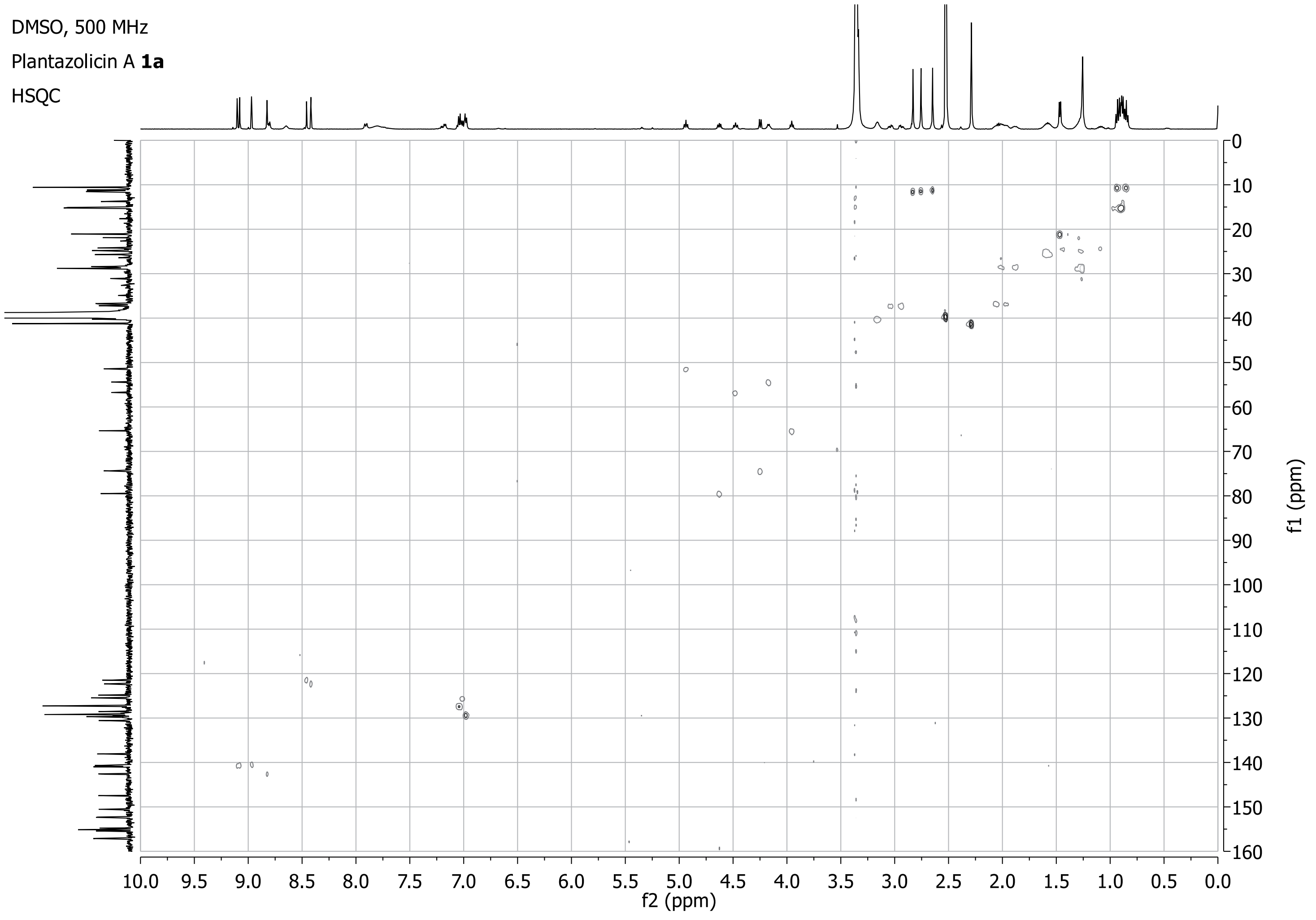

DMSO, 500 MHz

Plantazolicin A **1a**

HMBC

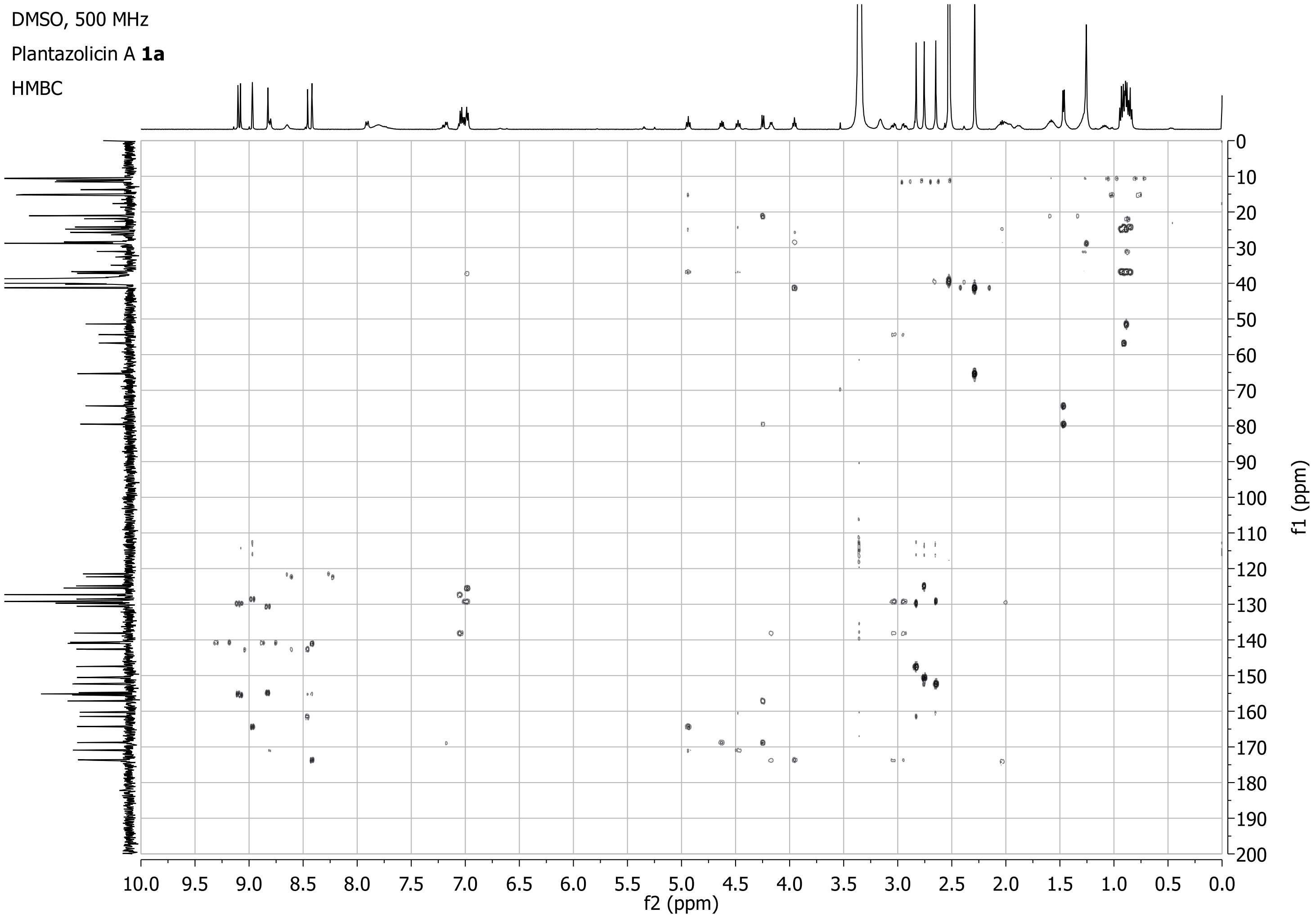

DMSO, 500 MHz

Plantazolicin A **1a**

NOESY

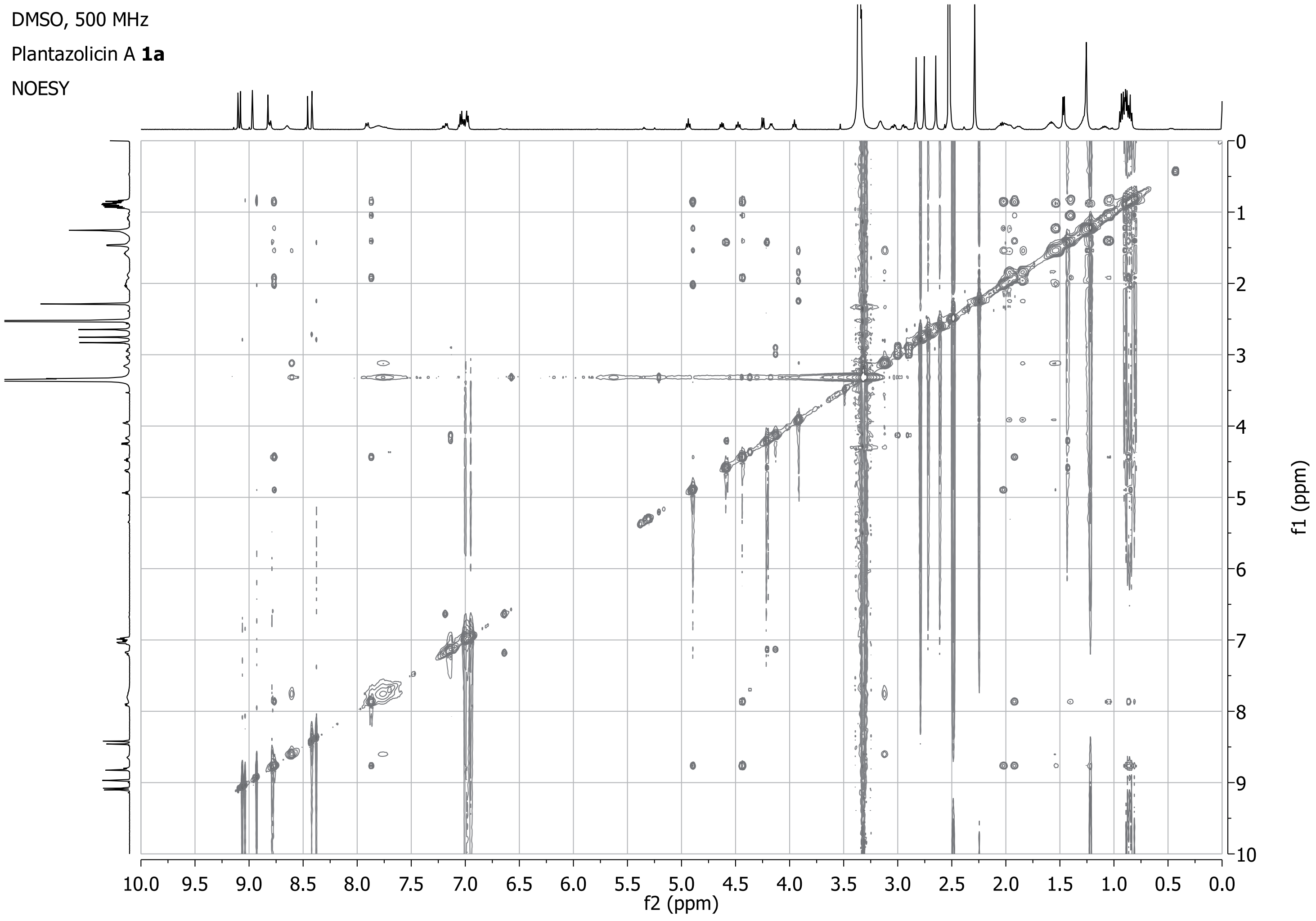

CDCl$_3$, 400 MHz

37b

^{1}H NMR

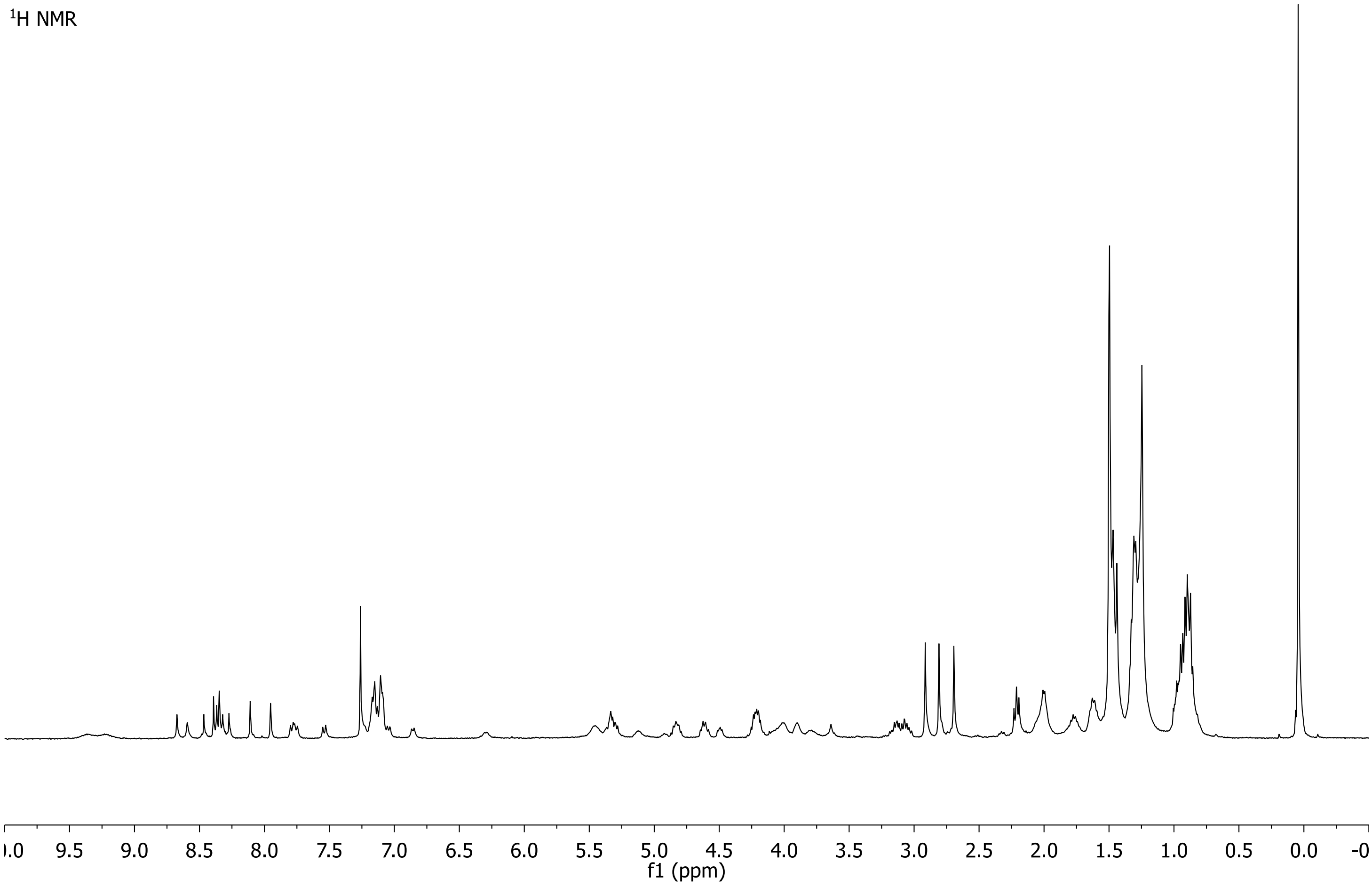

CDCl$_3$, 500 MHz

38b

^{1}H NMR

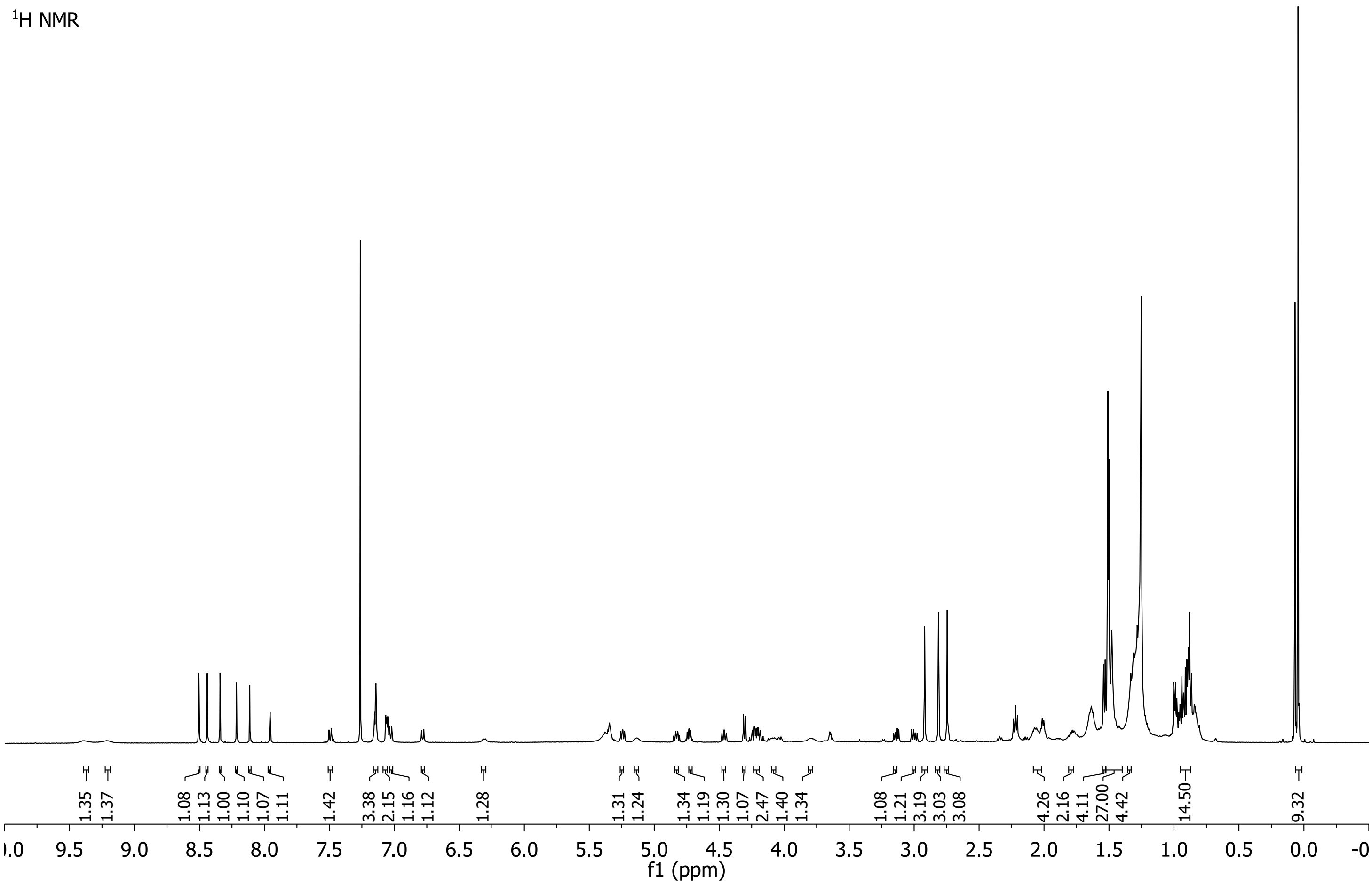

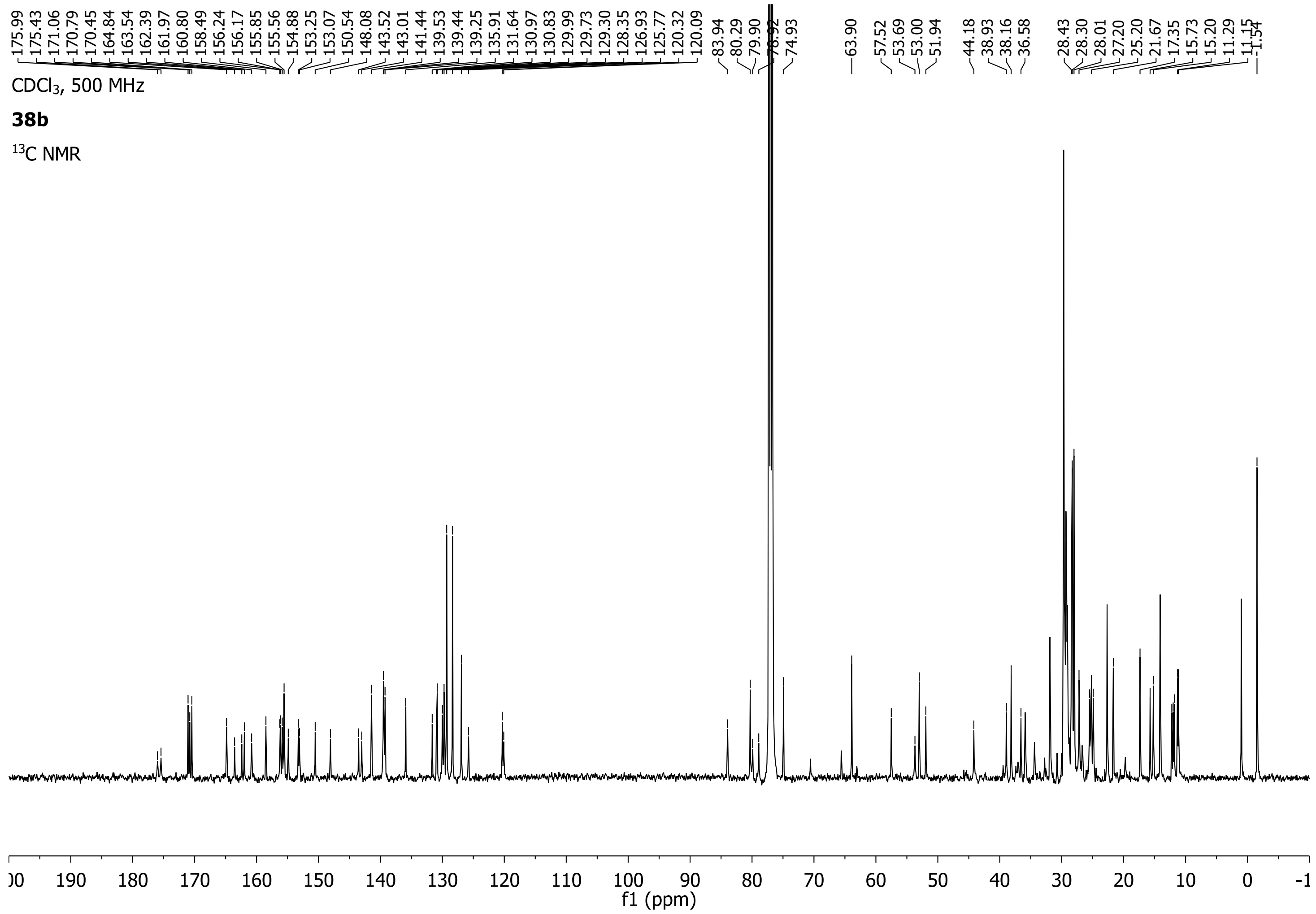
175.99
175.43
171.06
170.79
170.45
164.84
163.54
162.39
161.97
160.80
158.49
156.24
156.17
155.85
155.56
154.88
153.25
153.07
150.54
148.08
143.52
143.01
141.44
139.53
139.44
139.25
135.91
131.64
130.97
130.83
129.99
129.73
129.30
128.35
126.93
125.77
120.32
120.09
83.94
80.29
79.90
74.93
63.90
57.52
53.69
53.00
51.94
44.18
38.93
38.16
36.58
28.43
28.30
28.01
27.20
25.20
21.67
17.35
15.73
15.20
11.29
11.15
1.54
CDCl3, 500 MHz
38b
13C NMR
f1 (ppm)

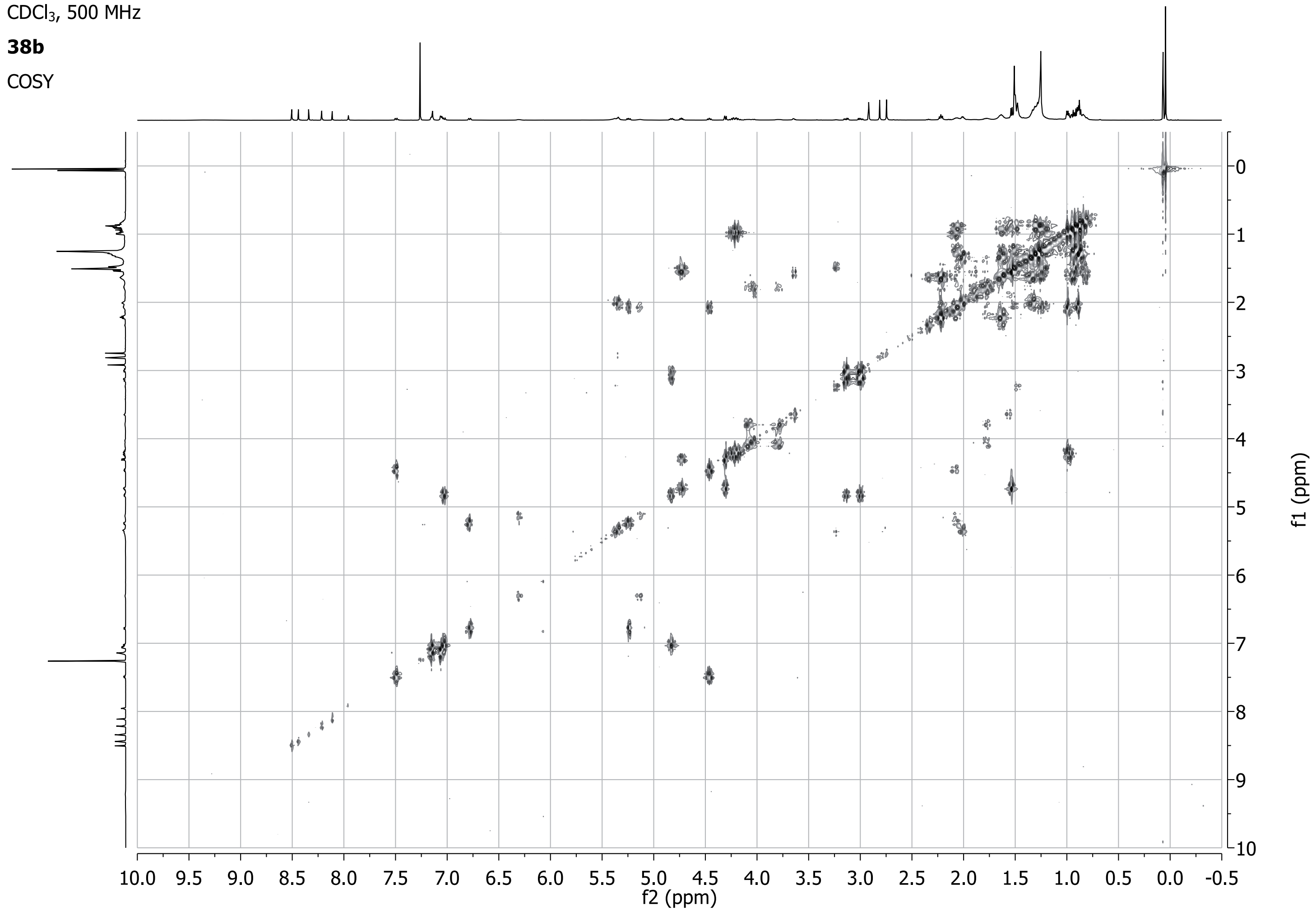
CDCl3, 500 MHz
38b
COSY
f2 (ppm)
f1 (ppm)

CDCl$_3$, 500 MHz

38b

HSQC

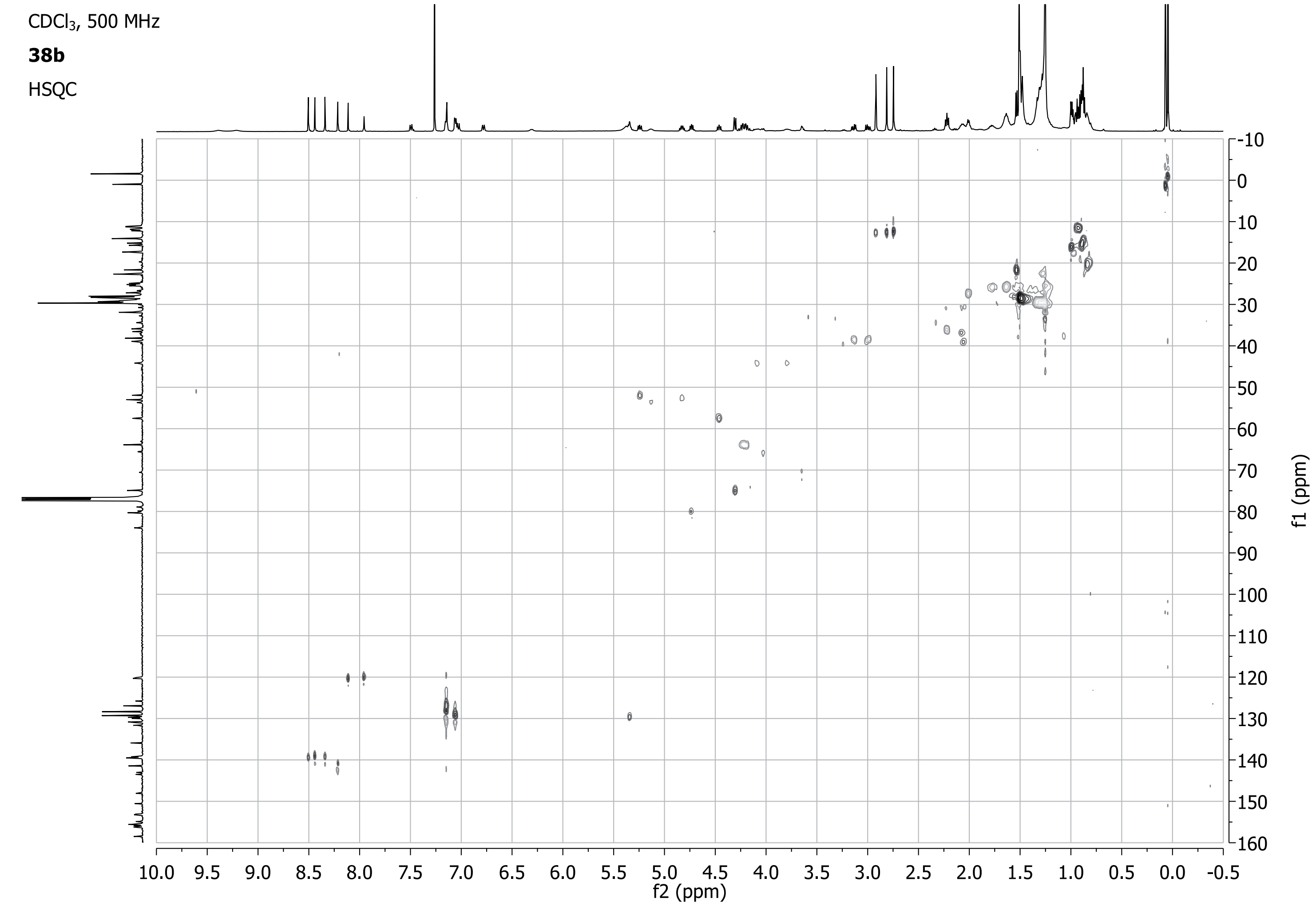

CDCl$_3$, 500 MHz

38b

HMBC

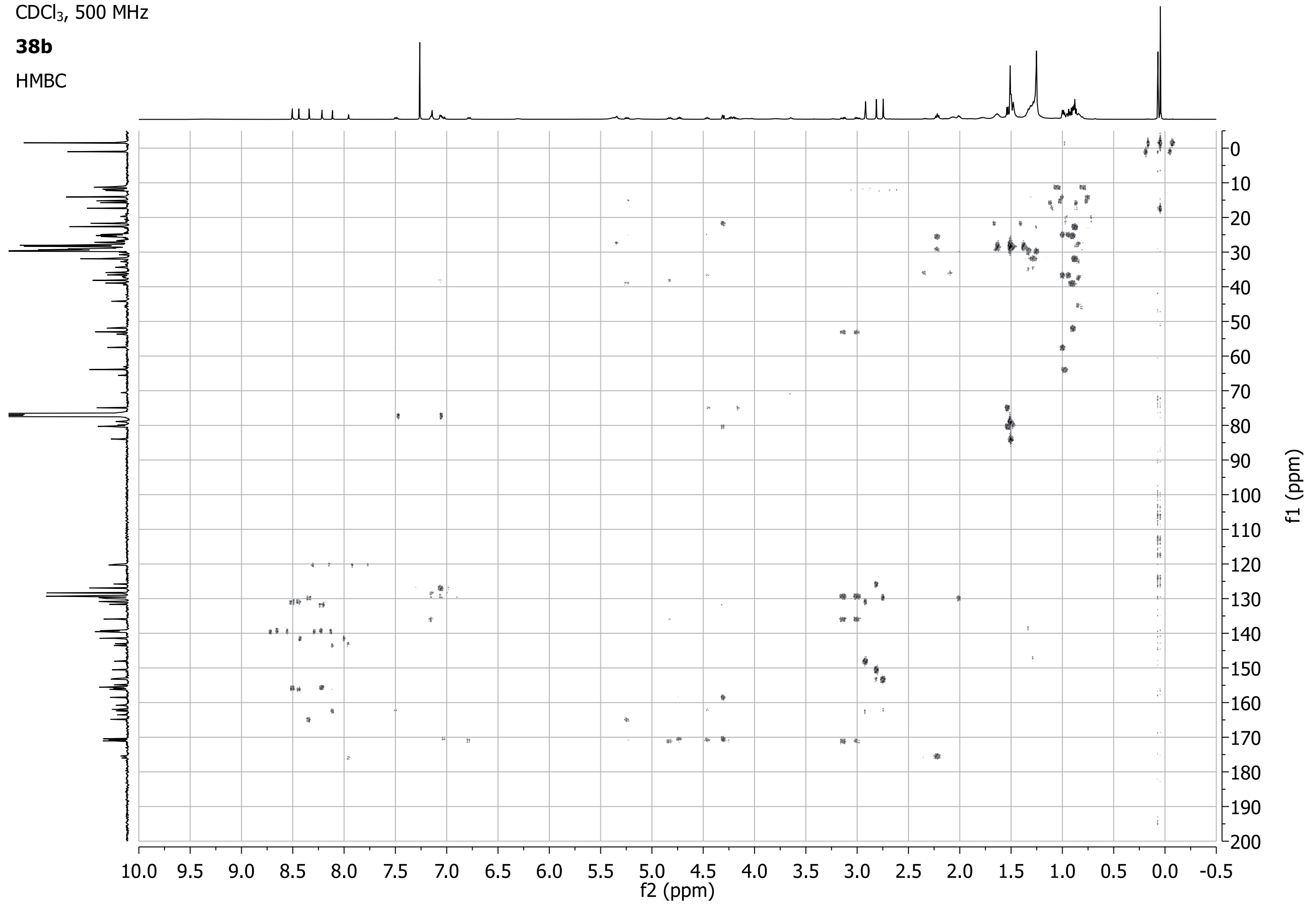

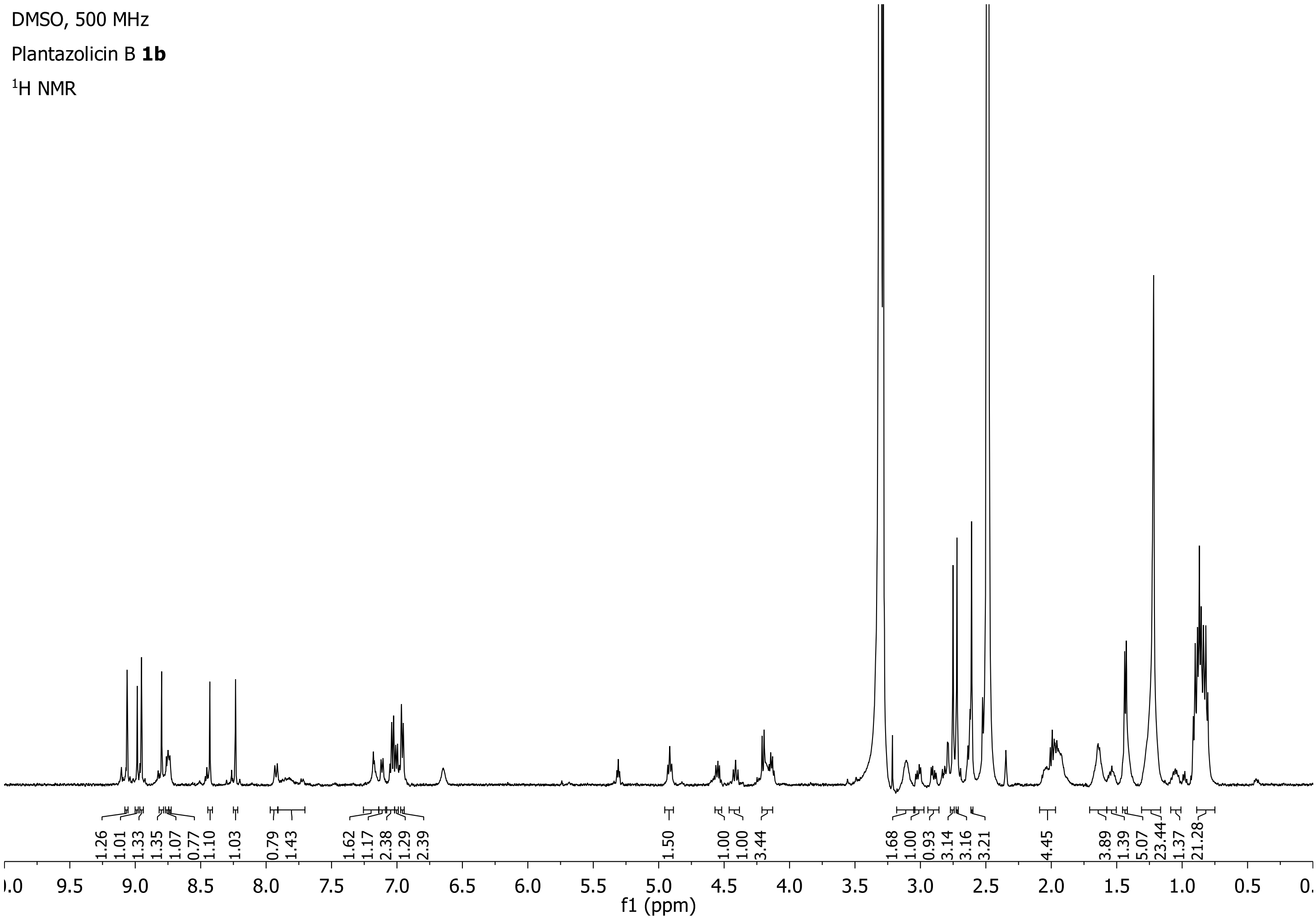
DMSO, 500 MHz
Plantazolicin B **1b**
^{1}H NMR
f1 (ppm)

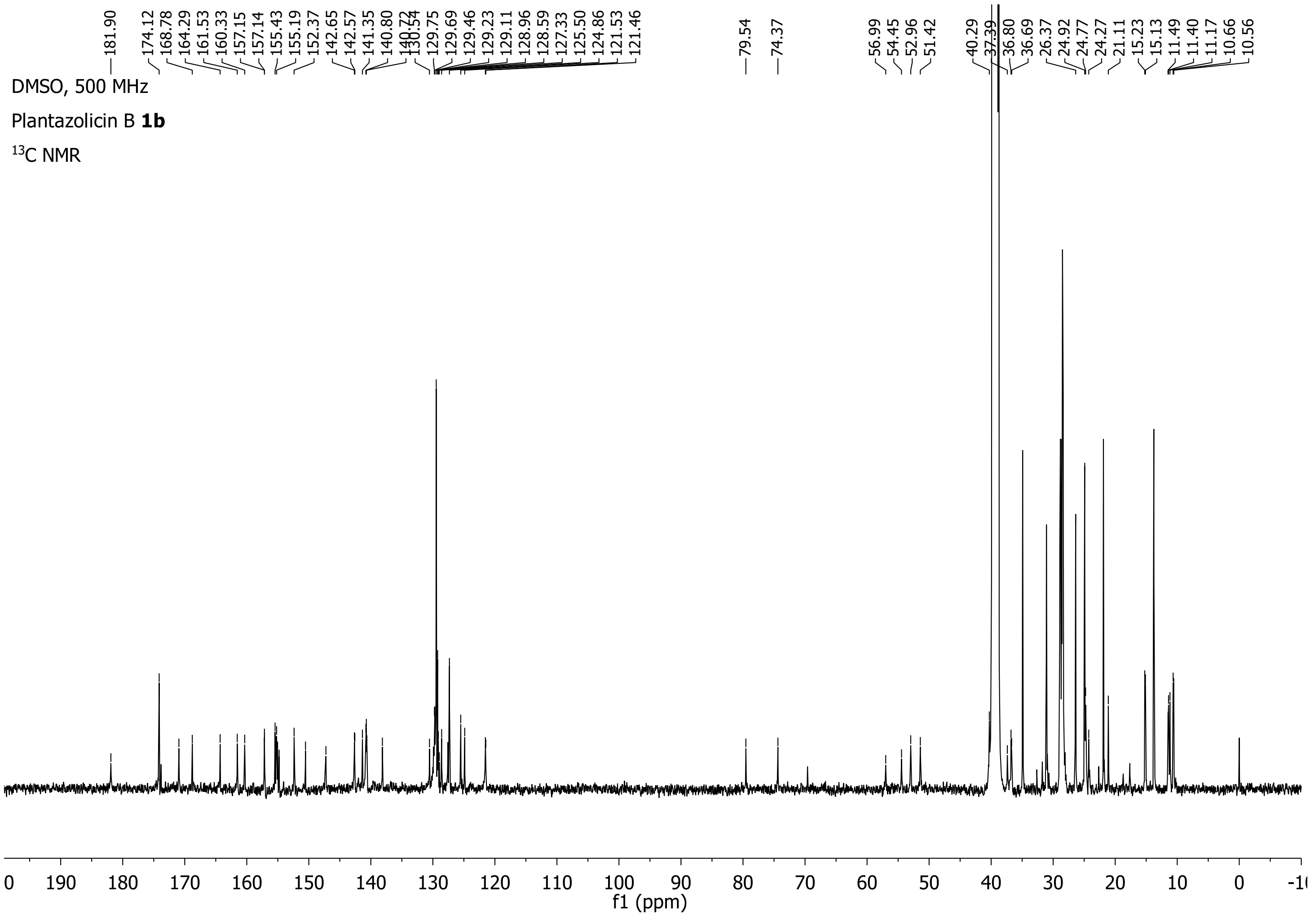
DMSO, 500 MHz
Plantazolicin B **1b**
^{13}C NMR
181.90
174.12
168.78
164.29
161.53
160.33
157.15
157.14
155.43
155.19
152.37
142.65
142.57
141.35
140.80
140.72
130.54
129.75
129.69
129.46
129.23
129.11
128.96
128.59
127.33
125.50
124.86
121.53
121.46
79.54
74.37
56.99
54.45
52.96
51.42
40.29
37.39
36.80
36.69
26.37
24.92
24.77
24.27
21.11
15.23
15.13
11.49
11.40
11.17
10.66
10.56
f1 (ppm)

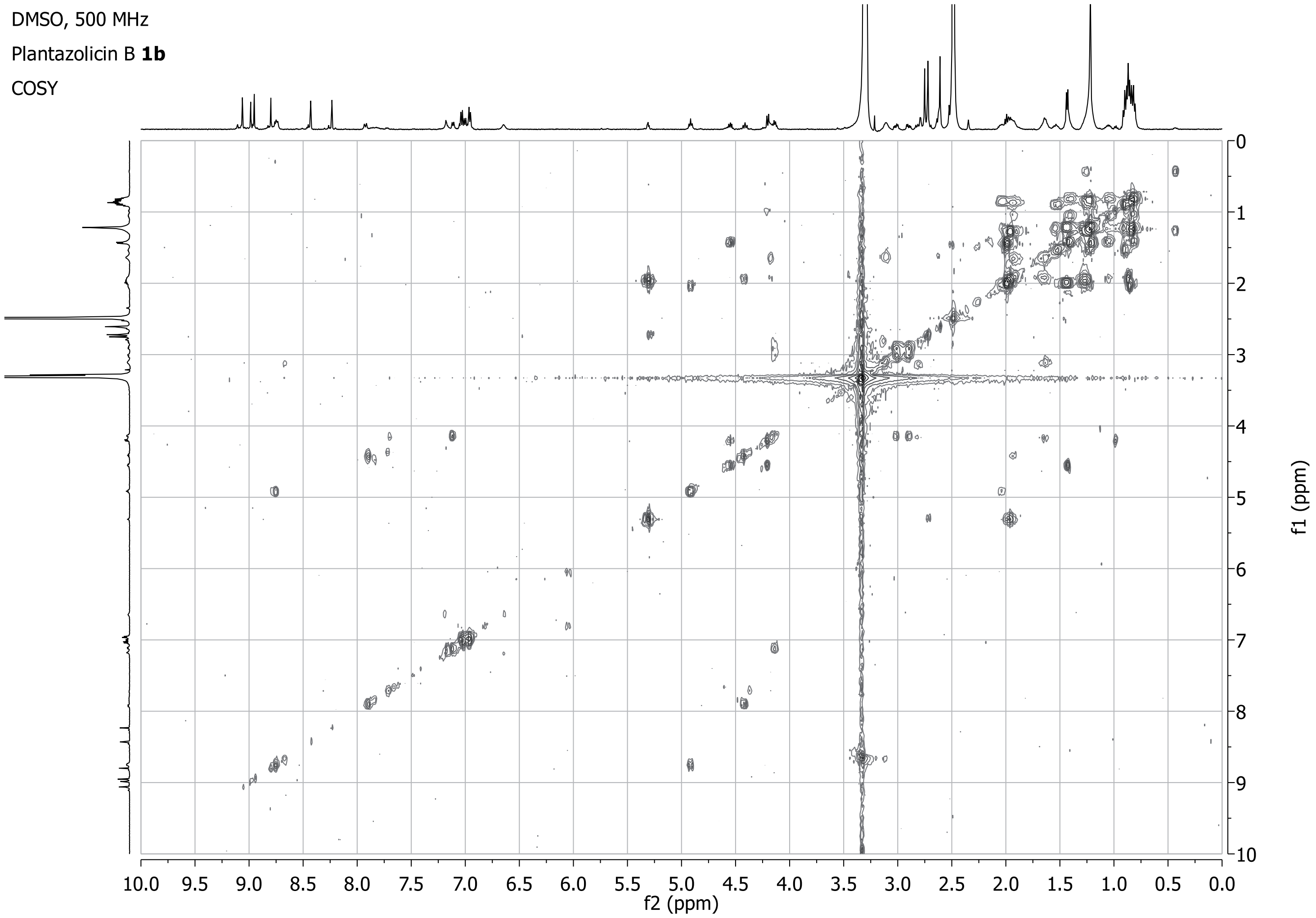

DMSO, 500 MHz
Plantazolicin B **1b**
COSY
f1 (ppm)
f2 (ppm)

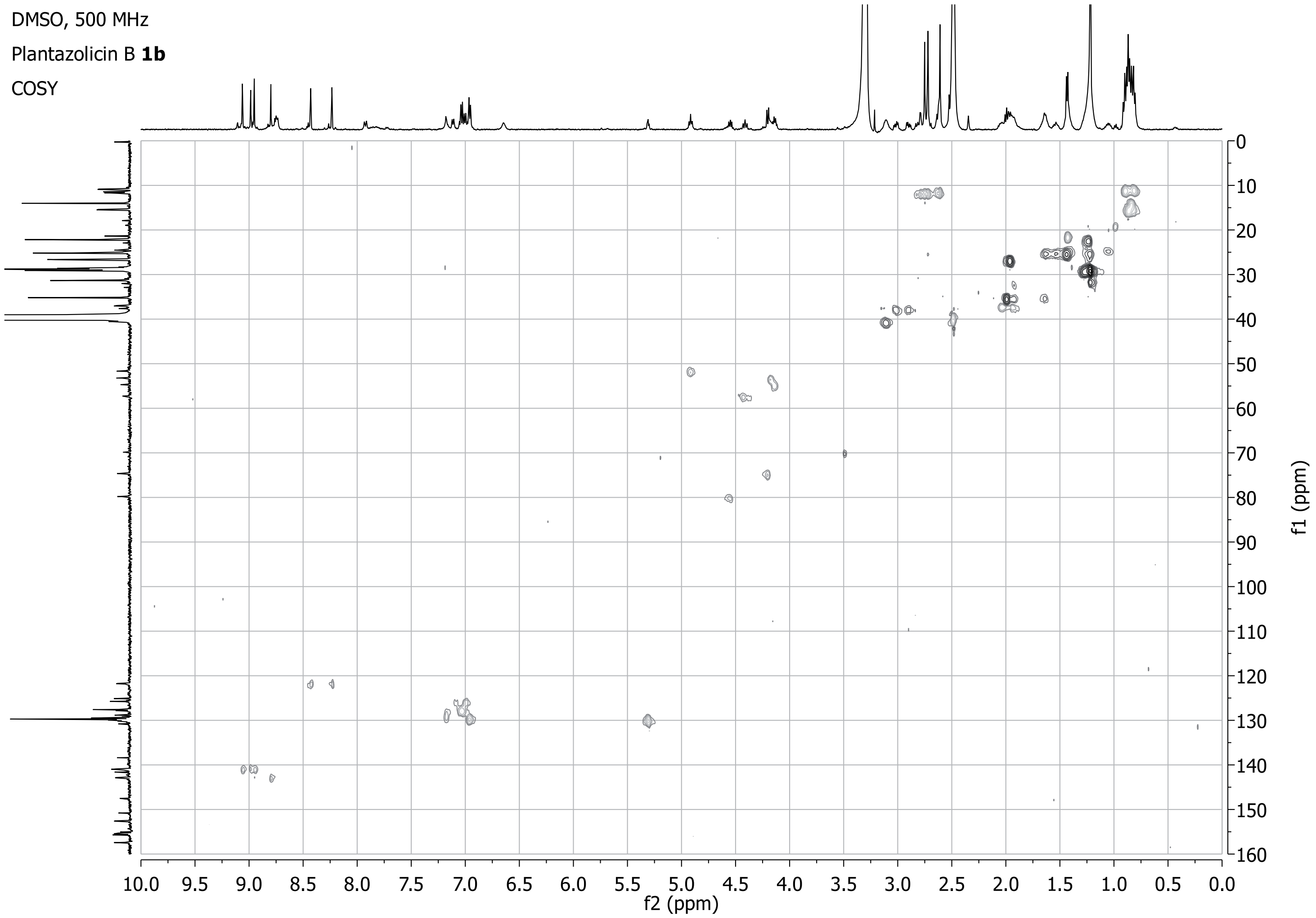

DMSO, 500 MHz
Plantazolicin B **1b**
COSY
f1 (ppm)
f2 (ppm)

DMSO, 500 MHz

Plantazolicin B **1b**

COSY

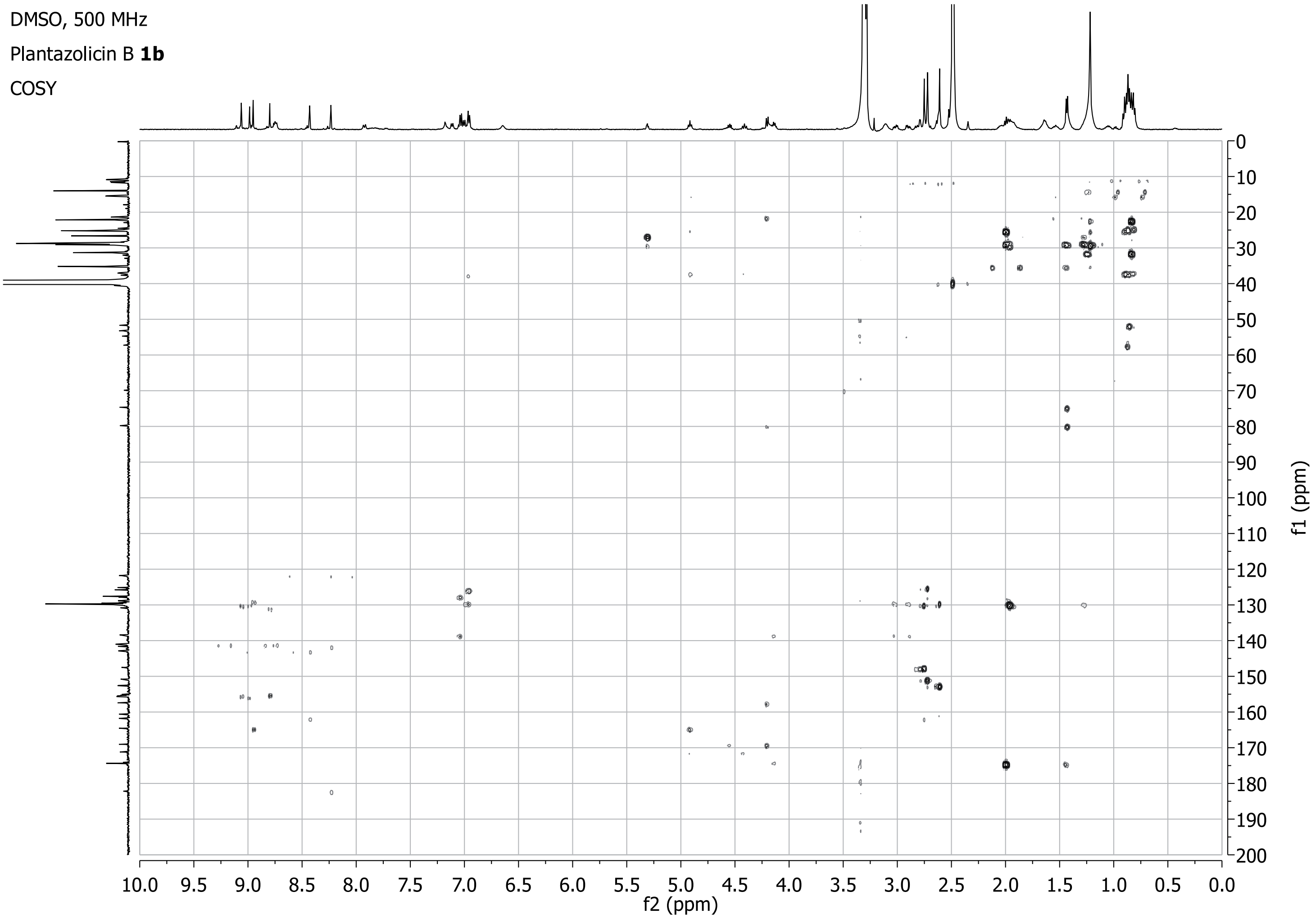

15. Comparison of synthesised and natural Plantazolicin A

15.1. Comparison of 1H NMR for Published[1], Natural[2], Previously Synthesised[3] and Synthetic[4] Plantazolicin A

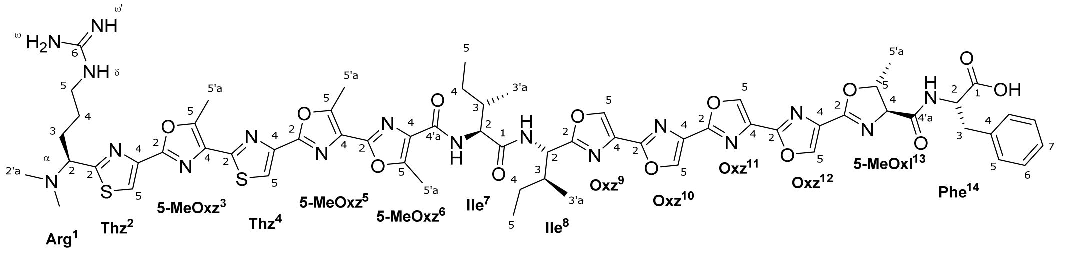

Residue	Atom	^{1}H NMR (500 MHz^a or 700 MHz^b, DMSO) δ and multiplicity			
		Published[1,a]	Natural (from sample provided)[2,a]	Previously Synthesised[3,b]	Synthetic (this work)[4,a]
Arg^1	NαC$\underline{H}_3$	2.23 (s)	2.29 (s)	2.25 (s)	2.29 (s)
	C2$\underline{H}$	3.93 (t, *J* = 6.1 Hz)	3.96 (t, J = 6.9 Hz	3.92 (t, *J* = 7.2 Hz)	3.96 (t, *J* = 7.1 Hz),
	C3$\underline{H}_2$	1.52 (m)	1.93 – 1.83 (m,1H) and peak at ~2.00 ppm$^\#$	1.87 (m), 1.97 (m)	1.93 – 1.84 (m, 1H), 2.12 – 1.91 (m, 1 out of 3H)
	C4$\underline{H}_2$	0.87, 1.24 (m)	1.64 – 1.53 (m, 2 out of 3H)	1.11	1.63 – 1.54 (m, 2 out of 3H)
	C5$\underline{H}_2$	1.85, 1.98 (m)	3.17 – 3.13 (m*)	3.12 (m)	3.19 – 3.13 (m)
	Nδ$\underline{H}$	9.23	8.55 (br. s.)	n.d.	8.65 (br. s.)
	Nω$\underline{H}$	7.67/7.72 (m)	n.d.	n.d.	7.80 (br. s.,)
	Nω′$\underline{H}$	7.67/7.72 (m)	n.d.	n.d.	7.80 (br. s.,)
Thz^2	C5$\underline{H}$	8.41 (s)	8.43 (s)	8.36 (s)	8.42 (s)
5-MeOxz^3	C5′a$\underline{H}_3$	2.83 (s)	2.83 (s)	2.79 (s)	2.83 (s)
Thz^4	C5$\underline{H}$	8.47 (s)	8.47 (s)	8.43 (s)	8.46 (s)
5-MeOxz^5	C5′a$\underline{H}_3$	2.75 (s)	2.76 (s)	2.68 (s)	2.75 (s)
5-MeOxz^6	C5′a$\underline{H}_3$	2.64 (s)	2.67 (s)	2.65 (s)	2.65 (s)
Ile^7	N$\underline{H}$	7.89 (d, *J* = 9.2 Hz)	7.92 (d, *J* = 10.5 Hz)	7.67 (d, *J* = 8.9 Hz)	7.91 (d, *J* = 9.4 Hz)
	C2$\underline{H}$	4.48 (t, *J* = 8.2 Hz)	4.47 (t, J = 8.5Hz)	4.57 (br. t)	4.48 (t, *J* = 8.7 Hz)
	C3$\underline{H}$	1.93 (m)	peak at ~2.00 ppm$^\#$	1.88 (m)	2.12 – 1.91 (m, 1 of 3H)
	C3′a$\underline{H}_3$	1.05 (d, *J* = 6.6 Hz)	0.97 – 0.80 (m, 3 of 12H)	1.39	0.97 – 0.79 (m, 3 of 12H)
	C4$\underline{H}_2$	0.87 (m)	*Either* 1.64 – 1.53 (m, 1 out of 3H) and peak at ~1.26 ppm$^\#$ *or* peak at ~1.45$^\#$ and 1.11 – 1.03 (m*)	0.88 (m)	*Either* 1.63 – 1.54 (m, 1 of 3H) and 1.32 – 1.24 (m 1H) *or* 1.49 – 1.41 (m, 1H) and 1.09 (dt, *J* = 14.6, 7.7 Hz, 1H)
	C5$\underline{H}_3$	0.85 (m)	0.97 – 0.80 (m, 3 of 12H)	0.85 (m)	0.97 – 0.79 (m, 3 of 12H)
Ile^8	N$\underline{H}$	8.78 (d, *J* = 7.1 Hz)	8.79 (d, *J* = 7.8 Hz)	8.91 (br. s)	8.81 (d, *J* = 7.5 Hz)
	C2$\underline{H}$	4.93 (t, *J* = 7.8 Hz)	4.93 (t, J = 7.9 Hz)	4.98 (br. t)	4.94 (t, *J* = 7.8 Hz)
	C3$\underline{H}$	2.07 (m)	peak at ~2.00 ppm$^\#$	2.04 (m)	2.12 – 1.91 (m, 1 of 3H)
	C3′a$\underline{H}_3$	1.05 (d, *J* = 6.6 Hz)	0.97 – 0.80 (m, 3 of 12H)	1.12	0.97 – 0.79 (m, 3 of 12H)
	C4$\underline{H}_2$	0.84	*Either* 1.64 – 1.53 (m, 1 out of 3H) and peak at ~1.26 ppm$^\#$ *or* peak at ~1.45$^\#$ and 1.11 – 1.03 (m*)	0.86 (m)	*Either* 1.63 – 1.54 (m, 1 of 3H) and 1.32 – 1.24 (m 1H) *or* 1.49 – 1.41 (m, 1H) and 1.09 (dt, *J* = 14.6, 7.7 Hz, 1H)
	C5$\underline{H}_3$	0.85 (m)	0.97 – 0.80 (m, 3 of 12H)	0.85 (m)	0.97 – 0.79 (m, 3 of 12H)

Oxz[9]	C5H	8.96 (s)	8.97 (s)	8.93 (s)	8.97 (s)
Oxz[10]	C5H	9.08 (s)	9.08 (s)	8.97 (s)	9.08 (s)
Oxz[11]	C5H	9.11 (s)	9.10 (s)	9.04 (s)	9.10 (s)
Oxz[12]	C5H	8.81 (s)	8.82 (s)	8.76 (s)	8.83 (s)
5-MeOxl[13]	C4H	4.23 (d, *J* = 7.6 Hz)	4.24 (d, J = 7.7Hz	4.26 (m)	4.25 (d, *J* = 7.7 Hz)
	C5H	4.61 (m)	4.65 – 4.60 (m	4.59 (m)	4.67 – 4.58 (m),
	C5′aH_3	1.44 (d, *J* = 6.2 Hz)	peak at ~1.47 ppm[#]	1.43 (d, *J* = 6.2 Hz)	1.47 (d, *J* = 6.2 Hz)
Phe[14]	NH	7.23 (d, *J* = 6.3 Hz)	7.16 (d, *J* = 7.5 Hz)	7.15 (d, *J* = 7.4 Hz)	7.12 (d, *J* = 7.2 Hz)
	C2H	4.10 (t, *J* = 5.3 Hz)	4.16 (q, *J* = 5.6 Hz)	4.12 (m)	4.17 (q, *J* = 5.7 Hz)
	C3H_2	2.92 (d, *J* = 12.3 Hz) and 3.03 (d, *J* = 5.8 Hz)	2.93 (dd, *J* = 13.4, 6.0 Hz) and 3.06 – 3.00 (m*)	2.89 (m), 2.98 (m)	3.04 (dd, *J* = 13.1, 5.4 Hz) and 2.94 (dd, *J* = 13.1, 5.4 Hz)
	C5H (2)	6.96 (d, *J* = 7.4 Hz)	7.06 – 6.95 (m, 2 out of 5H)	6.95 (m)	7.08 – 6.94 (m, 2 out of 5H)
	C6H (2)	7.02 (t, *J* = 7.1 Hz)	7.06 – 6.95 (m, 2 out of 5H)	7.01 (m)	7.08 – 6.94 (m, 2 out of 5H)
	C7H	6.99 (d, 6.9 Hz)	7.06 – 6.95 (m, 1 out of 5H)	6.97 (m)	7.08 – 6.94 (m, 1 out of 5H)

[1] Reported in: Kalyon et al., *Org. Lett.*, **2011**, *13*, (12), 2996 – 2999 (isolation)
[2] Provided by Professor Douglas Mitchell (University of Illinois at Urbana-Campaign), Molohon *et al.*, *ACS Chem. Biol.*, **2011**, *6*, 1037 – 1313
[3] Reported in: Banala et al., *Angew. Chem. Int. Ed.*, **2013**, *52*, 9518 – 9523
[4] This work
* = signal partially obscured
[#] = signal completely obscured
n.d. = not detected

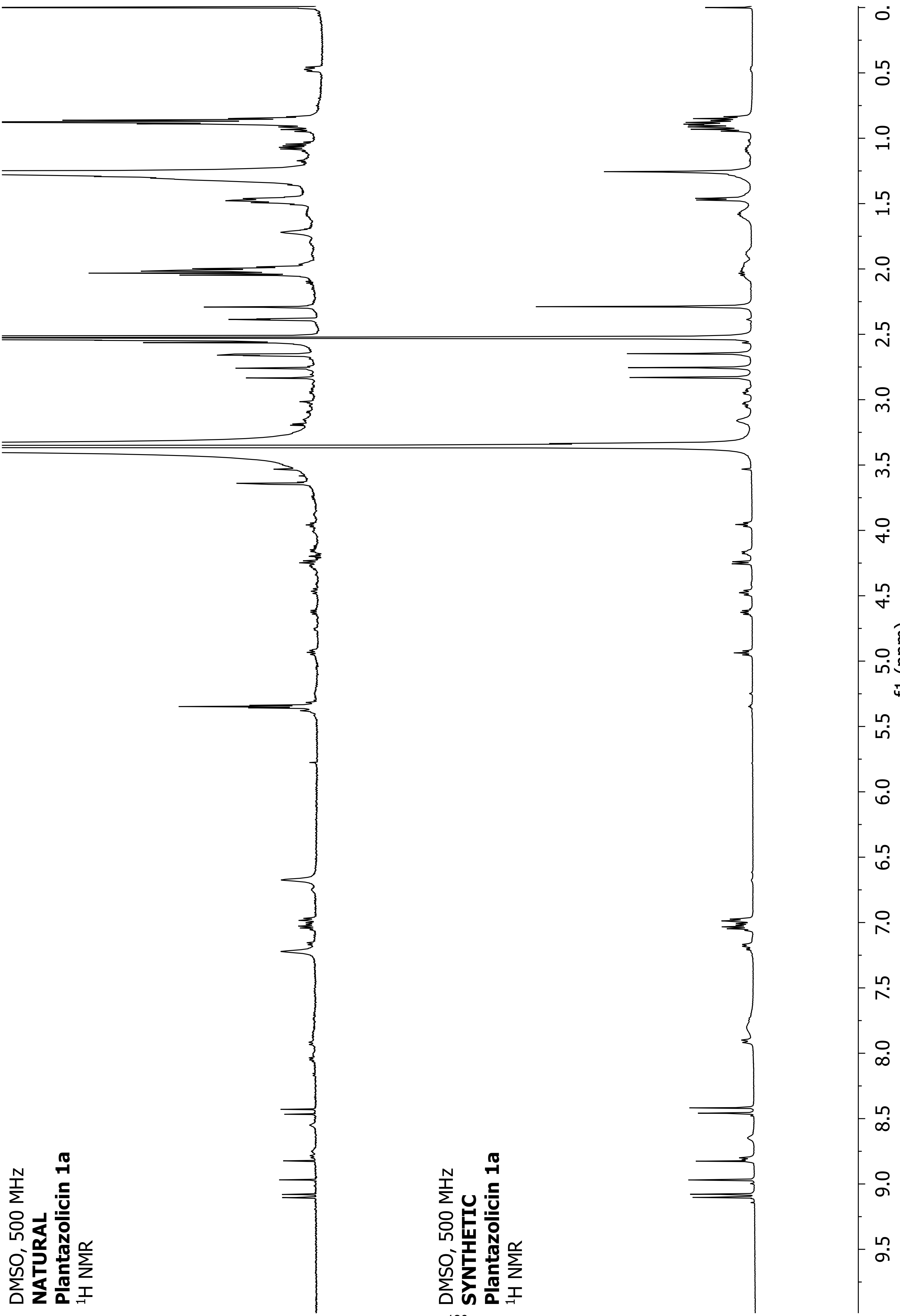
DMSO, 500 MHz
NATURAL
Plantazolicin 1a
1H NMR
DMSO, 500 MHz
SYNTHETIC
Plantazolicin 1a
1H NMR
f1 (ppm)
0.0
0.5
1.0
1.5
2.0
2.5
3.0
3.5
4.0
4.5
5.0
5.5
6.0
6.5
7.0
7.5
8.0
8.5
9.0
9.5

15.2. Comparison of ^{13}C NMR for Published[1] and Previously Synthesised[3] and Synthetic[4] Plantazolicin A

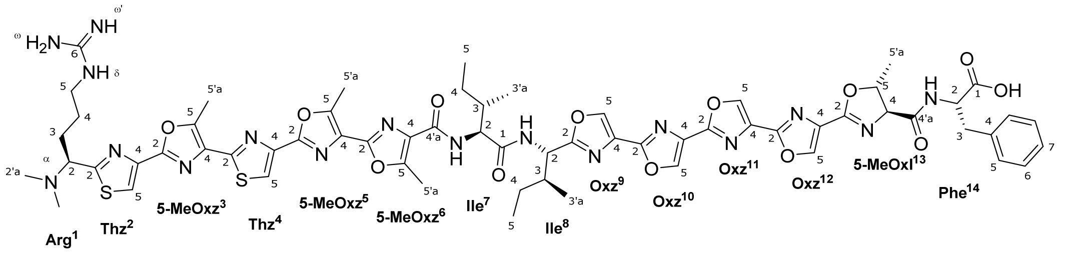

Residue	Atom	^{13}C NMR (500 MHz[a] or 700 MHz[b], DMSO) δ		
		Published[1,a]	Previously Synthesised[3,b]	Synthetic (this work)[4,a]
Arg[1]	NαC	41.1	41.2	41.5
	C2	65.2	65.2	65.6
	C3	24.2	28.9	28.7
	C4	22.2	24.2	26.0
	C5	36.9	40.1	40.5
	C6	n.d.	n.d.	129.7
Thz[2]	C2	172.6	n.d.	173.9
	C4	141.0	n.d.	141.2
	C5	122.2	122.1	121.7
5-MeOxz[3]	C2	173.3	n.d.	155.4
	C4	129.7	n.d.	130.0
	C5	147.4	n.d.	147.7
	C5′a	11.4	11.3	11.8
Thz[4]	C2	161.5	n.d.	161.7
	C4	142.5	n.d.	142.9
	C5	121.5	121.3	122.6
5-MeOxz[5]	C2	n.d.	n.d.	155.3
	C4	124.8	n.d.	125.1
	C5	150.5	n.d.	150.8
	C5′a	11.3	11.4	11.7
5-MeOxz[6]	C2	n.d.	n.d.	157.3
	C4	129.0	n.d.	129.3
	C4′a	n.d.	n.d.	160.6
	C5	152.2	n.d.	152.6
	C5′a	11.0	11.4	11.4
Ile[7]	C1	176.9	n.d.	171.2
	C2	56.4	56.1	57.0
	C3	36.7	37.2	37.0
	C3′a	21.5	26.4	15.5
	C4	22.3	10.7	24.5
	C5	10.6	10.6	10.9
Ile[8]	C2	51.2	51.3	51.7
	C3	36.6	36.9	37.1
	C3′a	21.5	24.2	15.4
	C4	22.6	14.8	25.1
	C5	10.6	13.9	10.9
Oxz[9]	C2	164.2	n.d.	164.6
	C4	n.d.	n.d.	128.8
	C5	140.6	140.5	140.90
Oxz[10]	C2	155.2	n.d.	155.7
	C4	n.d.	n.d.	130.0
	C5	140.6	140.5	140.94
Oxz[11]	C2	n.d.	n.d.	155.4
	C4	n.d.	n.d.	130.1
	C5	140.8	140.6	141.1
Oxz[12]	C2	154.7	n.d.	155.0
	C4	n.d.	n.d.	130.8

	C5	142.5	144.4	142.8
5-MeOxl[13]	C2	156.9	n.d.	157.4
	C4	74.3	74.5	74.6
	C4′a	168.53	n.d.	169.0
	C5	79.4	79.3	79.8
	C5′a	20.9	21.2	21.3
Phe[14]	C1	n.d.	n.d.	174.0
	C2	54.4	54.3	54.7
	C3	37.1	36.9	37.5
	C4	138.4	n.d.	138.4
	C5 (2)	129.15	129.1	129.5
	C6 (2)	127.1	127.2	127.6
	C7	125.3	125.7	125.7

[1] Reported in: Kalyon et al., *Org. Lett.*, **2011**, *13*, (12), 2996 – 2999 (isolation paper)
[3] Reported in: Banala et al., *Angew. Chem. Int. Ed.*, **2013**, *52*, 9518 – 9523
[4] This work
n.d. = not detected

15.3. Comparison of HPLC trace for Natural[2] and Synthetic[4] Plantazolicin A

[2] Provided by Professor Douglas Mitchel (University of Illinois at Urbana-Campaign),
Molohon *et al.*, *ACS Chem. Biol.*, **2011**, *6*, 1037 – 1313
[4] This work

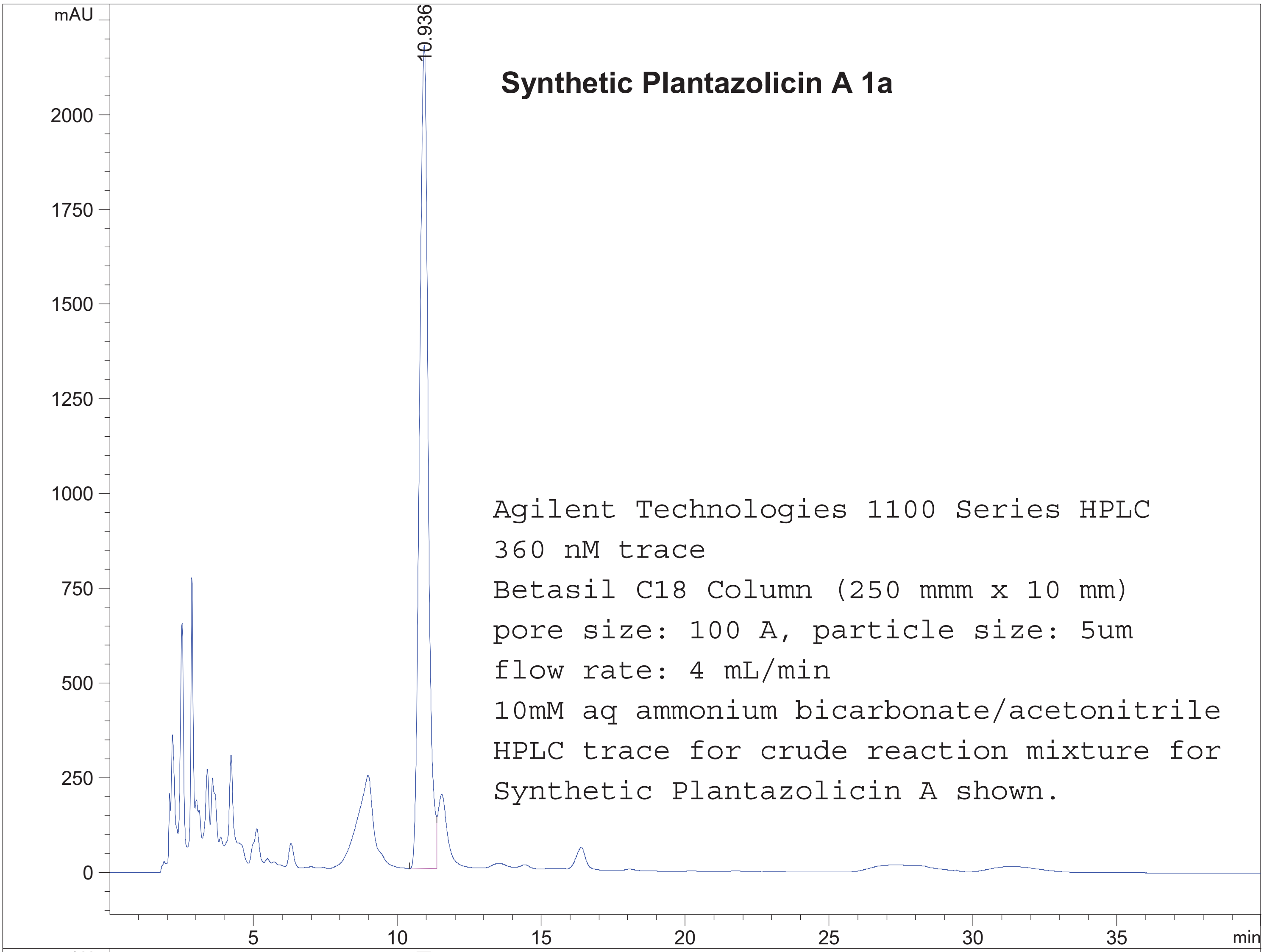
Synthetic Plantazolicin A 1a
10.936
mAU
min
Agilent Technologies 1100 Series HPLC
360 nM trace
Betasil C18 Column (250 mmm x 10 mm)
pore size: 100 A, particle size: 5um
flow rate: 4 mL/min
10mM aq ammonium bicarbonate/acetonitrile
HPLC trace for crude reaction mixture for
Synthetic Plantazolicin A shown.

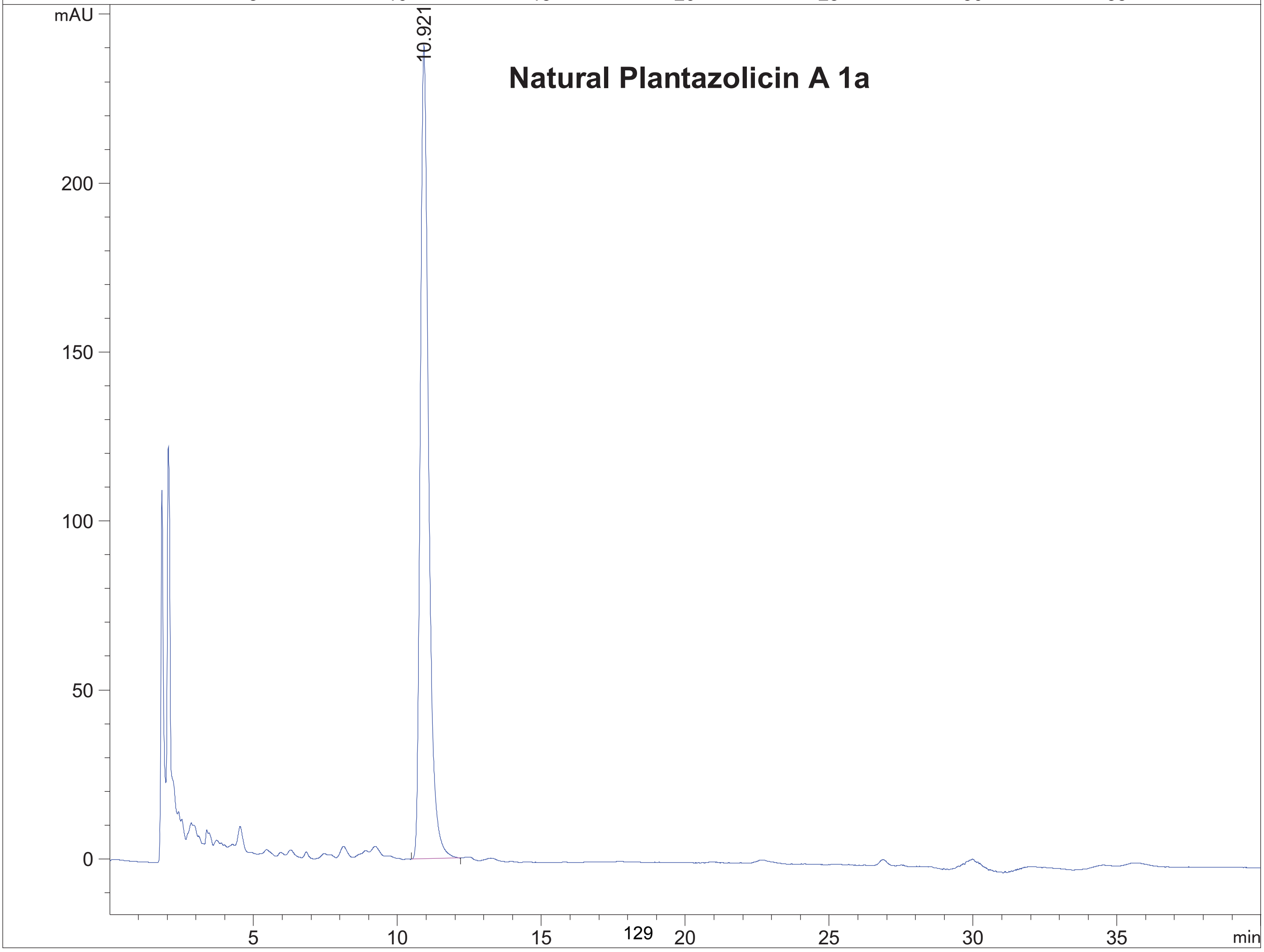
Natural Plantazolicin A 1a
10.921
mAU
min
